# Borane catalysed ring opening and closing cascades of furans leading to silicon functionalized synthetic intermediates

**DOI:** 10.1038/ncomms13431

**Published:** 2016-11-28

**Authors:** Chinmoy K. Hazra, Narasimhulu Gandhamsetty, Sehoon Park, Sukbok Chang

**Affiliations:** 1 Center for Catalytic Hydrocarbon Functionalizations, Institute for Basic Science (IBS), Daejeon 305-701, South Korea; 2 Department of Chemistry, Korea Advanced Institute of Science & Technology (KAIST), Daejeon 305-701, South Korea

## Abstract

The conversion of renewable biomass resources to synthetically valuable chemicals is highly desirable, but remains a formidable challenge in regards to the substrate scope and reaction conditions. Here we present the development of tris(pentafluorophenyl)borane–catalysed conversion of furans via ring-opening and closing cascade processes to afford silicon-functionalized synthetic chemicals under transition metal-free conditions. The furan ring-opening with hydrosilanes is highly efficient (TON up to 2,000) and atom-economical without forming any byproduct to give rise to α-silyloxy-(*Z*)-alkenyl silanes. Additional equivalents of silane smoothly induce a subsequent B(C_6_F_5_)_3_-catalysed cyclization of initially formed olefinic silane compounds to produce *anti*-(2-alkyl)cyclopropyl silanes, another versatile synthon being potentially applicable in the synthesis of natural products and pharmacophores.

## Abstract (web summary)

Furans are attractive staring materials in organic chemistry, due to the ease of functionalisation and sourcing from renewable feedstocks. Here the authors show the ring opening of furans to silane intermediates, followed by further conversion to cyclopropanes and other high value targets.

## Graphical abstract

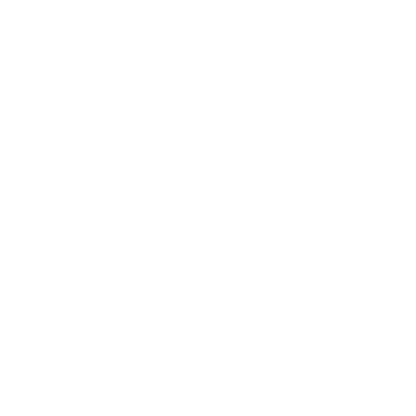

Production of furans and their subsequent transformations have received a great attention^1^,^2^. This research activity can be attributed to the relevance of furan derivatives to the renewable biomass. Thermal dehydration of glucose or fructose, the most abundant monosaccharides, in the presence of acid catalysts provides important nonpetroleum chemicals including furfural and 5-hydroxymethylfurfural (HMF)^3^,^4^,^5^,^6^. These compounds are considered to be versatile platform chemicals with high potential utility in organic synthesis and industrial applications to give rise to a diverse range of furans and their derivatives. In fact, a number of catalytic transformations of furans or their congeners to valuable chemicals have long been developed in both academia and industry^7^,^8^,^9^. Among the precedents, however, the ring-opening reactions of furans are relatively less highlighted^10^,^11^,^12^ when compared with the derivatization of furans maintaining the ring skeleton^13^,^14^,^15^. One notable example of the ring-opening of furans is the production of levulinic acid via an acid-catalysed hydration of HMF (Fig. 1a)^16^,^17^. Recently, Gordon and coworkers showed that a sequential catalysis involving ring-opening hydrolysis of chain-extended HMF derivatives followed by hydrodeoxygenation using a combination of acid and metal catalyst can produce linear alkanes^18^. In addition, while a borane catalyst system was proved to be applicable in the transformations of carbohydrates, it was also employed for the ring-opening of furan derivatives. For instance, Gagné *et al*. found that B(C_6_F_5_)_3_ catalyses the deoxygenation of carbohydrate-based polyols with hydrosilanes to give chiral-alcohol synthons^19^ or hydrocarbons^20^,^21^ with remarkable chemoselectivity. Njardarson *et al*. employed B(C_6_F_5_)_3_ catalyst for the reduction of 2,5-dihydrofurans using hydrosilanes to obtain silyl(homo)allyl ethers, which was proposed to form via a hydrosilylative C−O bond cleavage^22^. On the other hand, Ashley *et al*. reported that hydrogenation of furan derivatives under the frustrated Lewis pairs catalysis using (B(C_6_Cl_5_)(C_6_F_5_)_2_) delivers reduced tetrahydrofuran compounds^23^. Despite of such progresses on furan transformation reactions, there have been no developments thus far for the selective conversion of furans to ring-opened products bearing a sp^3^ C−Si bond at ambient conditions.

Described herein is the development of a boron-catalysed cascade silylative transformation of furans (**I**) involving selective ring-opening and closing processes, thus allowing for the sequential formation of two types of new silylated products of α-silyloxy-(*Z*)-alkenyl silanes (**II**) and *trans*-(2-alkyl)cyclopropyl silanes (**IV**) (Fig. 1b). In this transformation, several aspects are especially notable: First, while the B(C_6_F_5_)_3_-catalysed hydrosilylation of olefins is known^24^, the sp^3^ C−O and sp^2^ C=C bonds in the initially ring-opened α-silyloxy-(*Z*)-alkenyl silanes (**II**) remain unreacted. Second, the subsequent ring-closing of **II** is readily enabled simply by additional equivalent of hydrosilane leading to silylcyclopropanes (**IV**) without the need to isolate the ring-opened compounds **II**. Third, the observed high regio and stereoselectivities in both the initial ring-opening and subsequent cyclopropanation processes are an outcome of kinetic differentiation within the borane catalytic cycle. In addition, both types of products obtained from the present furan conversion are synthetically highly valuable compounds possessing sp^3^ C−Si bonds^25^,^26^,^27^. Also, a broad range of furan congeners derived from 5-hydroxymethylfurfural are successfully applied for the selective cascade hydrosilylation to yield silicon-functionalized products (Fig. 1c).

## Results

### Discovery of new cascade transformations of furans

**Figure 2:**
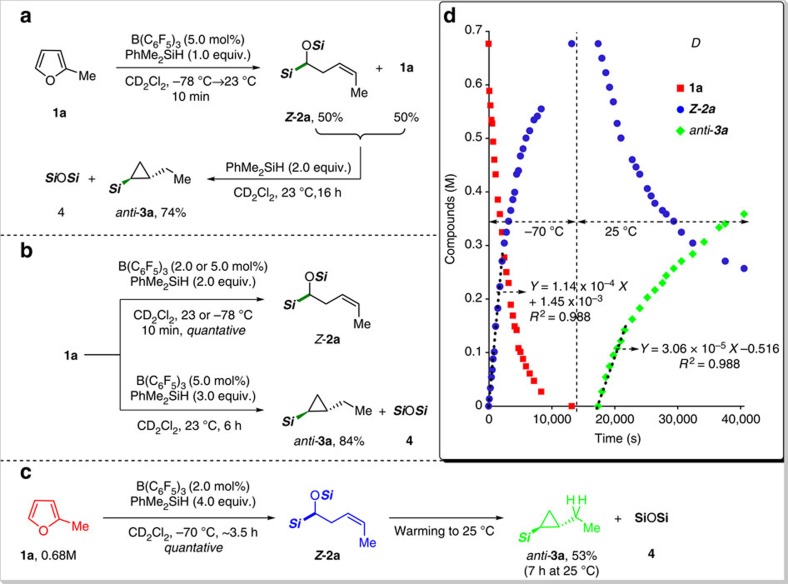
Preliminary catalytic reactions and NMR monitoring. (**a**) Sequential addition of hydrosilane. (**b**) Control of product distribution by the equivalents of hydrosilane. (**c**) Ring-opening and closing transformations of 2-methylfuran in one flask. (**d**) NMR monitoring of the B(C_6_F_5_)_3_-catalyzed cascade conversion of 2-methylfuran with hydrosilane. (***Si***=SiMe_2_Ph).

When 2-methylfuran was treated with 1.0 equiv. of PhMe_2_SiH in the presence of B(C_6_F_5_)_3_ (5.0 mol%) in dichloromethane solvent, a new product, α-silyloxy-3-pentenyl silane *Z*-**2a**, was observed to form in 10 min at −78 °C along with unreacted **1a** in a 1:1 ratio (Fig. 2a; see Supplementary Fig. 1). Subsequent addition of 2.0 equivalents of PhMe_2_SiH into the above reaction mixture brought about exclusive formation of silylated cyclopropane as a single stereoisomer (*anti***-3a**) in 74% yield over 16 h in addition to (PhMe_2_Si)_2_O byproduct **4** (Fig. 2a; see Supplementary Fig. 2). These results indicate that the kinetic barrier for the B(C_6_F_5_)_3_-catalysed ring-opening hydrosilylation of **1a** to *Z*-**2a** is much lower than that of the ring-closing process from *Z*-**2a** to *anti***-3a**. Consistent with this interpretation, we were able to confirm that **1a** was quantitatively converted to *Z*-**2a** with 2.0 equivalents of PhMe_2_SiH, while the use of 3.0 equiv. of the silane led to the exclusive formation of *anti***-3a** together with stoichiometric byproduct **4** (Fig. 2b).

To gain more insight into the kinetics of each step of the cascade hydrosilylation processes, we monitored the reaction progress by ^1^H NMR spectroscopy (Fig. 2c,d; see Supplementary Fig. 3). A precooled mixture of B(C_6_F_5_)_3_, **1a**, and PhMe_2_SiH (1:50:200) in CD_2_Cl_2_ at −70 °C was found to give *Z*-**2a** in a quantitative NMR yield over 3.5 h. On further warming up to 25 °C, *Z*-**2a** started to subsequently undergo a ring-closing process to afford silylated cyclopropane *anti***-3a** (53% NMR yield in 7 h). These results clearly indicate that the furan transformation proceeds via sequential reductive pathways, which are strongly governed by kinetic factors. Moreover, the initial rates for each process in the hydrosilylation cascade of 2-methylfuran were determined to be 1.14 × 10^−4^ M s^−1^ (for the ring-opening at −70 °C) and 3.06 × 10^−5^ M s^−1^ (for the cyclopropanation at 25 °C) (Fig. 2d; Supplementary Fig. 3).

### Substrate scope of the B(C_6_F_5_)_3_-catalysed cascade silylative reduction of furans

With the preliminary results on the 2-methylfuran conversion in hand, we carried out additional optimization studies (Supplementary Tables 1–3) and investigated the substrate scope of this catalysis. 2-Alkyl or 2-arylfuran derivatives employed in this study were easily prepared via the Pd-catalysed Suzuki–Miyaura cross-coupling reaction of (2-furanyl)boronic acid with the corresponding alkyl or aryl halides (Supplementary Methods, GP1). In the present cascade transformation of furans, several notable features were revealed: (i) only equimolar amounts of hydrosilanes are needed to enable the ring-opening process, thus indicating that the conversion is atom-economical; (ii) the reaction is highly stereoselective in that one isomeric products are formed; (iii) the subsequent cyclopropanation process can be carried out without isolation of ring-opened intermediates, α-silyloxy-(*Z*)-homoallylsilanes; (iv) again, the ring-closing process is highly stereoselective affording *anti*-products exclusively; and (v) the overall procedure is convenient and easy to scale up with high catalyst turnovers (∼2,000).

We were pleased to observe that the optimized conditions were readily applicable to a broad range of furan derivatives to give α-silyloxy-(*Z*)-homoallylsilanes (Table 1; Conditions A). Substrates with aliphatic substituents on the furan C-2 were all compatible with the B(C_6_F_5_)_3_-catalysed (2.0 mol%) ring-opening process at ambient temperature. Analytically pure α-silyloxy-(*Z*)-homoallylsilane products (*Z*-**2a**∼**2d**) were obtained in high yields and with excellent stereoselectivities (*Z/E*: >99/1, as measured by crude ^1^H NMR experiments). The reaction of aryl-substituted furans proceeded with a similar level of selectivity and efficiency irrespective of the electronic properties of the aryl moiety (*Z*-**2e**∼**2k**, *Z/E*: >99/1, 83–92%). Gratifyingly, potentially reactive functional groups (*Z*-**2l**∼**2n**) were tolerated under the reaction conditions, while reactions of the furan substrates bearing sterically bulky substituents were also smooth (*Z*-**2o**∼**2q**). In addition, substrates having multiple furan rings (**1r**∼**1t**) were readily employed for the current process to afford the corresponding multi-functionalized C-2 or C-3 symmetrical products (**2r**∼**2t**). Interestingly, the newly generated multiple double bonds are all in (*Z*)-form, strongly suggesting that the ring-opening process is highly stereoselective.

We next turned our attention to the formation of silylated cyclopropanes based on the above mechanistic insights (Fig. 2). A brief optimization study led us to establish the one-pot reaction conditions that do not need to isolate α-silyloxy-(*Z*)-homoallylsilanes compounds. The key in this triple cascade hydrosilylation process was the amounts of hydrosilanes employed: while dimethylphenylsilane was most effective, the use of slightly excessive this silane (4.0 equivalents) was found to be optimal for high product yields. Under these conditions, a range of furan substrates were smoothly converted to the desired 2-alkylcyclopropyl silanes with excellent *anti*-diastereoselectivity in the presence of B(C_6_F_5_)_3_ catalyst (Table 1; Conditions B). *Anti*-diastereoselectivity was confirmed by NMR analyses (Supplementary Figs 114–122). Furans substituted with alkyl groups at the 2-position smoothly underwent the triple hydrosilylation cascade with PhMe_2_SiH (4.0 equiv.) to afford the corresponding products (*anti*-**3a**, **3b**∼**3f**, diastereomeric ratio; >99/1; the diastereoselectivity was measured by ^1^H NMR of the crude reaction mixture). Likewise, 2-arylfurans with varying electronic properties were converted to *anti*-(2-arylmethyl)cyclopropyl silane in high yields (*anti-***3g**∼**3m**).

Furans bearing multi-substituted phenyl and polyaromatic moieties were also competent substrates for this hydrosilylation cascade (*anti-***3n**∼**3p**). Interestingly, multiple furans connected through a benzene core were smoothly converted to the corresponding products bearing bis- or tris-cyclopropyl groups (**3q**∼**3r**, respectively), still displaying an *anti*-stereochemical relationship in each newly generated cyclopropane. It should be mentioned that Stephan, Hashmi, and coworkers recently showed that a stoichiometric reaction of B(C_6_F_5_)_3_ with 1,6-enynes proceeds via initial cyclopropanation and then formal 1,1-carboboration^28^. In addition, Erker *et al*. reported that HB(C_6_F_5_)_2_ can mediate a conversion of allyldimesitylphosphanes to phosphinomethyl-substituted cyclopropane derivatives under frustrated Lewis pair conditions in a stoichiometric manner^29^.

### Structural diversity with other furans and benzofurans

In addition to 2-substituted furans, furan and other regioisomeric derivatives were observed to undergo the selective ring-opening reactions at ambient temperature to give structurally diverse products bearing sp^3^ C−Si bonds (Table 2). Unsubstituted furan was reacted with PhMe_2_SiH under the borane catalysis, leading to (*Z*)-α-hydroxy allylsilane upon treatment of the reaction mixture with methanol solution of K_2_CO_3_ (*Z*-**5**, Table 2). 3-Substituted furan was also reactive toward the silylative reduction, furnishing an allylsilane having both silyloxy and aryl moieties at the C1 and C2-positions (*Z*-**6**). It was interesting to see that disubstituted furan underwent the ring-opening process via slightly modified pathway (see Supplementary Discussion 2). For instance, 2,3-dimethylfuran was reacted under the same conditions to eventually provide γ-silylketone product (**7**) after treating the catalytic reaction mixture with tetrabutylammonium fluoride (*n*Bu_4_NF) in one pot (**7**, 73% over two steps). In a similar manner, 2,5- dimethylfuran was doubly hydrosilylated to afford the corresponding γ-silylketone (**8**) upon treatment of *n*Bu_4_NF (**8**, 75% over two steps). The present procedure was also applicable to the silylative reduction of benzofurans. Reactions of benzofuran or 2-substituted benzofurans with hydrosilane in the presence of borane catalyst selectively proceeded to provide a range of 2-alkyl-substituted phenols (**9**∼**12**) having a sp^3^ C−Si bond in high yields again after treating the methanol solution of K_2_CO_3_.

### Mechanistic experiments and proposed catalytic pathway

**Figure 3:**
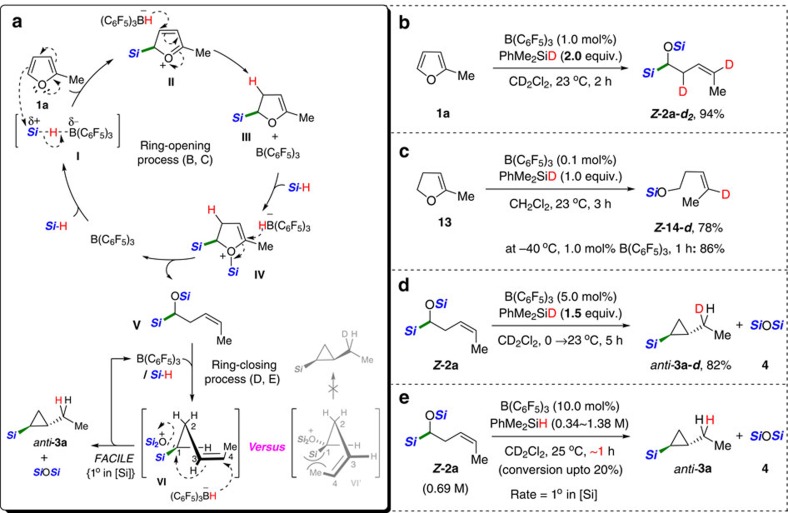
Mechanistic experiments and proposed catalytic pathway. (**a**) Proposed pathway of the B(C_6_F_5_)_3_-catalysed silylative ring-opening of furans and subsequent cyclopropanation. For simplicity, hydrosilane is shown as ***Si***-H/D (***Si***=SiMe_2_Ph). (**b**) Deuterium labelling experiment. (**c**) A model reaction with 2,3-dihydro-5-methylfuran. (**d**) A test ring-closing reaction of *Z***-2a** with 1.5 equiv. of deuterated hydrosilane. (**e**) Rate-order assessment of silane based on initial rates in a range of initial silane concentrations.

Based on the present observations and precedents^30^,^31^ regarding the mechanism of B(C_6_F_5_)_3_-mediated hydrosilylation, we propose a cascade catalytic pathway leading to the *Z*-selective homoallylic silane, and subsequently the *anti*-cyclopropyl silanes, as seen in Fig. 3a using 2-methylfuran as a model substrate. On the *in situ* generation of a borane-silane adduct **I**, 2-methylfuran **1a** attacks the silylium species to afford an oxonium species **II** that immediately reacts with the borohydride leading to a partially reduced furan intermediate **III** bearing a sp^3^ C−Si bond next to an oxygen atom. A subsequent hydrosilylation of **III** is suggested to take place through a key intermediate **IV**, at which a selective borohydride attack at the α-carbon via a nucleophilic vinylic substitution (S_N_V) pathway leads to α-silyloxy-(*Z*)-alkenyl silane (**V**)^32^ (Fig. 3a (top), **1a** to **V**).

An *O*-silyl oxonium species **VI** formed by a reaction of **V** with active species **I** is proposed to be involved in the ring-closing process, which will induce the borohydride nucleophilic attack (S_N_2′-type mechanism) to give the corresponding silylated cyclopropanes with the release of one equivalent of siloxane (Fig. 3a (bottom), **V** to *anti*-**3a**). With regard to the oxonium species **VI**, two different types of intermediates would be plausible: (*i*) **VI** that has C1 and C4 substituents (oxonium/silyl and methyl groups, respectively) at the opposite space and (*ii*) **VI**′ having two groups on the same side. The relative stereochemistry (*anti*-) of two substituents in the resultant cyclopropane product can be reasoned by proposing a nucleophilic substitution of an *in situ* generated oxonium species **VI** (ref. 20) by the borohydride nucleophile to minimize the steric repulsion between substituents^33^.

This mechanistic proposal was supported by a series of experiments, including kinetic and isotopic studies. A hydrosilylation of 2-methylfuran (**1a**) with PhMe_2_SiD (2.0 equiv.) under standard conditions led to the exclusive incorporation of deuterium at the two positions to give *Z***-2a-*****d***_**2**_ (Fig. 3b). A reaction of 2,3-dihydro-5-methylfuran, **13** with 1.0 equiv. of PhMe_2_SiD afforded a product *Z***-14-*****d*** with a selective deuterium incorporation at the olefinic carbon (Fig. 3c). These results led us to propose a selective and consecutive attack of a borohydride nucleophile following the silylium ion (R_3_Si^+^) (refs 34, 35, 36, 37, 38) transfer and the involvement of a partially reduced dihydrofuran intermediate such as **13** during the ring-opening process. Although the proposed vinylic substitution by the borohydride nucleophile is unknown to our best knowledge, an example of an inversion at the alkenyl configuration was previously reported in a reaction of alkylvinyliodonium electrophiles with halide nucleophiles^39^.

As expected, the subsequent ring-closing process from **V** to the corresponding silylated cyclopropanes was highly stereoselective as proved by an isotope experiment. Indeed, when α-silyloxy-(*Z*)-alkenyl silane (*Z*-**2a**) was allowed to react with PhMe_2_SiD (1.5equivalents), cyclized product (*anti*-**3a-*****d***) was obtained in 82% yield as a single isomer with the exclusive incorporation of deuterium at the α-ethyl position (Fig. 3d). A stoichiometric amount of siloxane was also confirmed to be generated during the cyclopropanation process. An initial-rate study for the cyclopropanation of **V** under the B(C_6_F_5_)_3_-catalysed hydrosilylation conditions clearly showed that the reaction was first-order in silane concentration (Fig. 3e; see Supplementary Figs 4 and 5), leading us to propose that the cyclization path involves an O-silylated oxonium ion possessing a borohydride anion^40^.

### Synthetic applications

**Figure 4:**
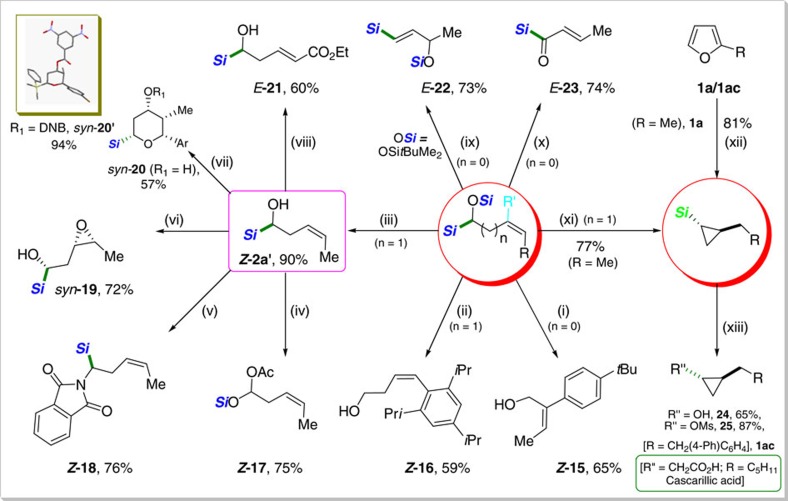
Enrichment and elaboration of products. (i) CsF (5 equiv.), 23 °C, 8 h, DMF, (R′=(*t*Bu)-C_6_H_4_, R=Me). (ii) TBAF (3 equiv., 1 M in THF), 23 °C, 15 h, (R′=H, R=(2,4,6-triisopropyl)C_6_H_2_). (iii) K_2_CO_3_ (2 equiv.), MeOH, 0→23 °C, 4 h, (R′=H, R=Me). (iv) Pb(OAc)_4_ (1.1 equiv.), C_6_H_6_, 0→23 °C, 2 h. (v) PPh_3_ (1.3 equiv.), Diethyl azodicarboxylate (1.3 equiv.), Phthalimide (1.3 equiv.), THF, 0→23 °C, 20 h. (vi) VO(acac)_2_ (5 mol%), TBHP (2.2 equiv.), CH_2_Cl_2_, −10→0 °C, 5 h. (vii) *p*-Br-C_6_H_4_CHO (1.2 equiv.), TFA (2.5 equiv.), CH_2_Cl_2_ (3 ml), −20 °C, 3 h; DNB=3,5-dinitrobenzoyl. (viii) Ethyl acrylate (10 equiv.), Grubbs-II (5 mol%), 50 °C, 15 h. (ix) BF_3_**·**Et_2_O (1.1 equiv.), CH_2_Cl_2_ (0.14 M), −78 °C, 5 h, (R′=H, R=Me). (x) (COCl)_2_ (2.0 equiv.), DMSO (4.0 equiv.), Et_3_N (5.0 equiv.), −78 °C, 4 h, (R′=H, R=Me). (xi) B(C_6_F_5_)_3_ (3.0 mol%), PhMe_2_SiH (1.5 equiv.), CH_2_Cl_2_, 0→23 °C, 12 h, (R′=H, R=Me). (xii) 2-Methylfuran (**1a**, 1.0 equiv.), B(C_6_F_5_)_3_ (5.0 mol%), Ph_2_SiH_2_ (3.0 equiv.), CH_2_Cl_2_, 0→23 °C, 8 h. (xiii) (a) **1ac** (1.0 equiv.), B(C_6_F_5_)_3_ (5.0 mol%), Ph_2_SiH_2_ (3.0 equiv.), CH_2_Cl_2_, 0→23 °C, 10 h; (b) H_2_O_2_ (20 equiv.), KF (10 equiv.), KHCO_3_ (10 equiv.), THF/MeOH (1/1), 23 °C, 16 h. (***Si***=SiMe_2_Ph, ***Si***=SiPh_2_H/SiMe_2_Ph).

The synthetic utility of the products obtained was demonstrated to be very broad, including a wide range of simple and convenient organic transformations (Fig. 4). α-Silyloxy-(*Z*)-alkenyl silanes were easily desilylated to give allylic- or homoallylic alcohols with retention of double bond stereochemistry (*Z***-15** and *Z*-**16**, respectively). α-Hydroxy homoallylic silane (*Z***-2a**′), accessed through the *O*-desilylation of α-silyloxy-(*Z*)-homoallylic silane, turned out to be also synthetically versatile. α-Silyloxy acetate (*Z*-**17**) was readily produced by a radical Brook rearrangement in good yield^41^. Moreover, *Z***-2a**′ was smoothly converted to (*Z*)-α-amino silane derivative (*Z*-**18**) under the Mitsunobu conditions leading to C−N bond formation. Epoxide (**19**) was obtained *syn*-selectively via the Sharpless directed epoxidation of *Z*-**2a**′ (ref. 42), thus proving that α-silylalcohol works as an effective directing group. An efficient Prins cyclization^43^ of *Z*-**2a**′ was achieved to furnish a tetrasubstituted pyran ring (*syn*-**20**) with complete stereocontrol. The observed *syn*-stereochemistry was unambiguously confirmed by NMR and X-ray diffraction analyses. Cross metathesis of *Z*-**2a**′ with ethyl acrylate was carried out stereoselectively with Grubbs II catalyst to give *E*-**21**. Lewis acid-promoted allylic rearrangement^44^ of α-silyloxy-(*Z*)-allylic silane bearing *tert*-butyldimethylsilyloxy group *Z***-5**′′ (*tert*-butyldimethylsilyl derivative of *Z***-5**, see Supplementary Methods) proceeded smoothly to afford silyloxy vinylic silane (*E*-**22**), another versatile synthetic building block. Acylsilane (*E*-**23**), a valuable organosilicon reagents in organic synthesis^45^, was readily obtained.

Two preparative procedures of cyclopropyl silanes were mild and efficient on a large scale (Supplementary Methods): (i) a stepwise route via α-silyloxy homoallylic silane (*Z*-**2a**, procedure “xi” in 77%), and (ii) a direct conversion of furan (procedure “xii” in 81% yield). The subsequent oxidation of the C−Si bond under the Tamao–Fleming conditions^46^ proceeded with retention of stereochemistry to furnish the versatile synthons, cyclopropanol (**24**) and its methansulfonyl derivative (**25**), which are broadly utilized in synthetic and medicinal chemistry^47^. For instance, cascarillic acid^48^, a natural product derived from the bark of the medicinal shrub *Croton eluteria*, can be envisioned to be accessible based on our current procedure.

In summary, we have developed the tris(pentafluorophenyl)borane-catalysed cascade transformation of furans that are readily available from renewable biomass resources to obtain synthetically valuable silicon-functionalized products such as α-silyloxy-(*Z*)-alkenyl silanes and *anti*-cyclopropyl silanes. Simply by varying the stoichiometry of employed hydrosilanes in presence of B(C_6_F_5_)_3_ catalyst, the product distribution could completely be controlled with high efficiency (TON up to 2,000) and excellent stereoselectivity. The transformation does not require transition metal catalysts, proceeds efficiently on large scale, and is broadly applicable to various types of furans and their derivatives, bringing about a significant structural diversity in the product obtained. A proposed mechanistic pathway involves a series of hydrosilylation cascades, containing a ring-opening and subsequent S_N_2′-type ring-closing process, both mediated by a B(C_6_F_5_)_3_ catalyst. The synthetic utility of obtained silicon-functionalized products was demonstrated in a range of post-transformations. It is anticipated that this study will stimulate future developments in the transformations of biomass-derived platform chemicals to synthetic valuables.

## Methods

### General procedure for the silylative ring opening reaction (conditions A, GP1)

In a flame-dried flask bearing a stirring bar, B(C_6_F_5_)_3_ (0.01∼0.02 mmol, 2.0 mol%) was dissolved in CH_2_Cl_2_ (0.4∼0.8 ml). Silane (1.025∼2.050 mmol) was added, and the solution was shaken shortly to make it homogeneous. The corresponding furan derivative (**1a**∼**1t**, 0.50∼1.0 mmol) was then added and the reaction mixture was stirred at 23 °C for the indicated time (1∼15 h). After quenching the reaction mixture with Et_3_N (10.0∼20.0 mol%), the crude reaction mixture was concentrated under reduced pressure and then purified by flash column chromatography on silica gel (using either hexane only or a mixture of hexane/ethyl acetate) to afford the desired products (*Z*-**2a**∼*Z*-**2t**, in all cases *Z*/*E* >99/1).

### General procedure for the cyclopropanation reaction (conditions B, GP2)

In a flame-dried flask bearing a stirring bar, B(C_6_F_5_)_3_ (0.025 mmol, 5.0 mol%) was dissolved in CH_2_Cl_2_ (0.2 ml). Silane (1.5∼2.0 mmol) was added, and the solution was shaken shortly to make it homogeneous. The corresponding furan derivative (**1a**∼**1t**, 0.50 mmol) was then added at 0 °C and the reaction mixture was stirred at 23 °C for the indicated time (4∼22 h). After quenching the reaction mixture with Et_3_N (10.0∼20.0 mol%), the crude reaction mixture was concentrated under reduced pressure and then purified by flash column chromatography on silica gel (using either hexane or a mixture of hexane and ethyl acetate) to afford the desired products (*anti*, **3a**∼**3r**, in all cases dr >99/1).

### Data availability

The authors declare that the data supporting of the findings of this study are available within the article and Supplementary Information files. For the experimental procedures and spectroscopic and physical data of compounds, see Supplementary Methods. For NMR analysis of the compounds in this article, see Supplementary Figs 6–187. The CCDC 1505482 (*Z*-**2a″**) and CCDC 1505484 (*syn*-**20′**) contain the supplementary crystallographic data for this paper (Supplementary Tables 4 and 5). These data can be obtained free of charge from The Cambridge Crystallographic Data Centre via http://www.ccdc.cam.ac.uk/data_request/cif. All other data are available from the authors on reasonable request.

## Additional information

**How to cite this article:** Hazra, C. K. *et al*. Borane catalysed ring opening and closing cascades of furans leading to silicon functionalized synthetic intermediates. *Nat. Commun.* **7,** 13431 doi: 10.1038/ncomms13431 (2016).

**Publisher's note**: Springer Nature remains neutral with regard to jurisdictional claims in published maps and institutional affiliations.

## Supplementary Material

Supplementary Information Supplementary Figures 1-187, Supplementary Tables 1-5, Supplementary Discussions, Supplementary Methods and Supplementary References.

Peer Review File

## Figures and Tables

**Figure 1:**
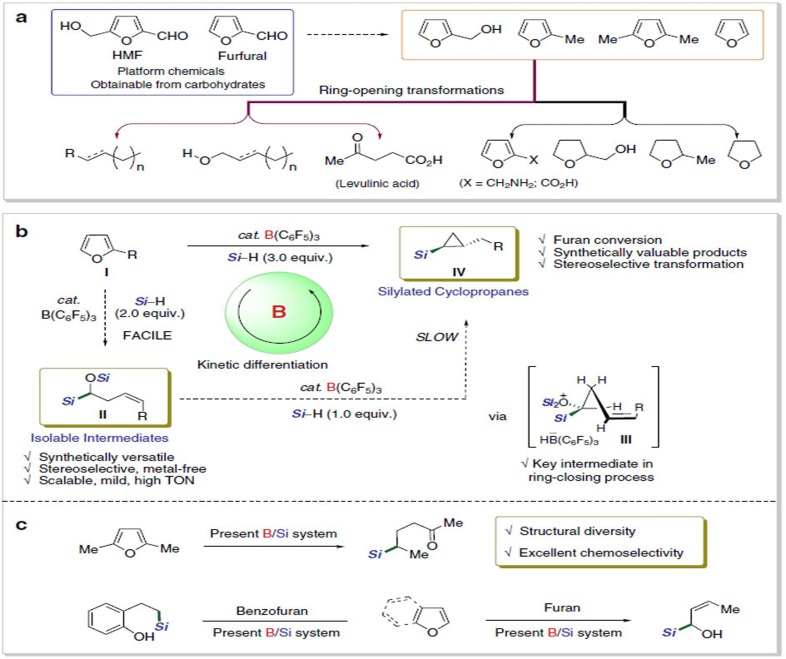
Furans from biomass resources and their transformations. (**a**) Furan platform chemicals and known post-transformations leading to diverse synthetic valuables. (**b**,**c**) Present work; Borane-catalysed ring-opening and closing cascades of furans giving rise to synthetically valuable silicon compounds in reaction with hydrosilanes (***Si***=SiR′_2_R″).

**Table 1:**
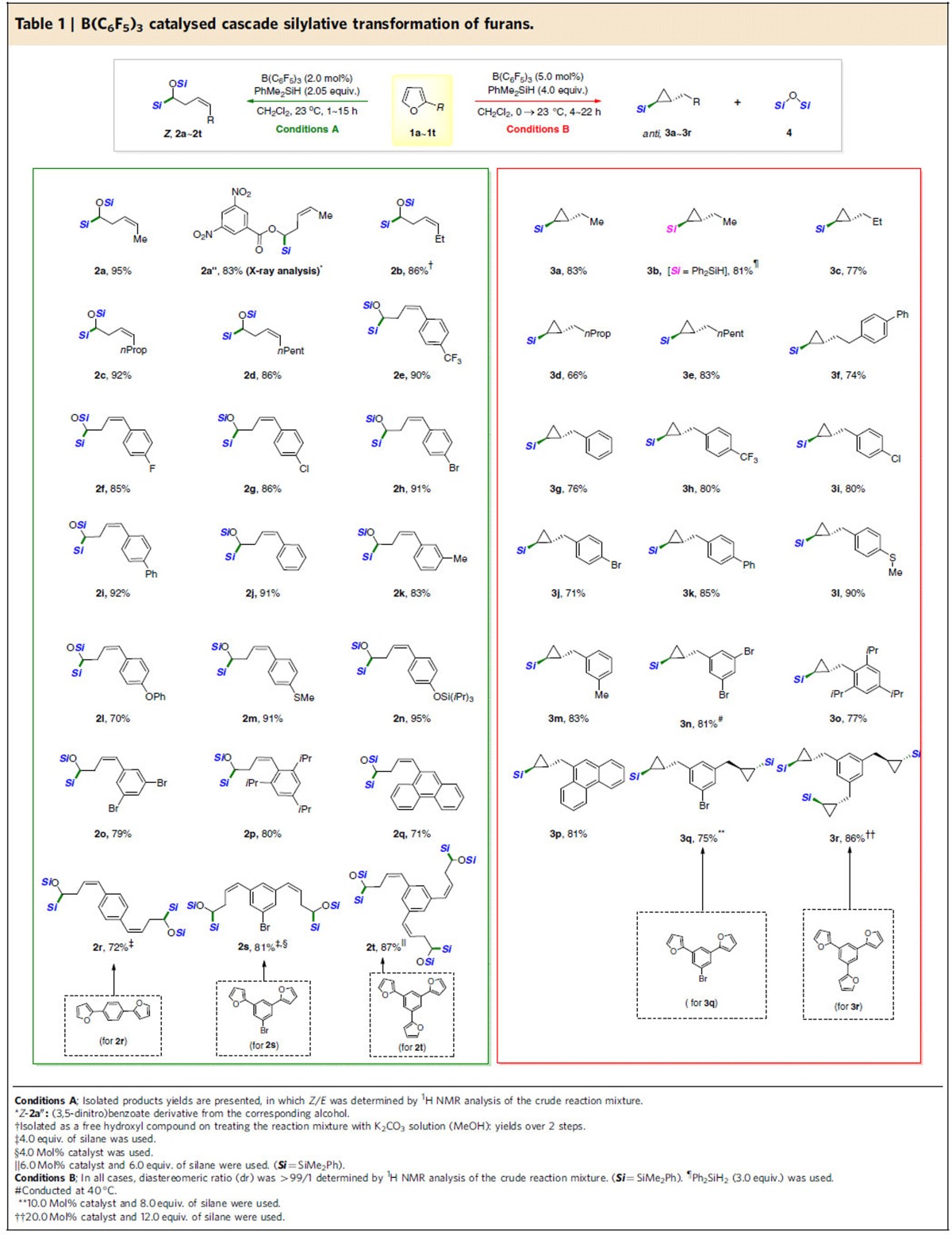
B(C_6_F_5_)_3_ catalysed cascade silylative transformation of furans.

**Table 2:**
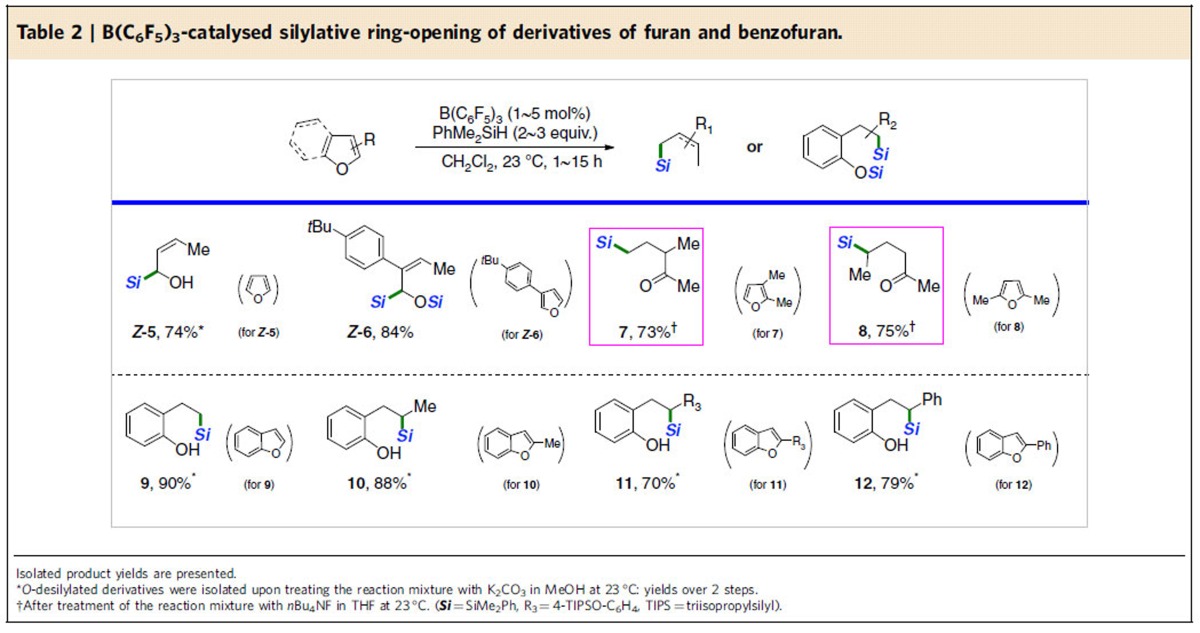
B(C_6_F_5_)_3_-catalysed silylative ring-opening of derivatives of furan and benzofuran.
