# Supplementary material for: Borane catalysed ring opening and closing cascades of furans leading to silicon functionalized synthetic intermediates
Source: Nat Commun. 2016 Nov 28;7:13431. doi: 10.1038/ncomms13431 (PMC5133710; doi:10.1038/ncomms13431)
Supplement: Supplementary Information — Supplementary Figures 1-187, Supplementary Tables 1-5, Supplementary Discussions, Supplementary Methods and Supplementary References. [file ncomms13431-s1.pdf]

Supplementary Figure 1.
Reaction of 2-methylfuran with $PhMe_2SiH$ (1.0 equiv., Fig. 2A, crude reaction mixture)

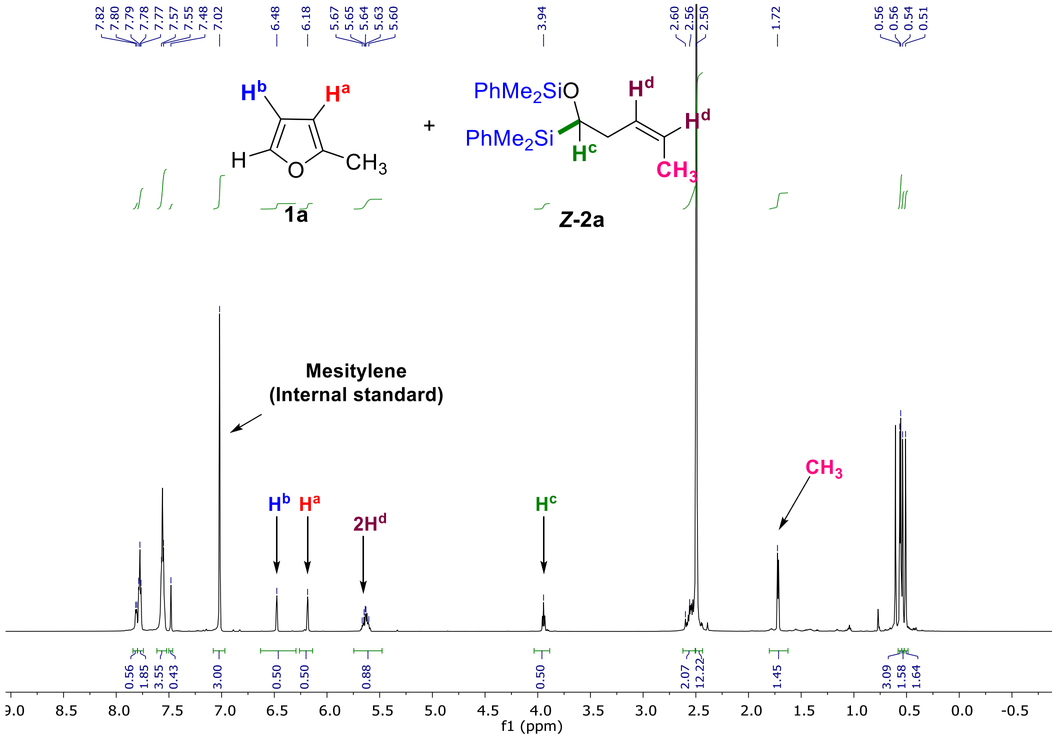

Supplementary Figure 2. Reaction of (*Z*-2a + 1a) with additional $PhMe_2SiH$ (2.0 equiv., Fig. 2A, crude reaction mixture)

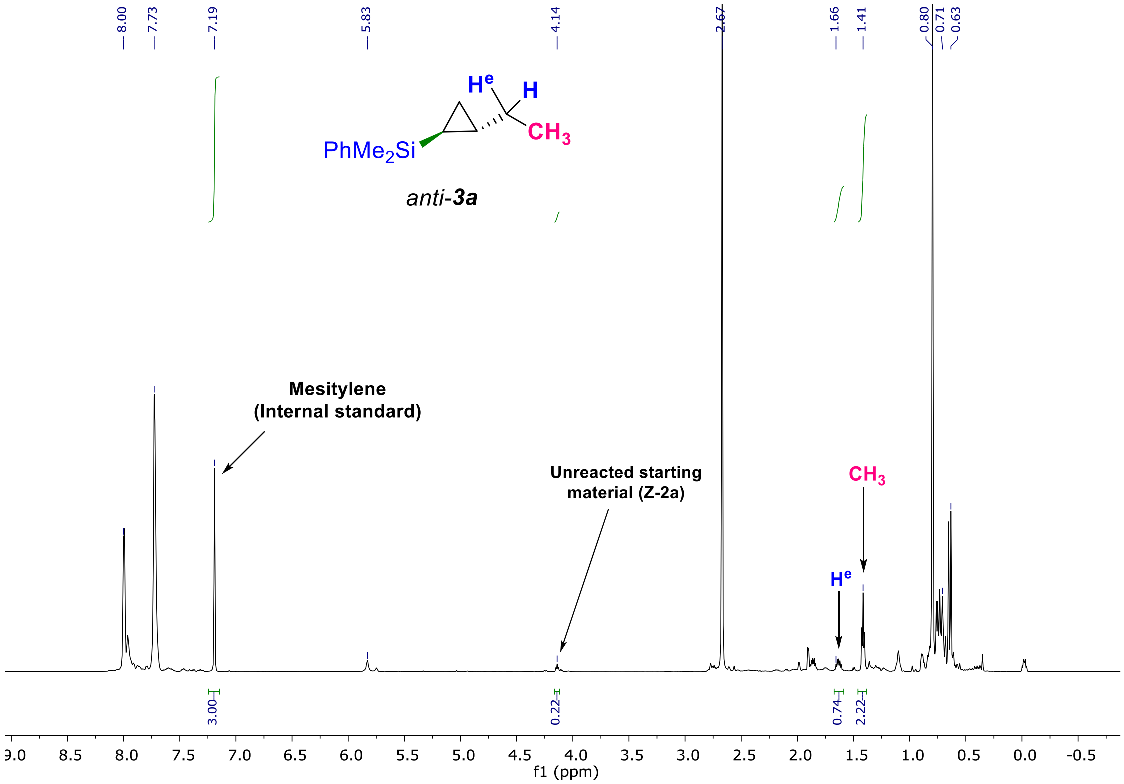

Supplementary Figure 3. ^{1}H NMR Monitoring of the Reaction of 2-methylfuran with $PhMe_2SiH$ (4.0 equiv., Fig. 2C)

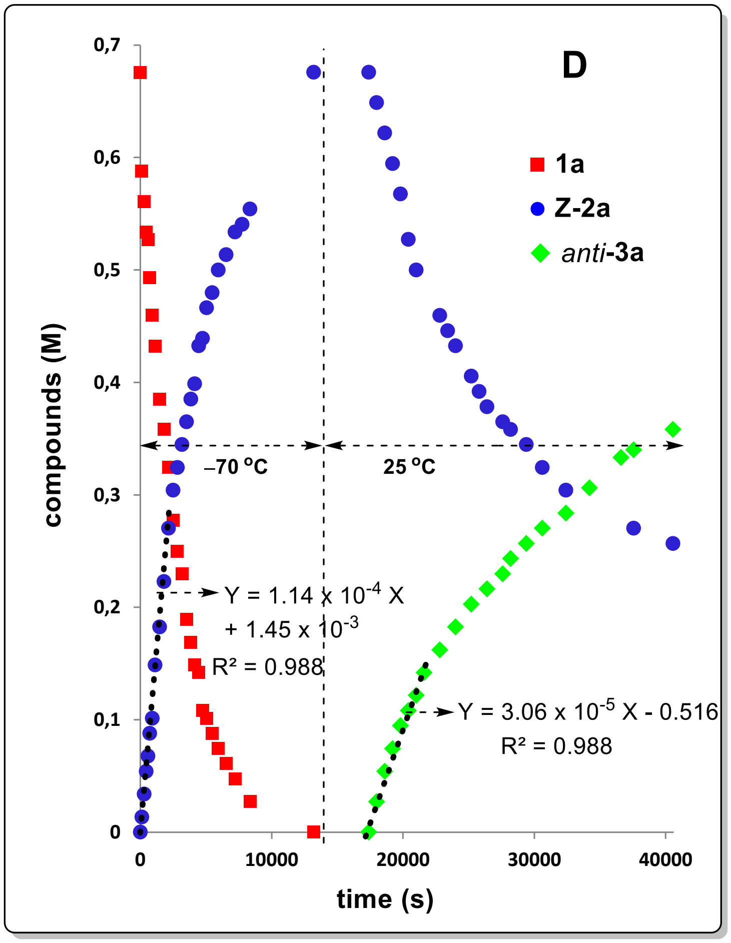

Concentration versus time curve for the silylative reduction of 2-methylfuran 1a (1.0 equiv.) with $PhMe_2SiH$ (4.0 equiv.) catalyzed by $B(C_6F_5)_3$ at -70 °C and 25 °C in CD_2Cl_2.

Supplementary Figure 4. Initial-Rate Kinetic Measurements (Fig. 3E)

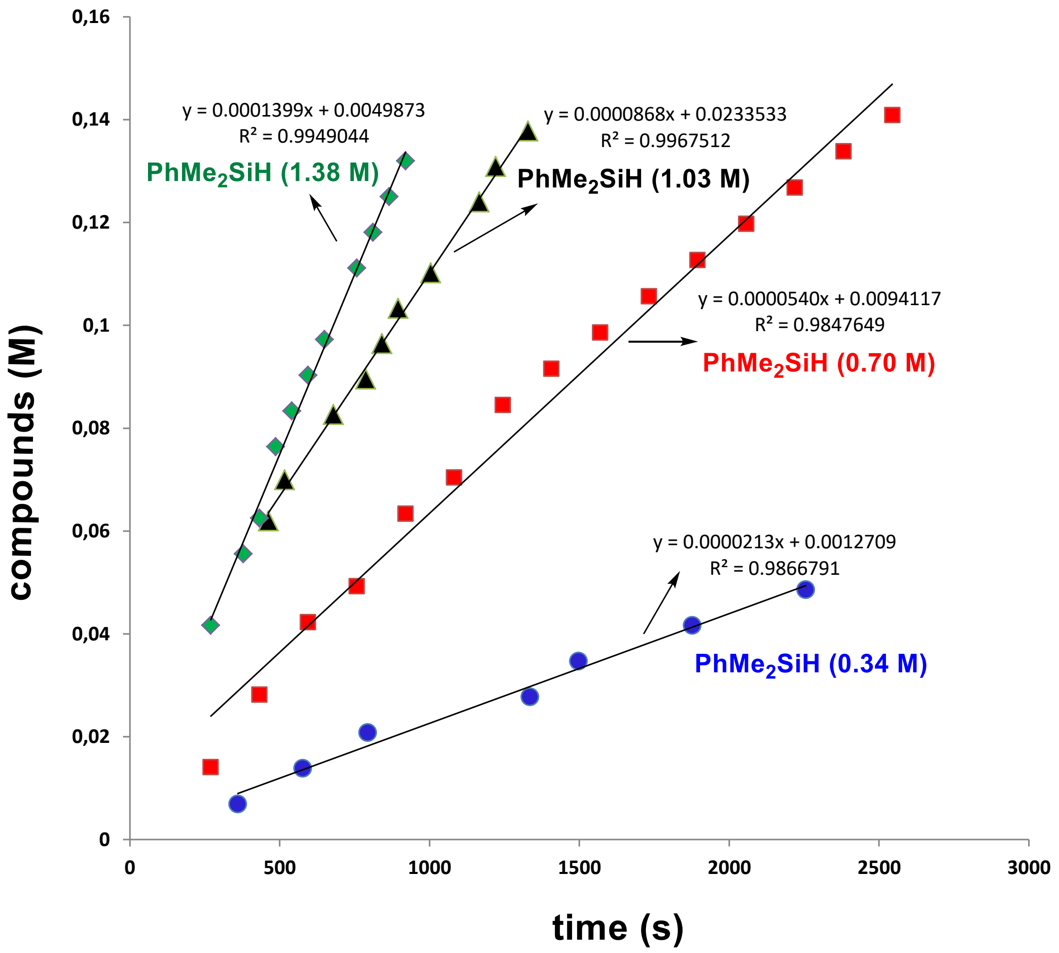

Reaction Profile for Initial Rate Calculation in the Cyclopropanation of *Z*-2a at 25 °C

Supplementary Figure 5. Initial rates in a range of $[Si]_0$ (Fig. 3E)

$[PhMe_2SiH]_0$ (M)	Rate (M/s)	R^2
0.34	0.00002139	0.9866791
0.70	0.0000540	0.9847649
1.03	0.0000868	0.9967512
1.38	0.0001399	0.9949044

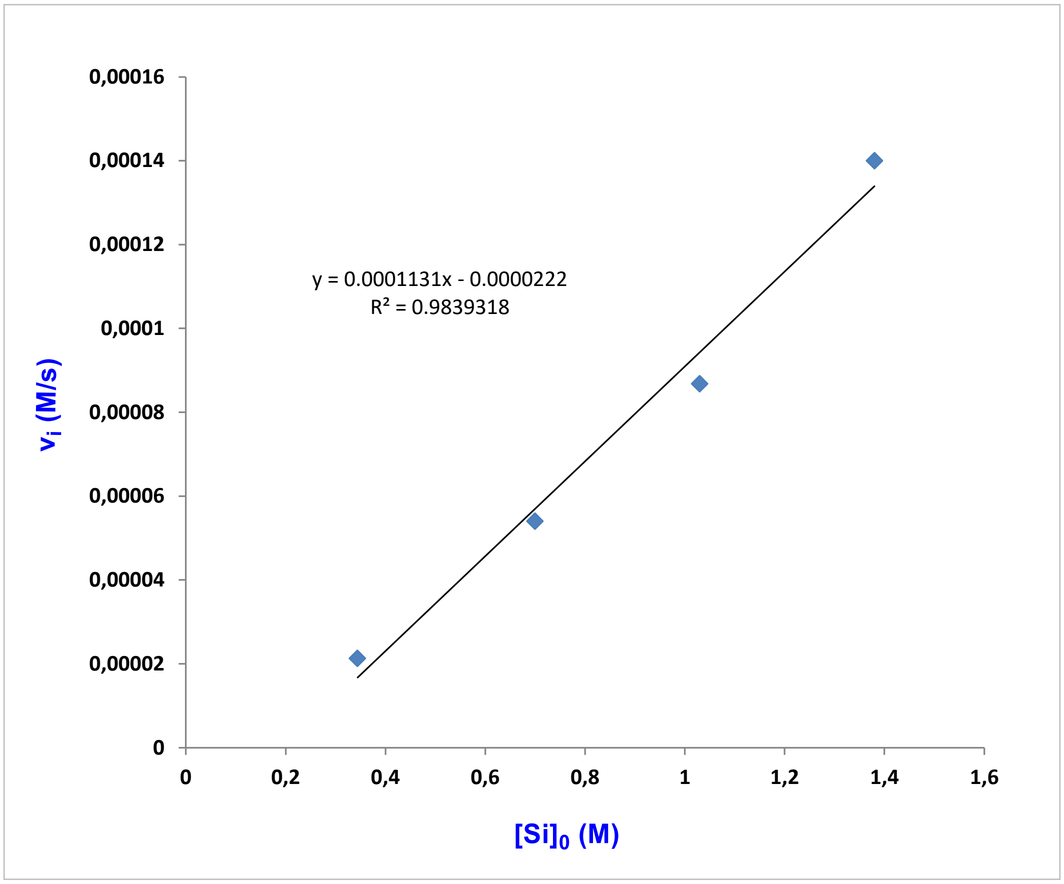

Plot of the initial rate v_i of appearance of *anti*-3a Vs initial $PhMe_2SiH$ concentration at 25 °C.

Supplementary Figure 6. ^{1}H and ^{13}C-NMR spectra of **1e**

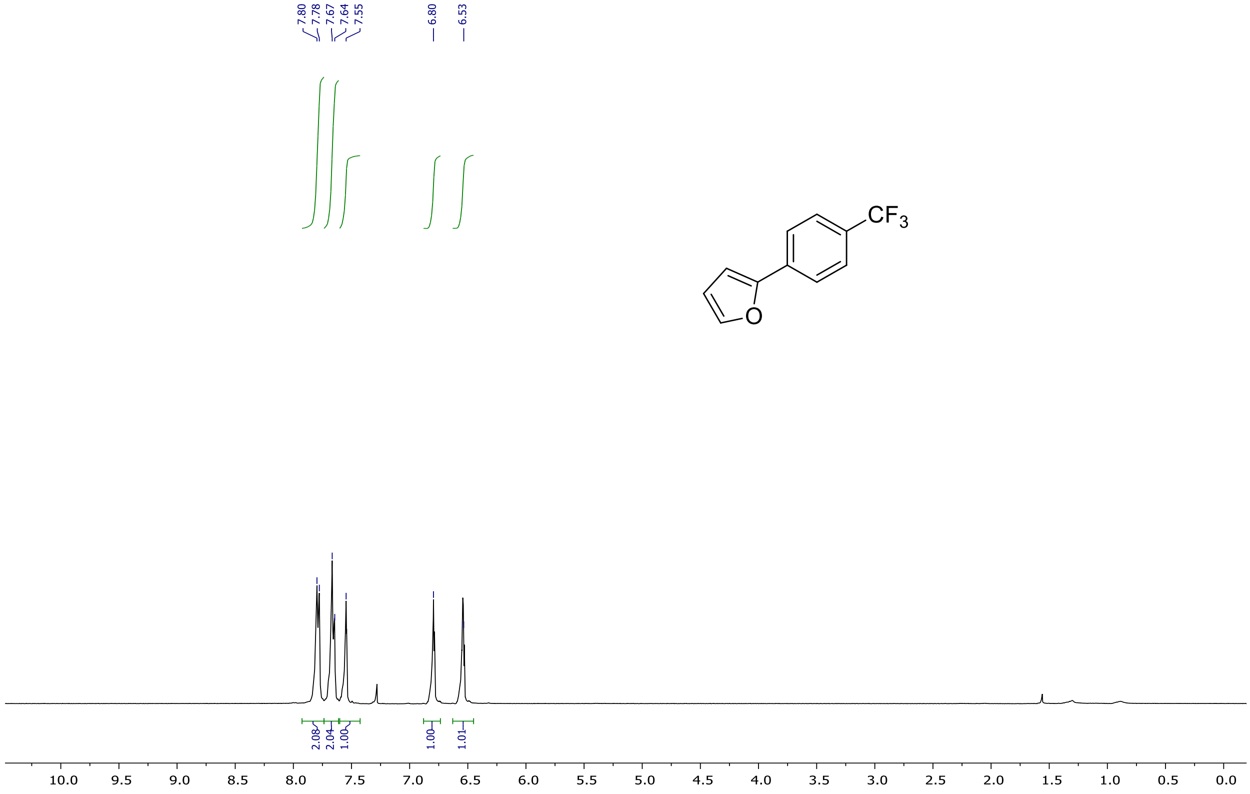

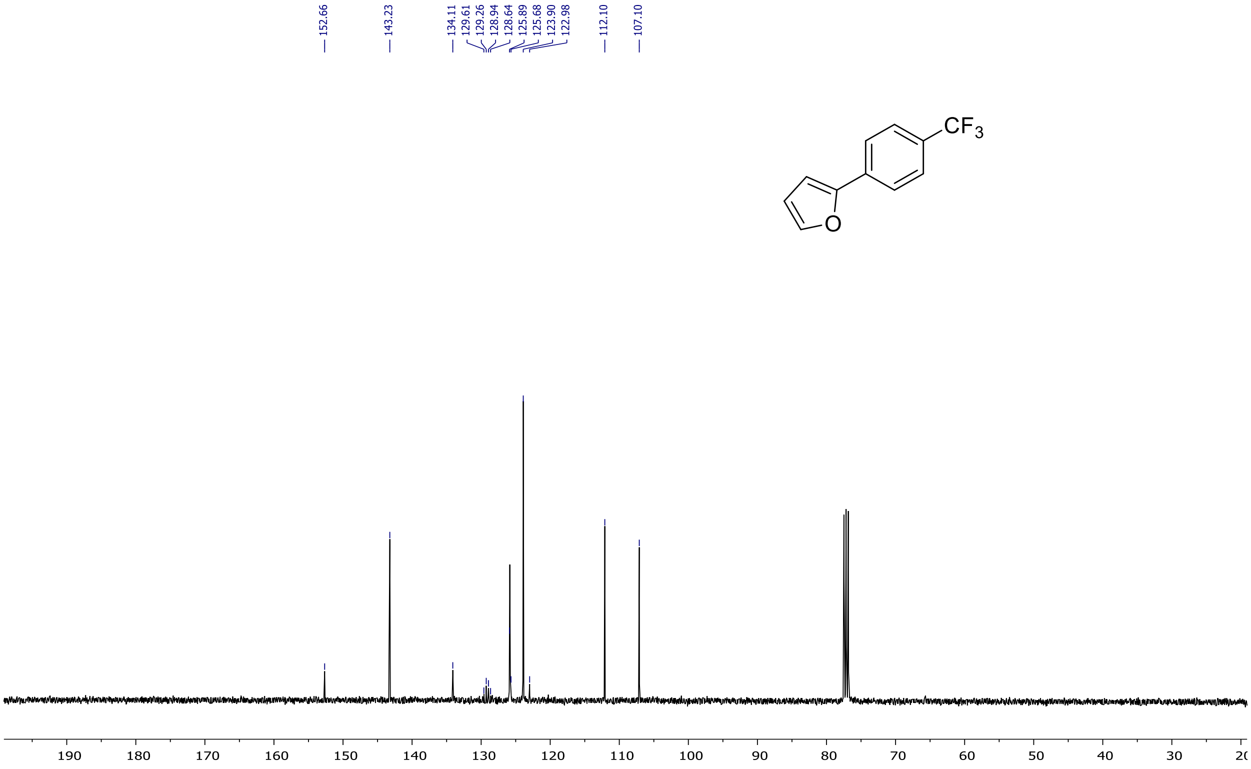

Supplementary Figure 7. ^{19}F-NMR spectrum of **1e**

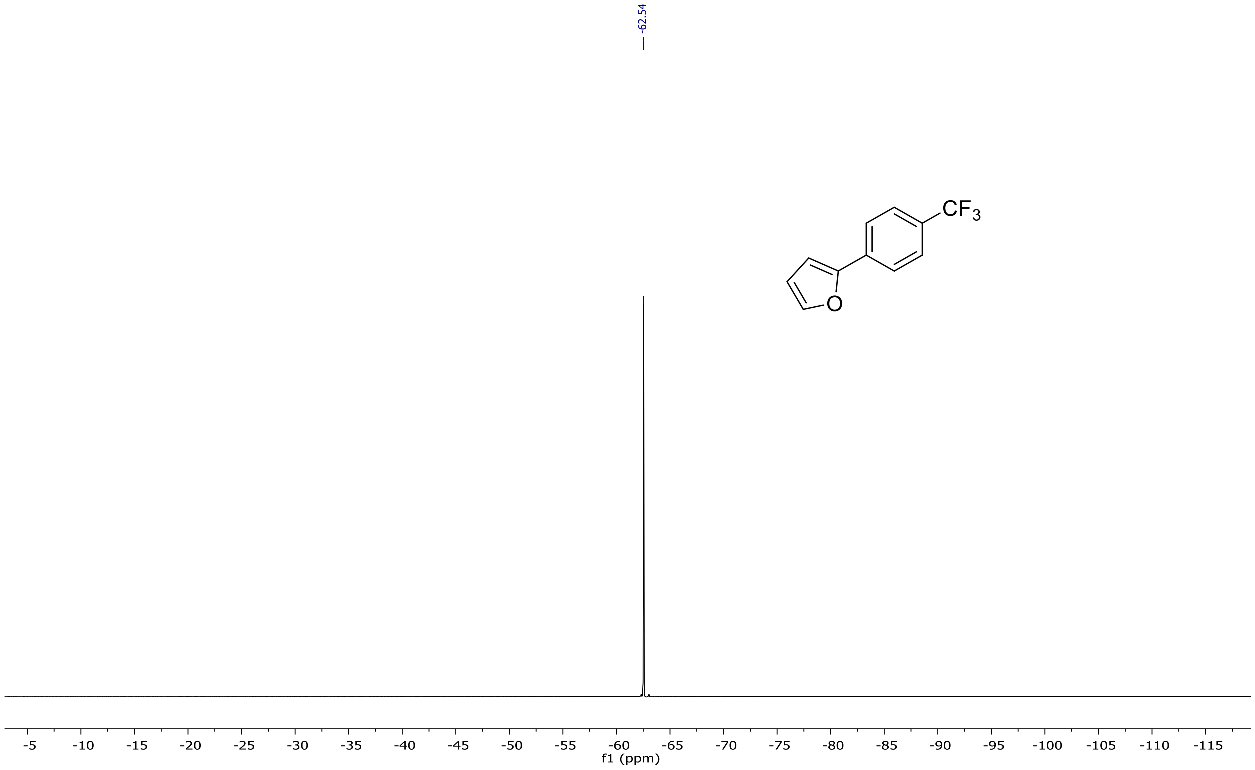

Supplementary Figure 8. ^{1}H-NMR spectrum of **1f**

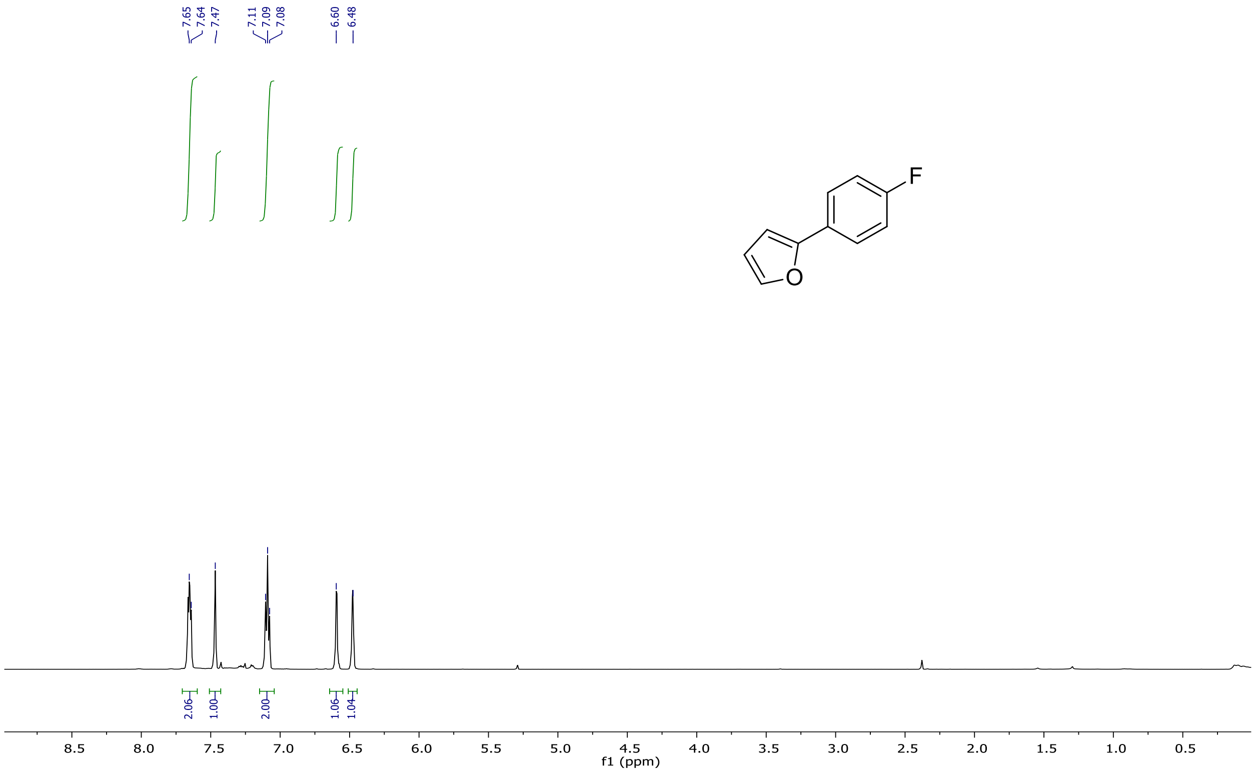

Supplementary Figure 9. ^{13}C and ^{19}F-NMR spectra of **1f**

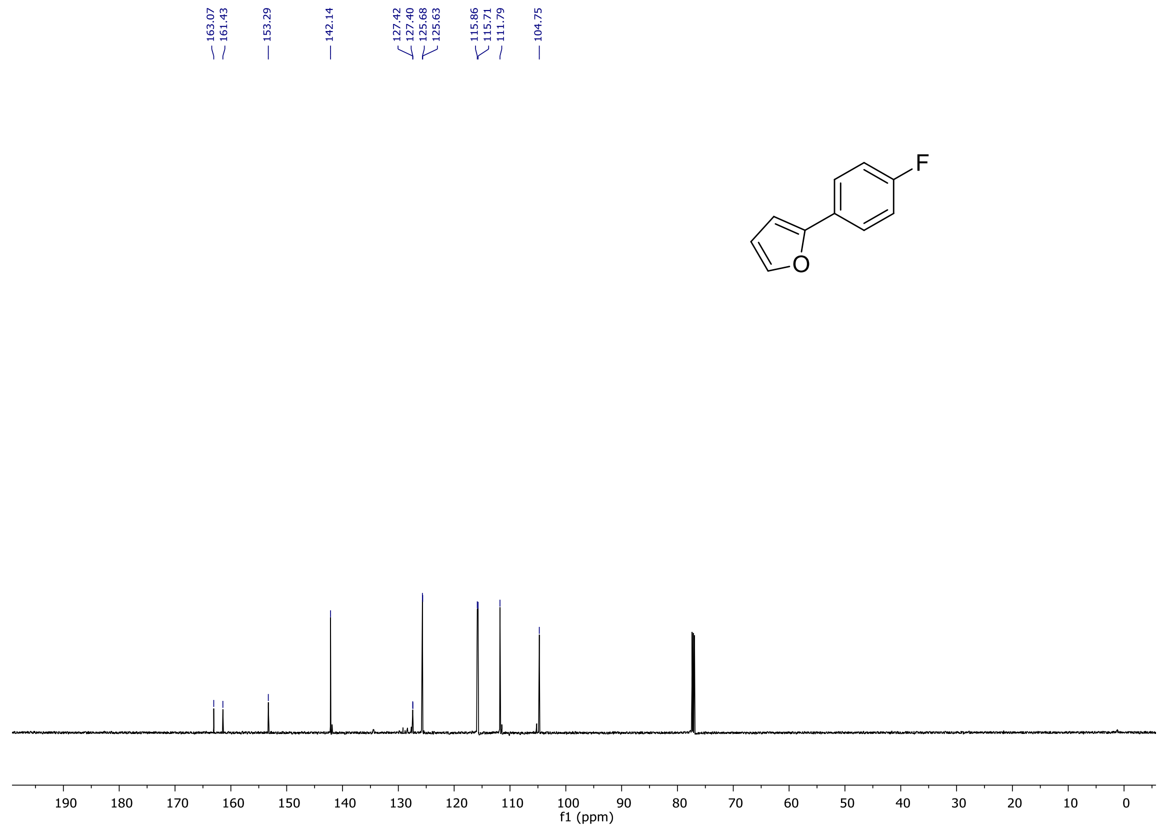

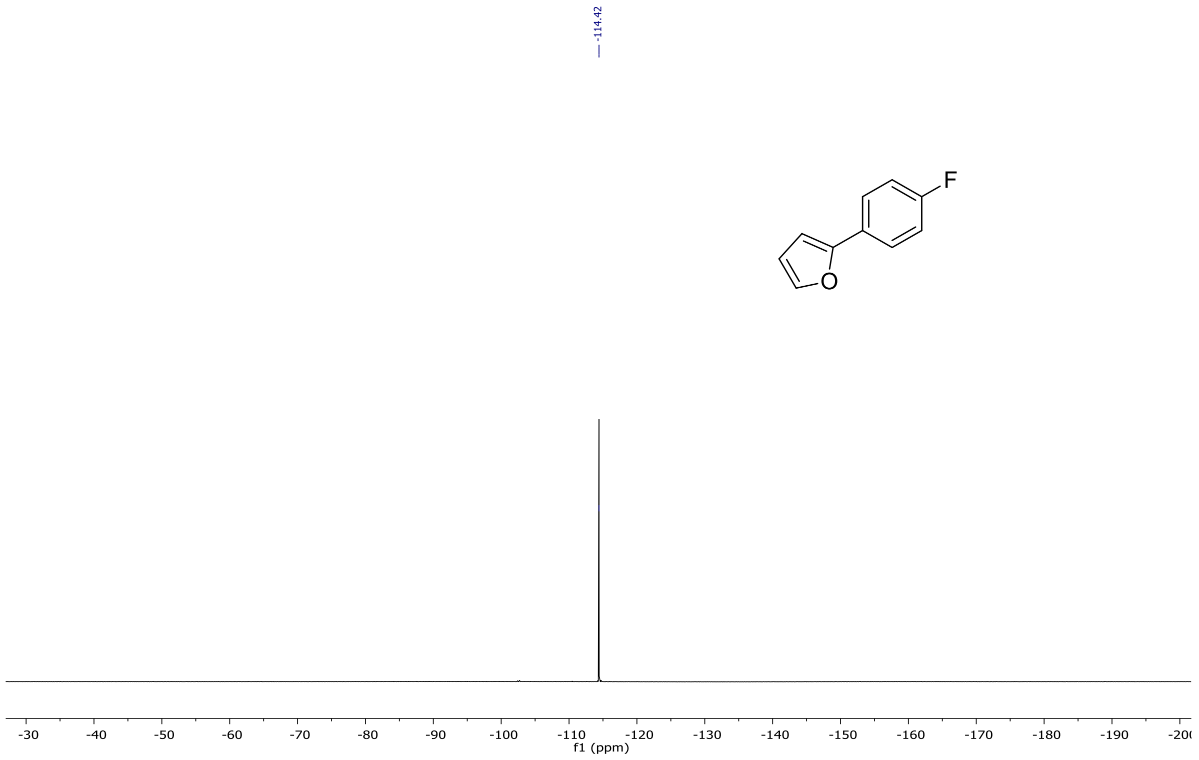

Supplementary Figure 10. ^{1}H and ^{13}C-NMR spectra of **1g**

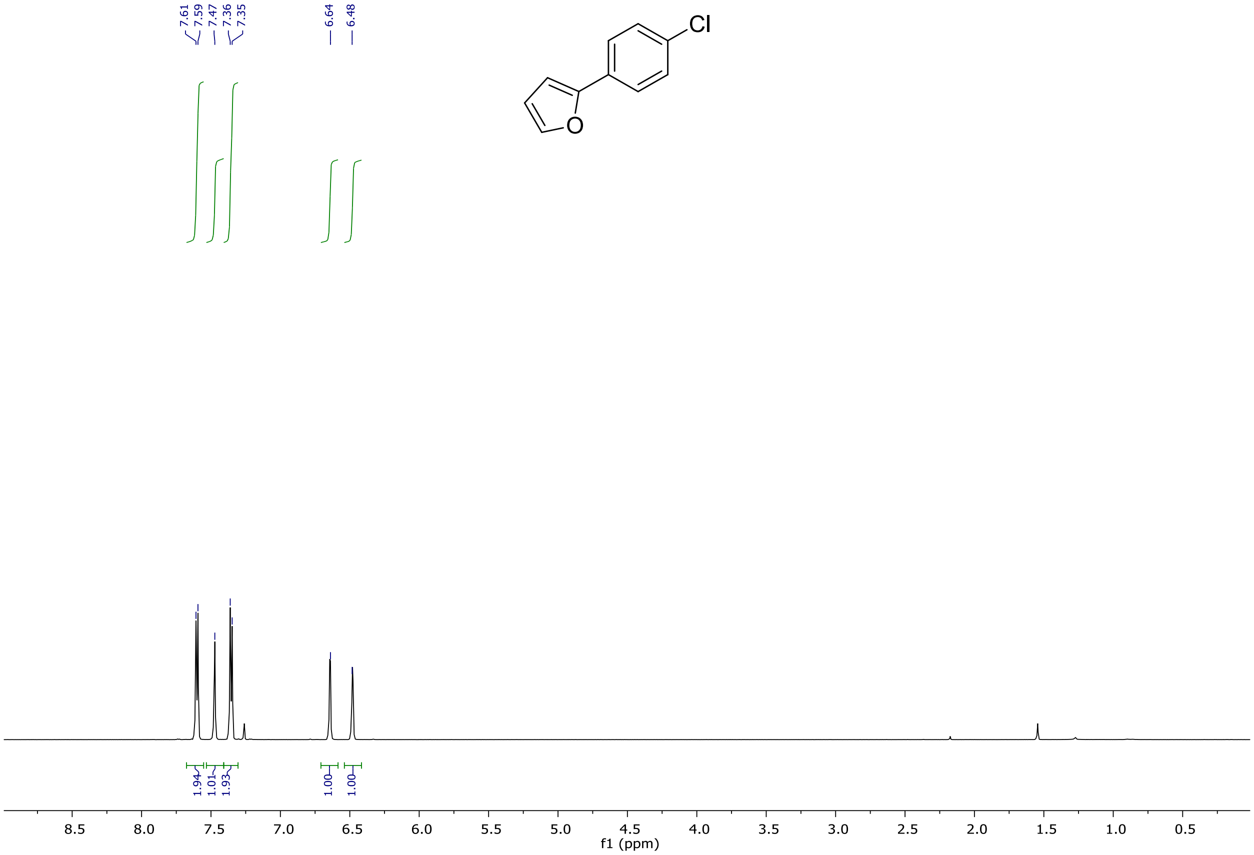

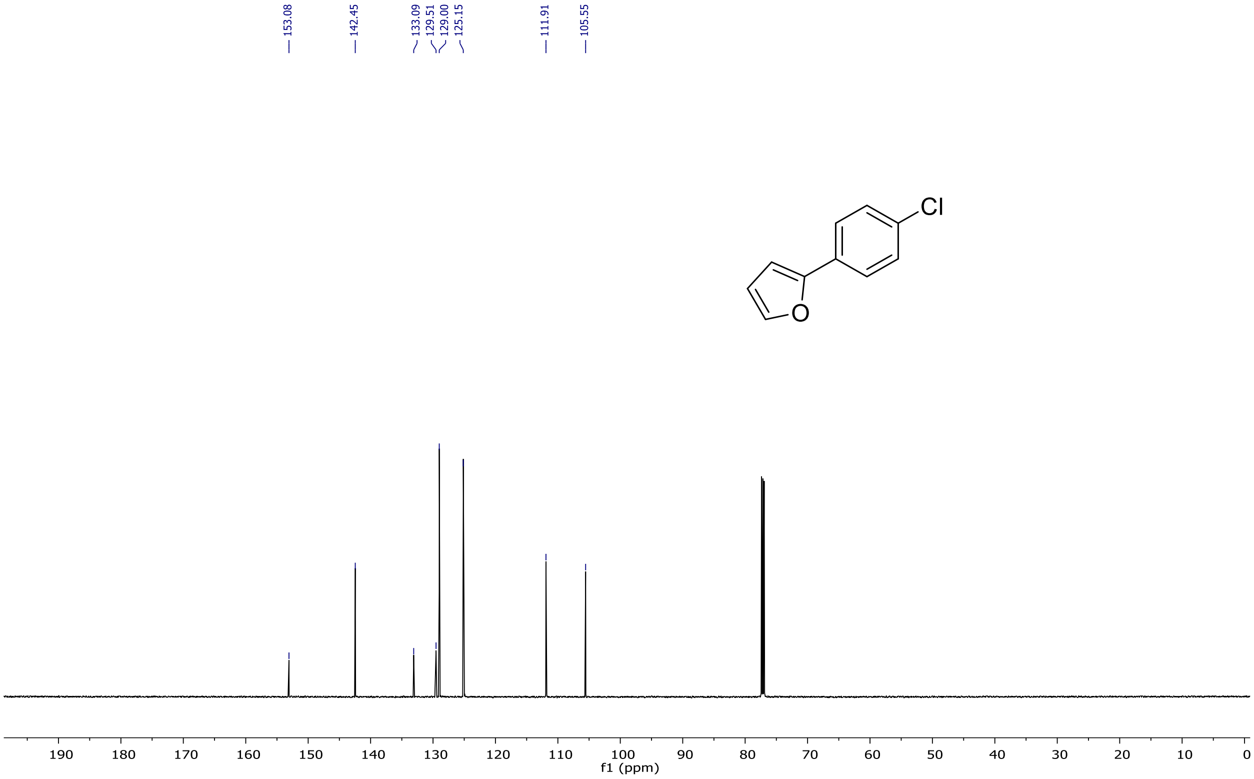

Supplementary Figure 11. ^{1}H and ^{13}C-NMR spectra of **1h**

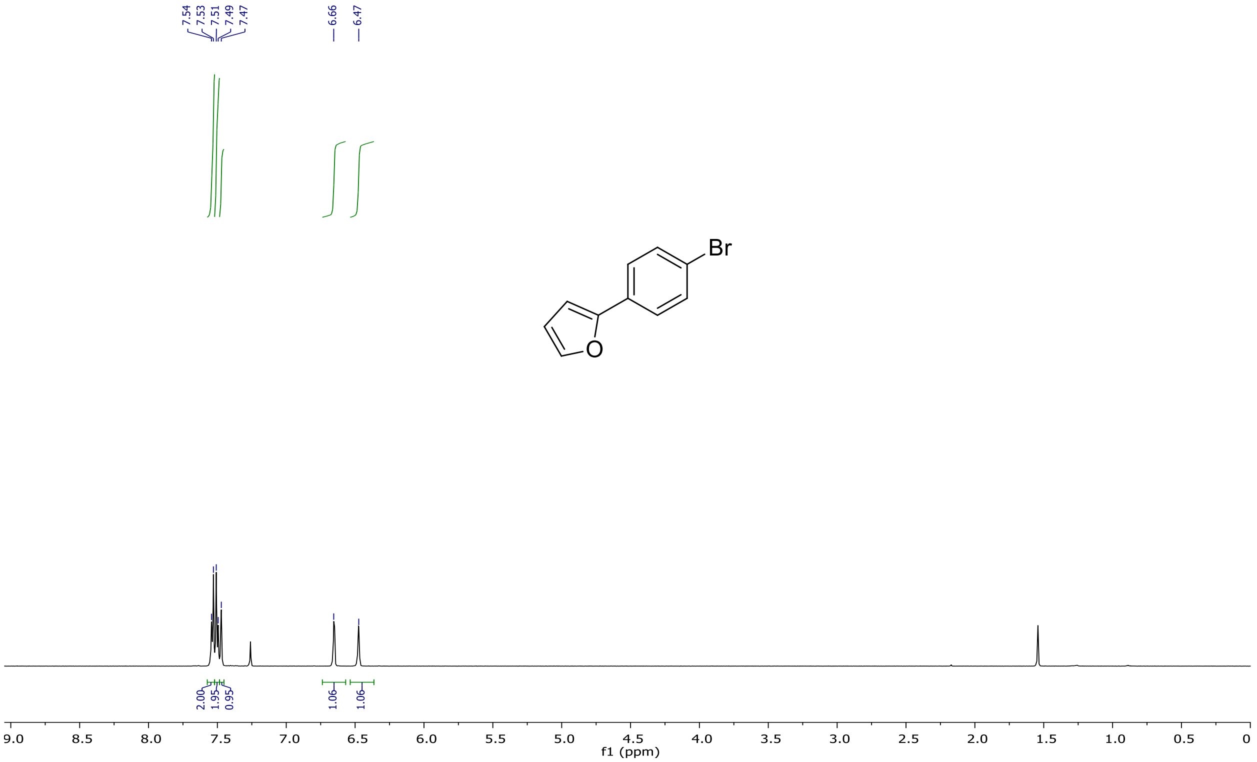

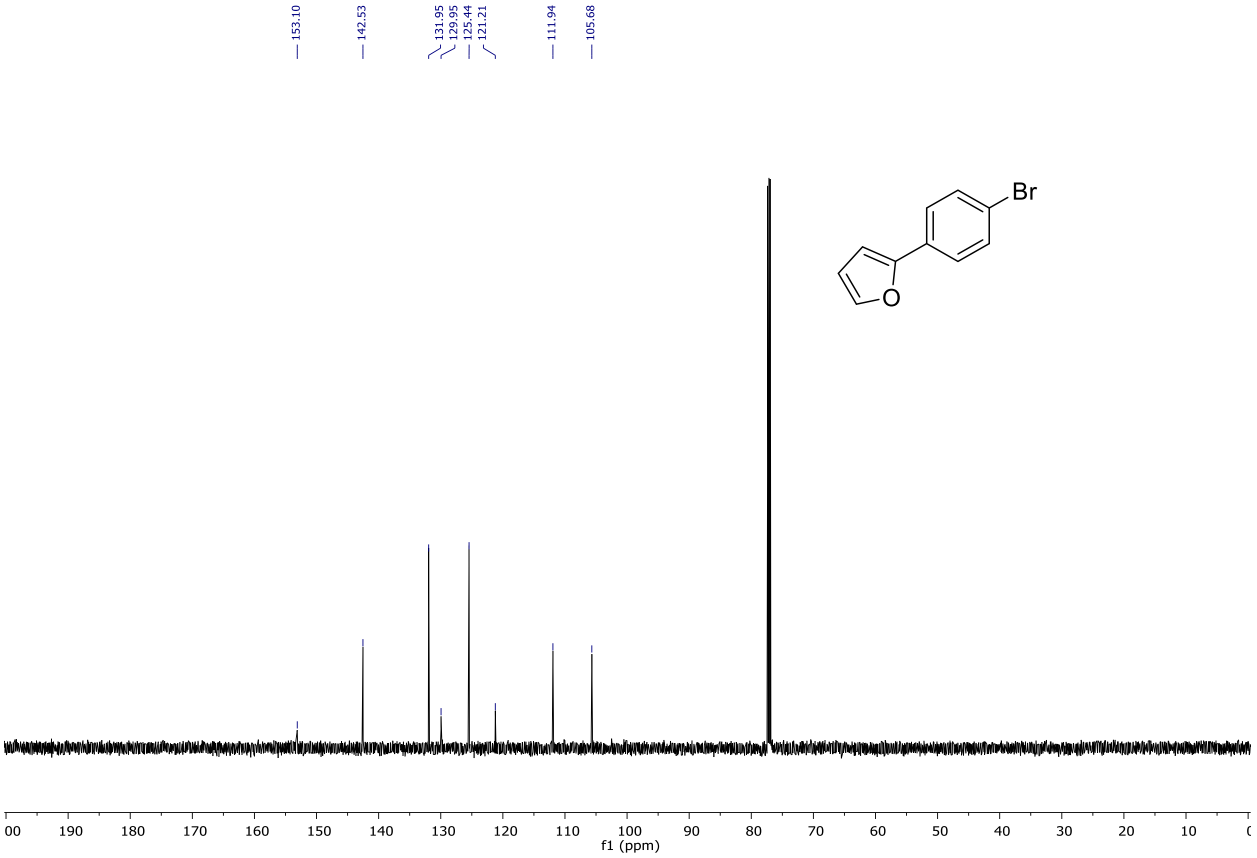

Supplementary Figure 12. ^{1}H and ^{13}C-NMR spectra of **1i**

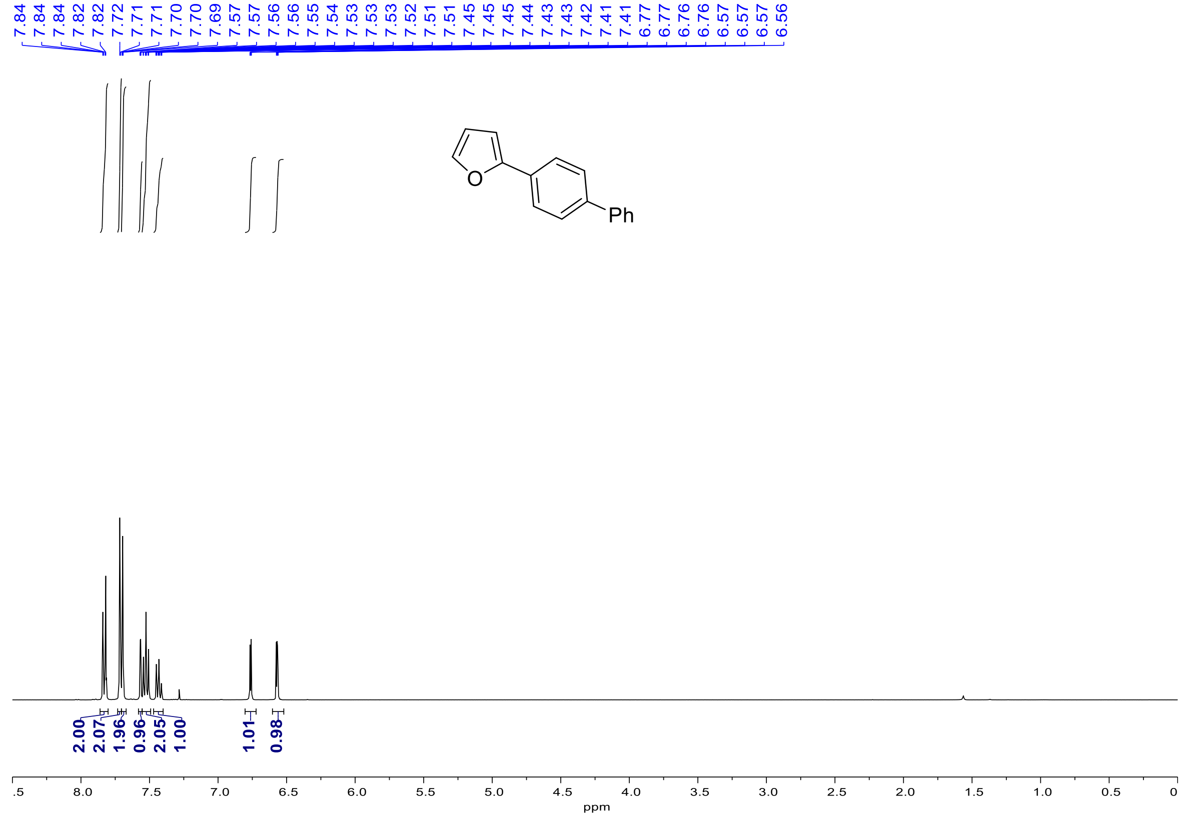

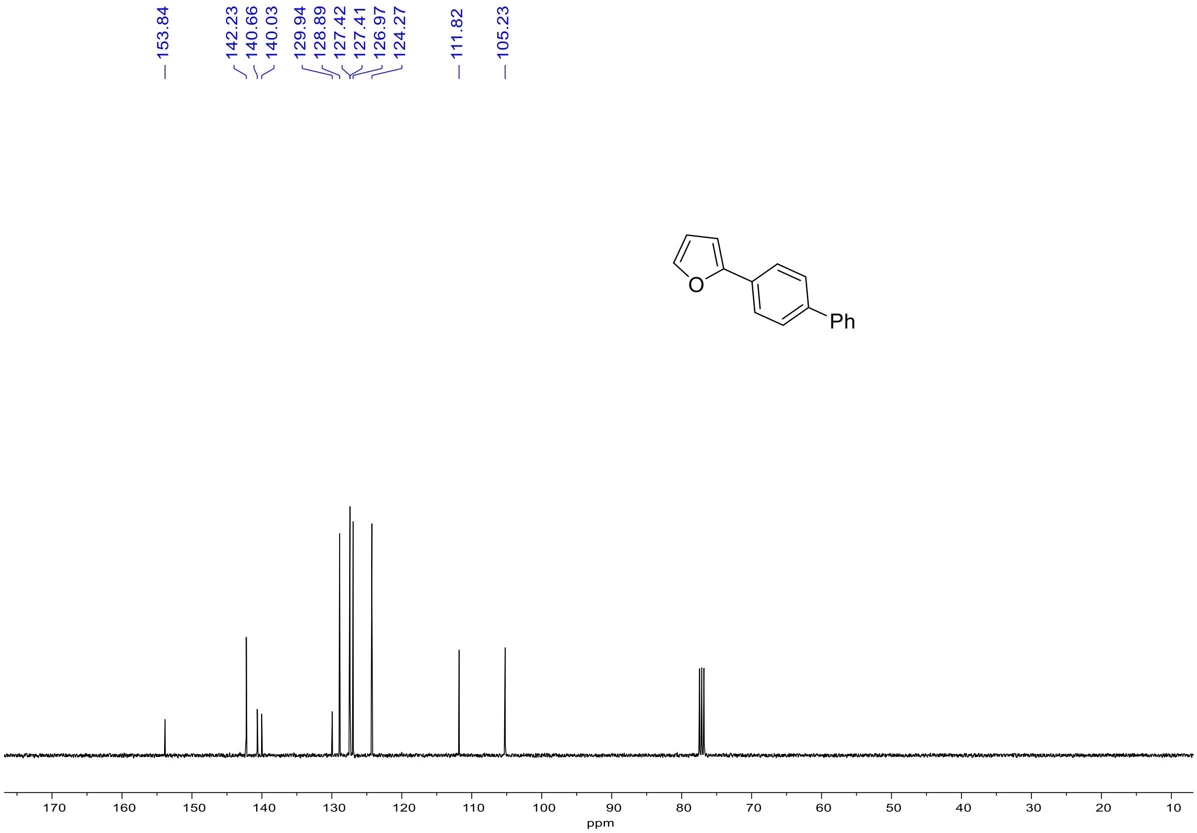

Supplementary Figure 13. ^{1}H and ^{13}C-NMR spectra of **1j**

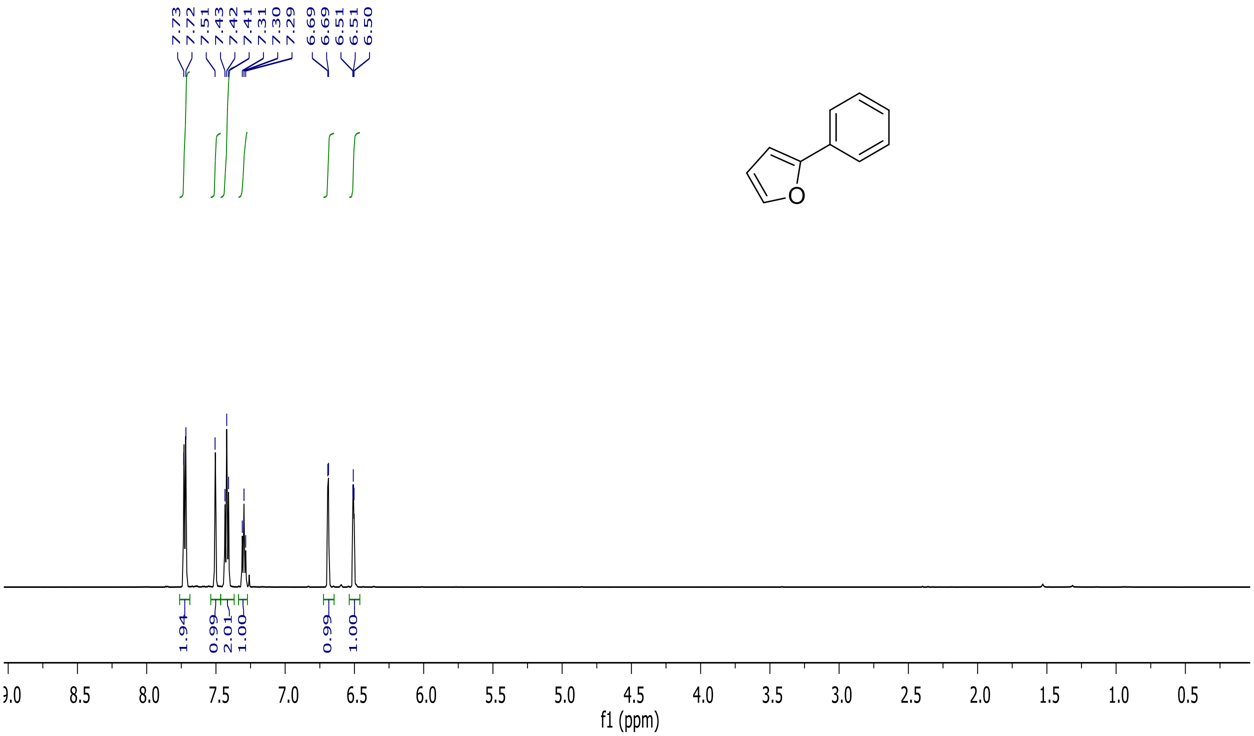

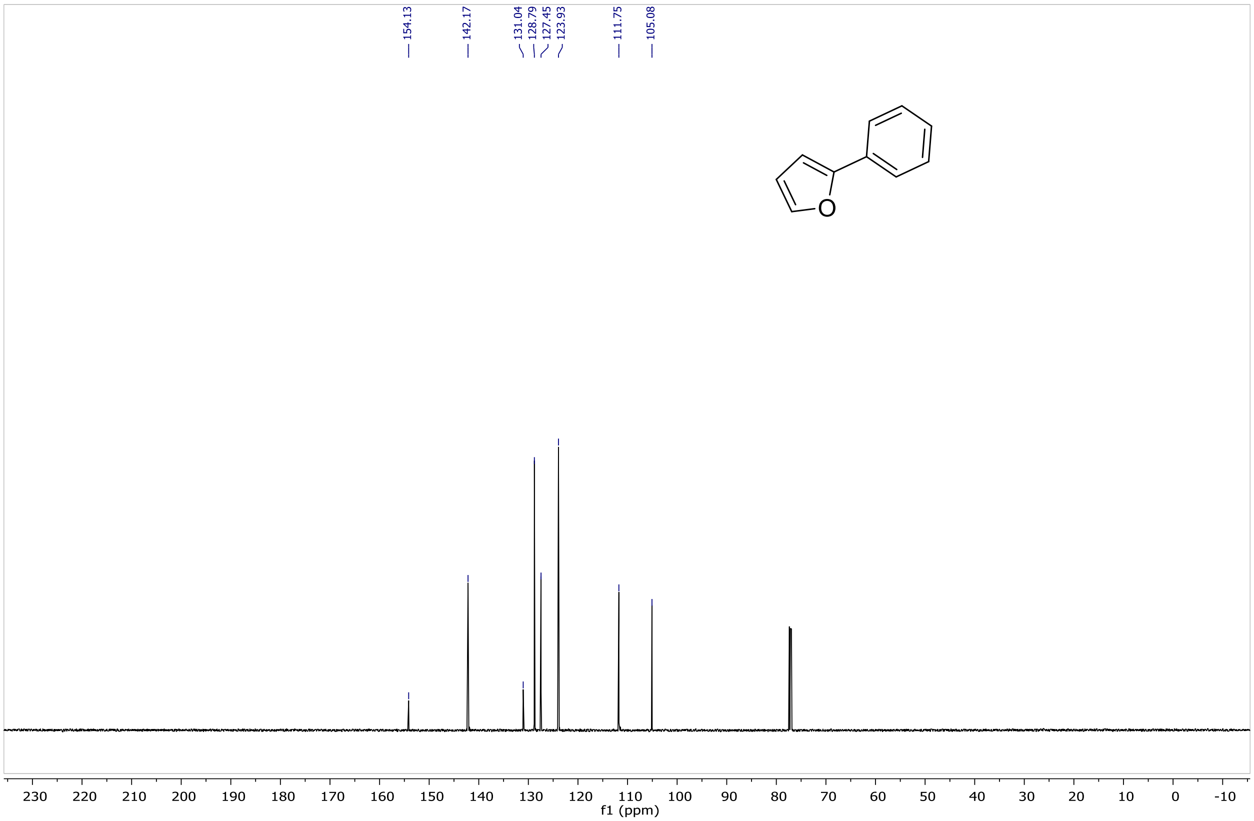

Supplementary Figure 14. ^{1}H and ^{13}C-NMR spectra of **1k**

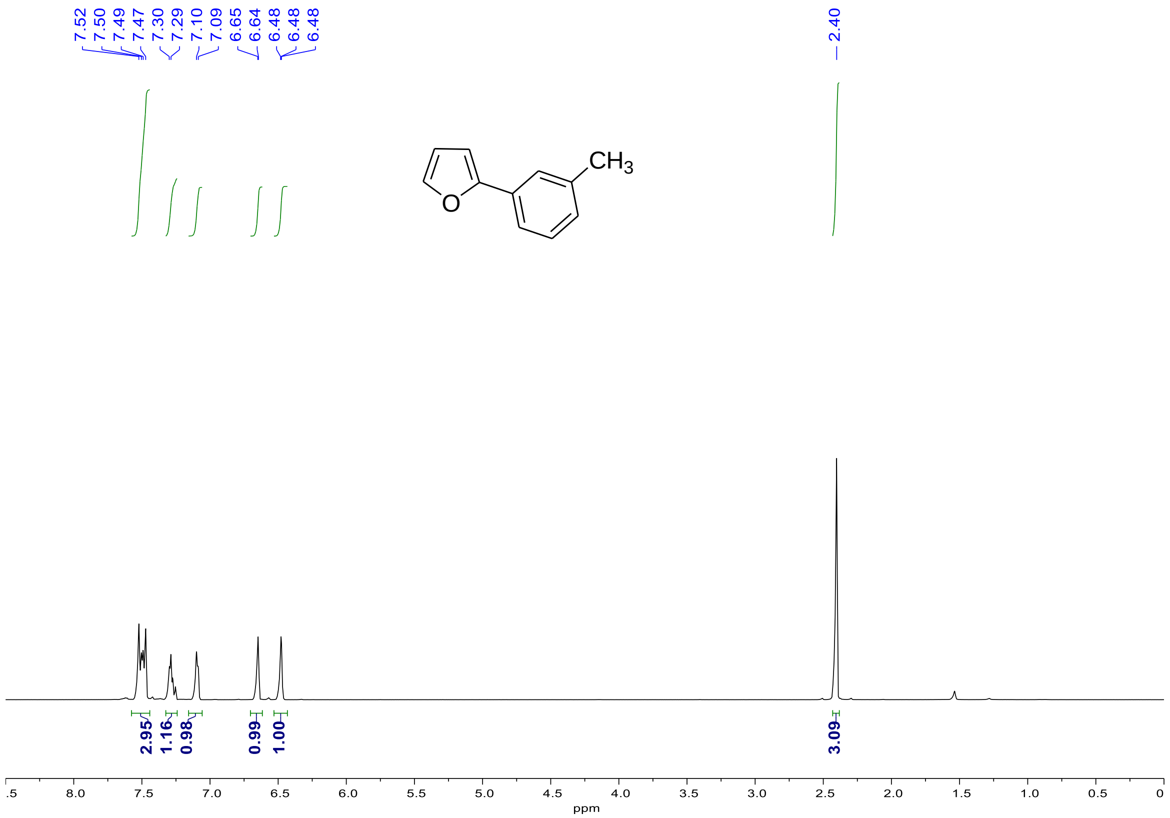

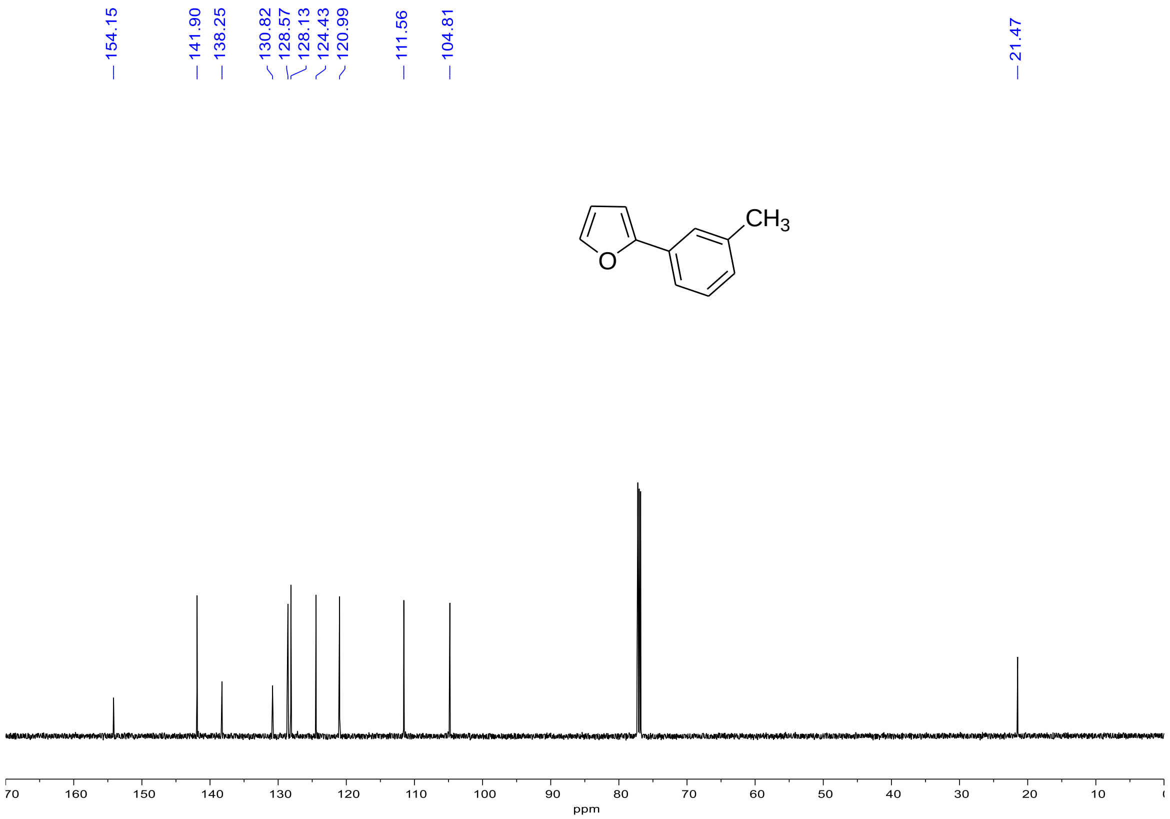

Supplementary Figure 15. ^{1}H and ^{13}C-NMR spectra of **1l**

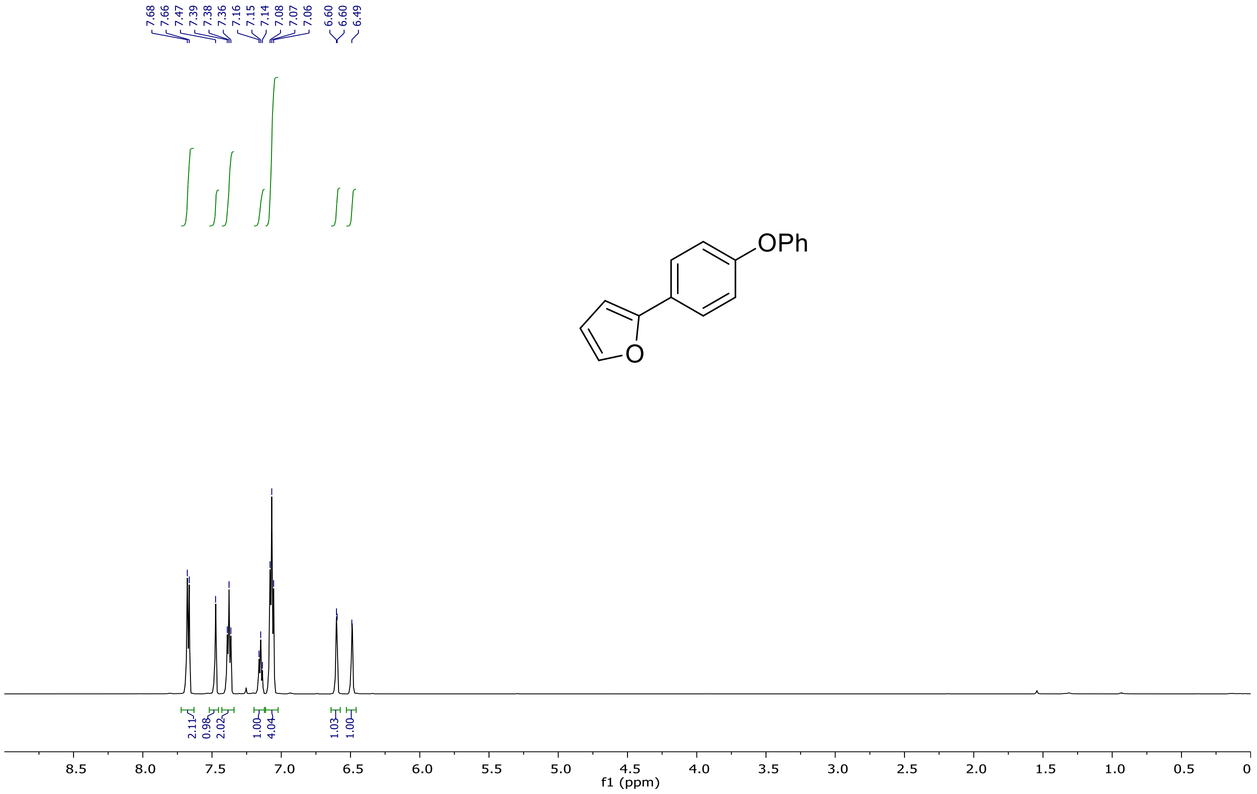

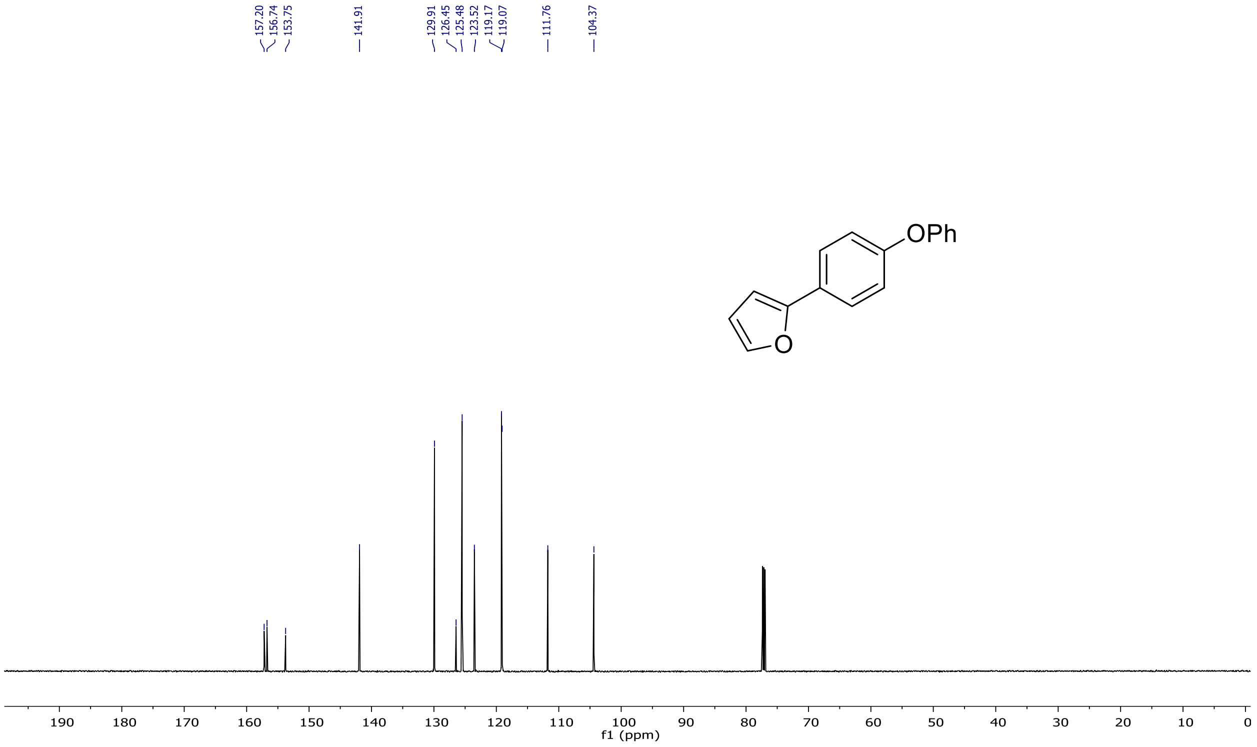

Supplementary Figure 16. ^{1}H and ^{13}C-NMR spectra of **1m**

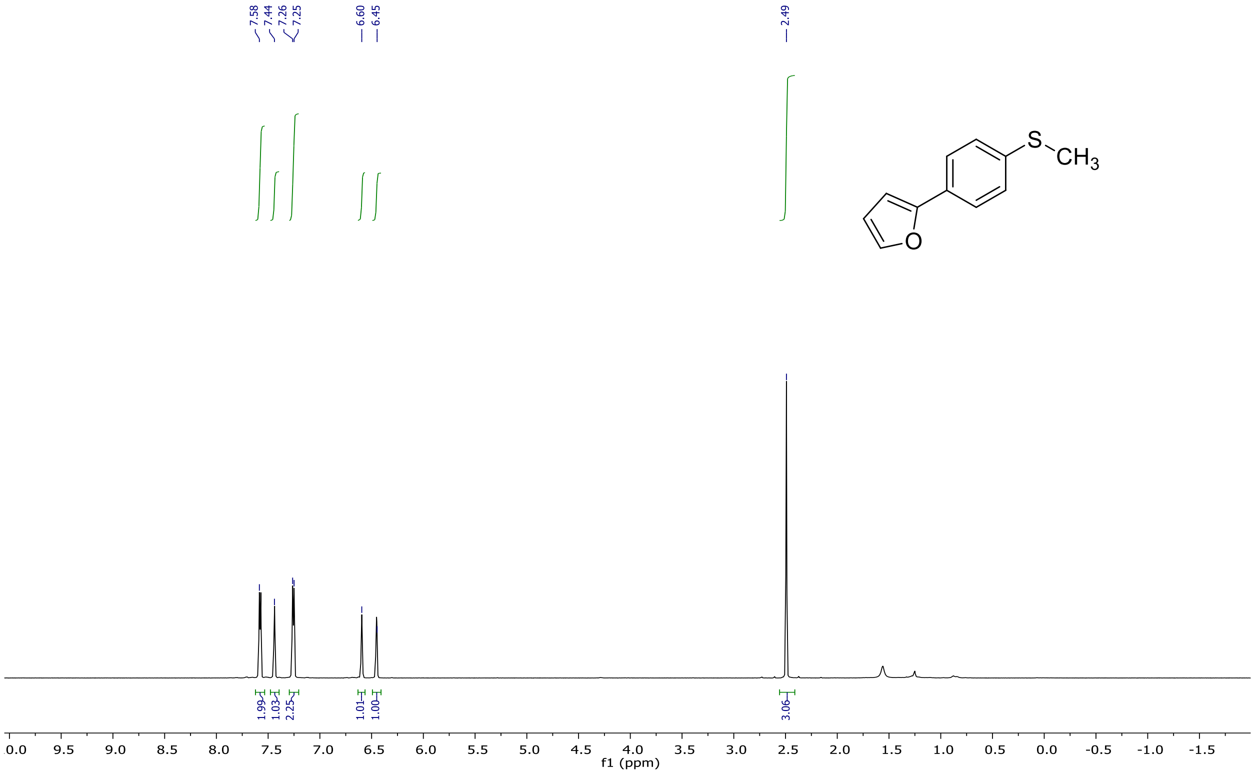

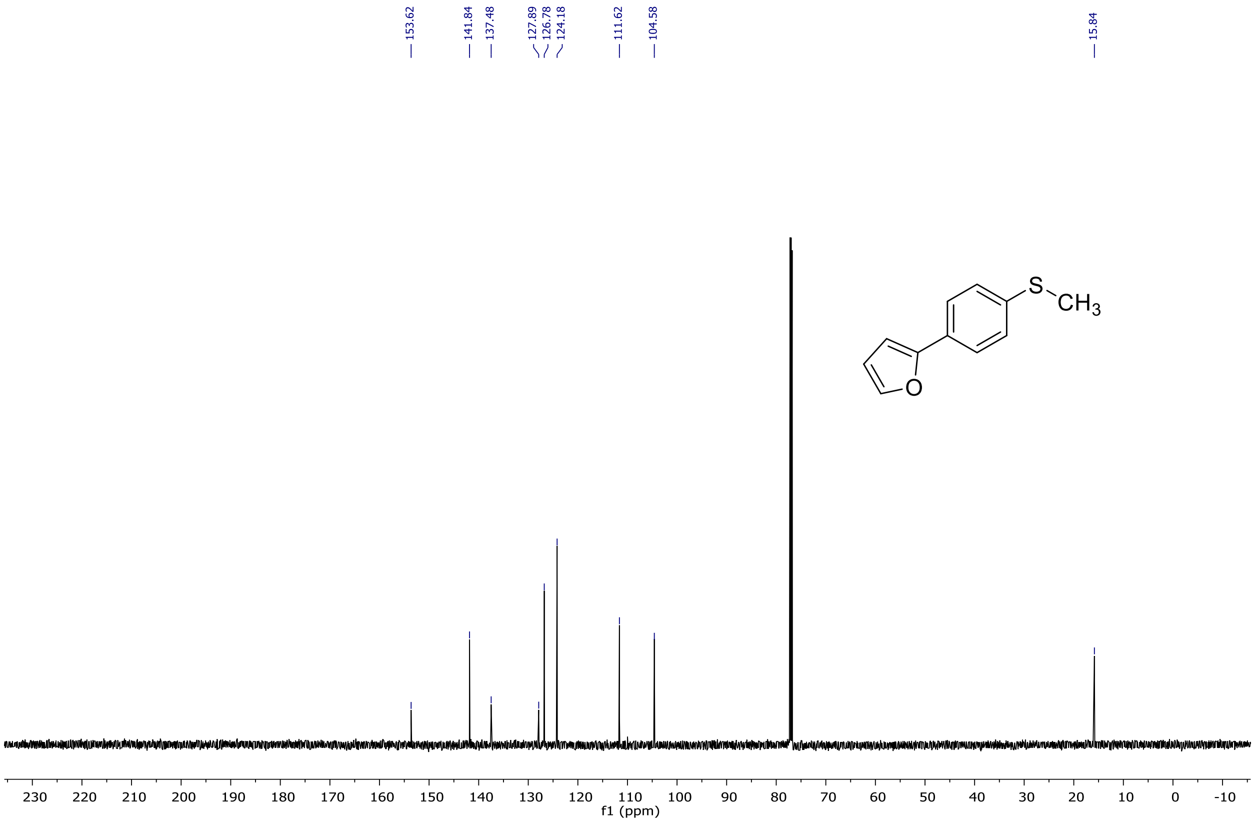

Supplementary Figure 17. ^{1}H and ^{13}C-NMR spectra of **1n**

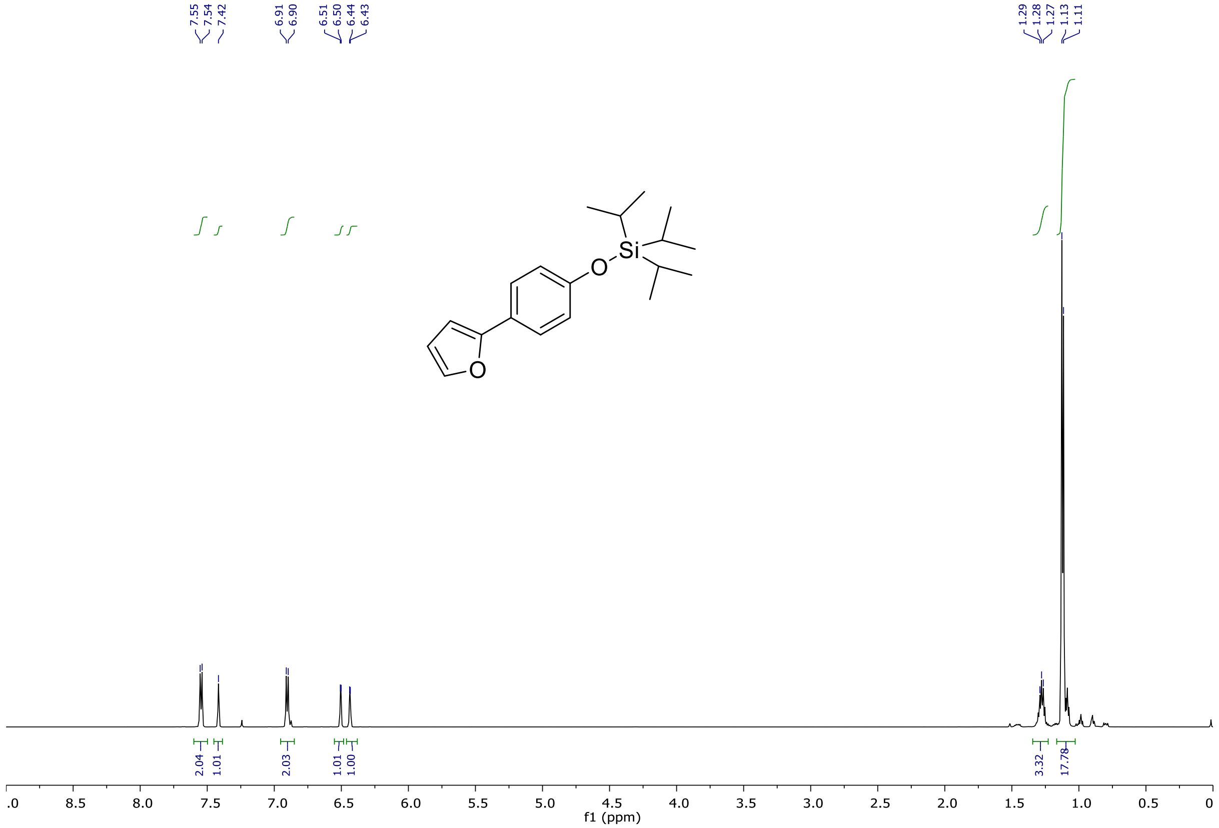

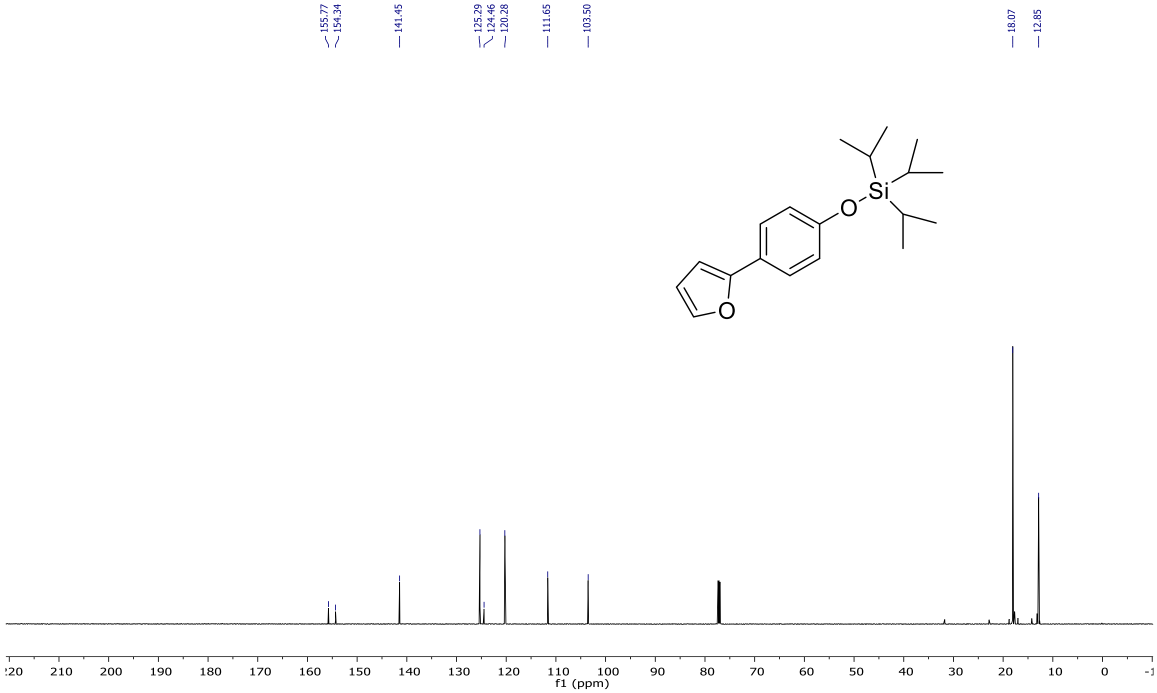

Supplementary Figure 18. ^{29}Si-NMR spectrum of **1n**

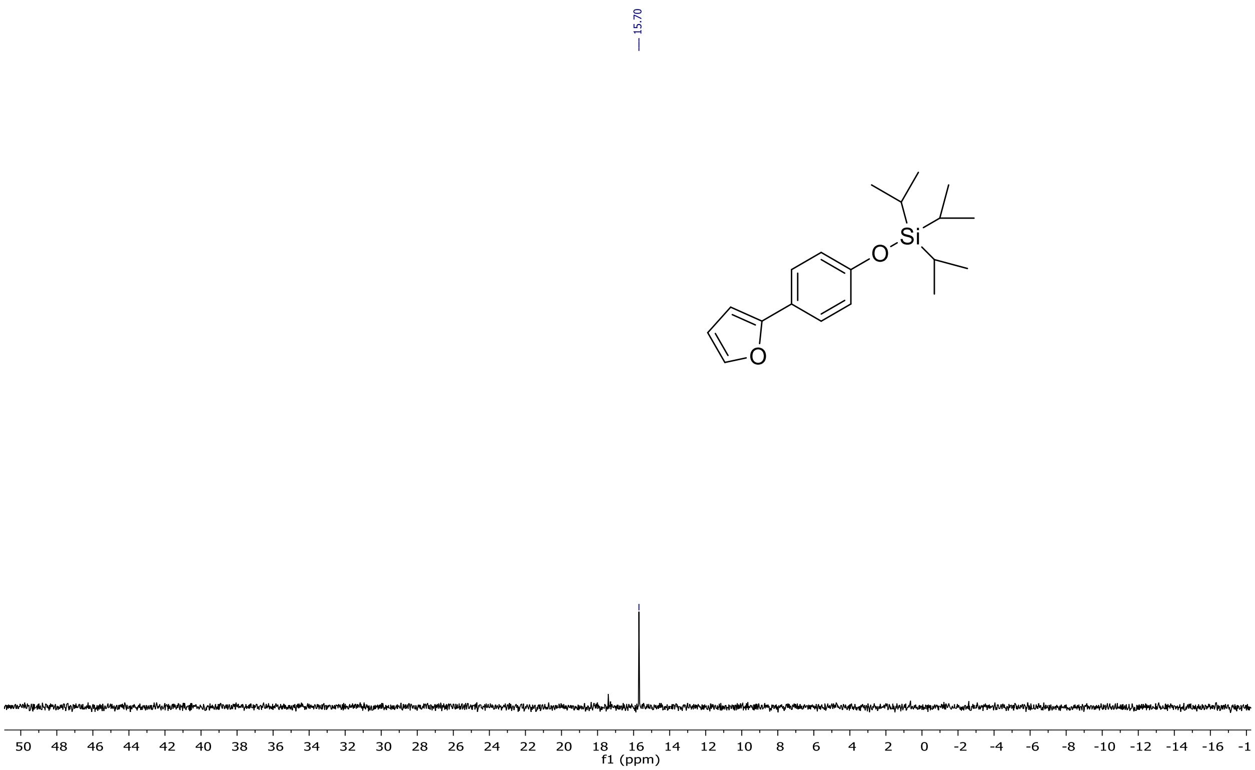

Supplementary Figure 19. ^{1}H-NMR spectrum of **1o**

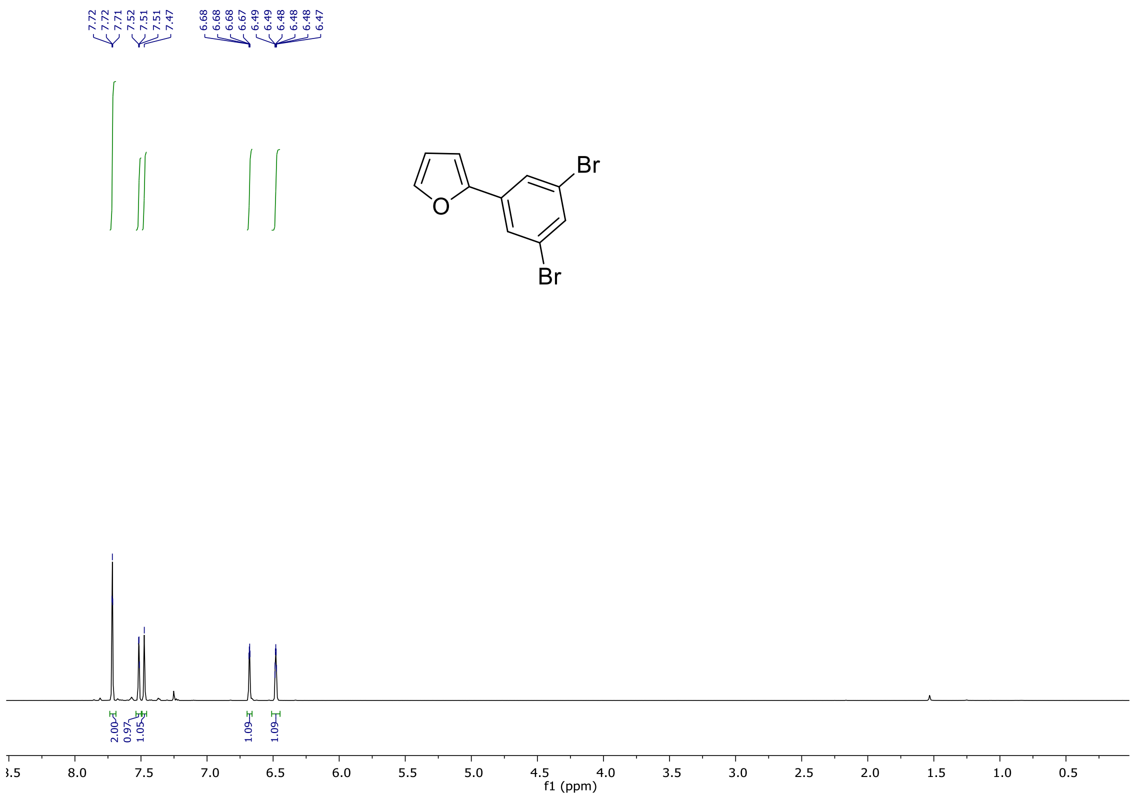

Supplementary Figure 20. ^{13}C-NMR spectrum of **1o**

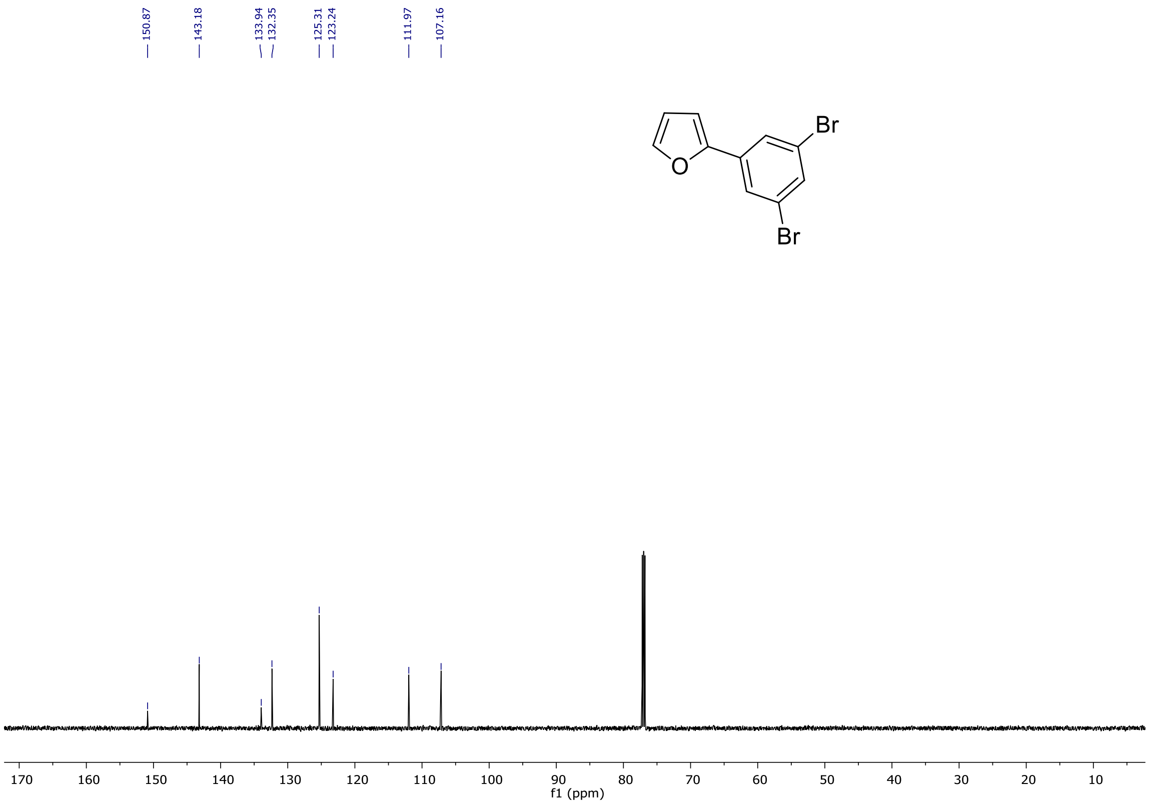

Supplementary Figure 21. ^{1}H-NMR spectrum of **1p**

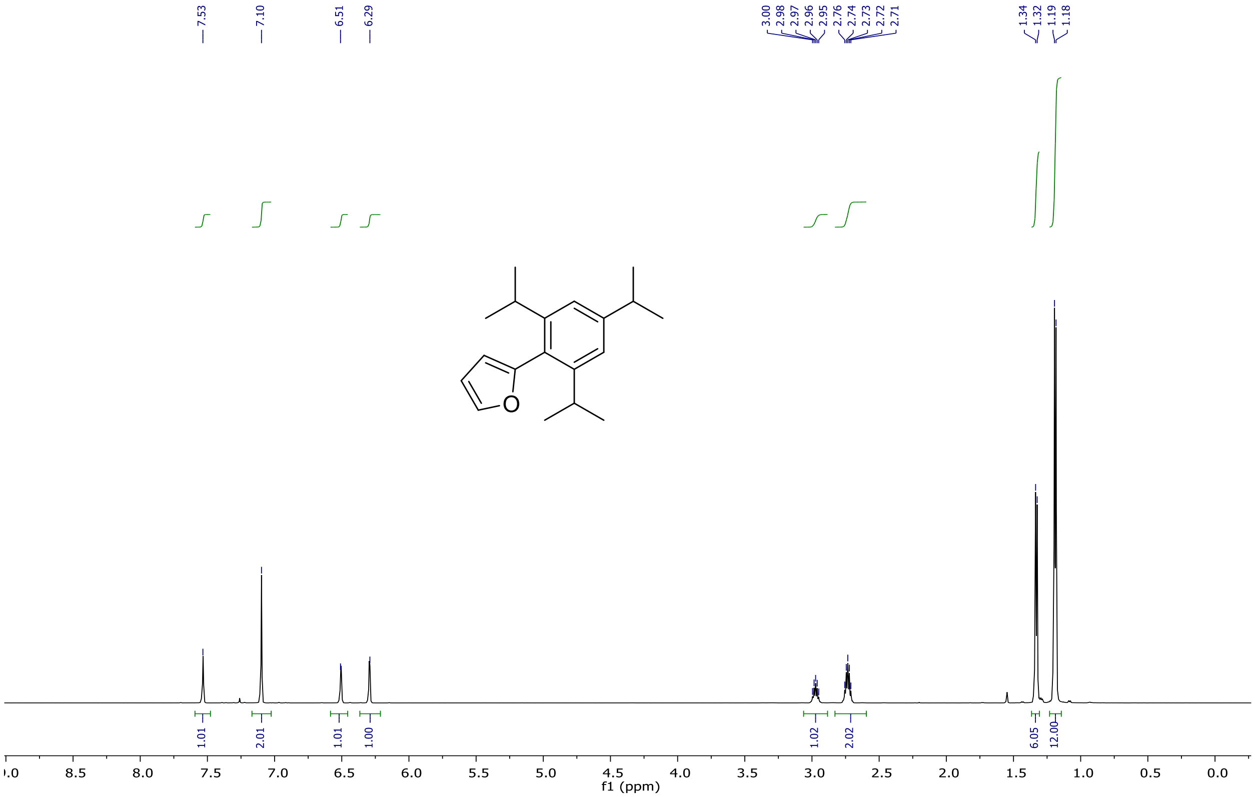

Supplementary Figure 22. ^{13}C-NMR spectrum of **1p**

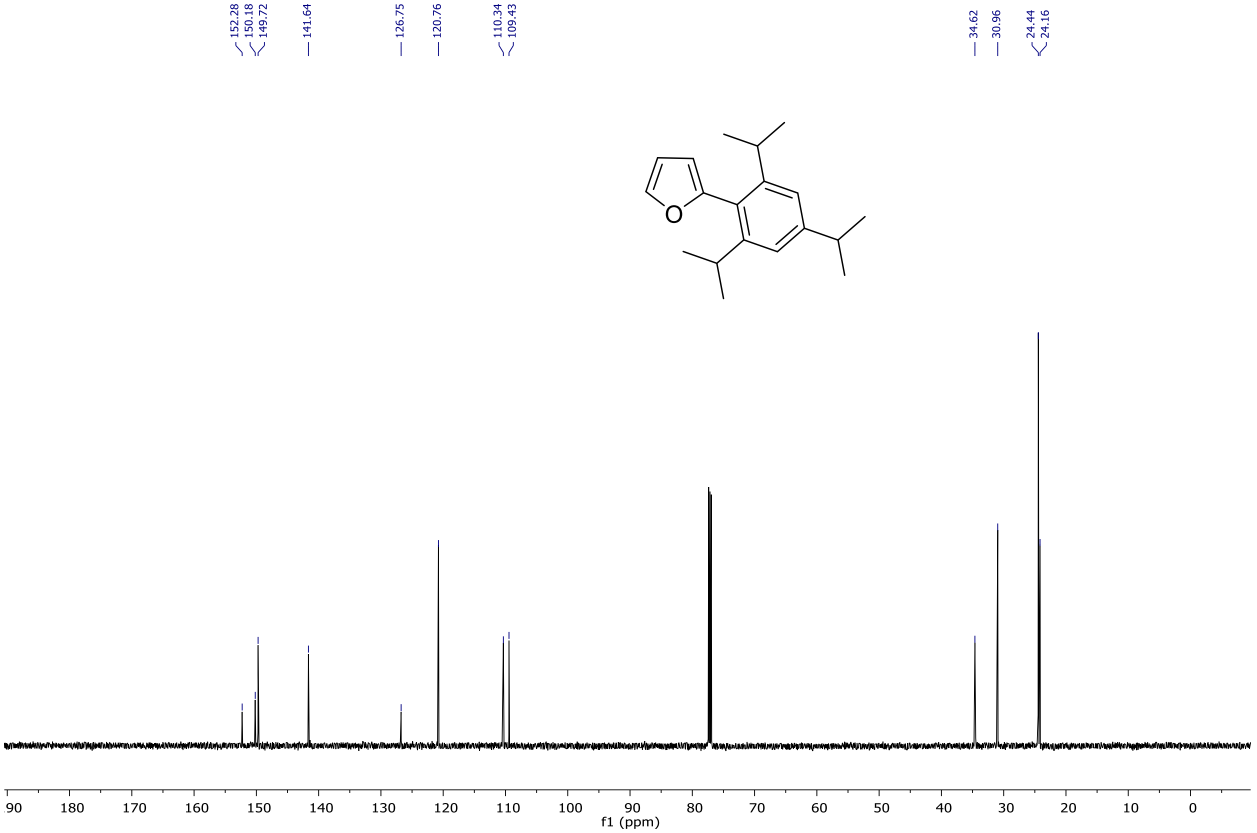

Supplementary Figure 23. ^{1}H-NMR spectrum of **1q**

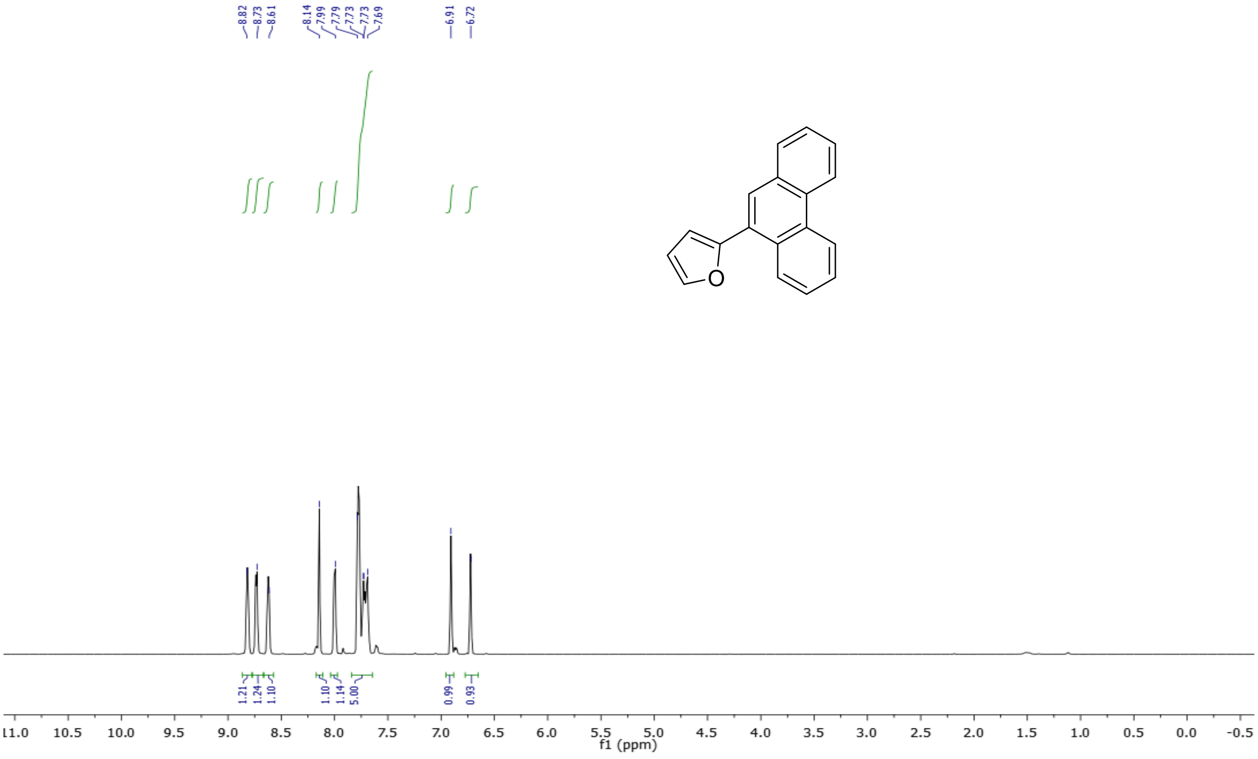

Supplementary Figure 24. ^{13}C-NMR spectrum of **1q**

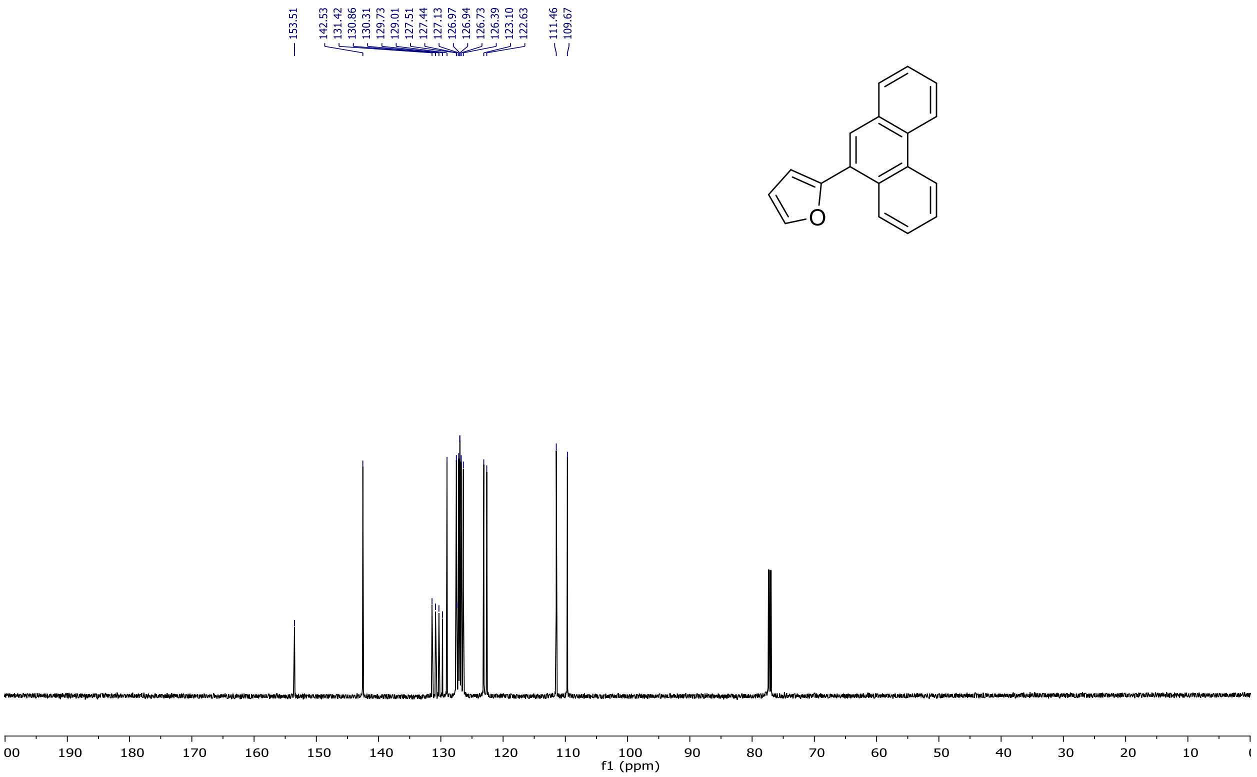

Supplementary Figure 25. ^{1}H-NMR spectrum of **1r**

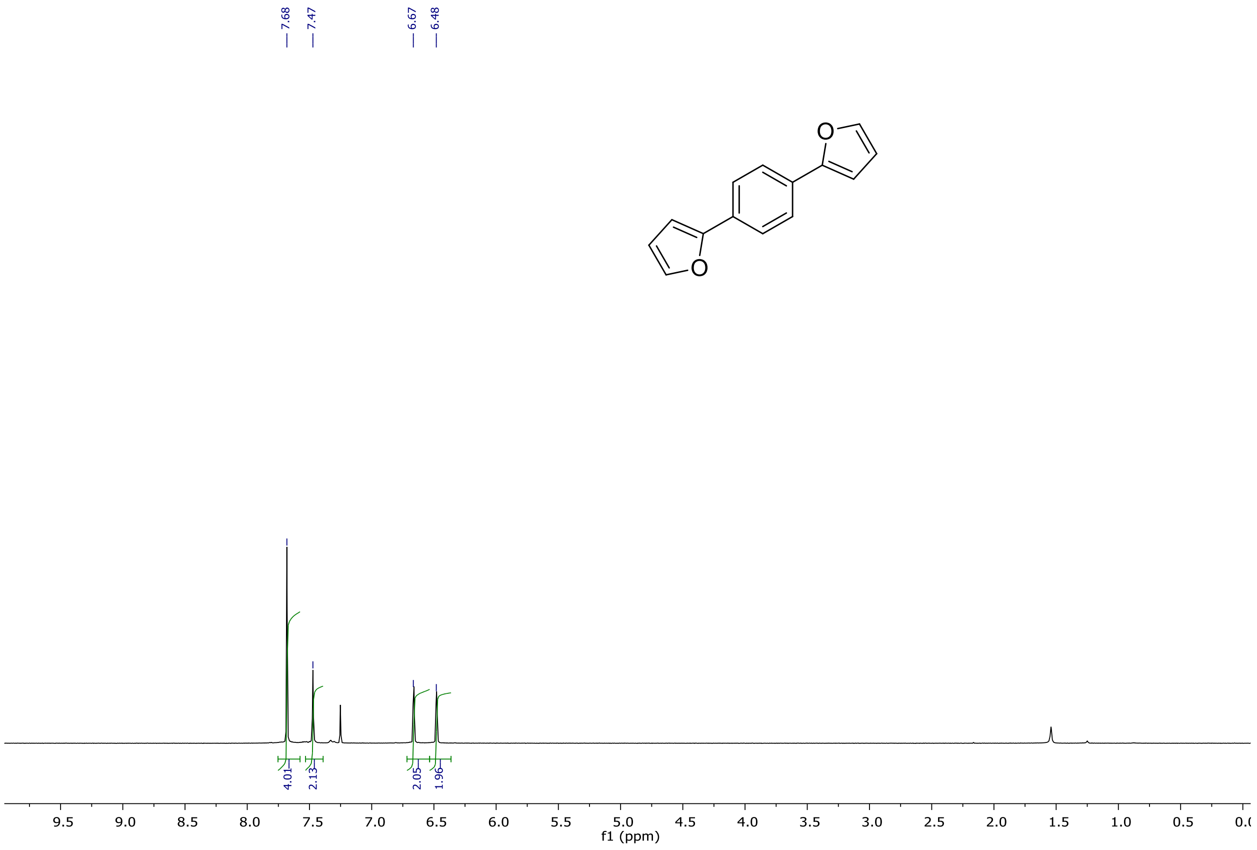

Supplementary Figure 26. ^{13}C-NMR spectrum of **1r**

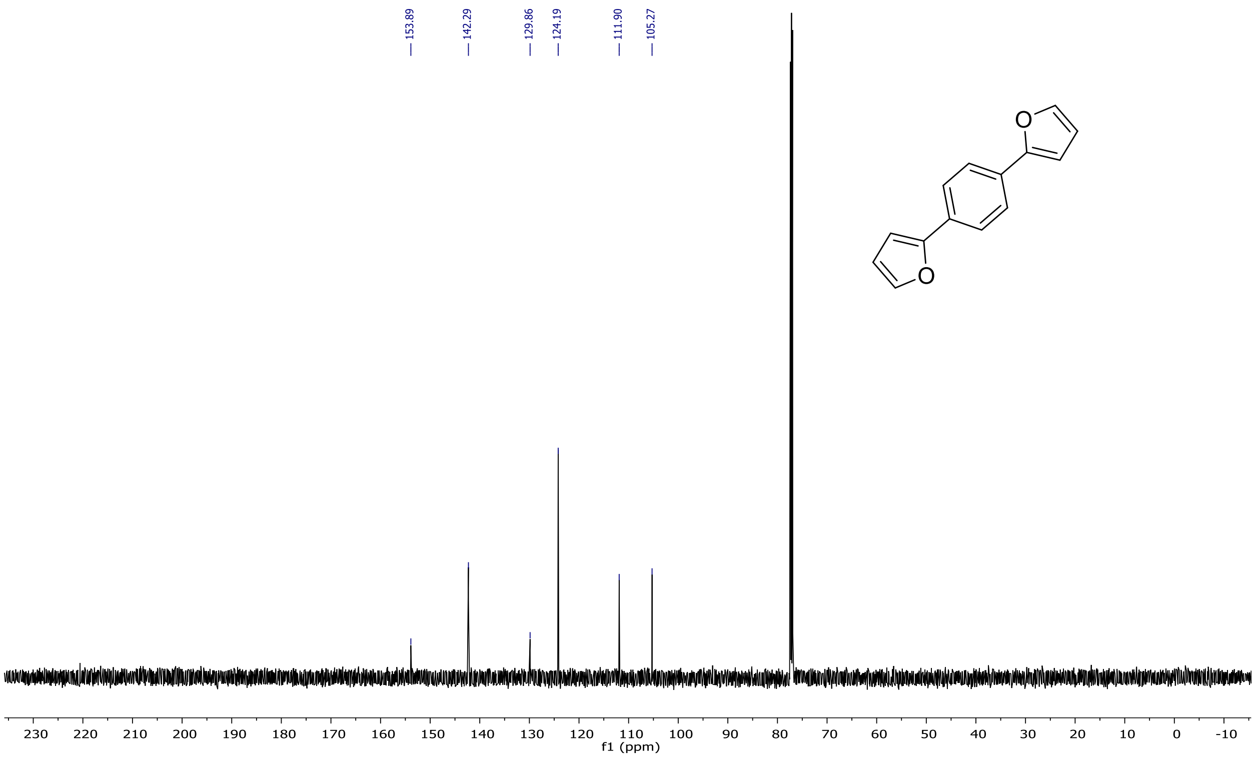

Supplementary Figure 27. ^{1}H-NMR spectrum of **1s**

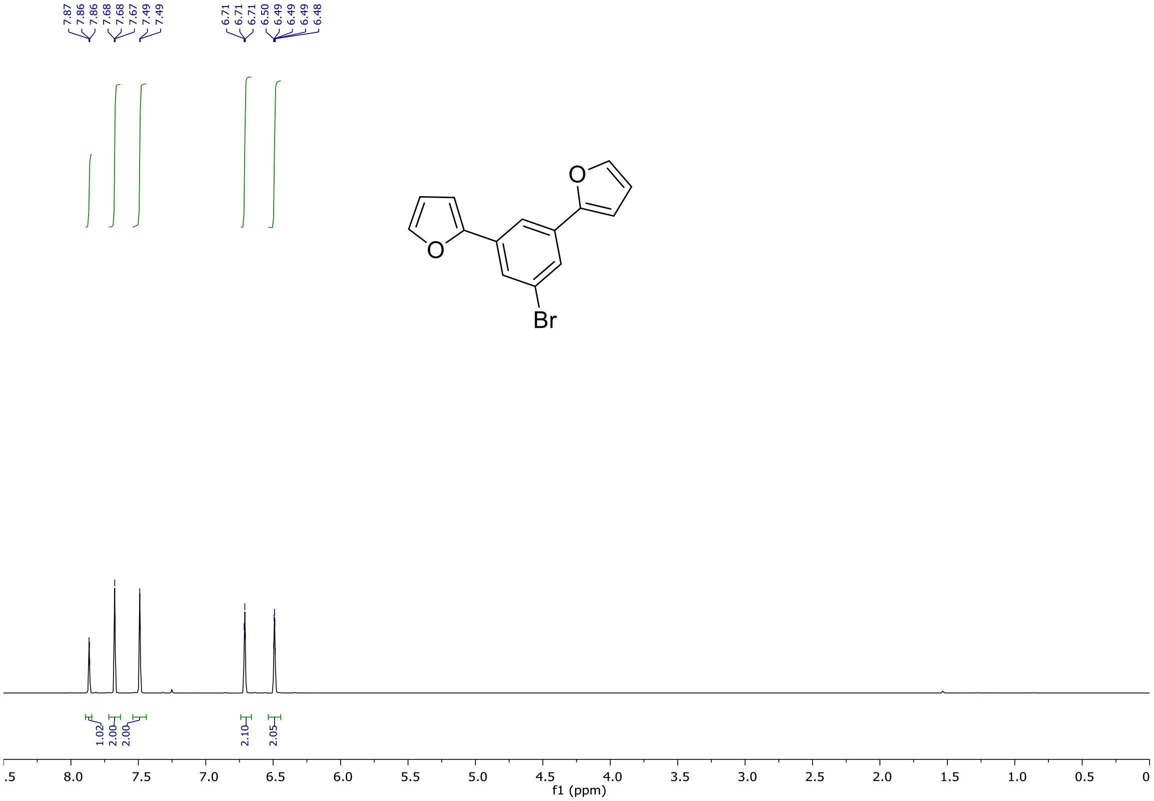

Supplementary Figure 28. ^{13}C-NMR spectrum of **1s**

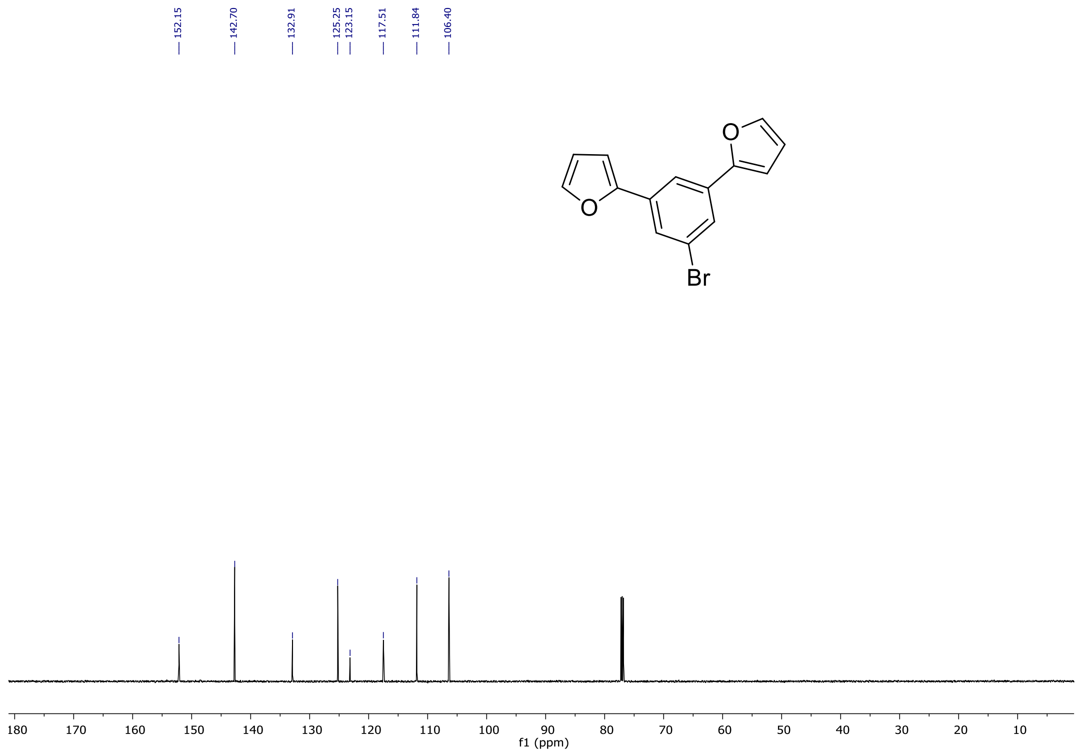

Supplementary Figure 29. ^{1}H-NMR spectrum of **1t**

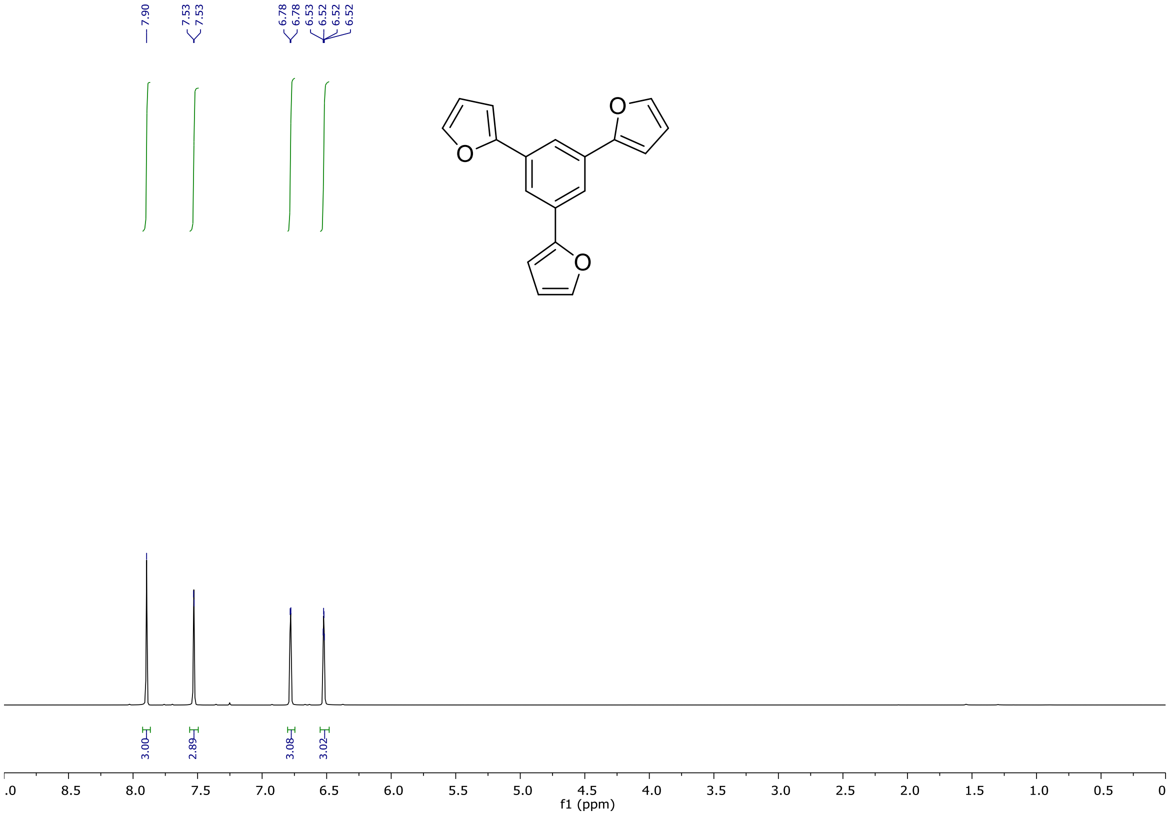

Supplementary Figure 30. ^{13}C-NMR spectrum of **1t**

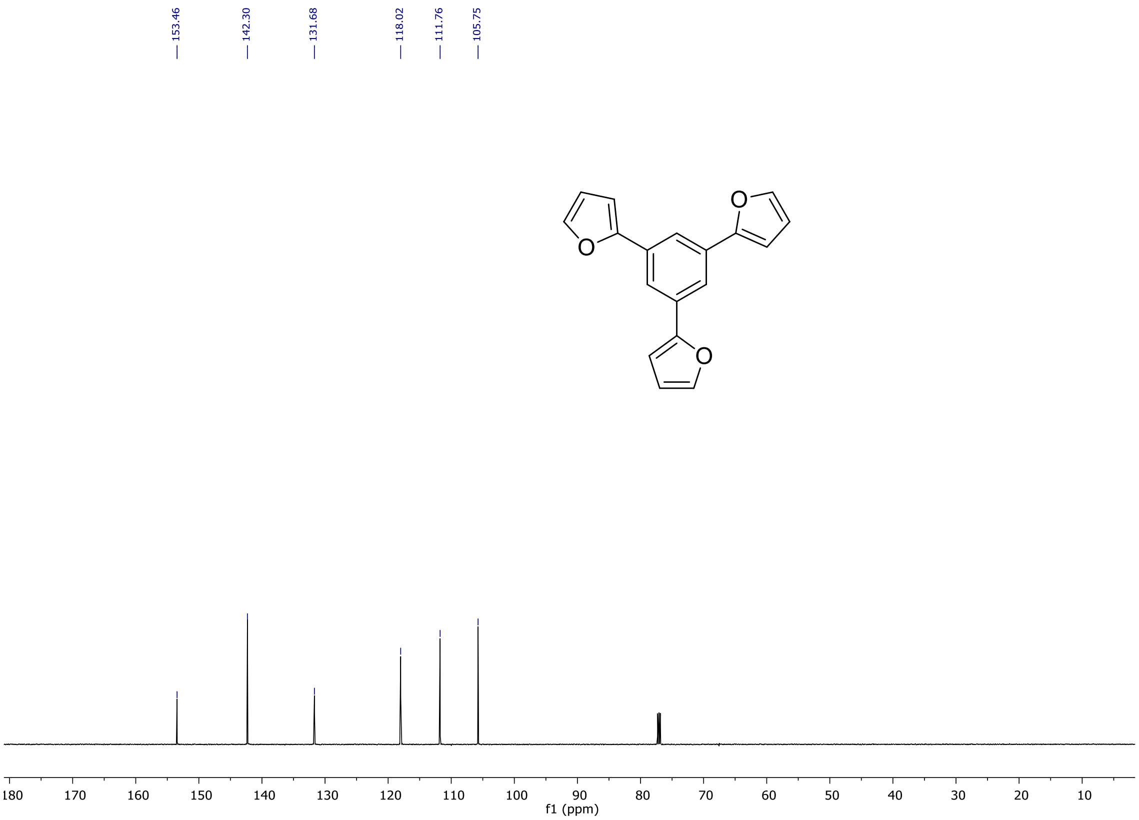

Supplementary Figure 31. ^{1}H-NMR spectrum (Starting material for *Z*-**6**)

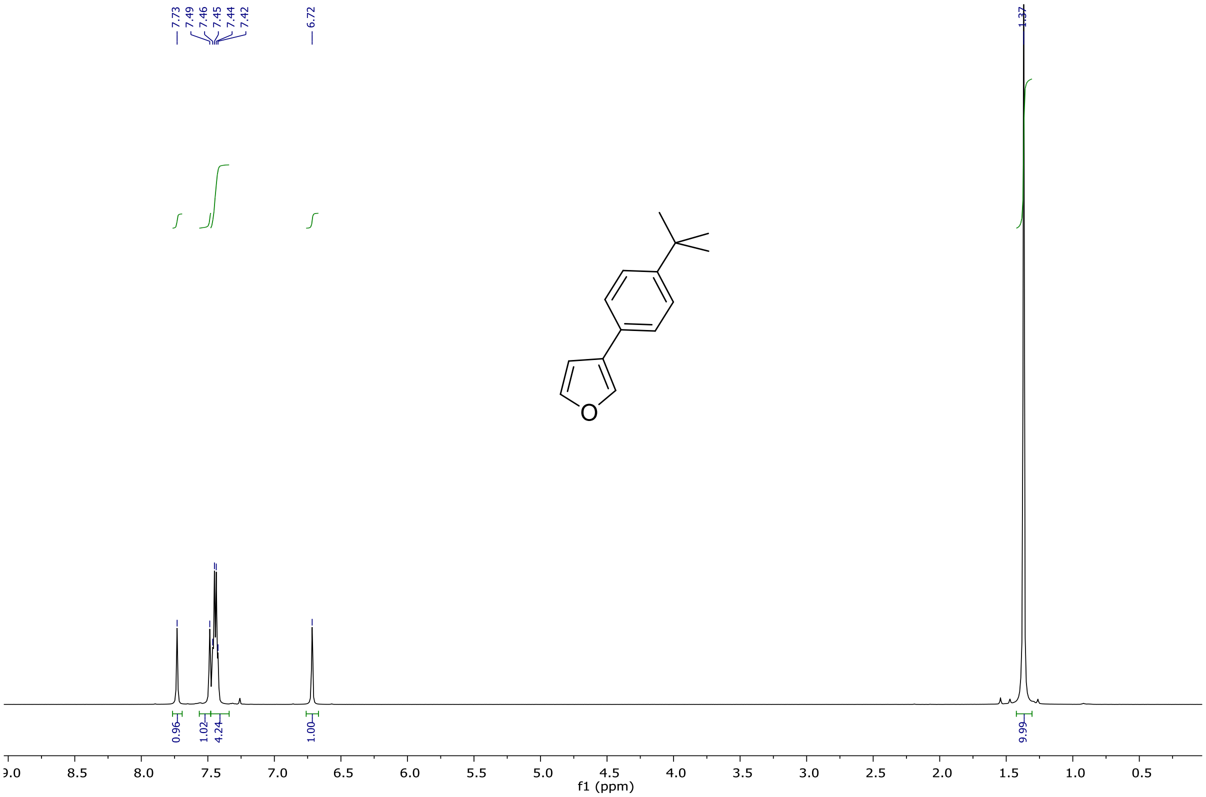

Supplementary Figure 32. ^{13}C-NMR spectrum (Starting material for *Z*-**6**)

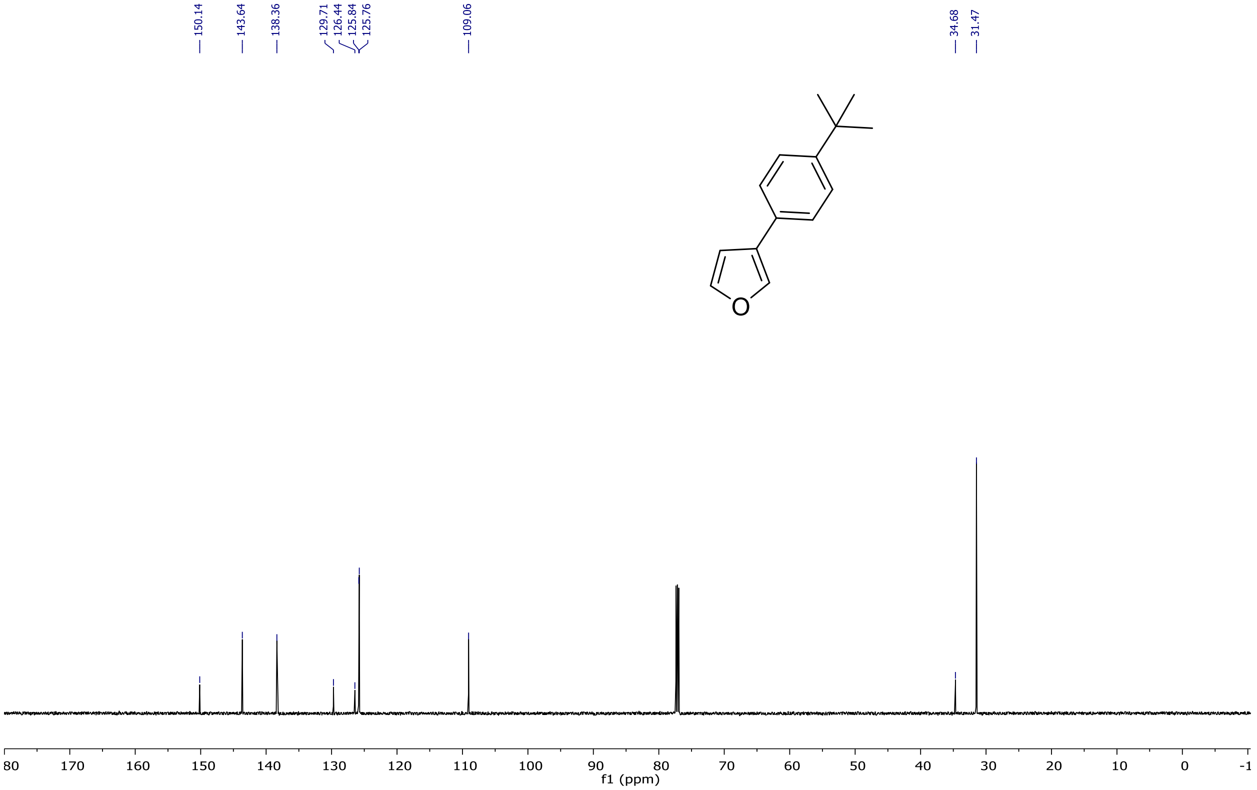

Supplementary Figure 33. ^{1}H-NMR spectrum (Starting material for **11**)

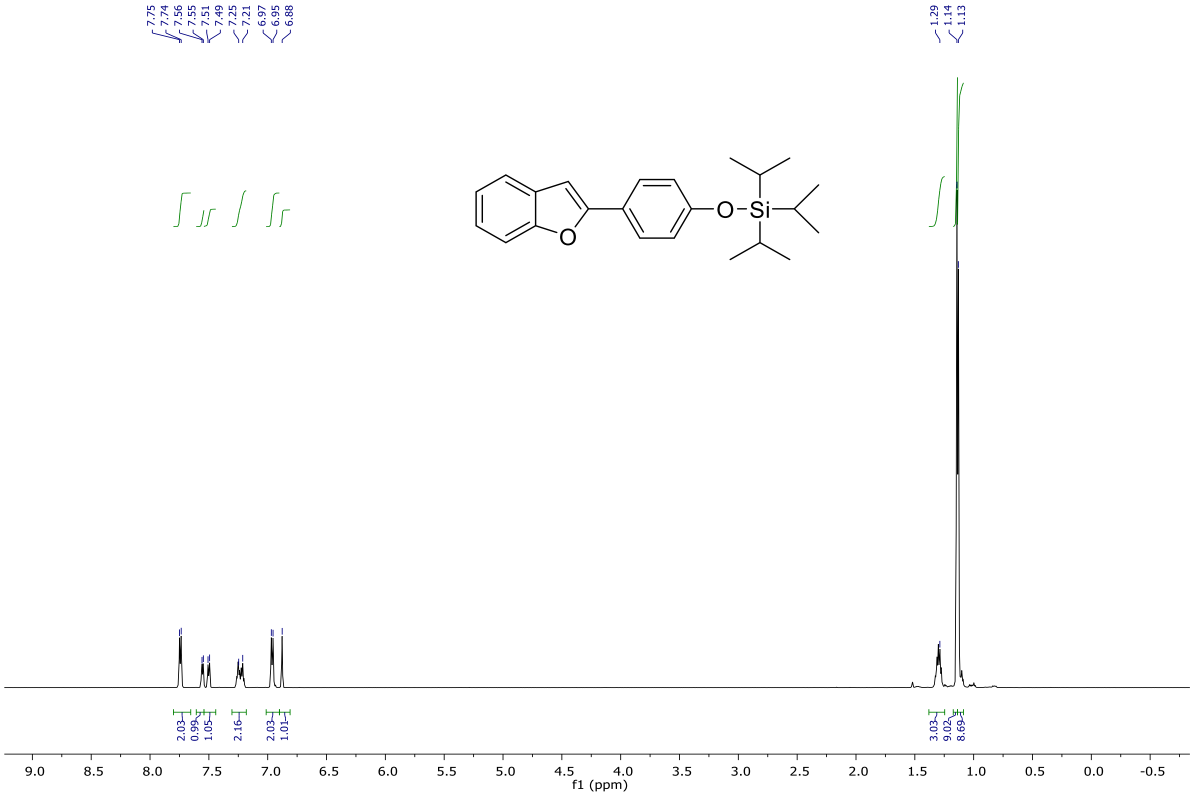

Supplementary Figure 34. ^{13}C and ^{29}Si-NMR spectra (Starting material for **11**)

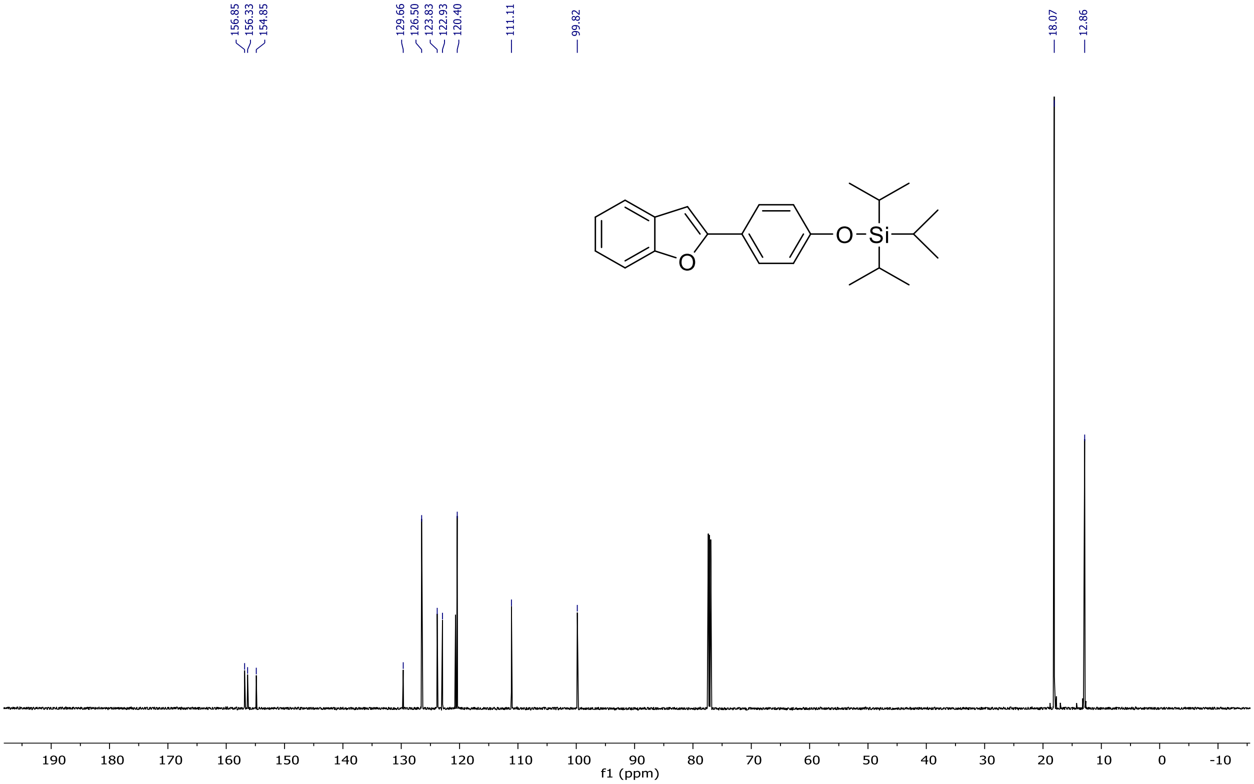

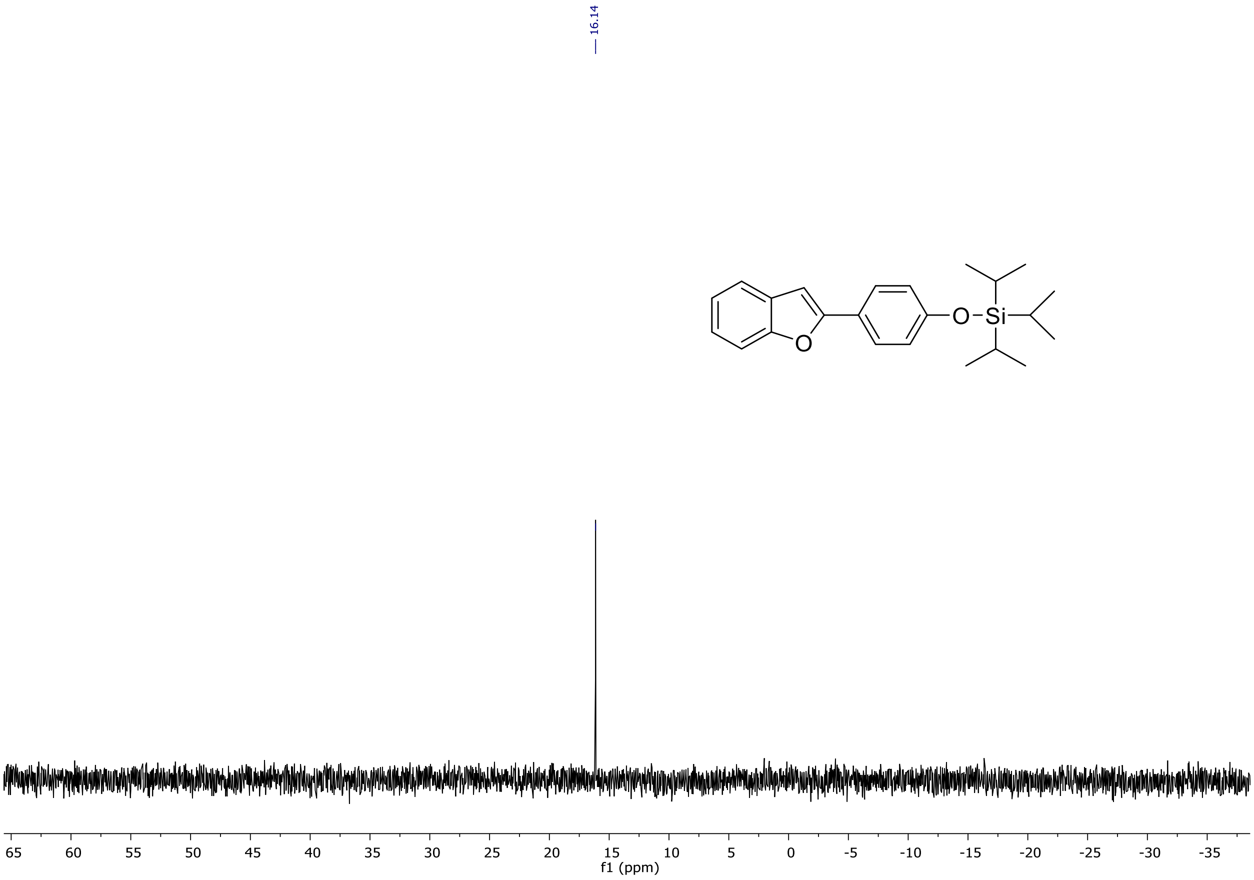

Supplementary Figure 35. ^{1}H and ^{13}C-NMR spectra (Starting material for **12**)

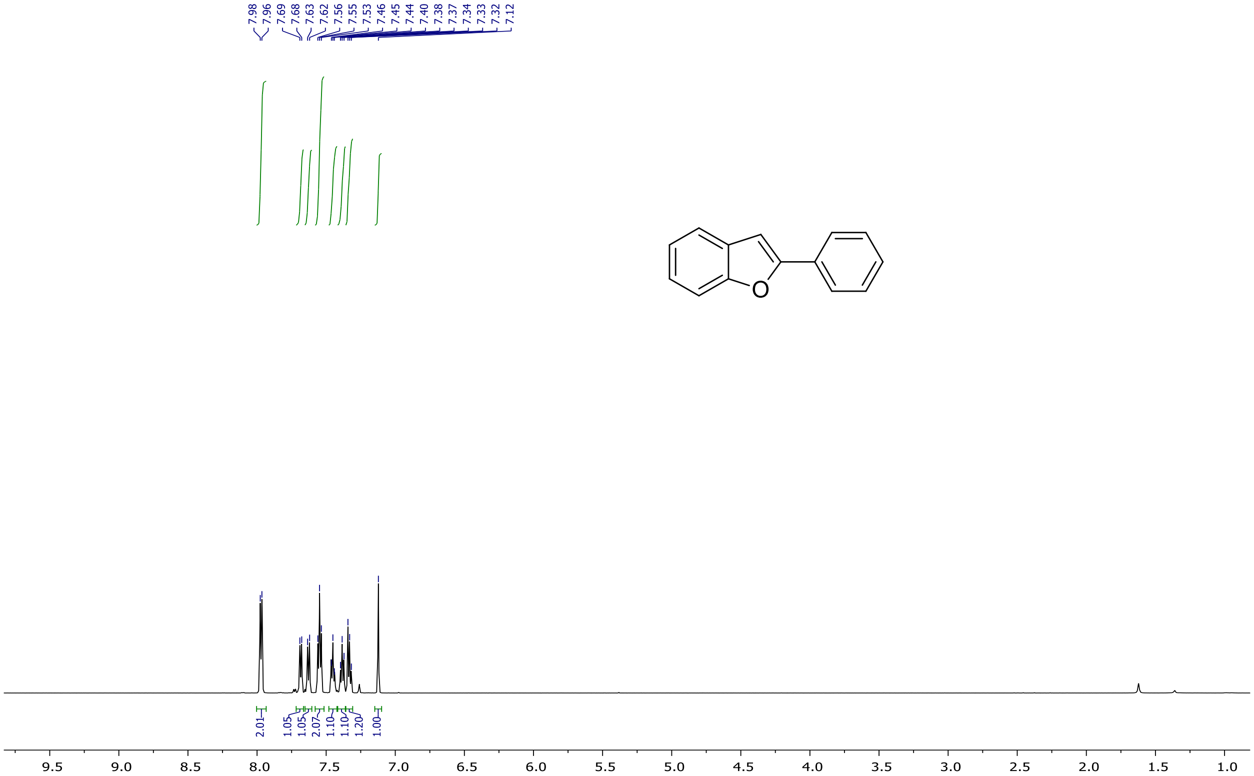

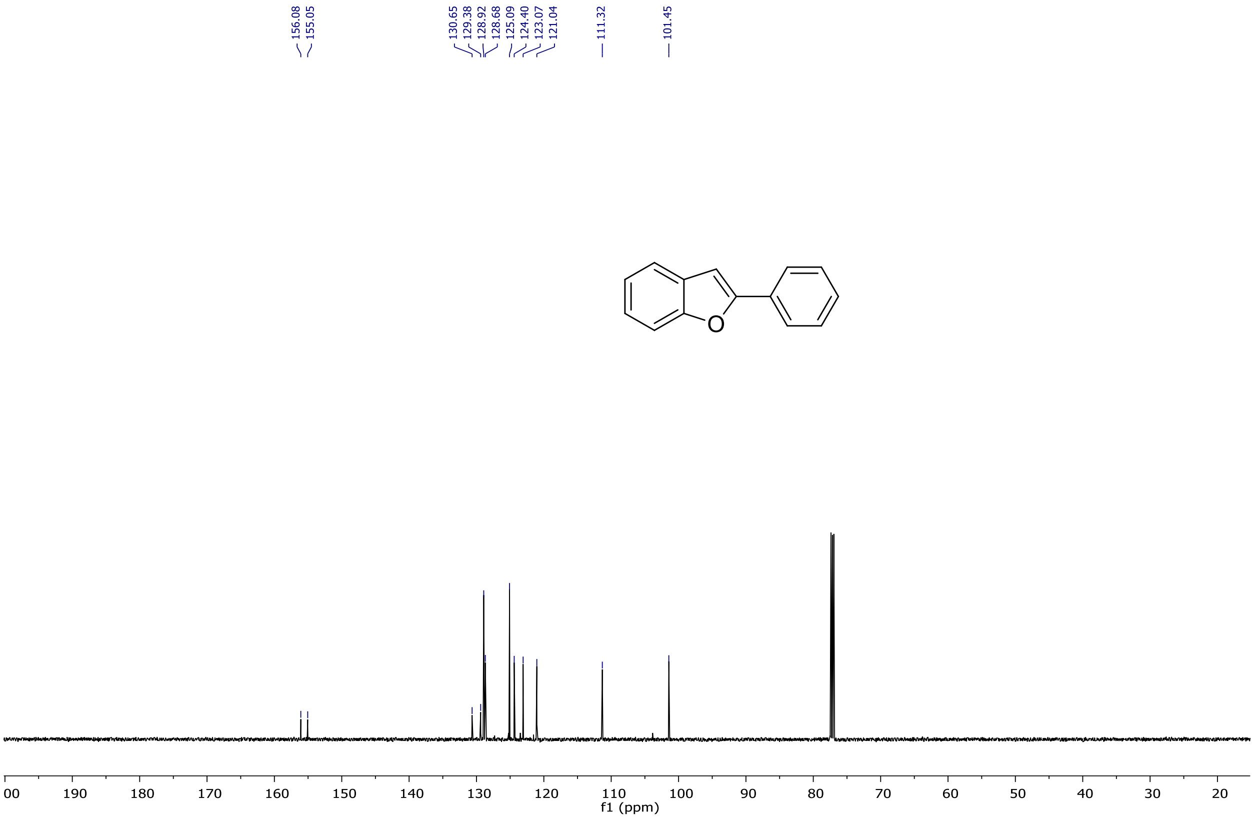

Supplementary Figure 36. ^{1}H and ^{13}C-NMR spectra of **1ac**

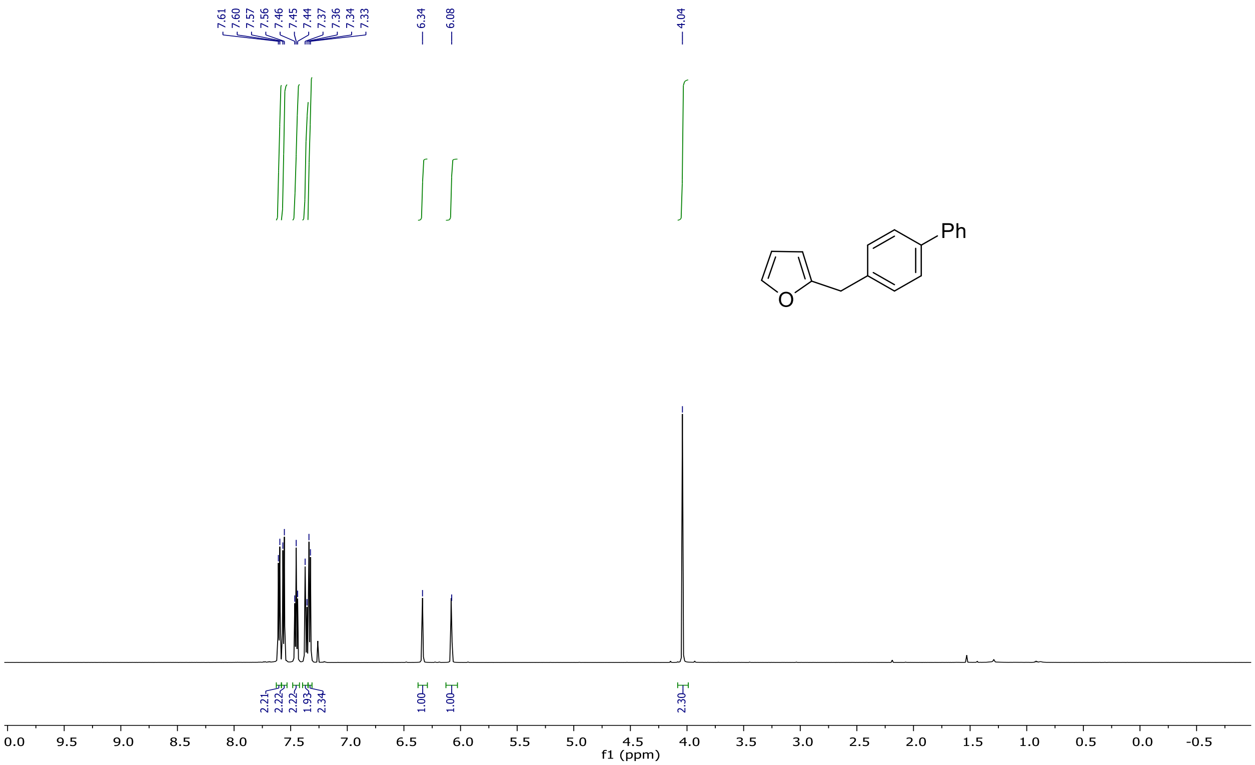

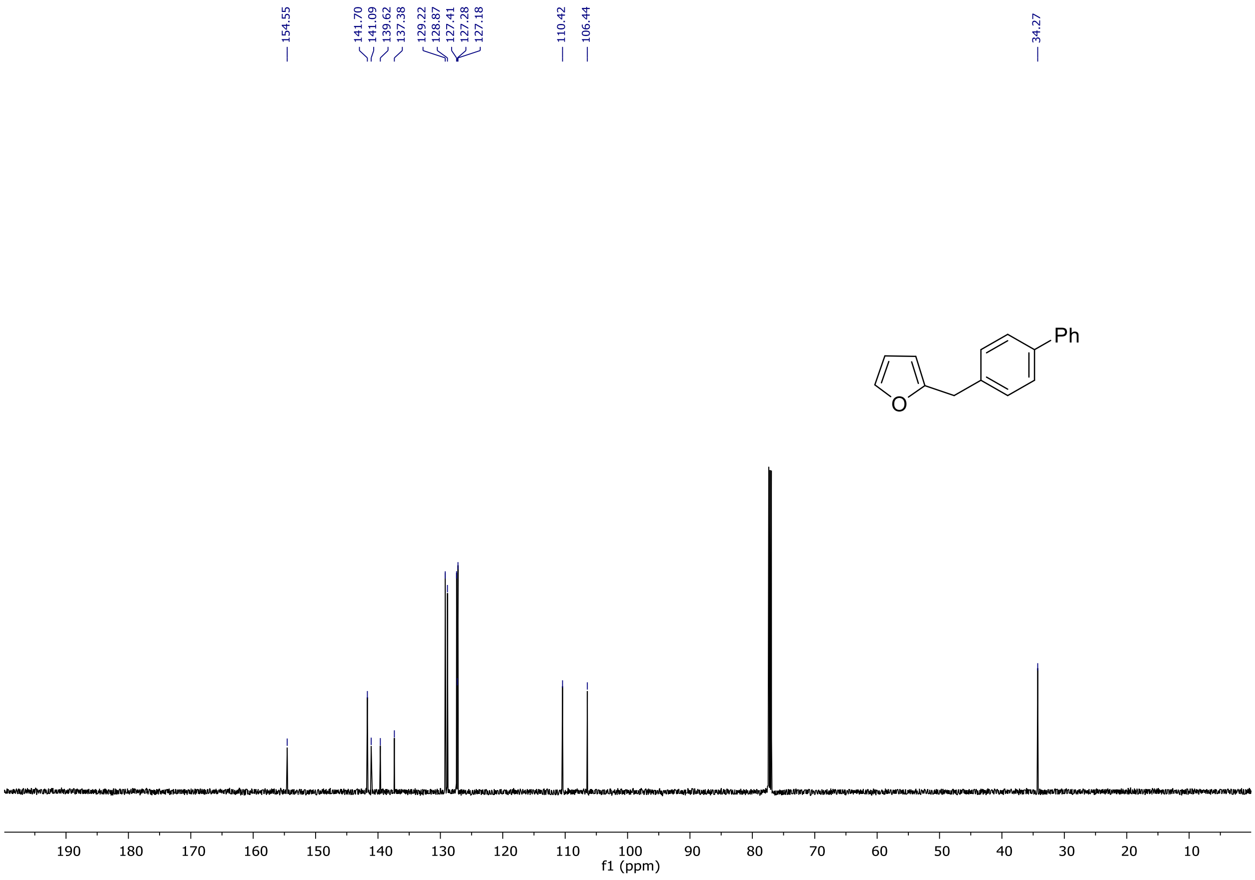

Supplementary Figure 37. ^{1}H and ^{13}C-NMR spectra of Dimethylphenylsilane-*d*

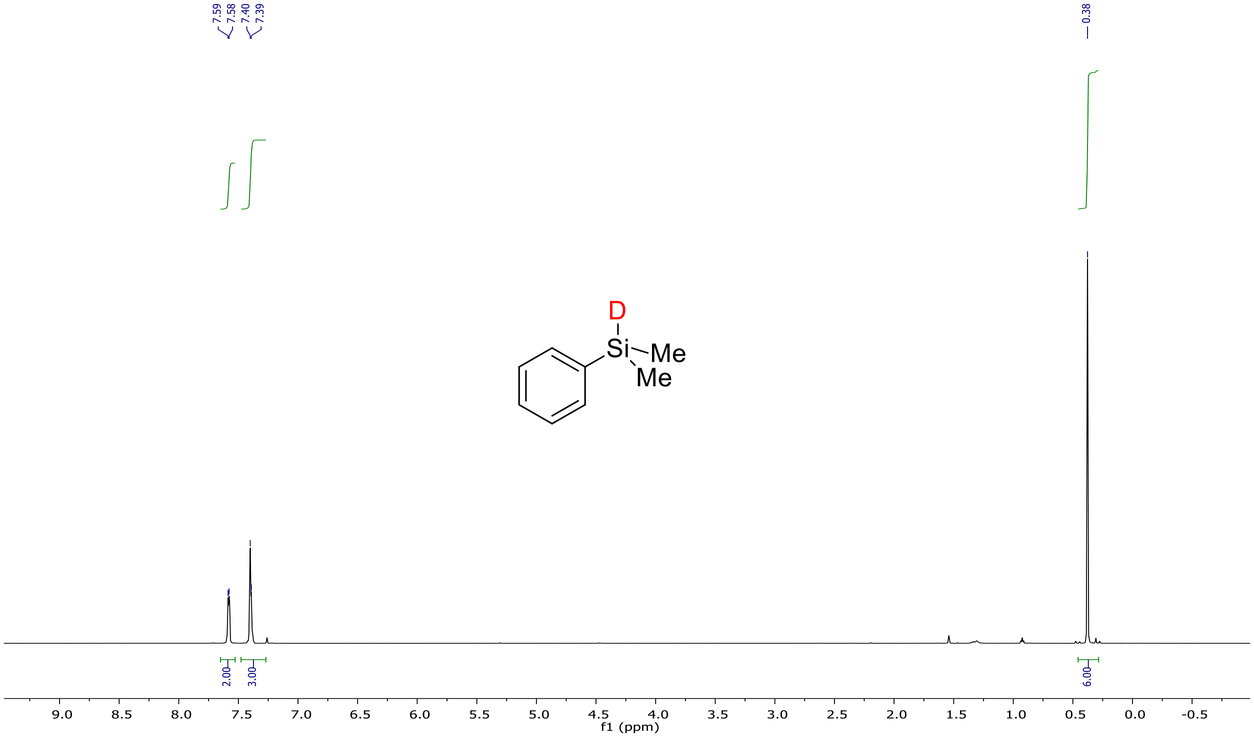

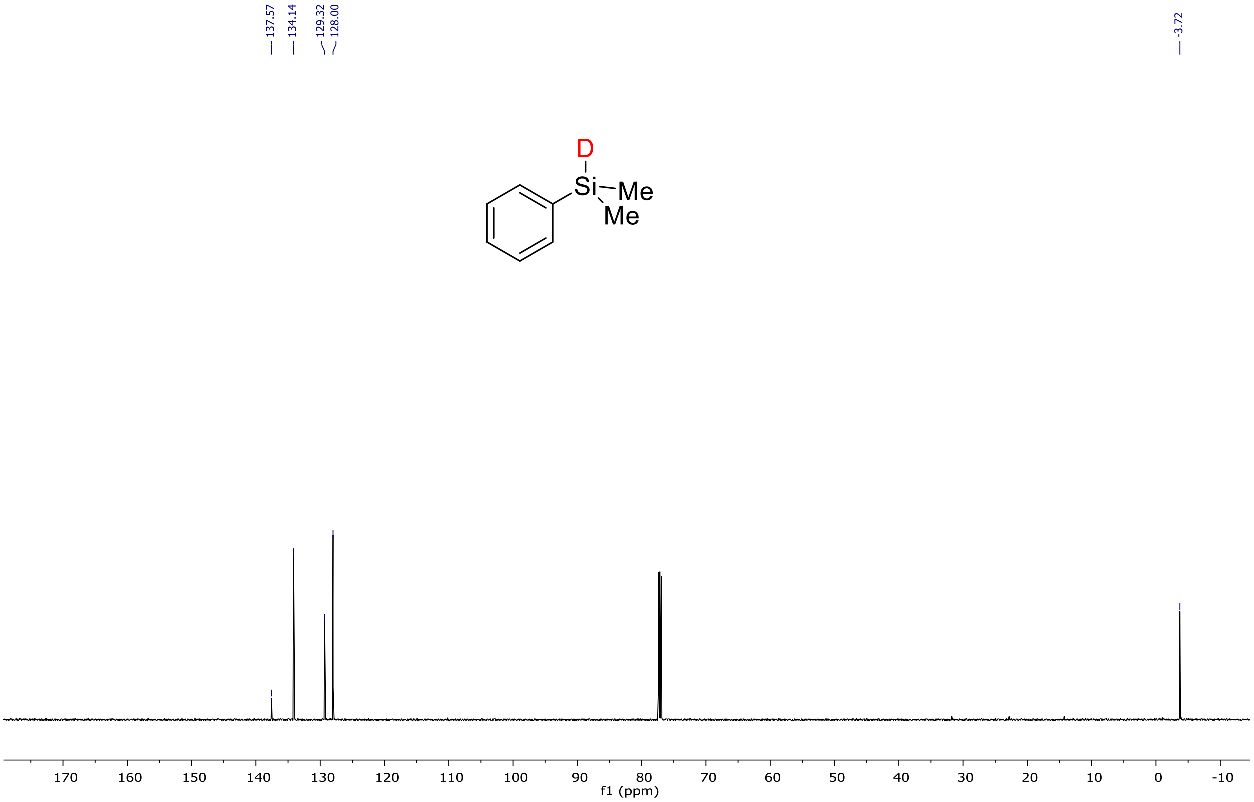

Supplementary Figure 38. ^{29}Si-NMR spectrum of Dimethylphenylsilane-*d*

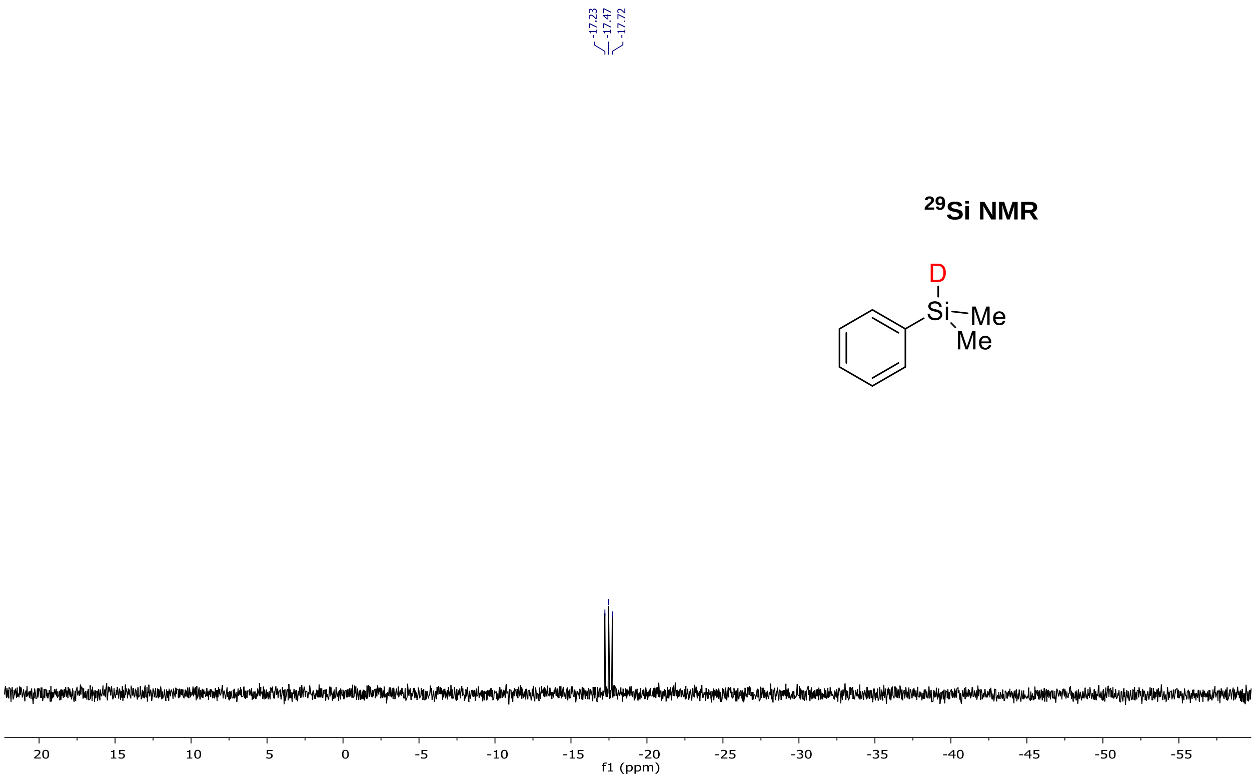

Supplementary Figure 39. ^{1}H and ^{13}C-NMR spectra of *Z*-**2a**

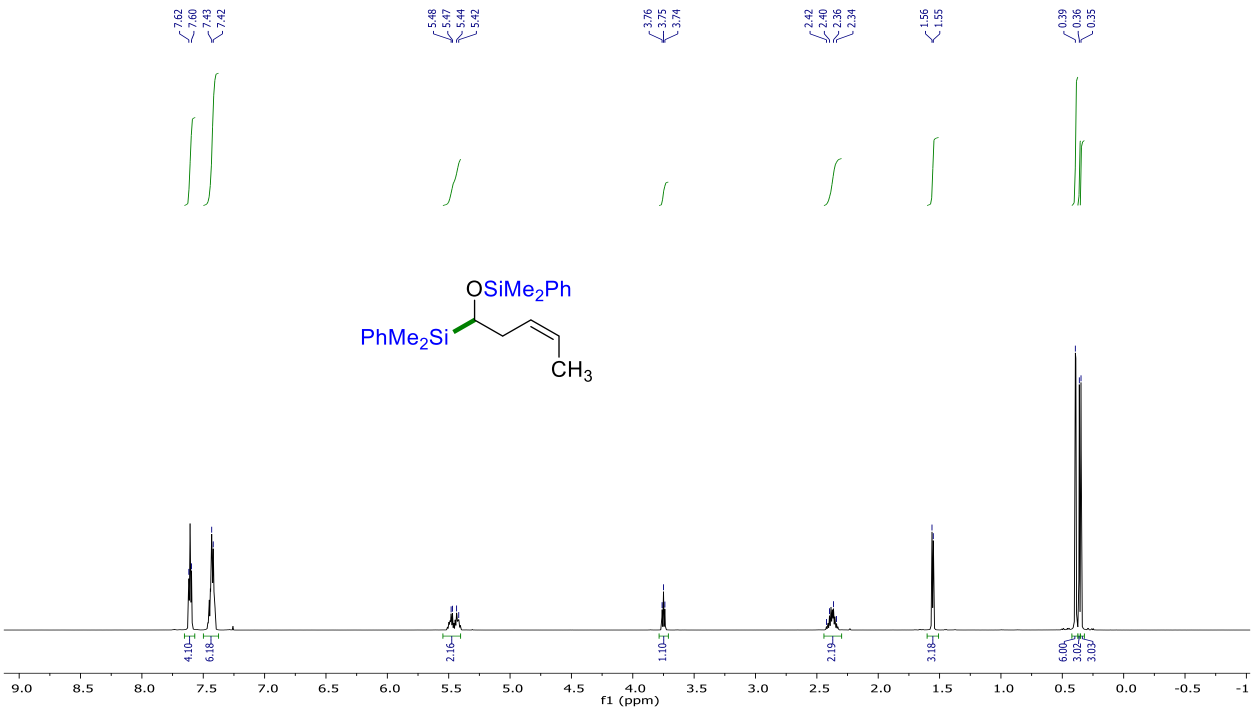

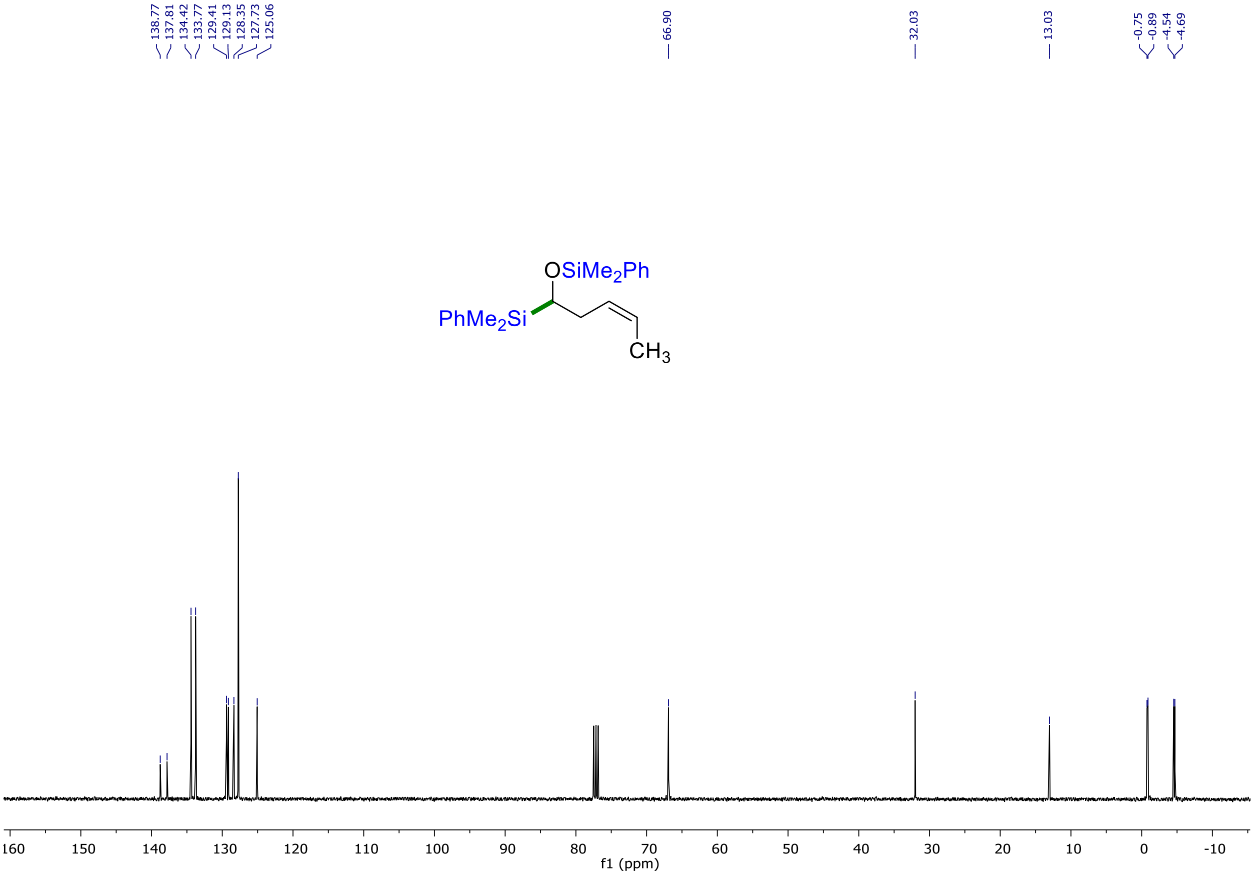

Supplementary Figure 40. ^{29}Si-NMR spectrum of *Z*-**2a**

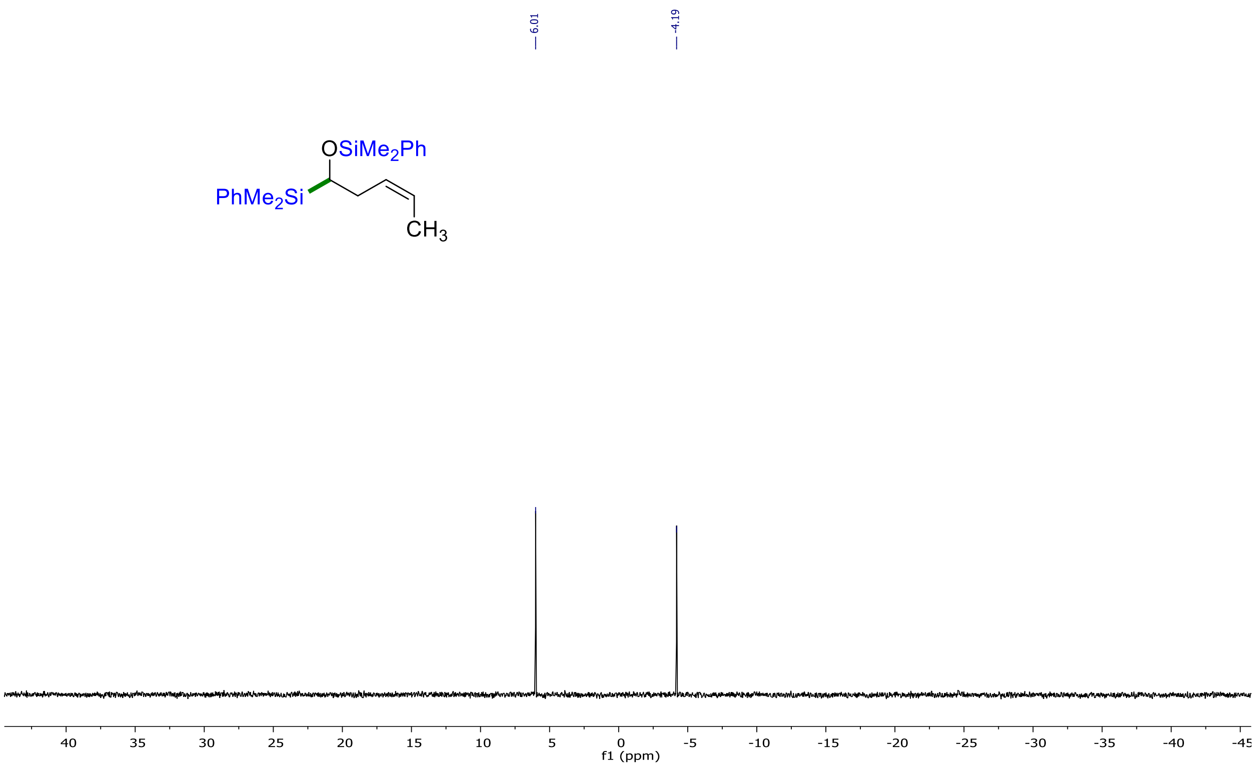

Supplementary Figure 41. ^{1}H-NMR spectrum of *Z*-**2a**-***d*$_2$**

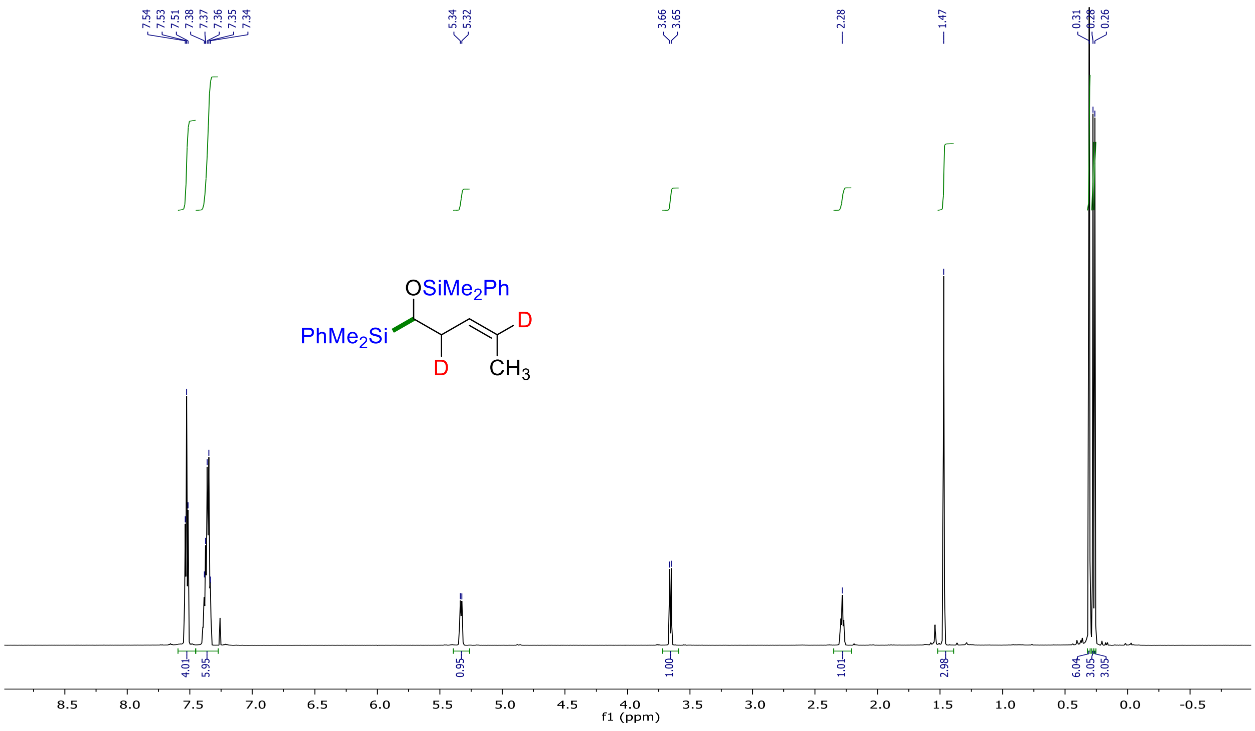

Supplementary Figure 42. ^{13}C and ^{29}Si-NMR spectra of *Z*-**2a**-***d***$_2$

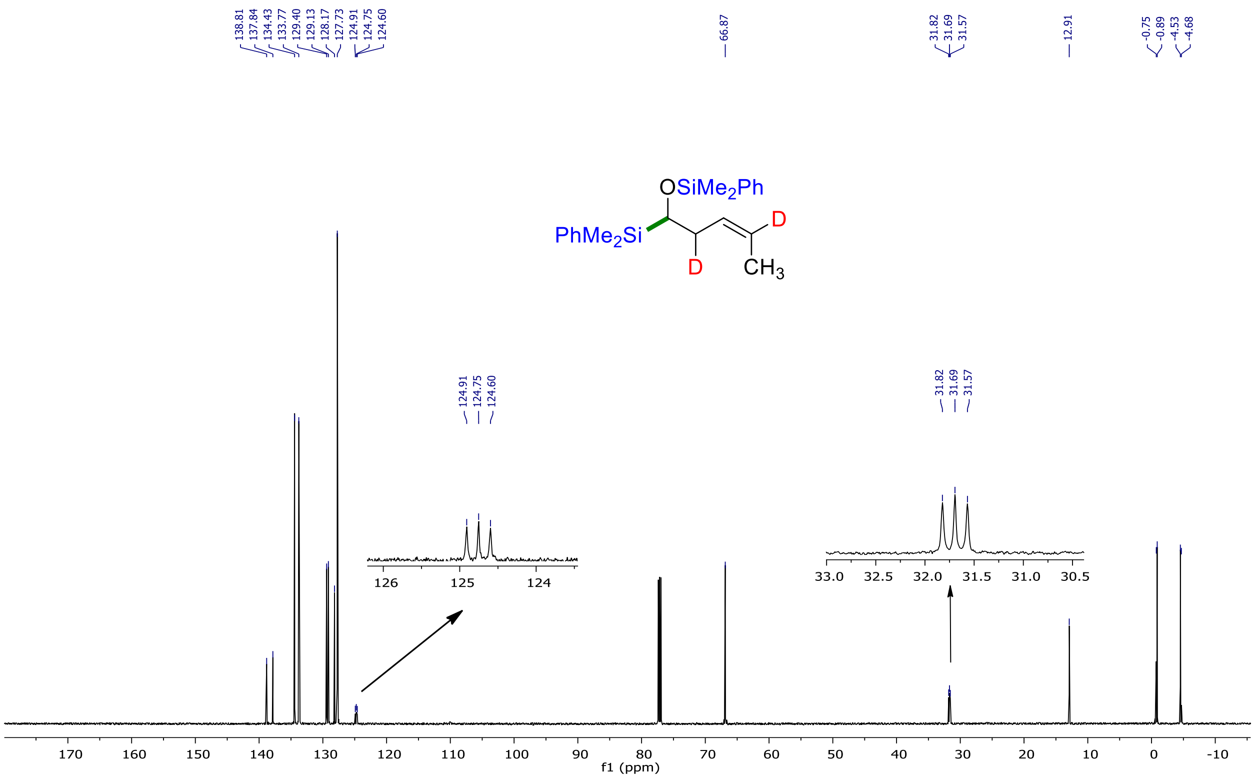

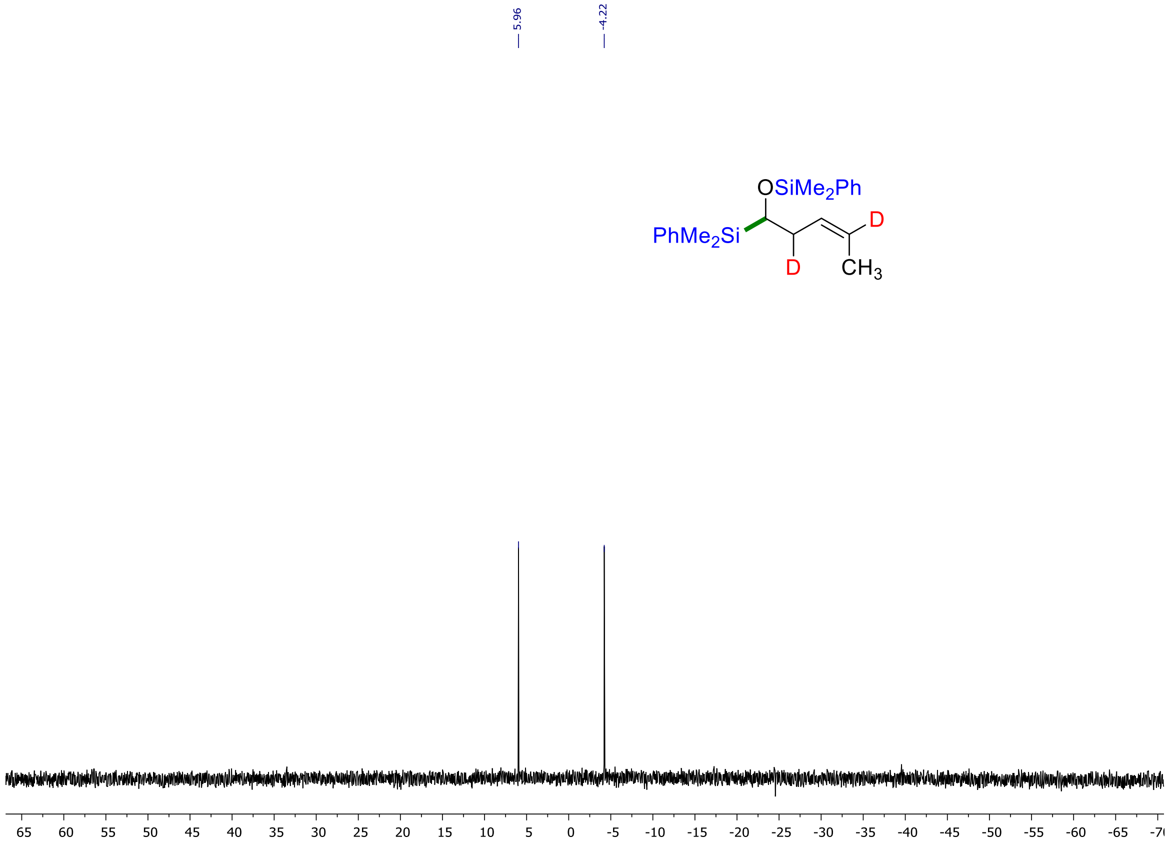

Supplementary Figure 43. ^{2}H-NMR spectrum of *Z*-**2a**-***d*$_2$**

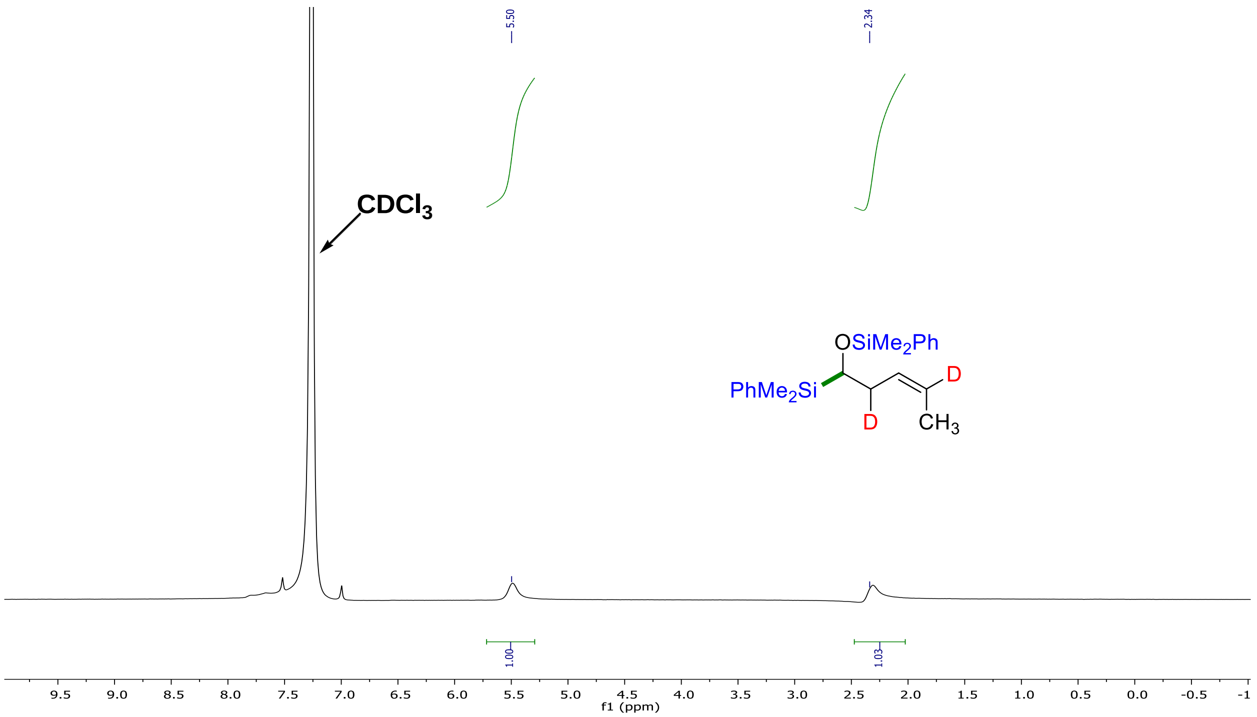

Supplementary Figure 44. ^{1}H-NMR spectrum of *Z*-**2a'**

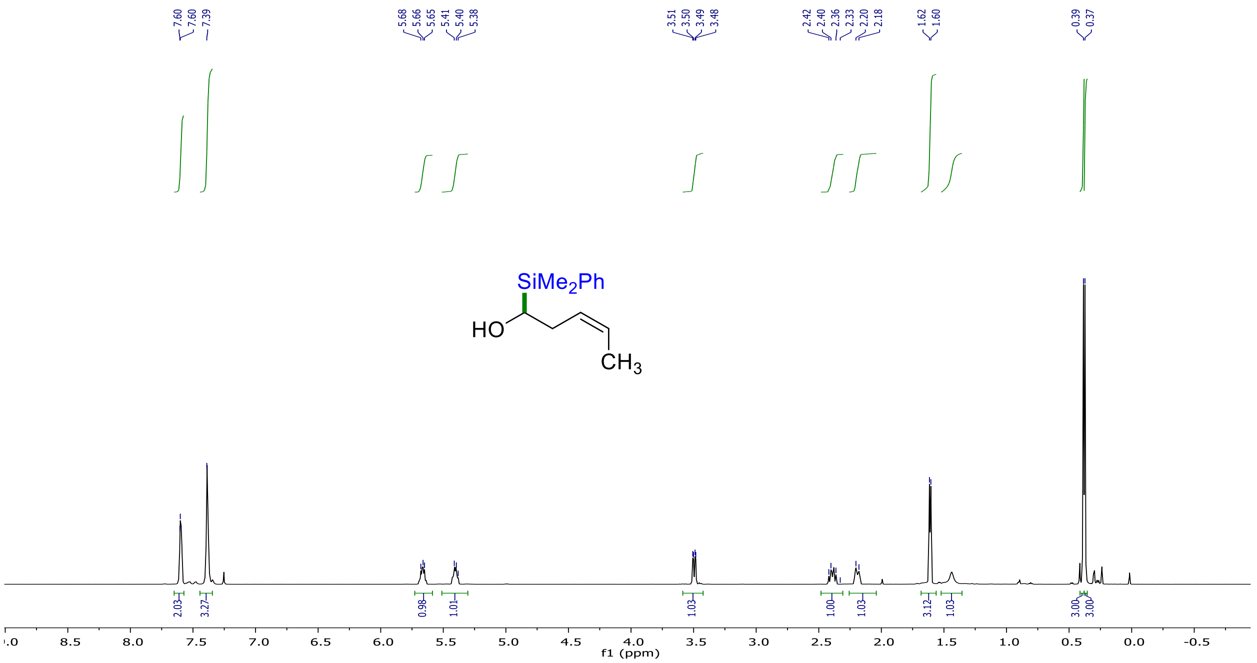

Supplementary Figure 45. ^{13}C and ^{29}Si-NMR spectra of *Z*-**2a'**

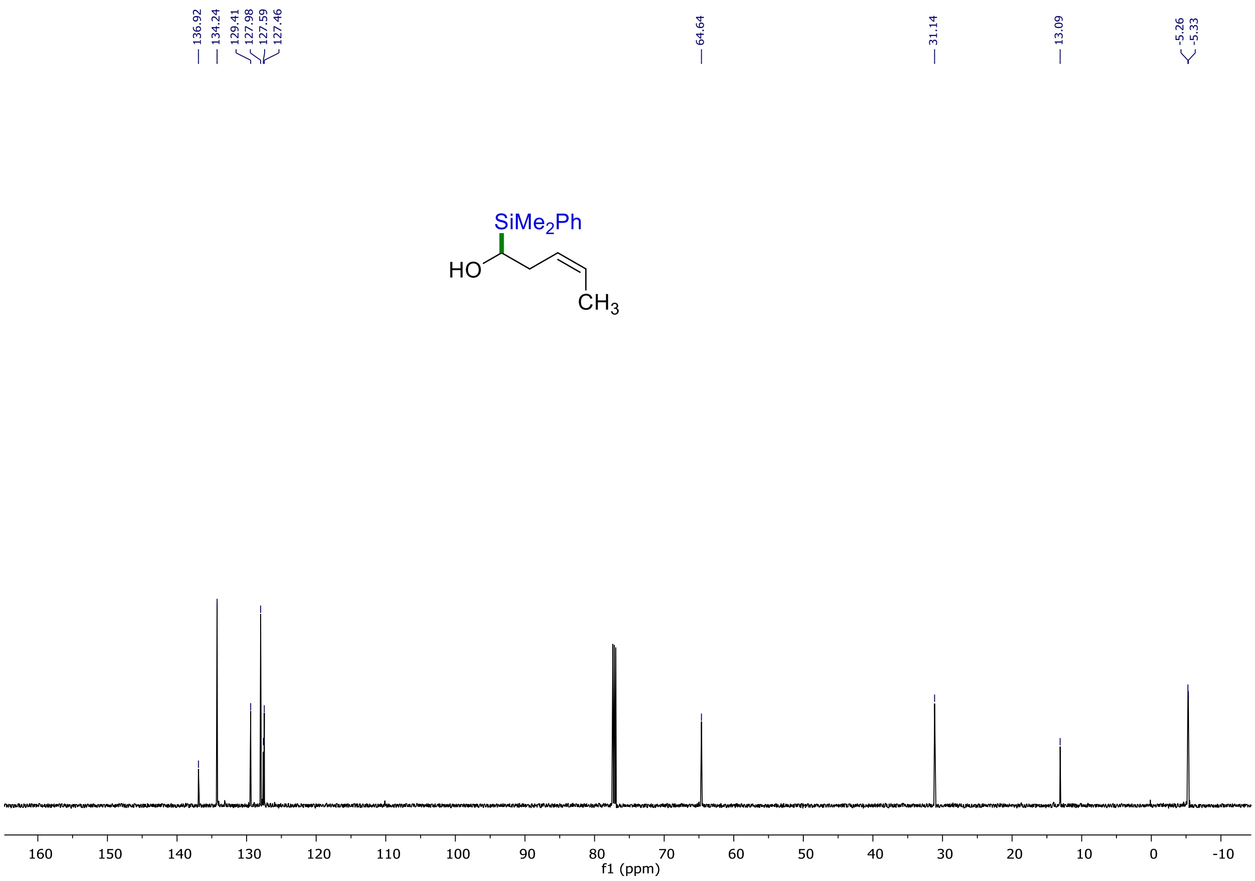

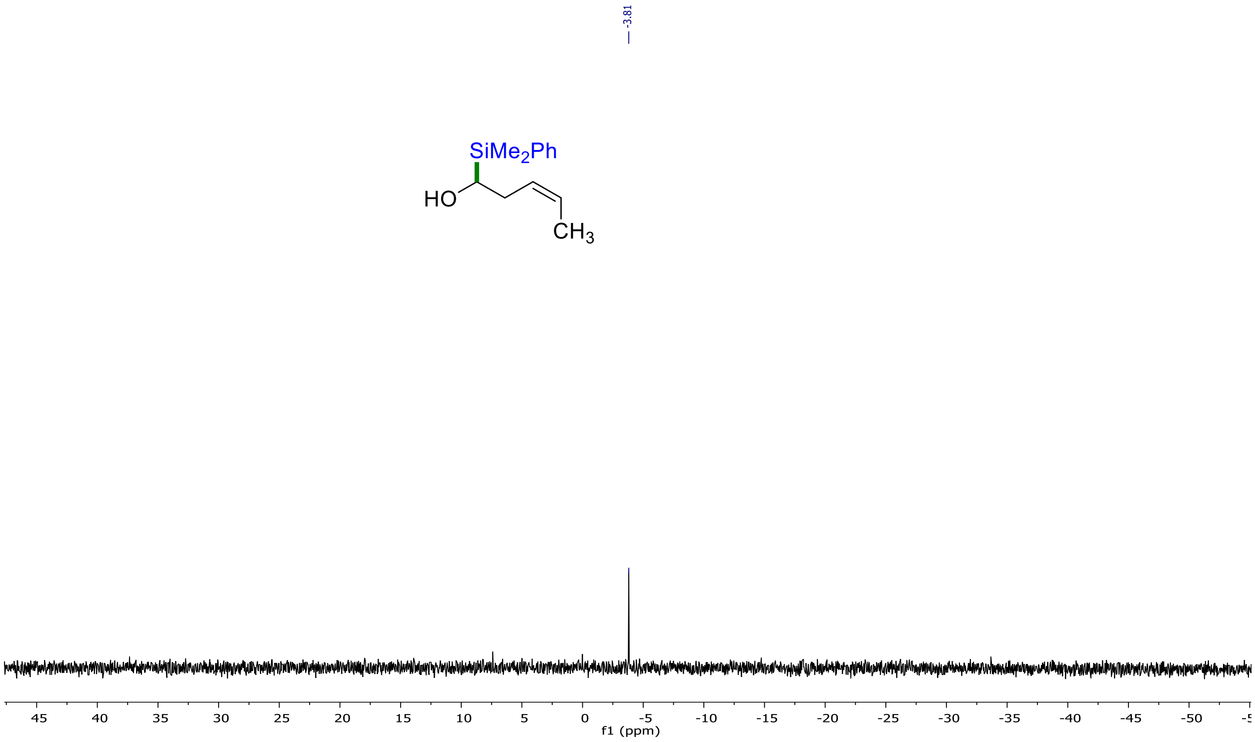

Supplementary Figure 46. ^{1}H and ^{13}C-NMR spectra of *Z*-**2a''**

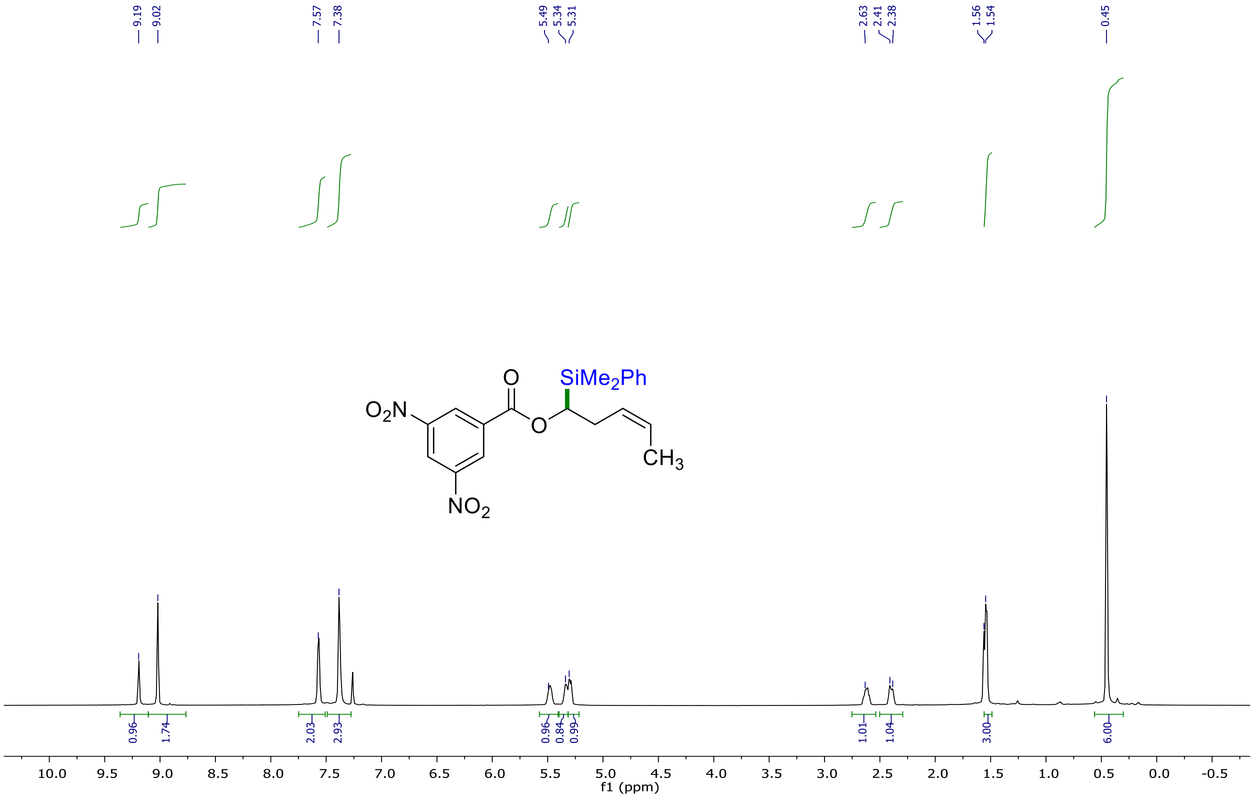

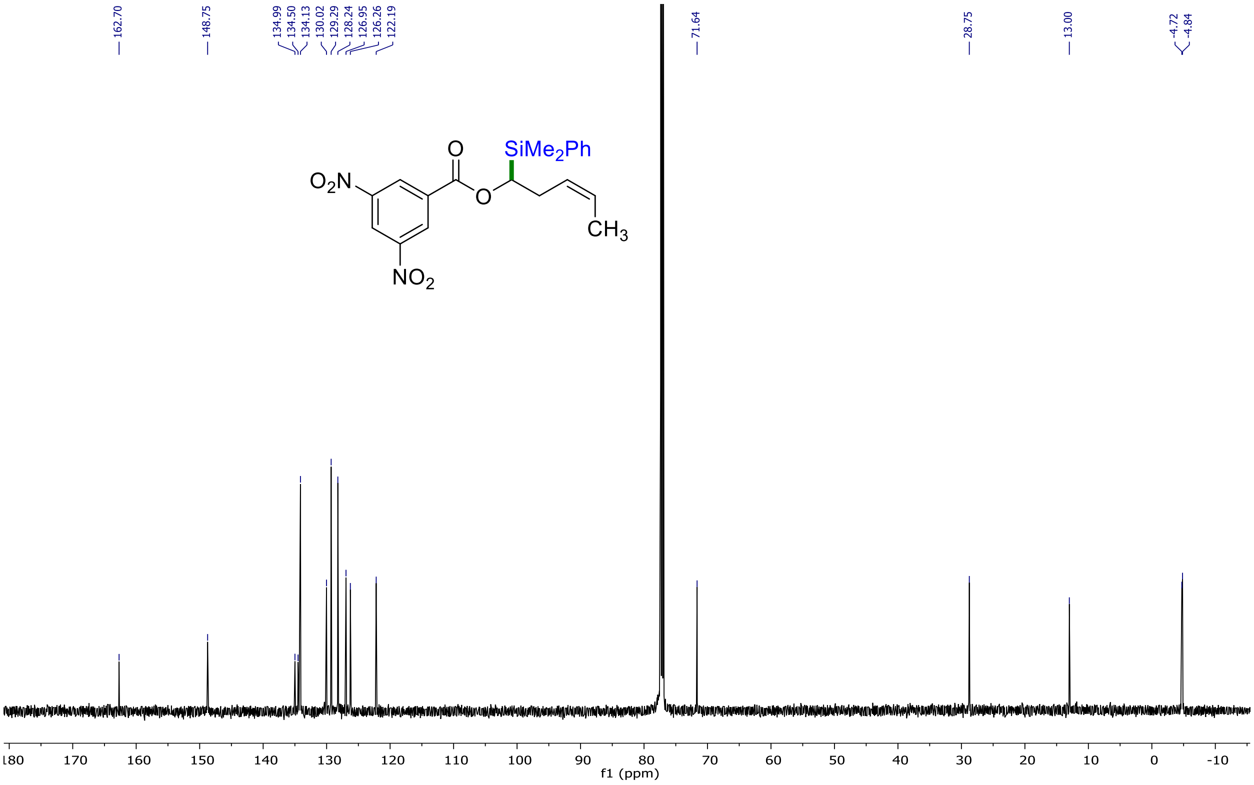

Supplementary Figure 47. ^{29}Si-NMR spectrum of *Z*-**2a''**

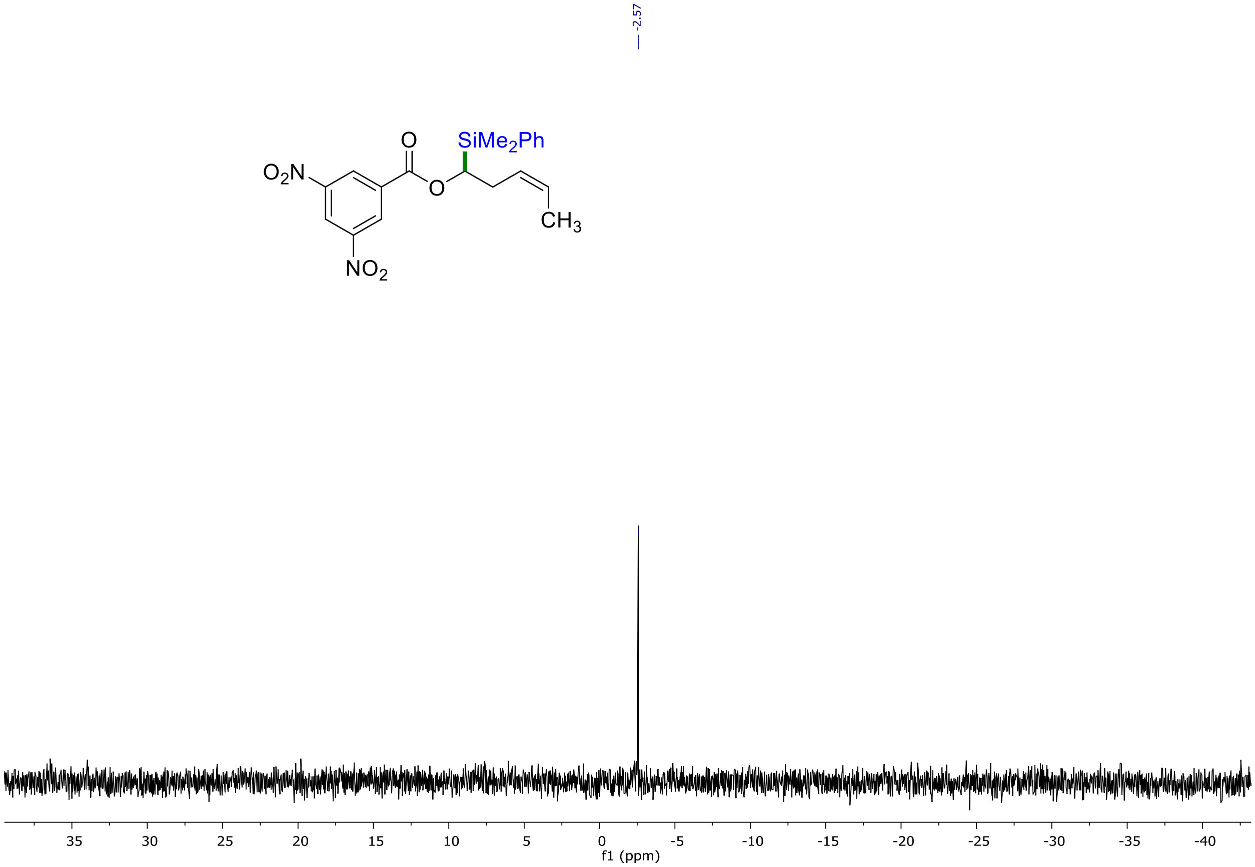

Supplementary Figure 48. ^{1}H-NMR spectrum of *Z*-**2b**

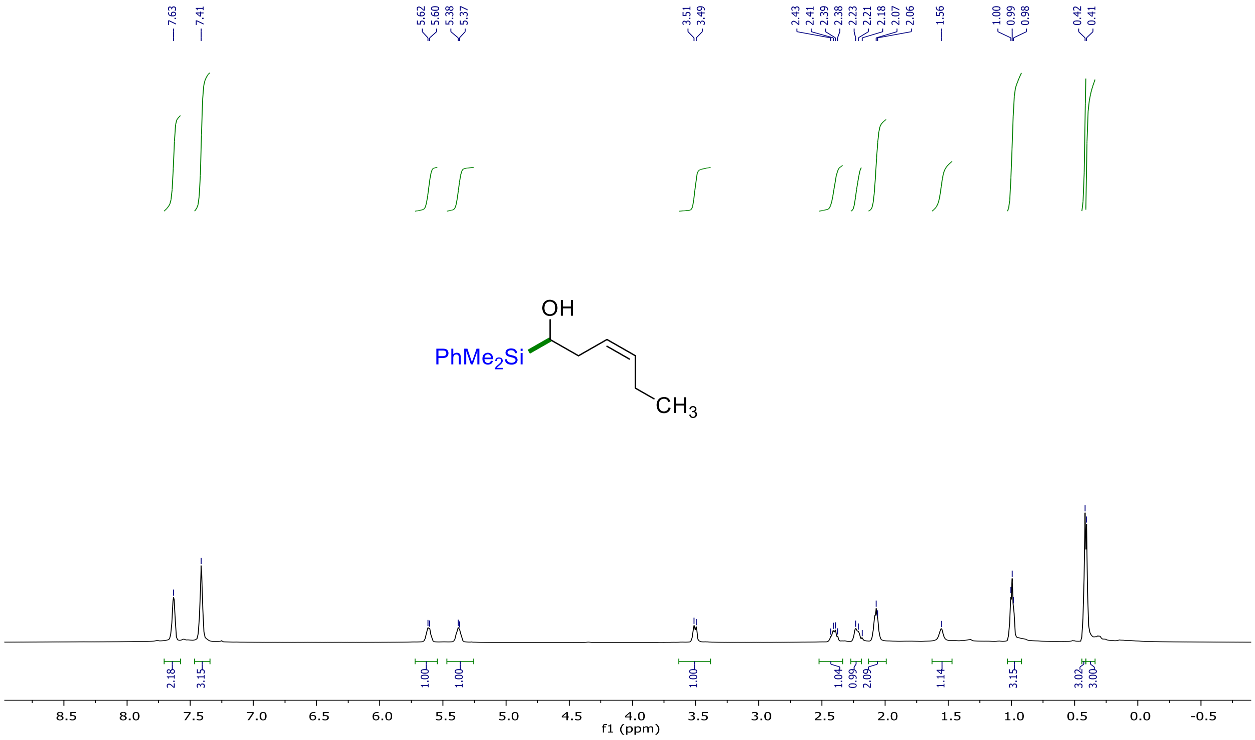

Supplementary Figure 49. ^{13}C and ^{29}Si-NMR spectra of *Z*-**2b**

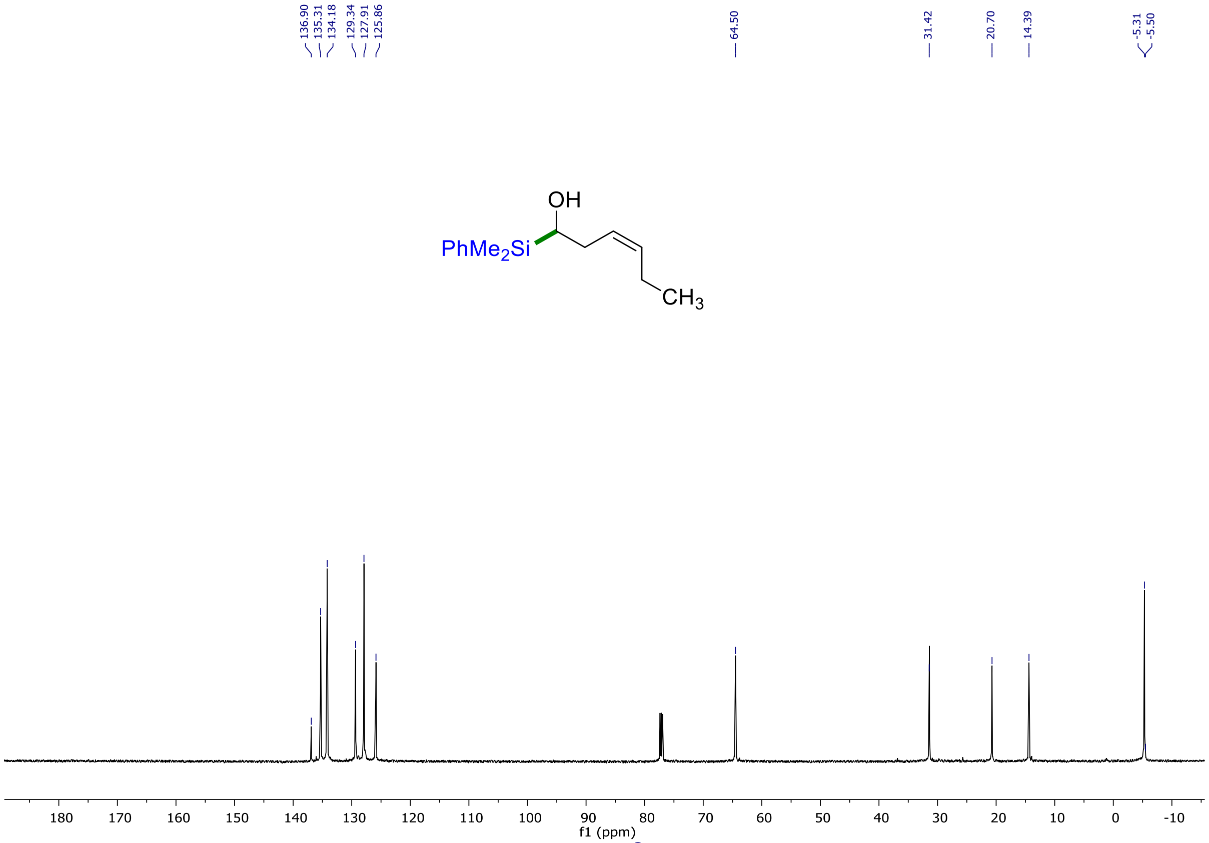

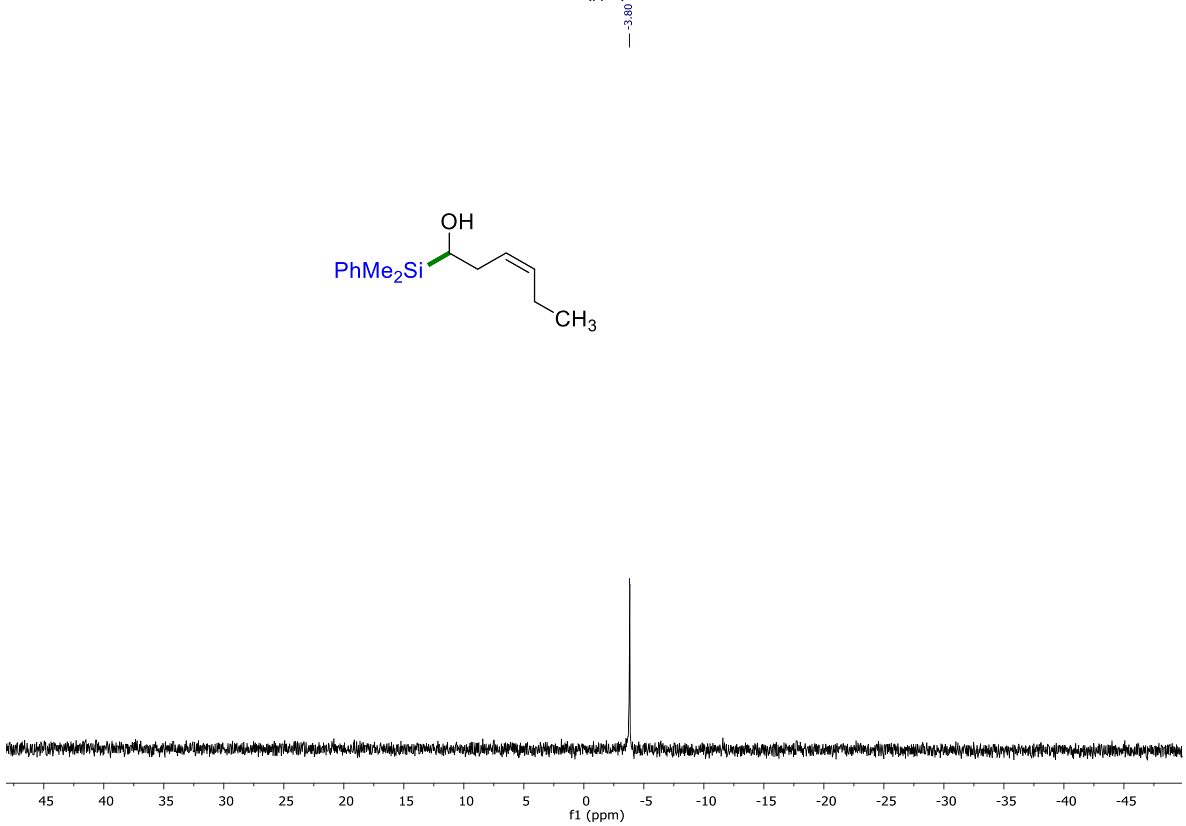

Supplementary Figure 50. ^{1}H and ^{13}C-NMR spectra of *Z*-**2c**

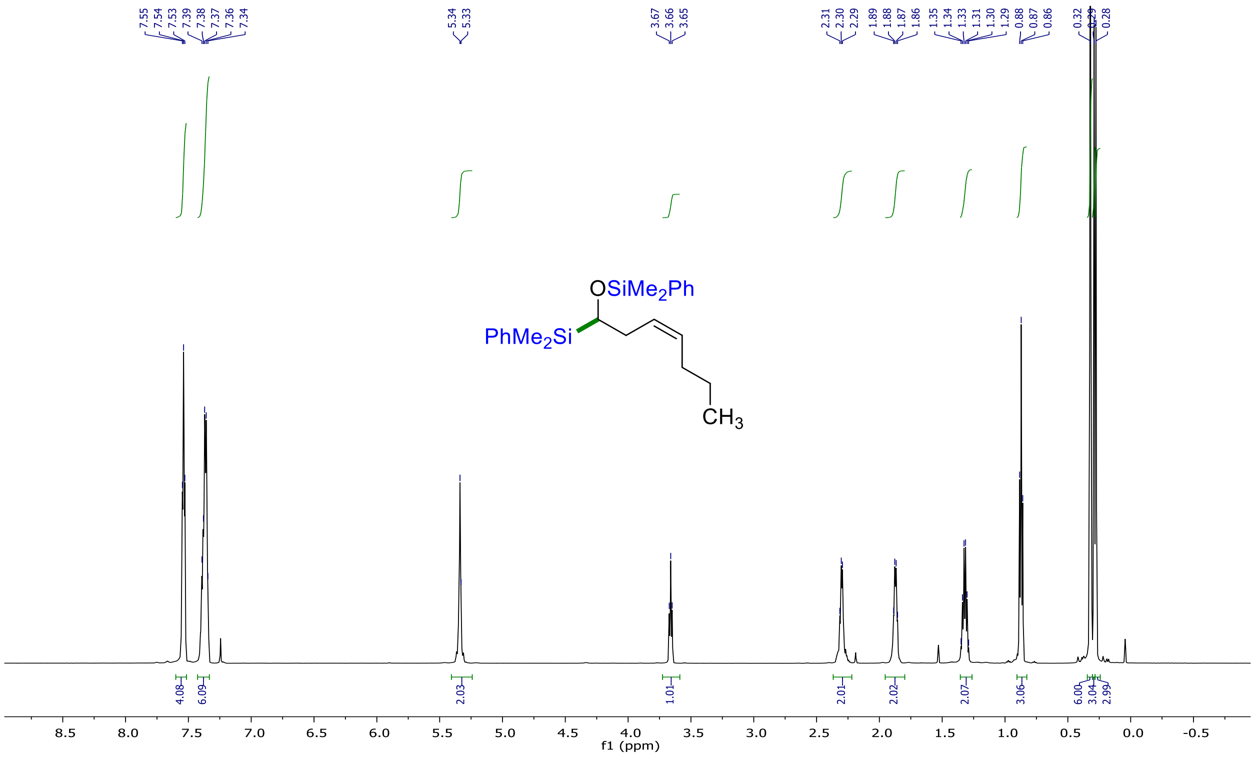

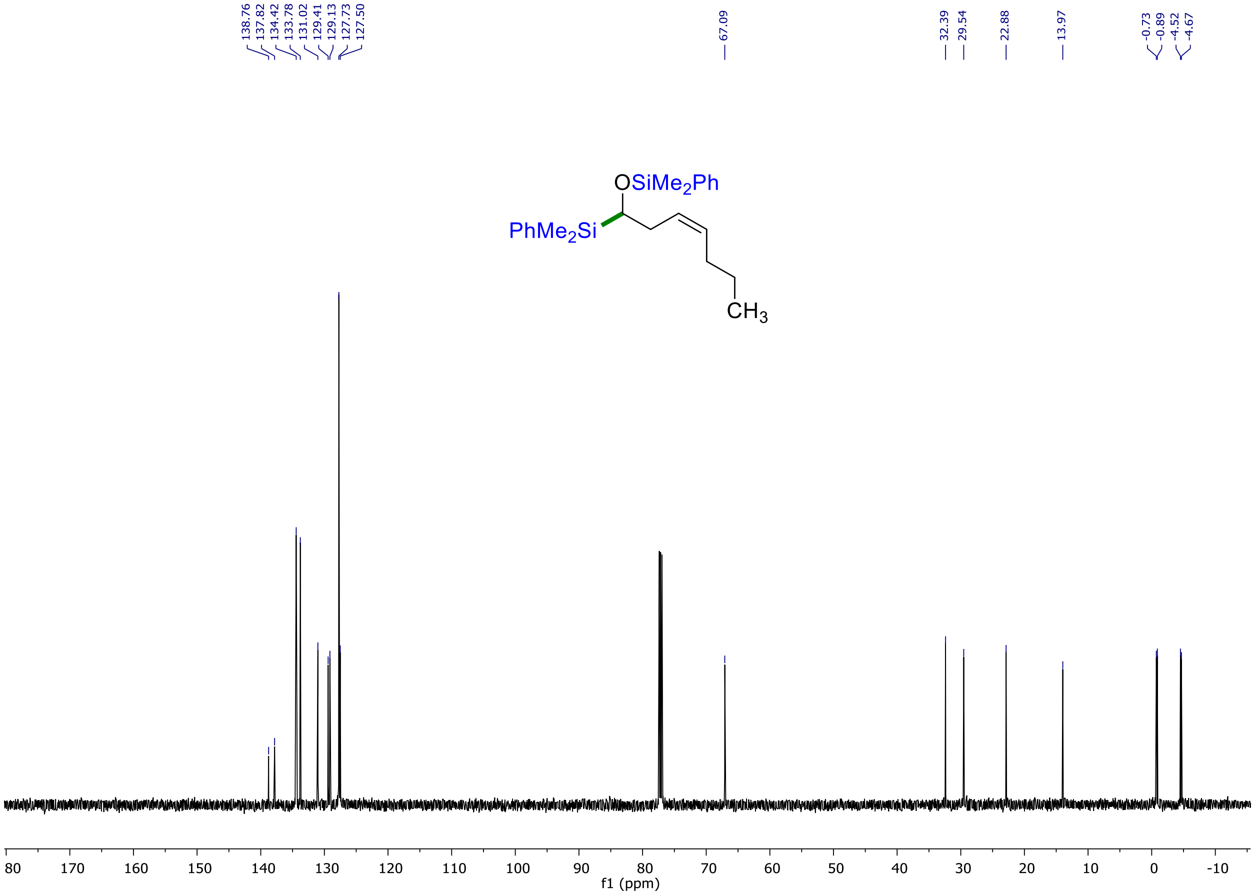

Supplementary Figure 51. ^{29}Si-NMR spectrum of *Z*-**2c**

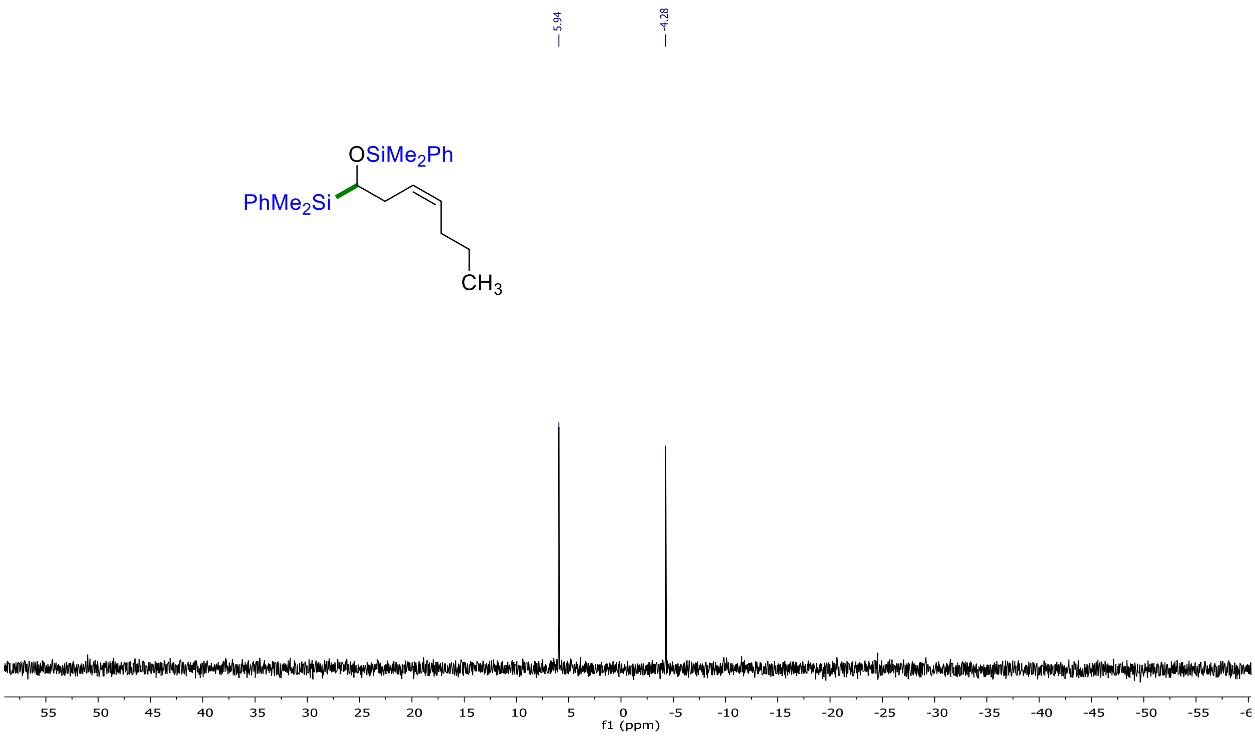

Supplementary Figure 52. ^{1}H-NMR spectrum of *Z*-**2d**

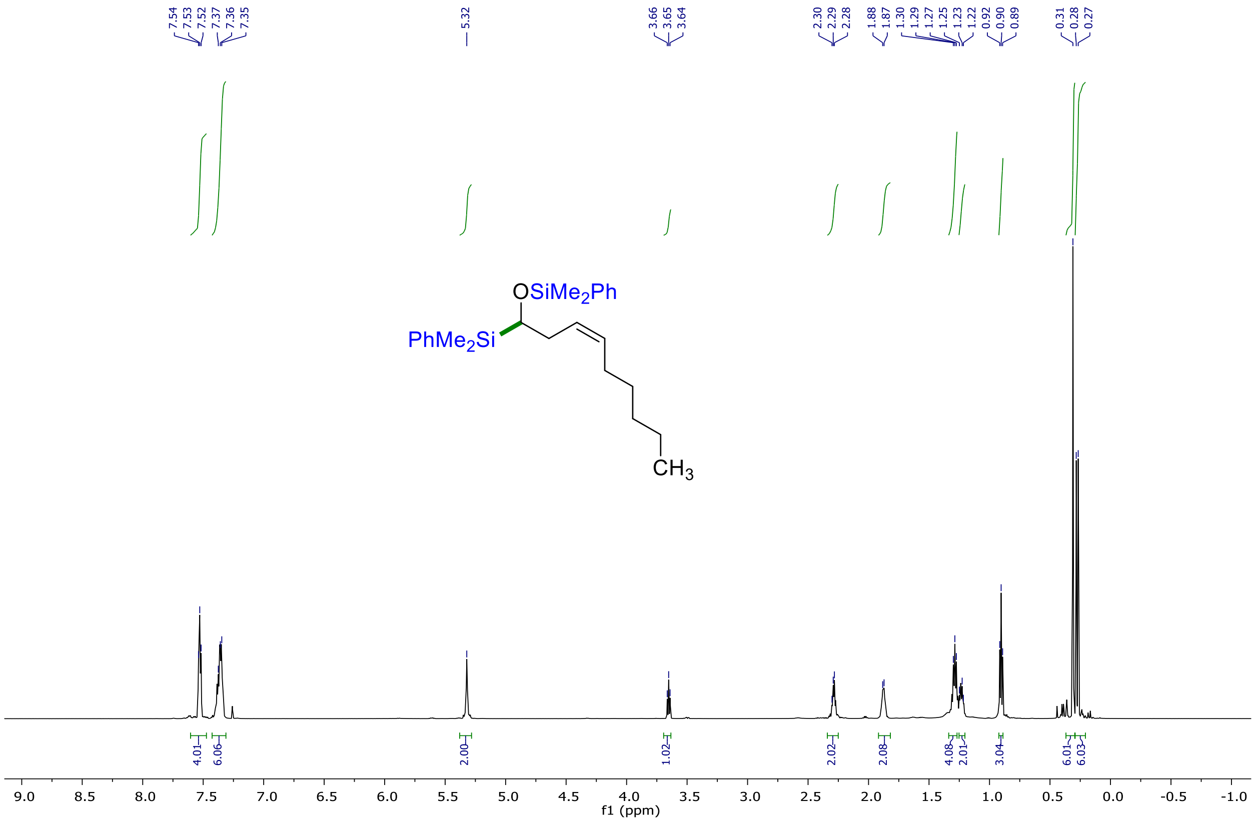

Supplementary Figure 53. ^{13}C and ^{29}Si-NMR spectra of *Z*-**2d**

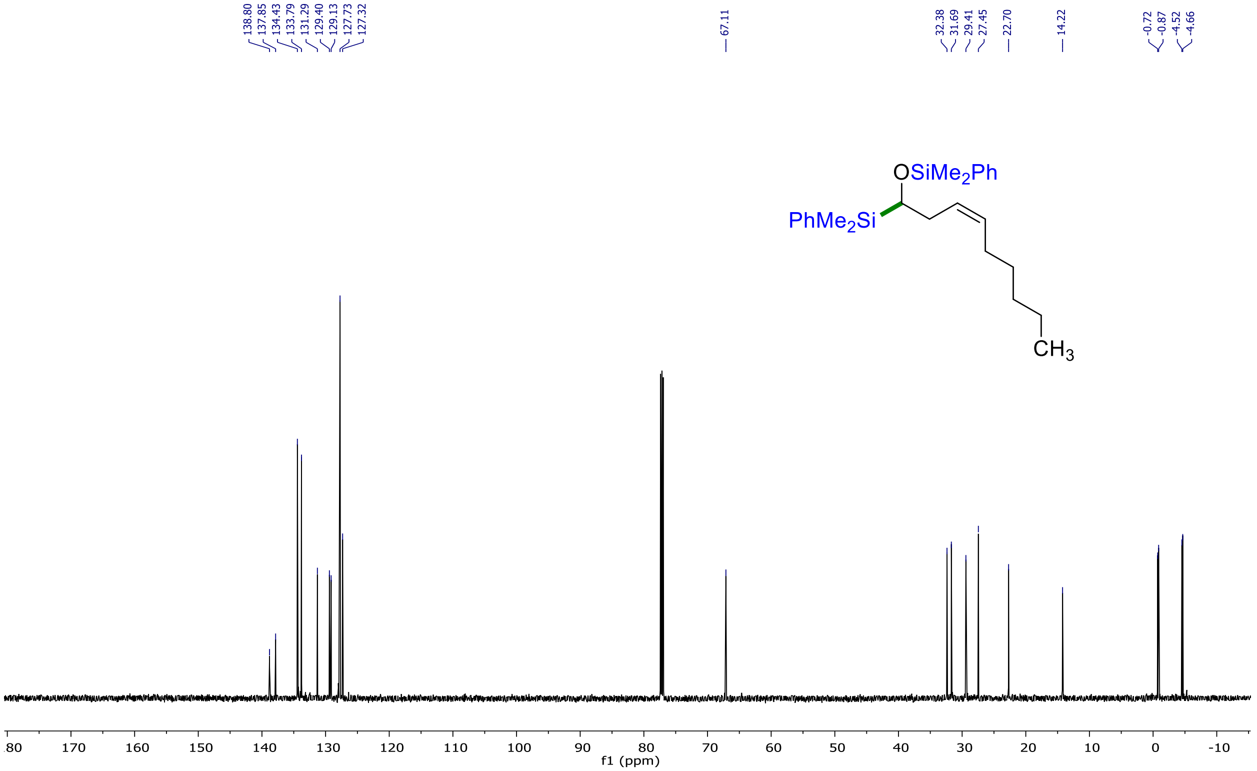

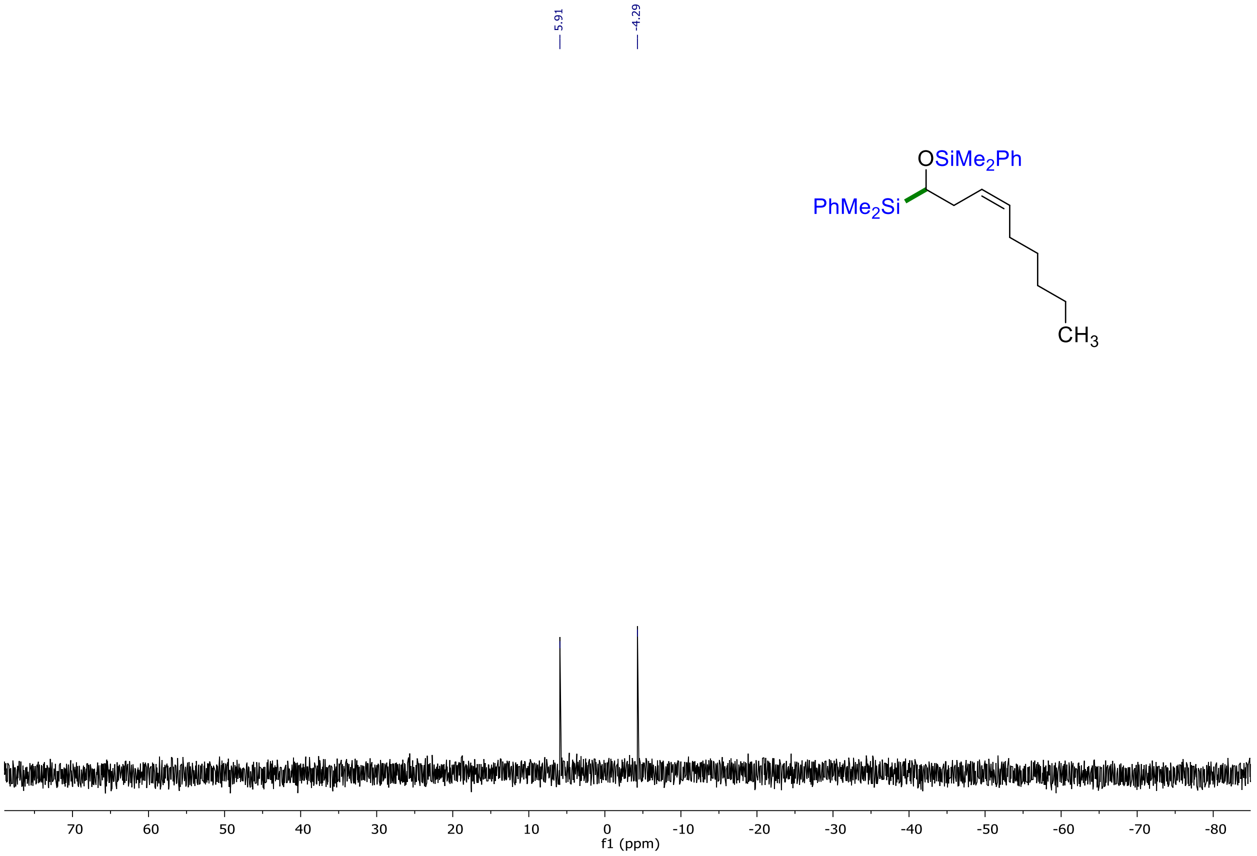

Supplementary Figure 54. ^{1}H and ^{13}C-NMR spectra of *Z*-**2e**

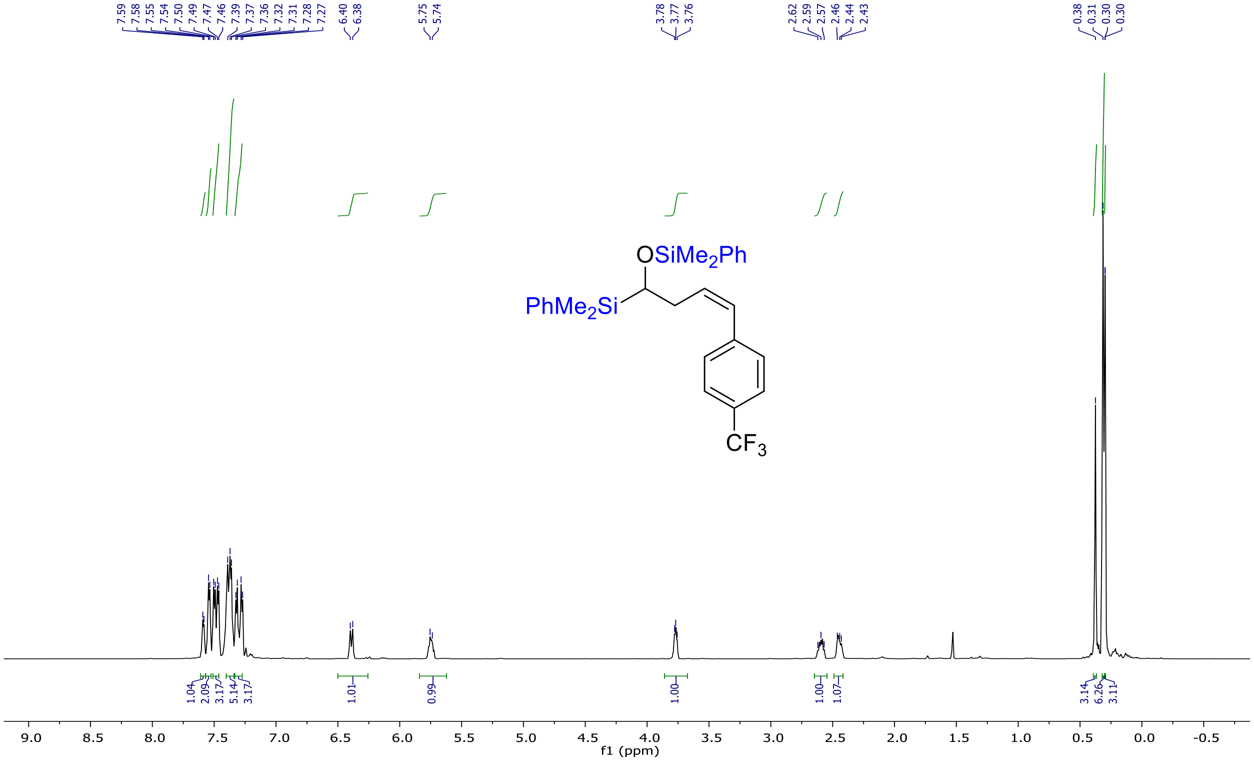

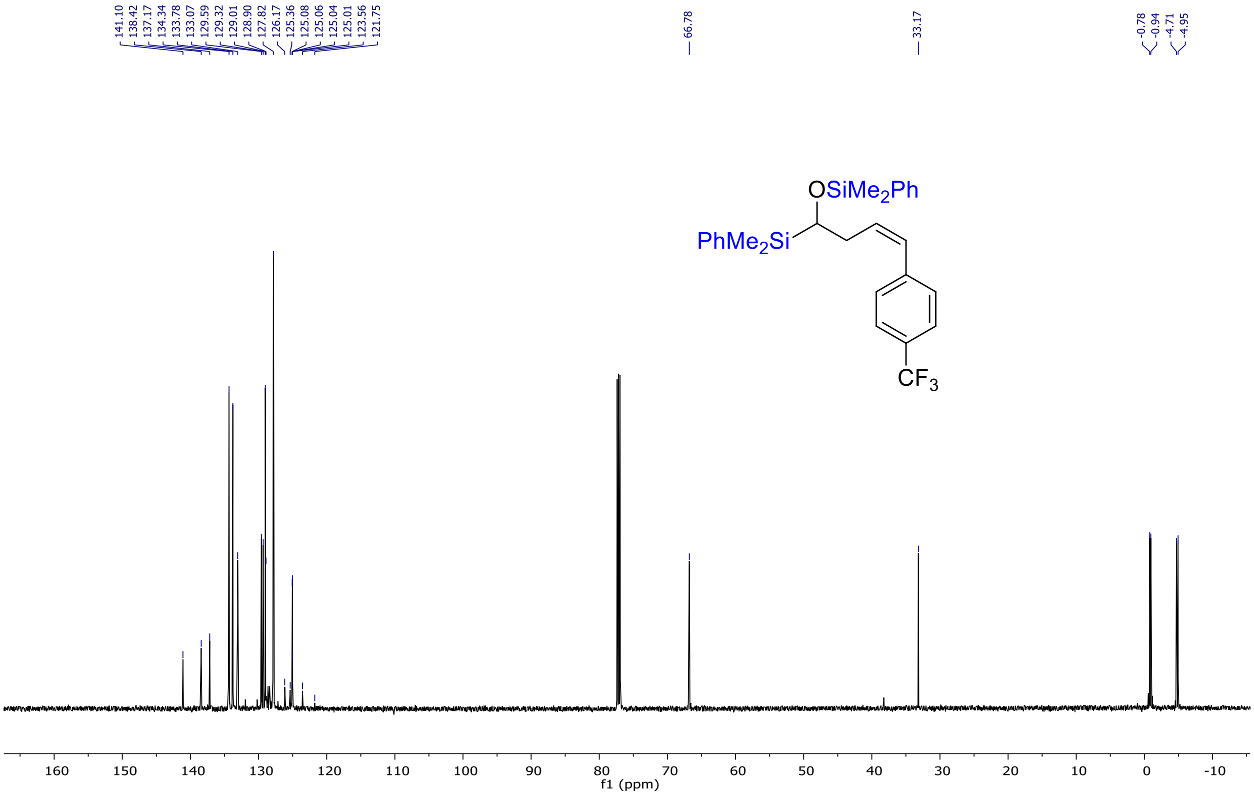

Supplementary Figure 55. ^{29}Si and ^{19}F-NMR spectra of *Z*-**2e**

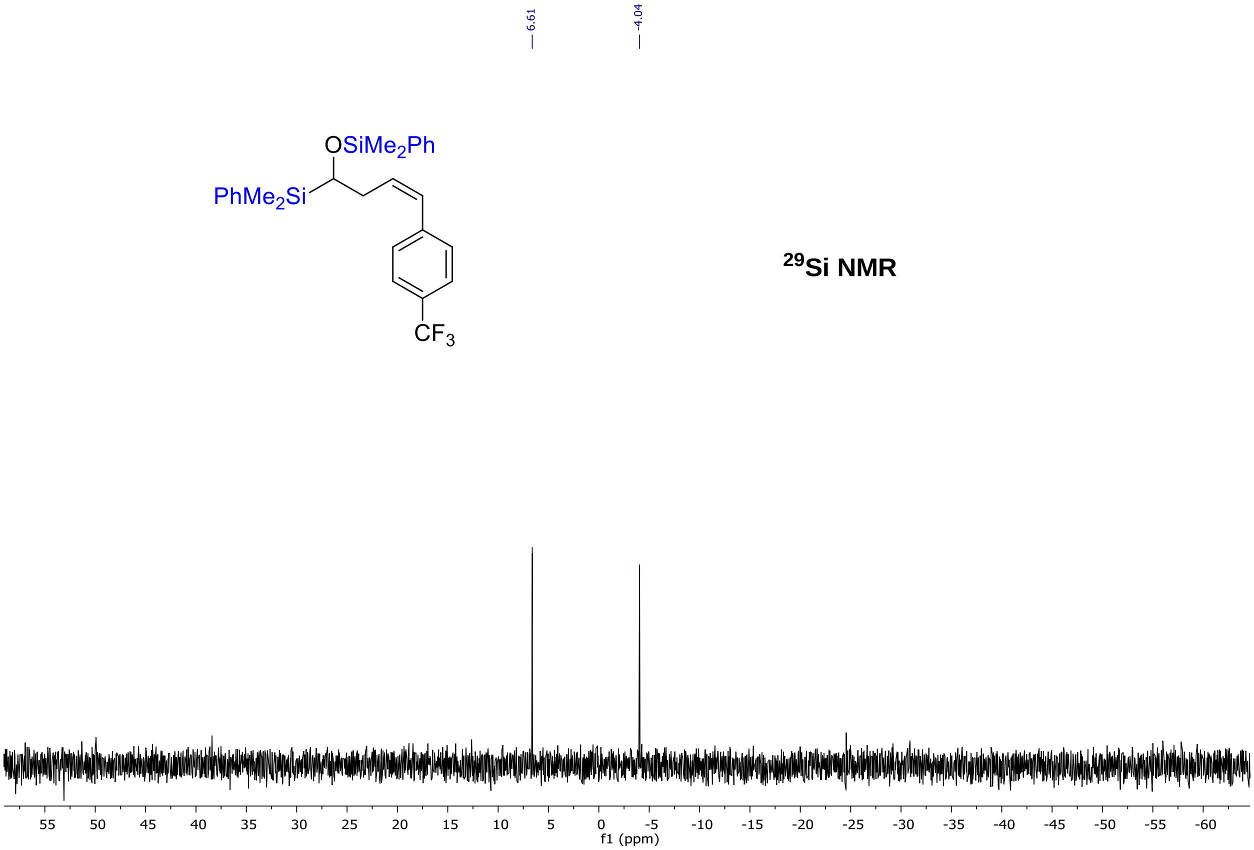

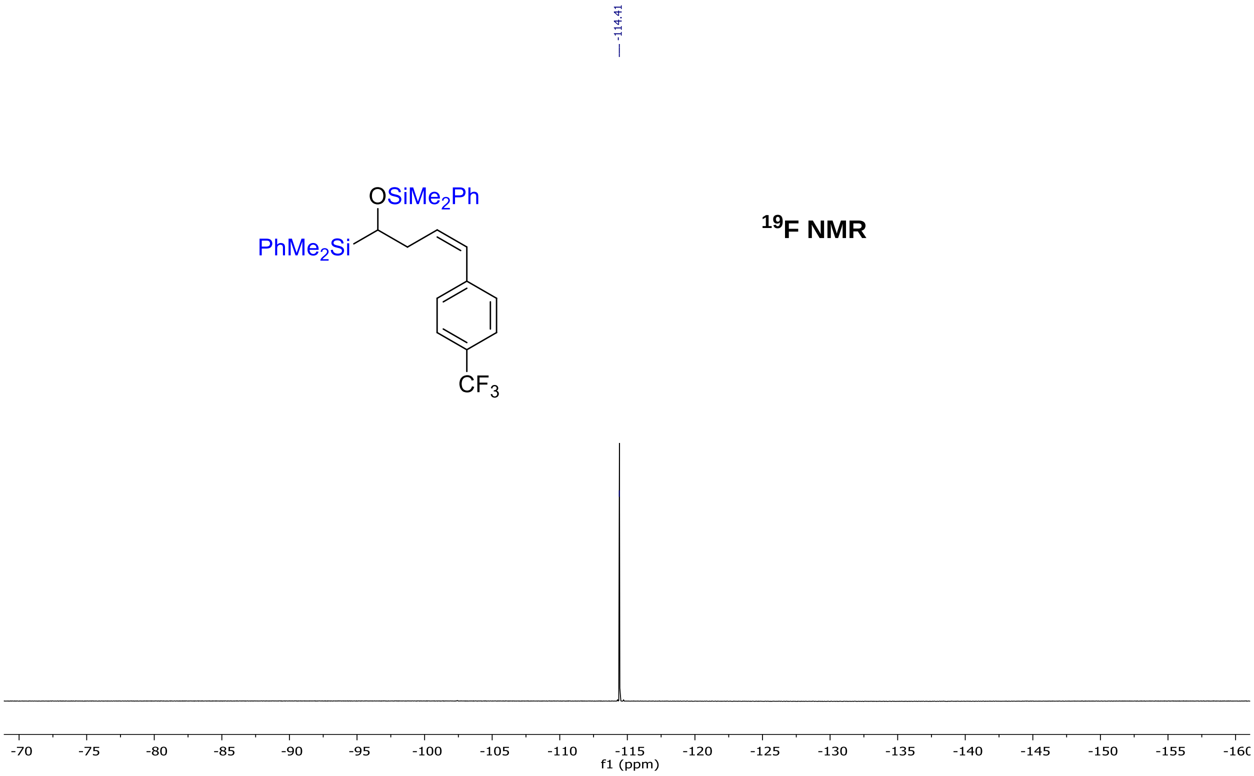

Supplementary Figure 56. ^{1}H and ^{13}C-NMR spectra of *Z*-**2f**

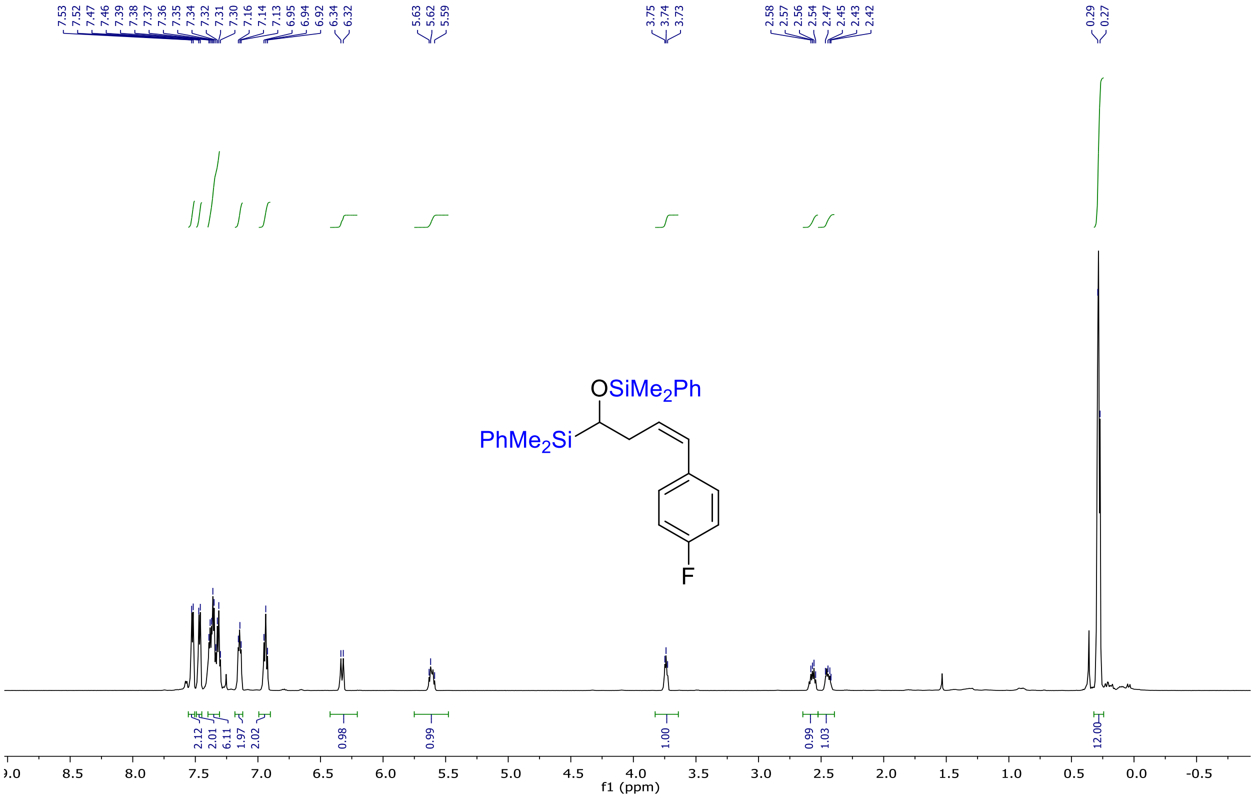

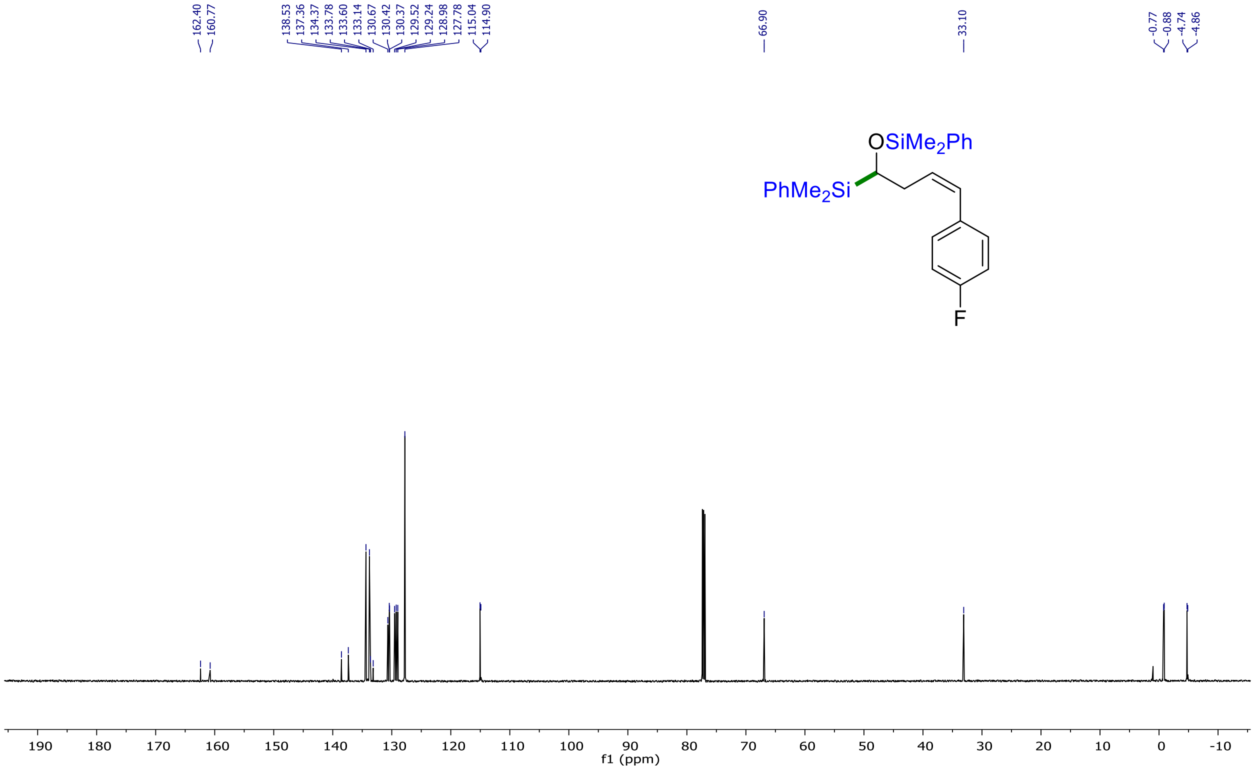

Supplementary Figure 57. ^{29}Si and ^{19}F-NMR spectra of *Z*-**2f**

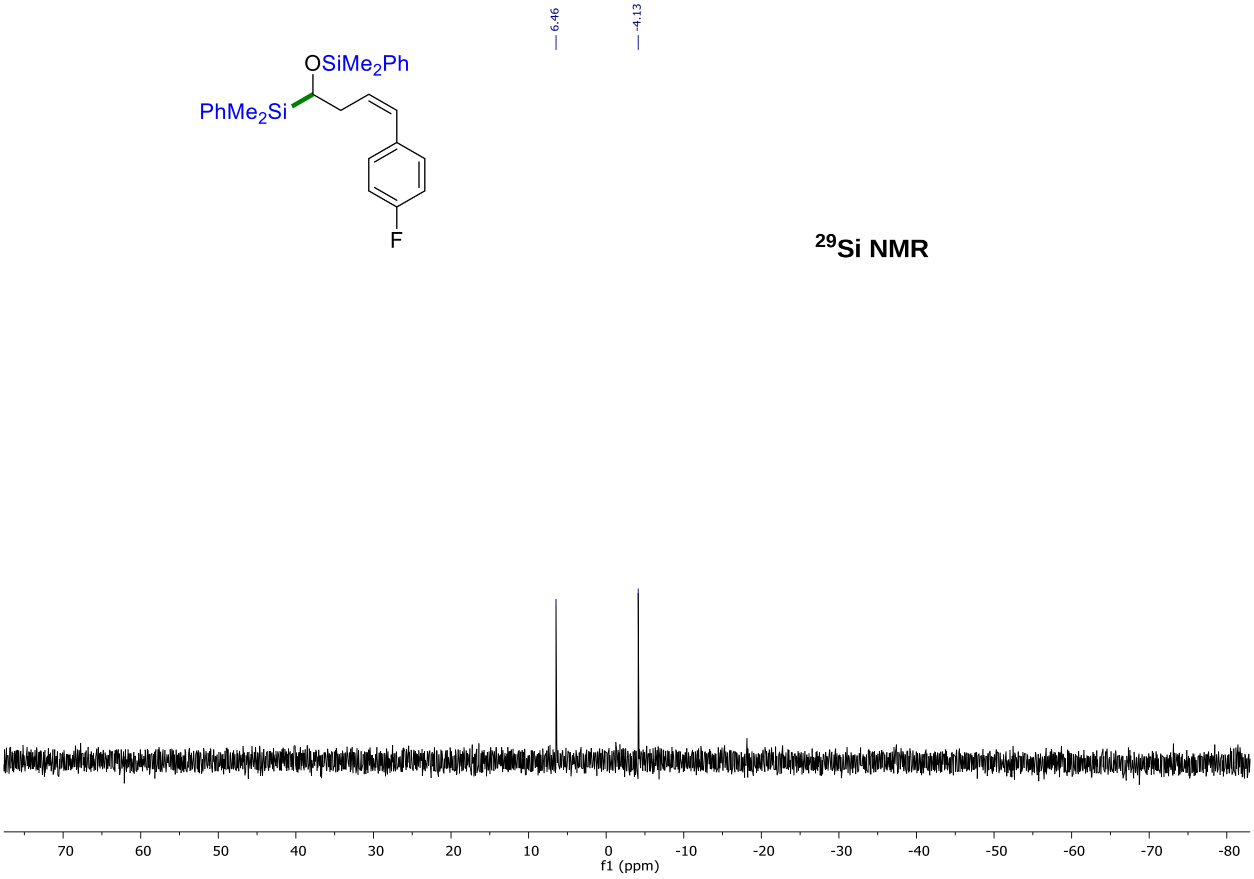

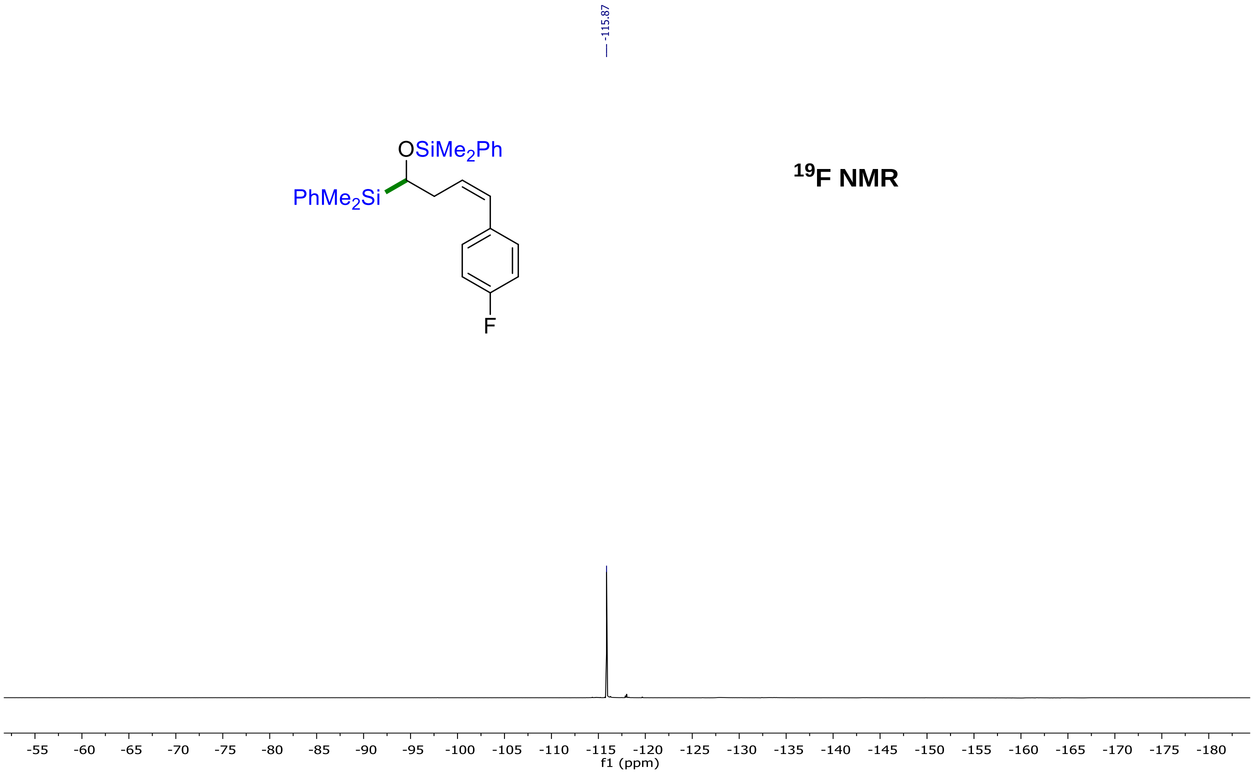

Supplementary Figure 58. ^{1}H and ^{13}C-NMR spectra of *Z*-**2g**

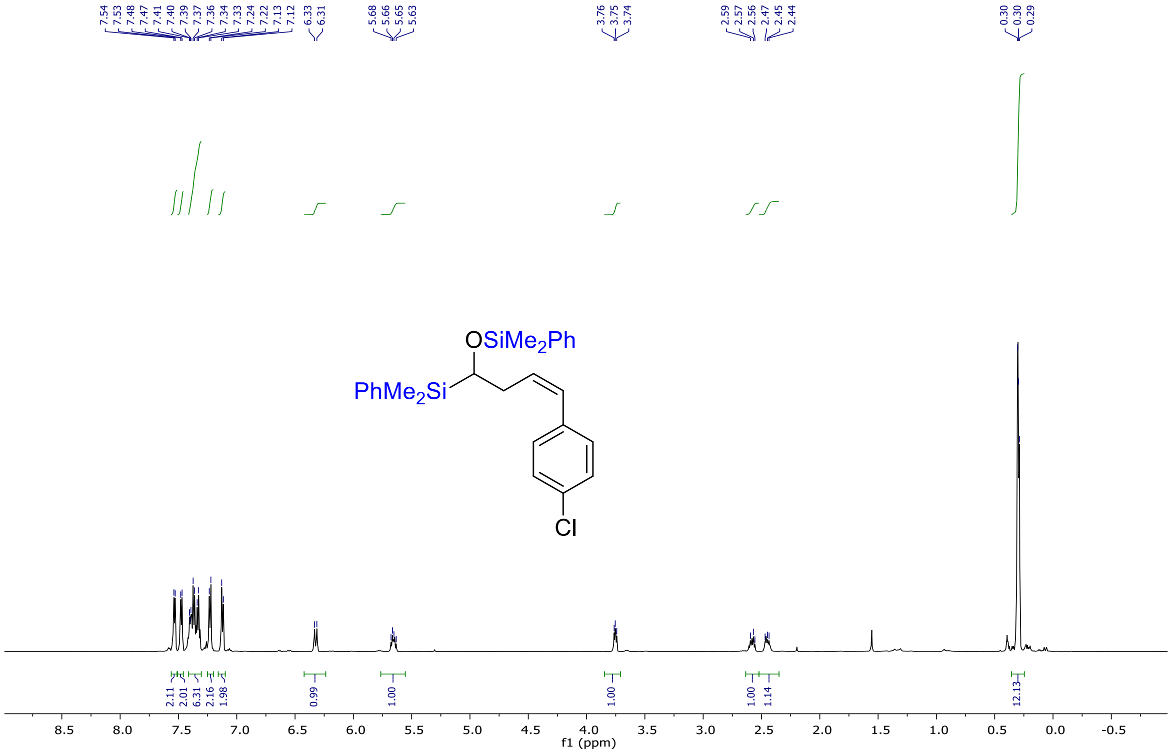

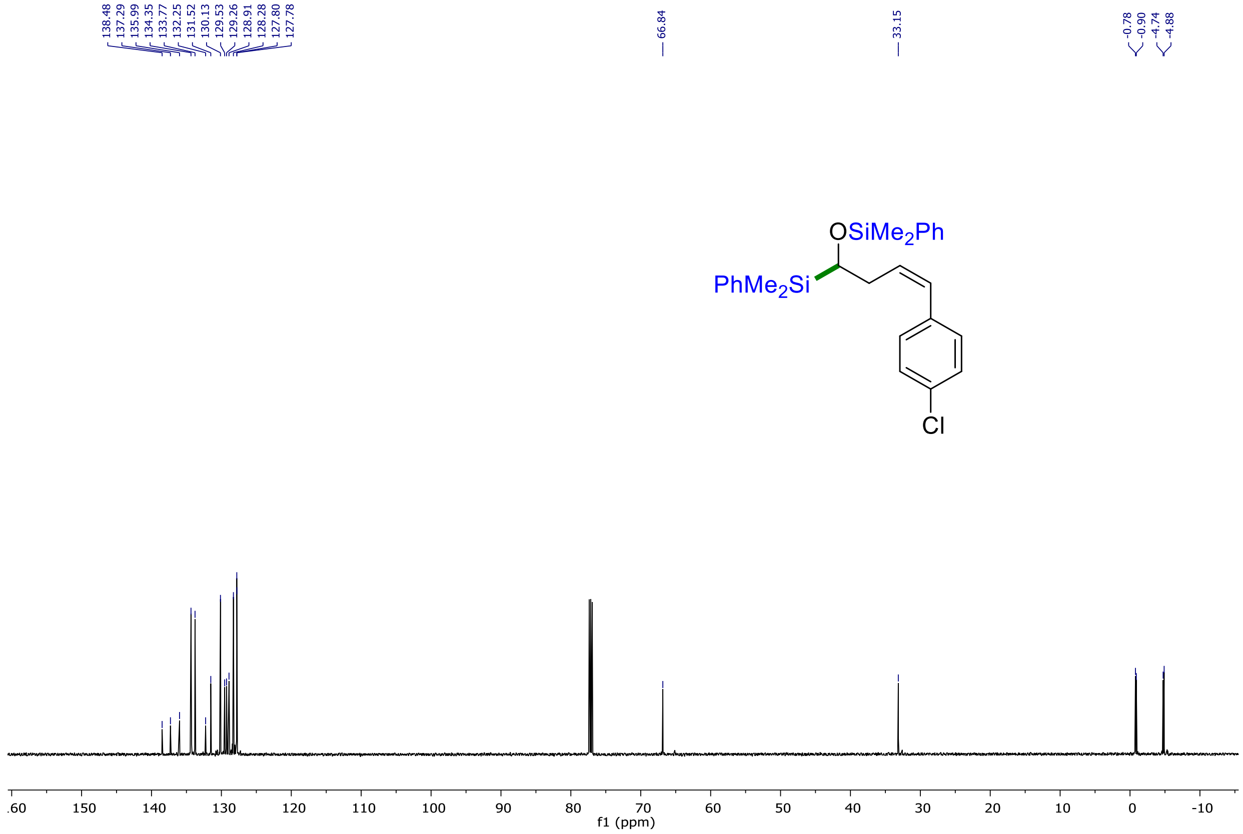

Supplementary Figure 59. ^{29}Si-NMR spectrum of *Z*-**2g**

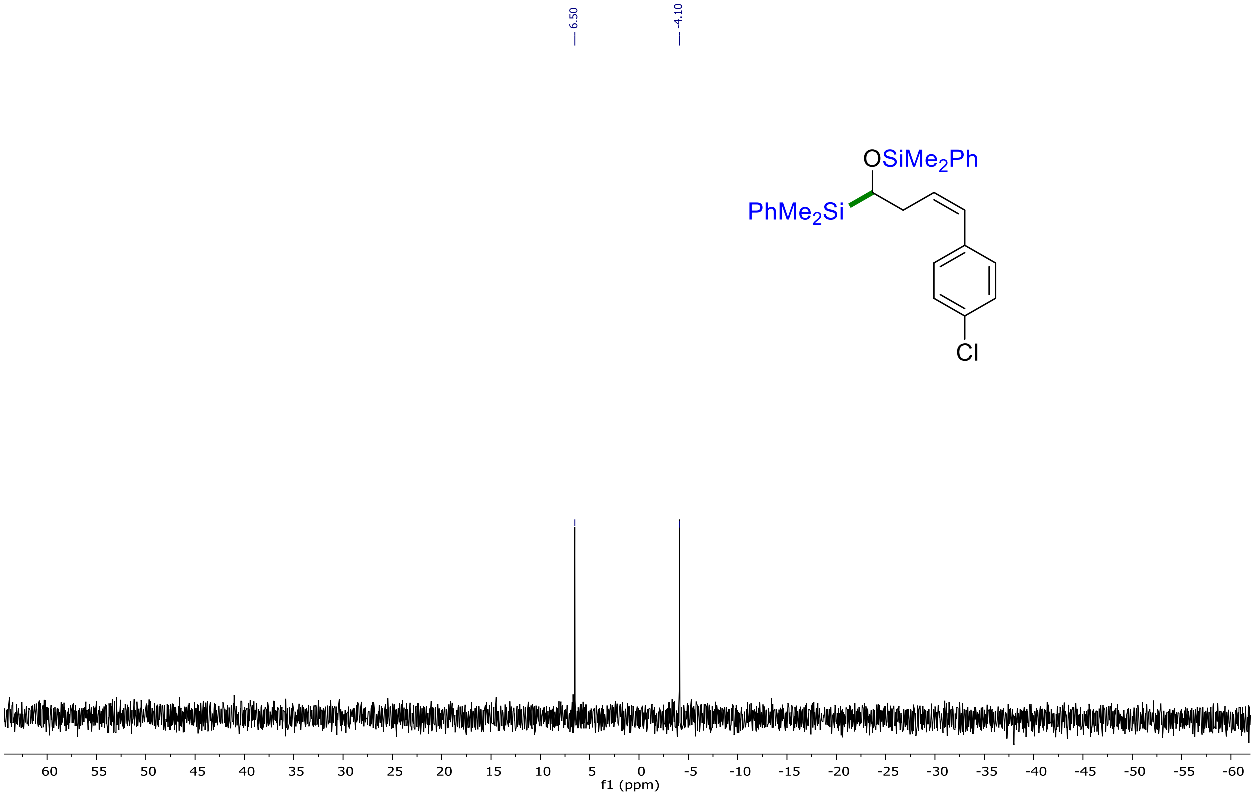

Supplementary Figure 60. ^{1}H-NMR spectrum of *Z*-**2h**

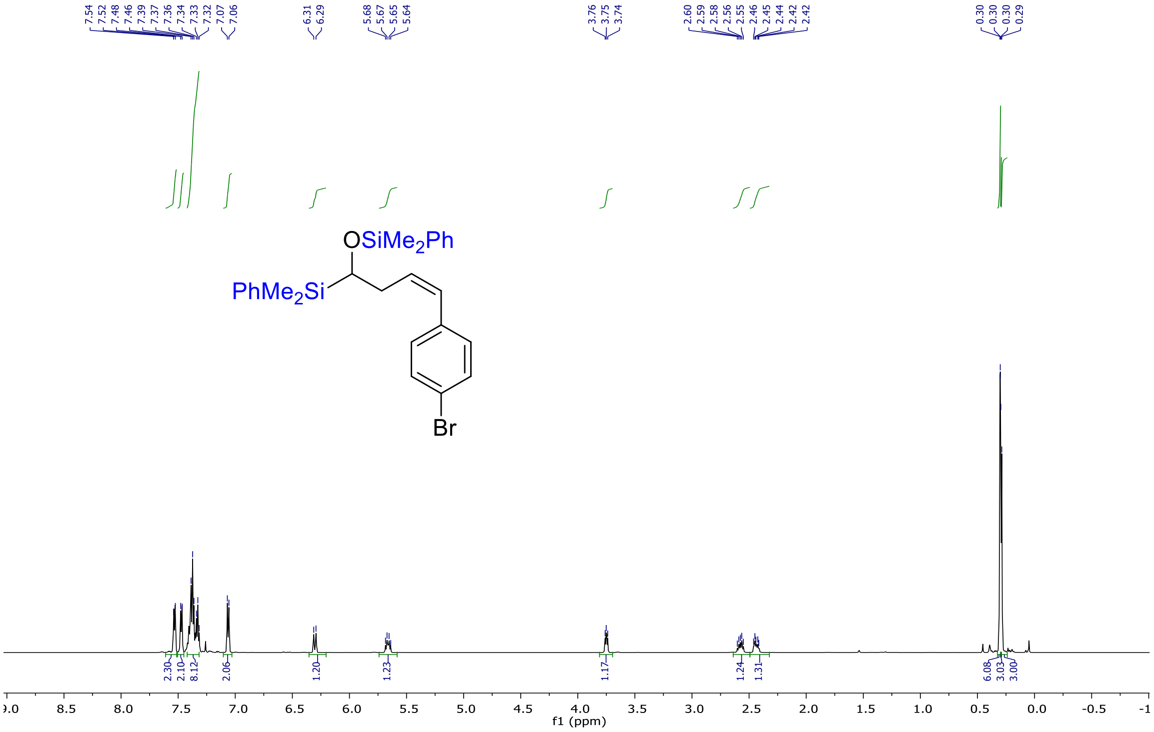

Supplementary Figure 61. ^{13}C and ^{29}Si-NMR spectra of *Z*-**2h**

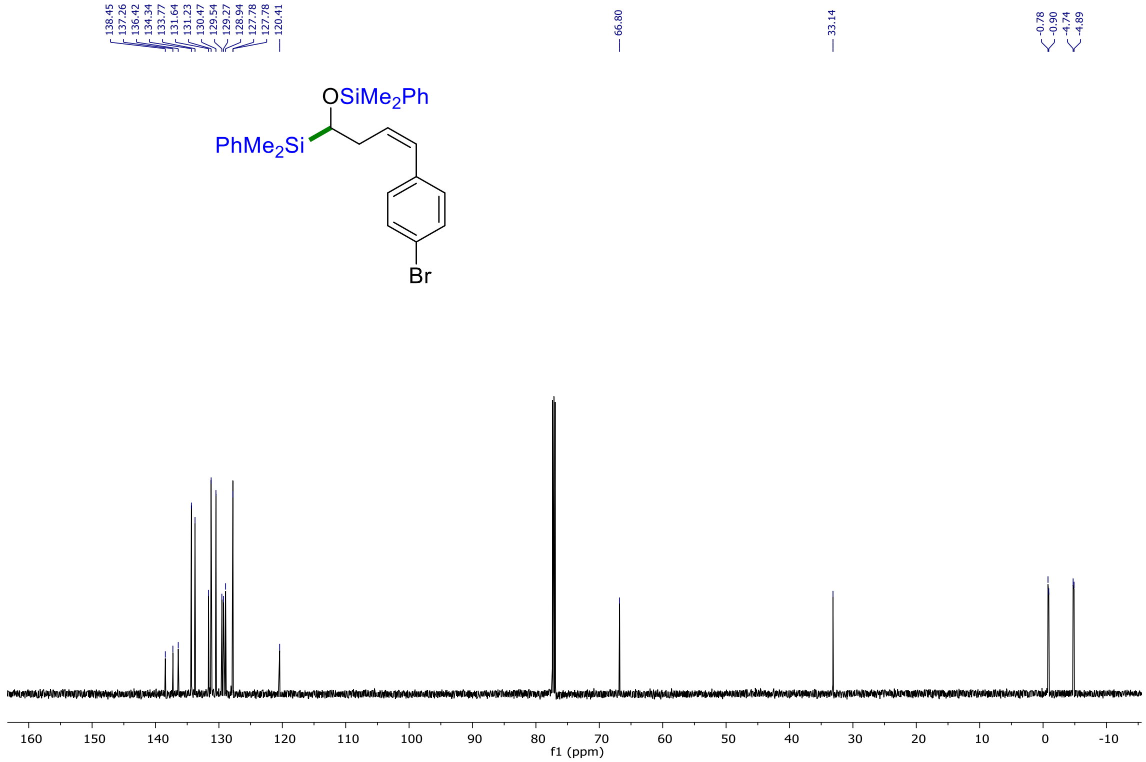

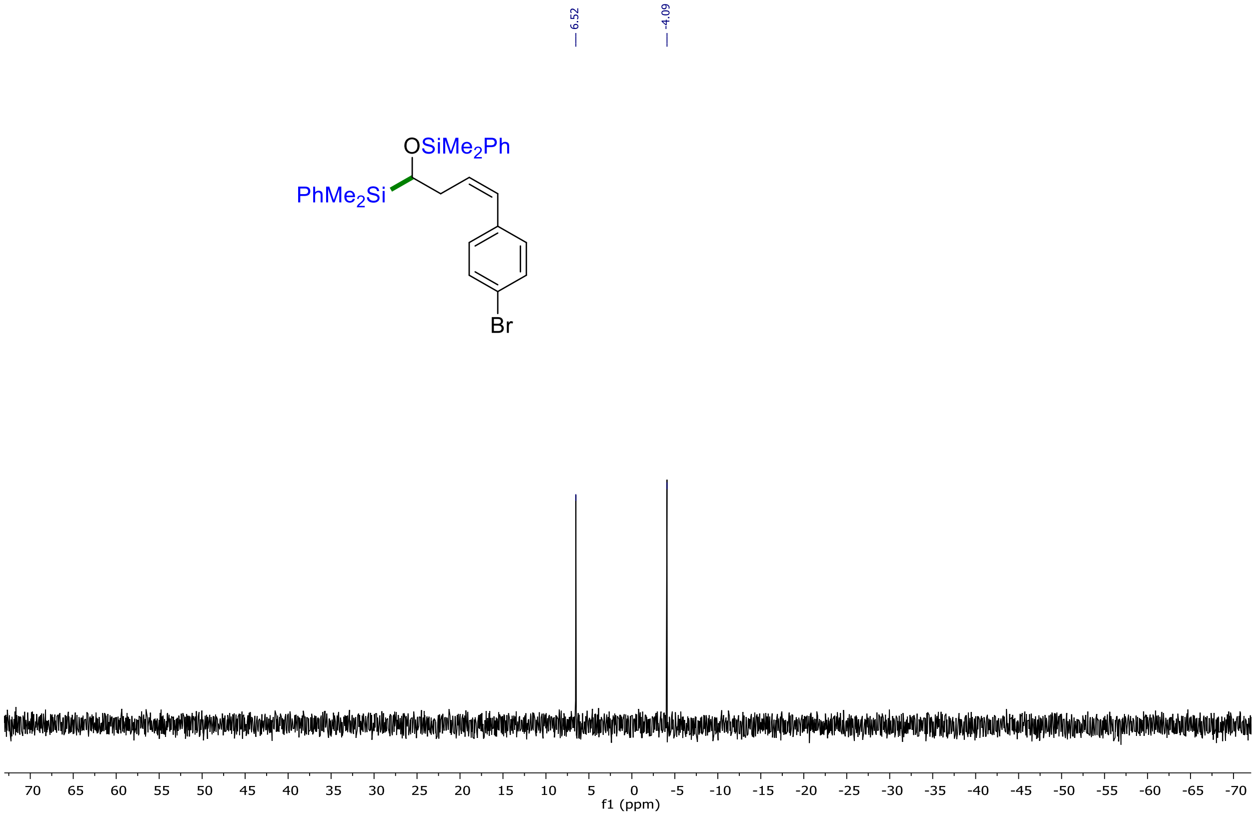

Supplementary Figure 62. ^{1}H and ^{13}C-NMR spectra of *Z*-**2i**

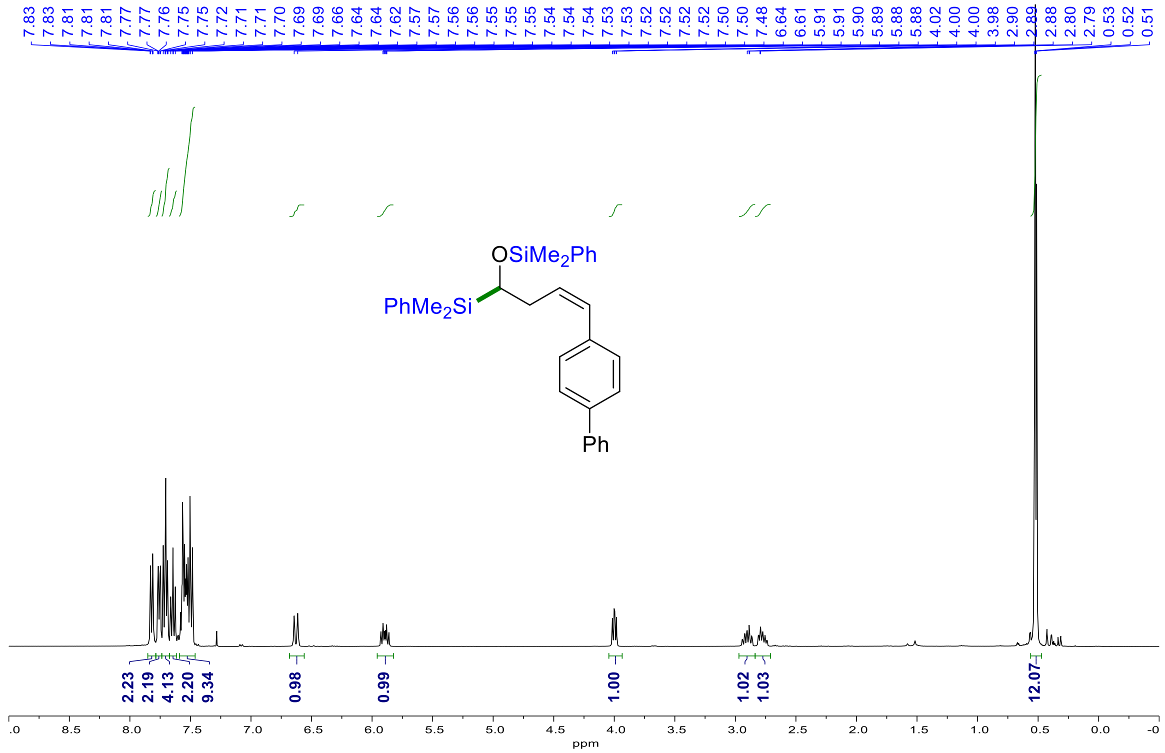

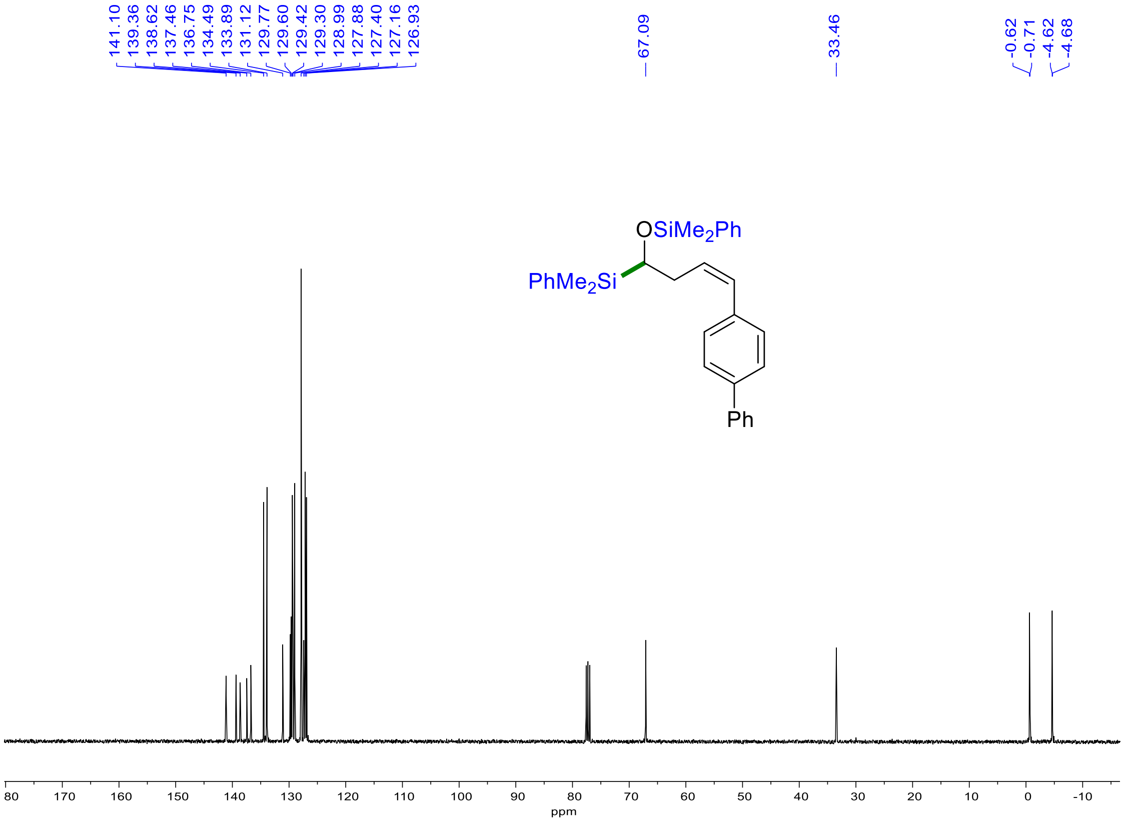

Supplementary Figure 63. ^{29}Si-NMR spectrum of *Z*-**2i**

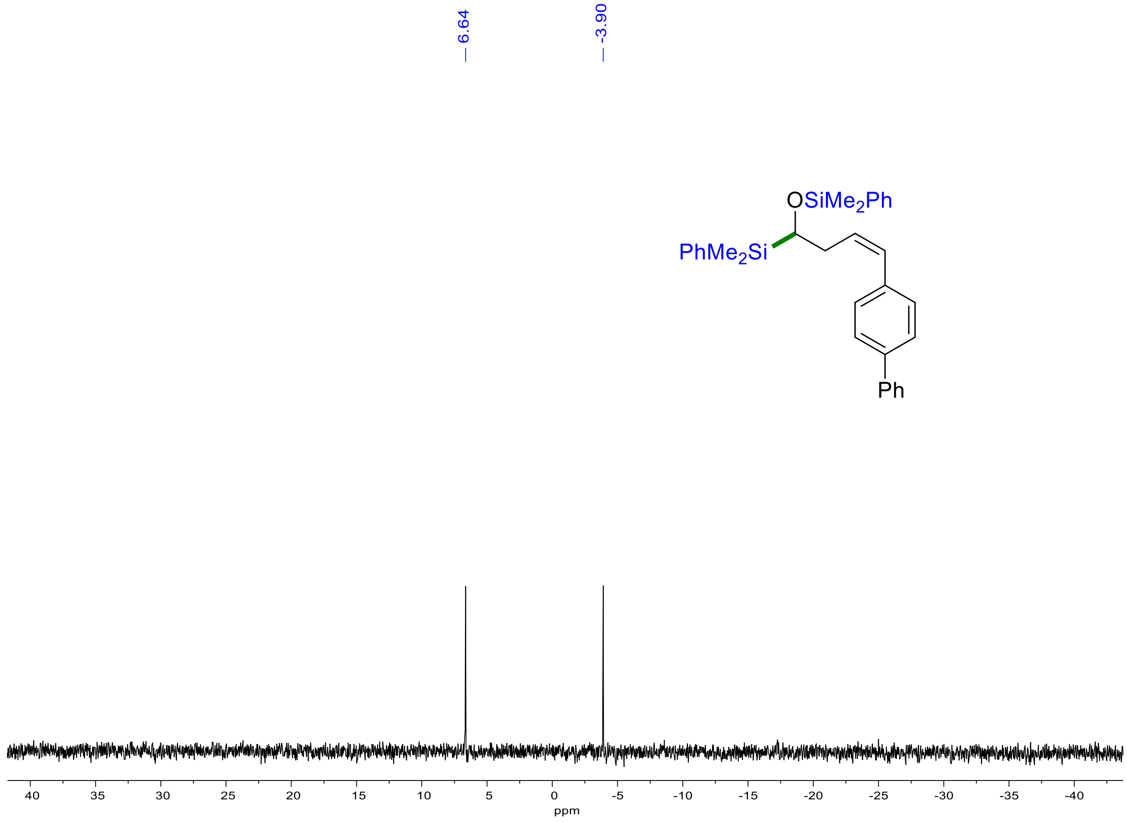

Supplementary Figure 64. ^{1}H-NMR spectrum of *Z*-**2j**

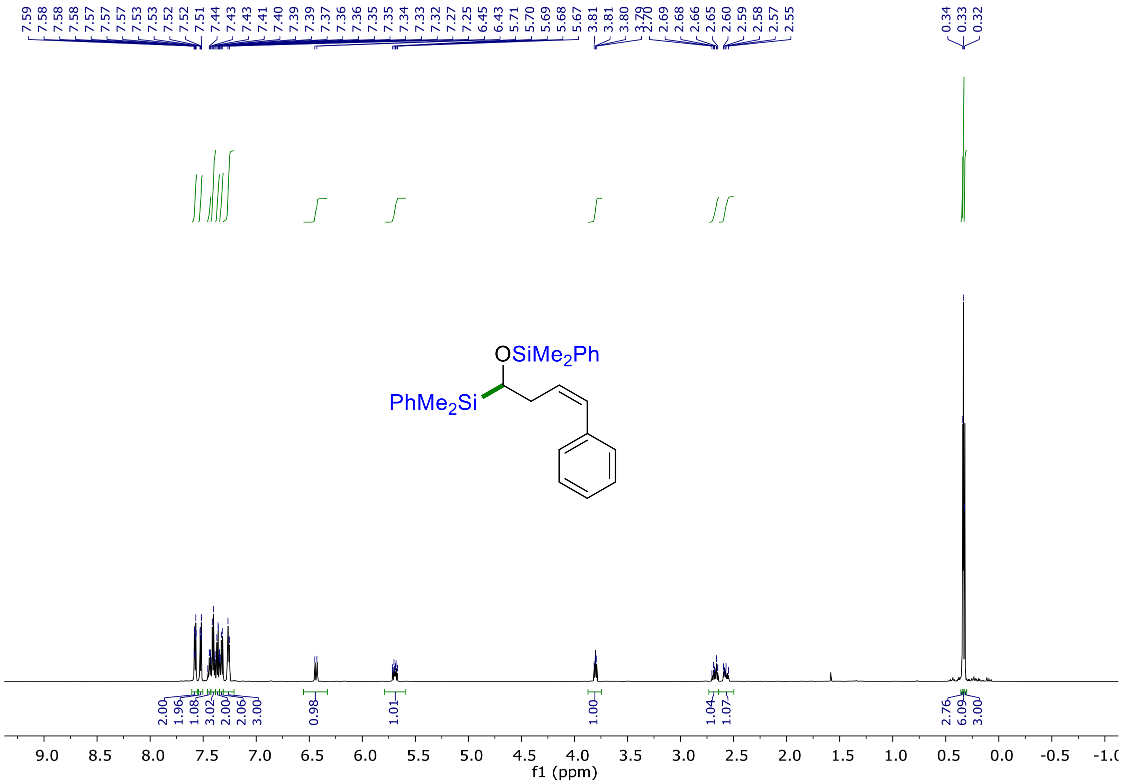

Supplementary Figure 65. ^{13}C and ^{29}Si-NMR spectra of *Z*-**2j**

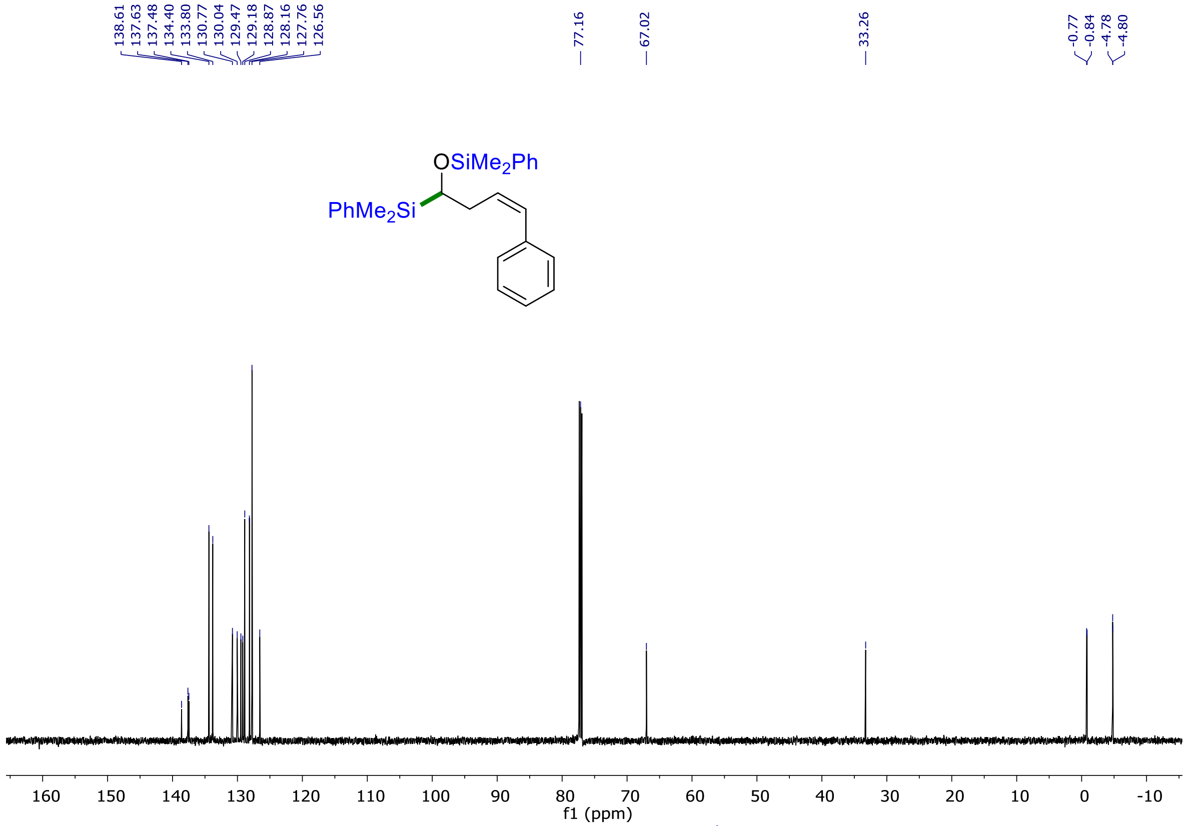

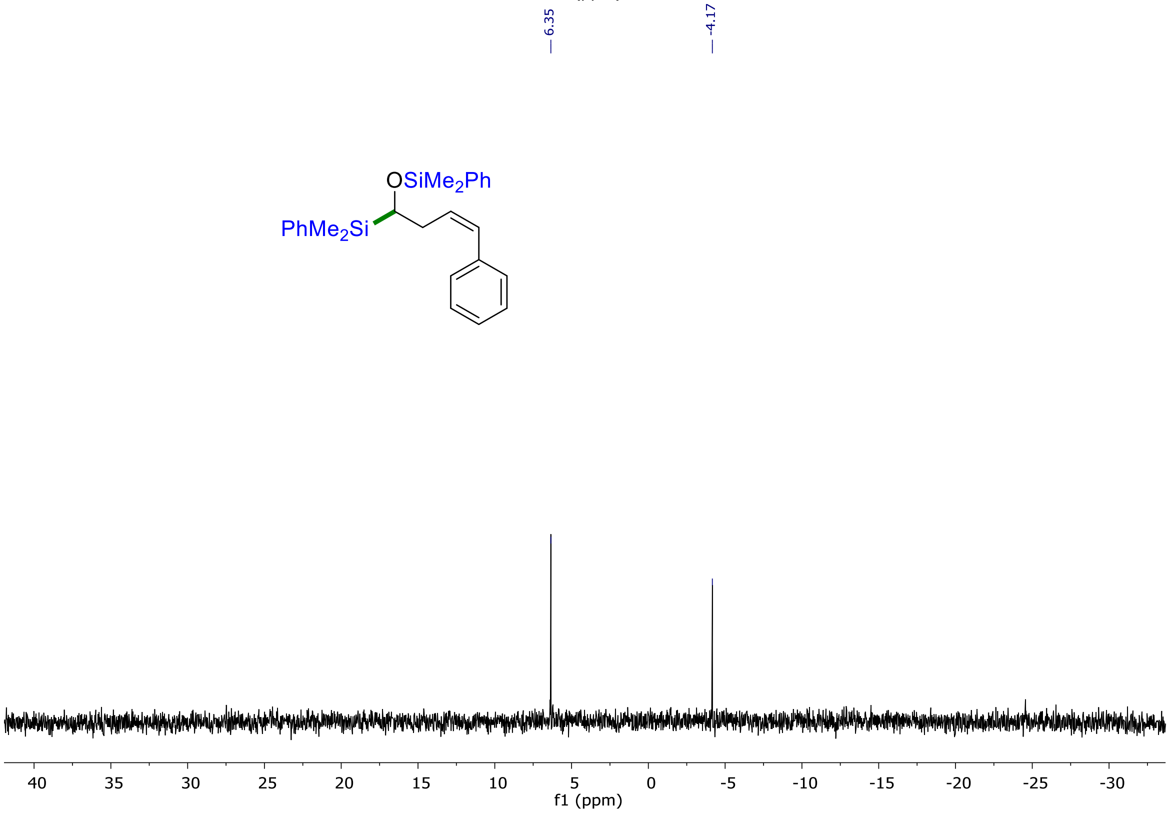

Supplementary Figure 66. ^{1}H and ^{13}C-NMR spectra of *Z*-**2k**

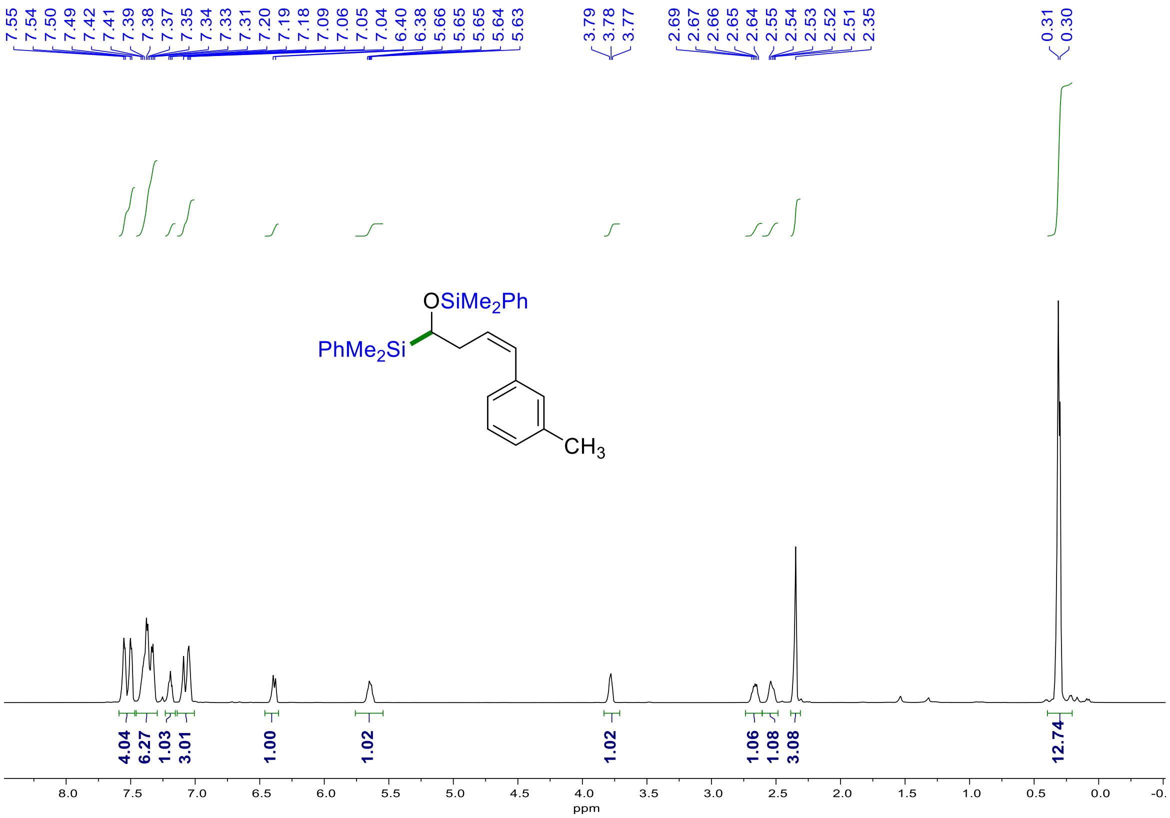

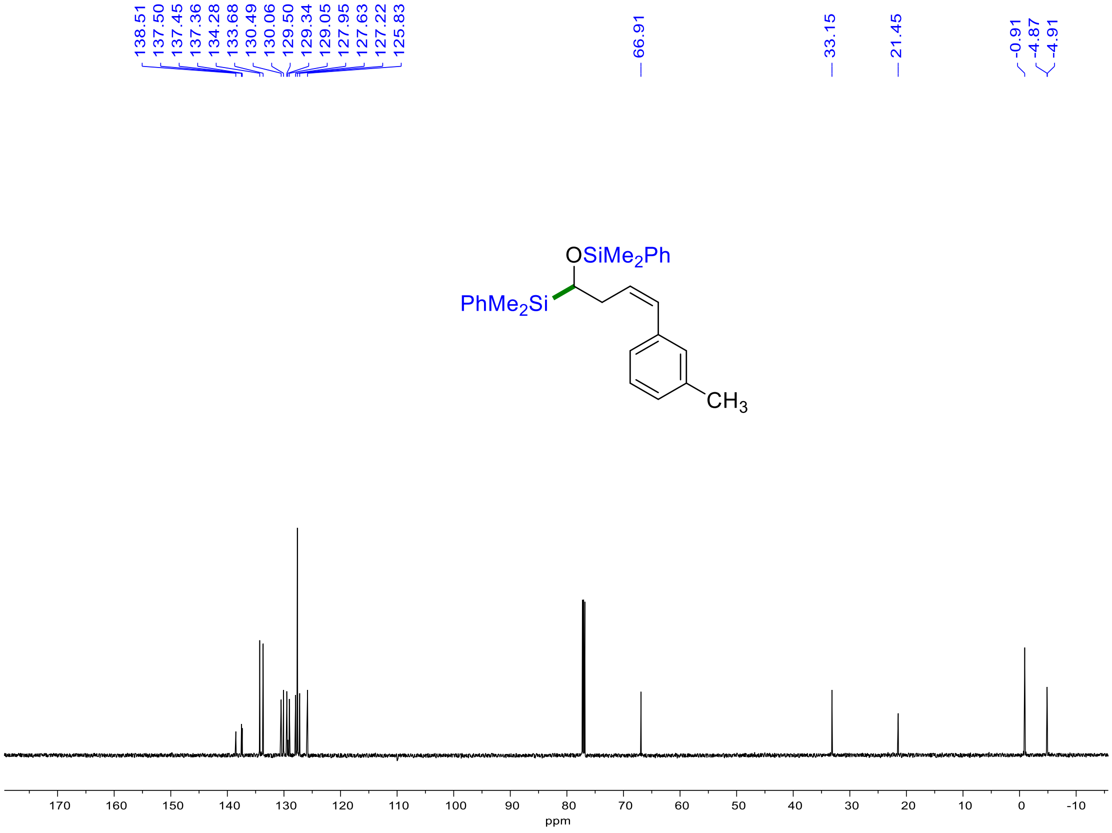

Supplementary Figure 67. ^{29}Si-NMR spectrum of *Z*-**2k**

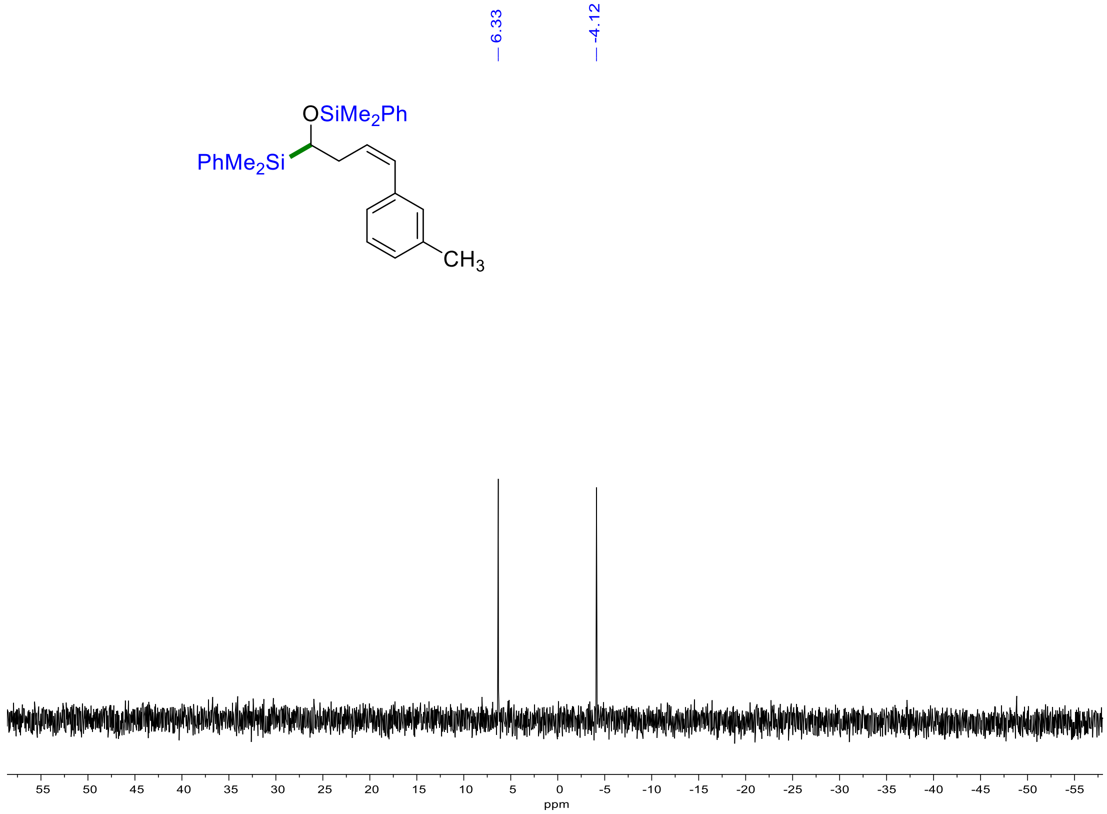

Supplementary Figure 68. ^{1}H-NMR spectrum of *Z*-**2l**

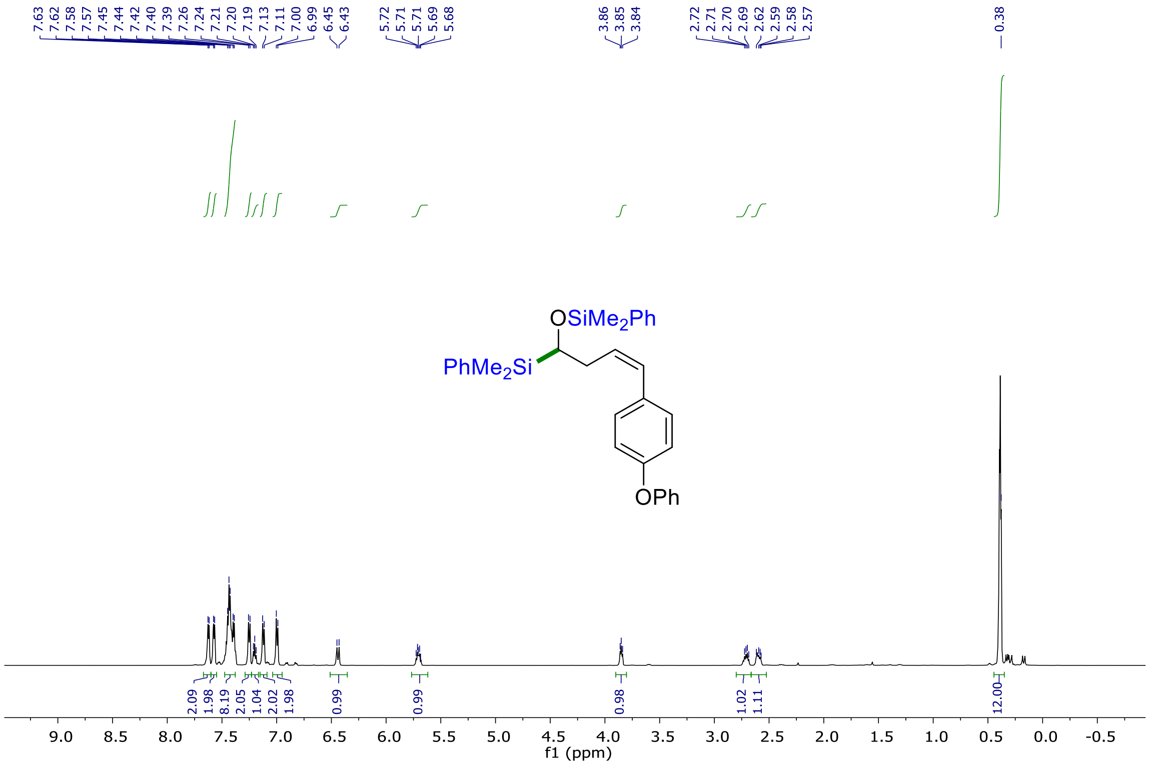

Supplementary Figure 69. ^{13}C and ^{29}Si-NMR spectra of *Z*-**2l**

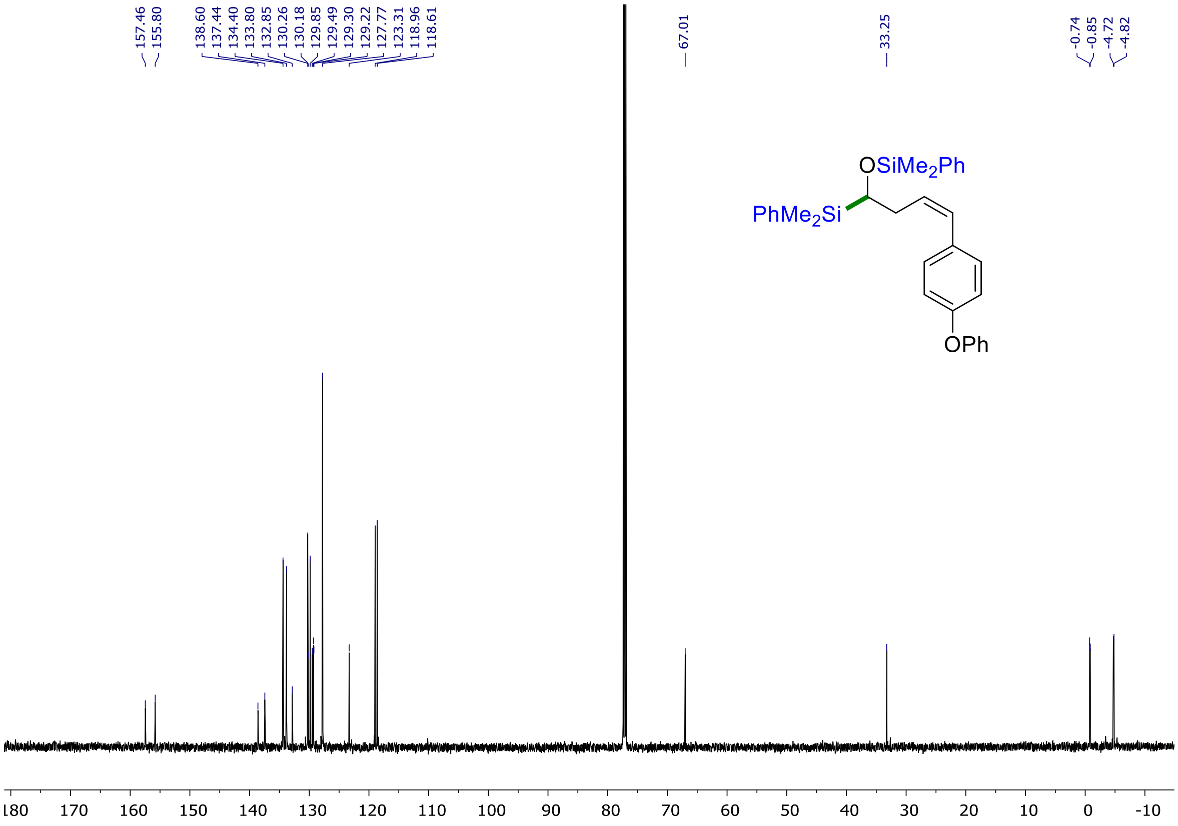

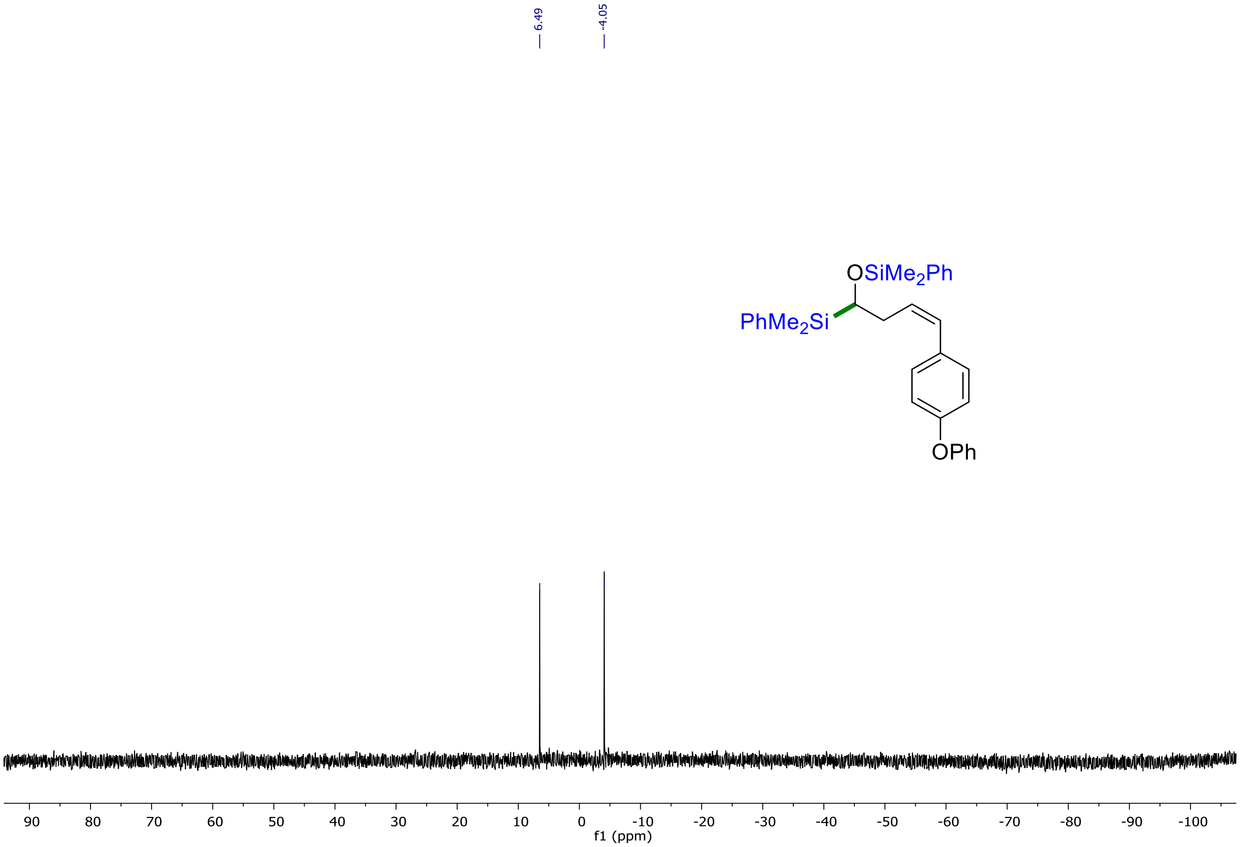

Supplementary Figure 70. ^{1}H and ^{13}C-NMR spectra of *Z*-**2m**

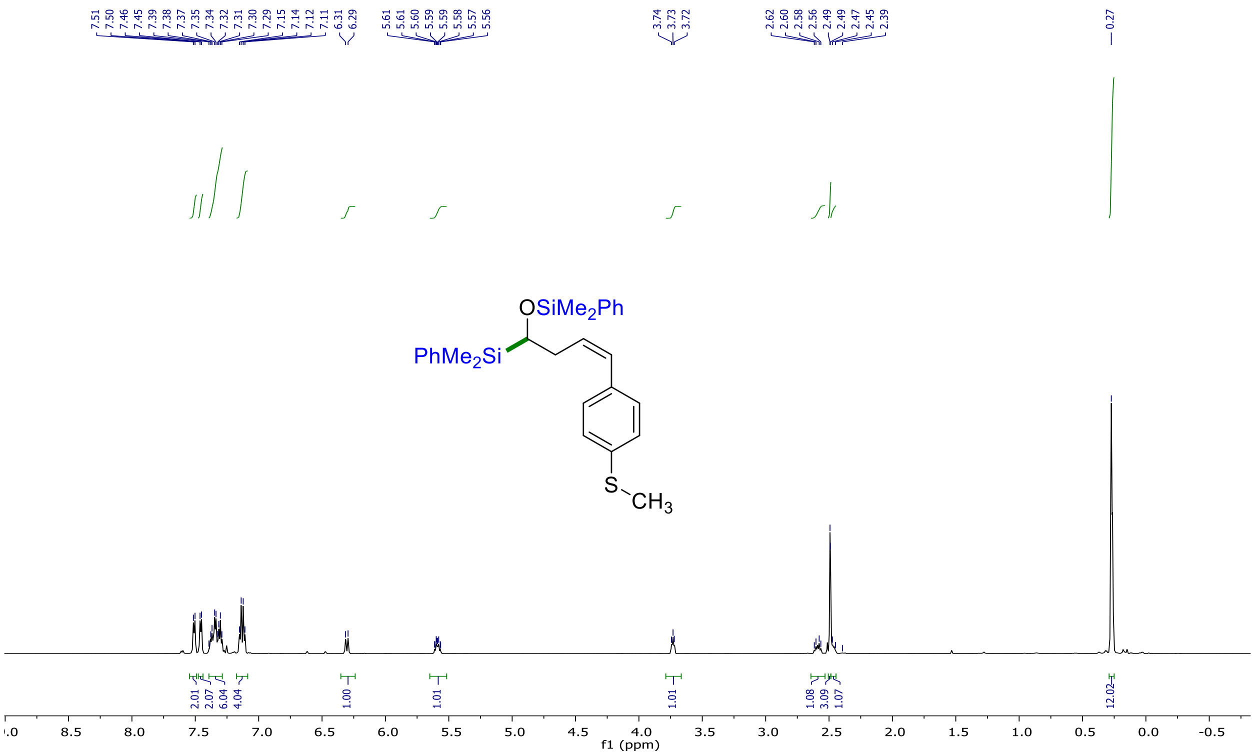

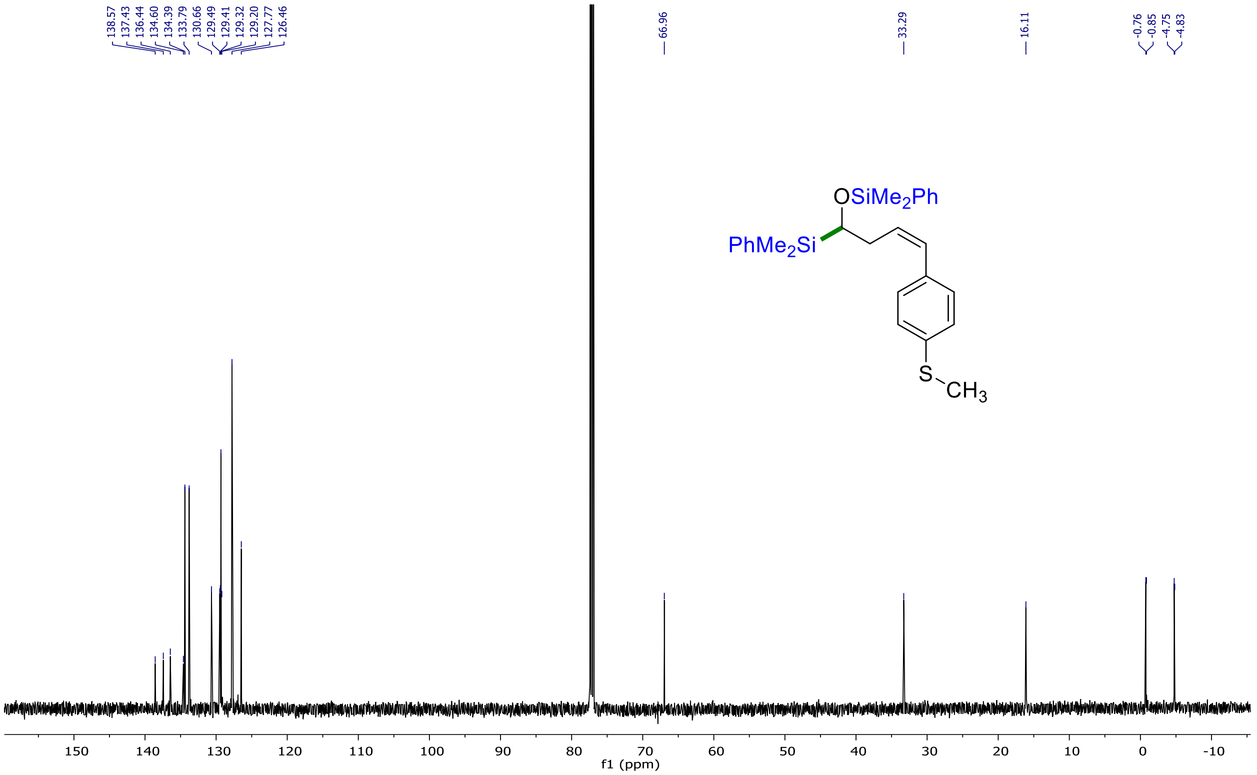

Supplementary Figure 71. ^{29}Si-NMR spectrum of *Z*-**2m**

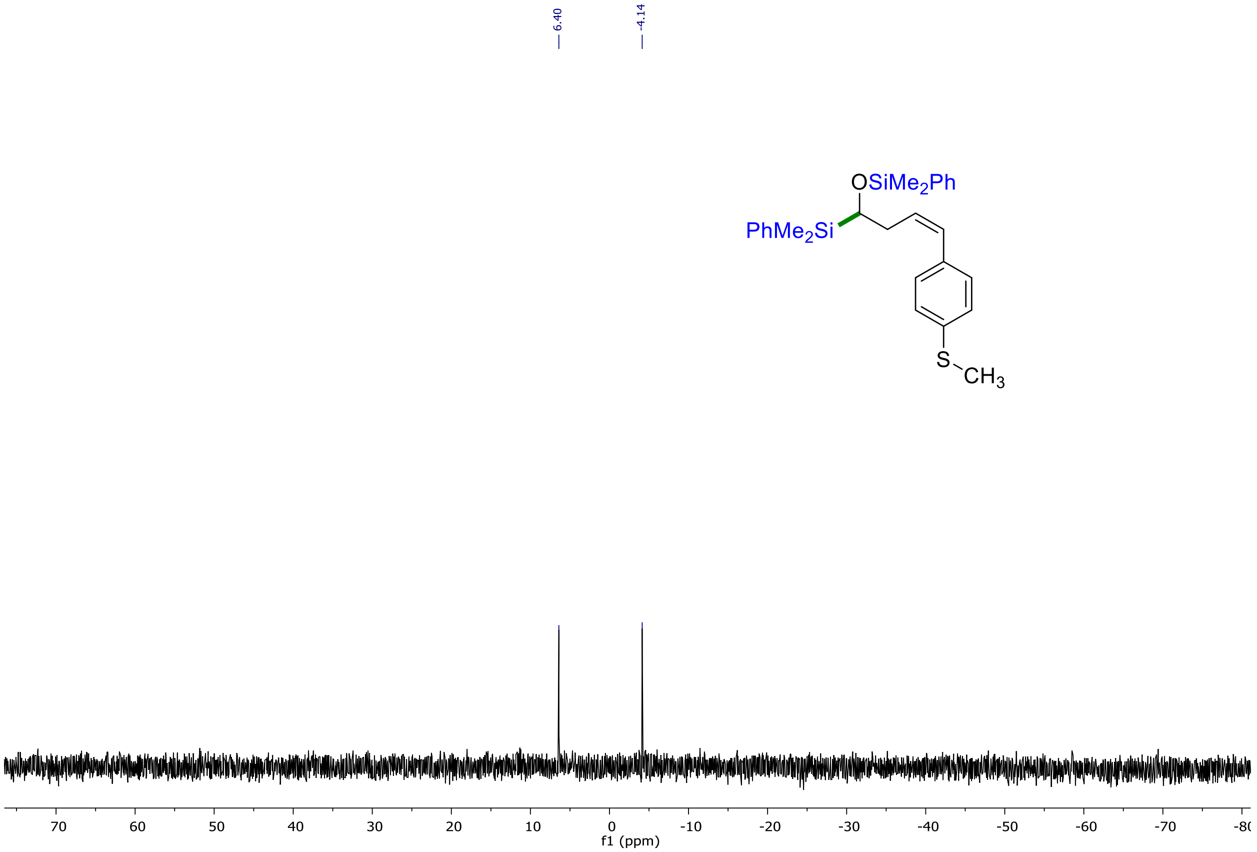

Supplementary Figure 72. ^{1}H-NMR spectrum of *Z*-**2n**

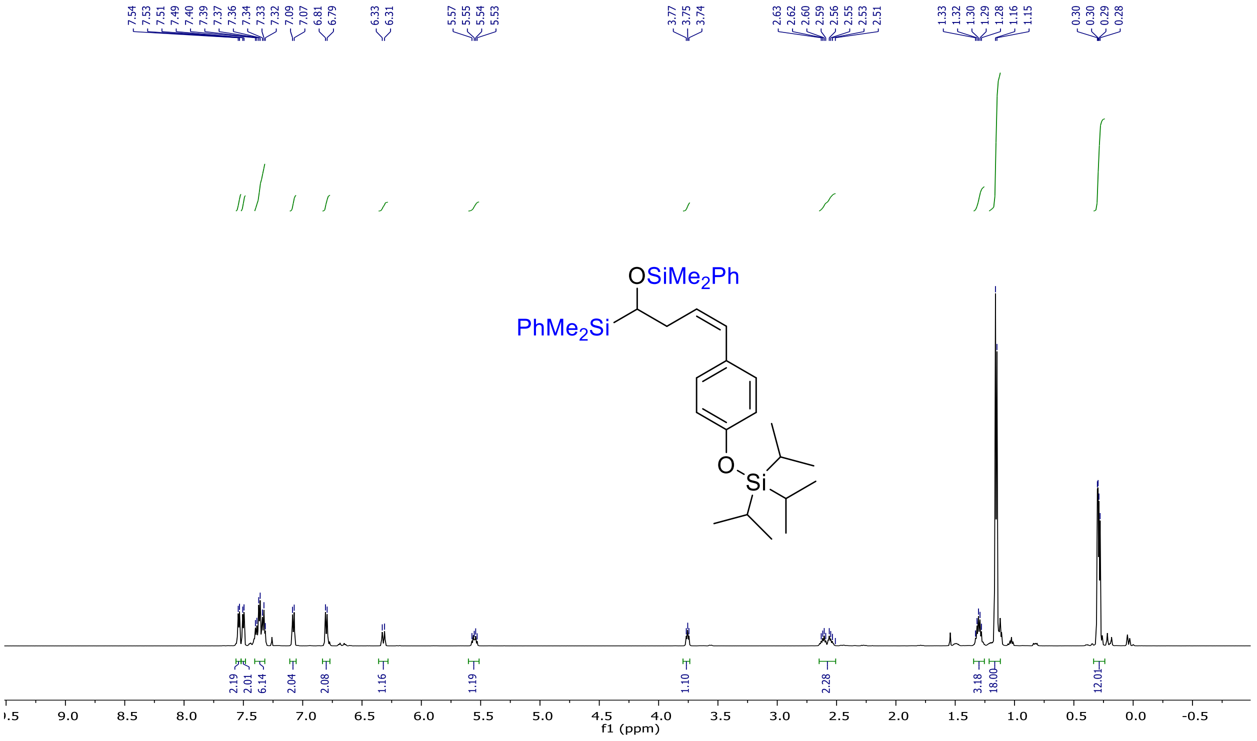

Supplementary Figure 73. ^{13}C and ^{29}Si-NMR spectra of *Z*-**2n**

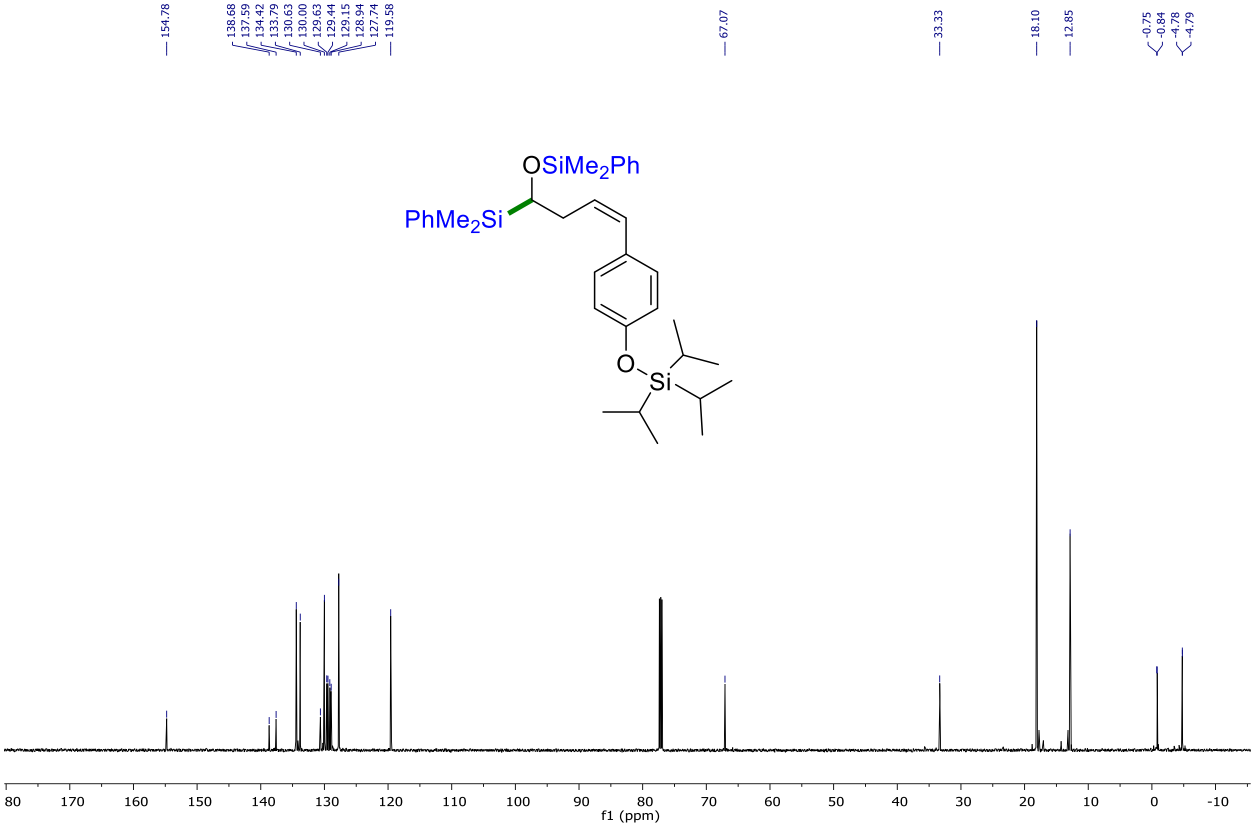

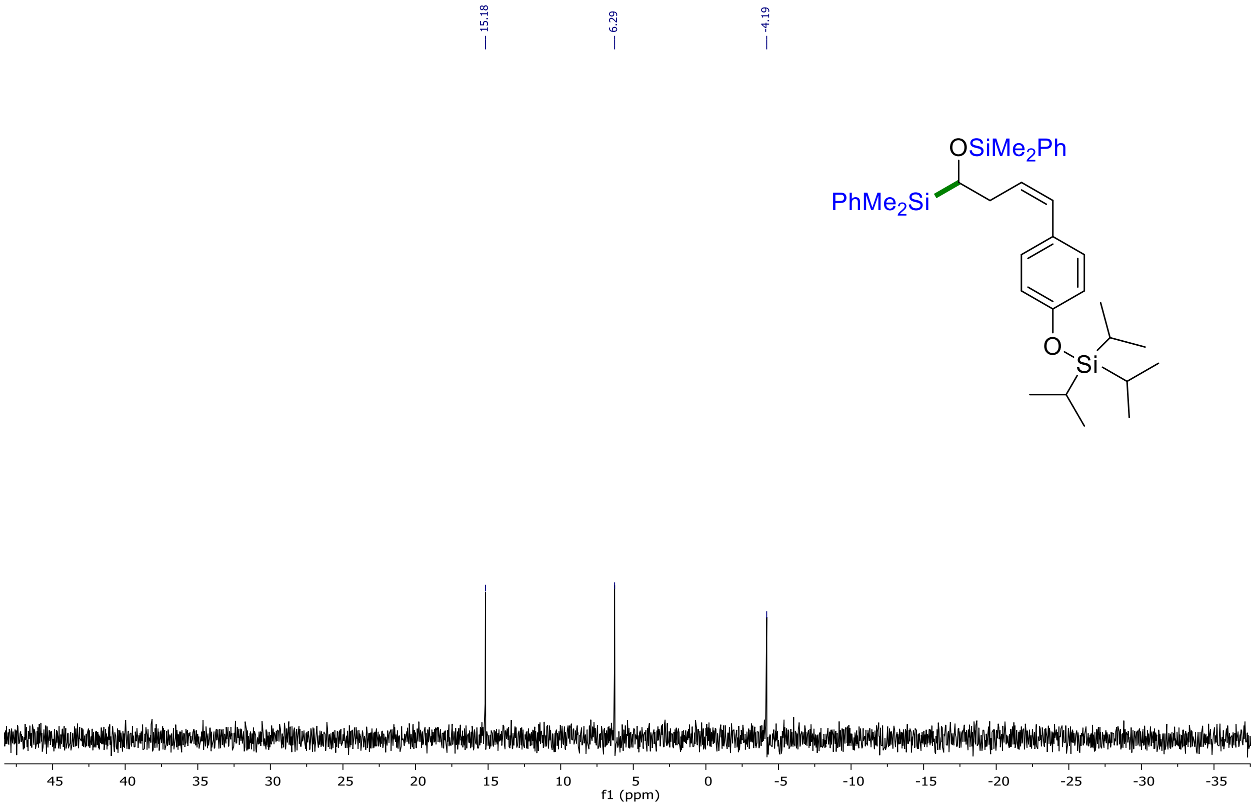

Supplementary Figure 74. ^{1}H and ^{13}C-NMR spectra of *Z*-**2o**

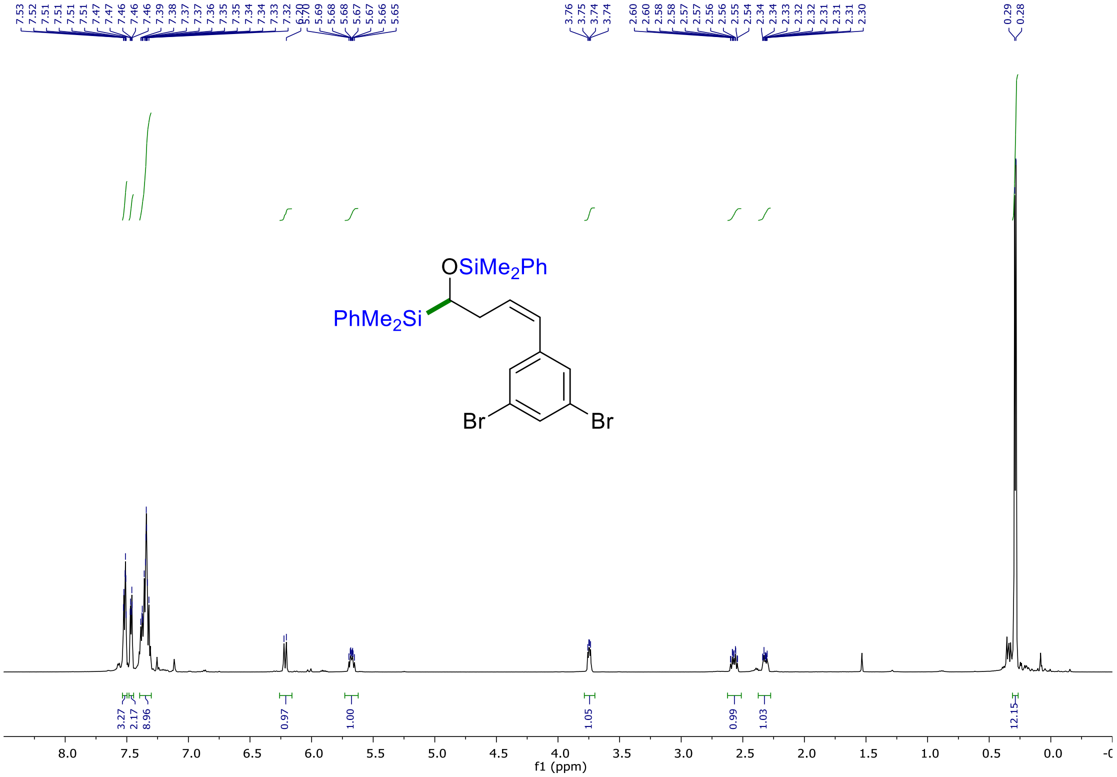

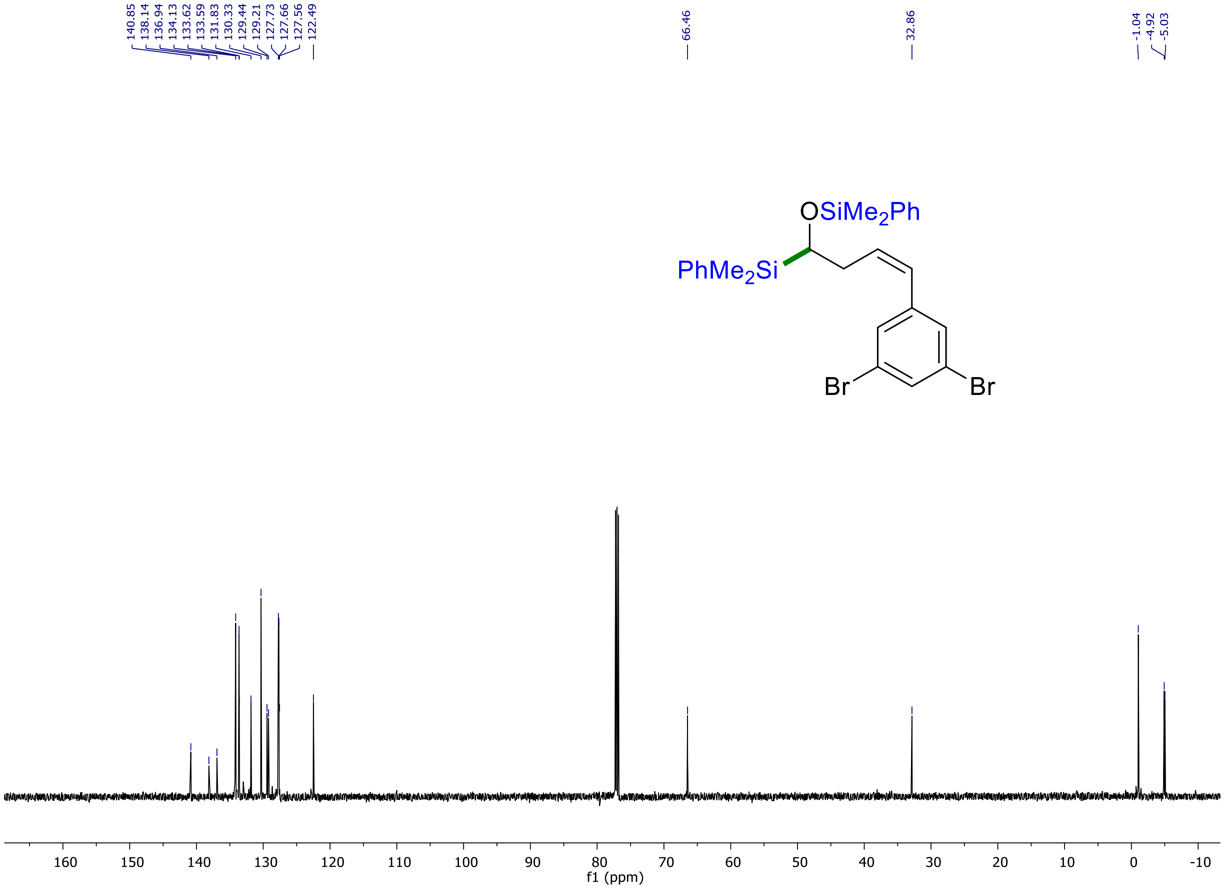

Supplementary Figure 75. ^{29}Si-NMR spectrum of *Z*-**2o**

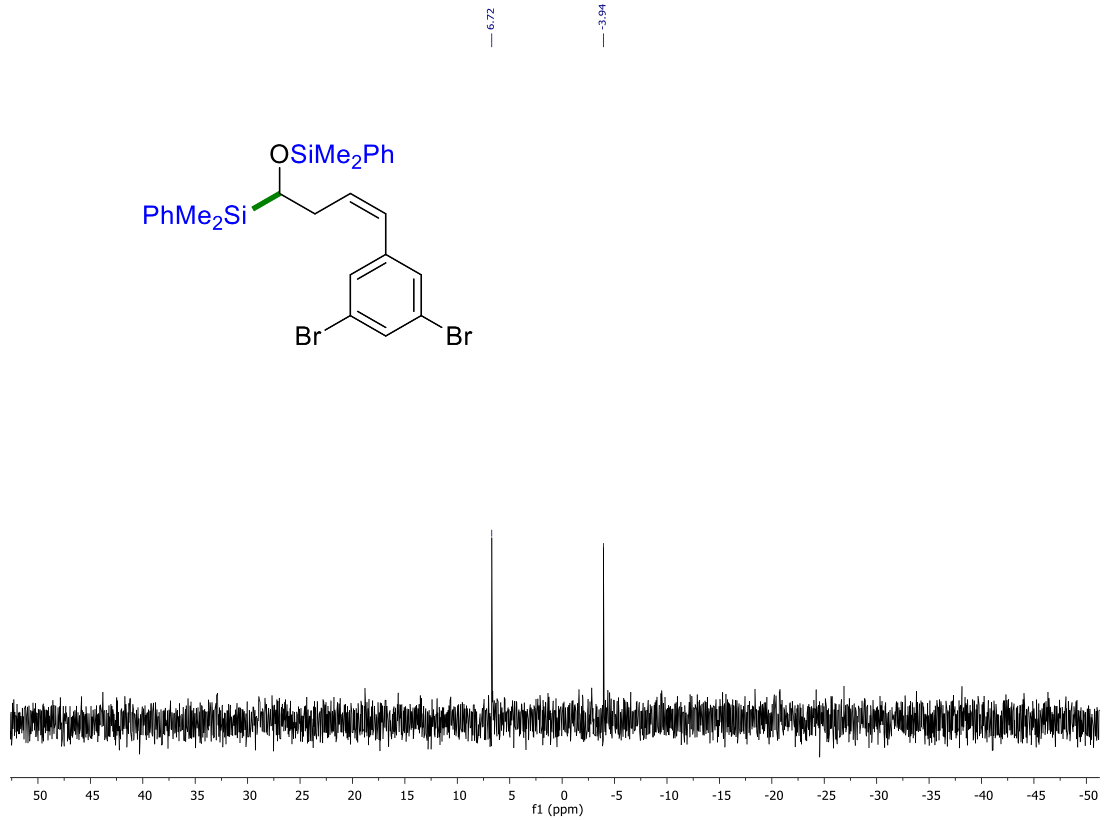

Supplementary Figure 76. ^{1}H-NMR spectrum of *Z*-**2p**

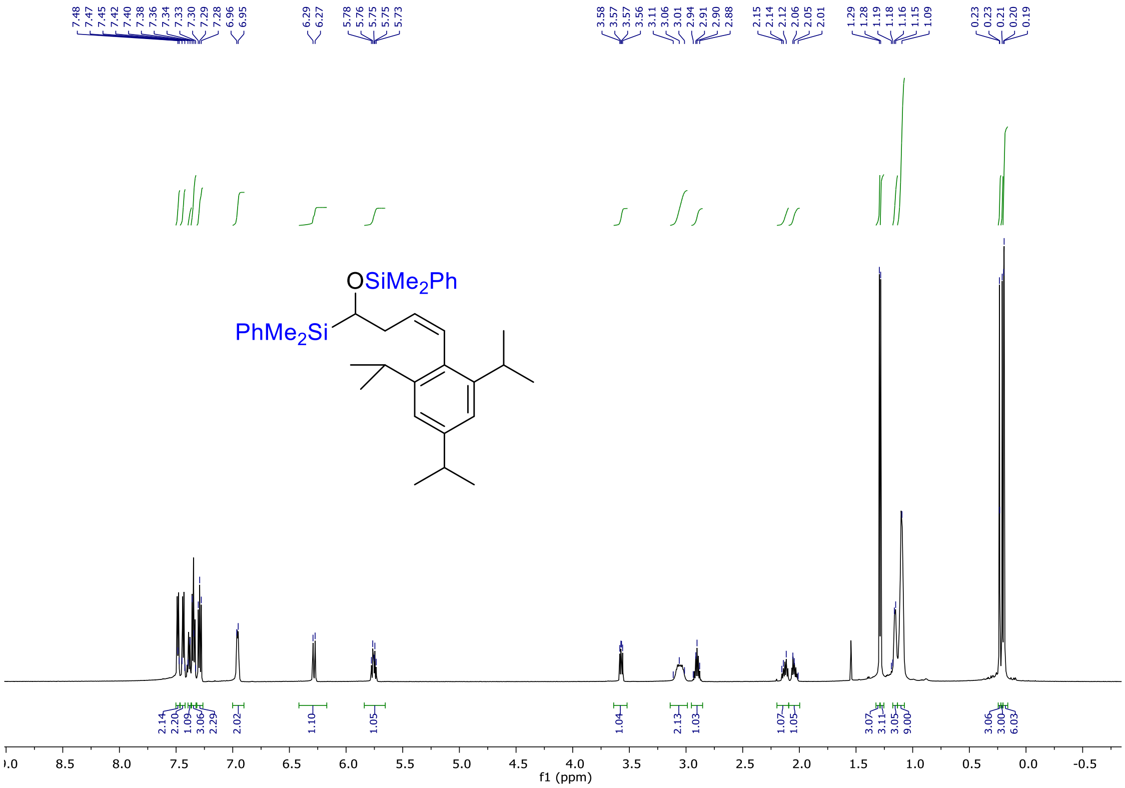

Supplementary Figure 77. ^{13}C and ^{29}Si-NMR spectra of *Z*-**2p**

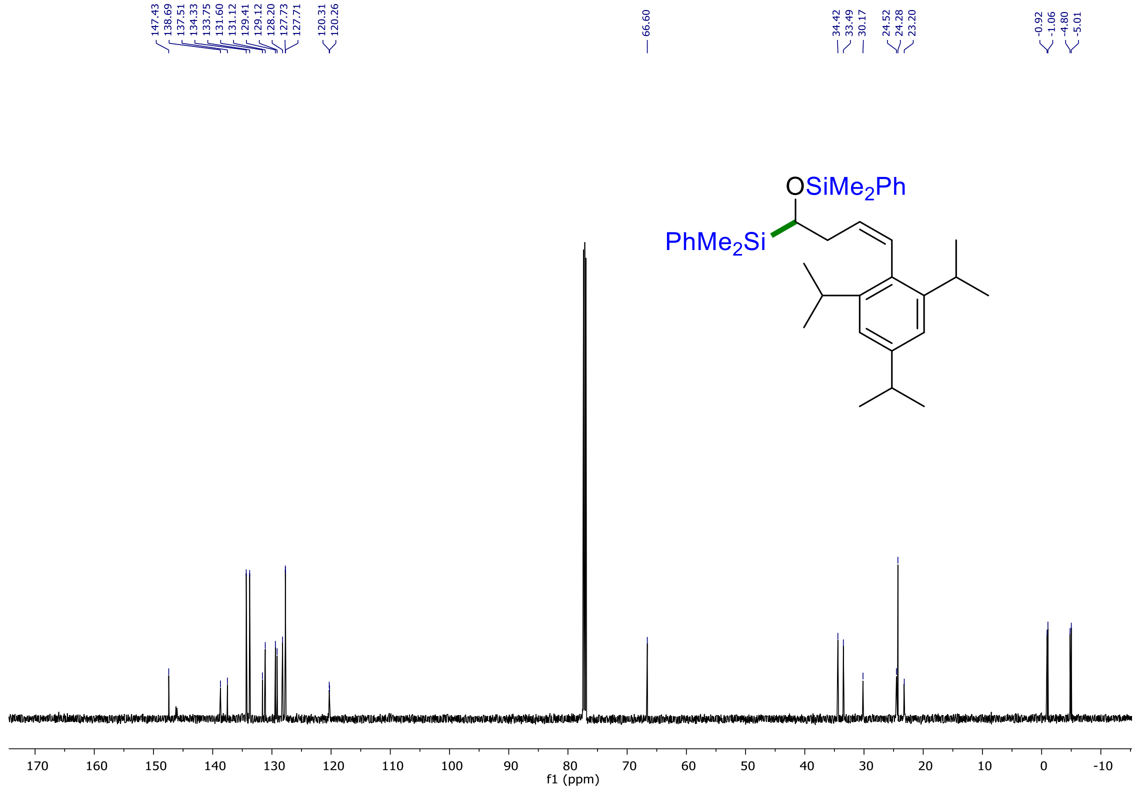

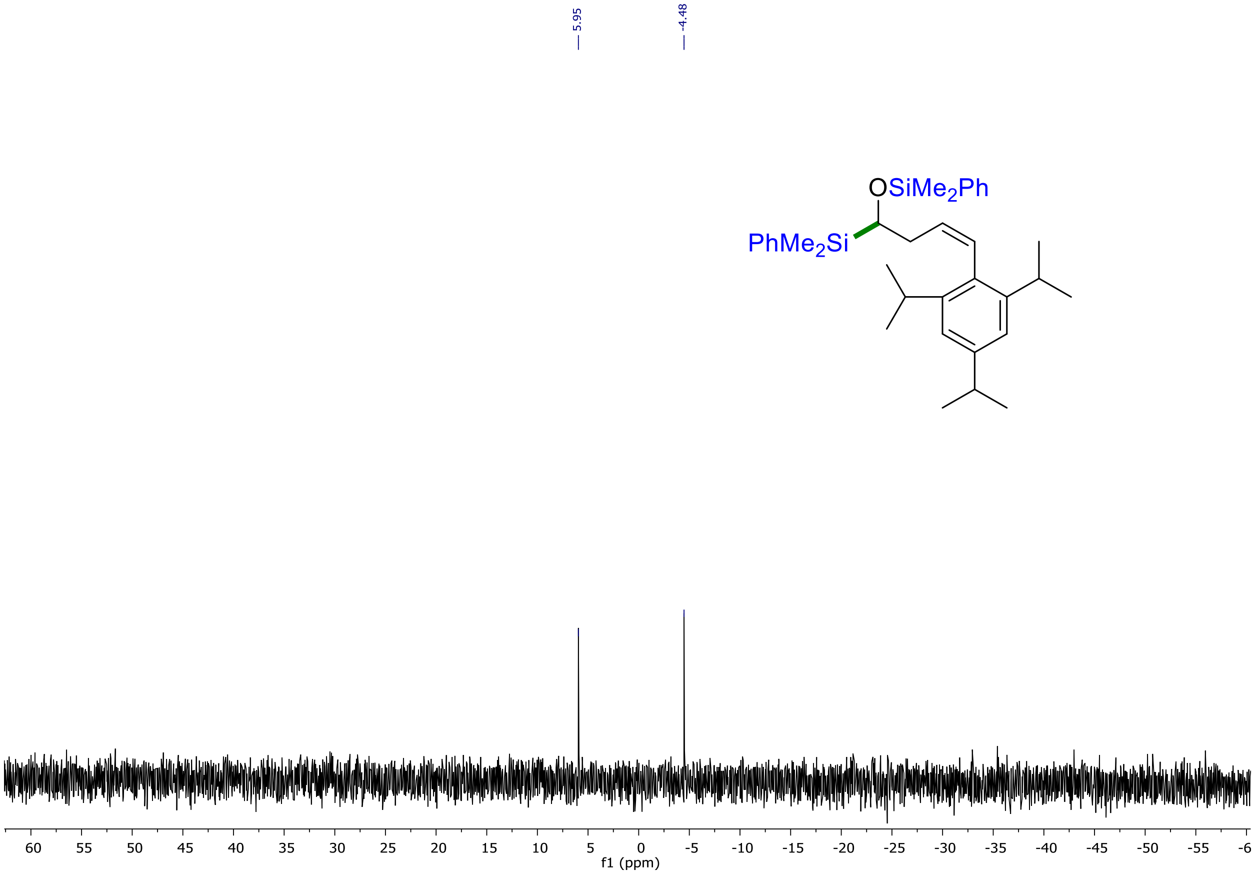

Supplementary Figure 78. ^{1}H and ^{13}C-NMR spectra of *Z*-**2q**

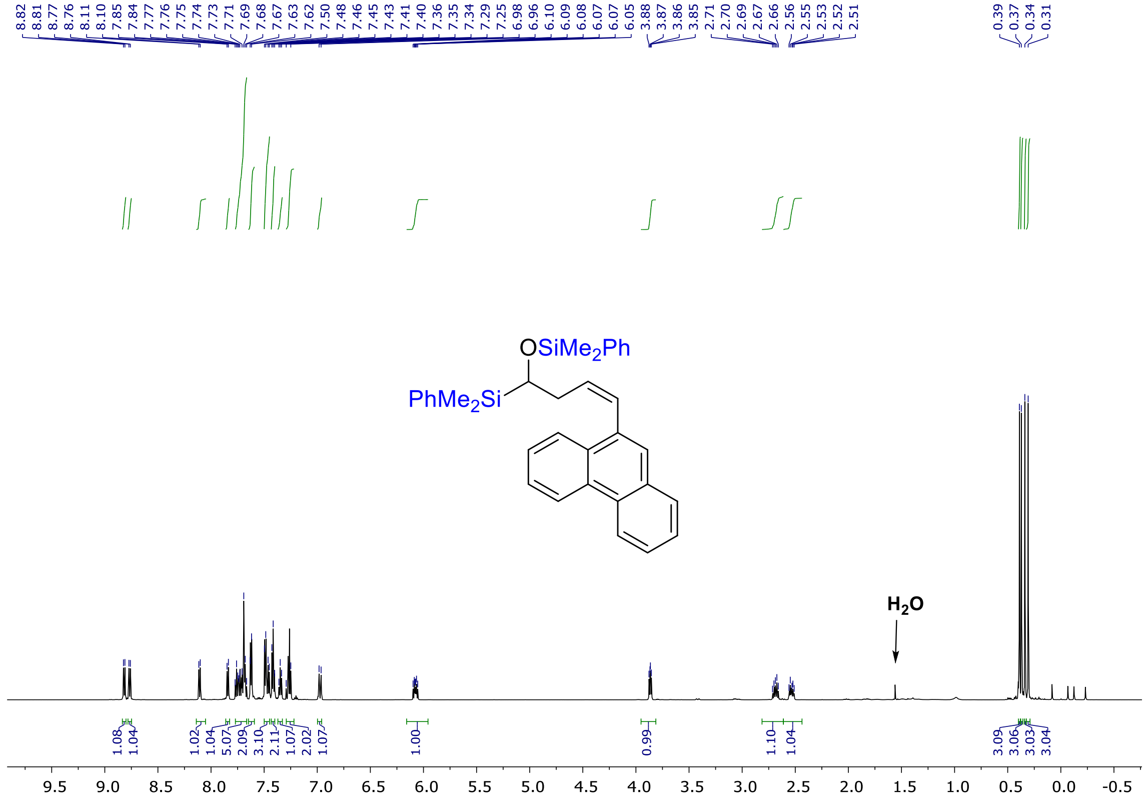

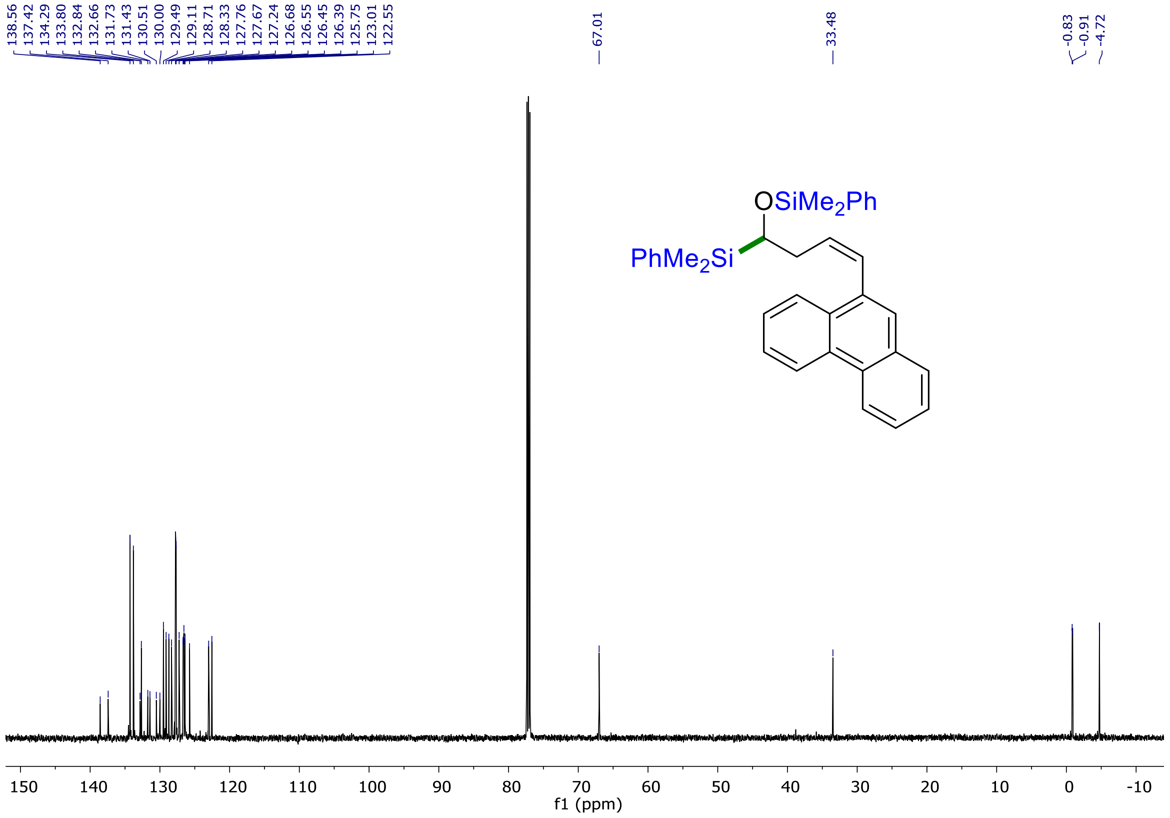

Supplementary Figure 79. ^{29}Si-NMR spectrum of *Z*-**2q**

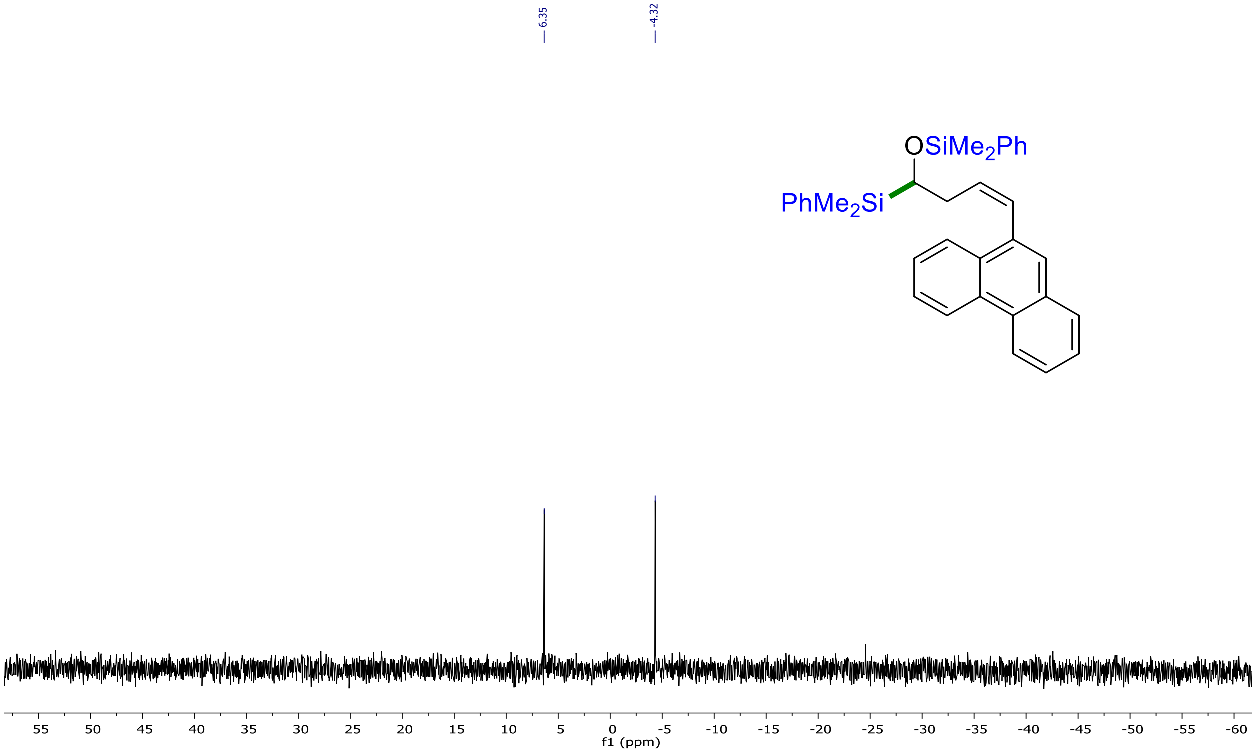

Supplementary Figure 80. ^{1}H-NMR spectrum of *Z*-**2r**

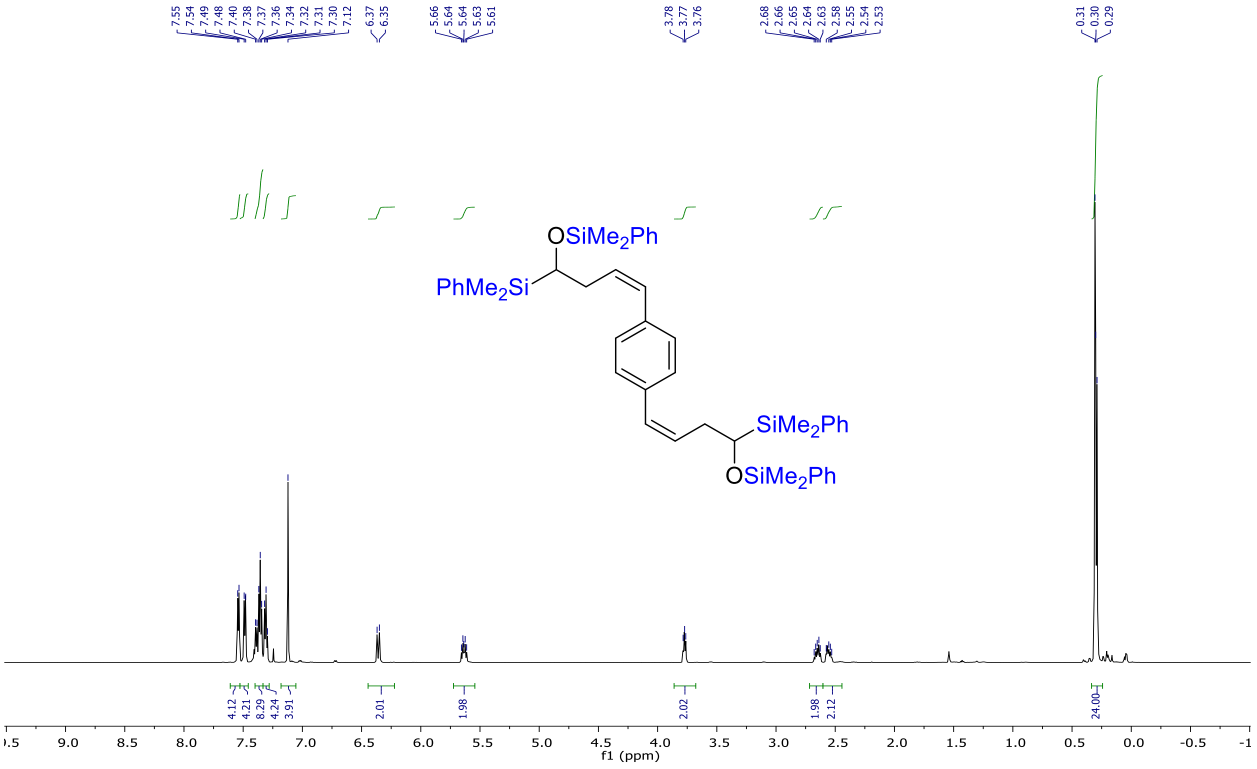

Supplementary Figure 81. ^{13}C and ^{29}Si-NMR spectra of *Z*-**2r**

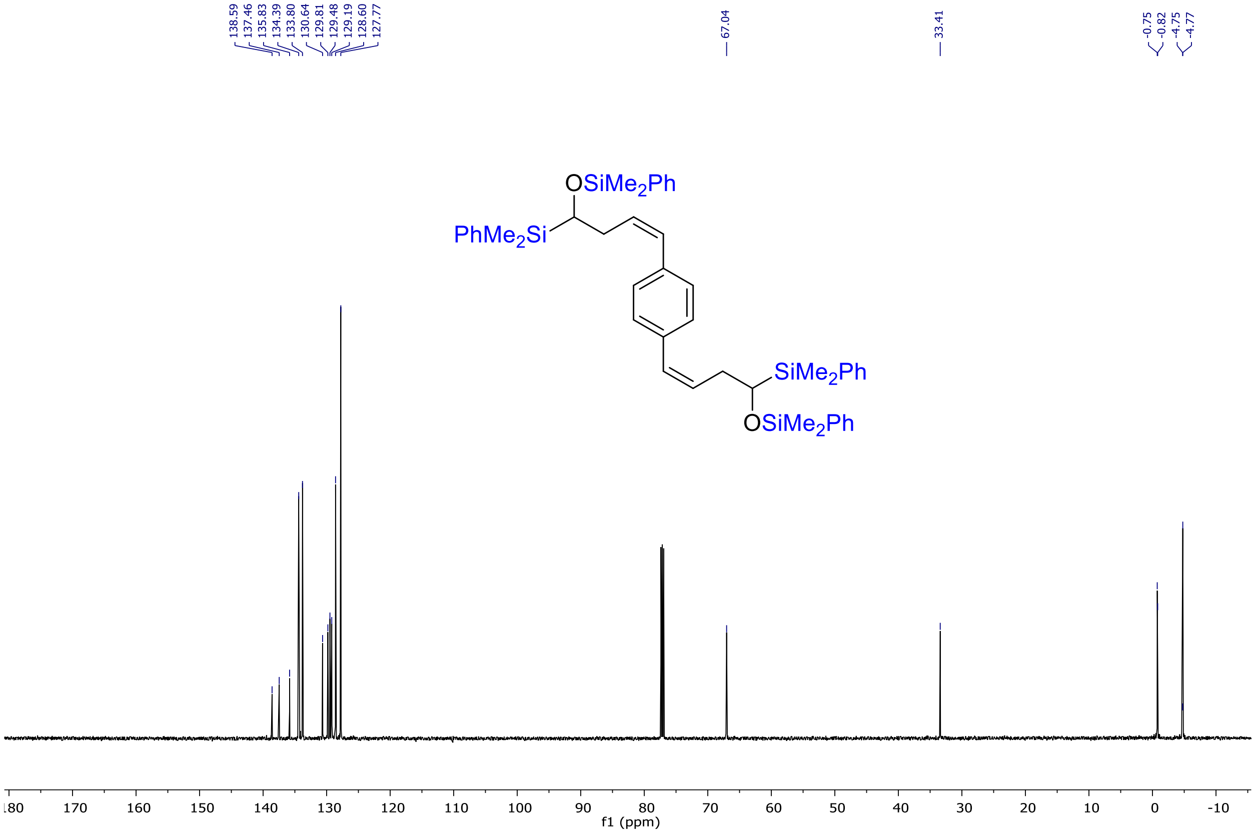

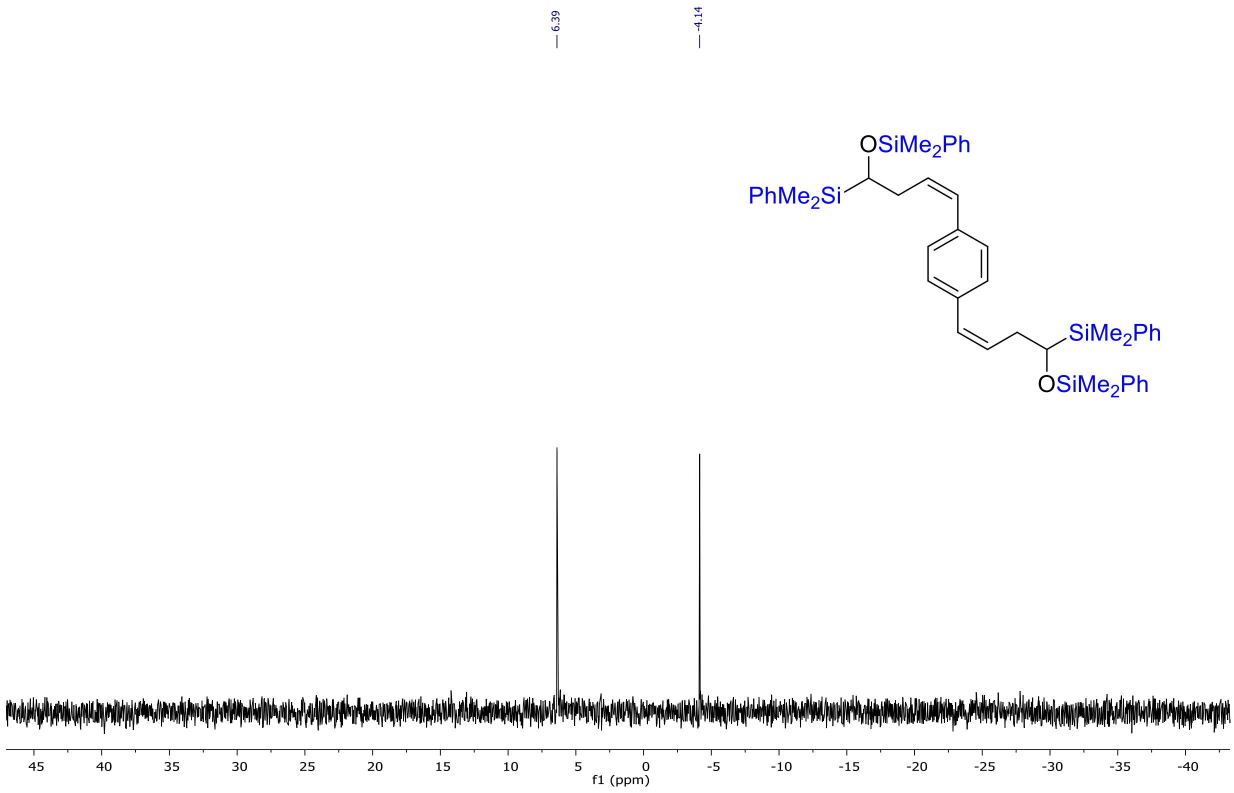

Supplementary Figure 82. ^{1}H and ^{13}C-NMR spectra of *Z*-**2s**

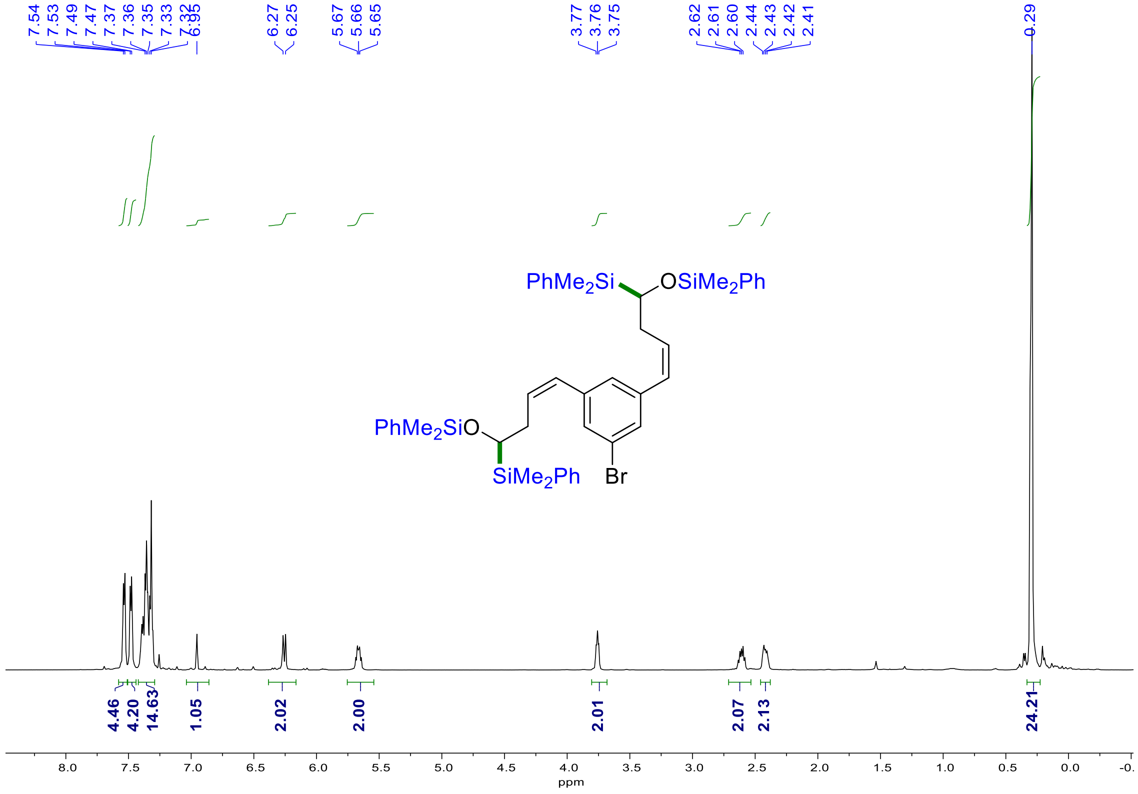

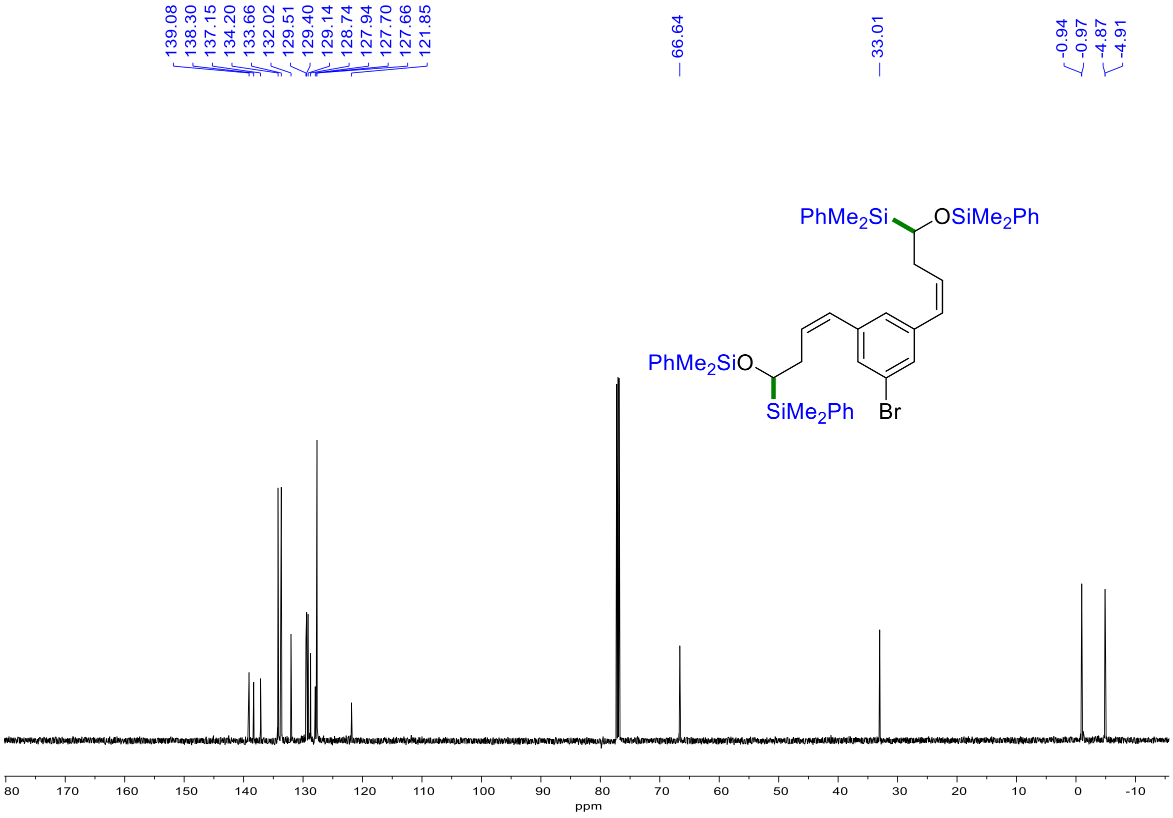

Supplementary Figure 83. ^{29}Si-NMR spectrum of *Z*-**2s**

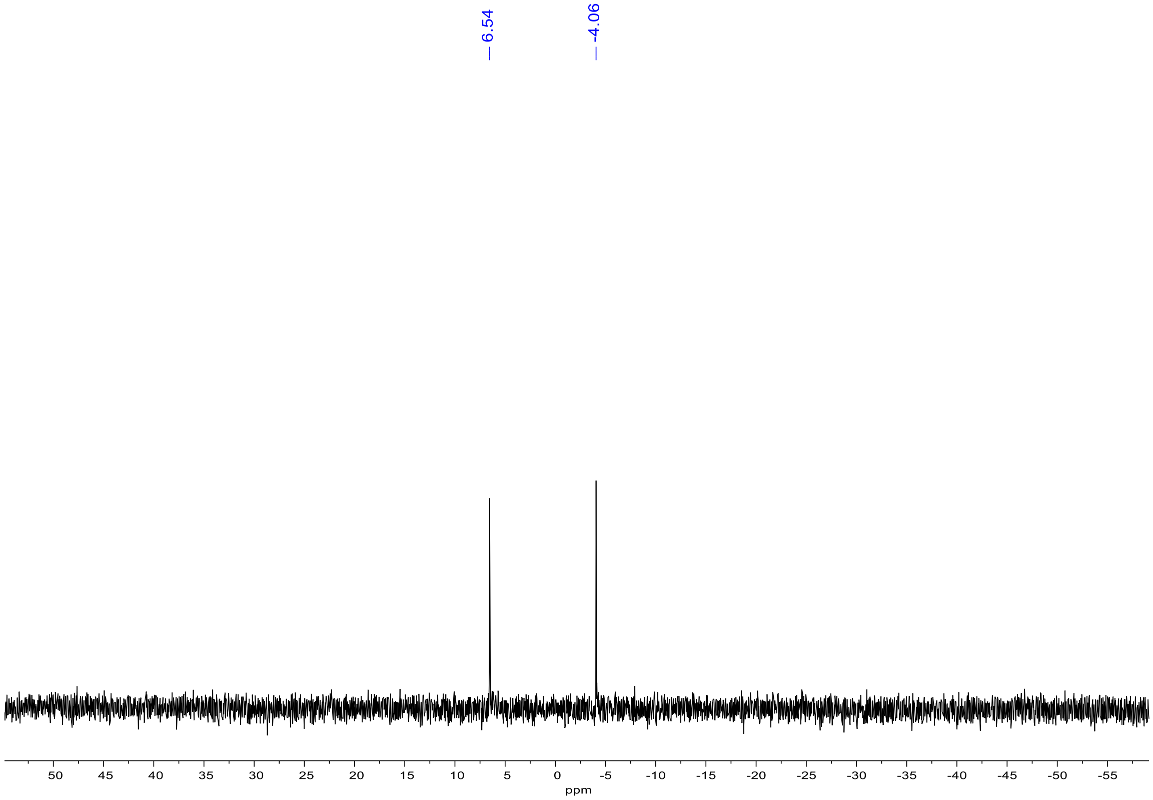

Supplementary Figure 84. ^{1}H-NMR spectrum of *Z*-**2t**

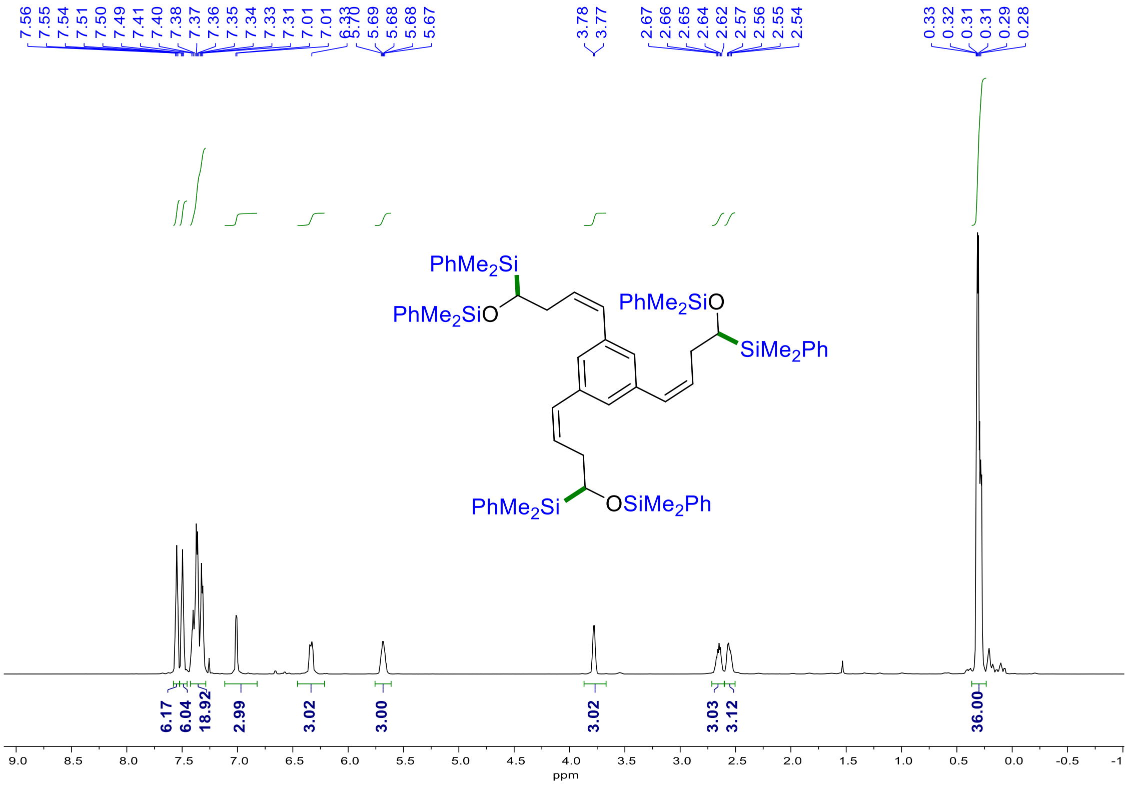

Supplementary Figure 85. ^{13}C and ^{29}Si-NMR spectra of *Z*-**2t**

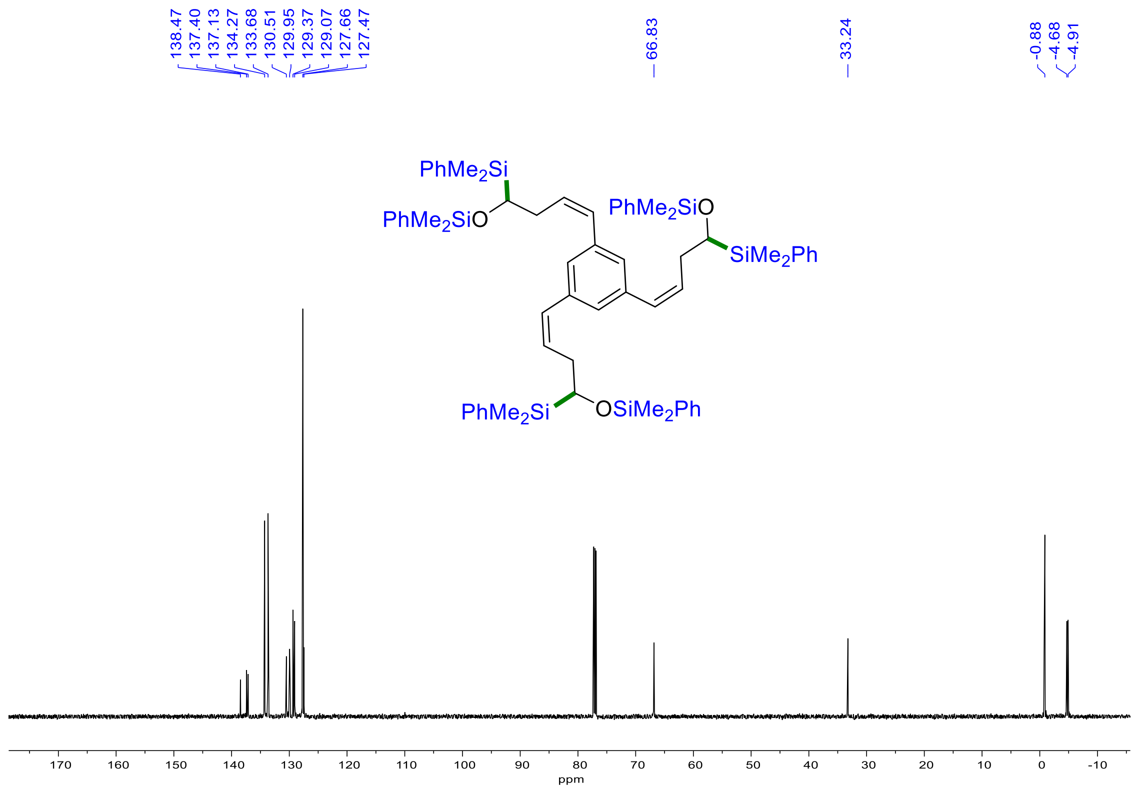

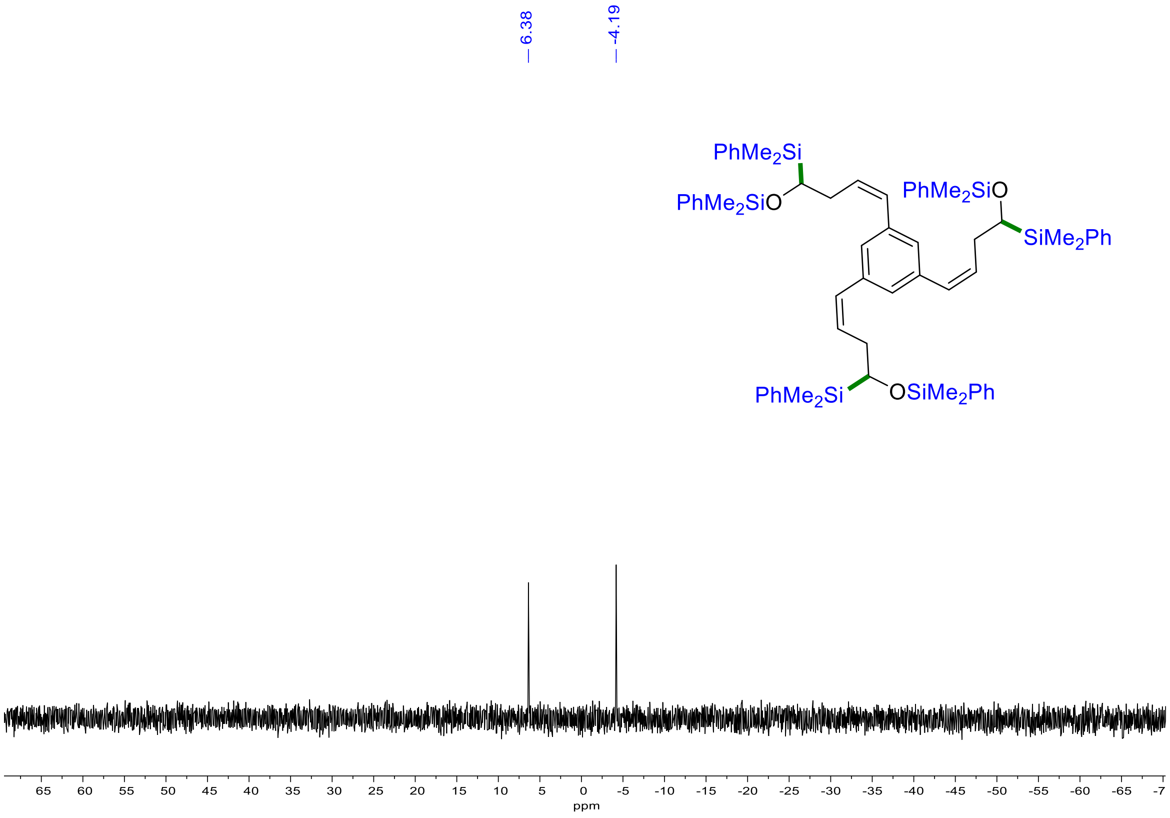

Supplementary Figure 86. ^{1}H and ^{13}C-NMR spectra of ***anti*-3a**

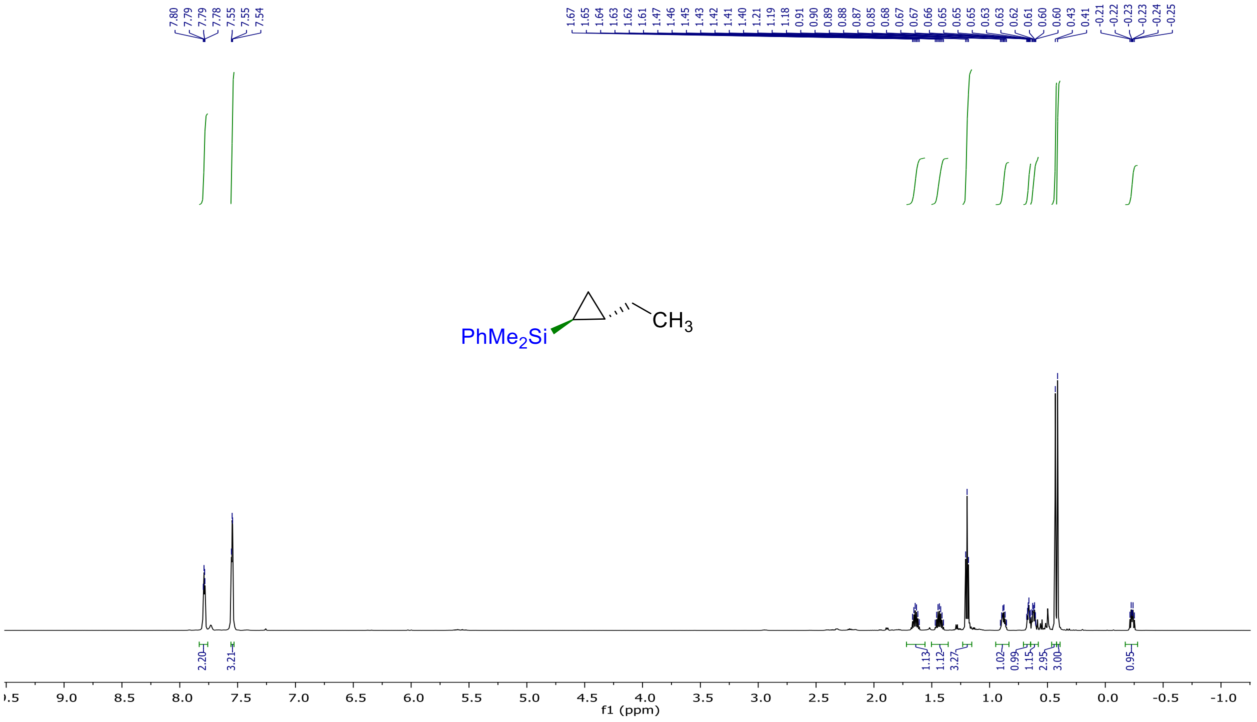

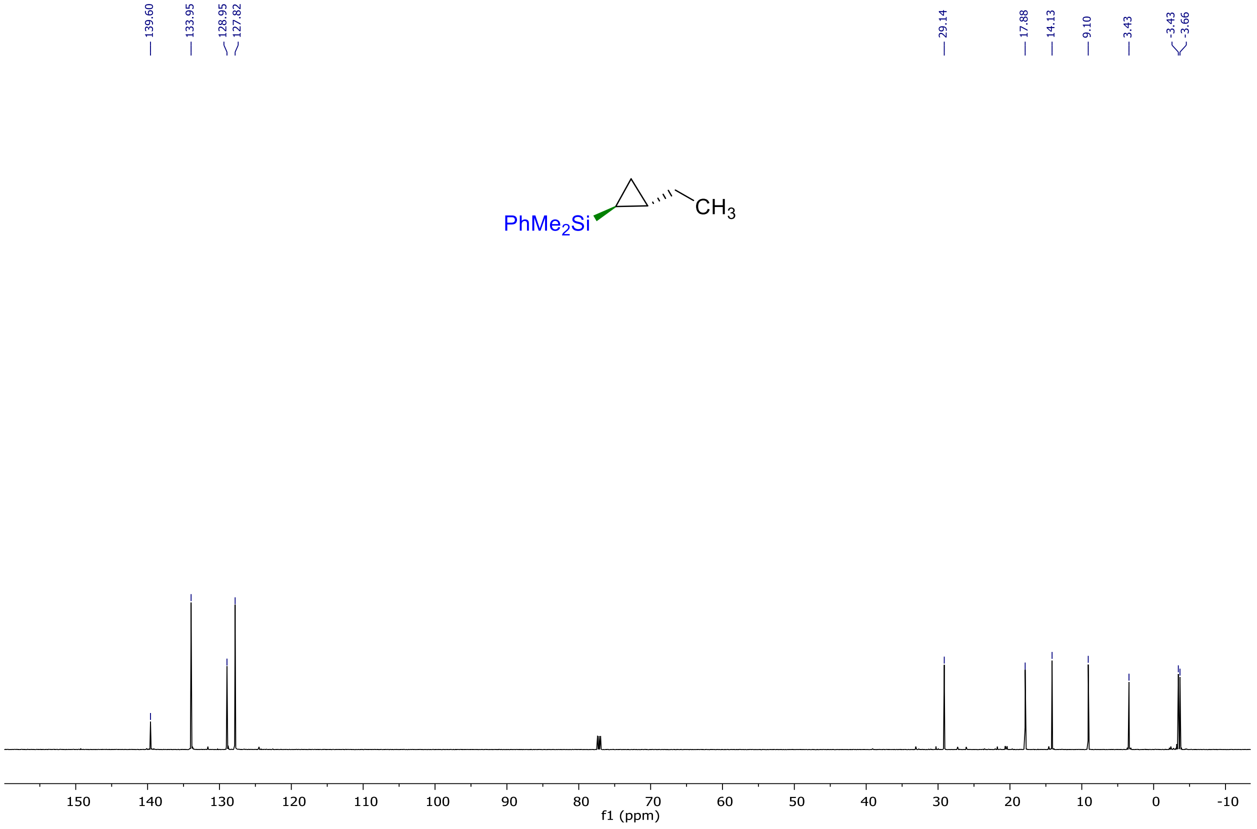

Supplementary Figure 87. ^{29}Si-NMR spectrum of ***anti*-3a**

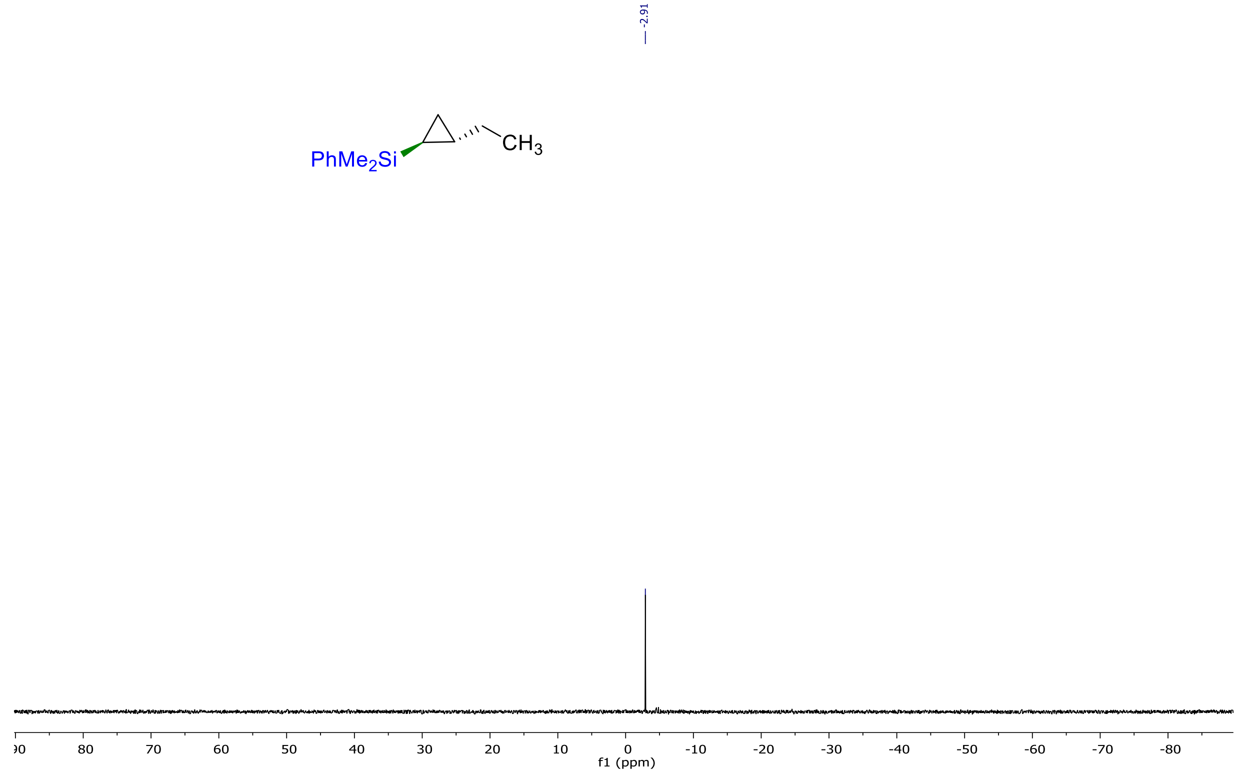

Supplementary Figure 88. ^{1}H-NMR spectrum of ***anti*-3a-*d***

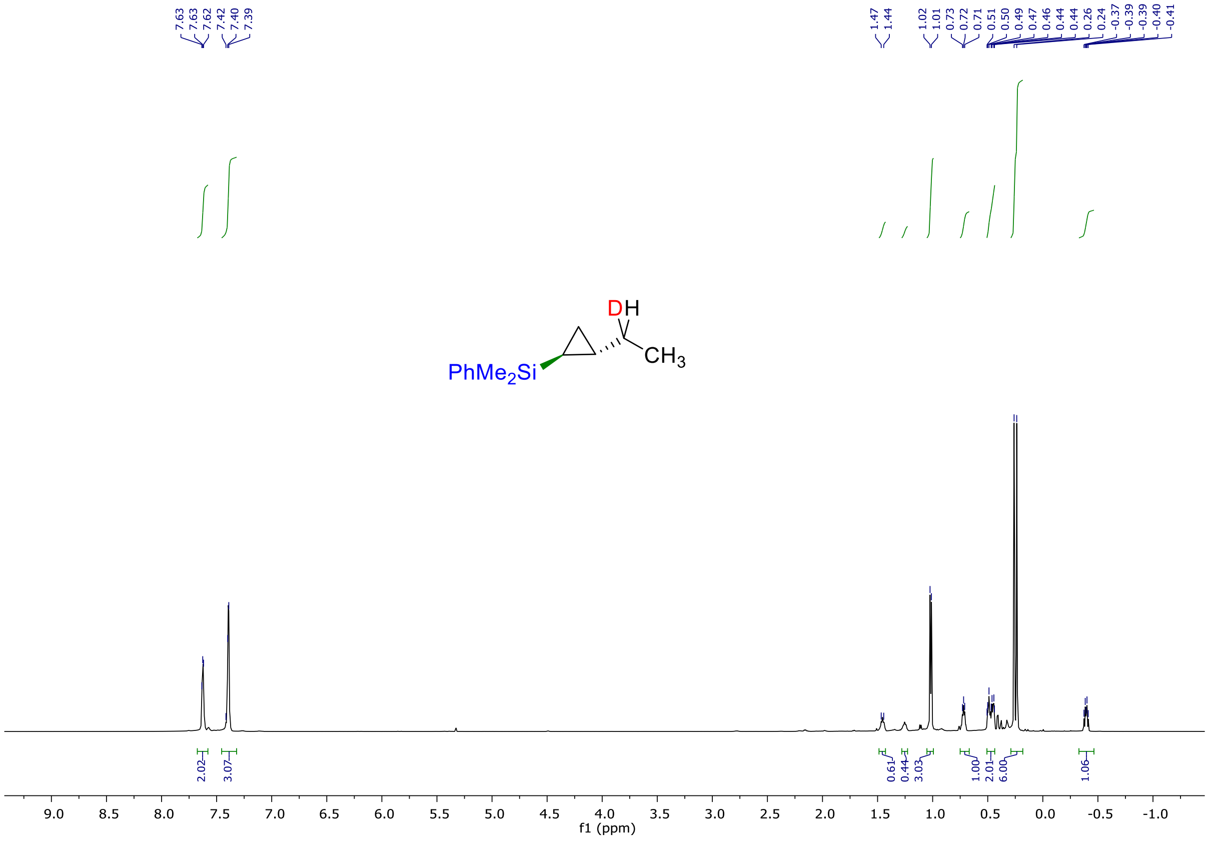

Supplementary Figure 89. ^{13}C and ^{29}Si-NMR spectra of ***anti*-3a-*d***

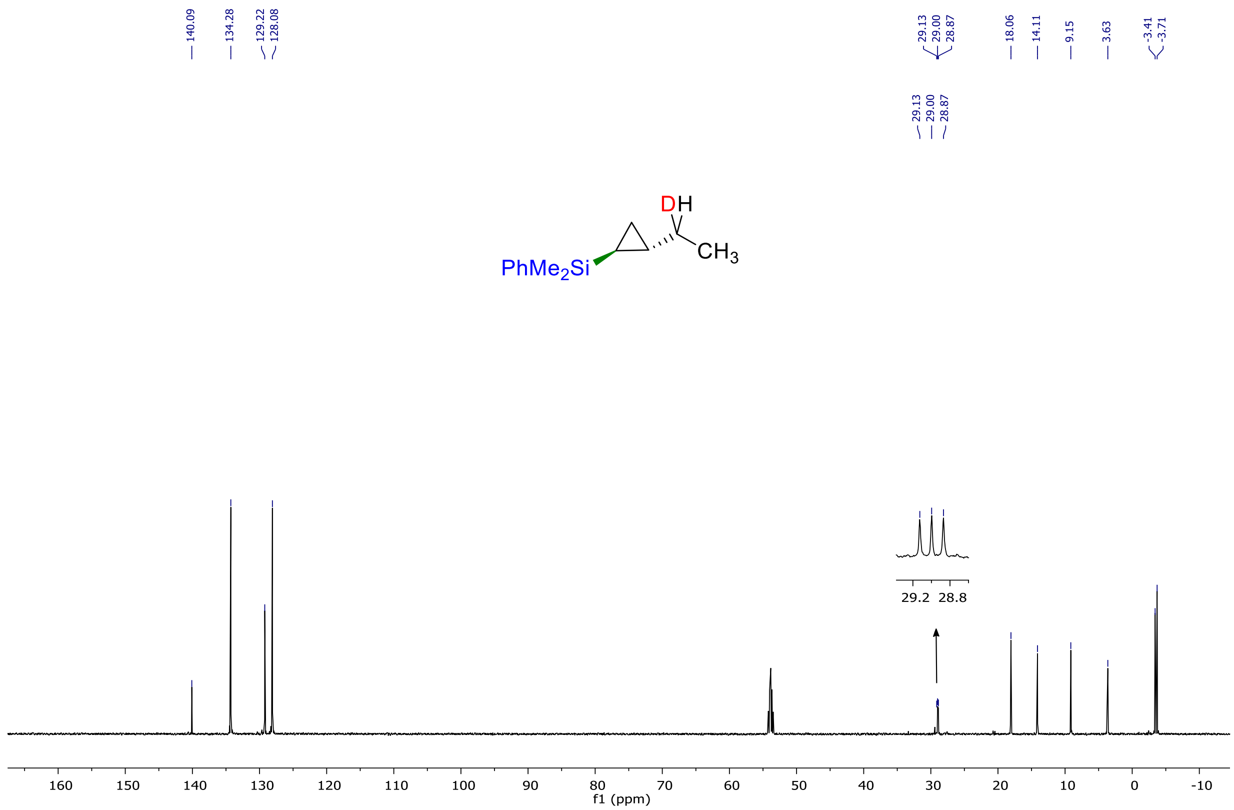

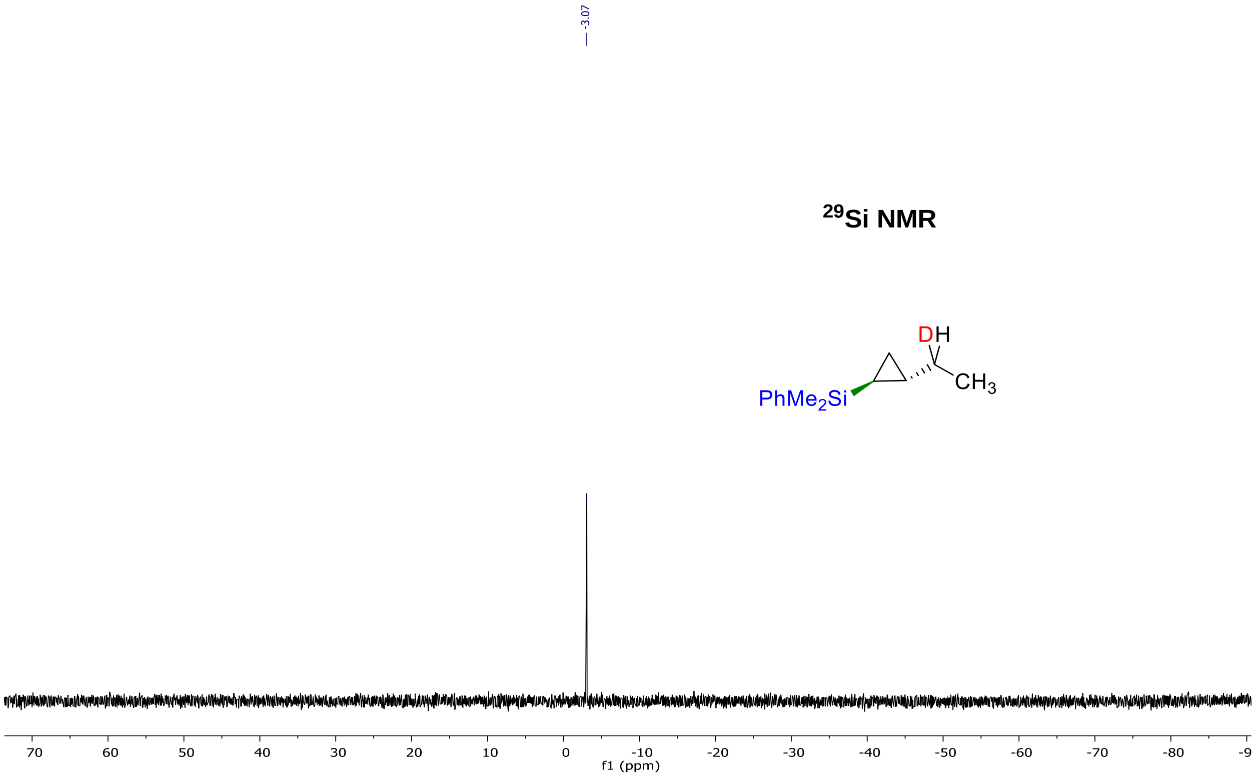

Supplementary Figure 90. ^{2}H-NMR spectrum of ***anti*-3a-*d***

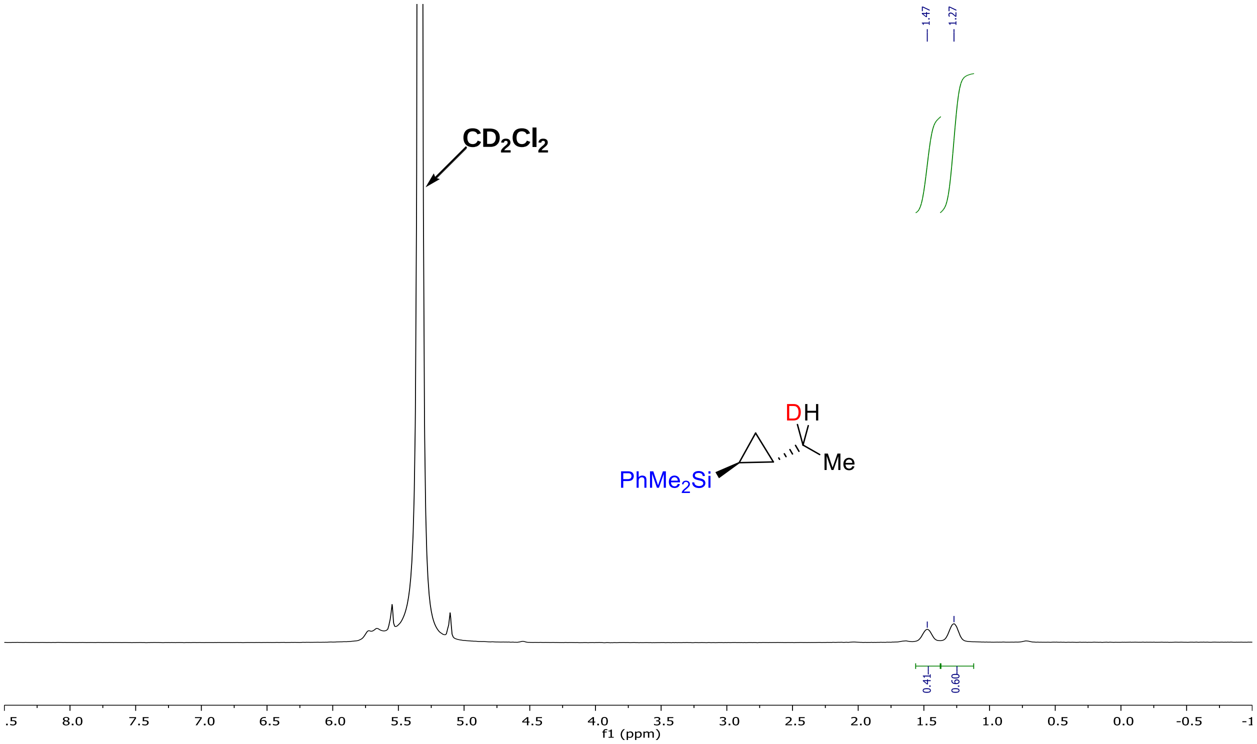

Supplementary Figure 91. ^{1}H-NMR spectrum of ***anti*-3b**

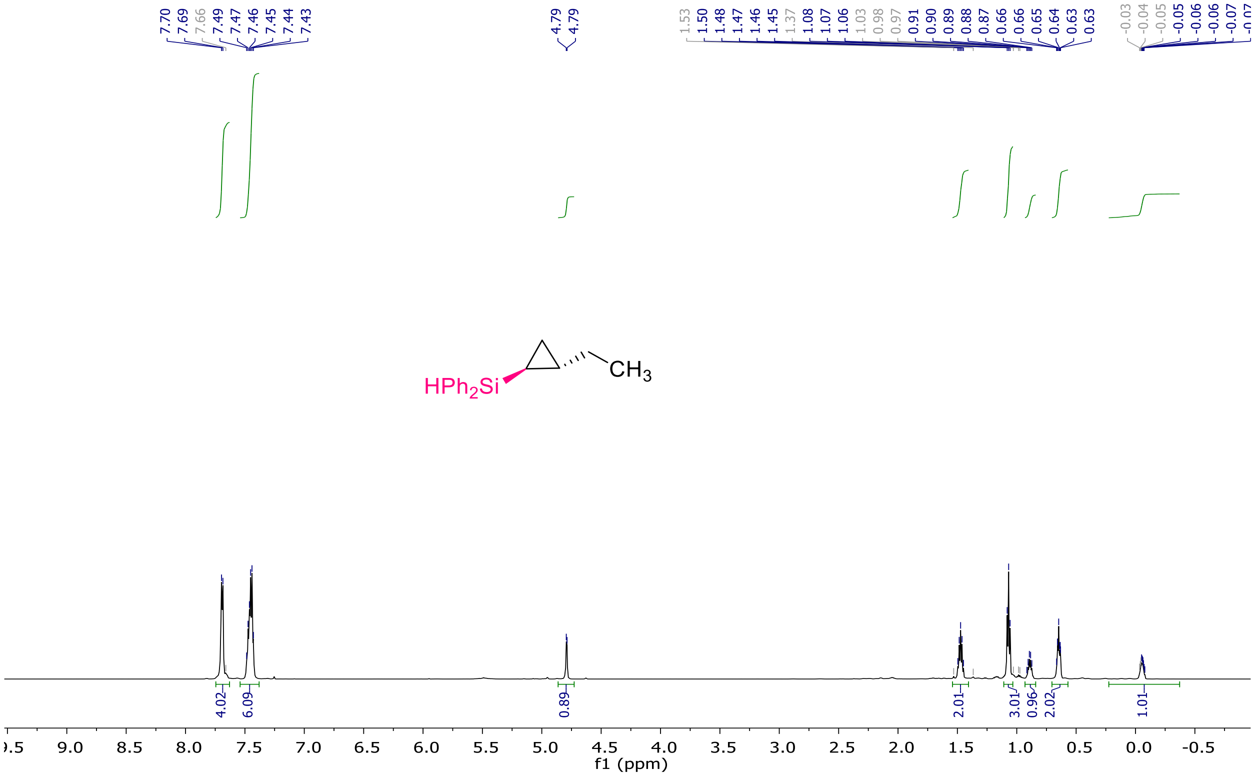

Supplementary Figure 92. ^{13}C and ^{29}Si-NMR spectra of ***anti*-3b**

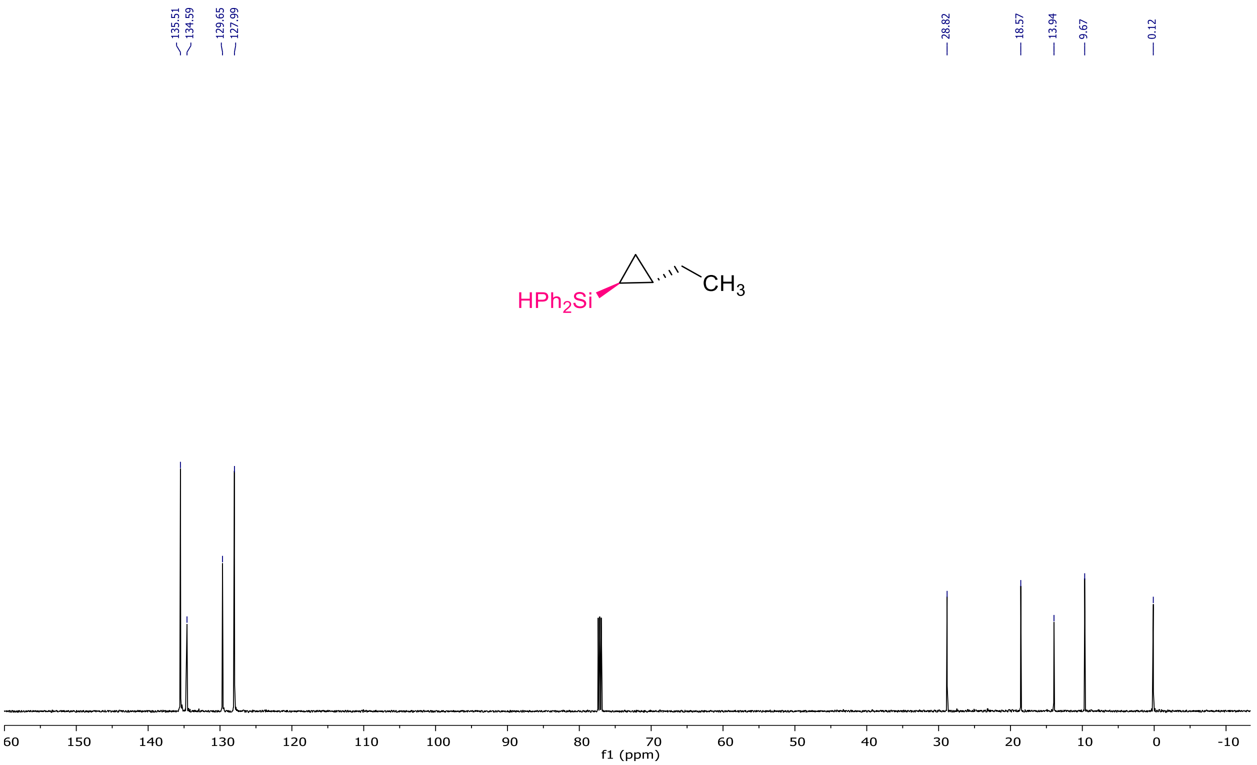

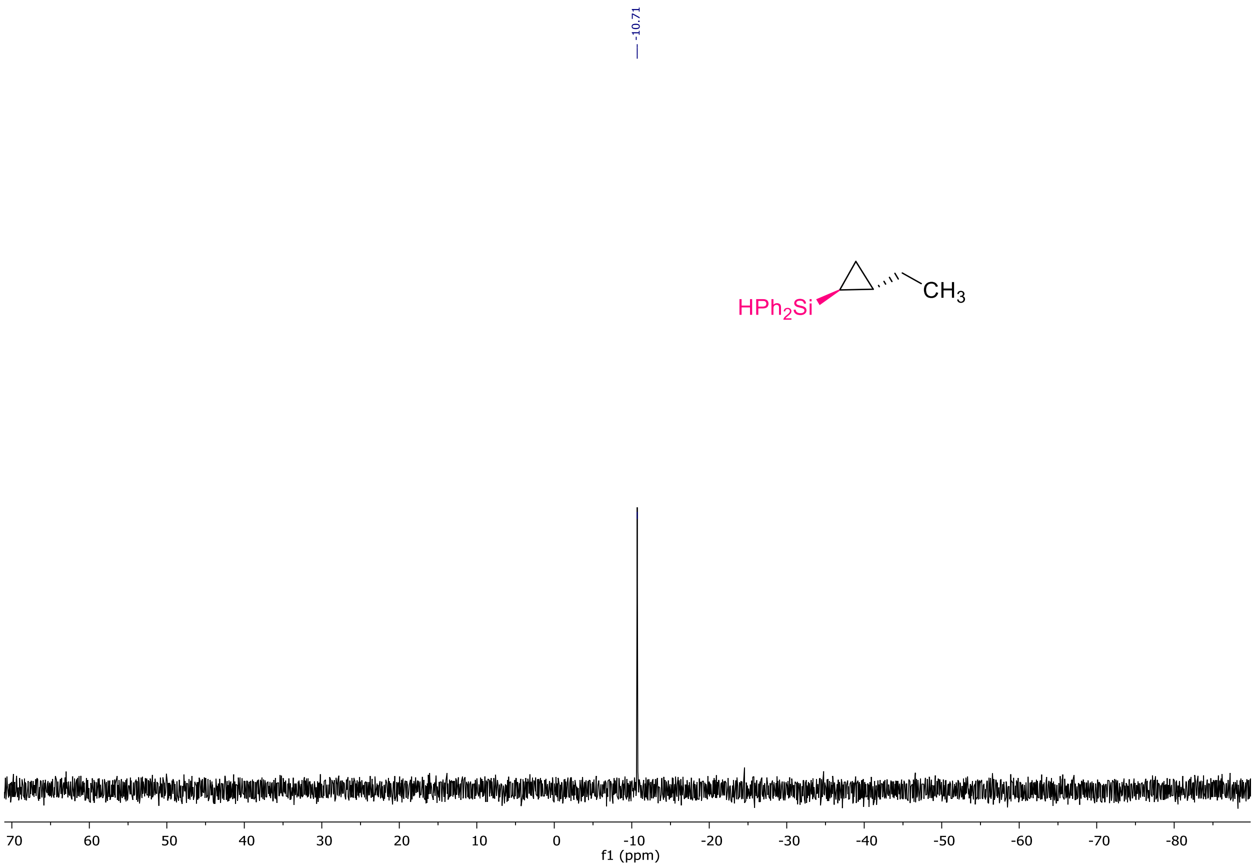

Supplementary Figure 93. ^{1}H and ^{13}C-NMR spectra of ***anti*-3c**

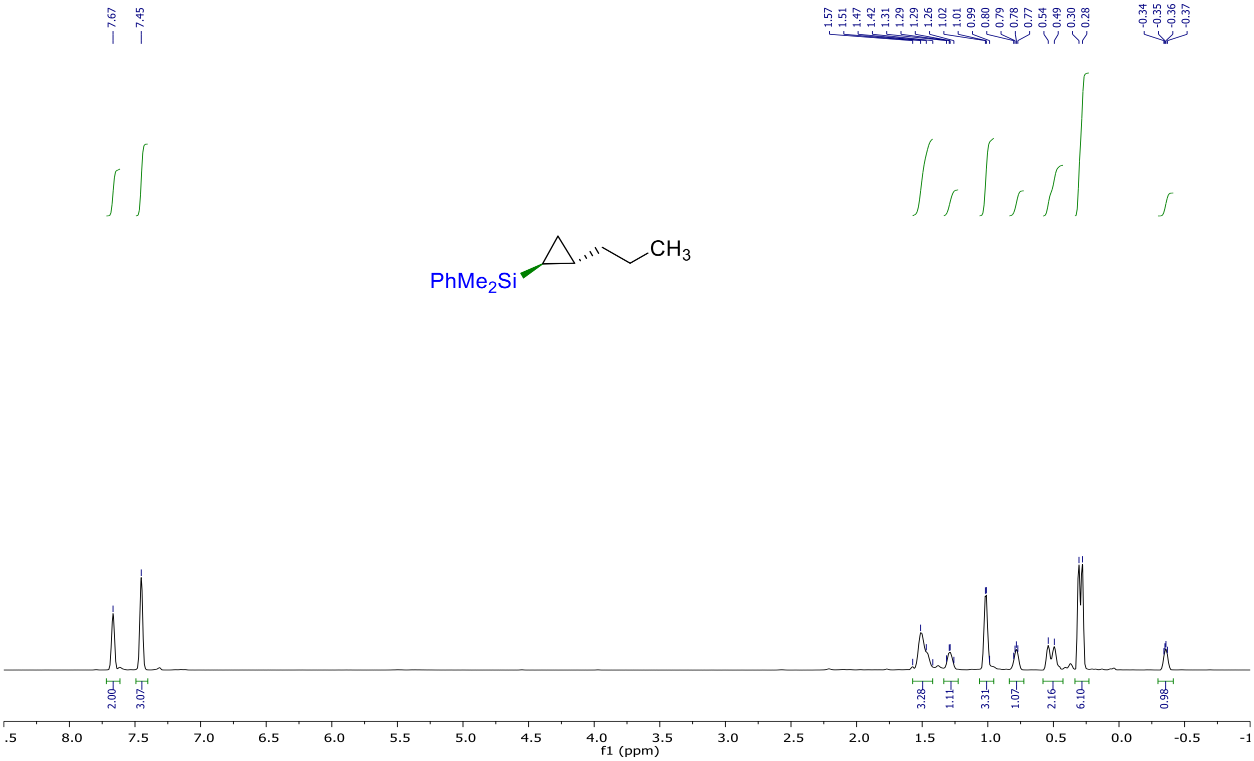

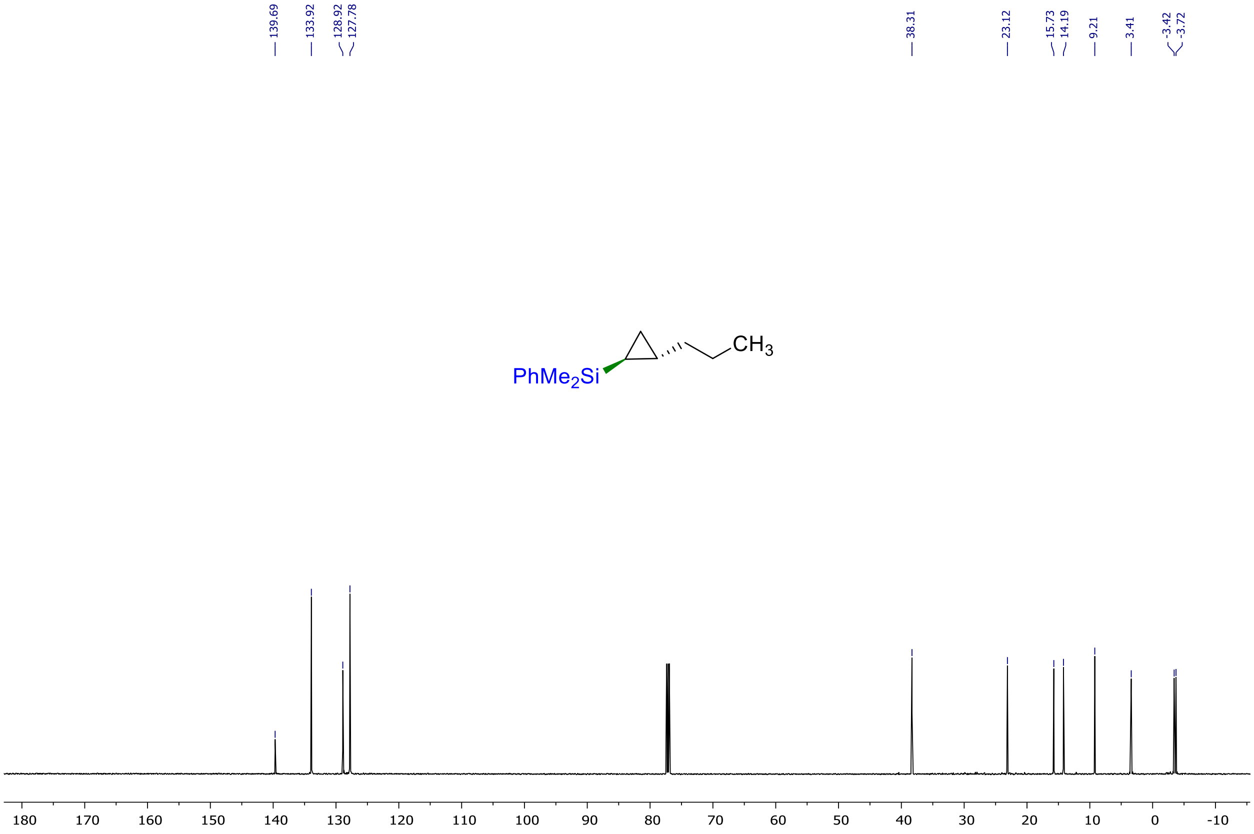

Supplementary Figure 94. ^{29}Si-NMR spectrum of ***anti*-3c**

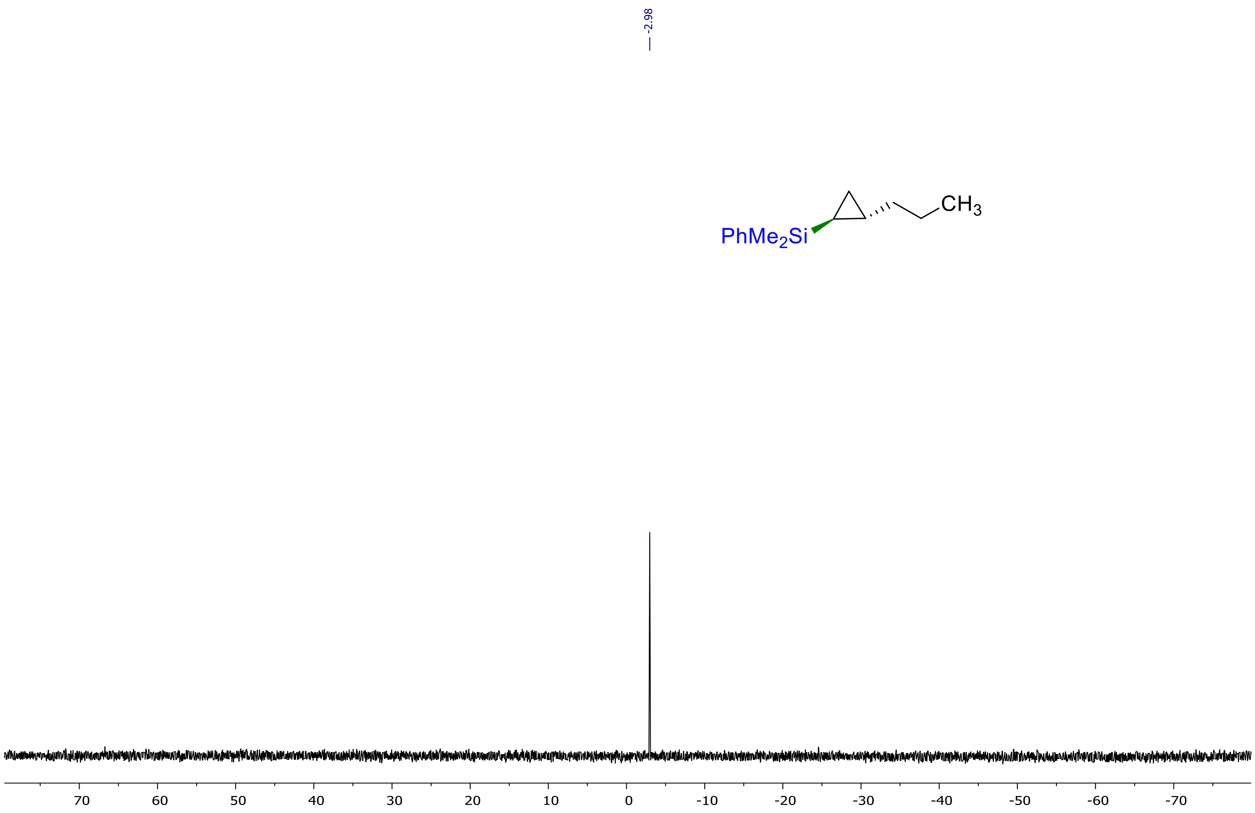

Supplementary Figure 95. ^{1}H-NMR spectrum of ***anti*-3d**

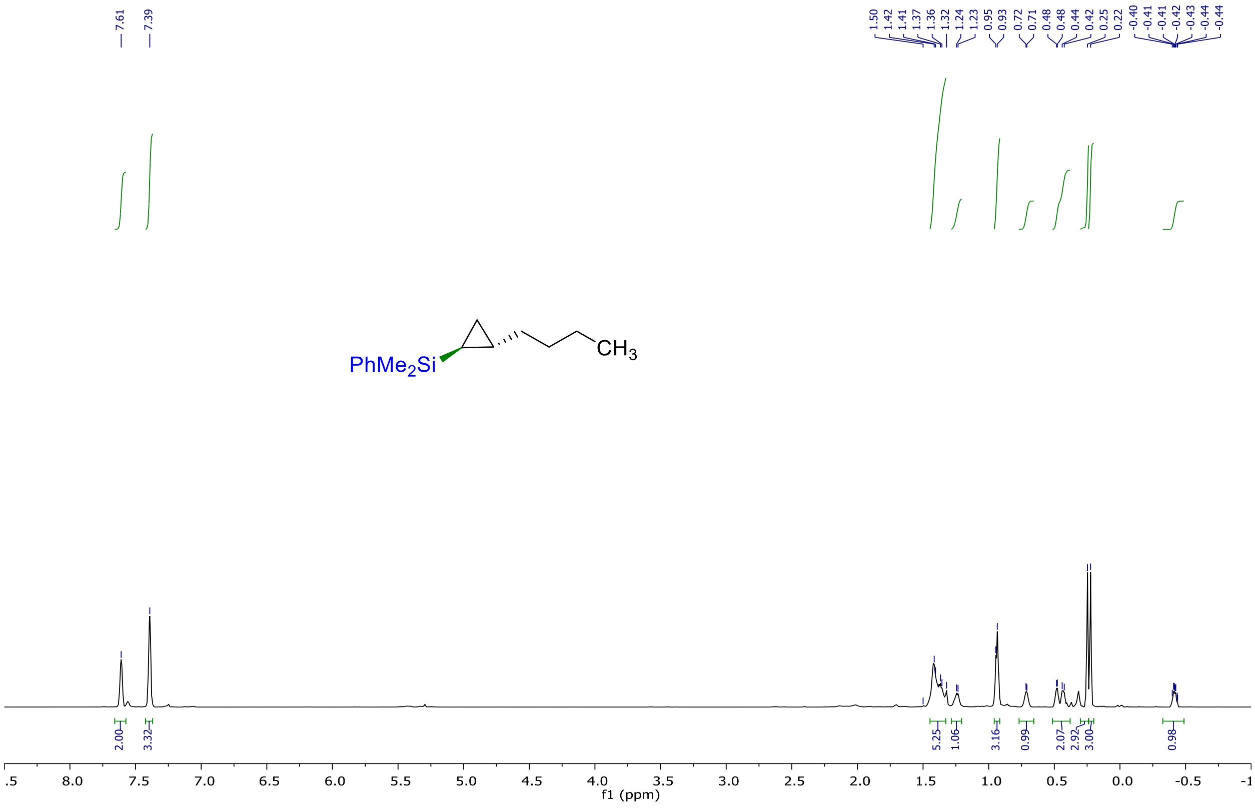

Supplementary Figure 96. ^{13}C and ^{29}Si-NMR spectra of ***anti*-3d**

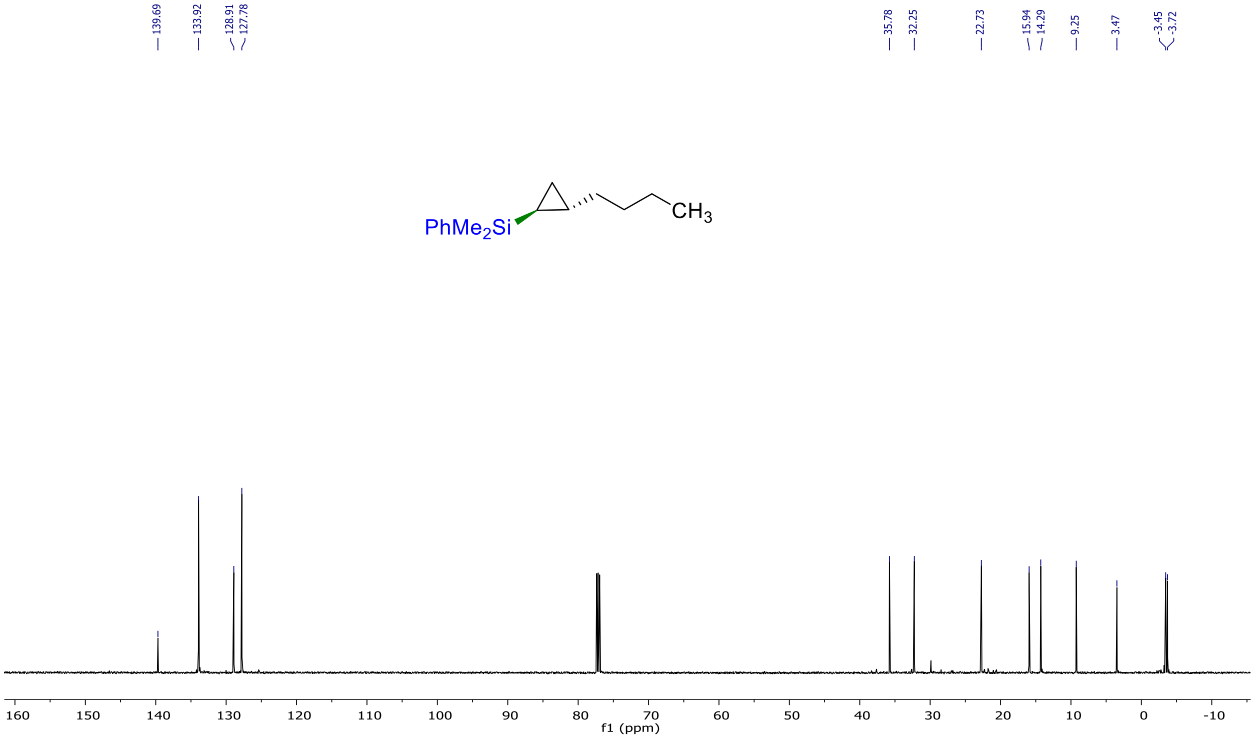

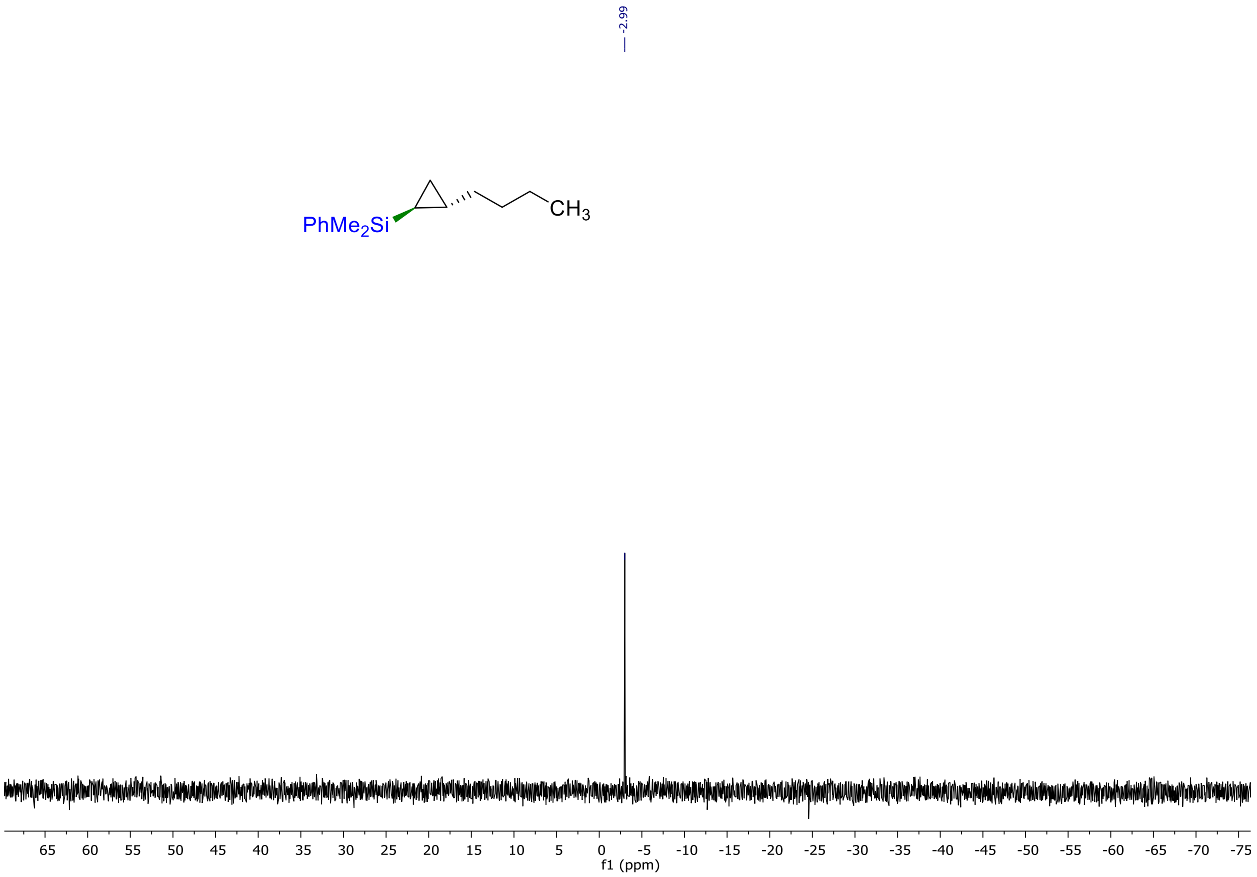

Supplementary Figure 97. ^{1}H and ^{13}C-NMR spectra of ***anti*-3e**

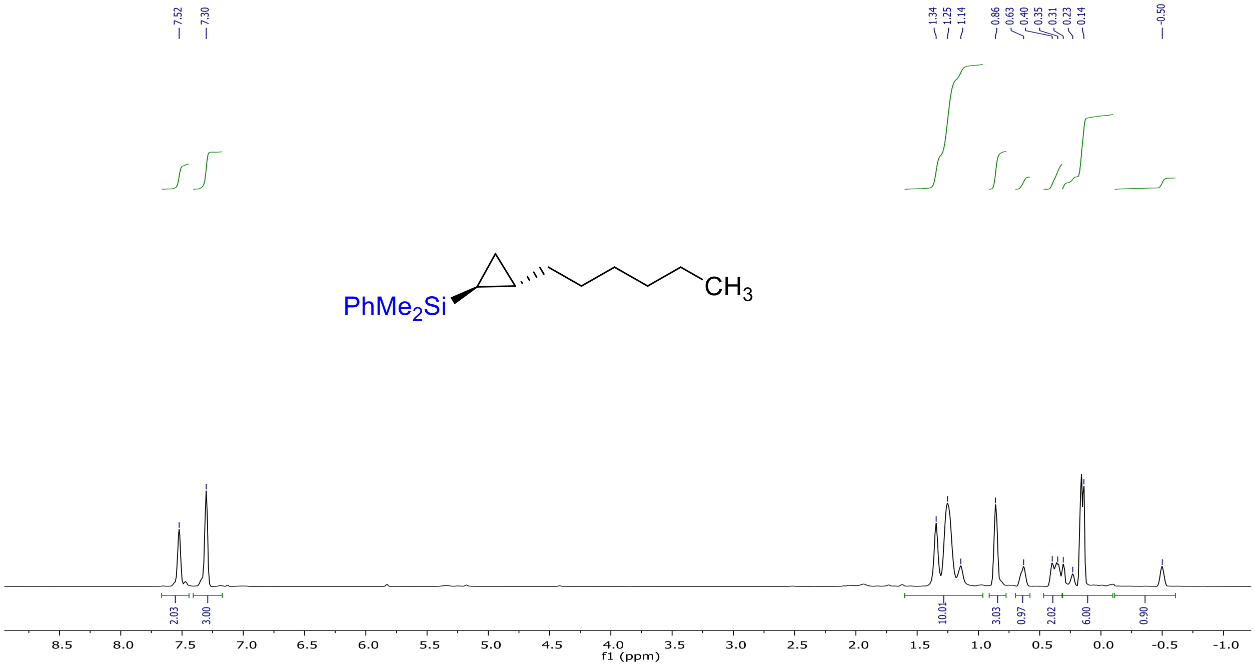

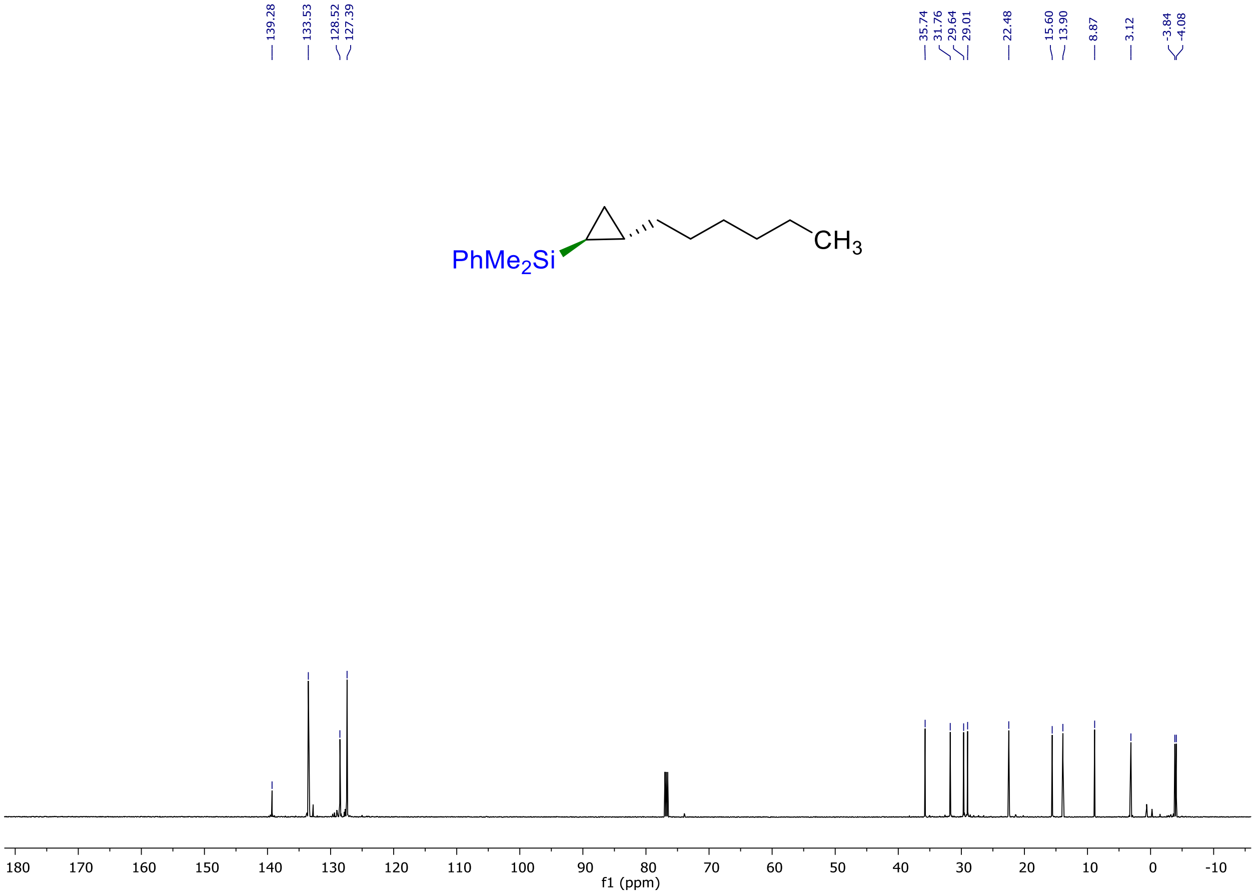

Supplementary Figure 98. ^{29}Si-NMR spectrum of ***anti*-3e**

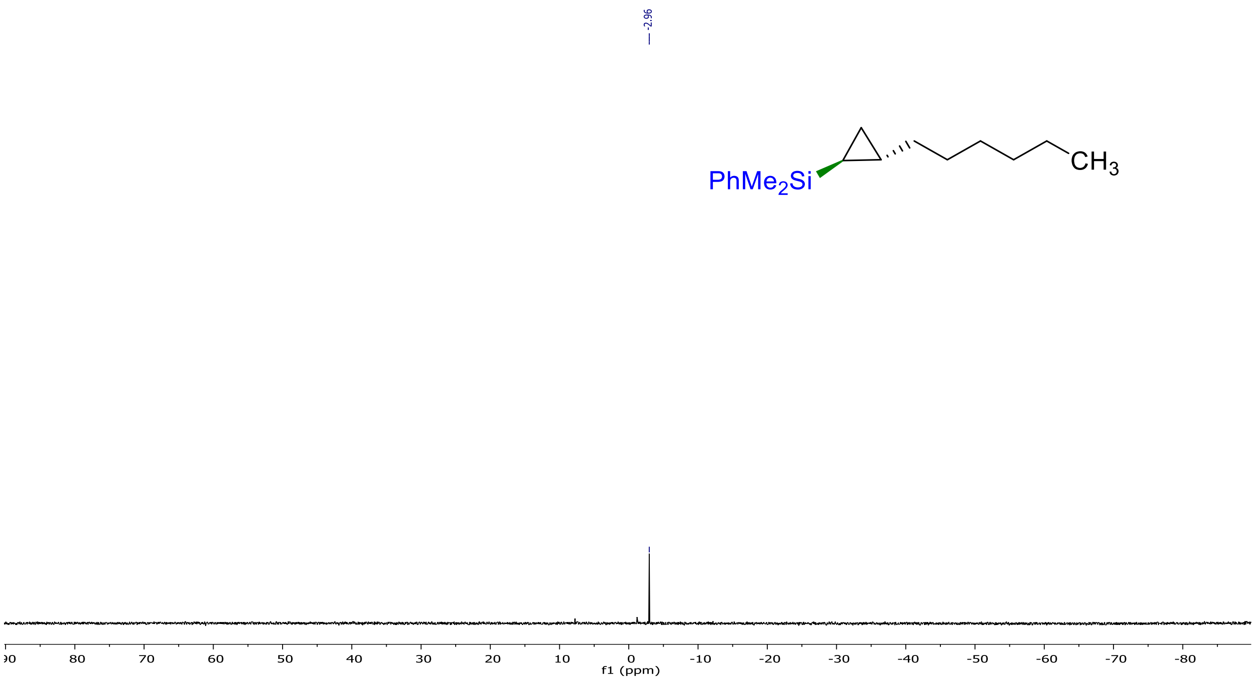

Supplementary Figure 99. ^{1}H-NMR spectrum of ***anti*-3f**

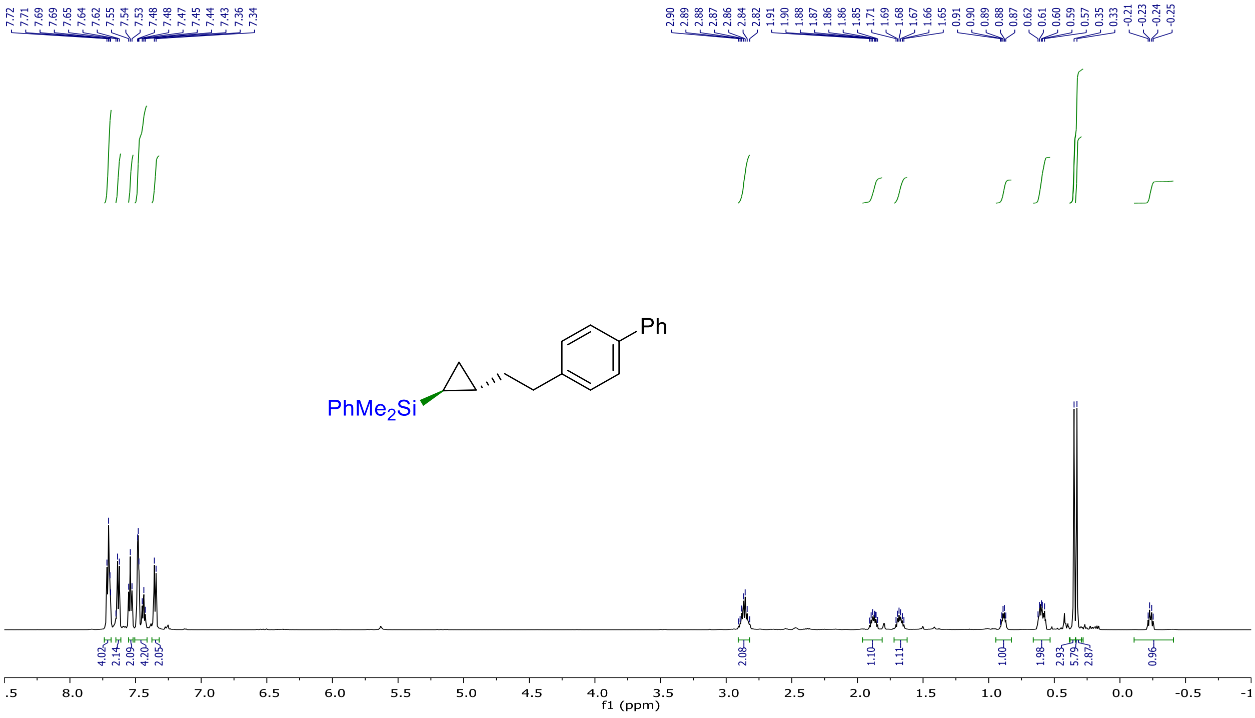

Supplementary Figure 100. ^{13}C and ^{29}Si-NMR spectra of ***anti*-3f**

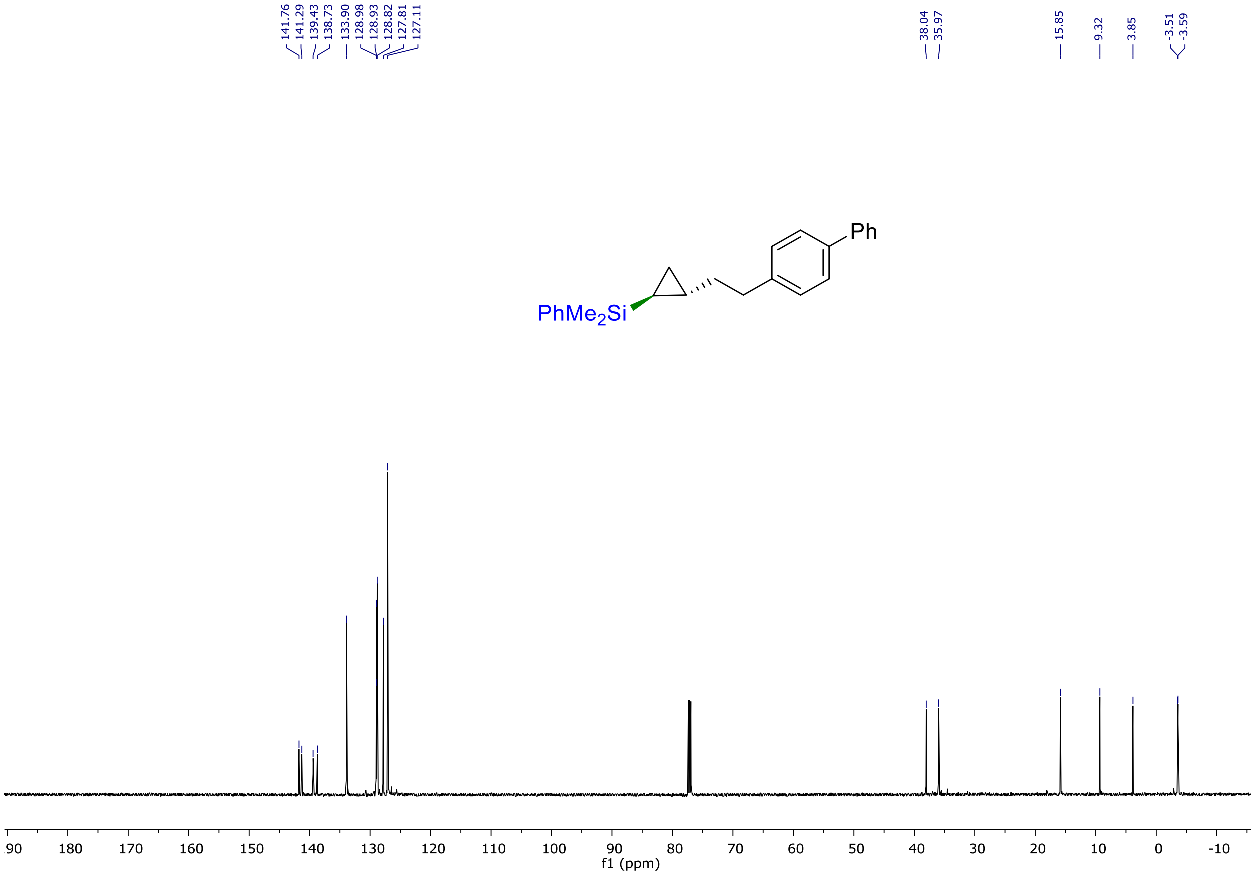

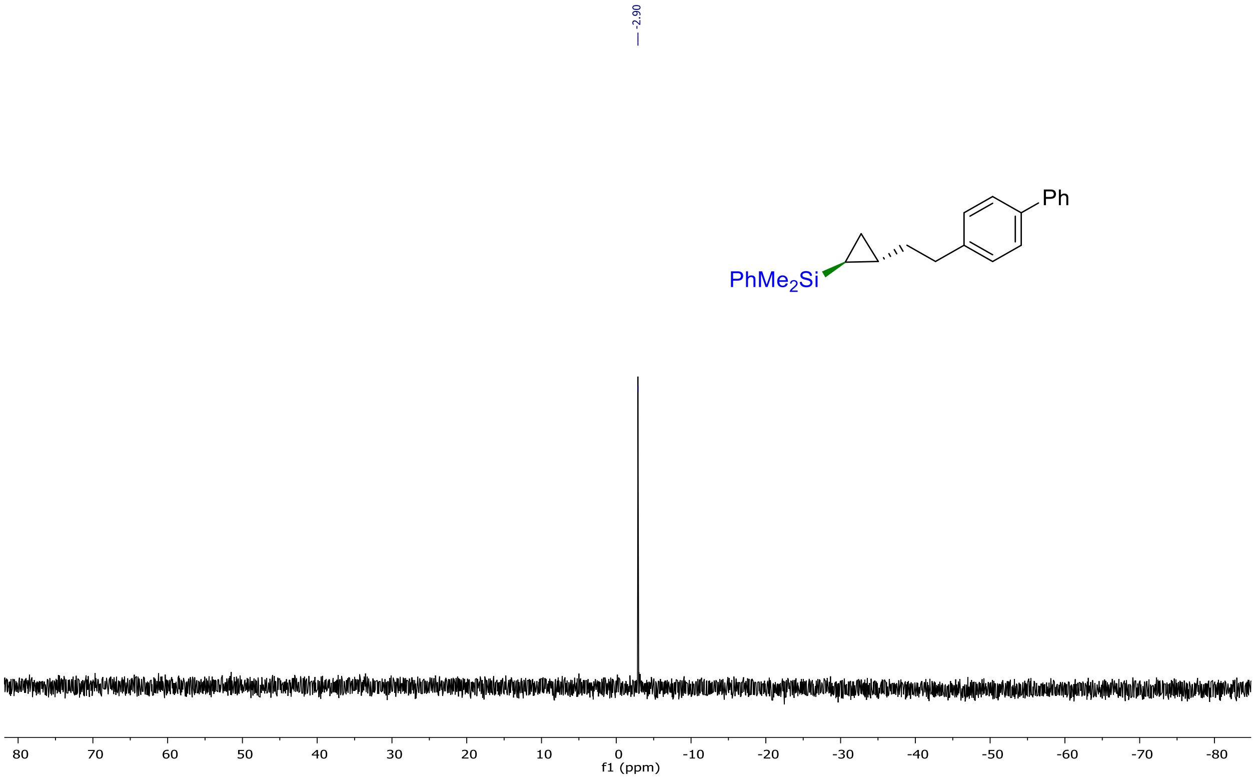

Supplementary Figure 101. ^{1}H and ^{13}C-NMR spectra of ***anti*-3g**

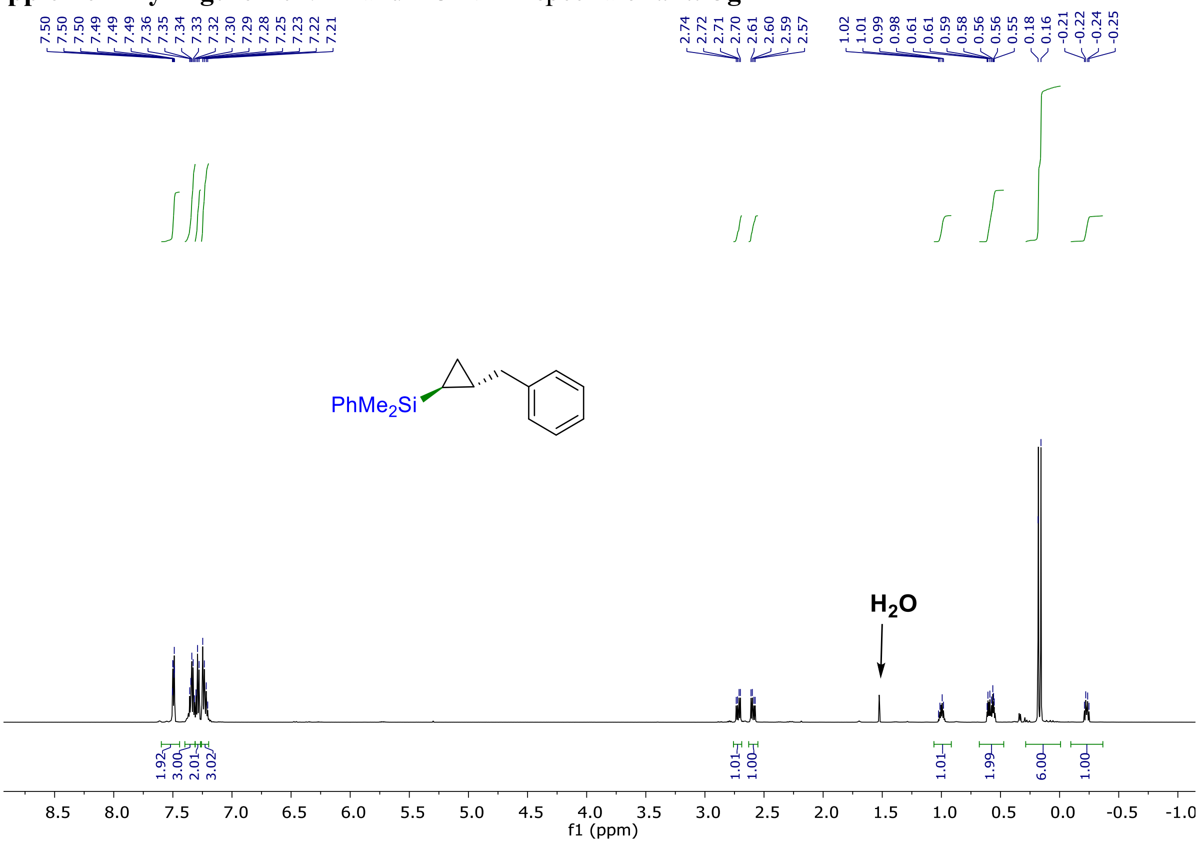

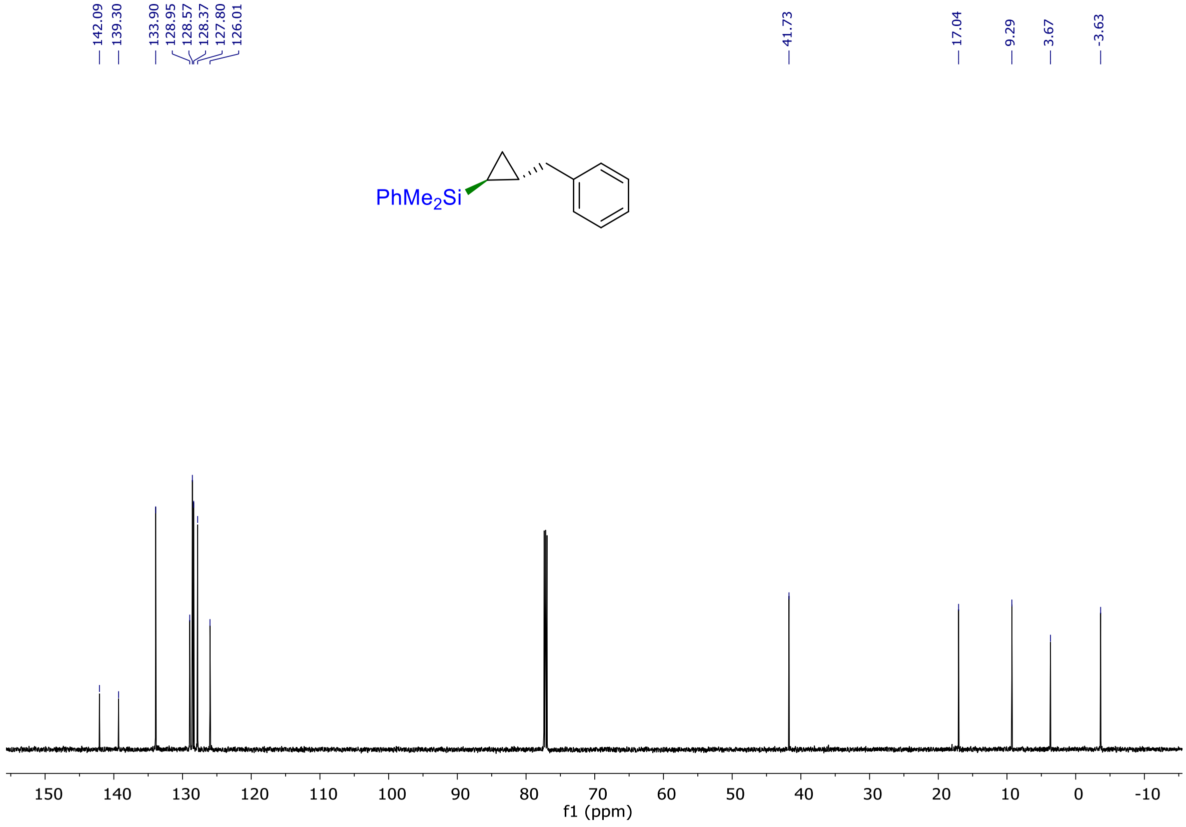

Supplementary Figure 102. ^{29}Si-NMR spectrum of ***anti*-3g**

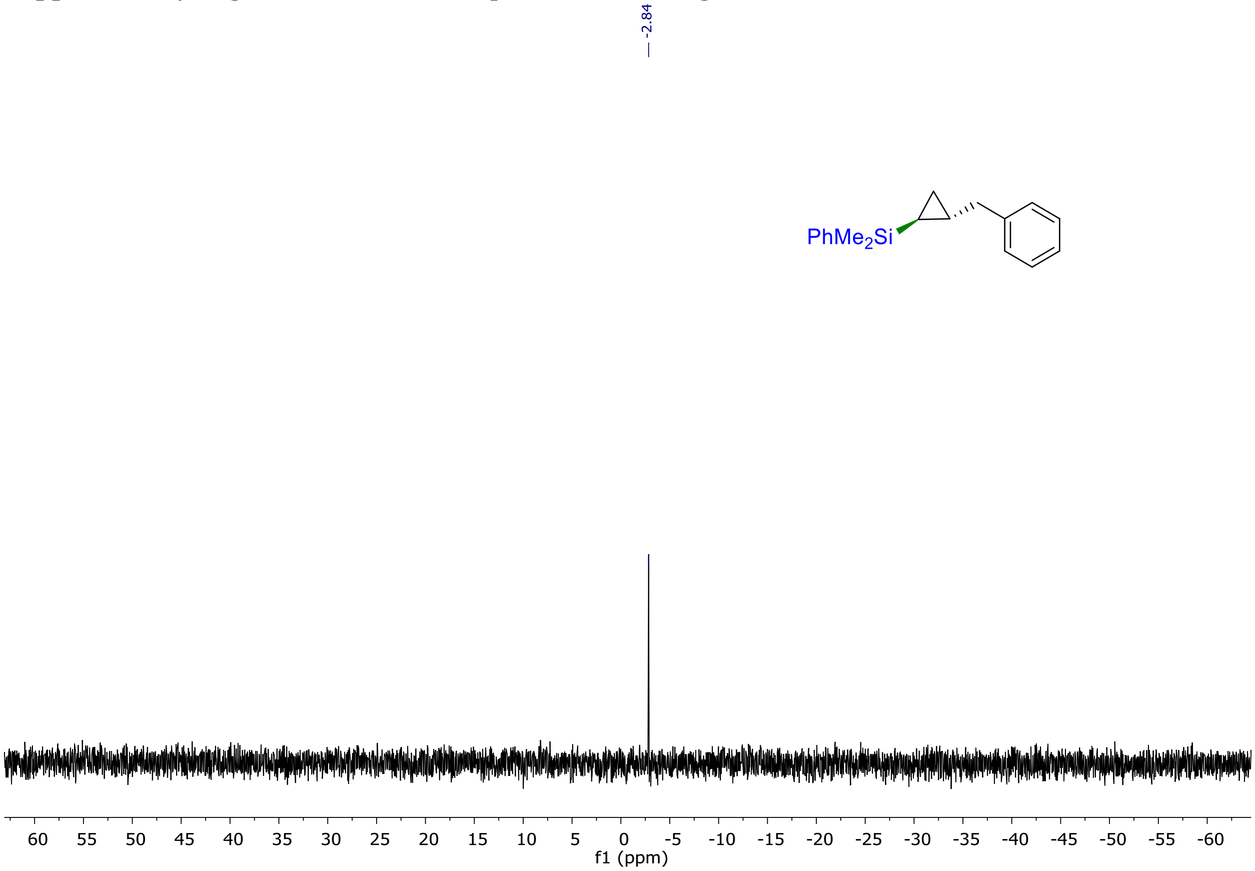

Supplementary Figure 103. ^{1}H-NMR spectrum of ***anti*-3h**

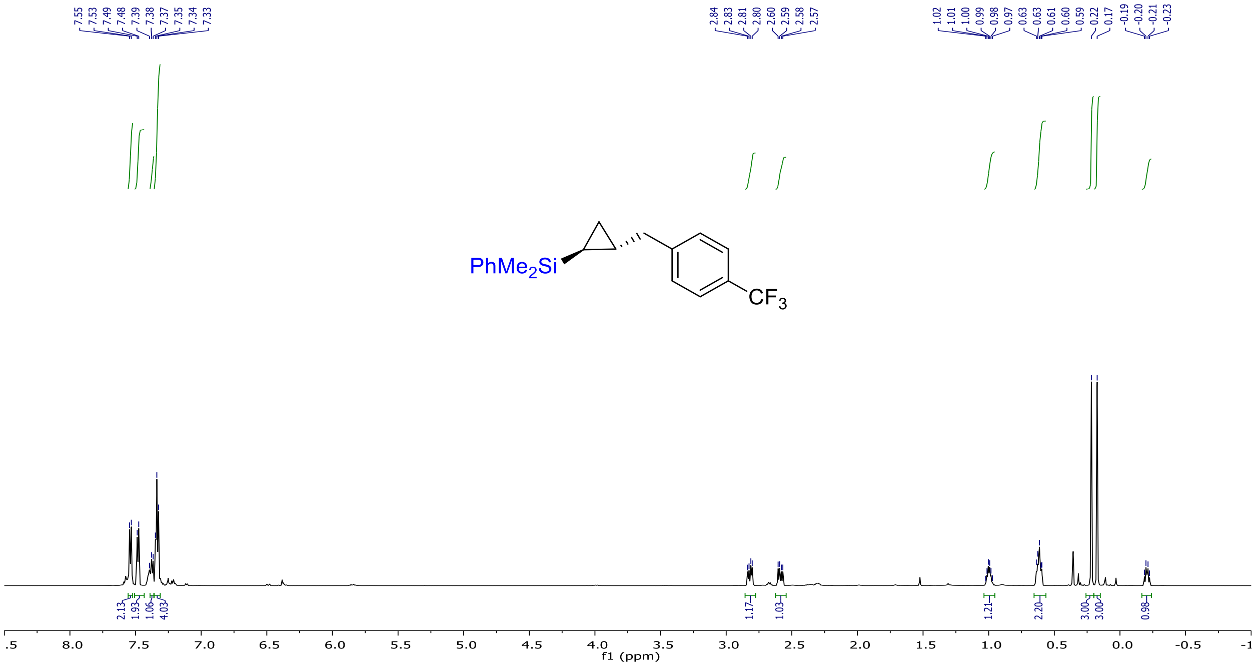

Supplementary Figure 104. ^{13}C and ^{29}Si-NMR spectra of ***anti*-3h**

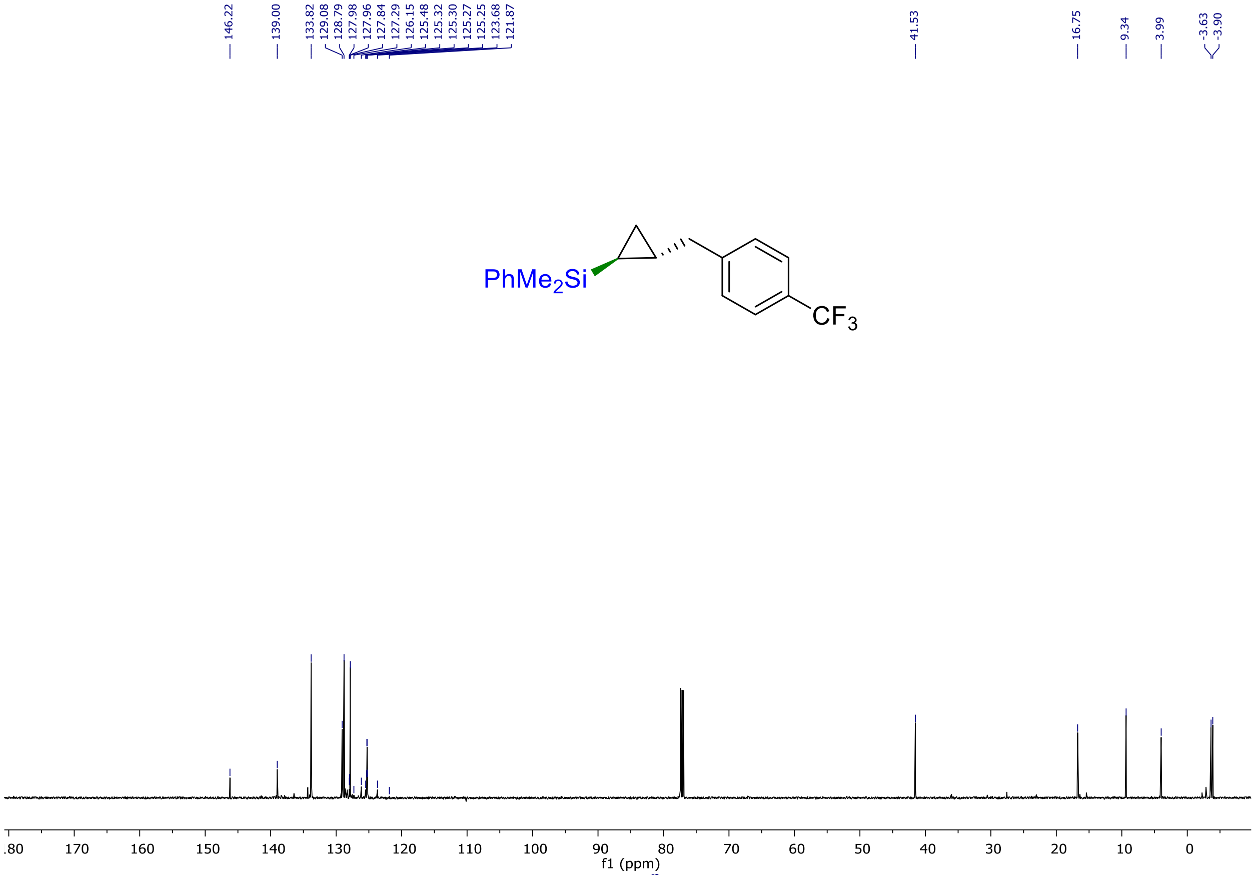

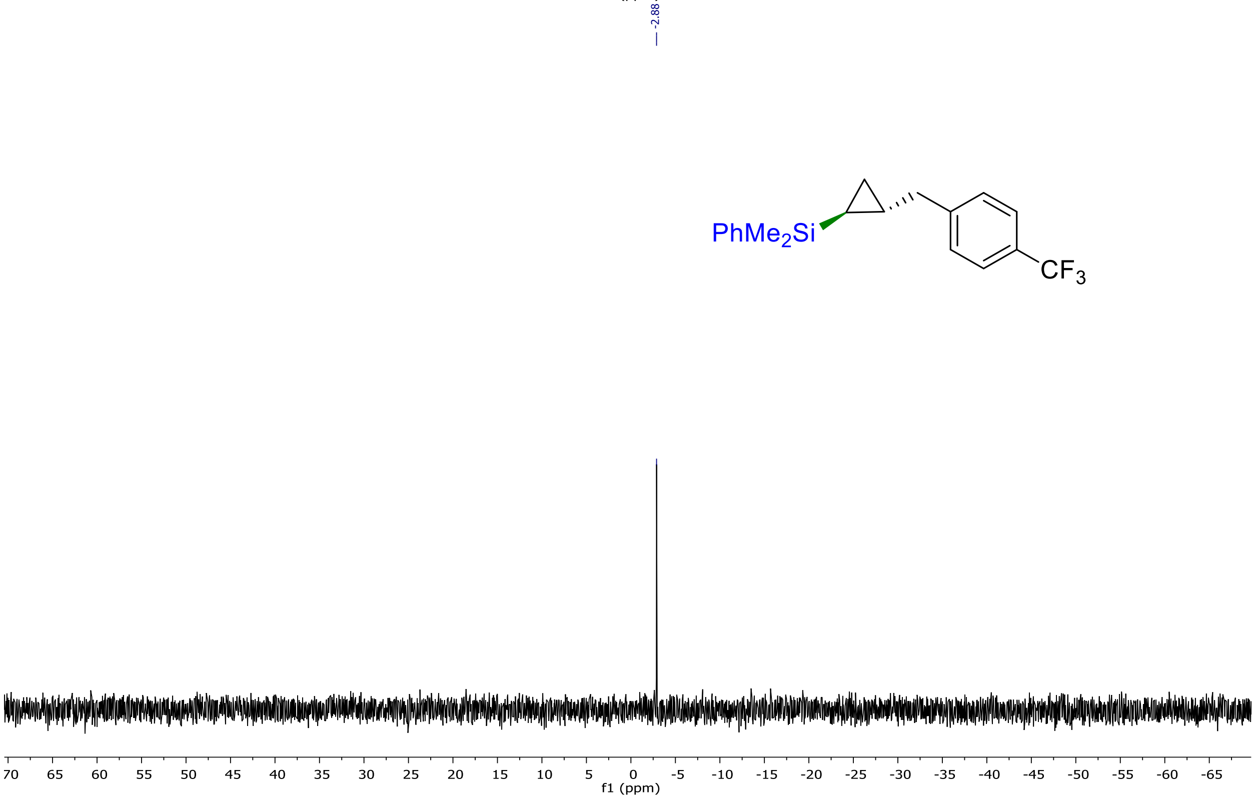

Supplementary Figure 105. ^{19}F-NMR spectrum of ***anti*-3h**

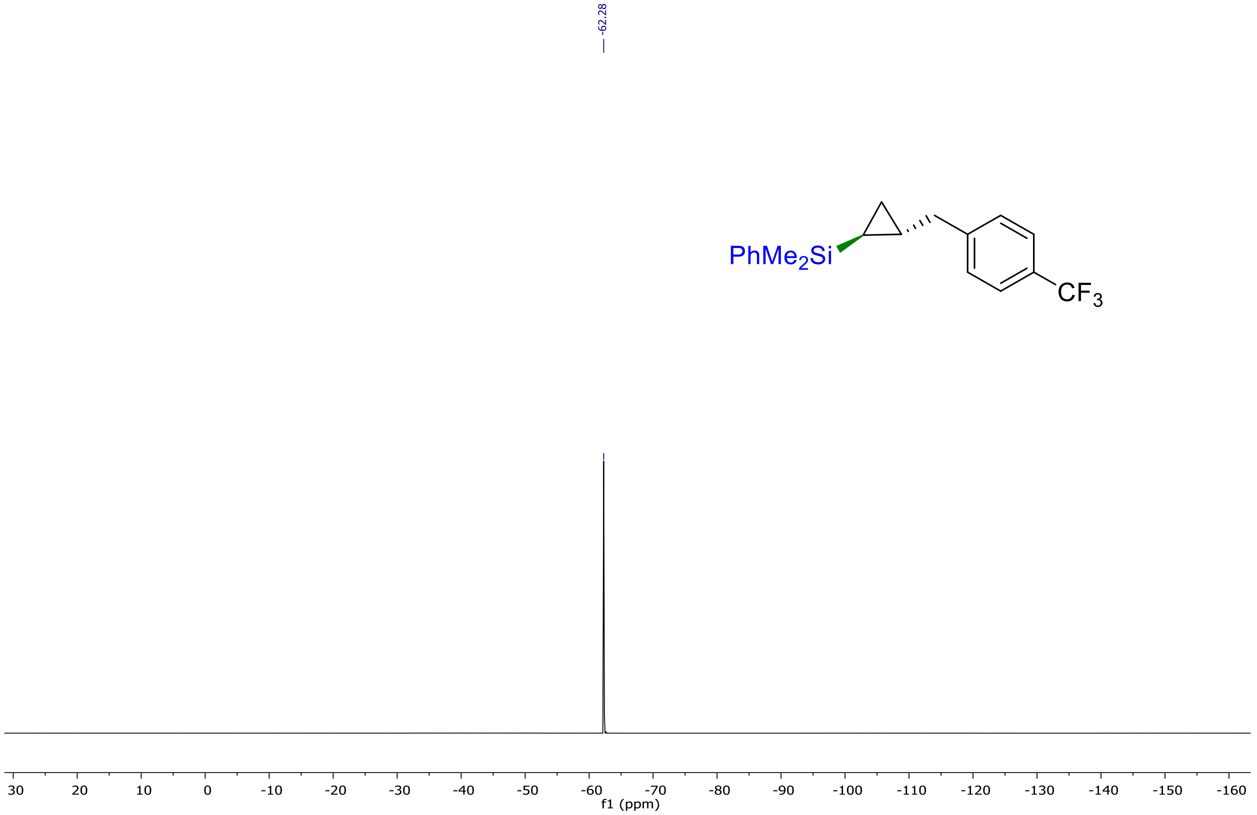

Supplementary Figure 106. ^{1}H-NMR spectrum of ***anti*-3i**

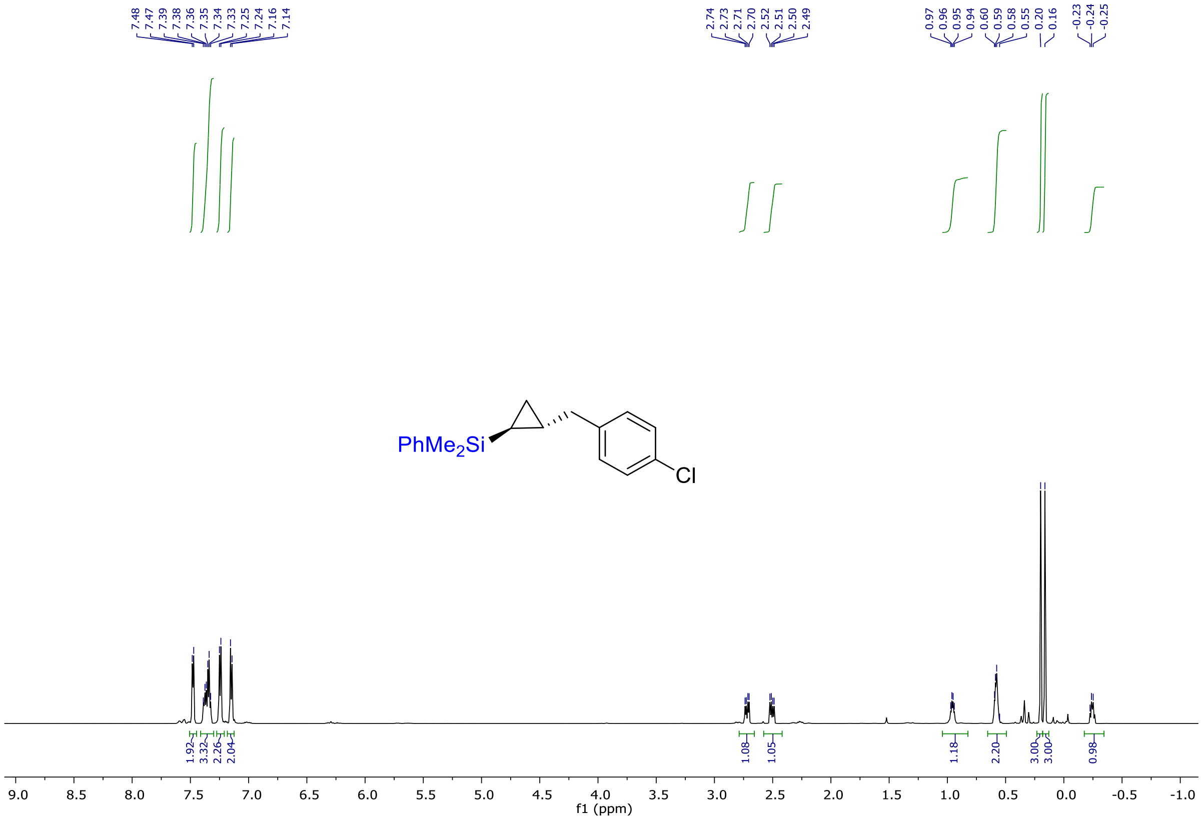

Supplementary Figure 107. ^{13}C and ^{29}Si-NMR spectra of ***anti*-3i**

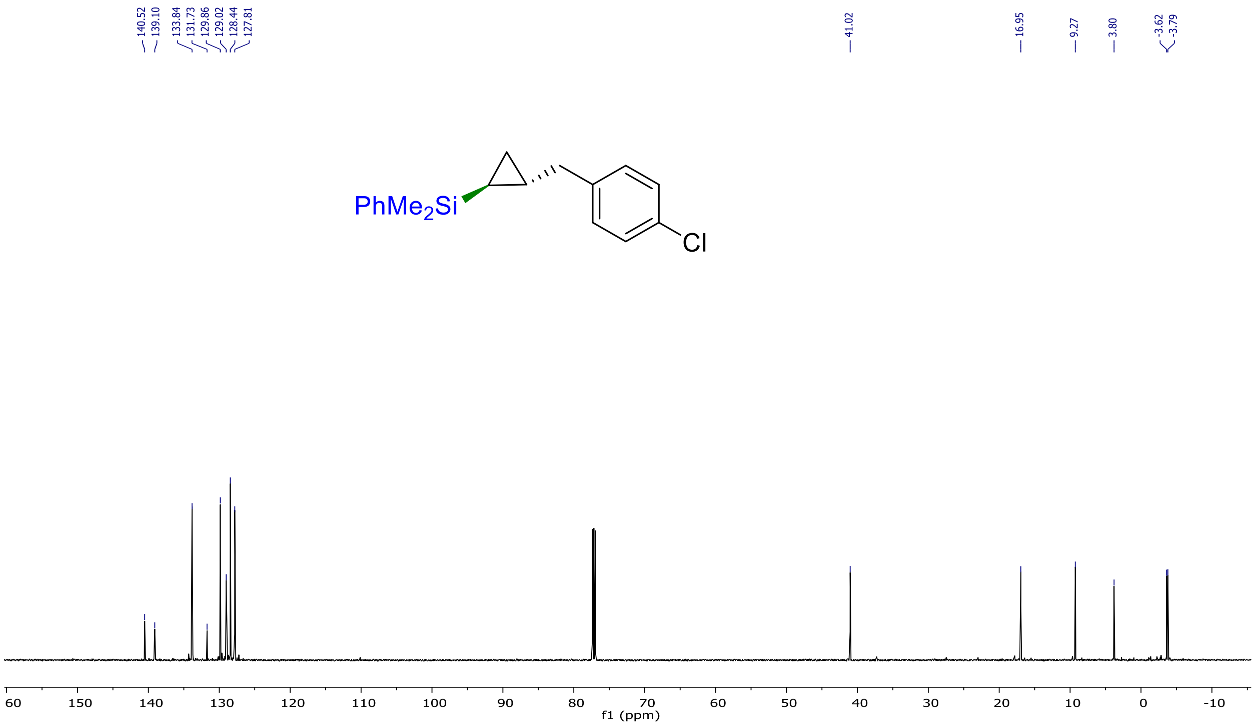

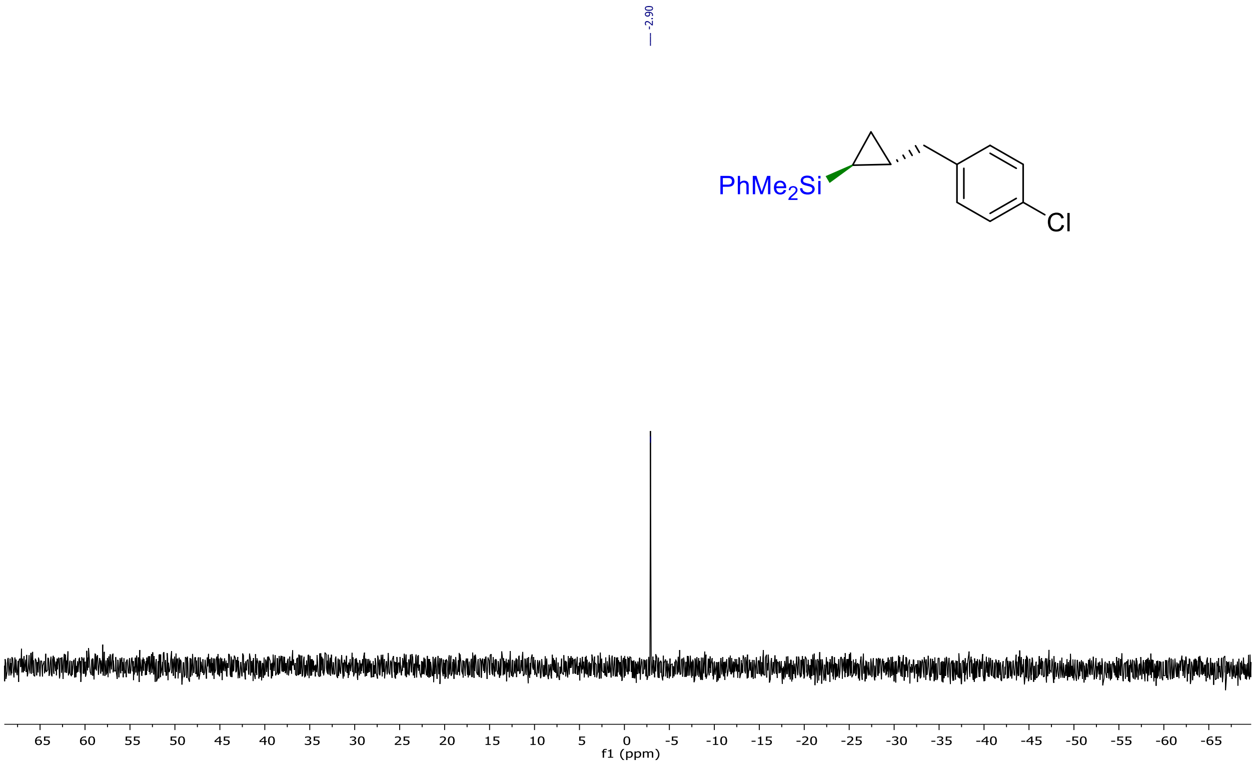

Supplementary Figure 108. ^{1}H and ^{13}C-NMR spectra of ***anti*-3j**

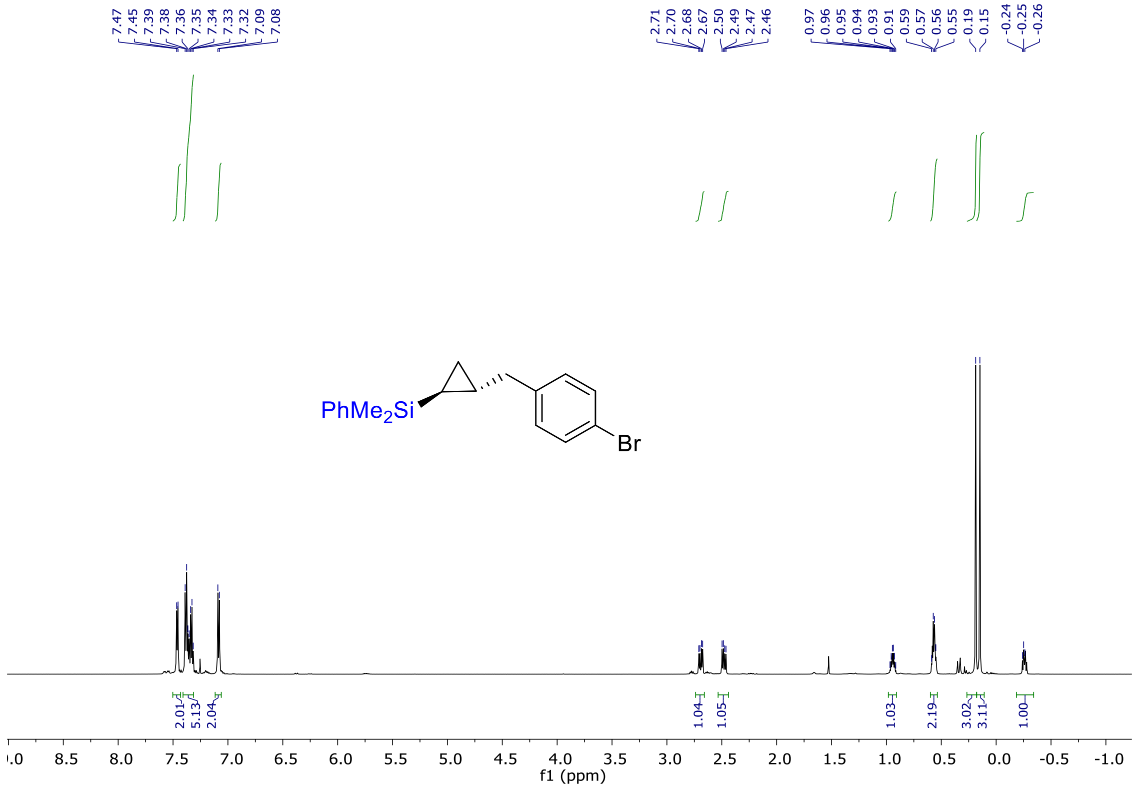

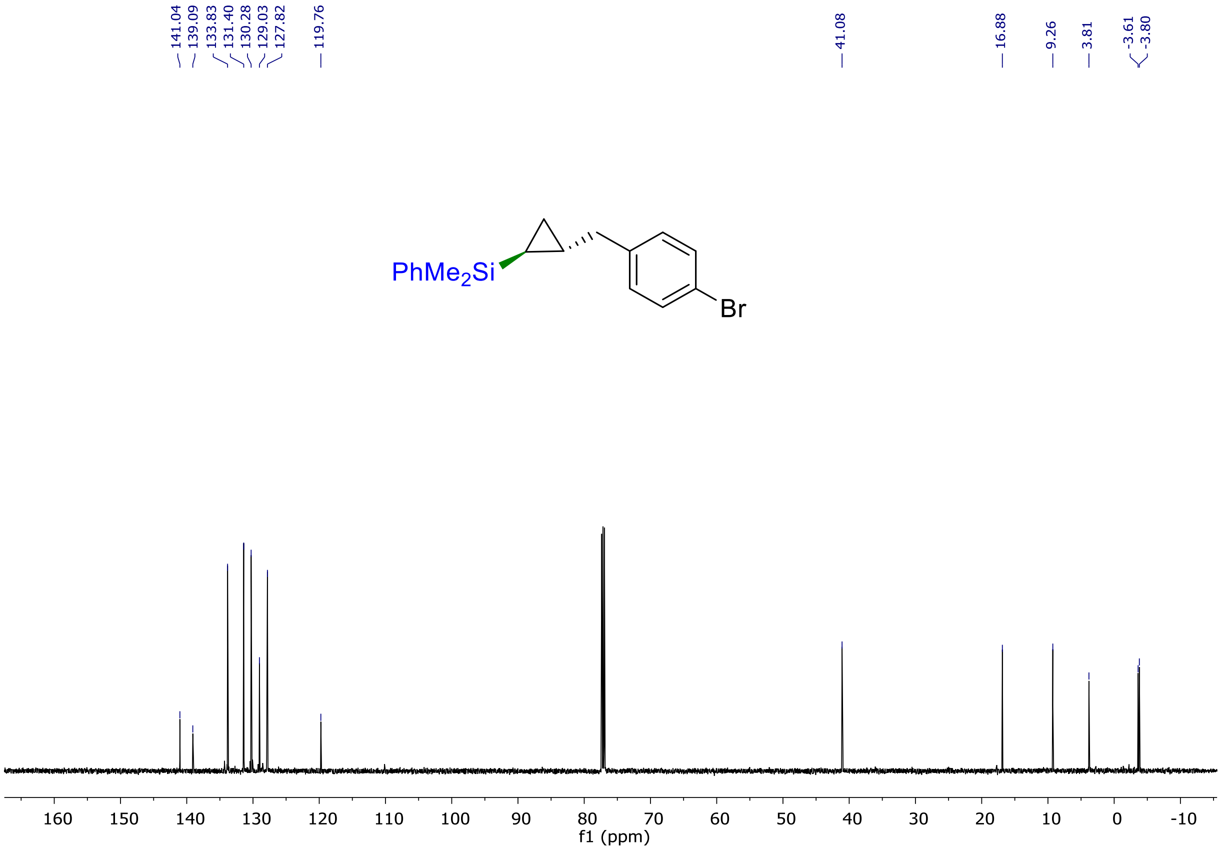

Supplementary Figure 109. ^{29}Si-NMR spectrum of ***anti*-3j**

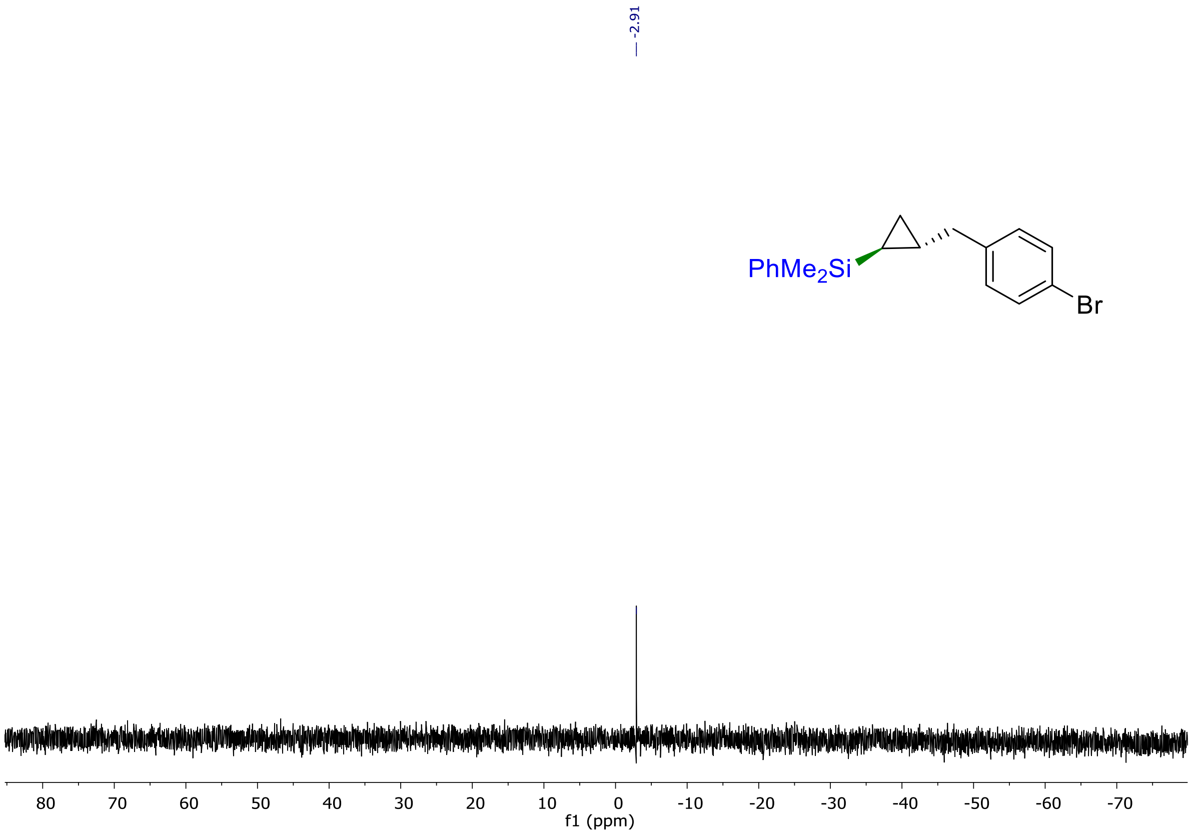

Supplementary Figure 110. ^{1}H-NMR spectrum of ***anti*-3k**

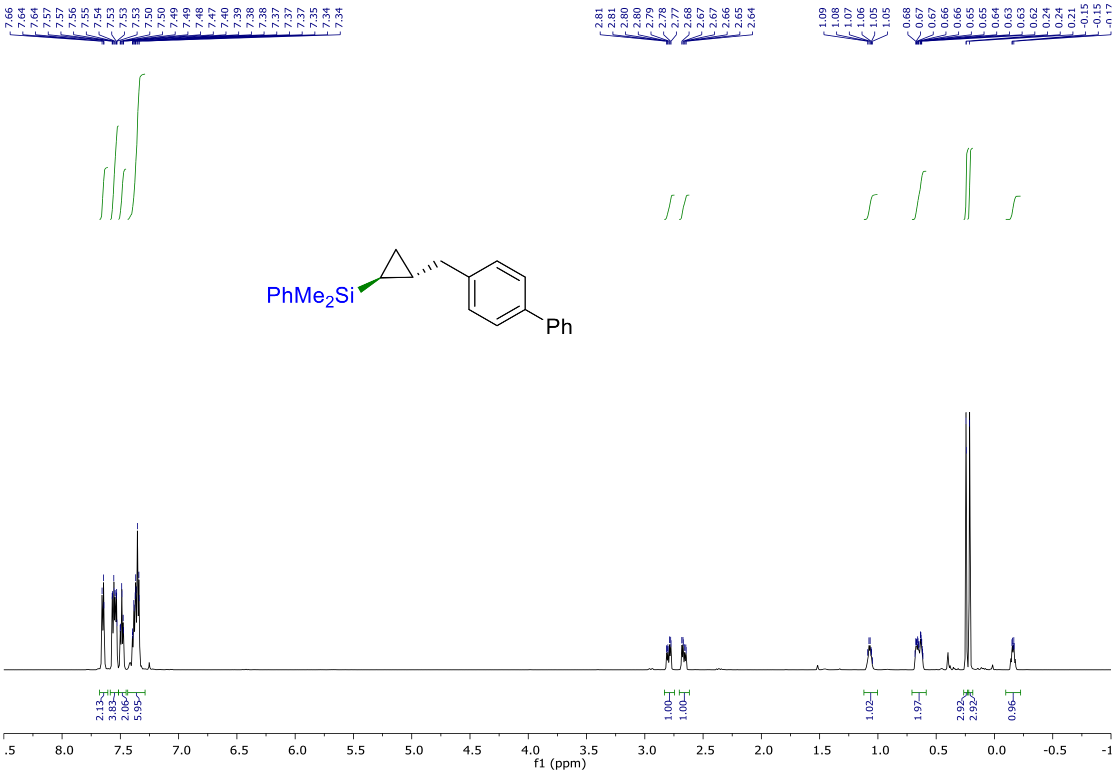

Supplementary Figure 111. ^{13}C and ^{29}Si-NMR spectra of ***anti*-3k**

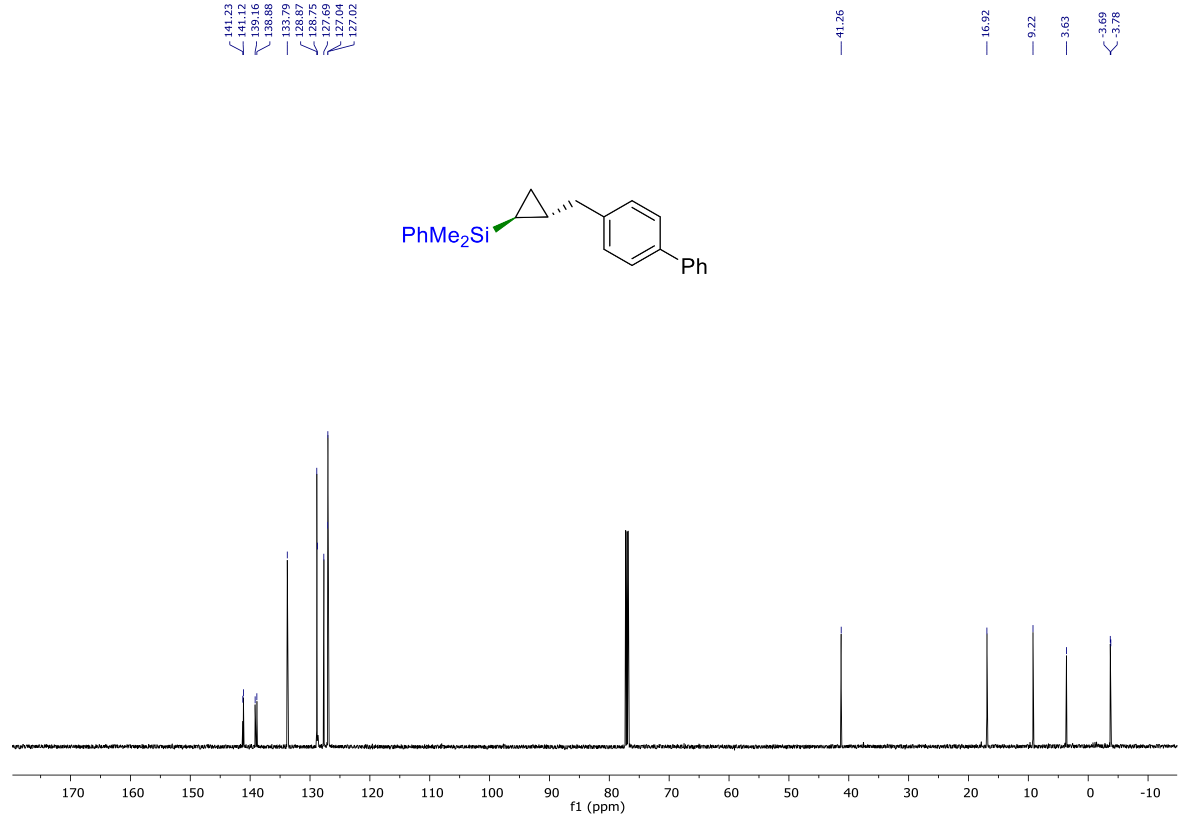

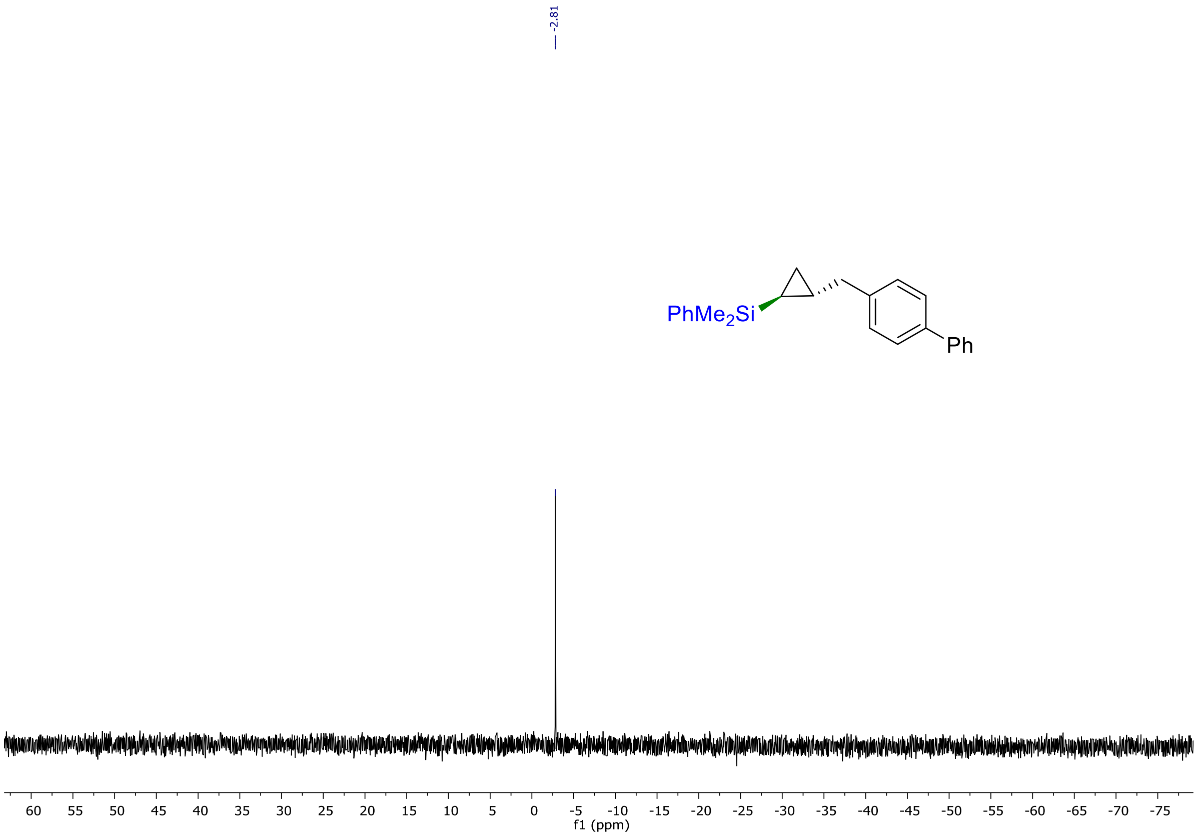

Supplementary Figure 112. ^{1}H and ^{13}C-NMR spectra of ***anti*-3l**

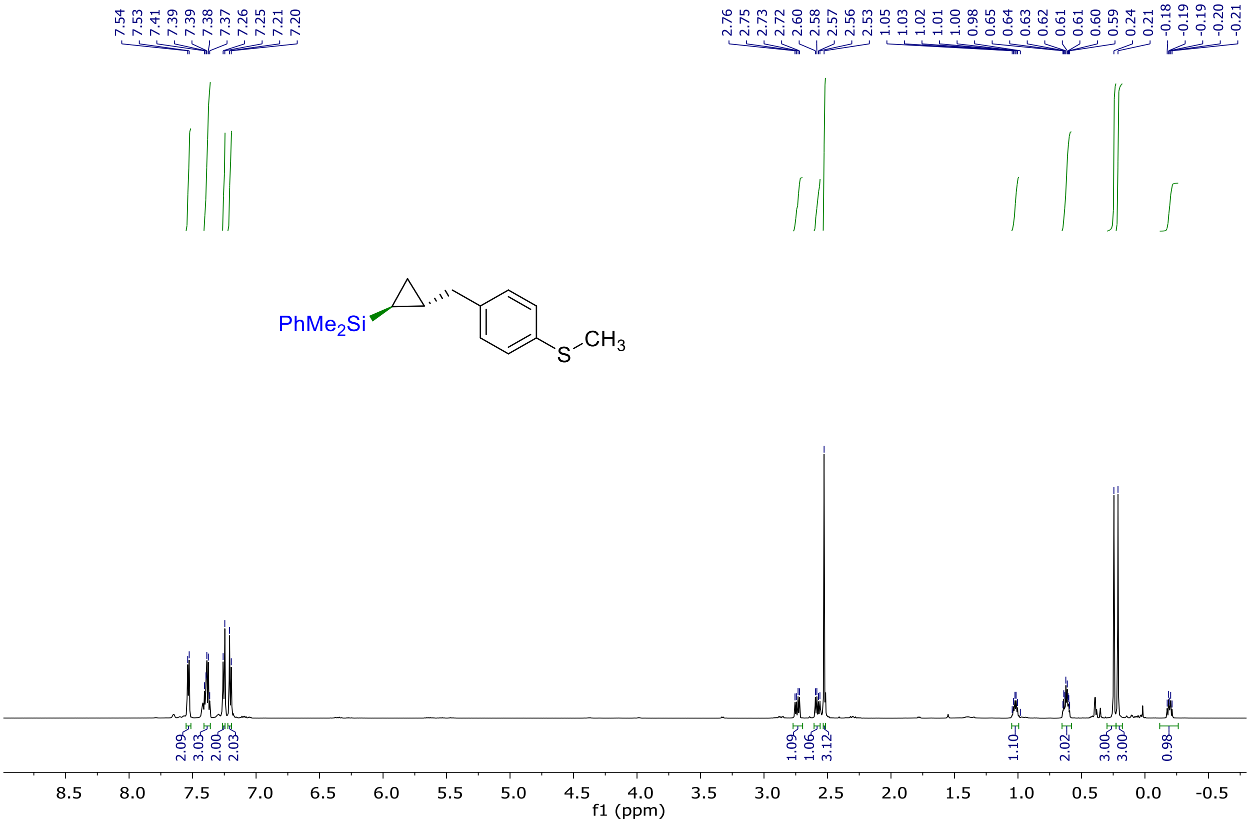

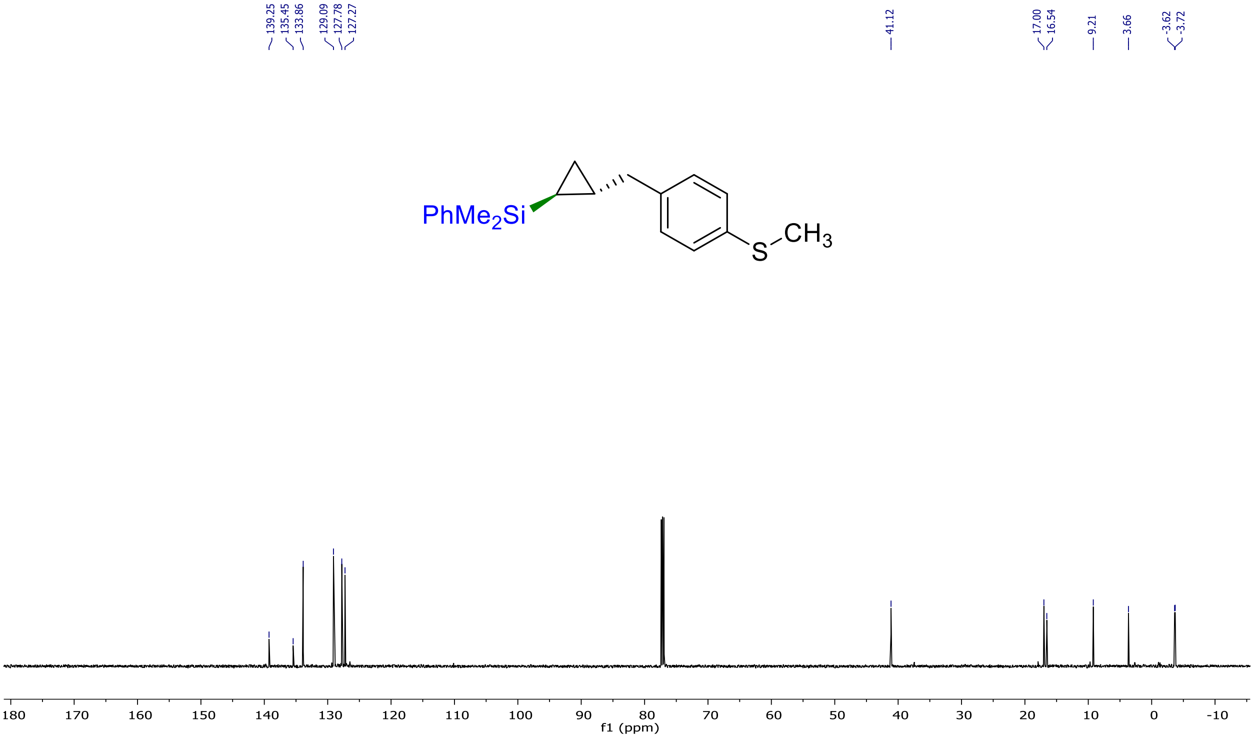

Supplementary Figure 113. ^{29}Si-NMR spectrum of ***anti*-3l**

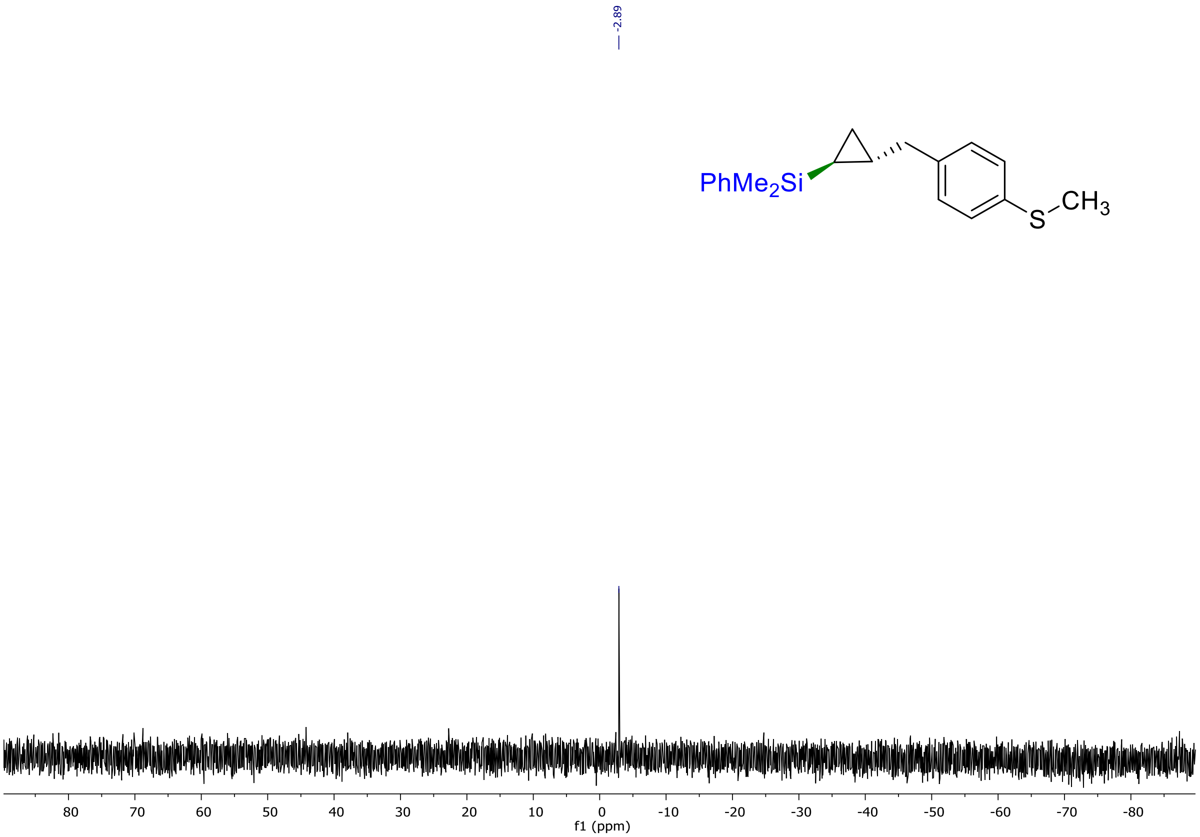

Supplementary Figure 114. ^{1}H-NMR spectrum of ***anti*-3m**

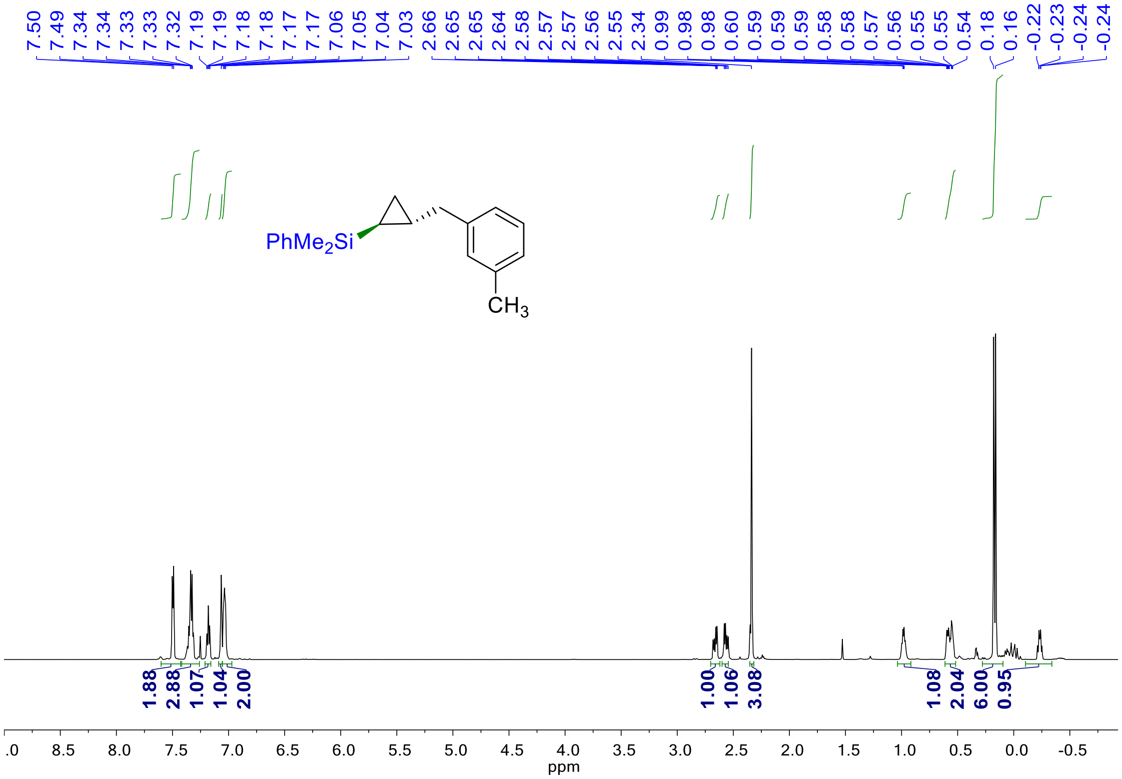

Supplementary Figure 115. ^{13}C and ^{29}Si-NMR spectra of ***anti*-3m**

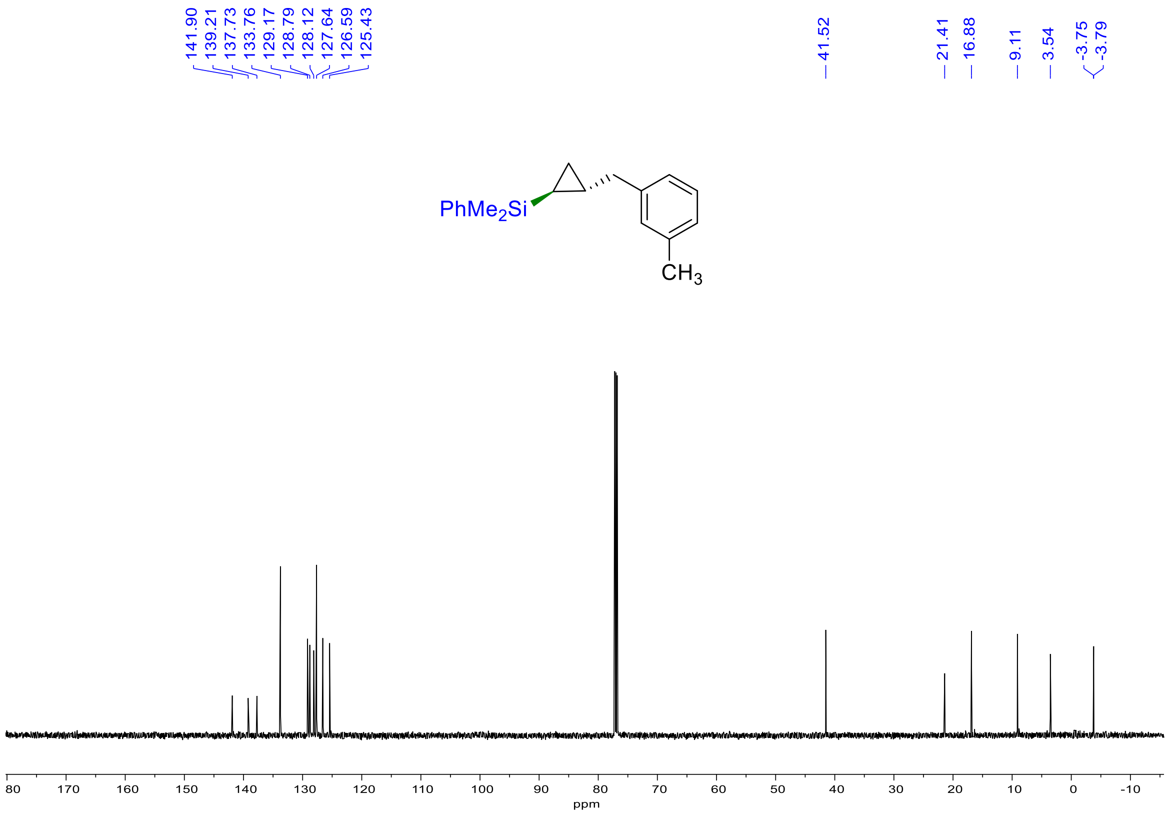

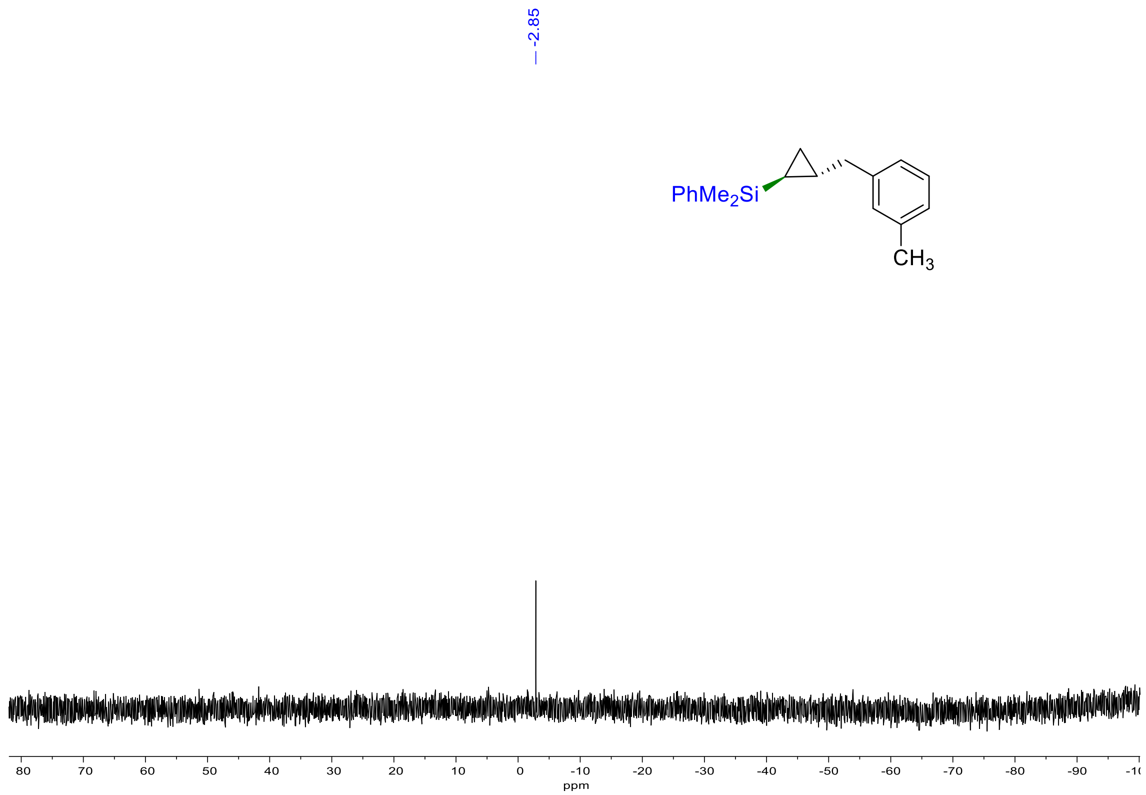

Supplementary Figure 116. ^{1}H and ^{13}C-NMR spectra of ***anti*-3m**

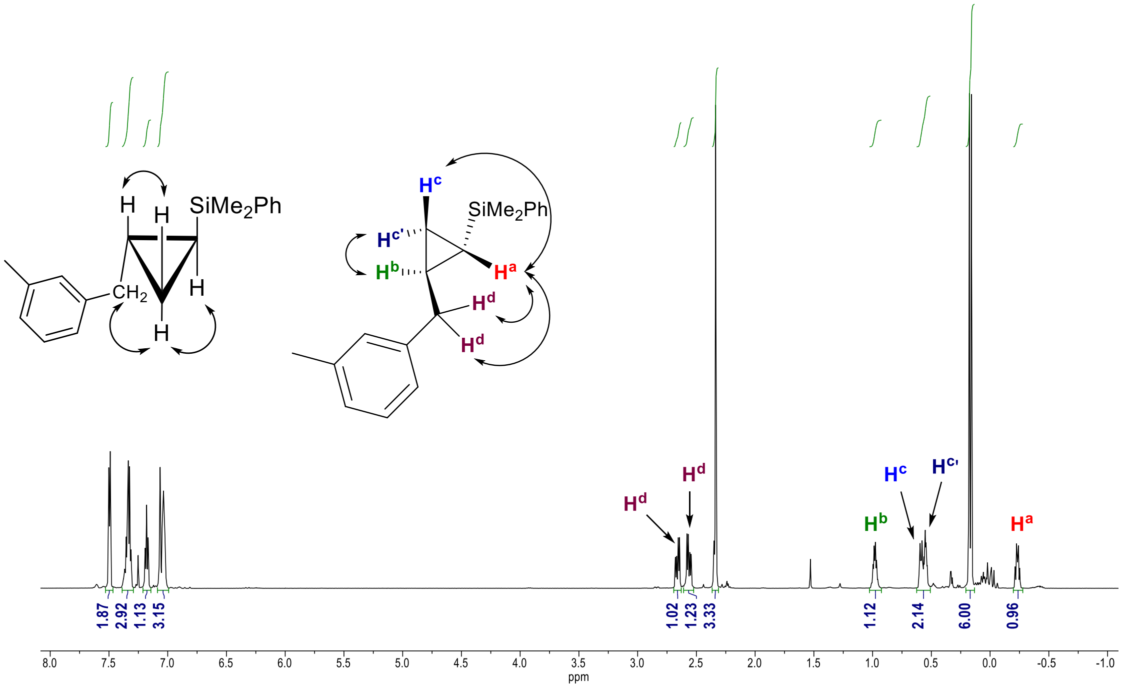

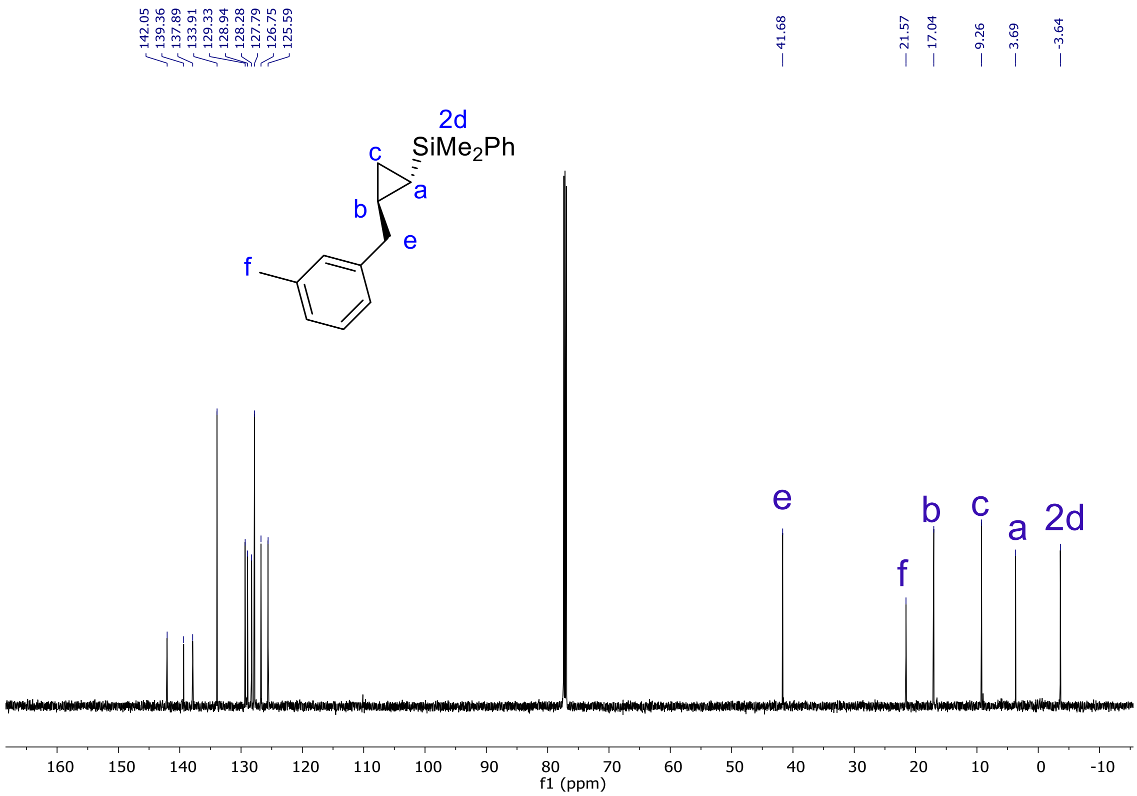

Supplementary Figure 117. DEPT 135 spectrum of ***anti*-3m**

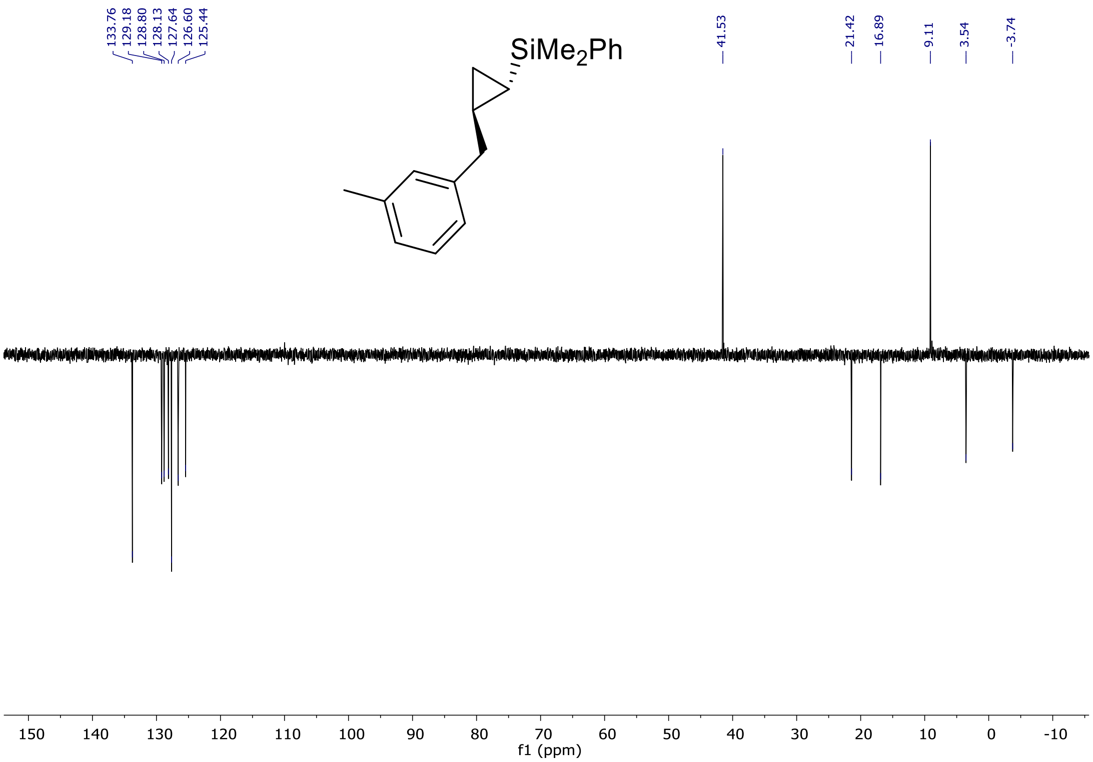

Supplementary Figure 118. 1D NOESY spectrum of ***anti*-3m**

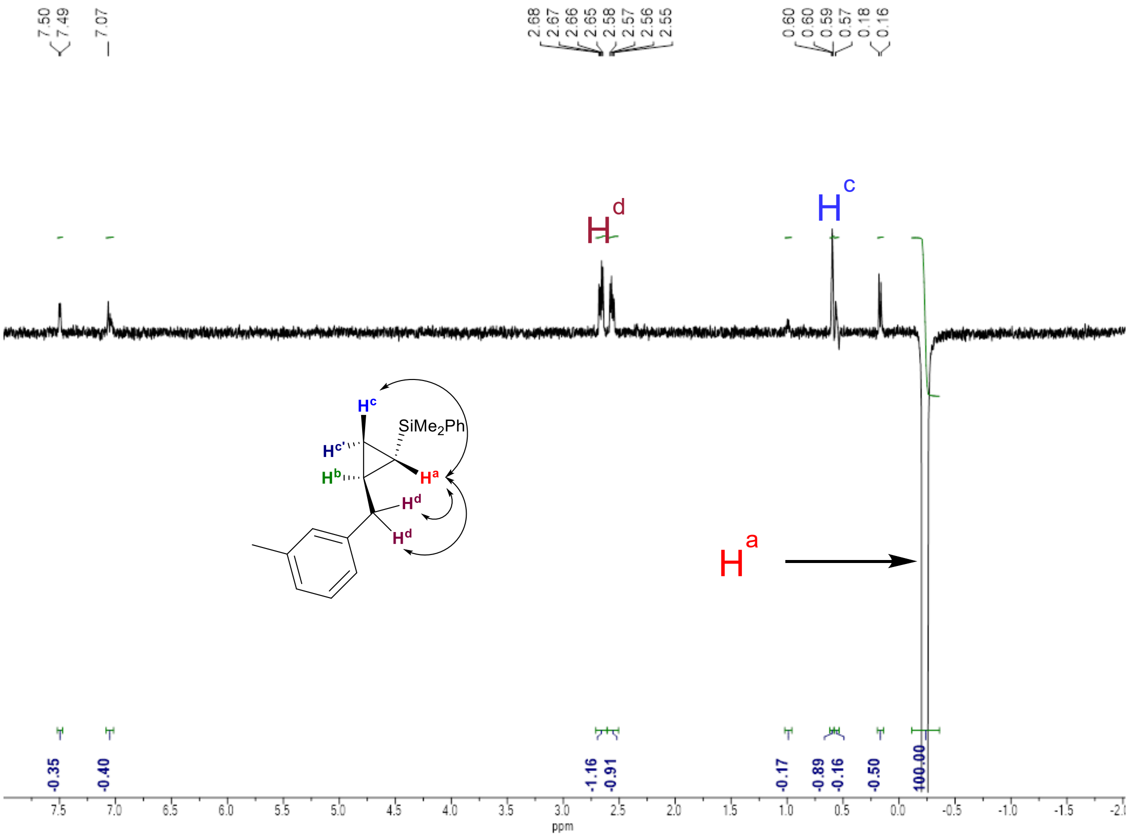

Supplementary Figure 119. 1D NOESY spectrum of ***anti*-3m**

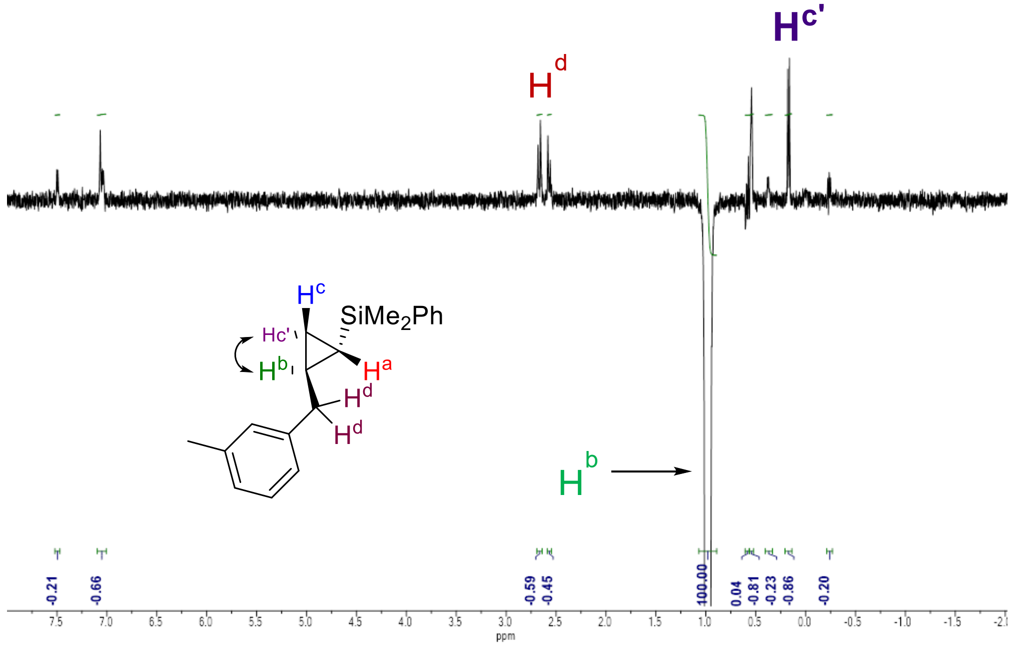

Supplementary Figure 120. H-H COSY spectrum of ***anti*-3m**

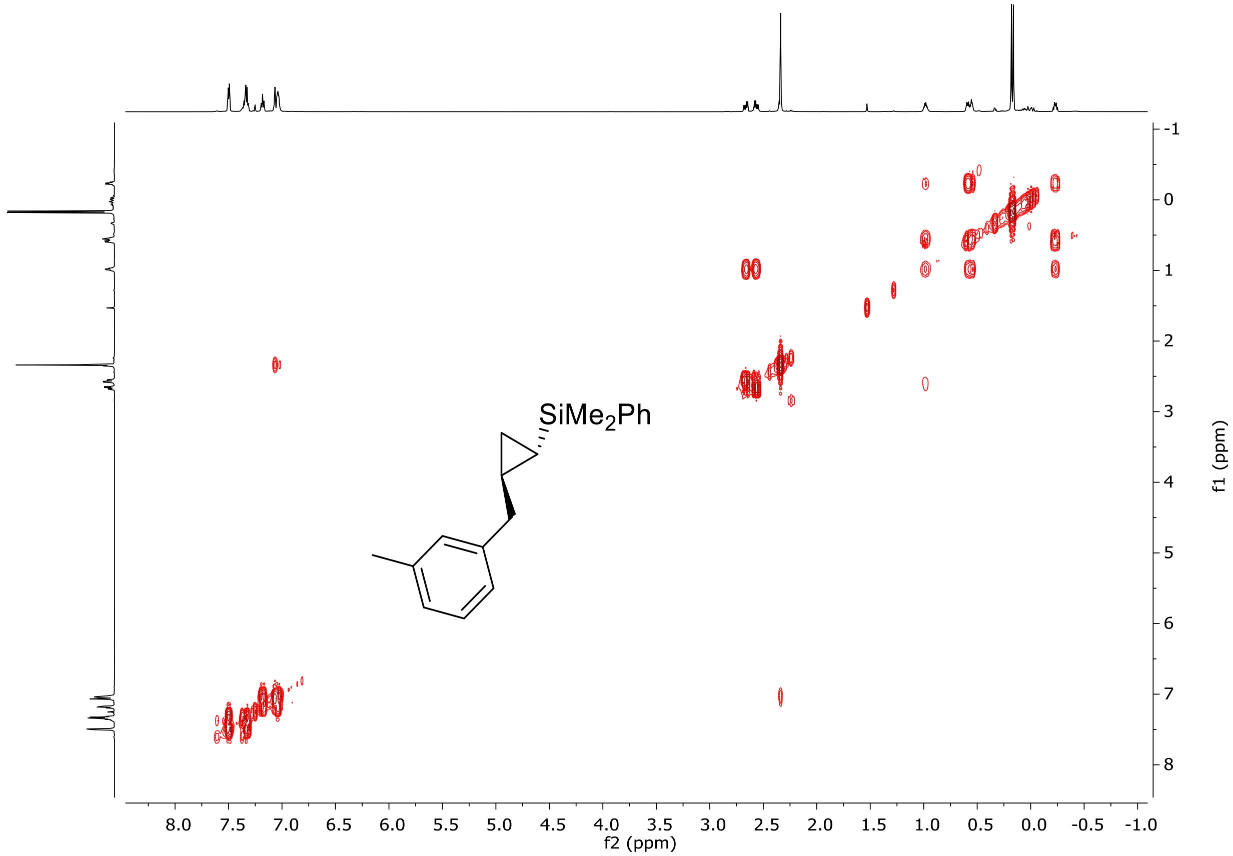

Supplementary Figure 121. HMBC spectrum of ***anti*-3m**

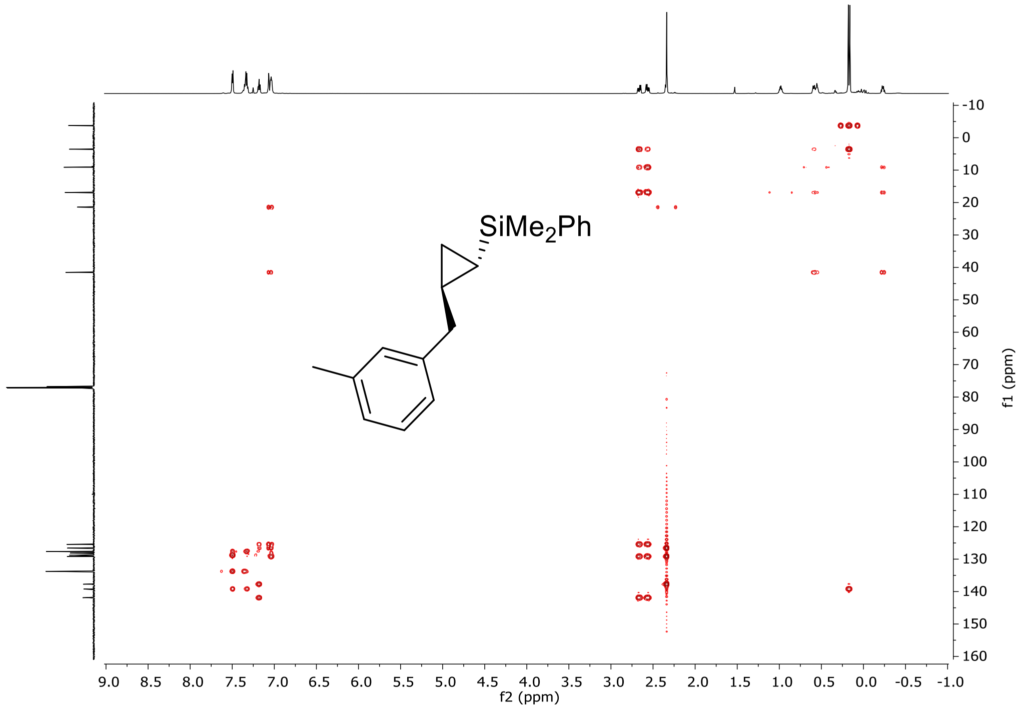

Supplementary Figure 122. HSQCspectrum of ***anti*-3m**

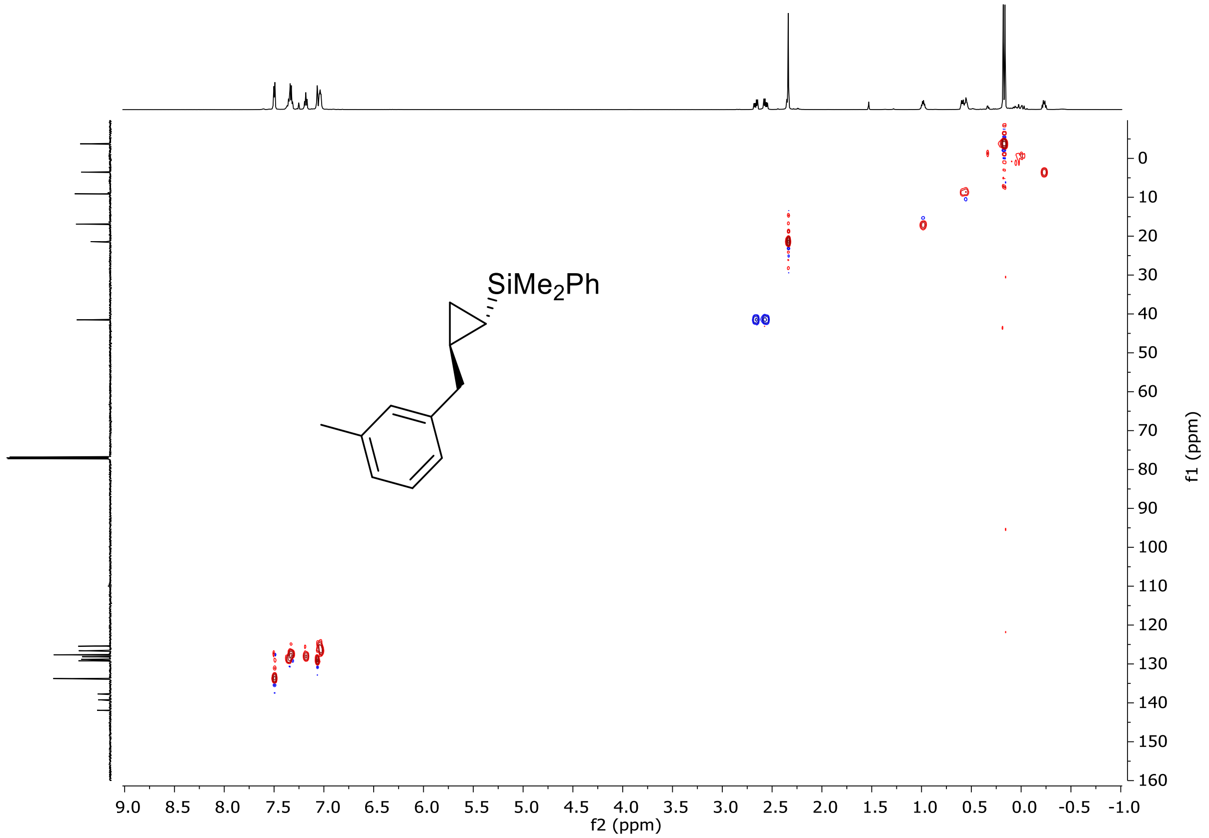

Supplementary Figure 123. ^{1}H and ^{13}C-NMR spectra of ***anti*-3n**

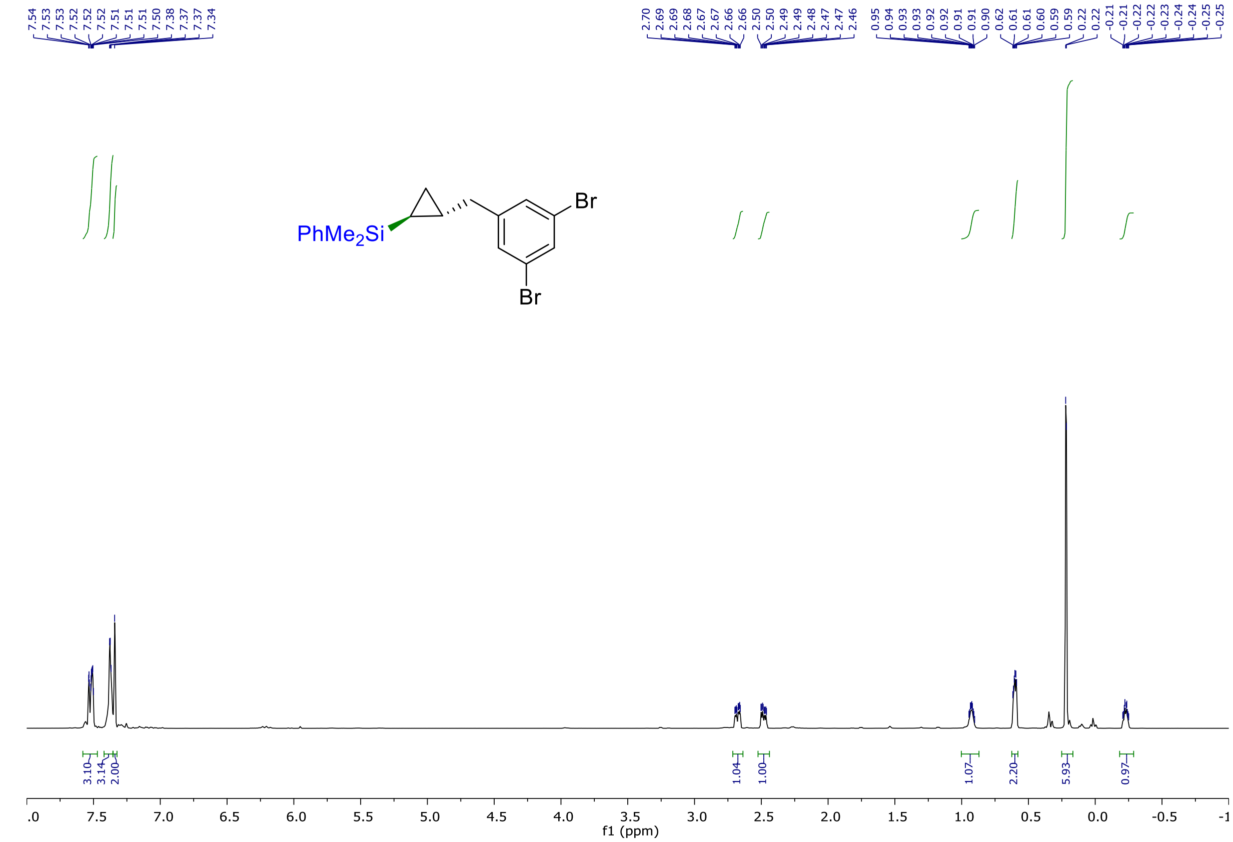

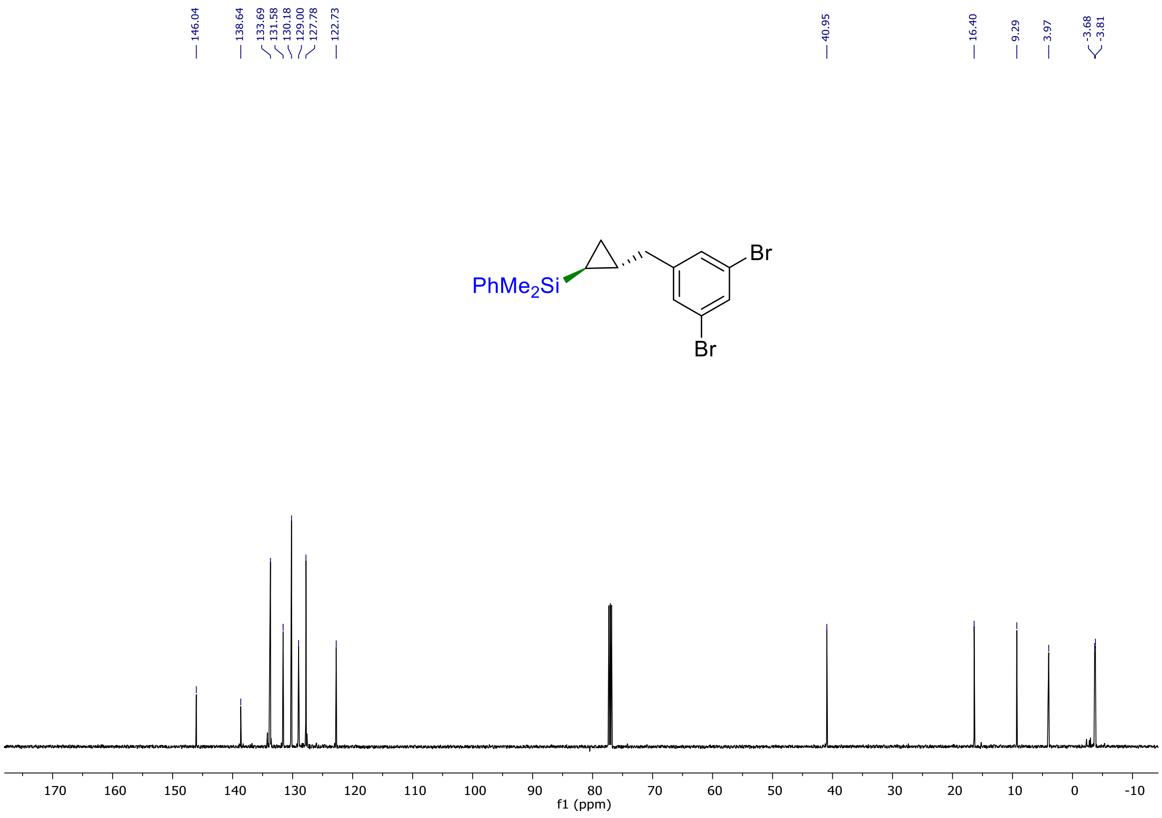

Supplementary Figure 124. ^{29}Si-NMR spectrum of ***anti*-3n**

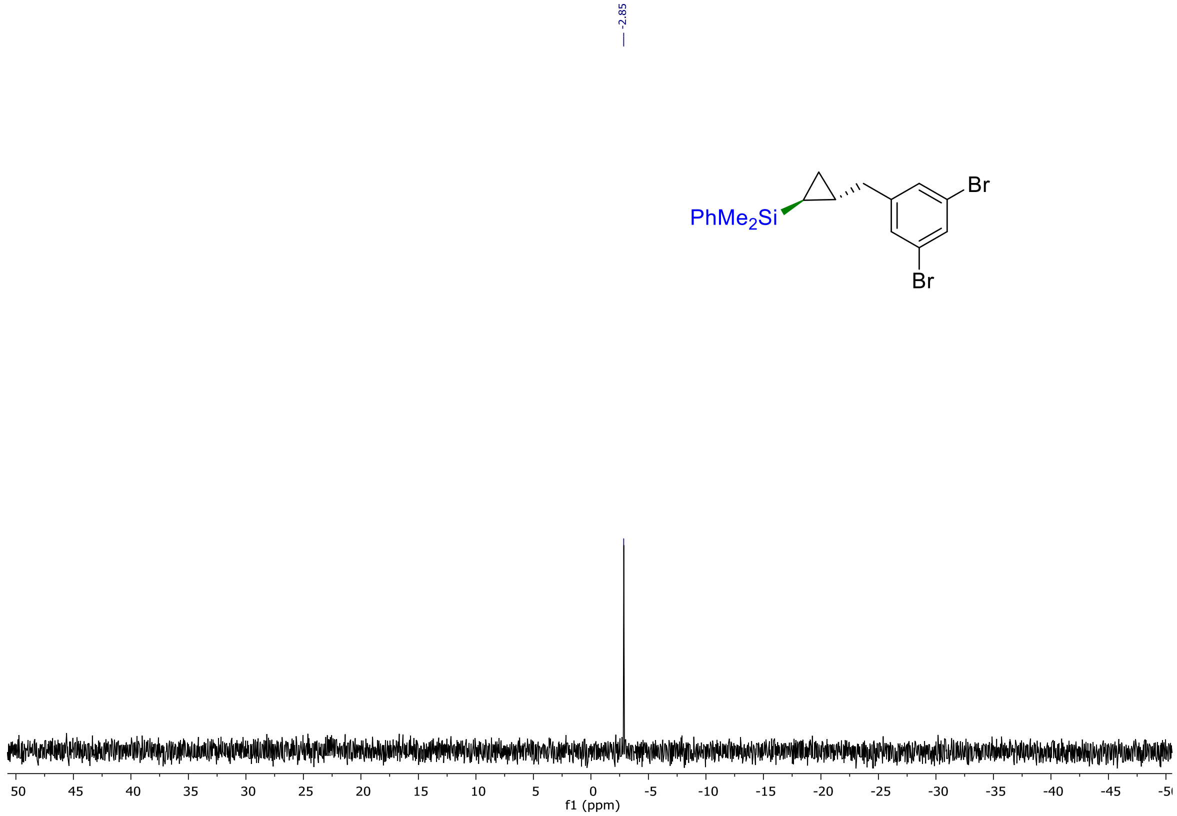

Supplementary Figure 125. ^{1}H-NMR spectrum of ***anti*-3o**

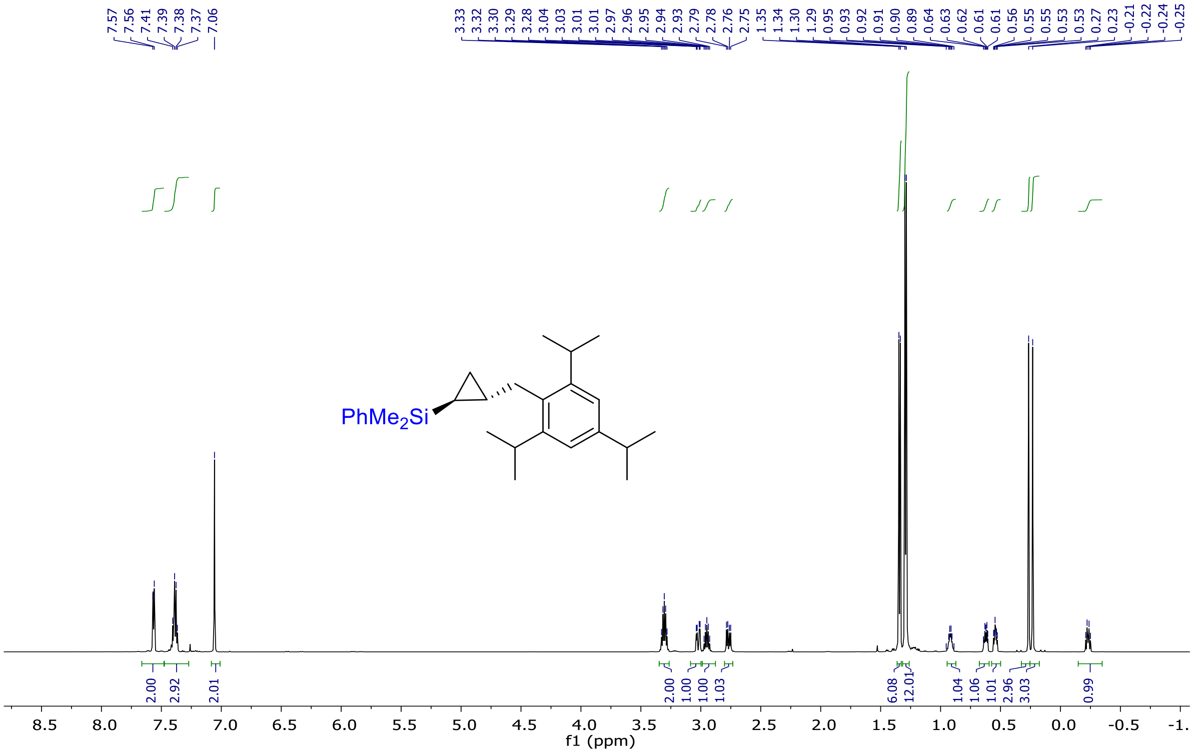

Supplementary Figure 126. ^{13}C and ^{29}Si-NMR spectra of ***anti*-3o**

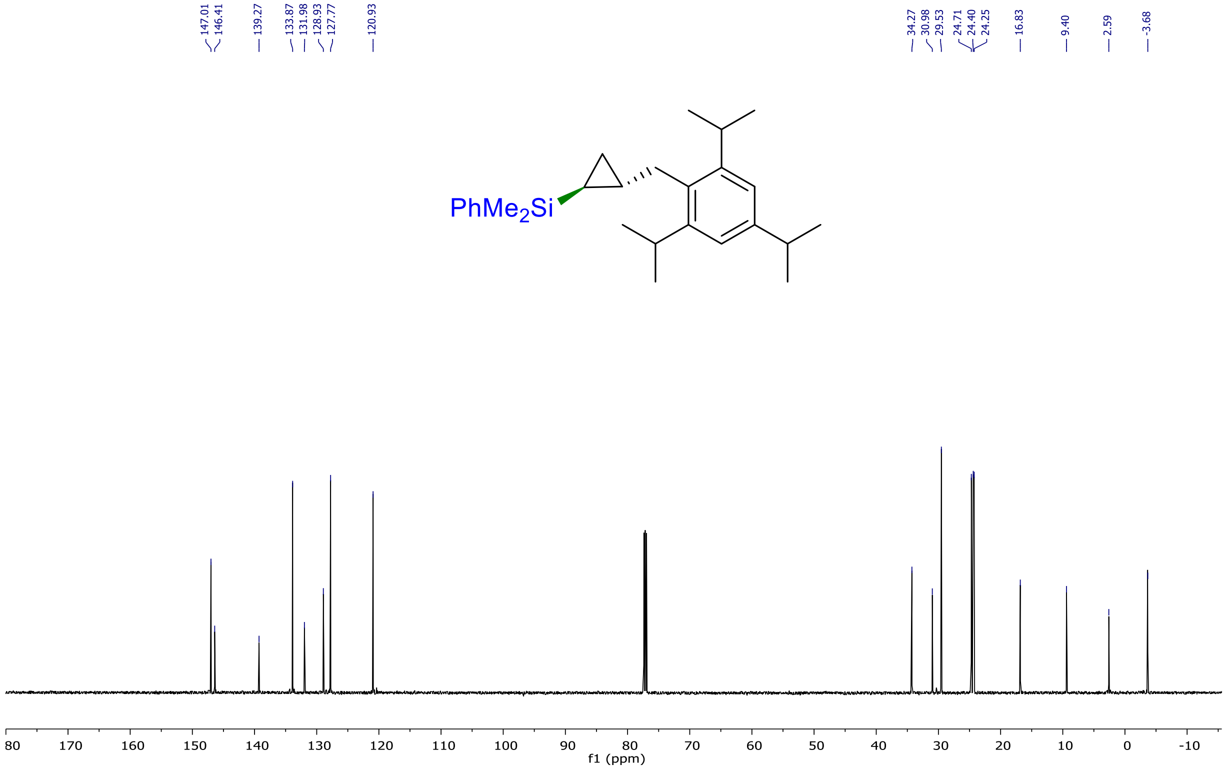

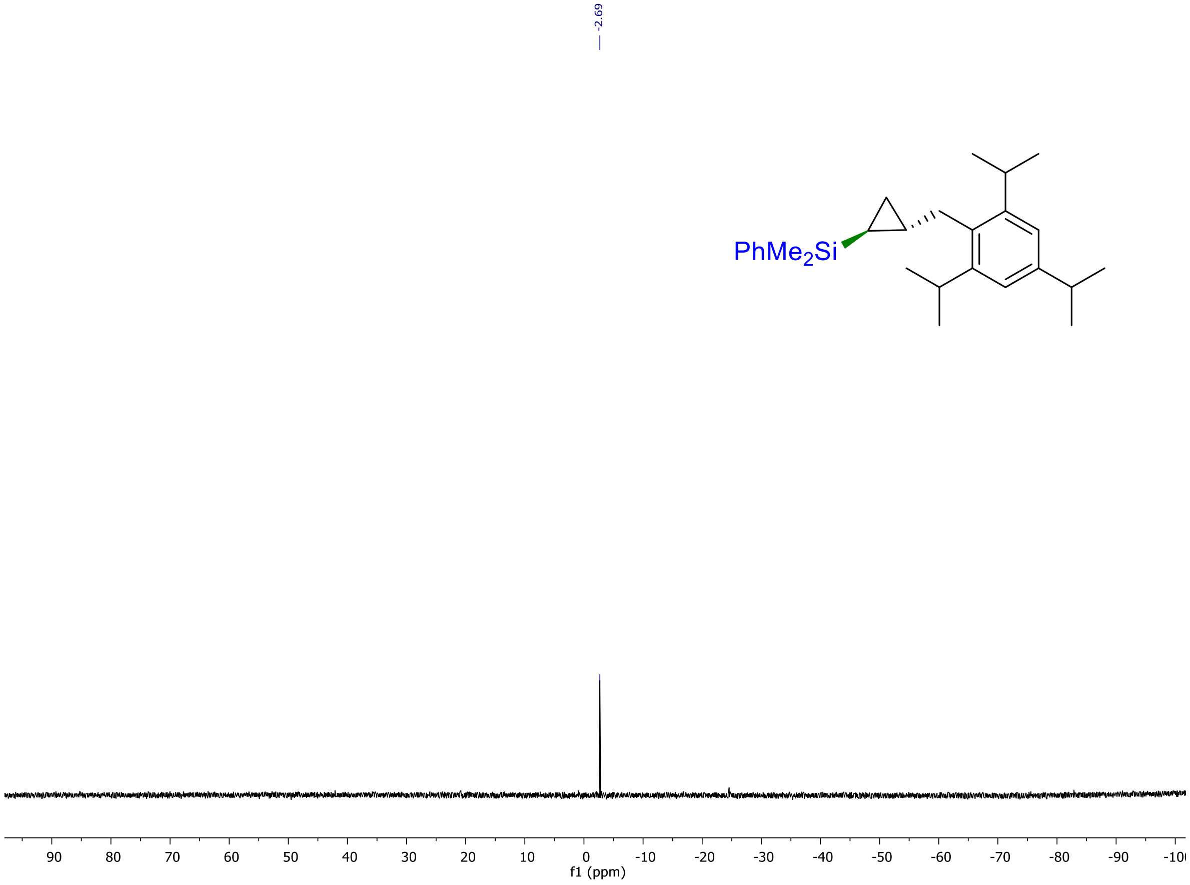

Supplementary Figure 127. ^{1}H and ^{13}C-NMR spectra of ***anti*-3p**

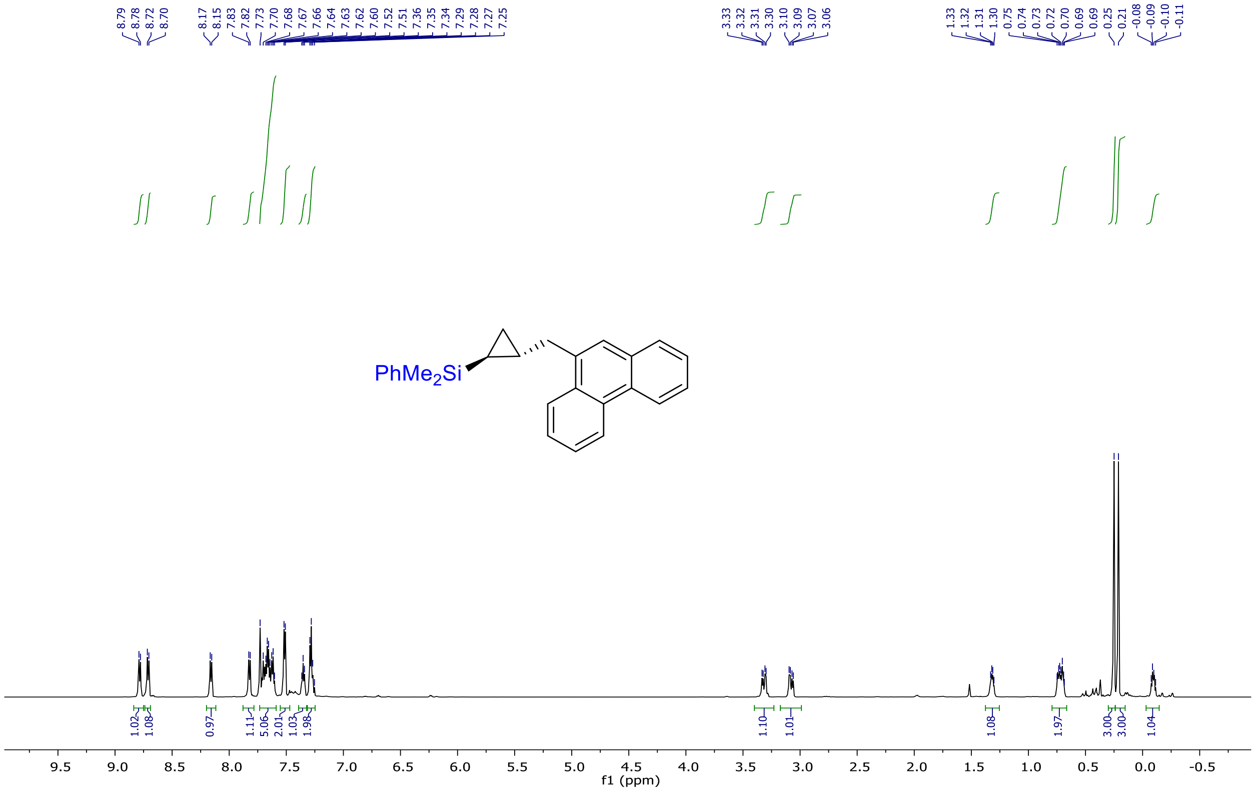

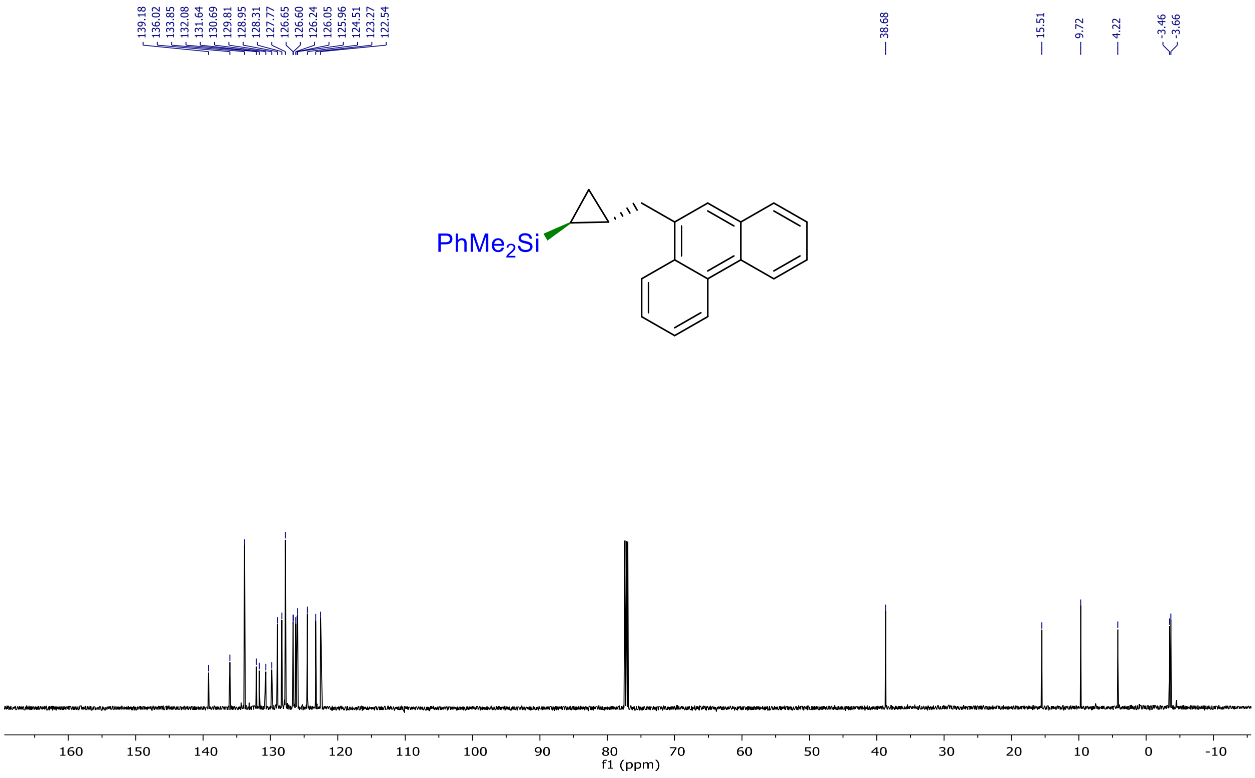

Supplementary Figure 128. ^{29}Si-NMR spectrum of ***anti*-3p**

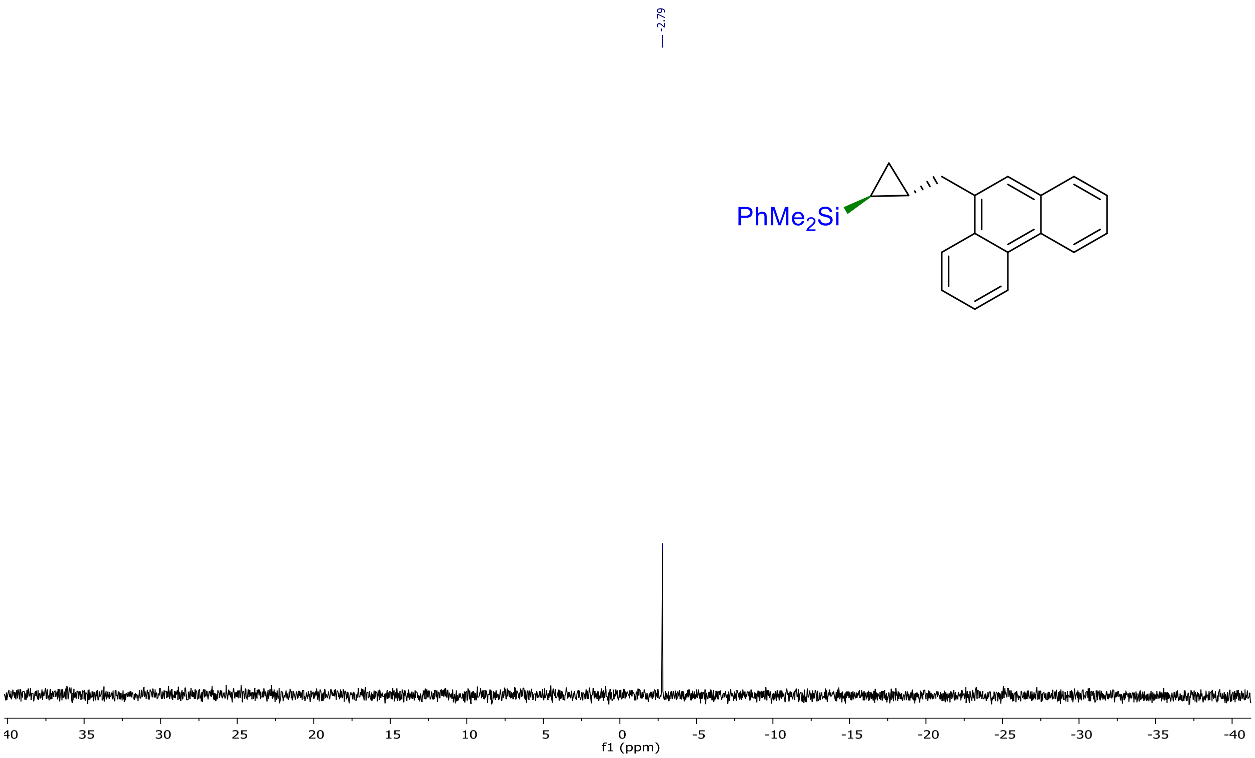

Supplementary Figure 129. ^{1}H-NMR spectrum of ***anti*-3q**

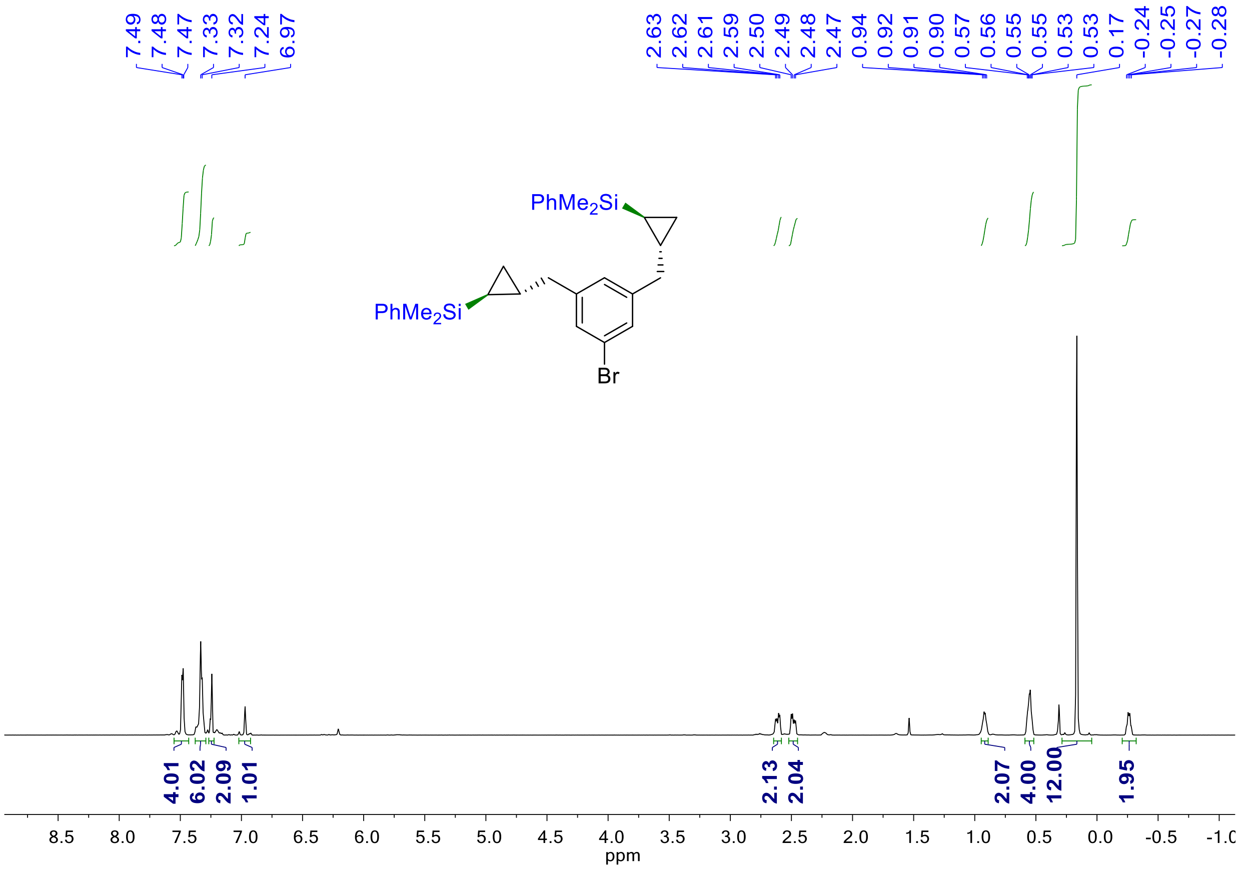

Supplementary Figure 130. ^{13}C and ^{29}Si-NMR spectra of ***anti*-3q**

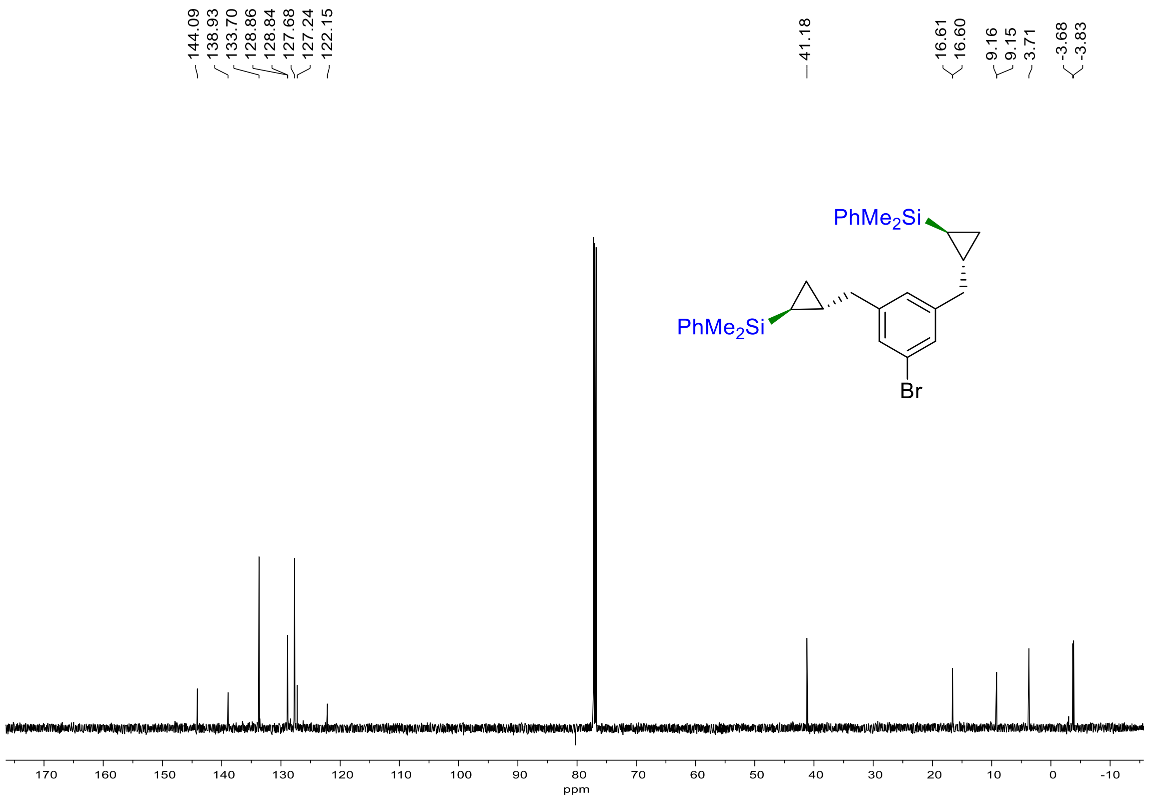

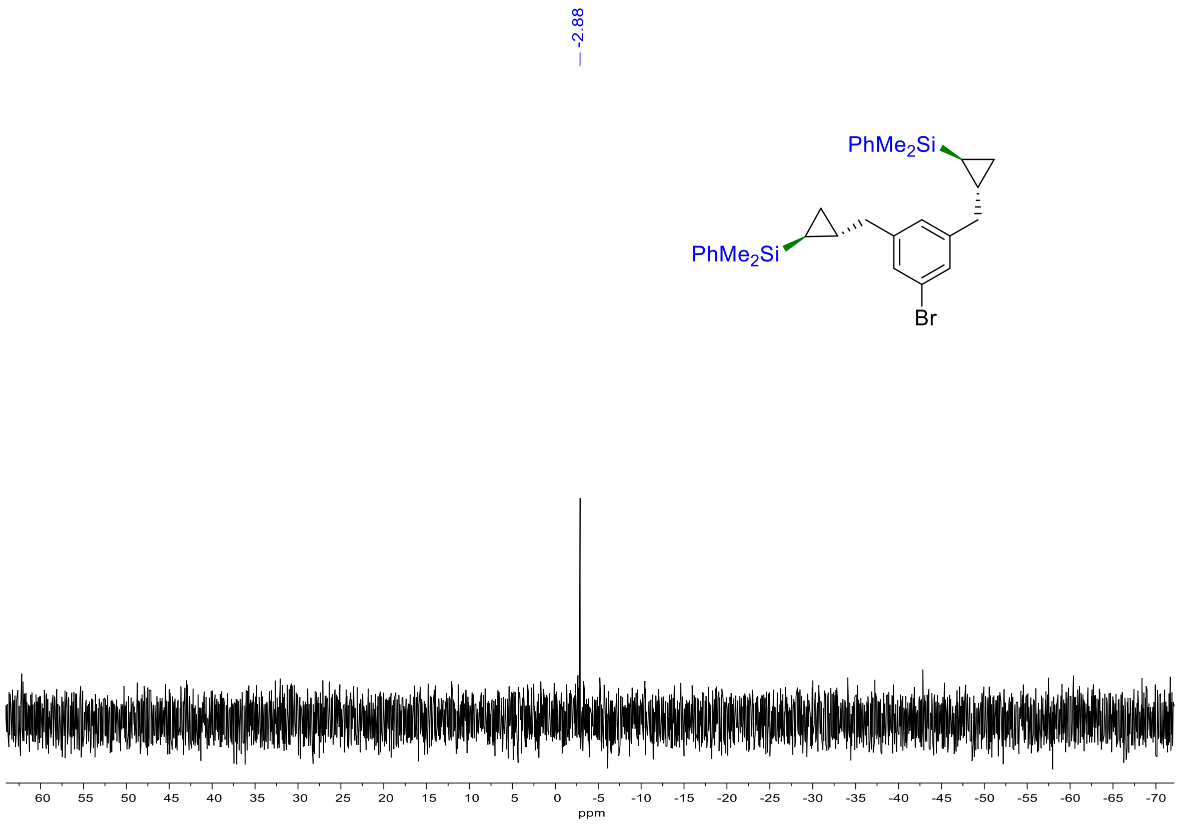

Supplementary Figure 131. ^{1}H and ^{13}C-NMR spectra of ***anti*-3r**

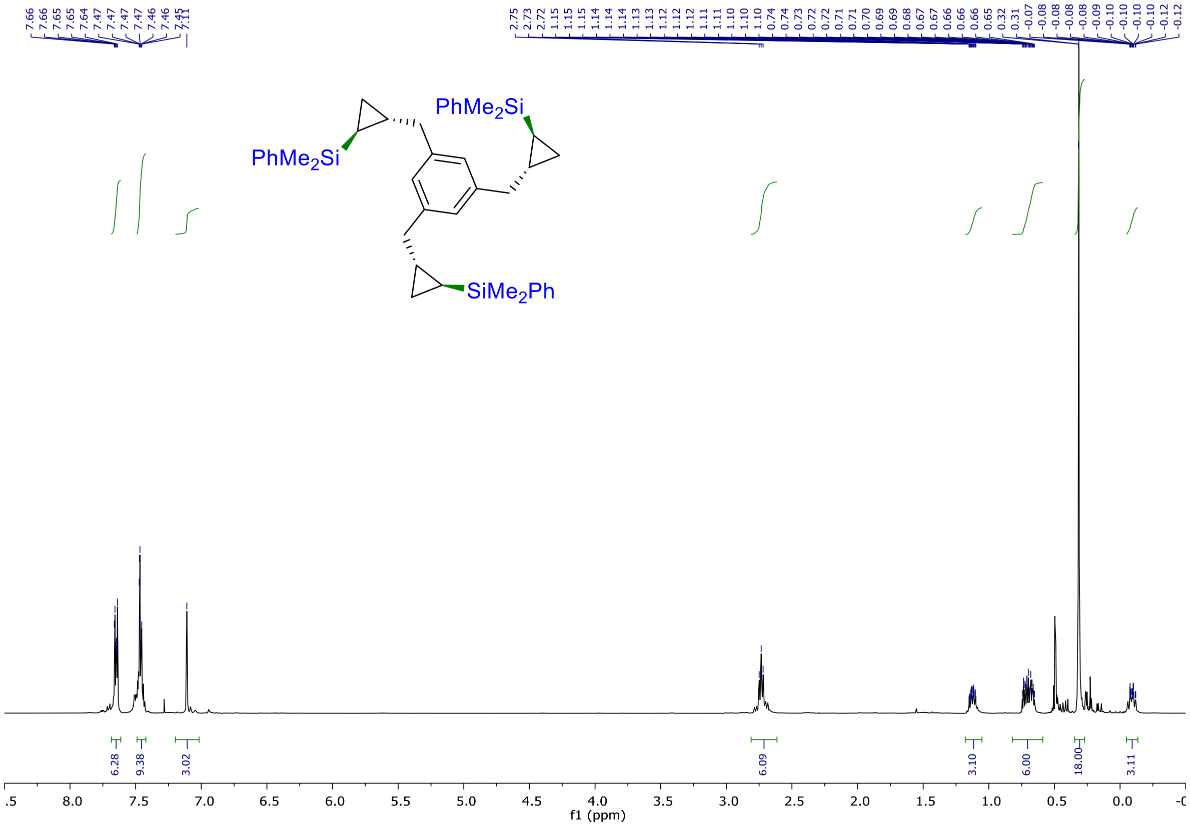

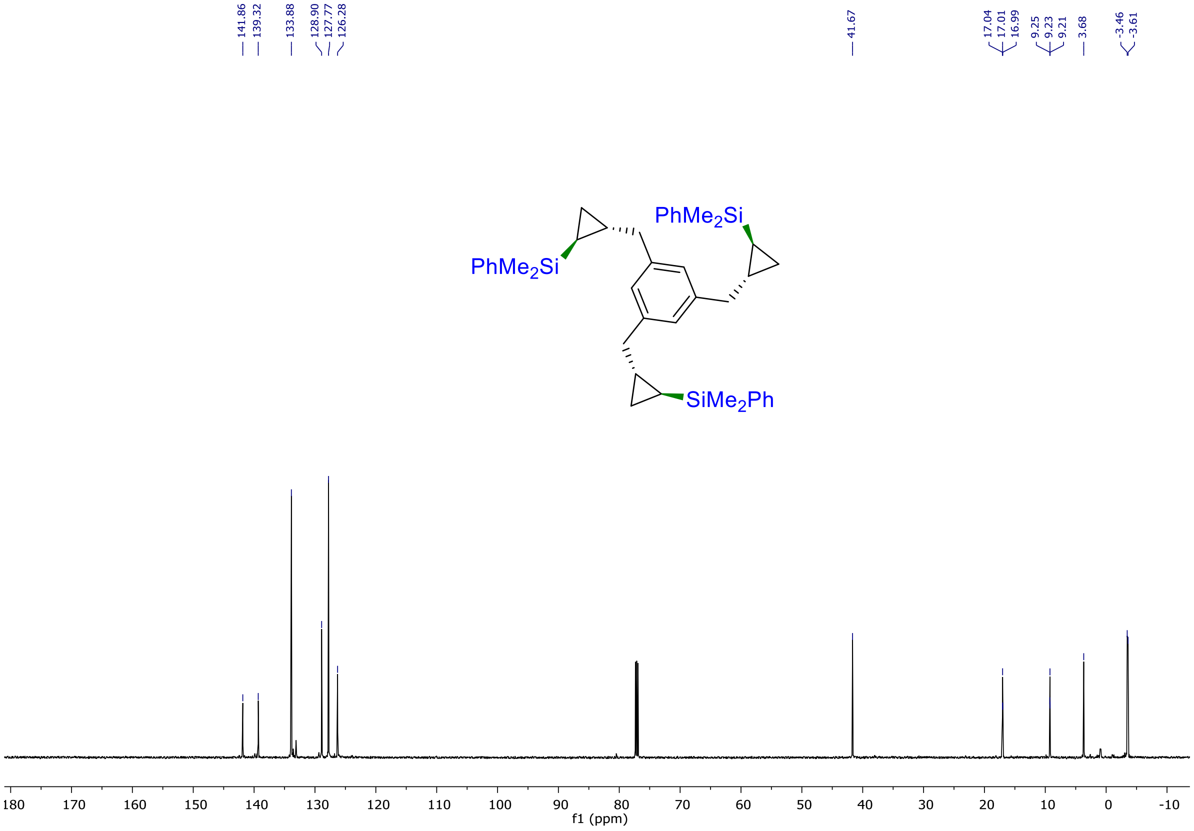

Supplementary Figure 132. ^{29}Si-NMR spectrum of ***anti*-3r**

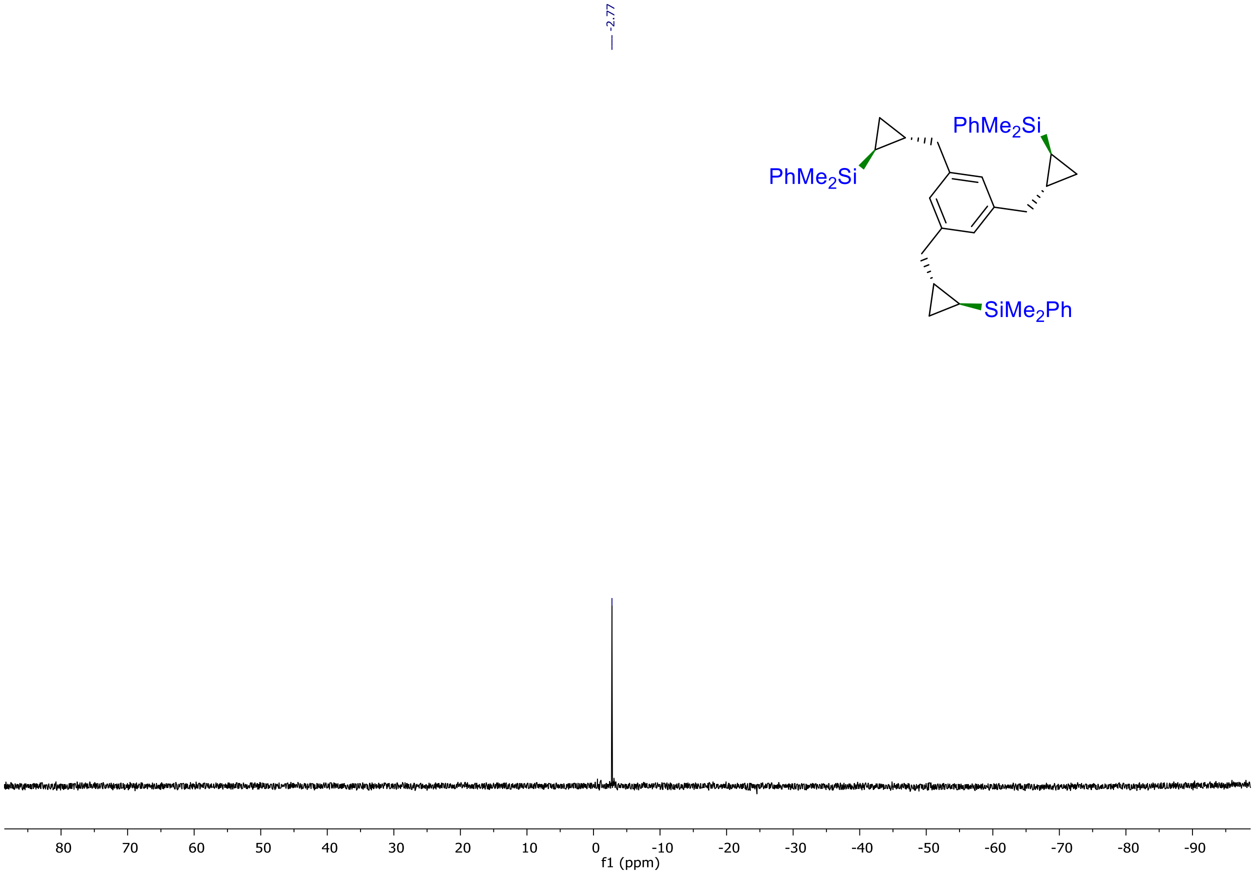

Supplementary Figure 133. ^{1}H-NMR spectrum of **4**

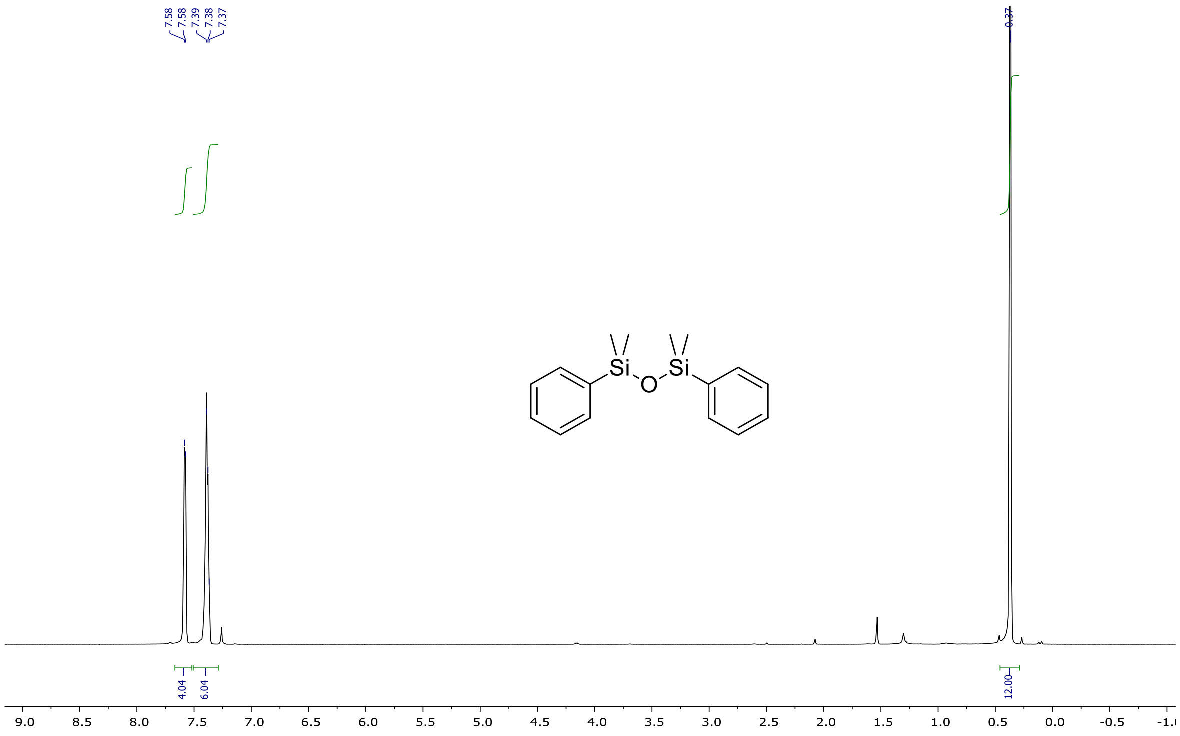

Supplementary Figure 134. ^{13}C and ^{29}Si-NMR spectra of **4**

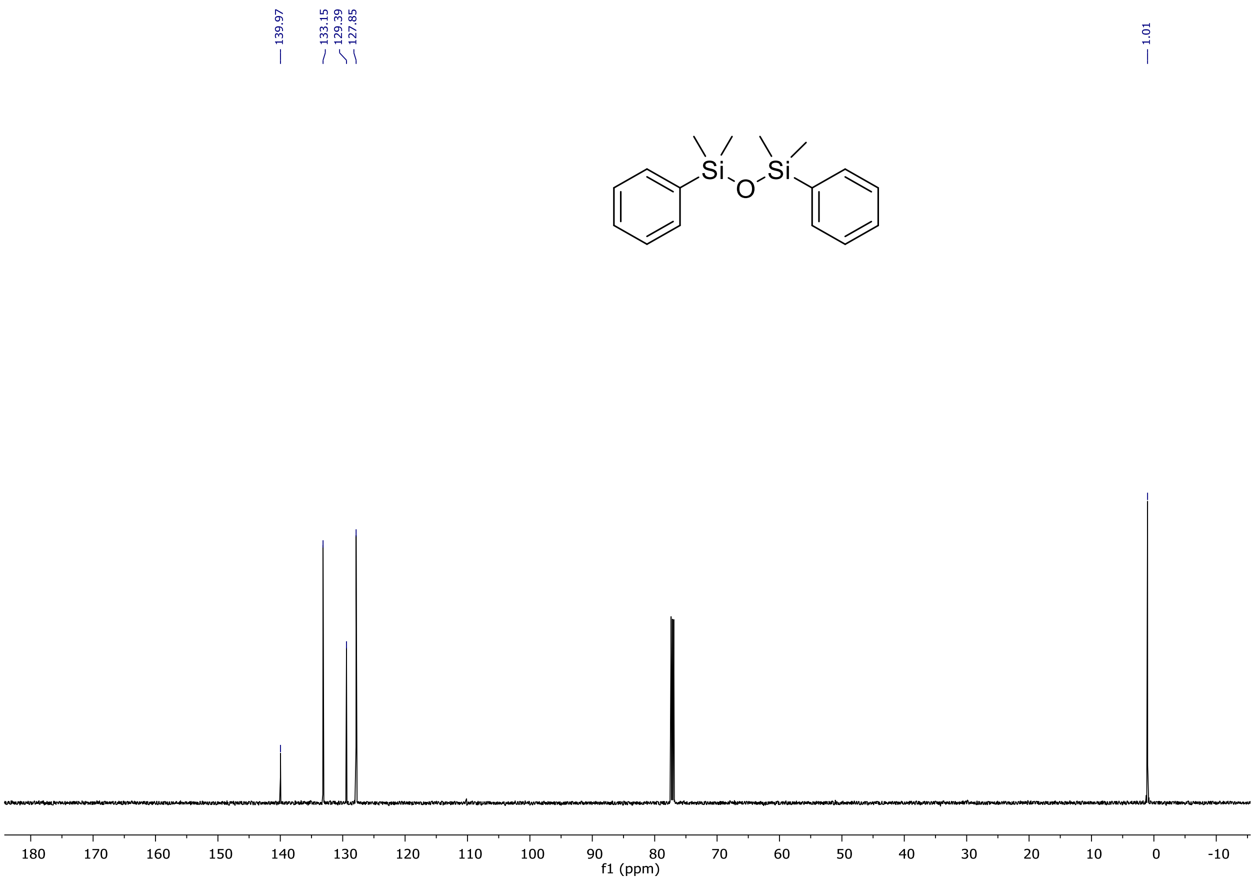

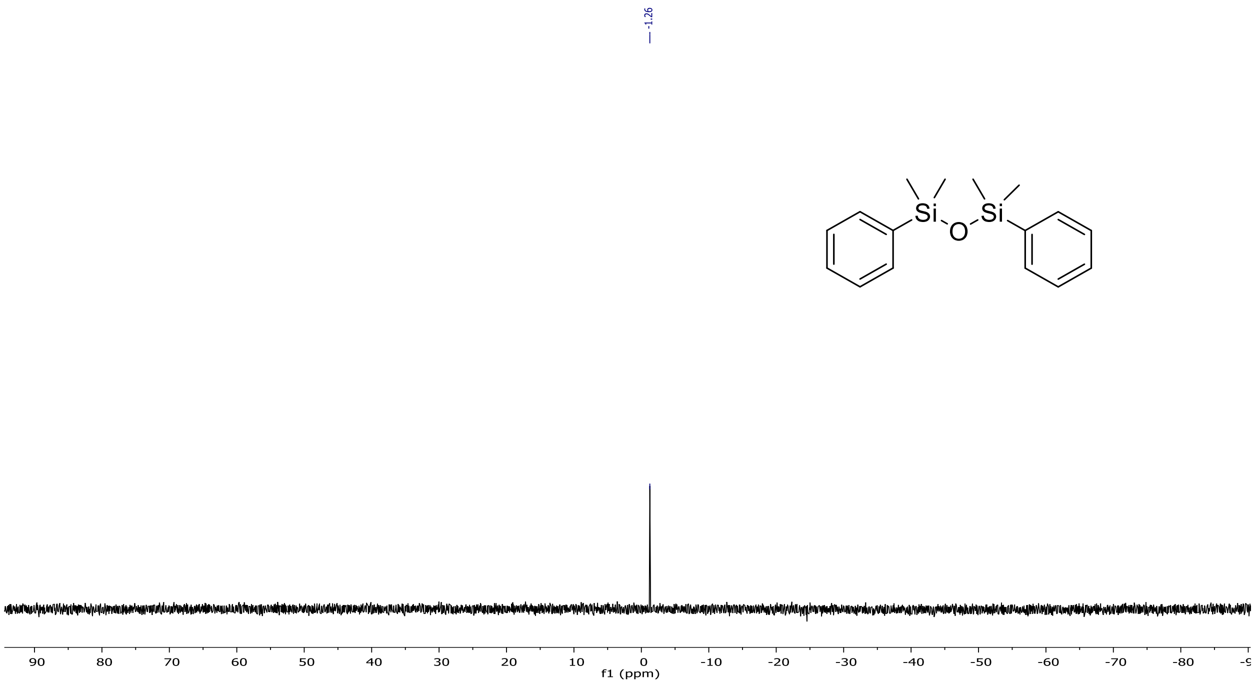

Supplementary Figure 135. ¹H and ¹³C-NMR spectra of *Z*-**5**

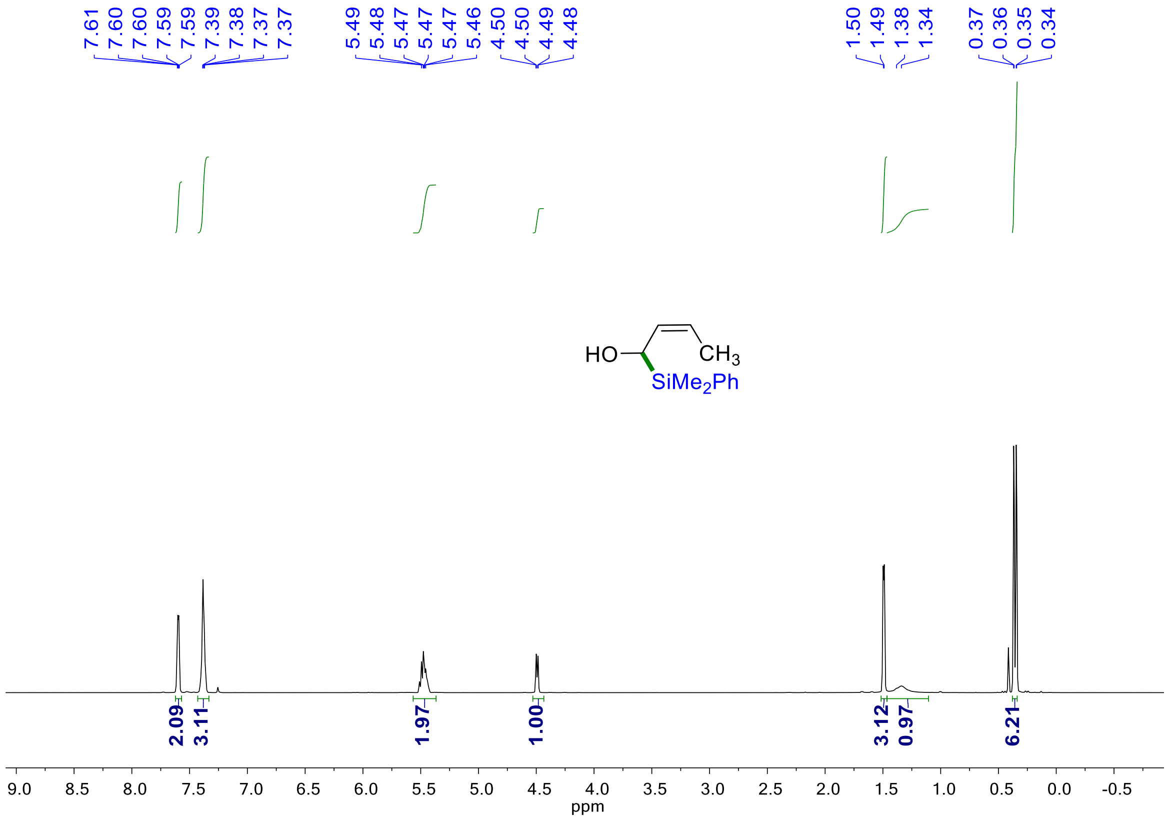

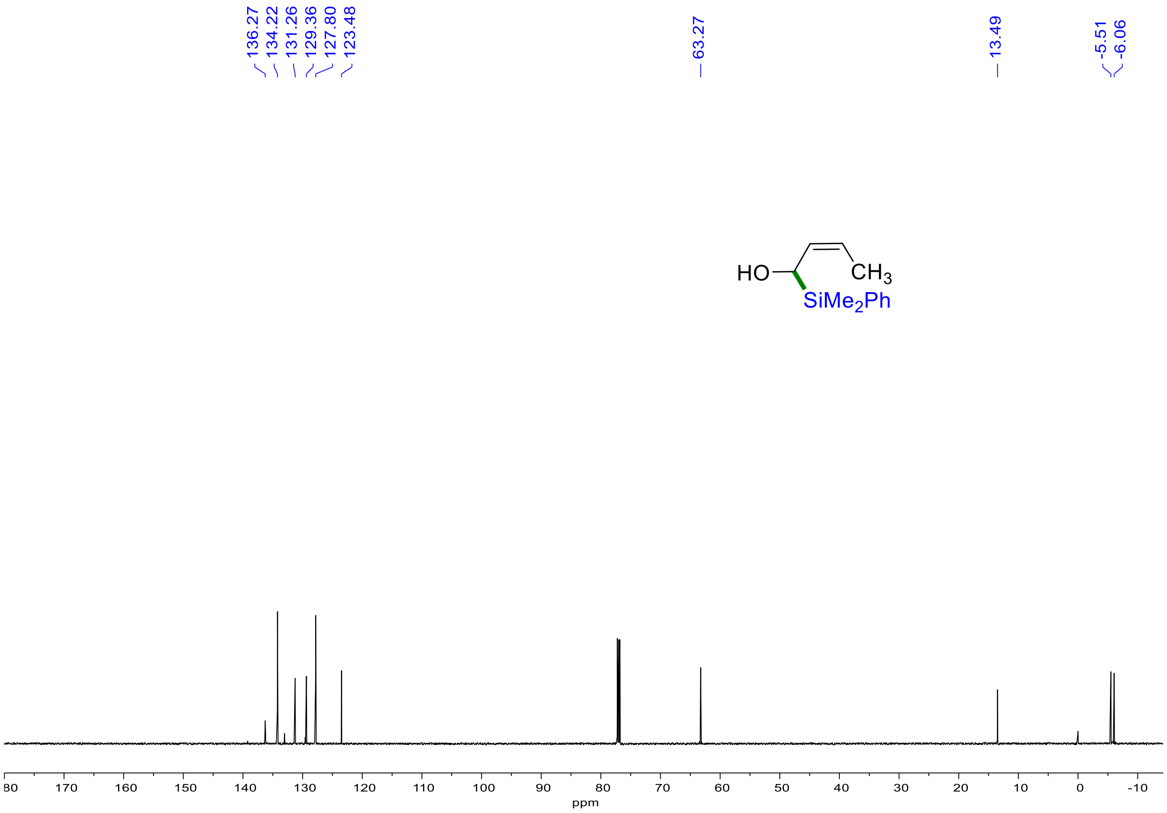

Supplementary Figure 136. ^{29}Si-NMR spectrum of *Z*-**5**

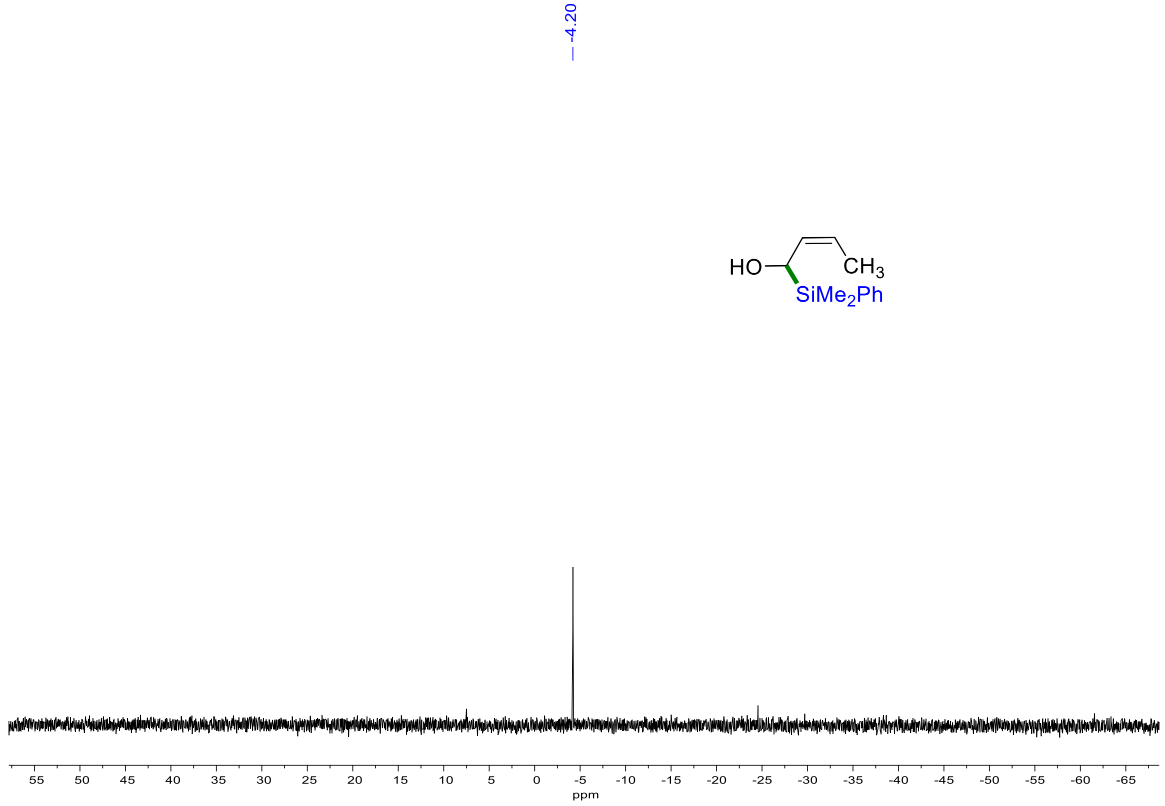

Supplementary Figure 137. ^{1}H-NMR spectrum of *Z*-**5'**-***d***$_2$

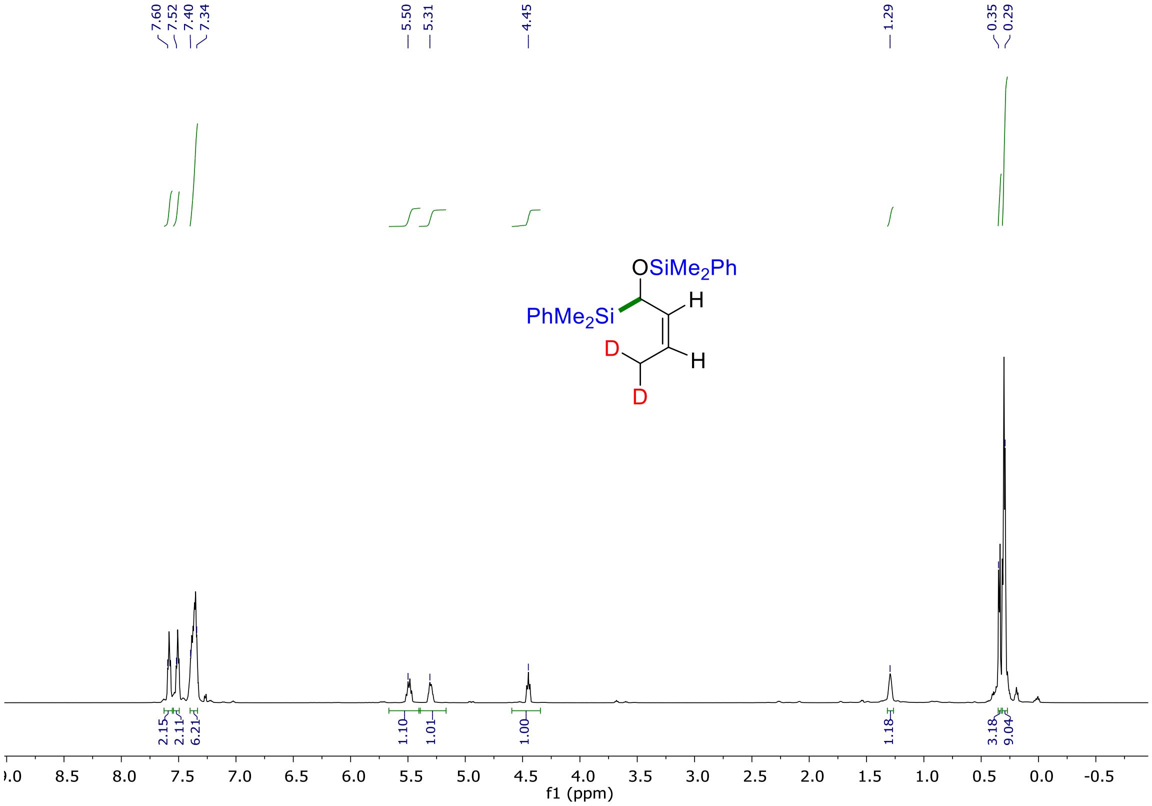

Supplementary Figure 138. ^{13}C and ^{29}Si-NMR spectra of Z-**5'**-$\boldsymbol{d_2}$

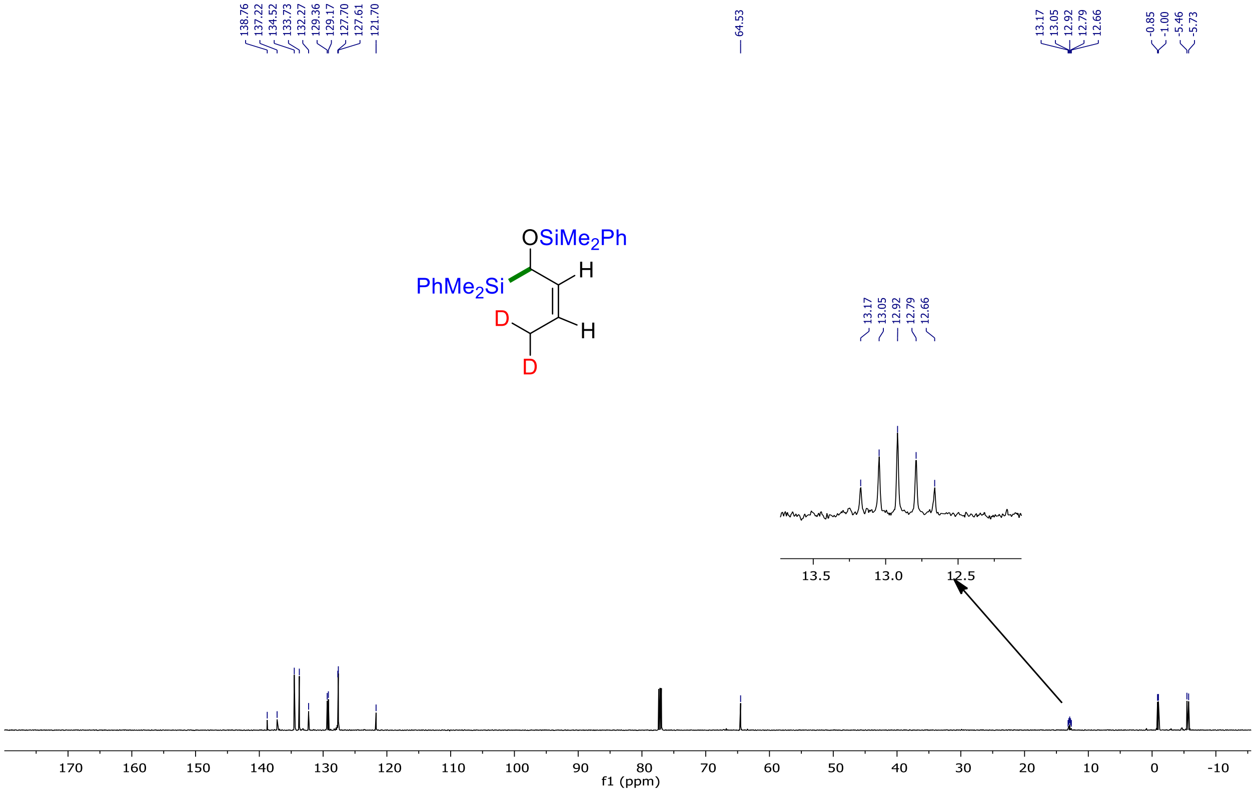

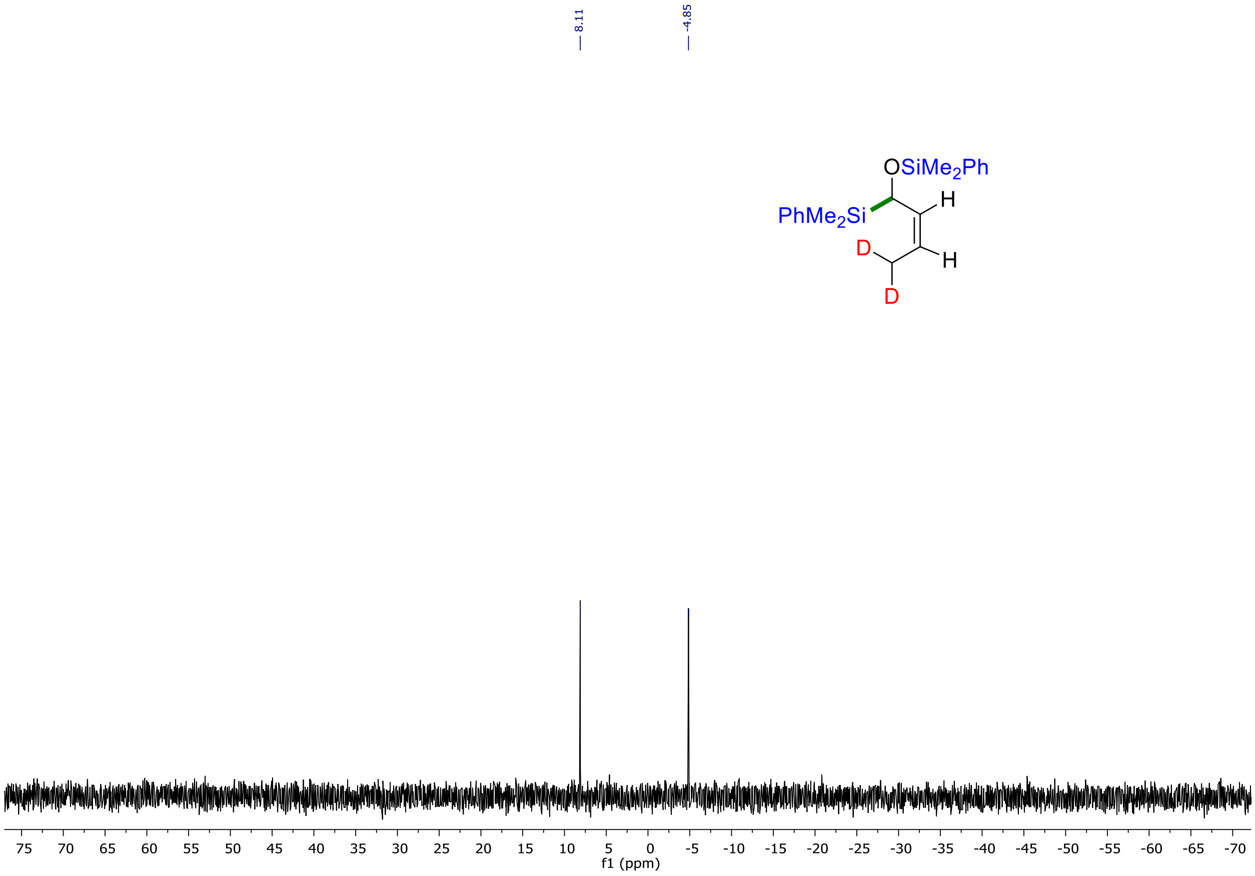

Supplementary Figure 139. ^{2}H-NMR spectrum of *Z*-**5'**-$\boldsymbol{d_2}$

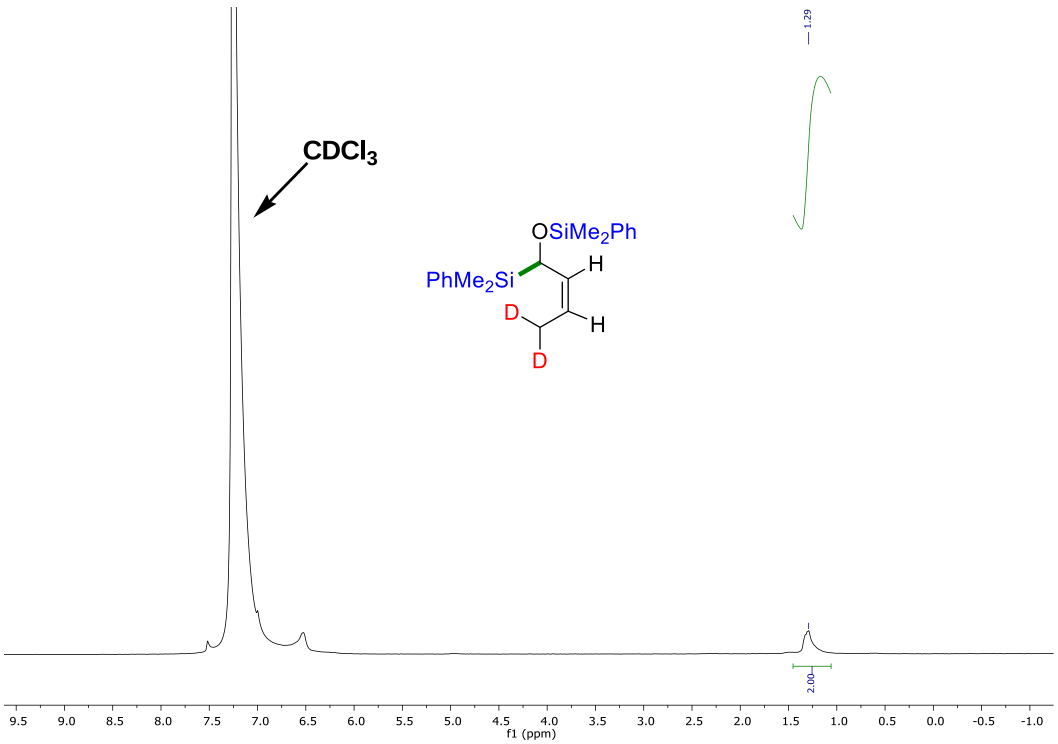

Supplementary Figure 140. ^{1}H-NMR spectrum of *Z*-**5''**

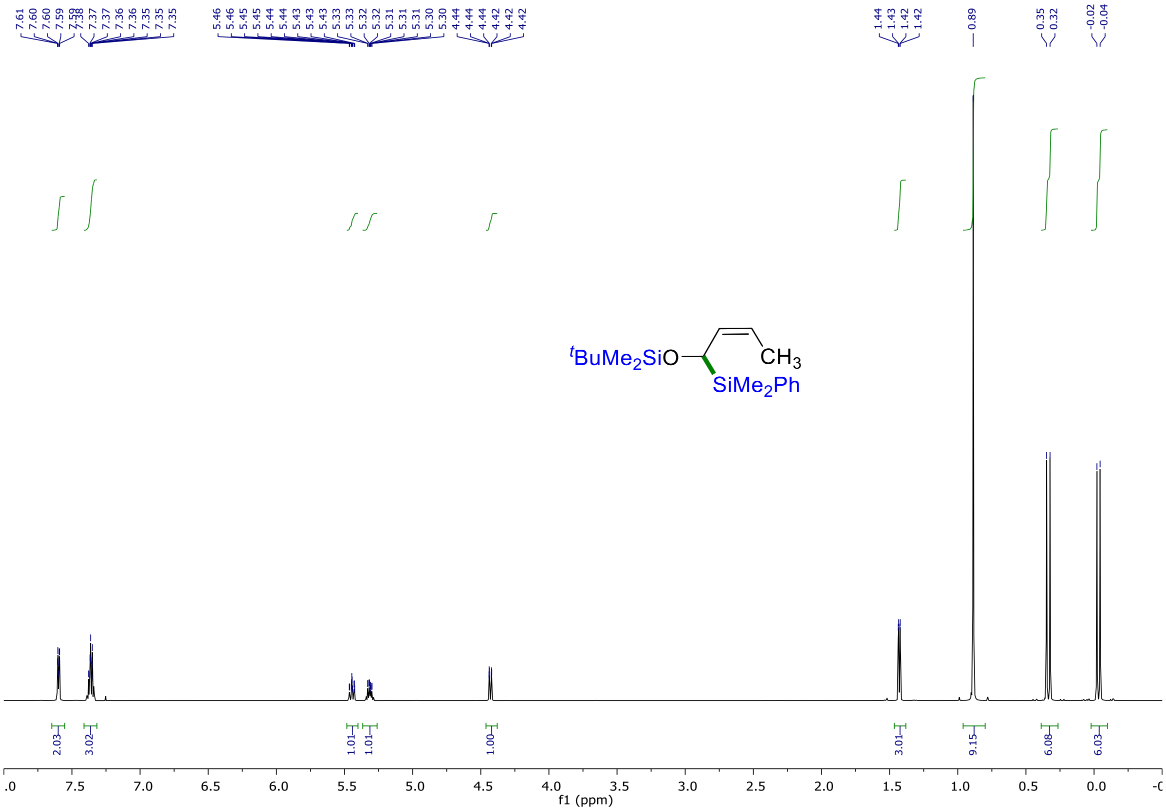

Supplementary Figure 141. ^{13}C and ^{29}Si-NMR spectra of *Z*-**5''**

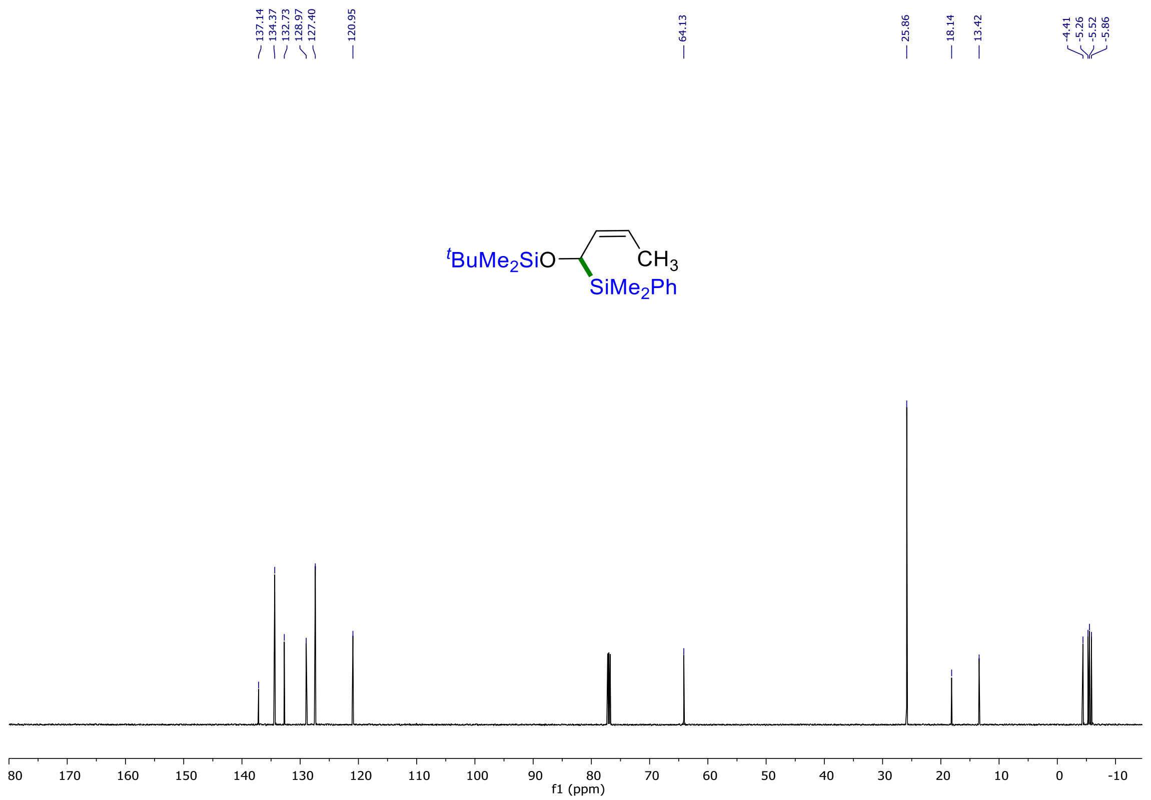

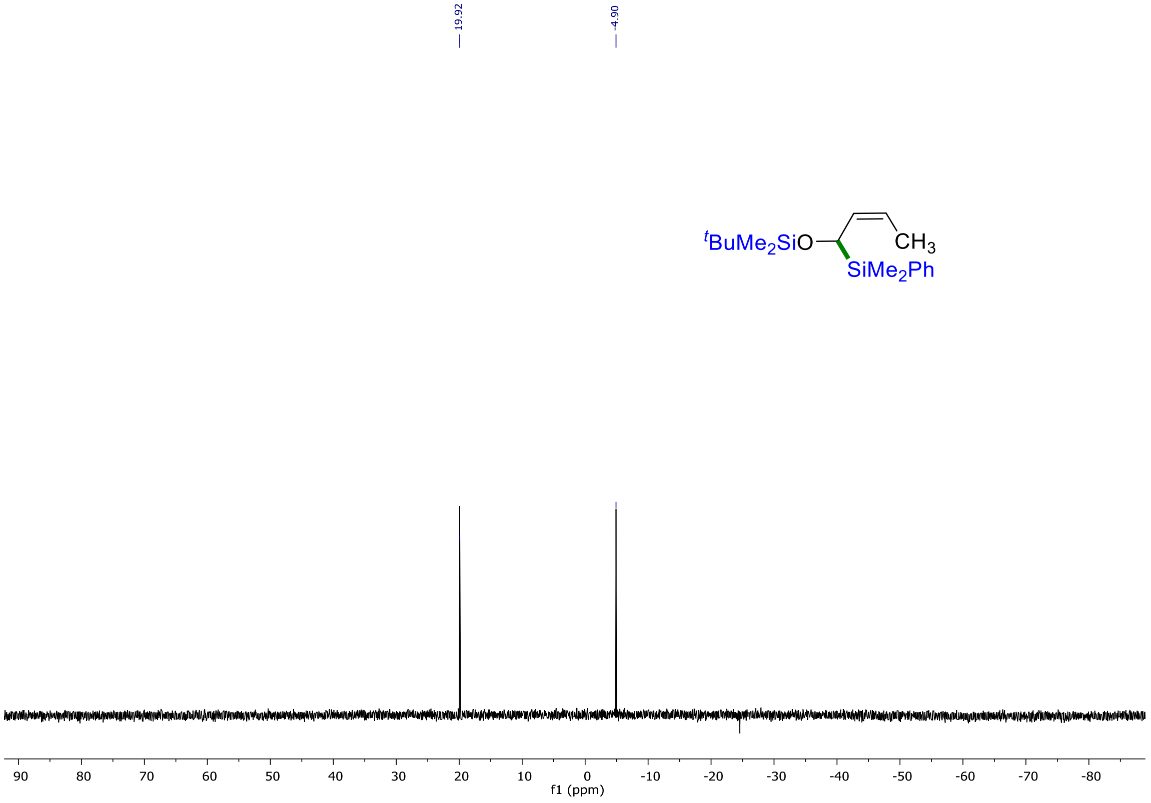

Supplementary Figure 142. ^{1}H and ^{13}C-NMR spectra of *Z*-**6**

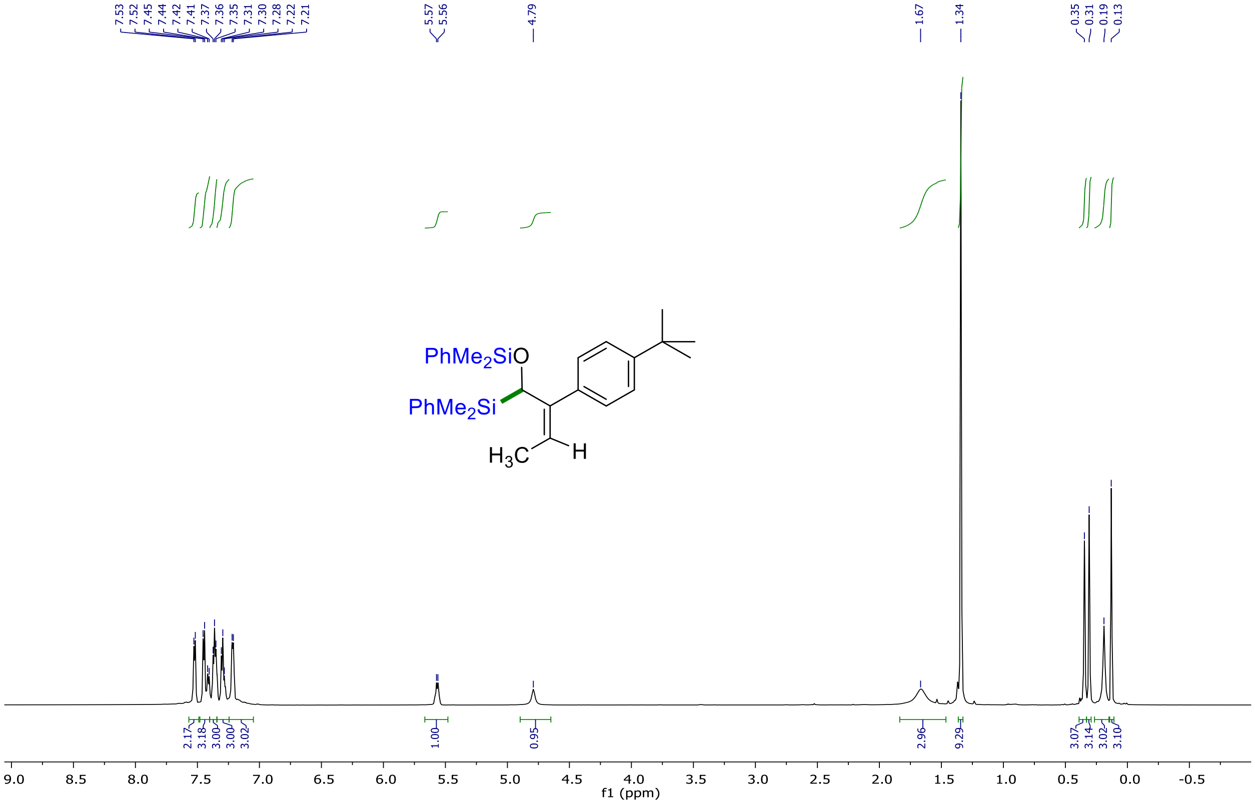

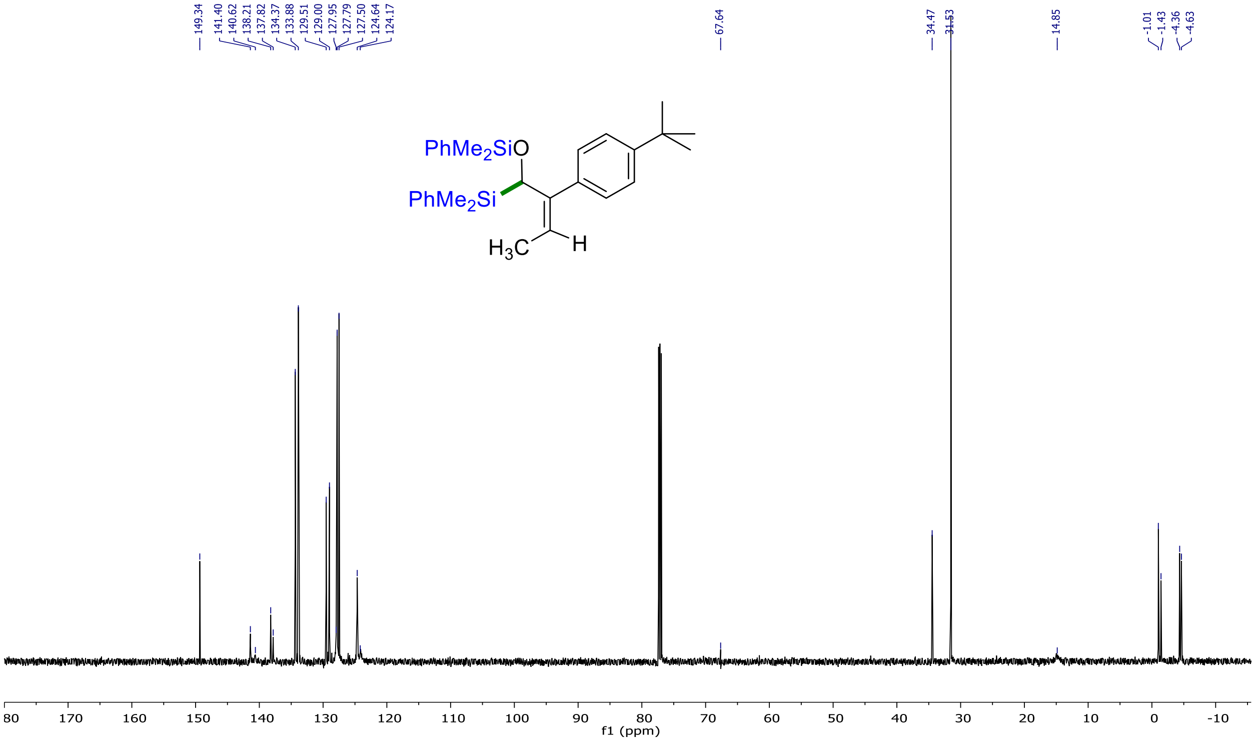

Supplementary Figure 143. ^{29}Si-NMR spectrum of *Z*-**6**

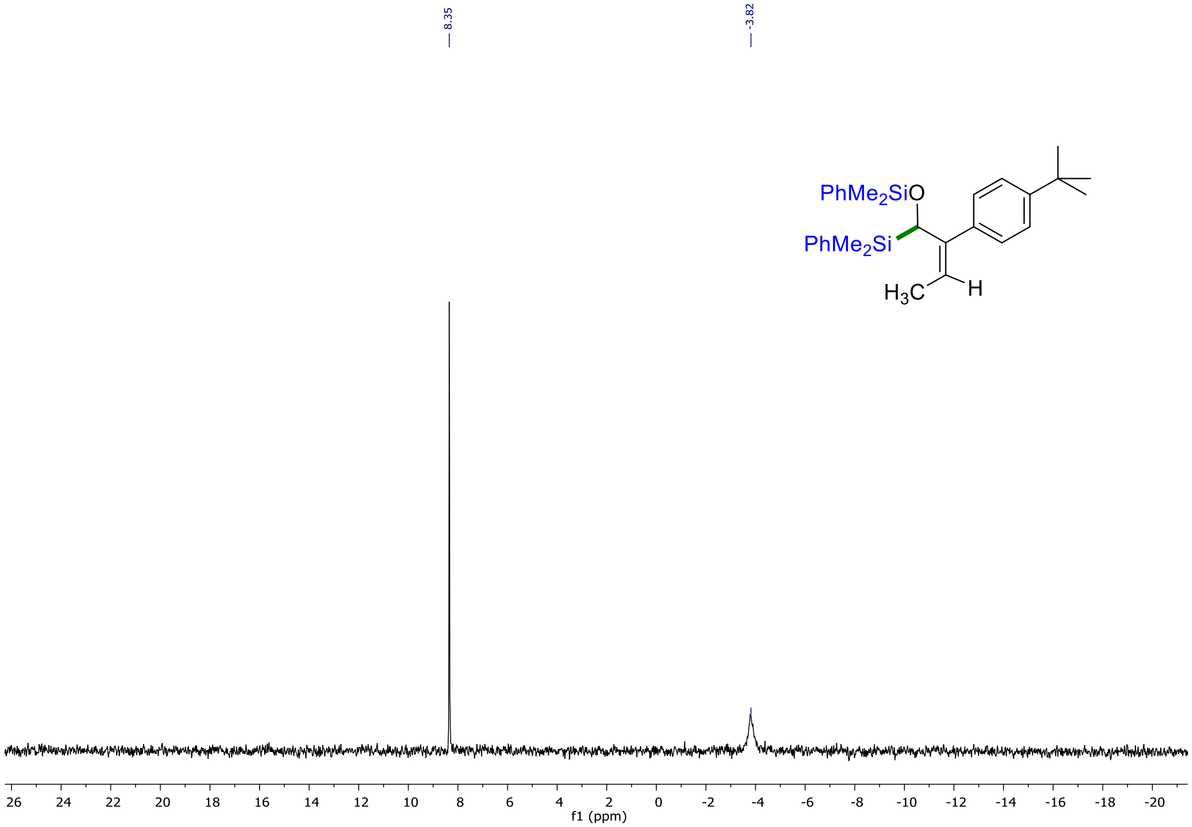

Supplementary Figure 144. ^{1}H-NMR spectrum of **7**

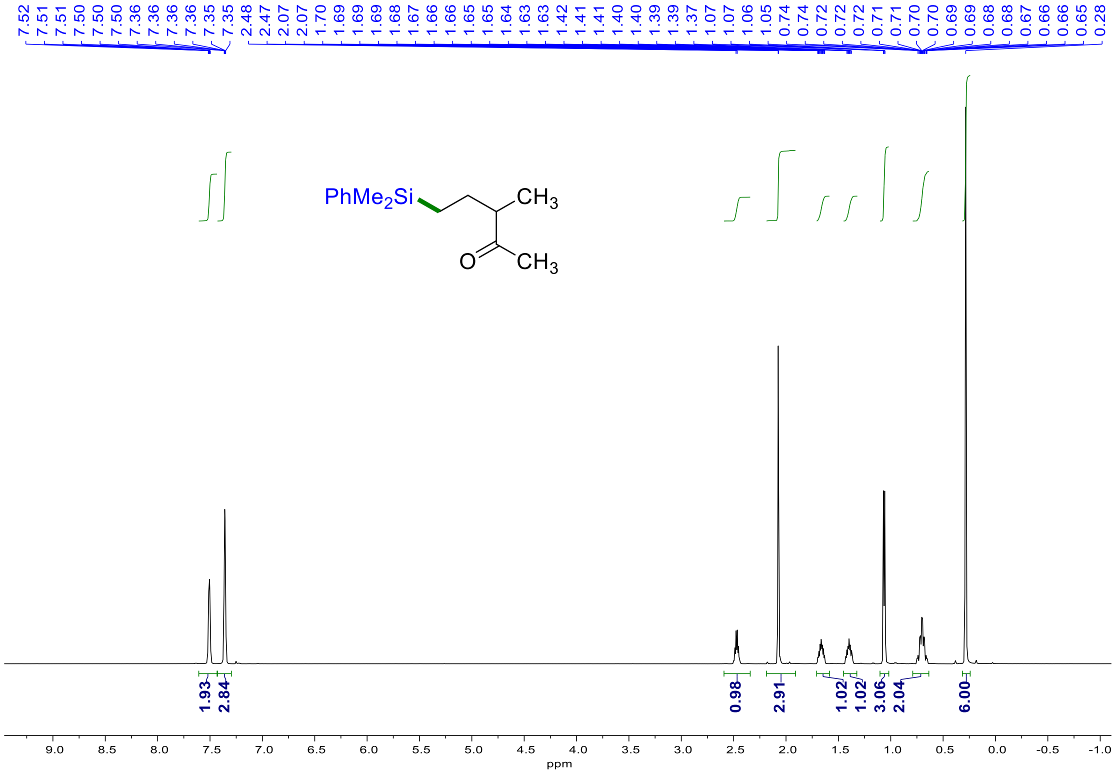

Supplementary Figure 145. ^{13}C and ^{29}Si-NMR spectra of **7**

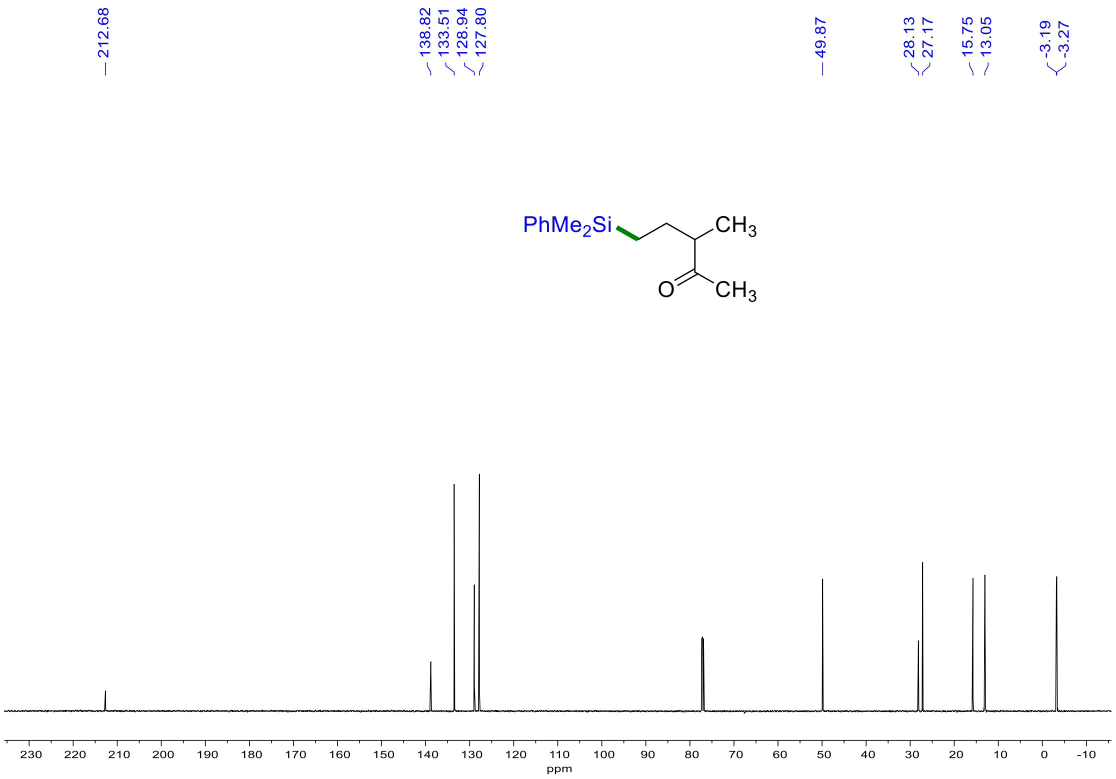

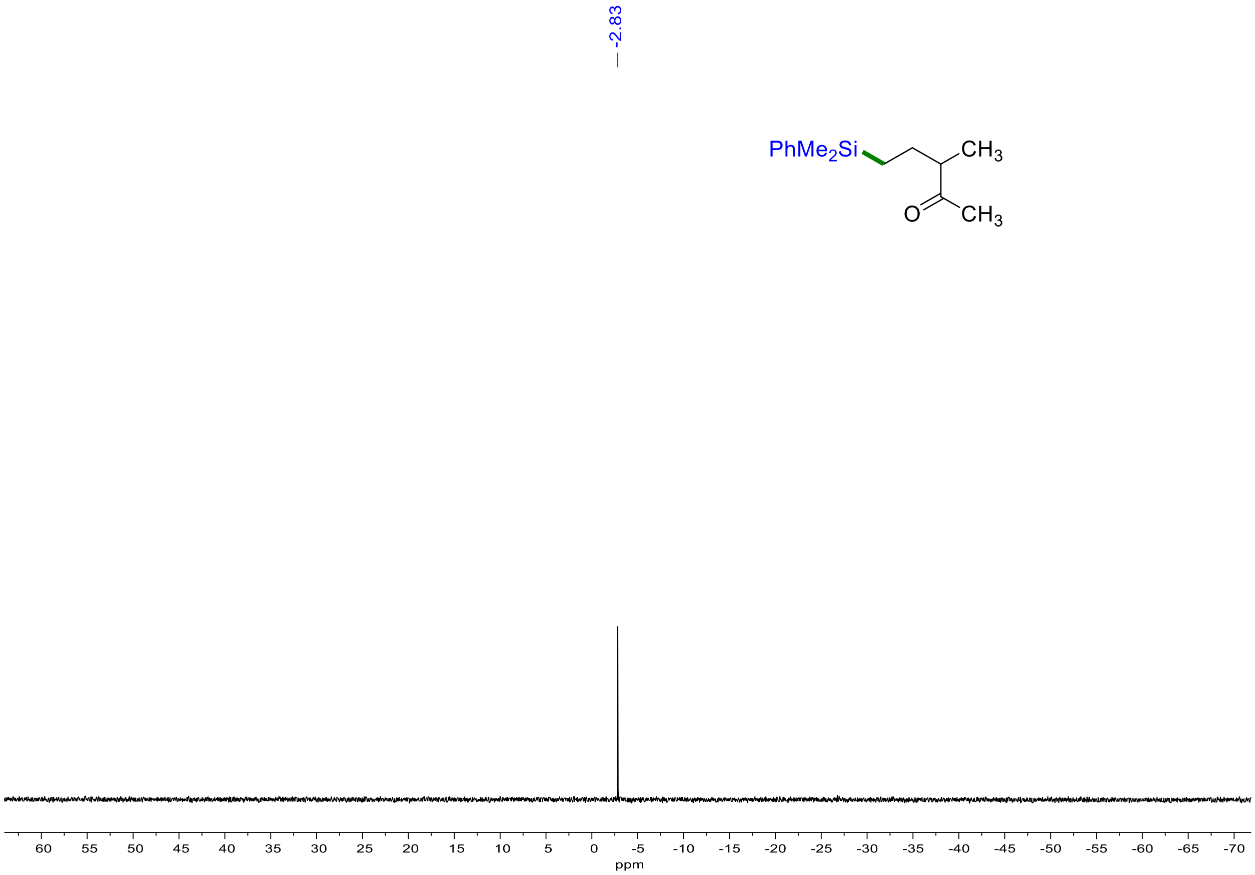

Supplementary Figure 146. ^{1}H and ^{13}C-NMR spectra of **7-*d*$_2$**

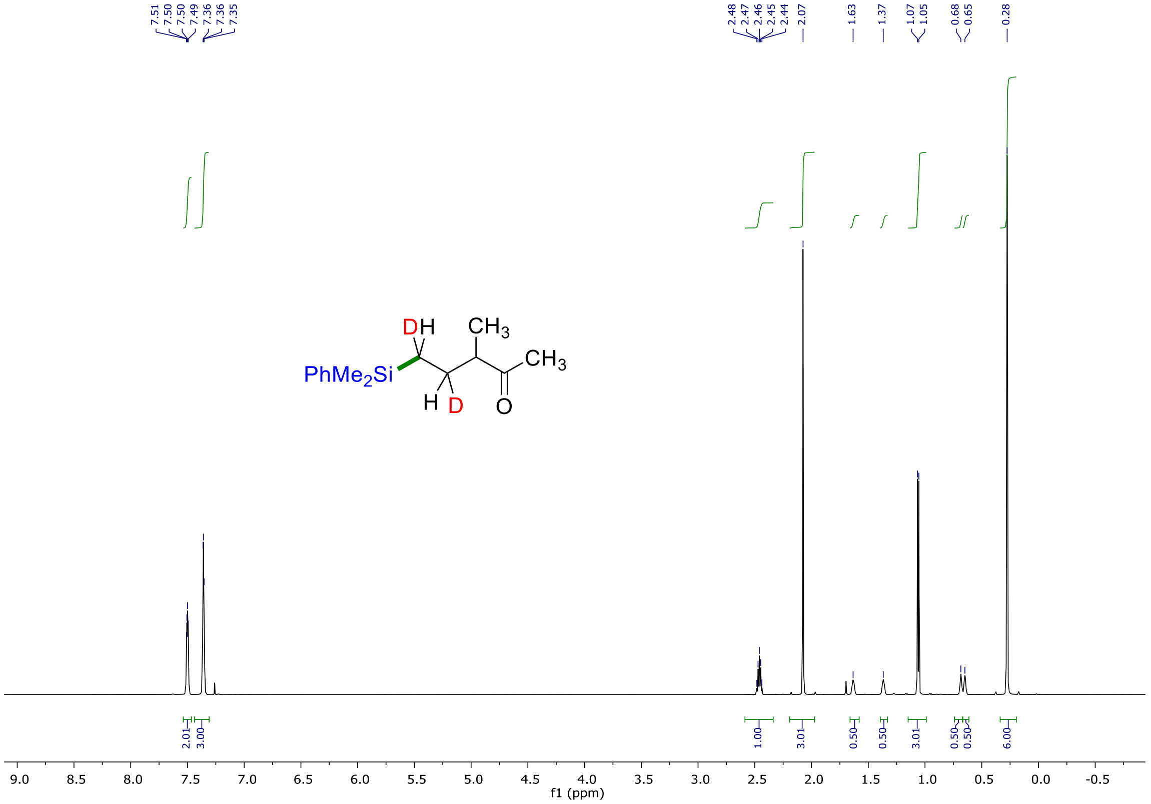

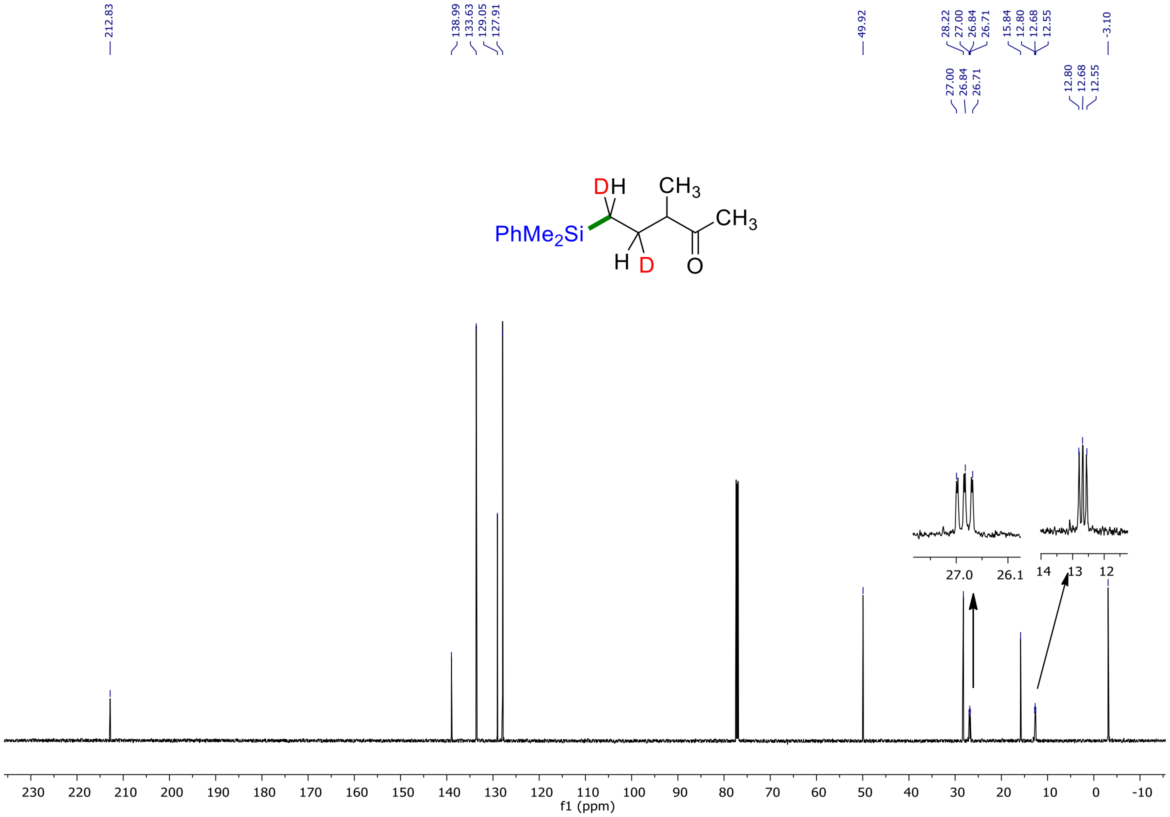

Supplementary Figure 147. ^{29}Si and ^{2}H-NMR spectra of **7-*d*$_2$**

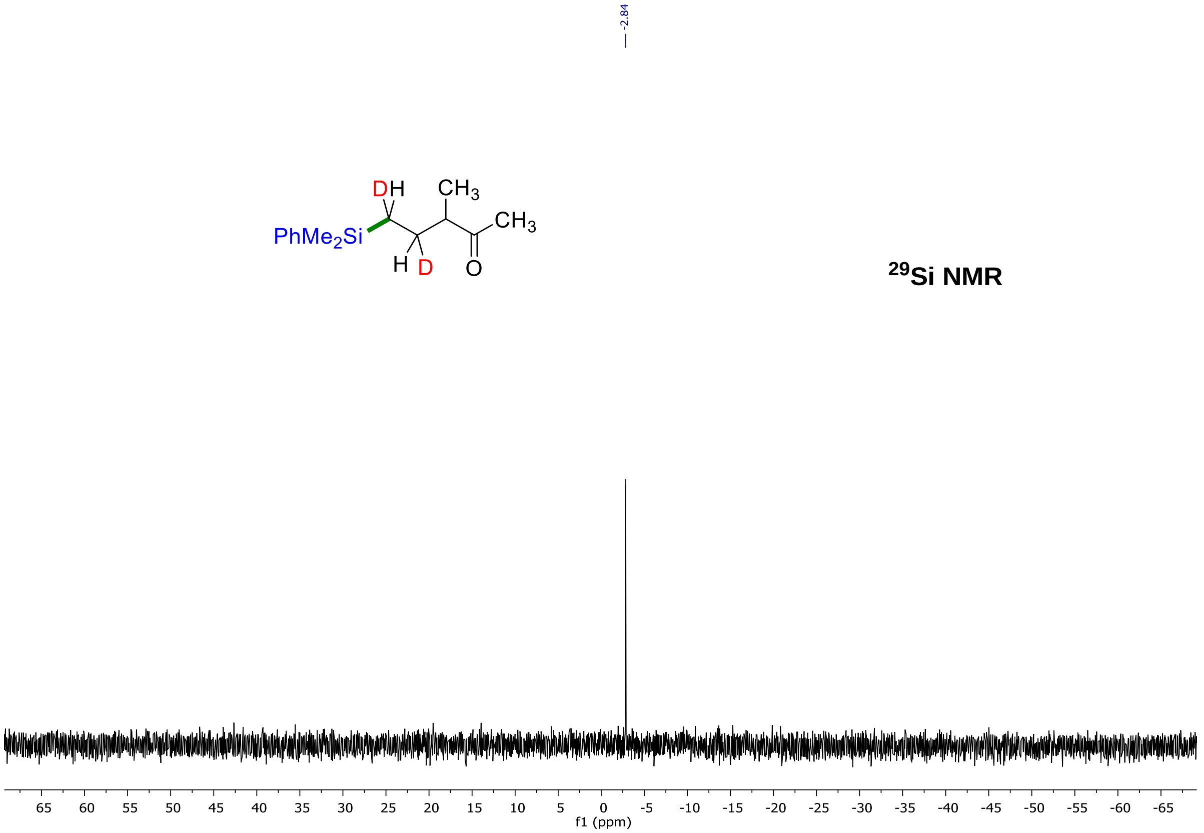

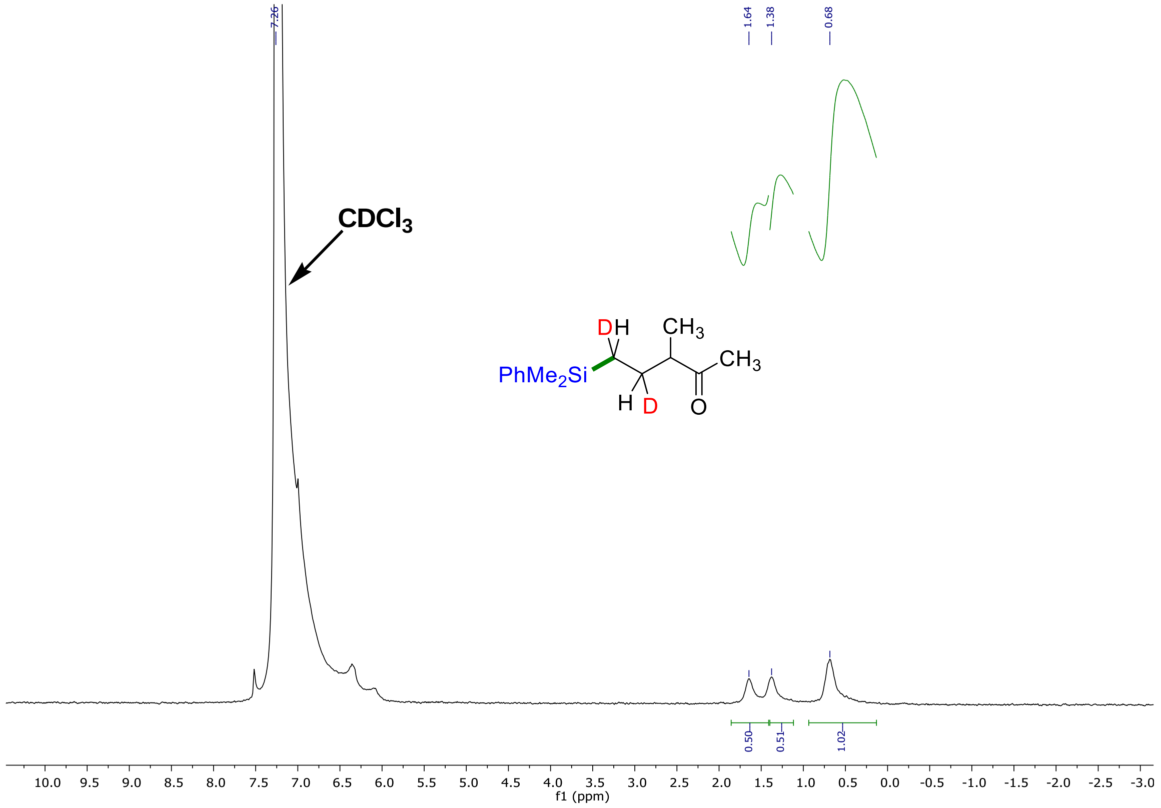

Supplementary Figure 148. ^{1}H and ^{13}C-NMR spectra of **8**

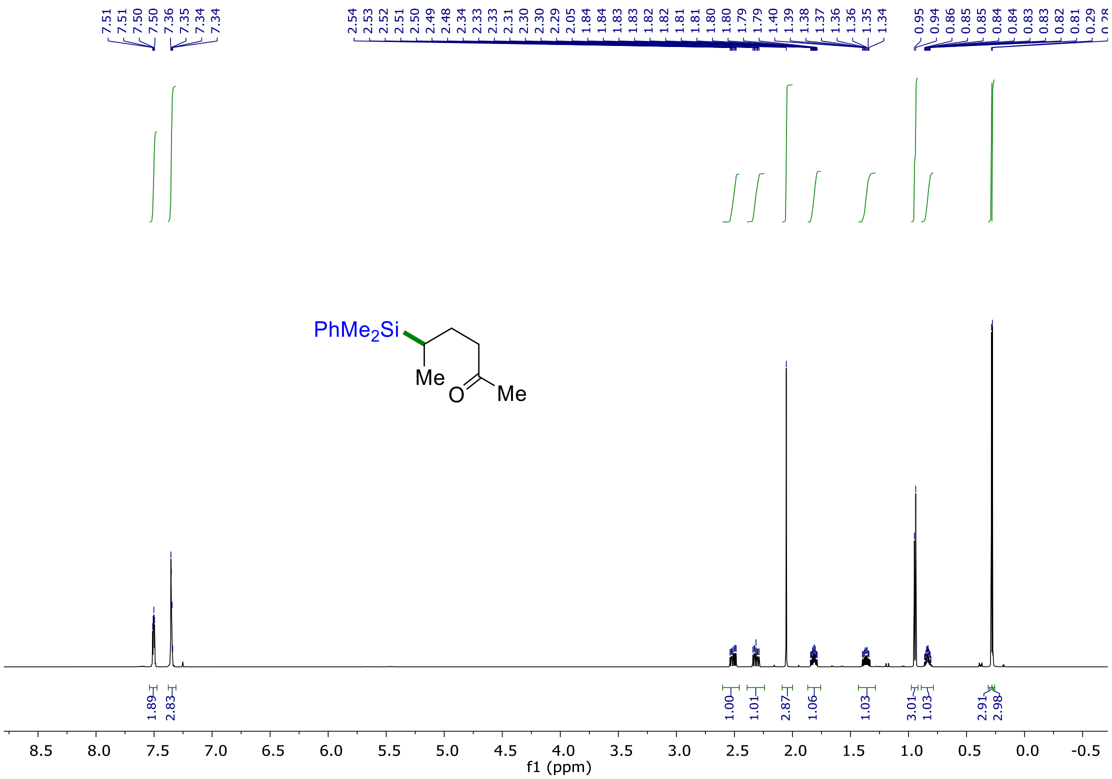

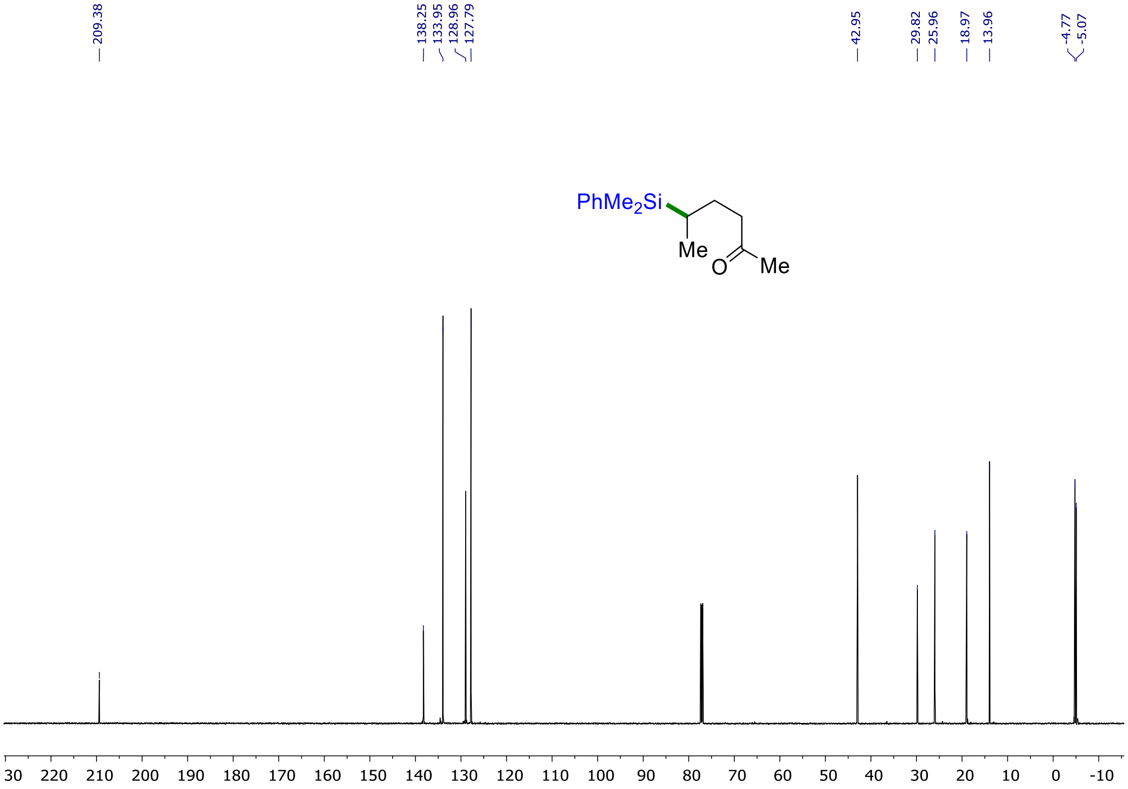

Supplementary Figure 149. ^{29}Si-NMR spectrum of **8**

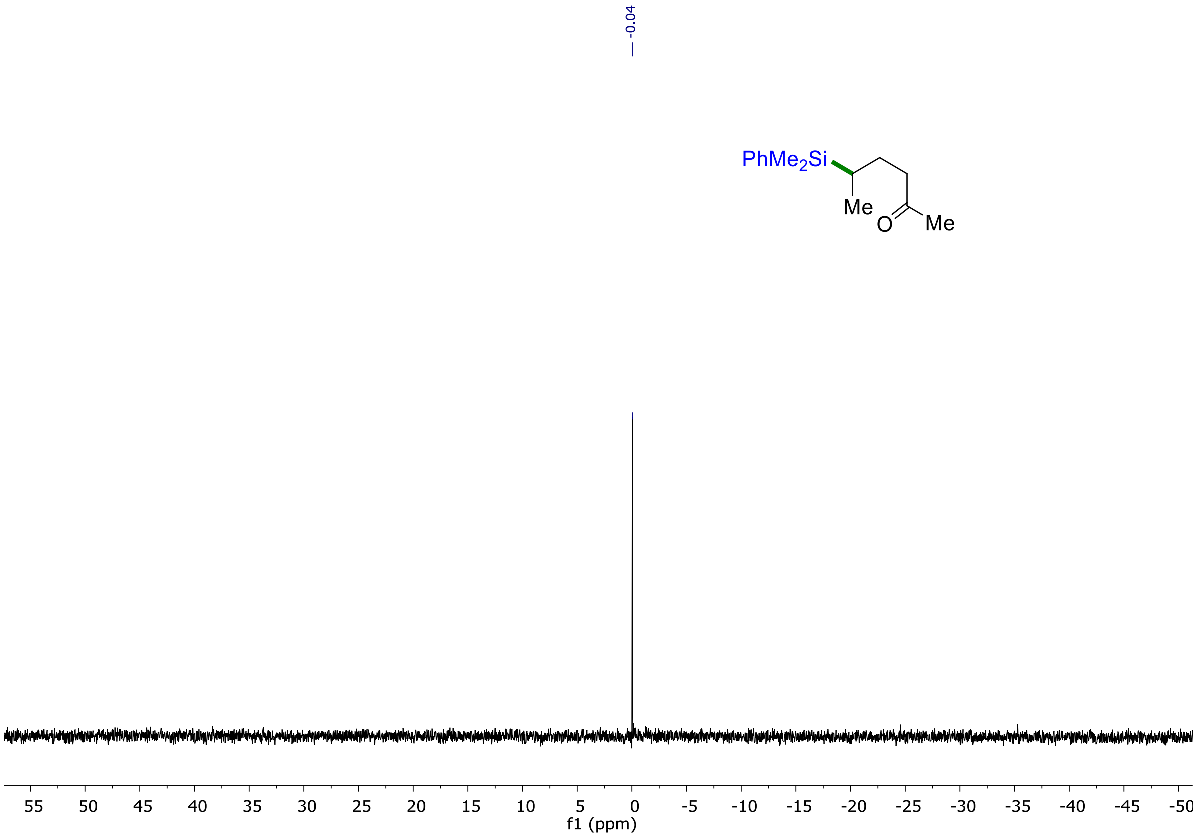

Supplementary Figure 150. ^{1}H-NMR spectrum of **9**

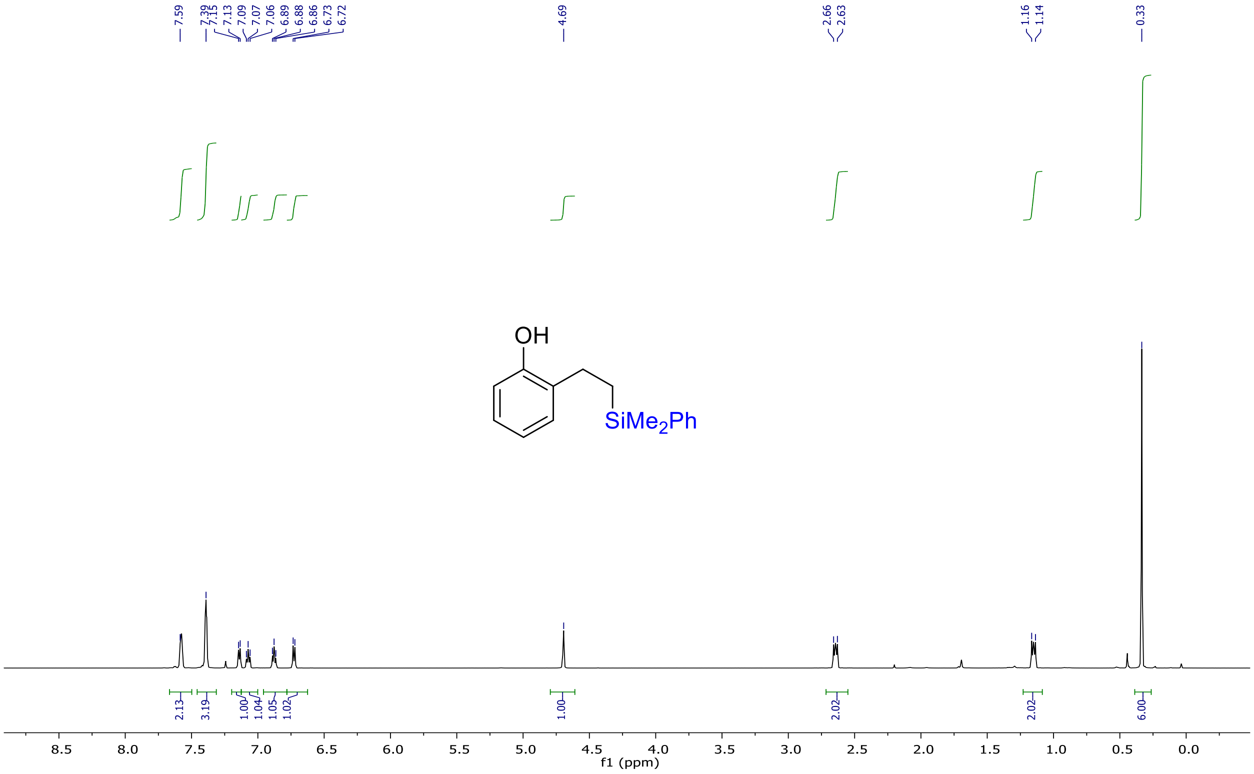

Supplementary Figure 151. ^{13}C and ^{29}Si-NMR spectra of **9**

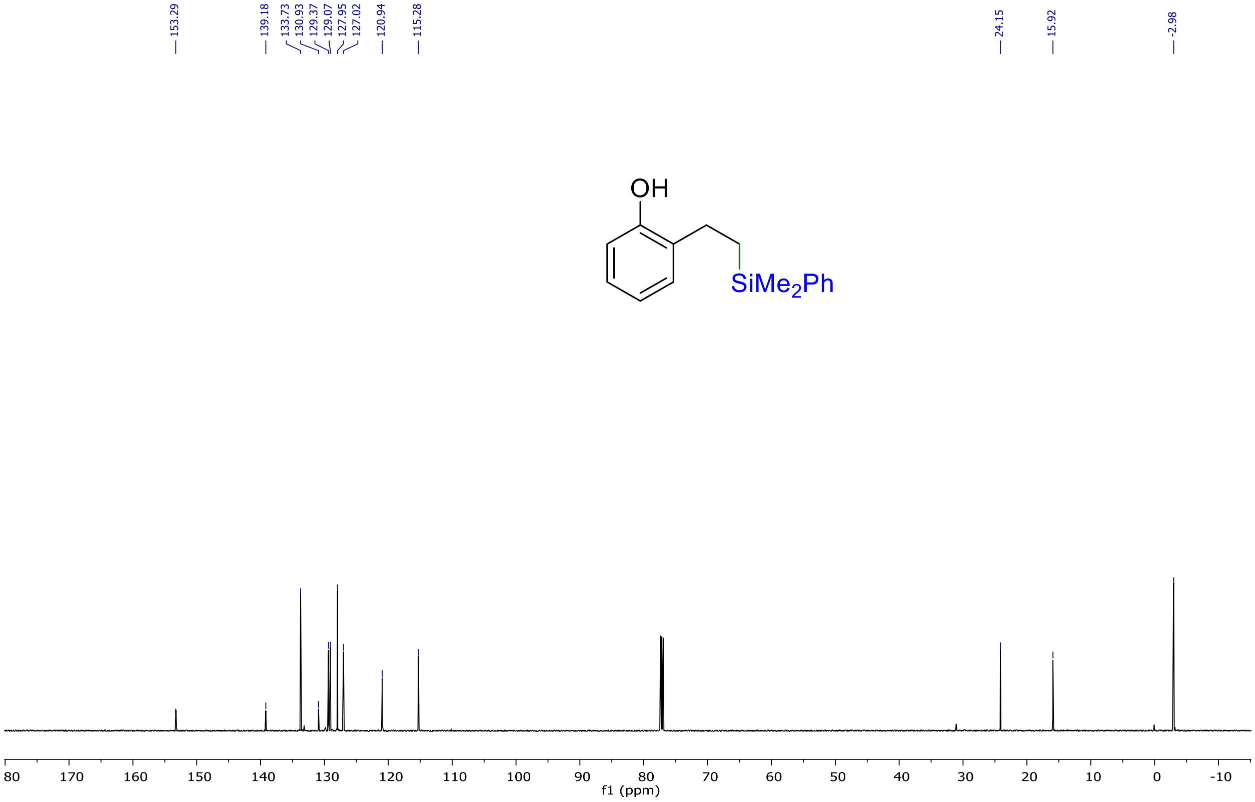

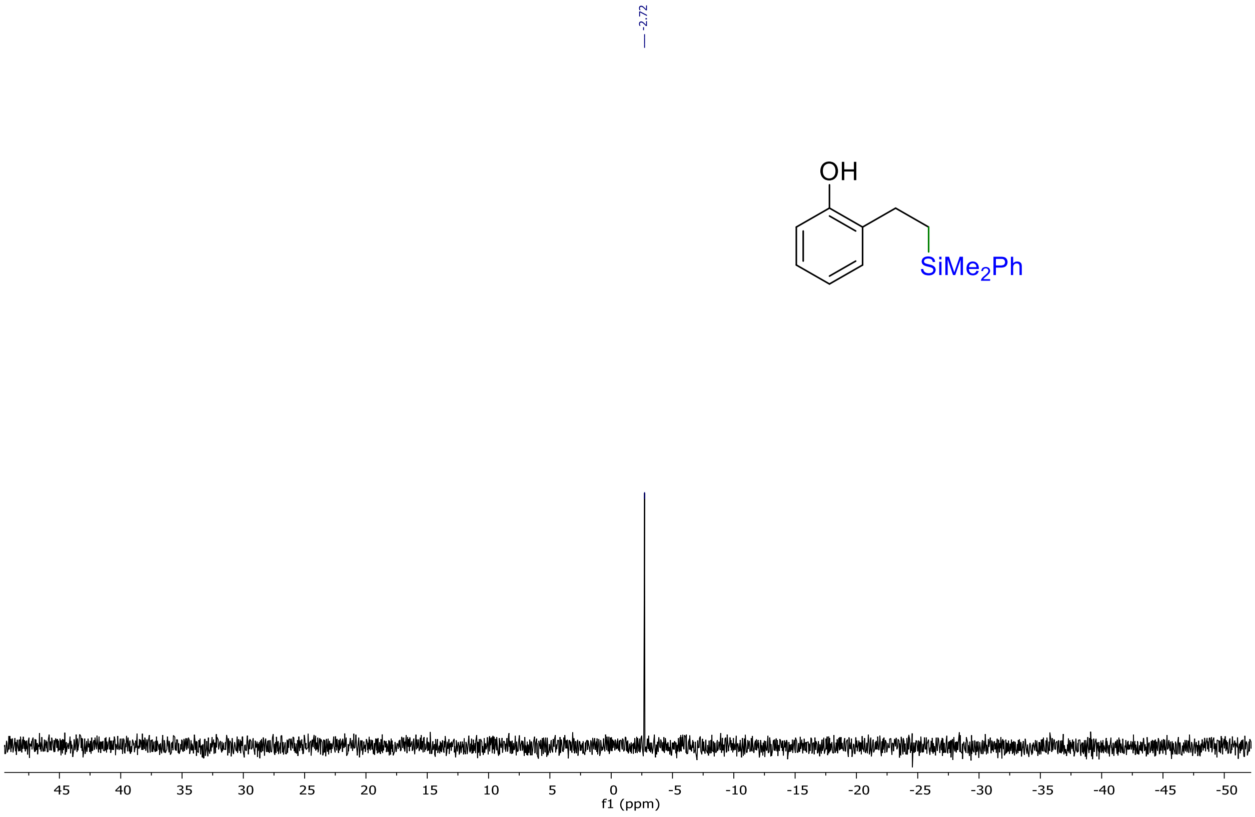

Supplementary Figure 152. ^{1}H and ^{13}C-NMR spectra of **10**

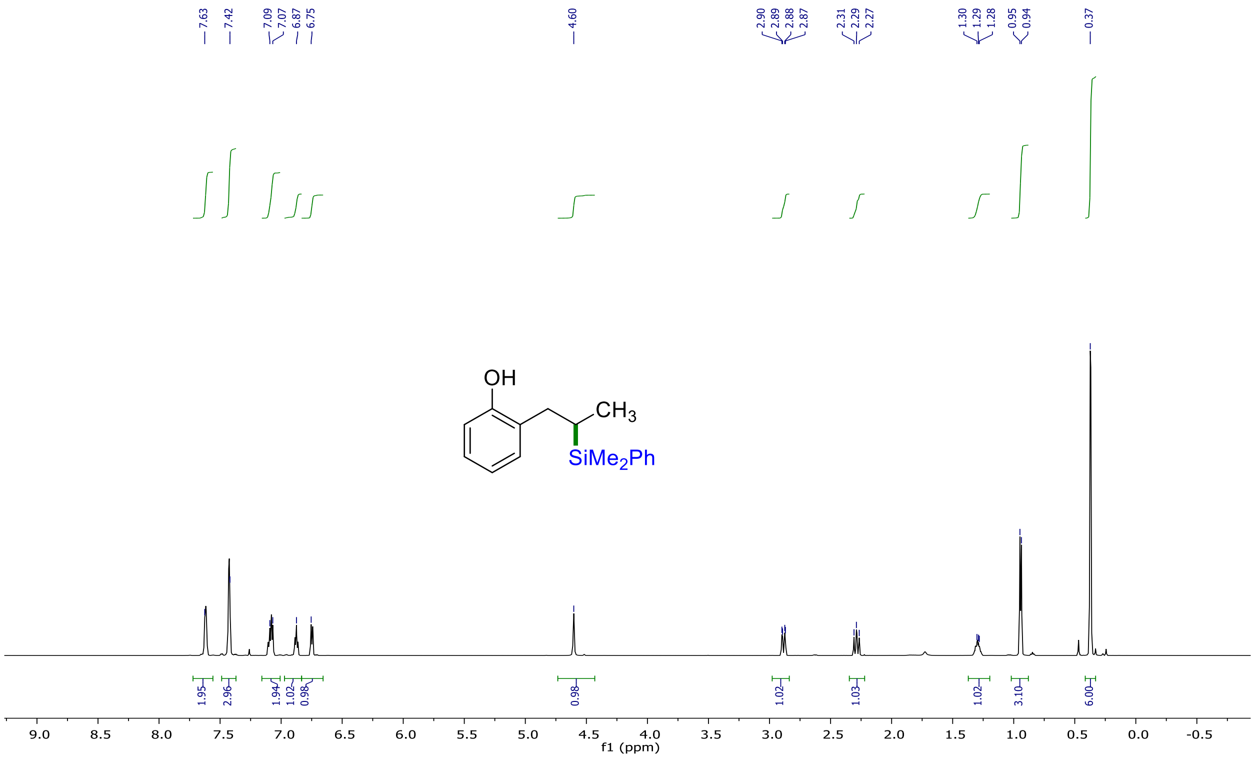

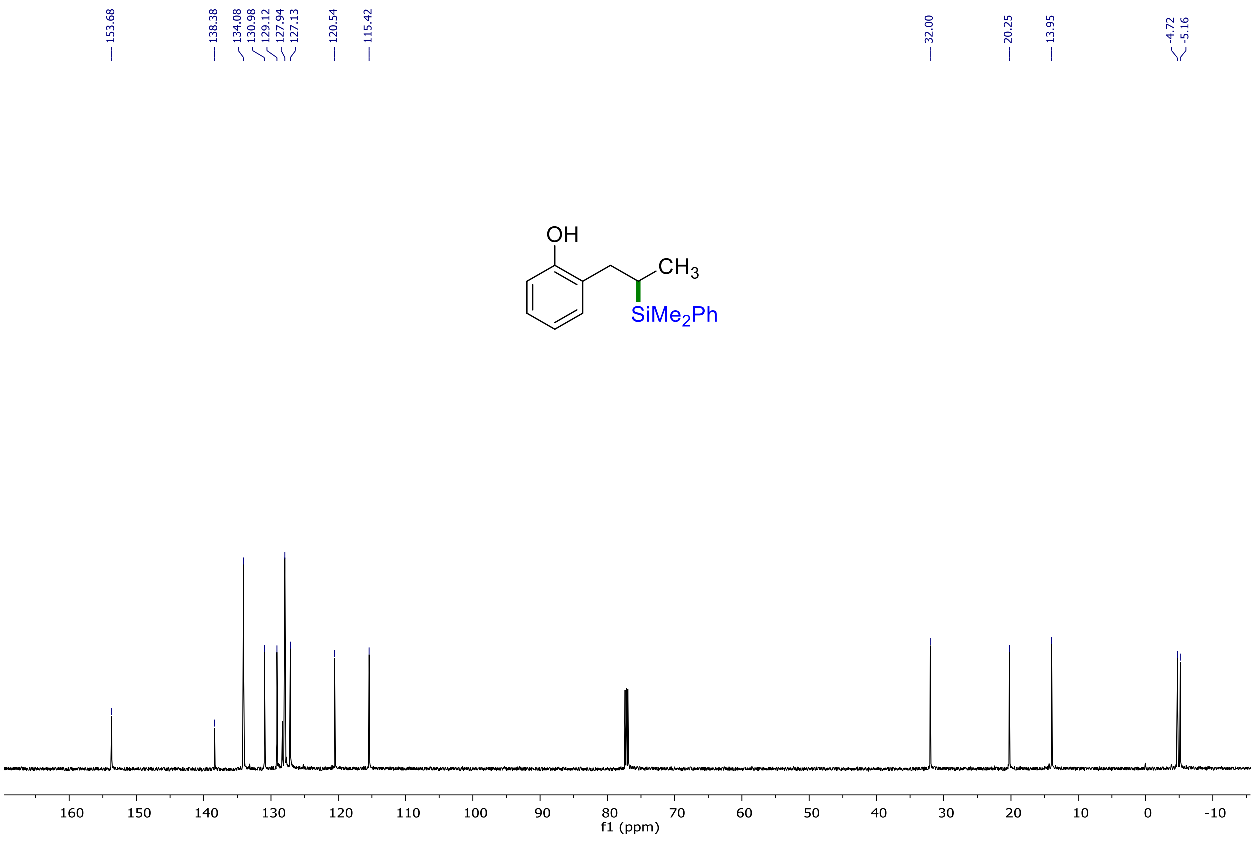

Supplementary Figure 153. ^{29}Si-NMR spectrum of **10**

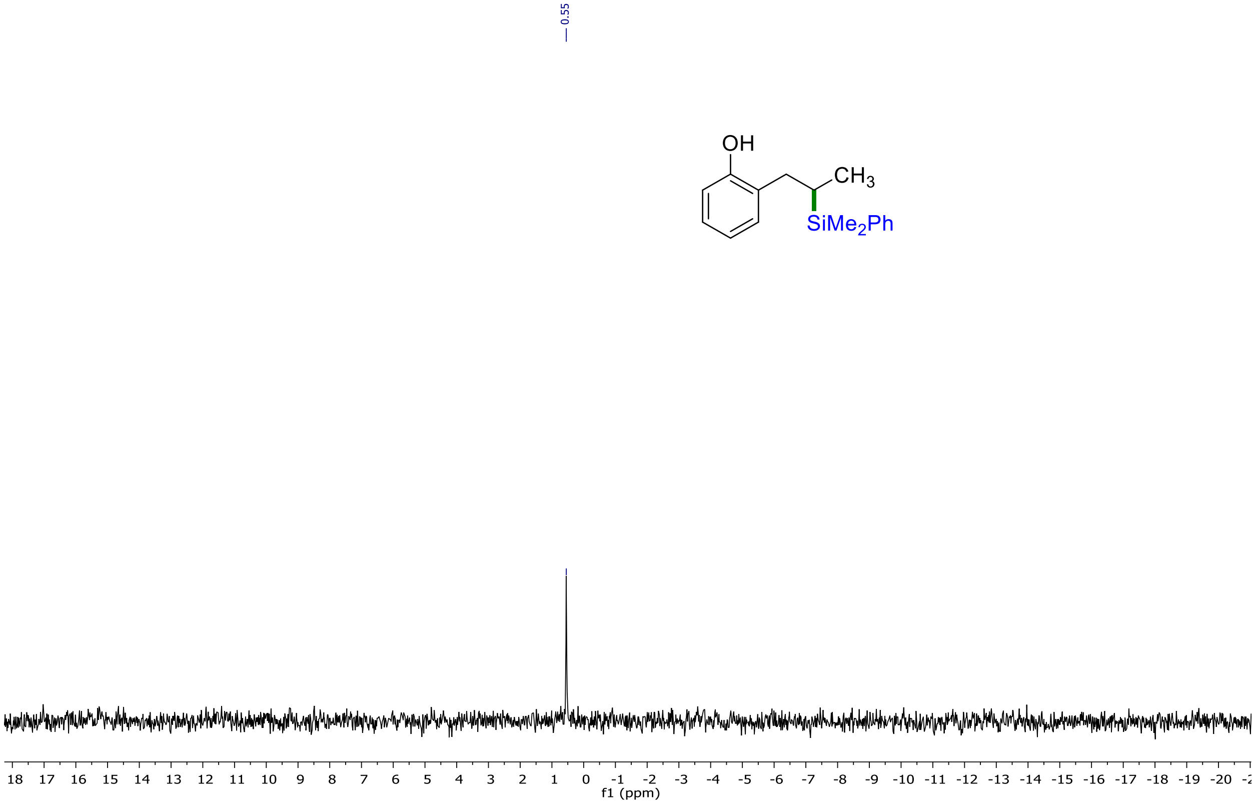

Supplementary Figure 154. ^{1}H-NMR spectrum of **10-*d*$_2$**

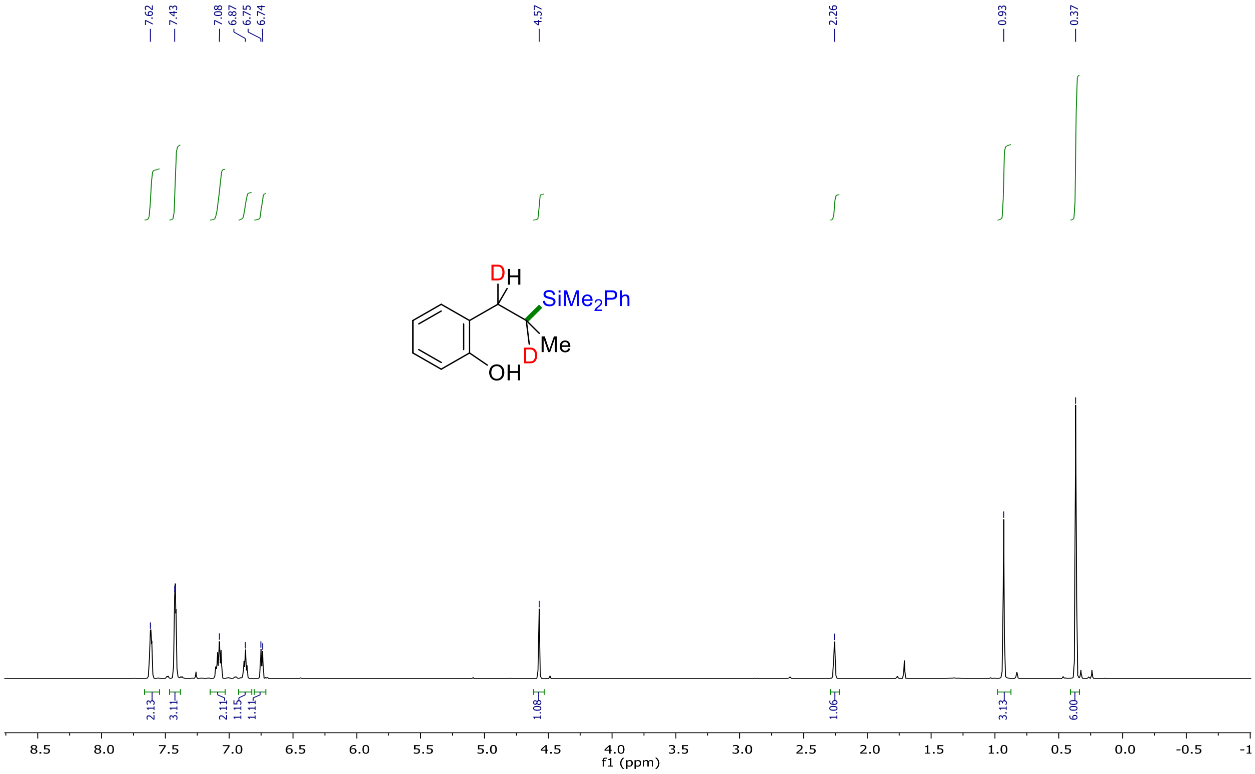

Supplementary Figure 155. ^{13}C and ^{29}Si-NMR spectra of **10-*d*$_2$**.

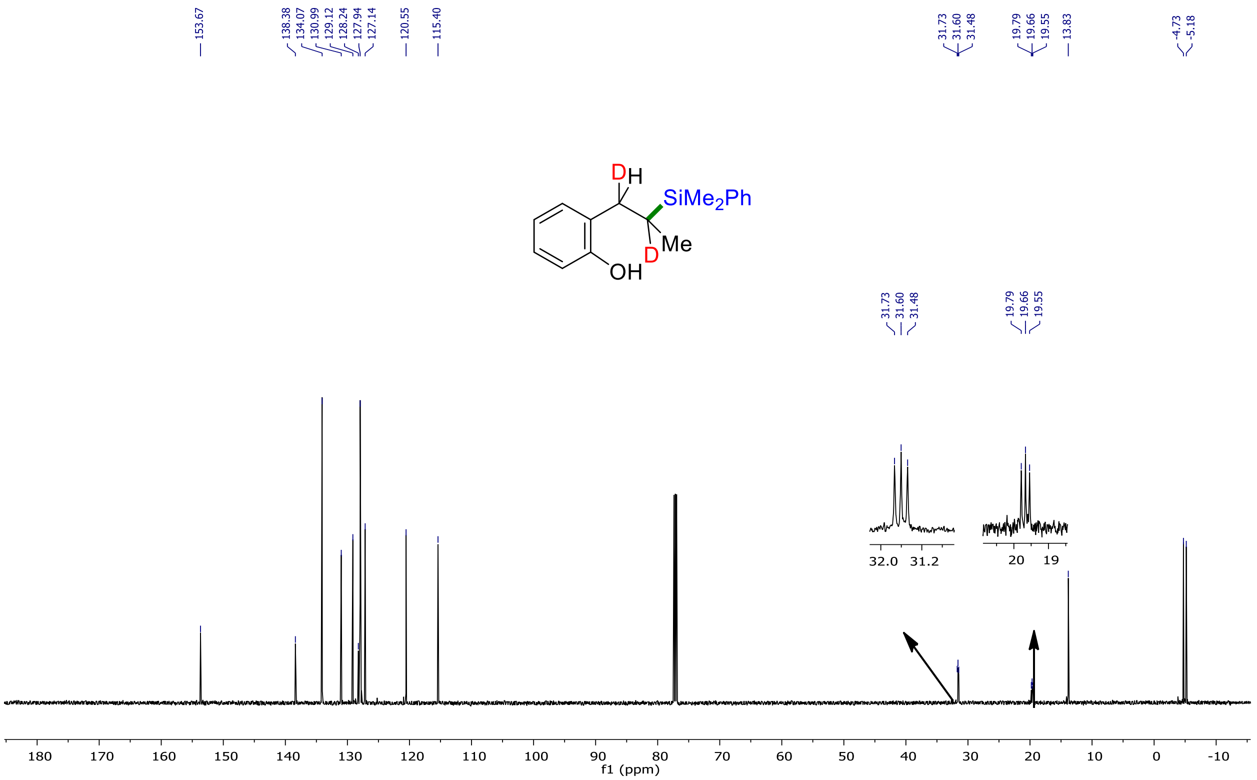

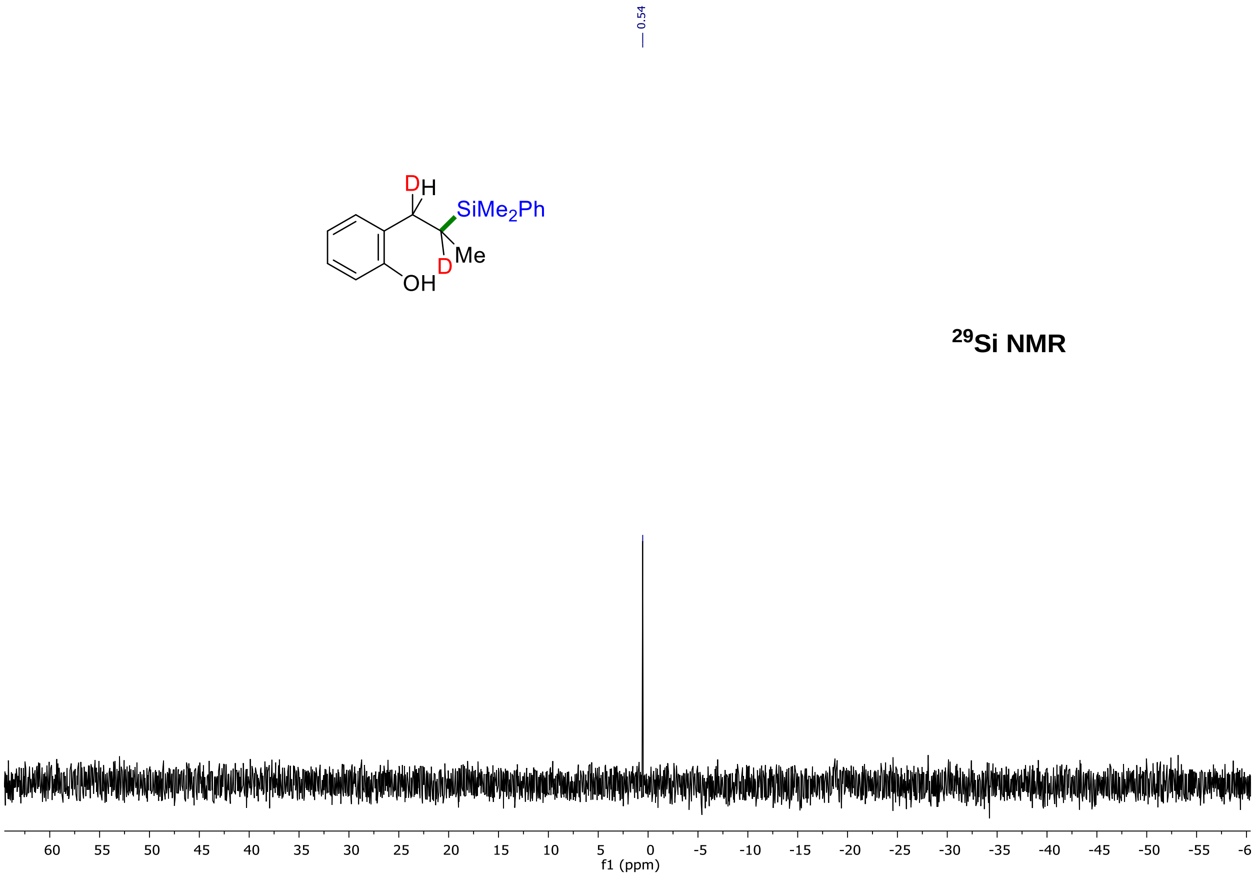

Supplementary Figure 156. ^{2}H-NMR spectrum of **10-*d*$_2$**

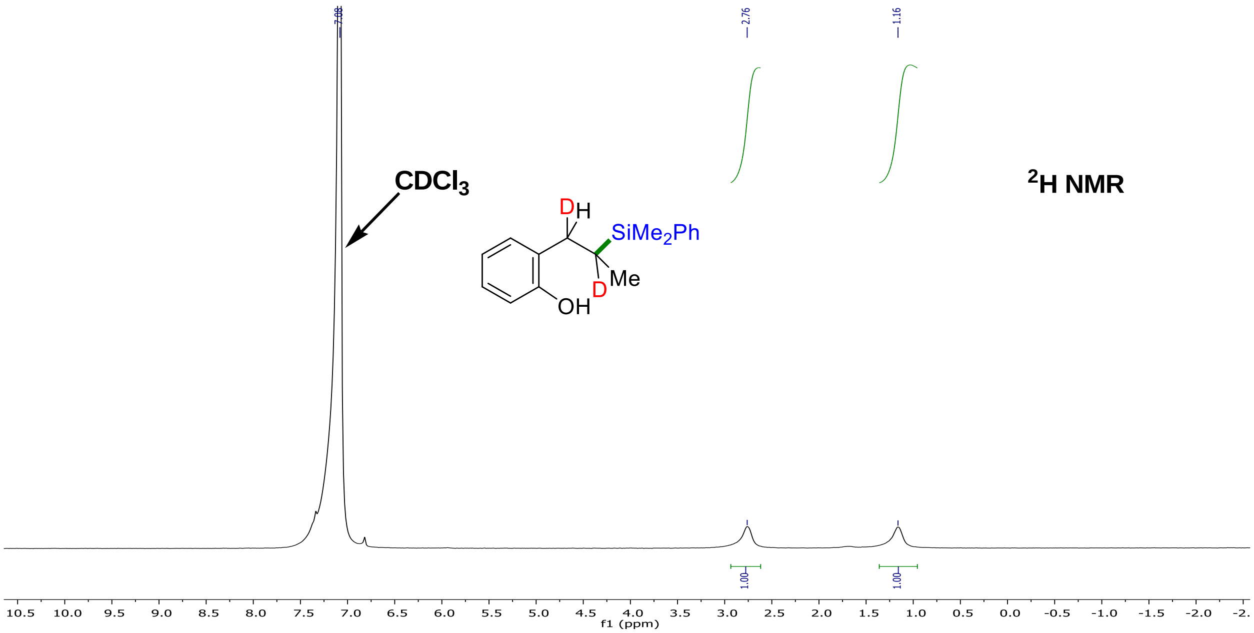

Supplementary Figure 157. ^{1}H-NMR spectrum of **11**

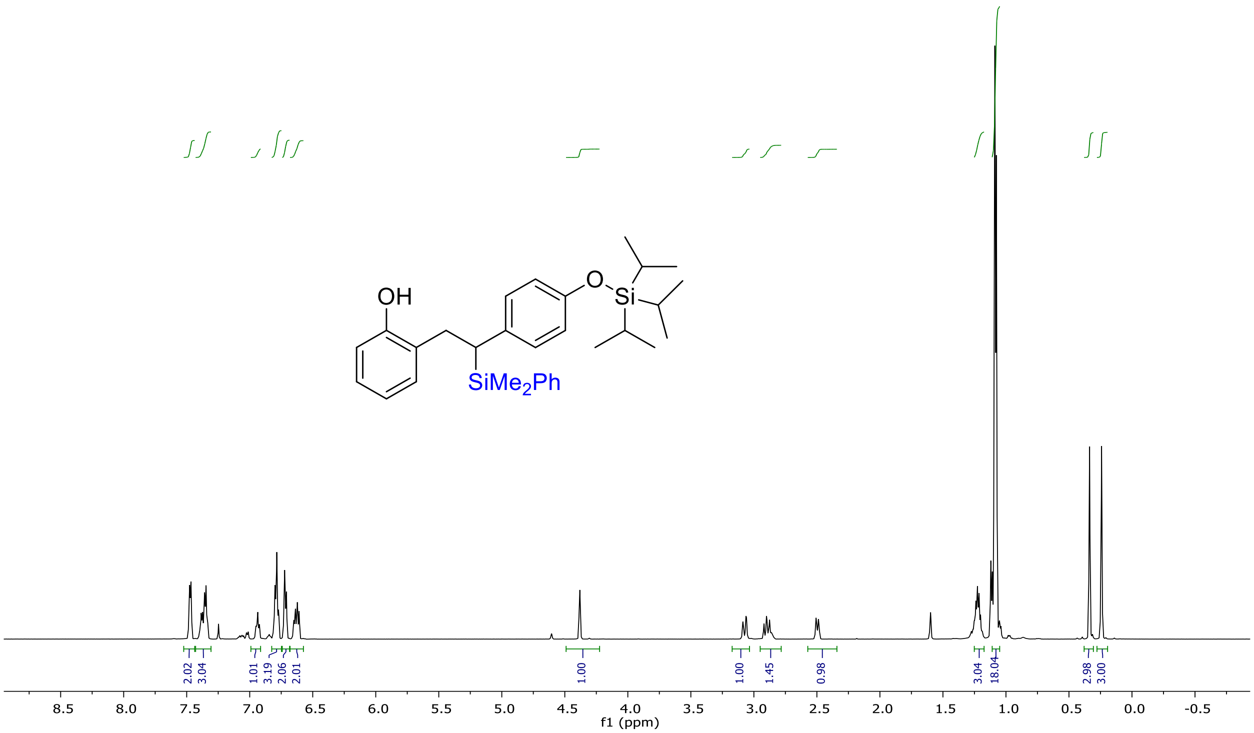

Supplementary Figure 158. ^{13}C and ^{29}Si-NMR spectra of **11**

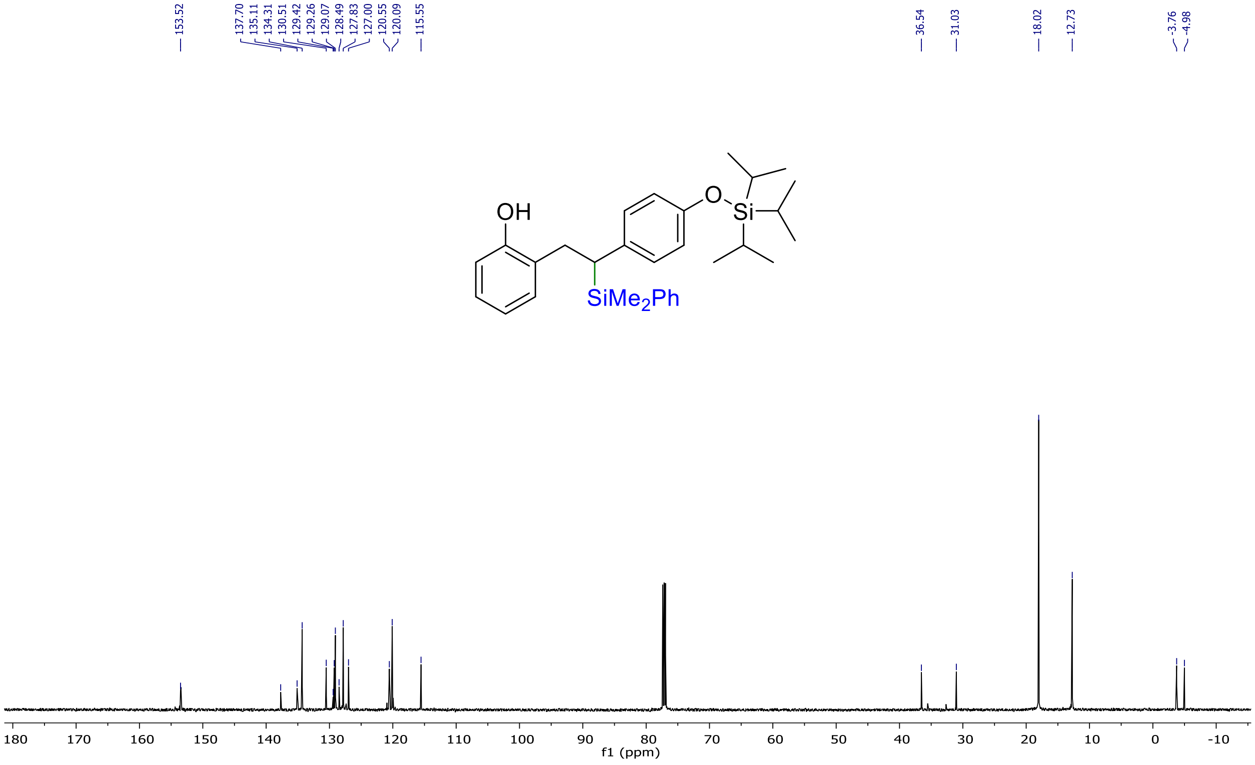

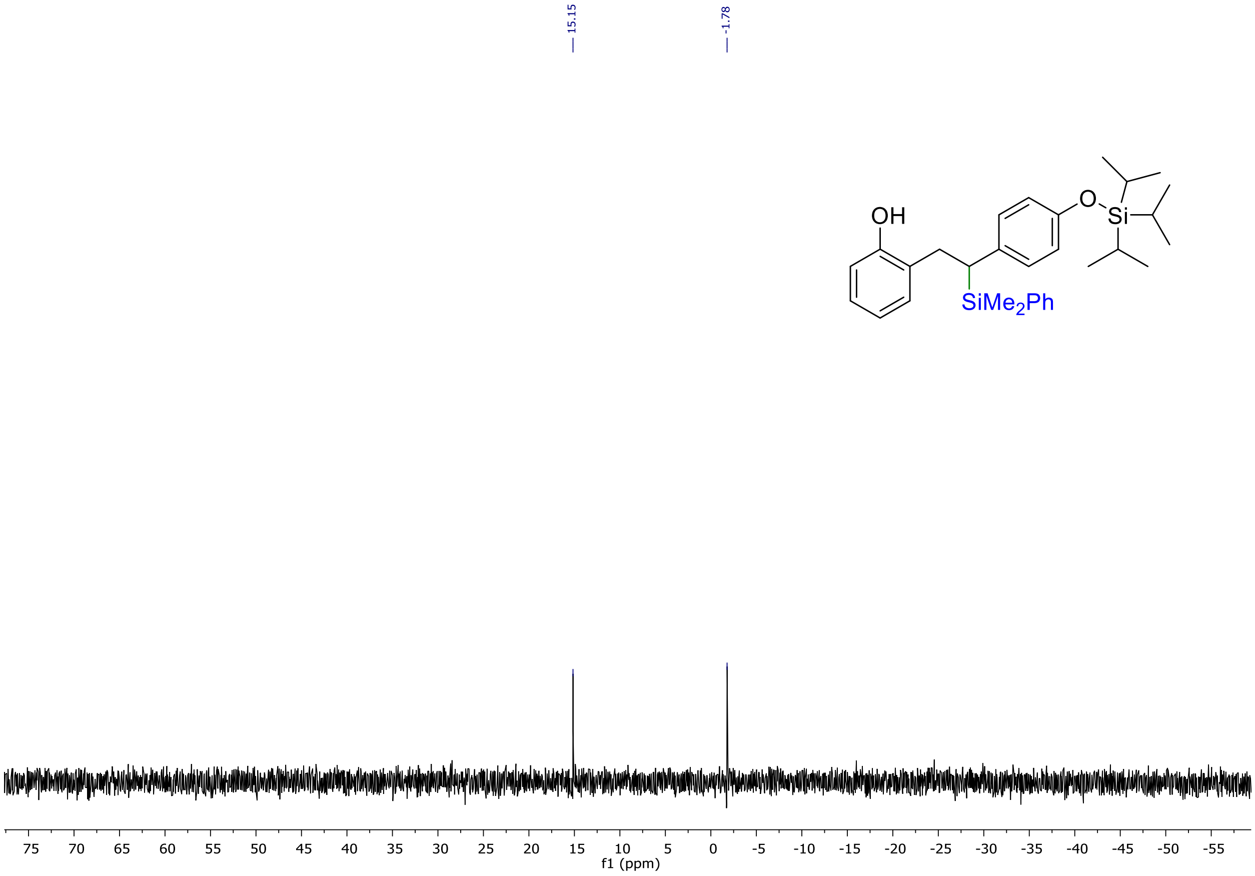

Supplementary Figure 159. ^{1}H and ^{13}C-NMR spectra of **12**

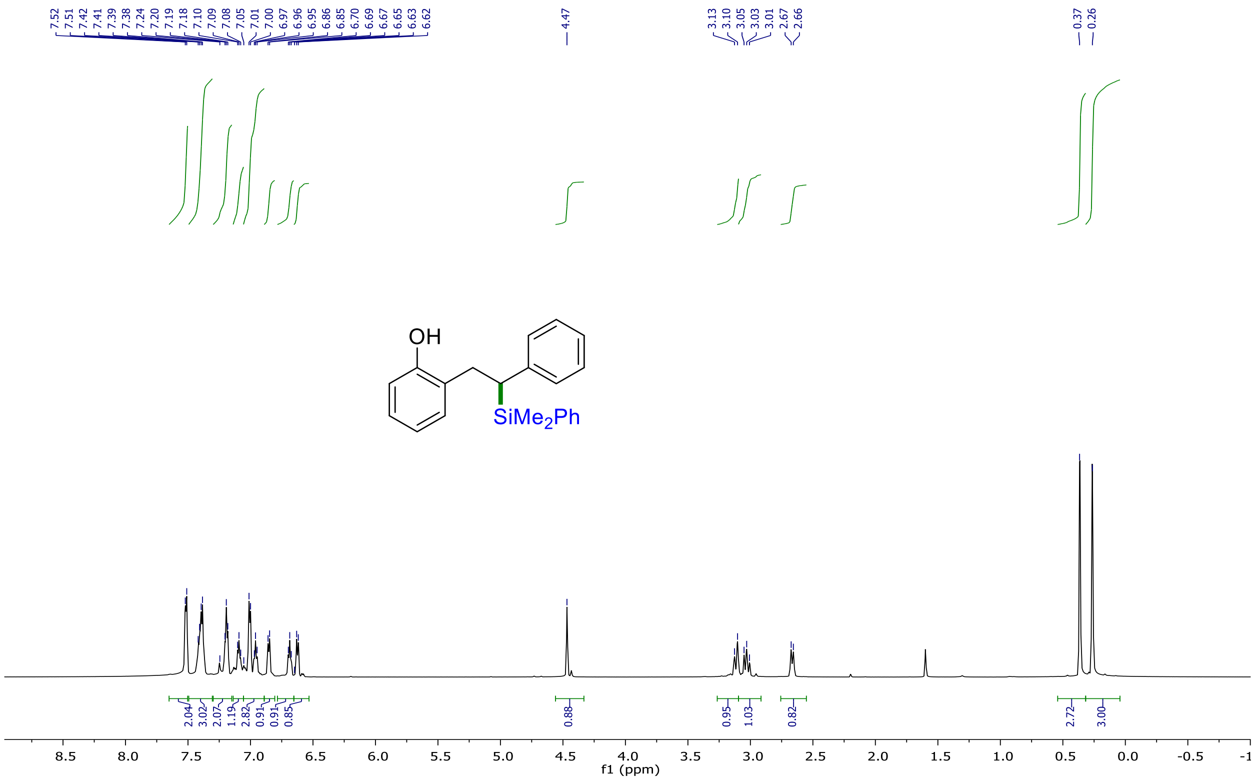

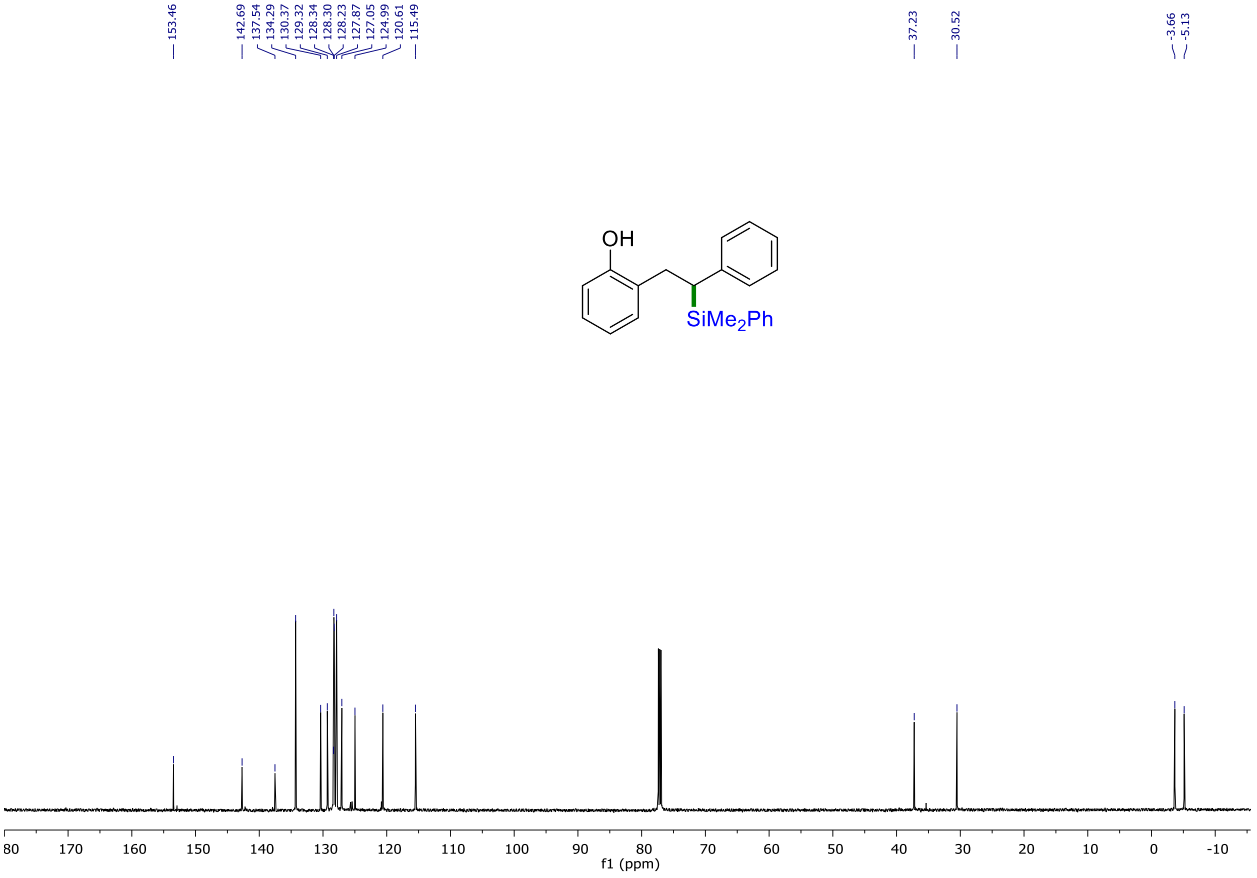

Supplementary Figure 160. ^{29}Si-NMR spectrum of **12**

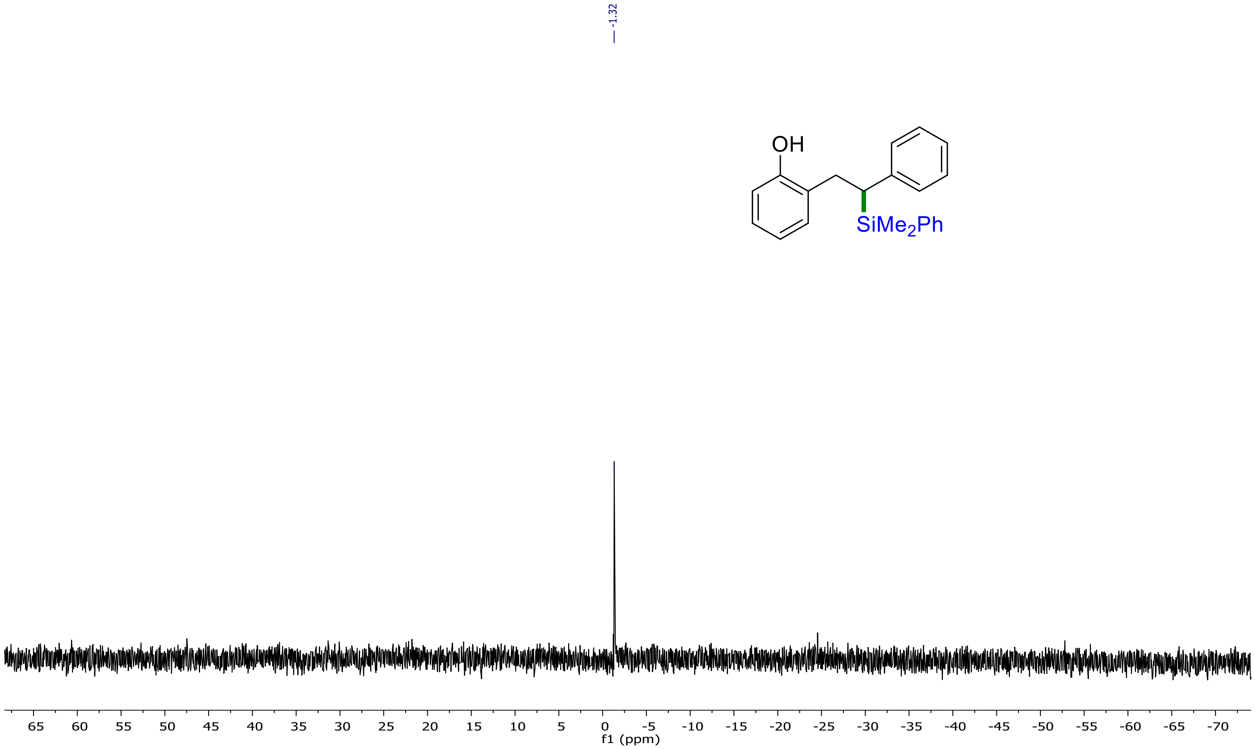

Supplementary Figure 161. ^{1}H-NMR spectrum of *Z*-**14-*d***

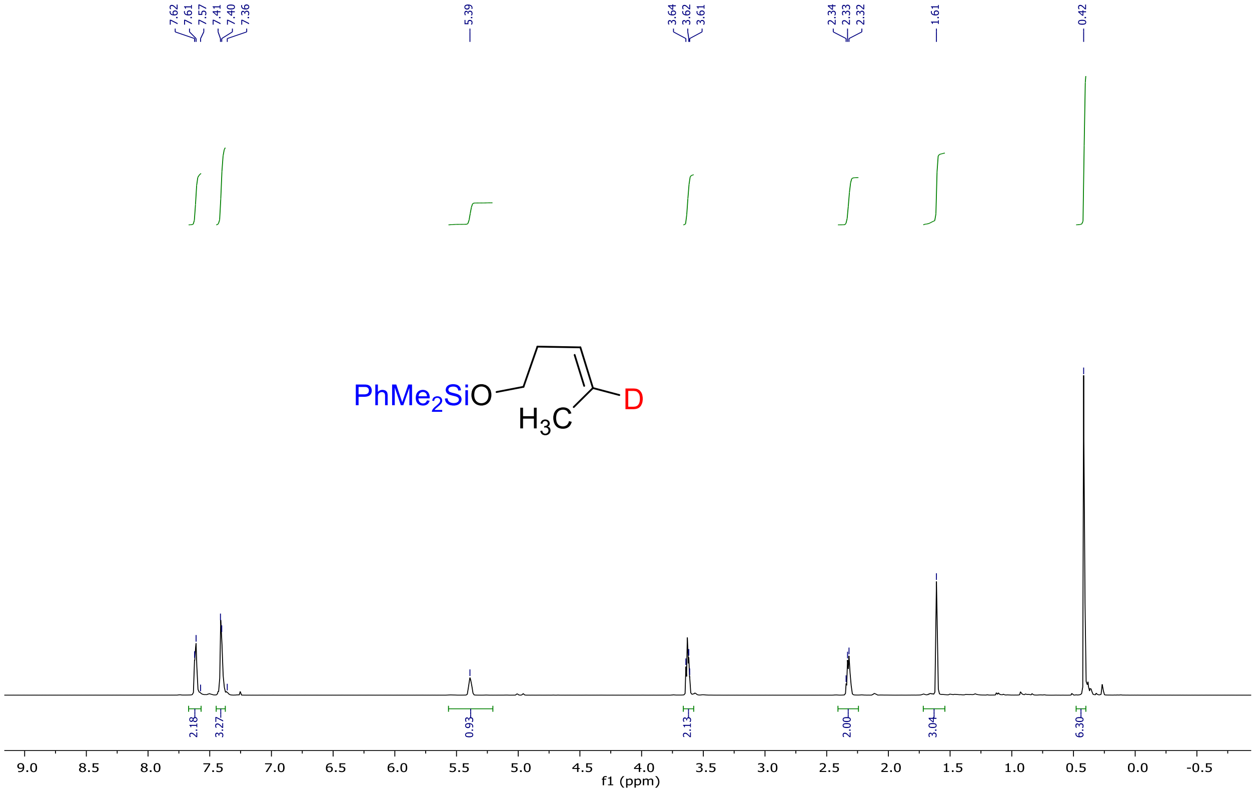

Supplementary Figure 162. ^{13}C and ^{29}Si-NMR spectra of *Z*-**14-*d***

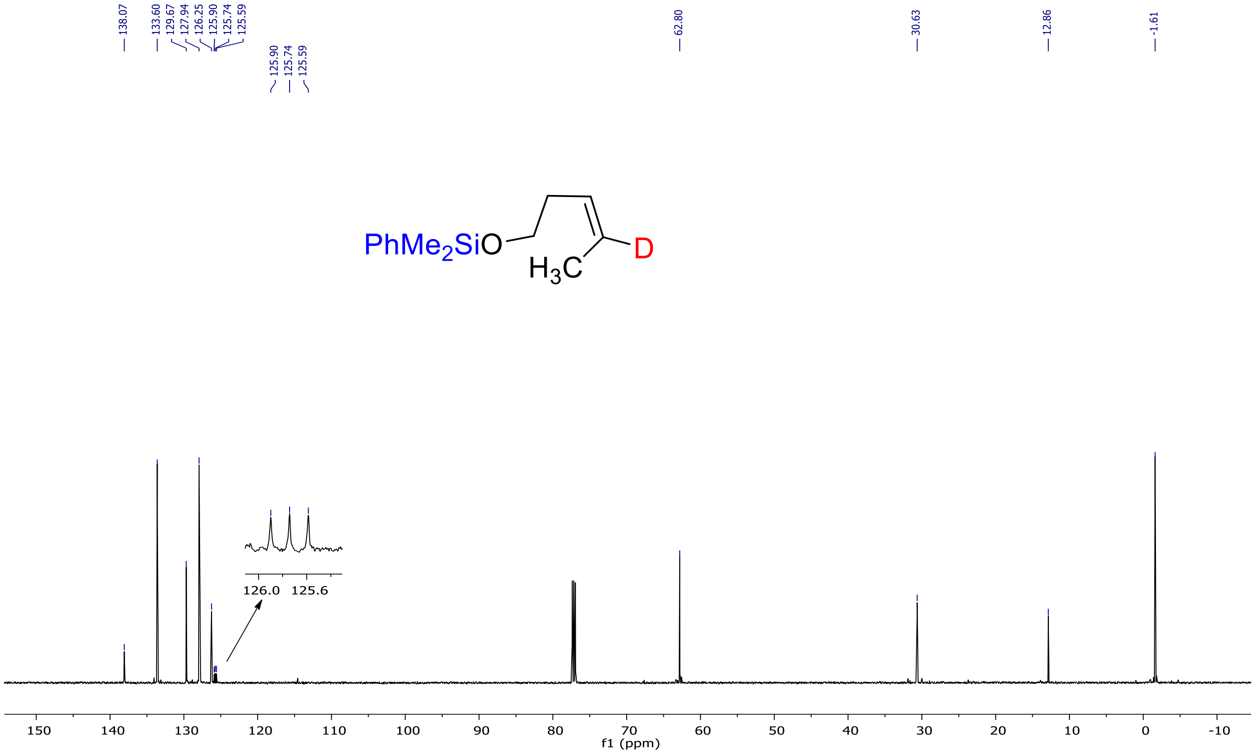

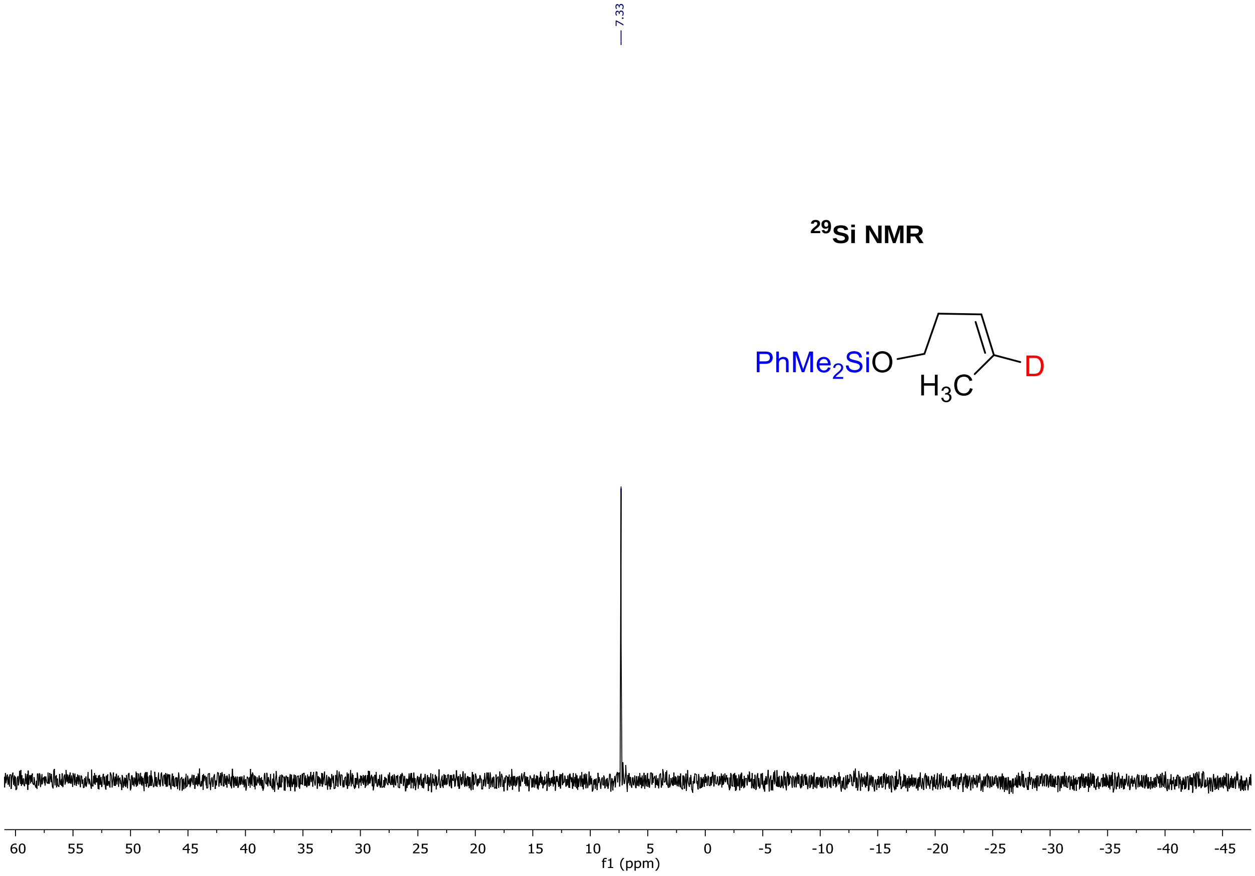

Supplementary Figure 163. ^{2}H-NMR spectrum of *Z*-**14-*d***

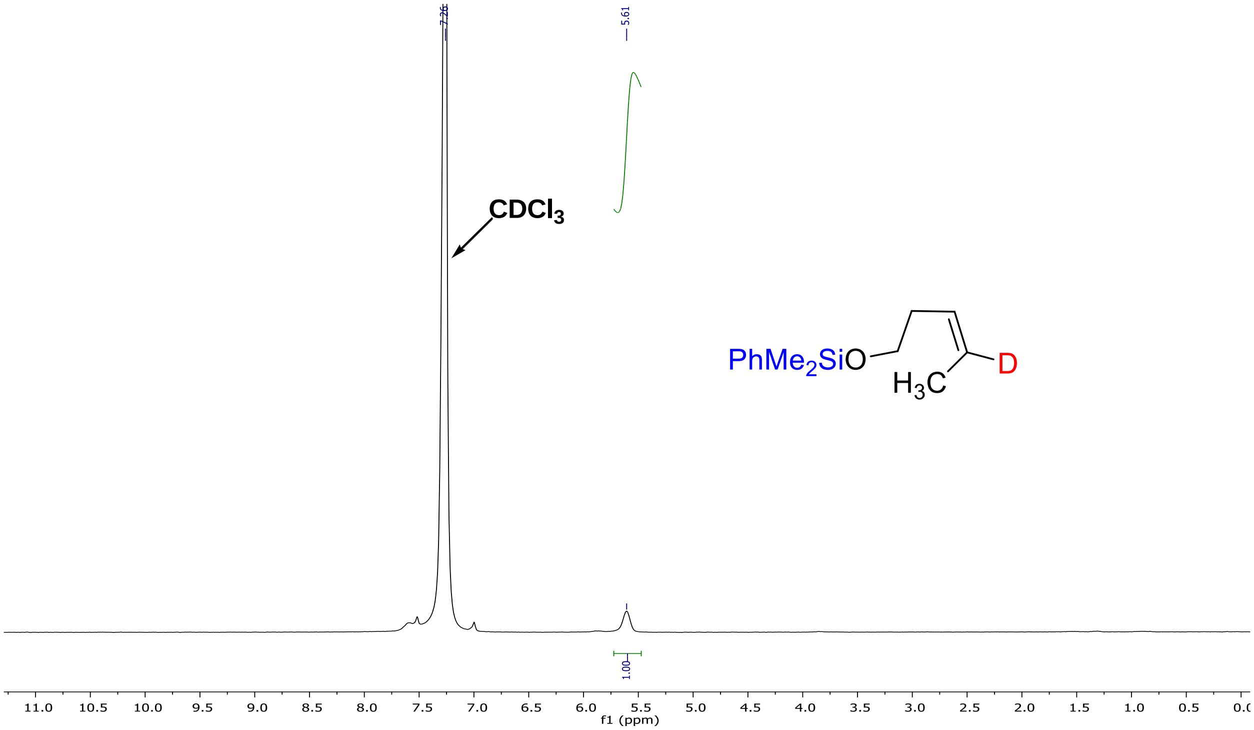

Supplementary Figure 164. ^{1}H-NMR spectrum of *Z*-**15**

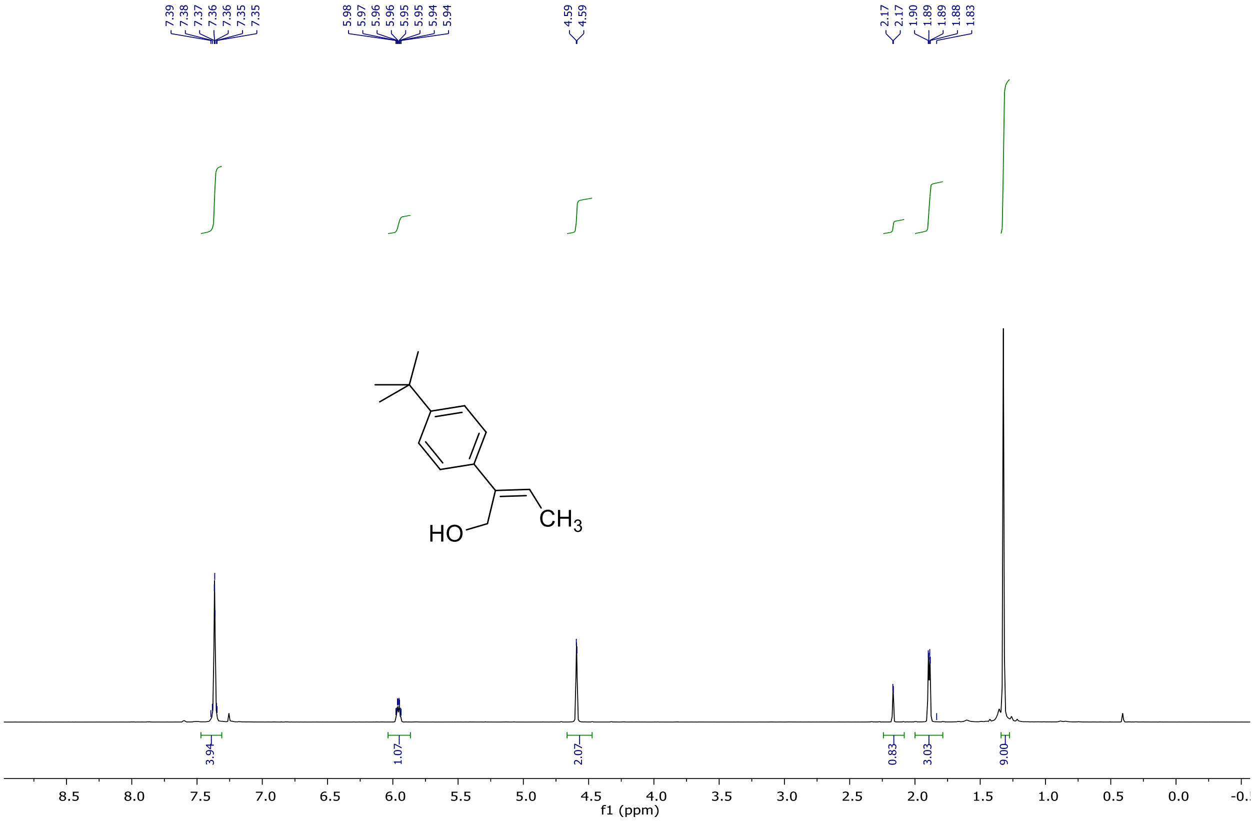

Supplementary Figure 165. ^{13}C-NMR spectrum of *Z*-**15**

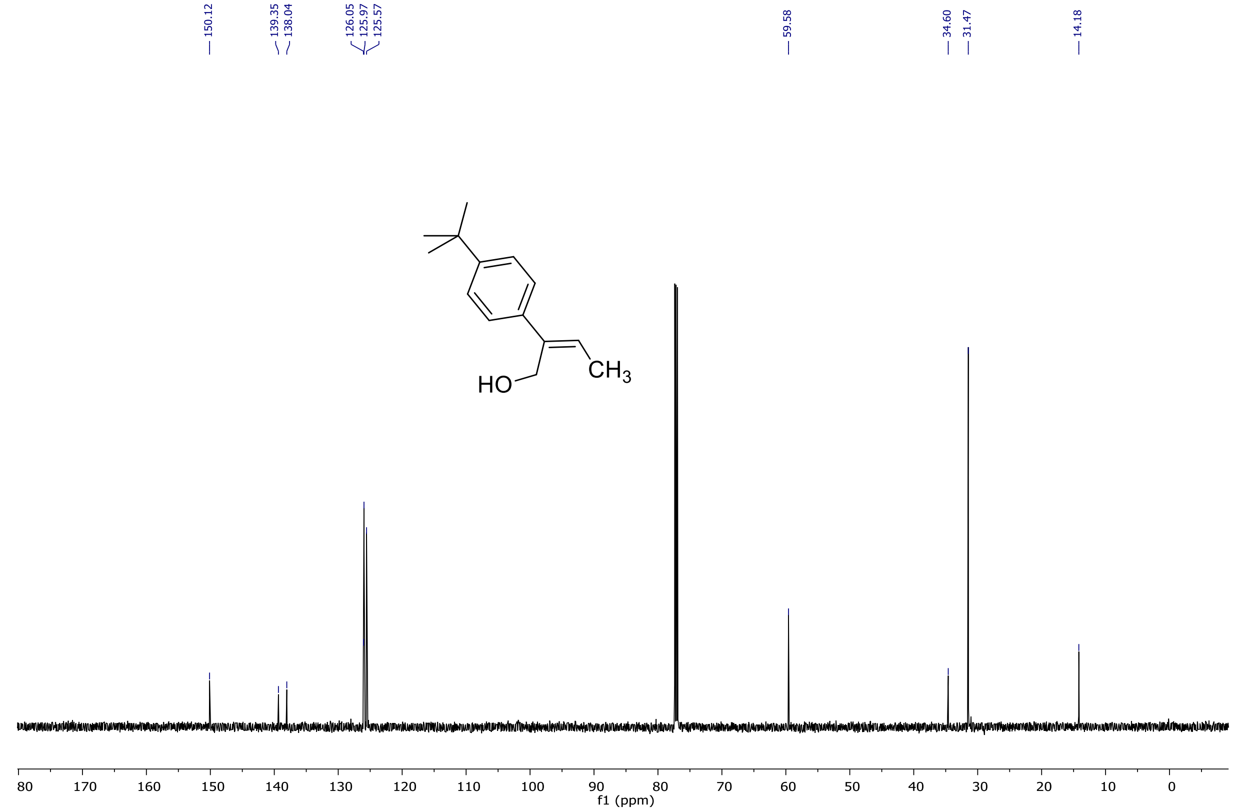

Supplementary Figure 166. ^{1}H-NMR spectrum of *Z*-**16**

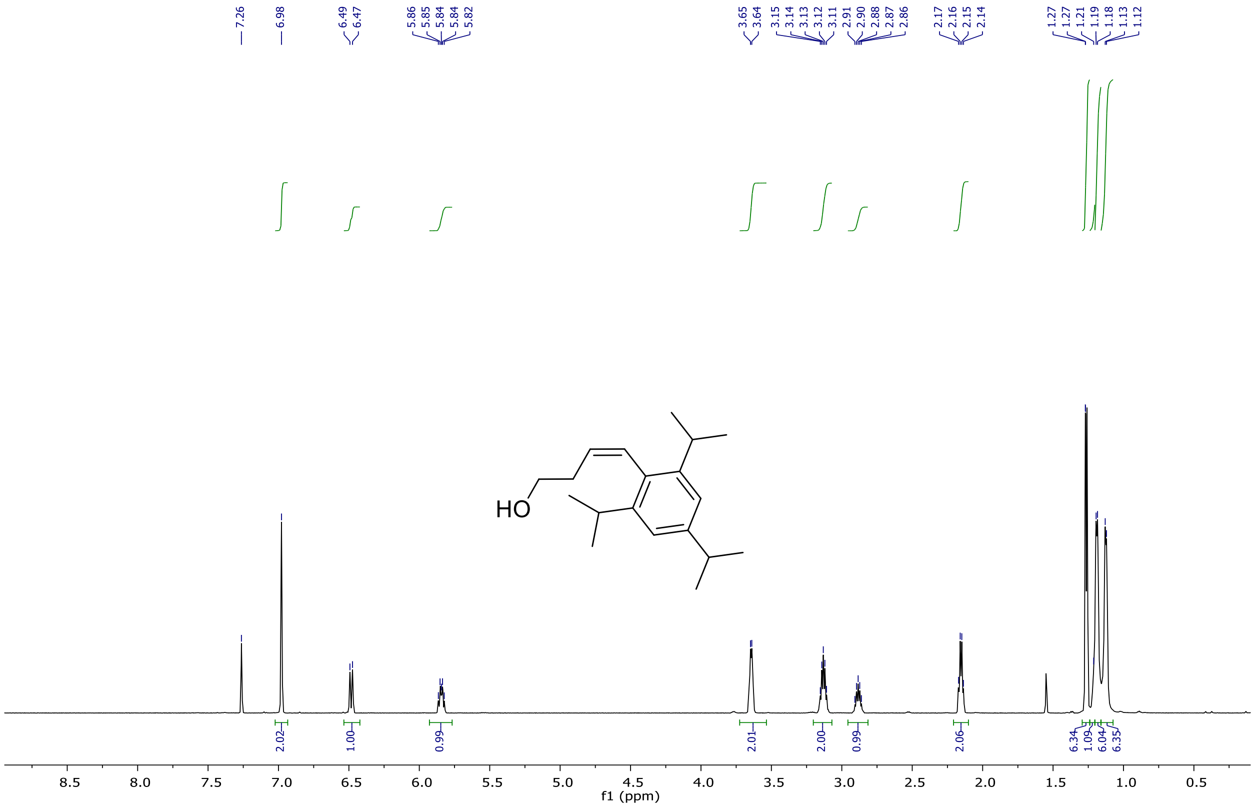

Supplementary Figure 167. ^{13}C-NMR spectrum of *Z*-**16**

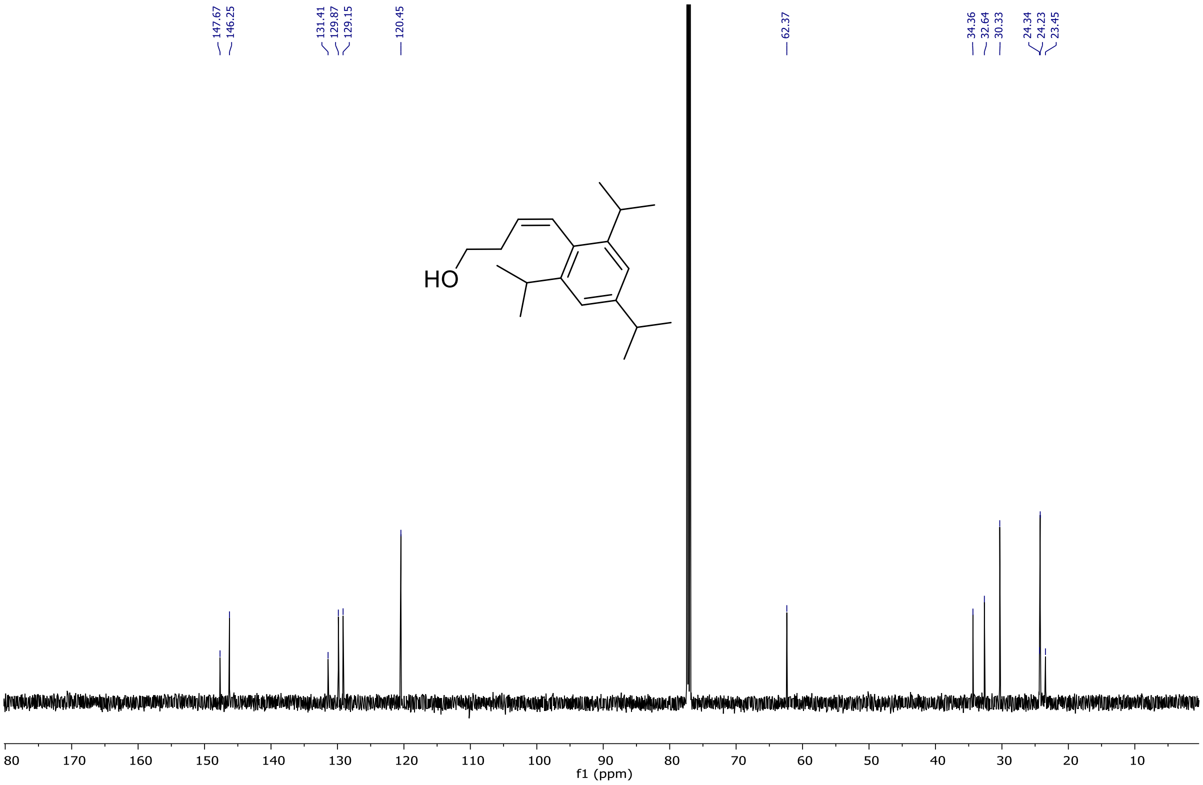

Supplementary Figure 168. ^{1}H-NMR spectrum of *Z*-**17**

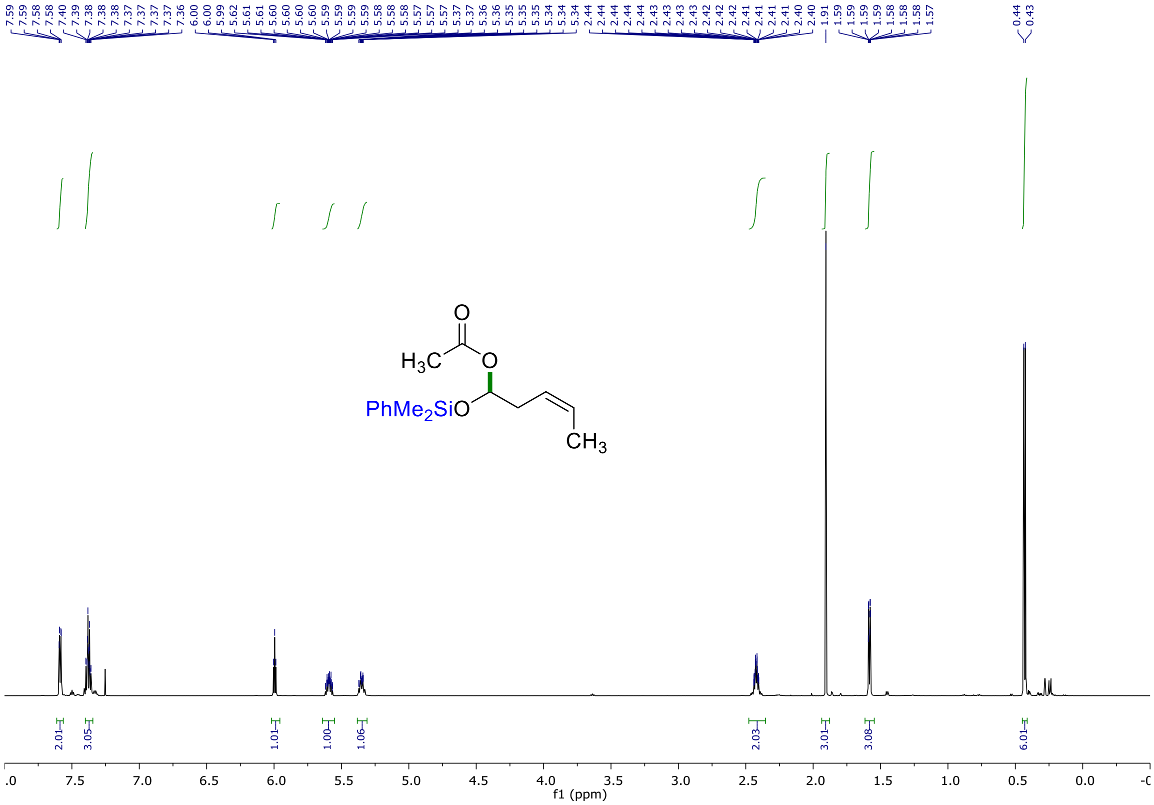

Supplementary Figure 169. ^{13}C and ^{29}Si-NMR spectra of *Z*-**17**

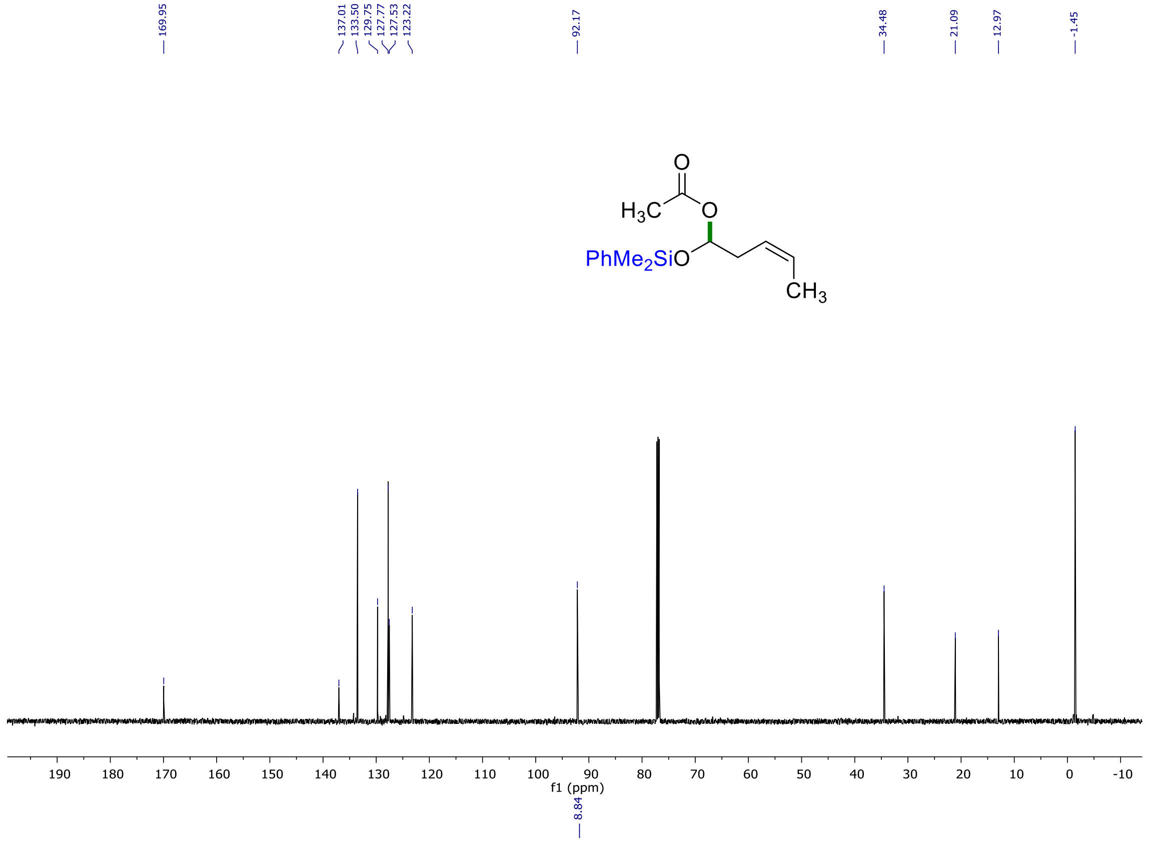

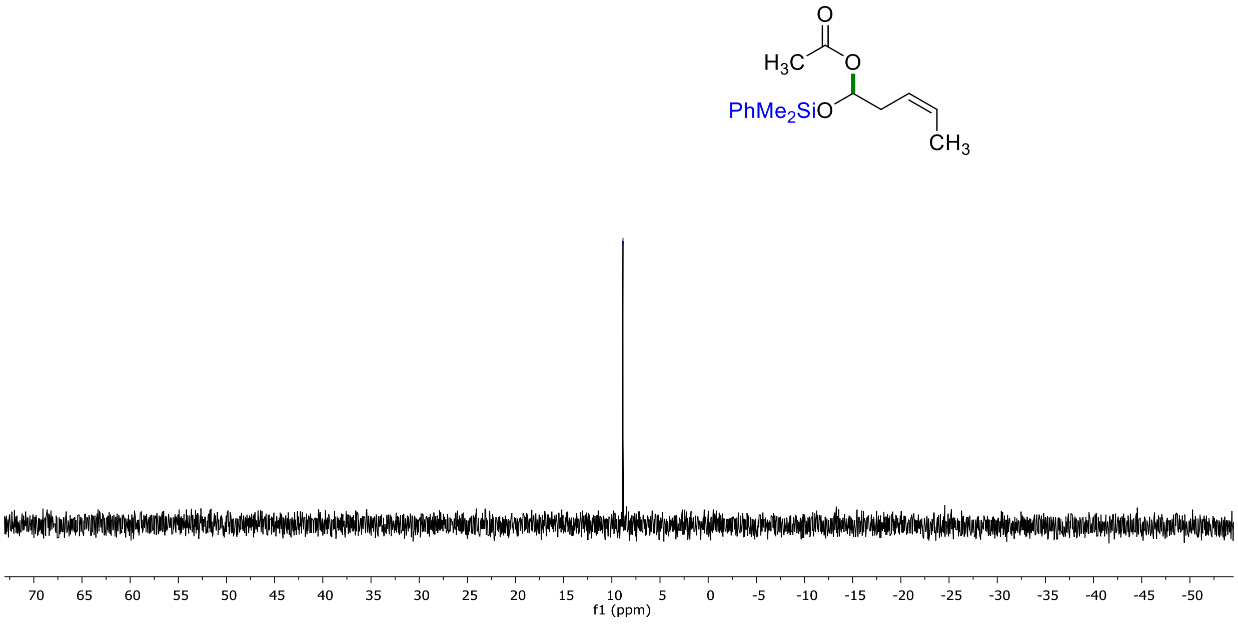

Supplementary Figure 170. ^{1}H and ^{13}C-NMR spectra of *Z*-**18**

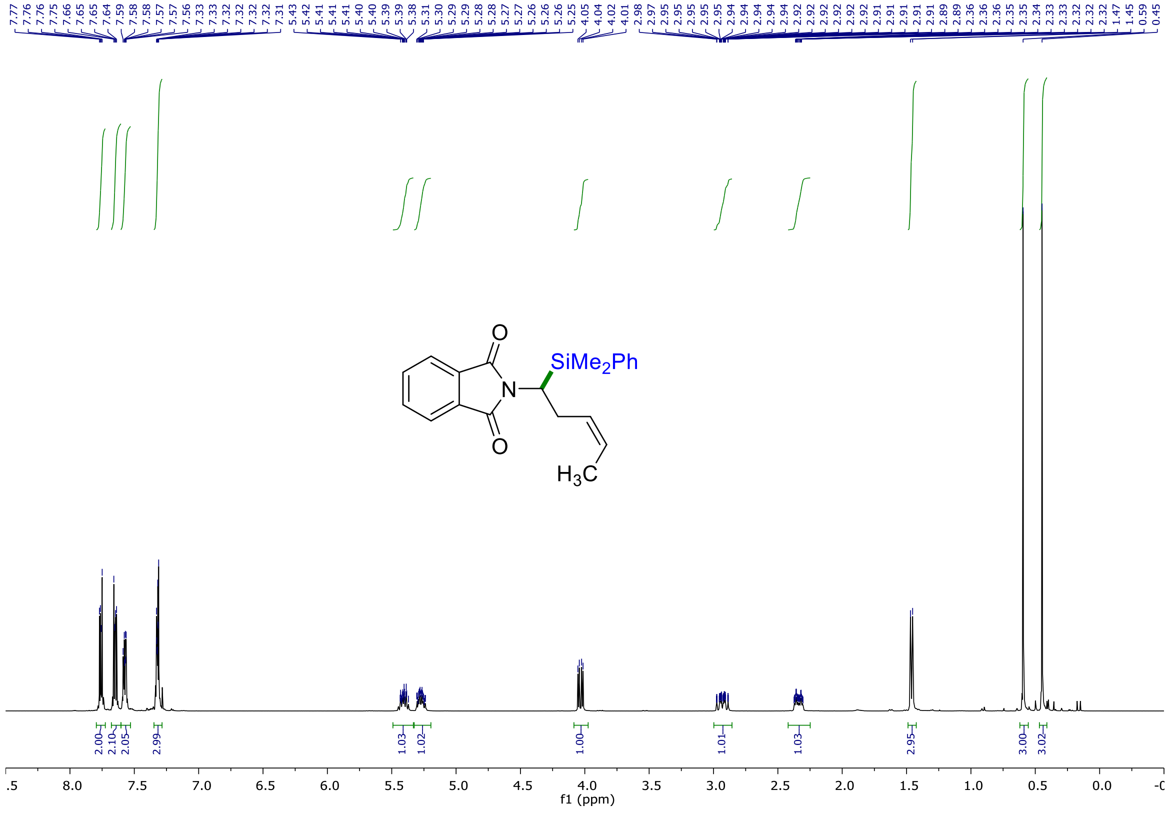

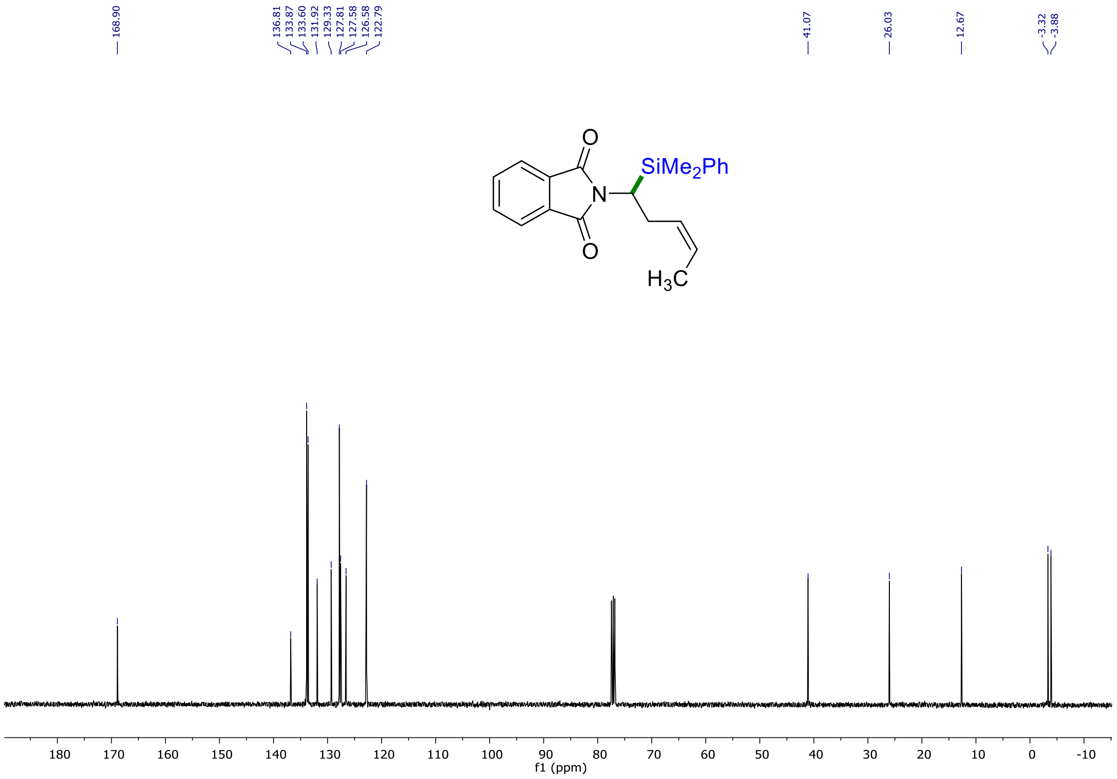

Supplementary Figure 171. ^{29}Si-NMR spectrum of *Z*-**18**

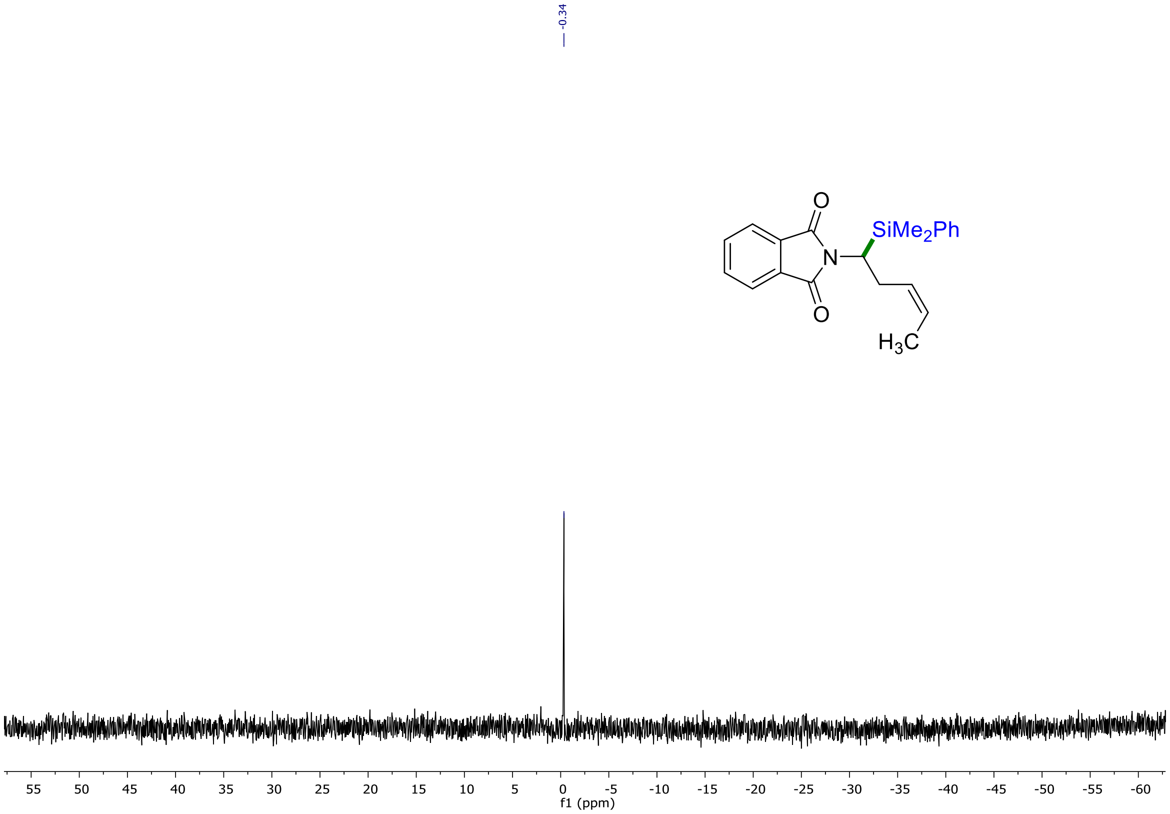

Supplementary Figure 172. ^{1}H-NMR spectrum of ***syn*-19**

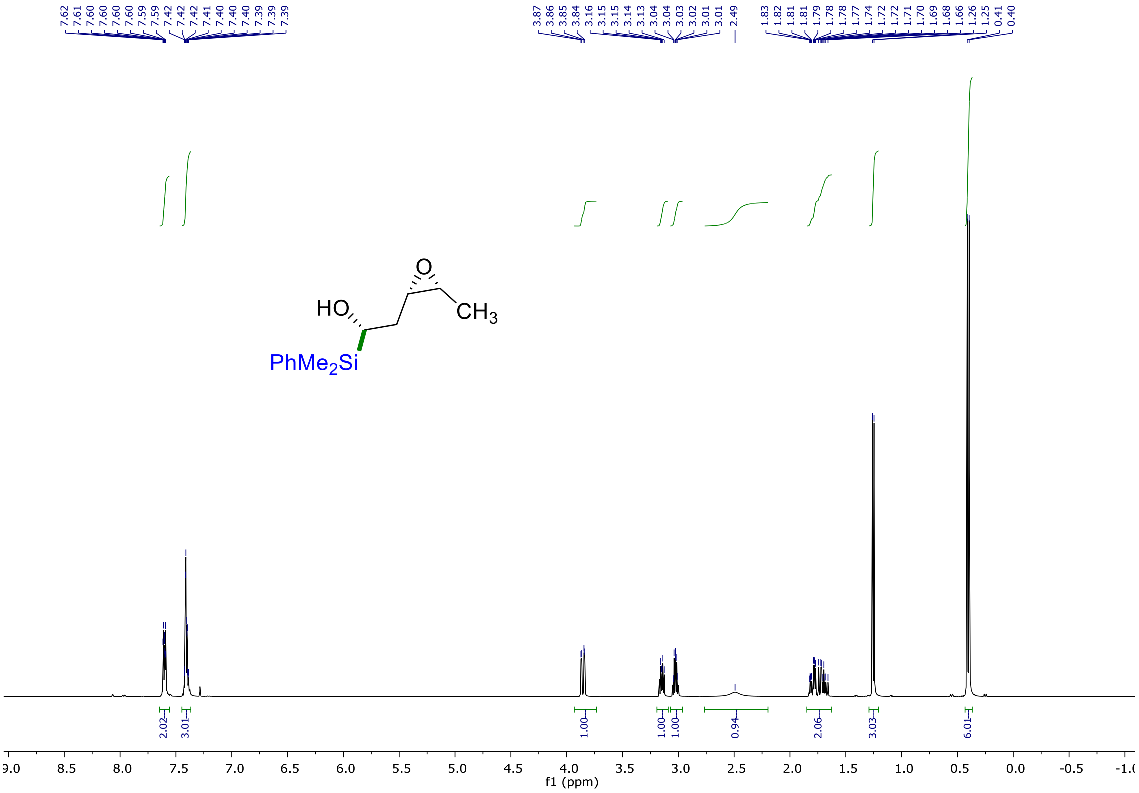

Supplementary Figure 173. ^{13}C and ^{29}Si-NMR spectra of ***syn*-19**

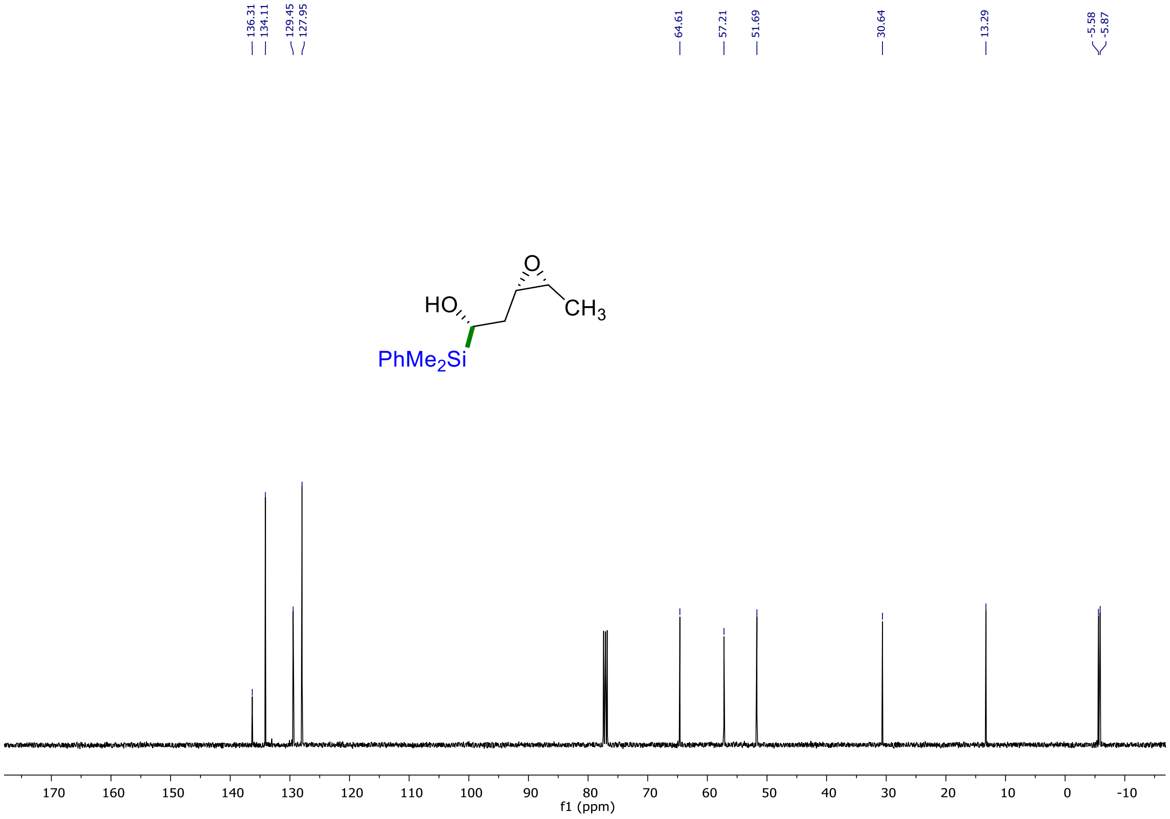

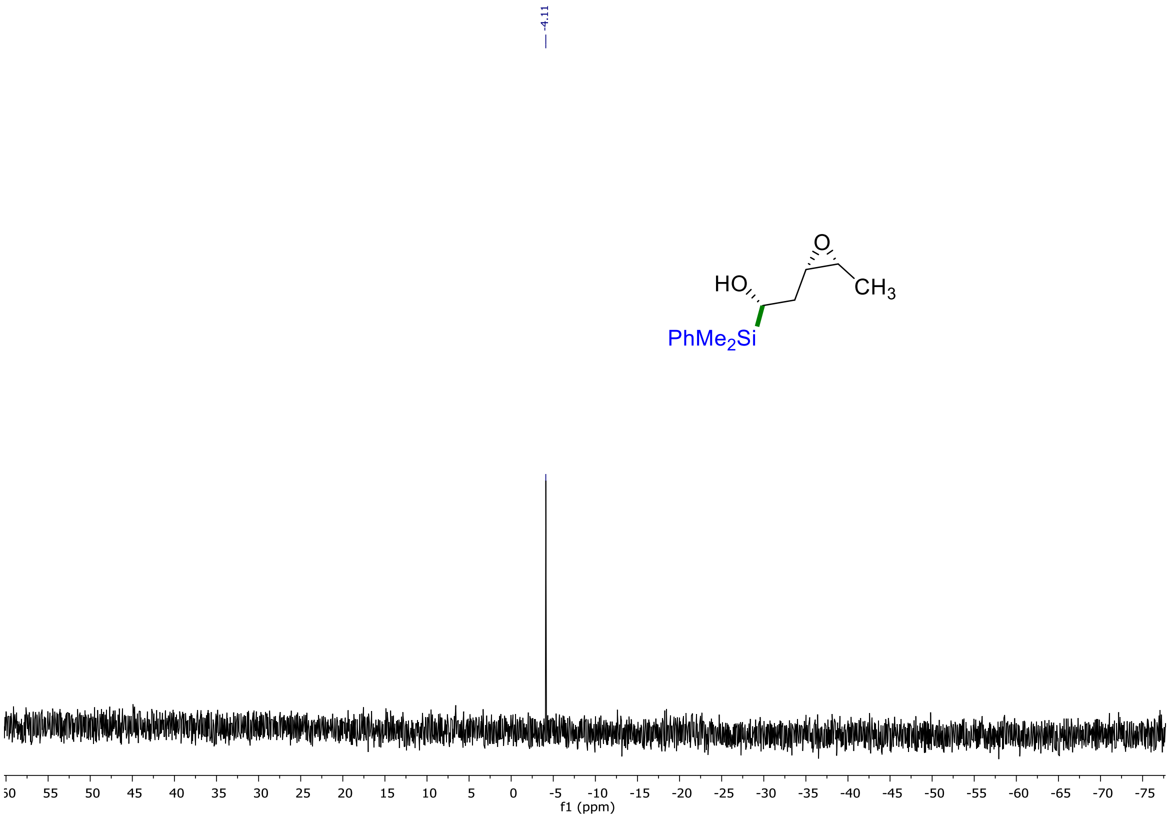

Supplementary Figure 174. ^{1}H and ^{13}C-NMR spectra of ***syn*-20**

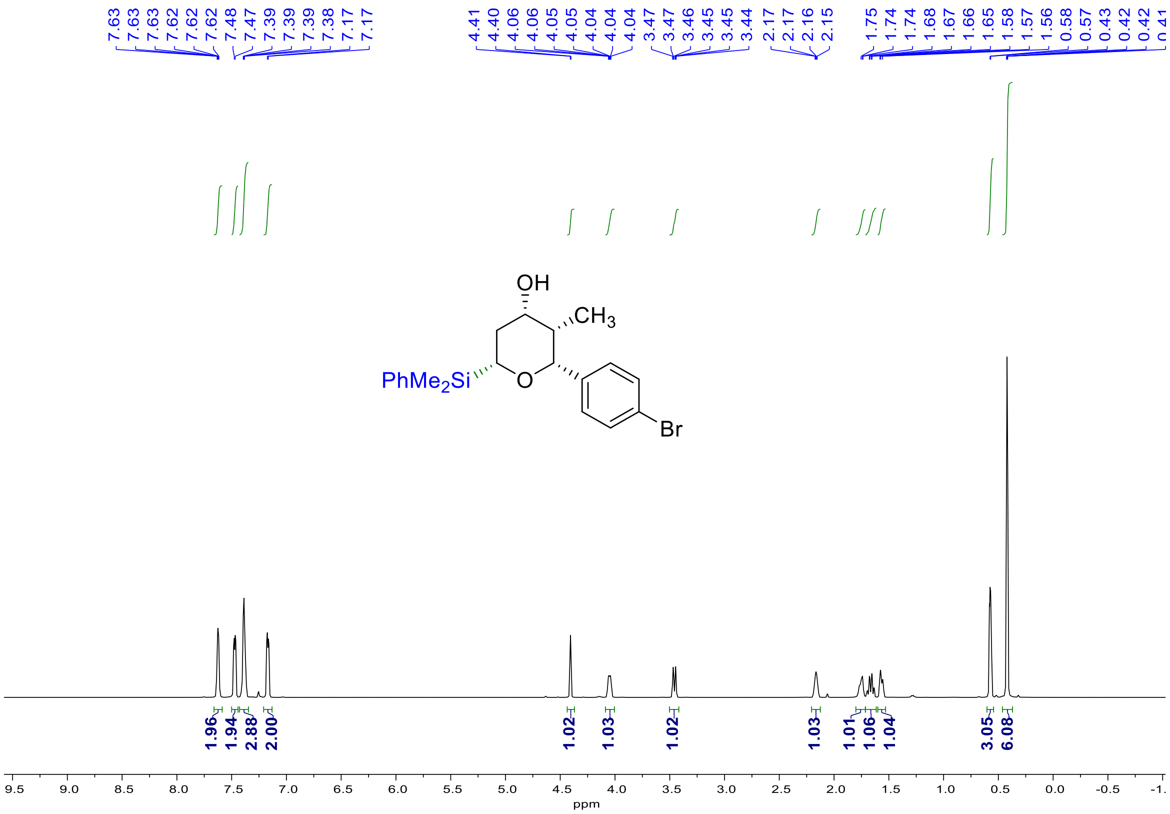

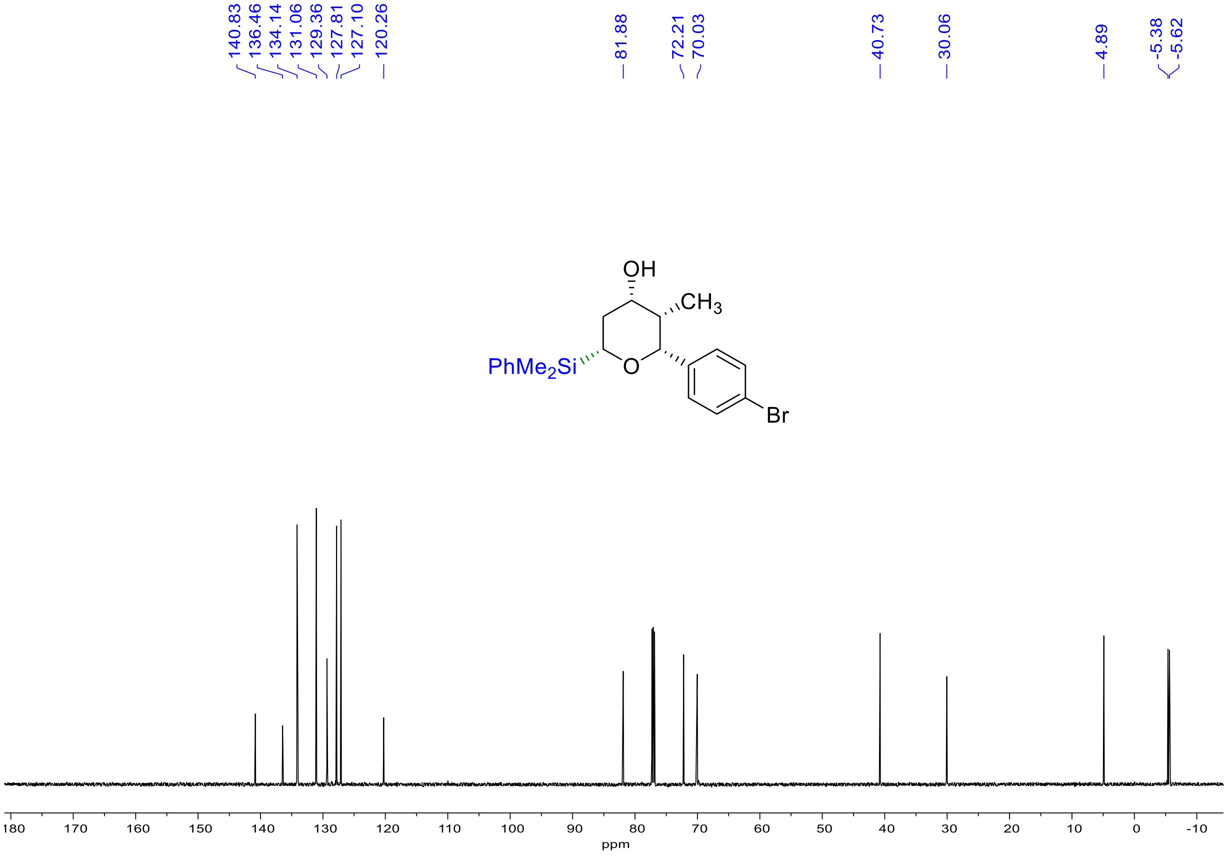

Supplementary Figure 175. ^{29}Si-NMR spectrum of ***syn*-20**

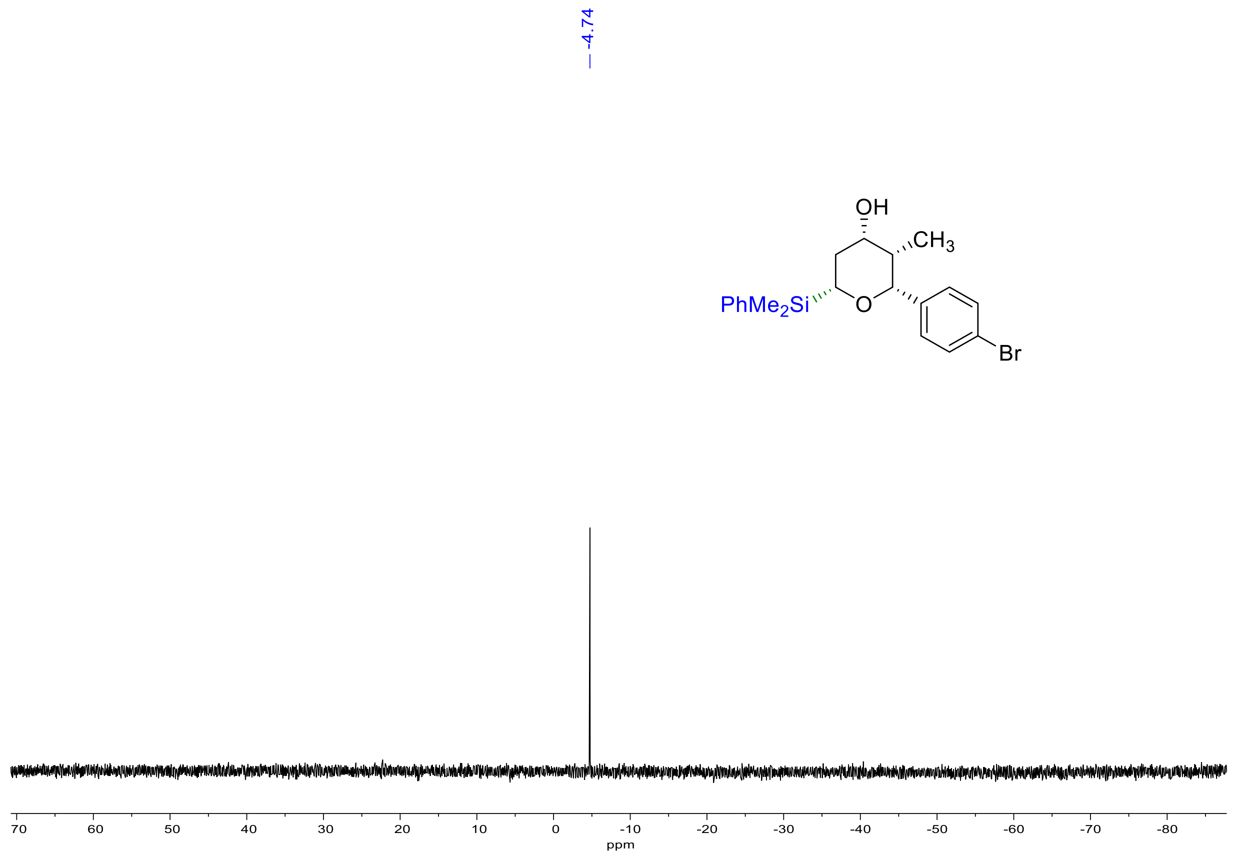

Supplementary Figure 176. ^{1}H-NMR spectrum of ***syn*-20'**

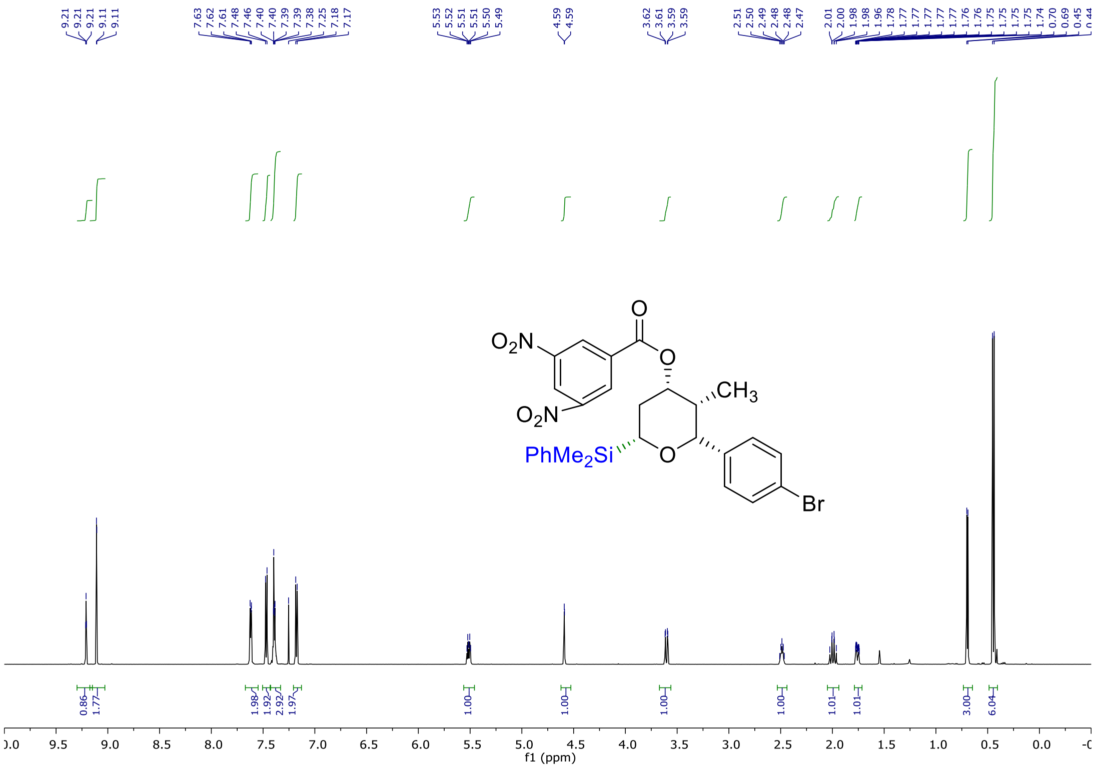

Supplementary Figure 177. ^{13}C and ^{29}Si-NMR spectrum of ***syn*-20'**

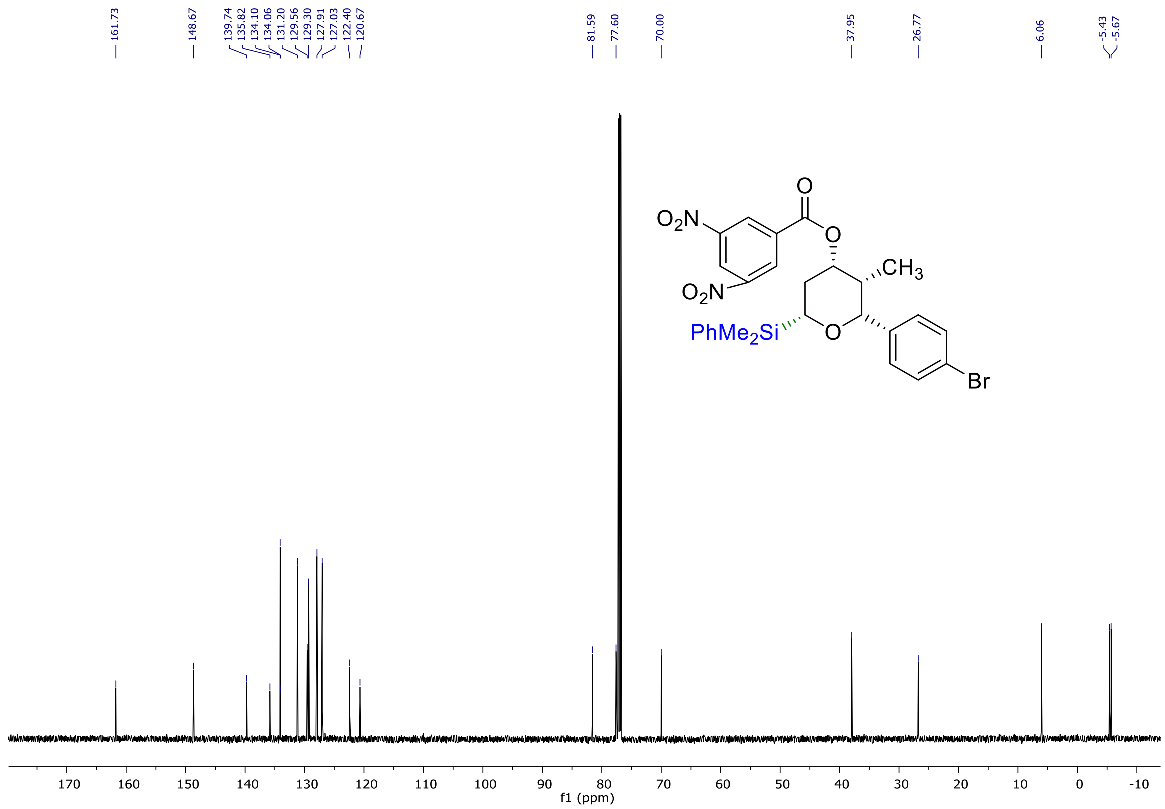

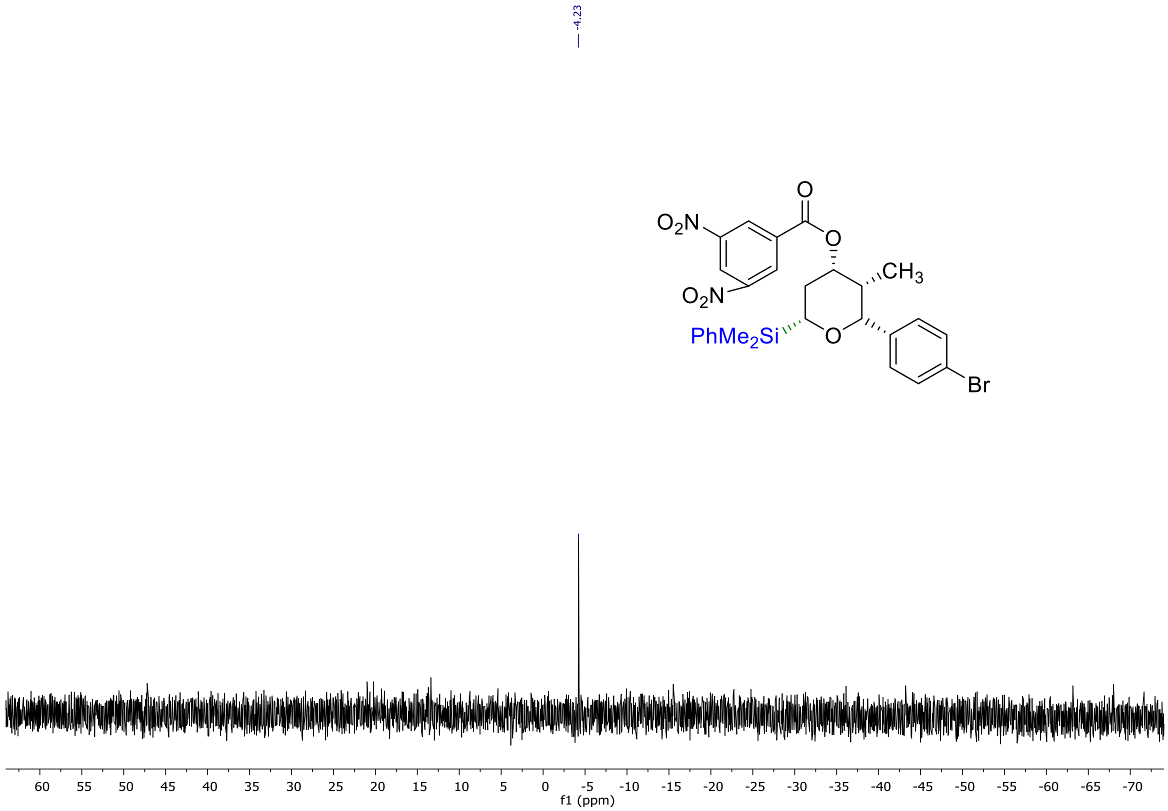

Supplementary Figure 178. ^{1}H and ^{13}C-NMR spectra of ***E*-21**

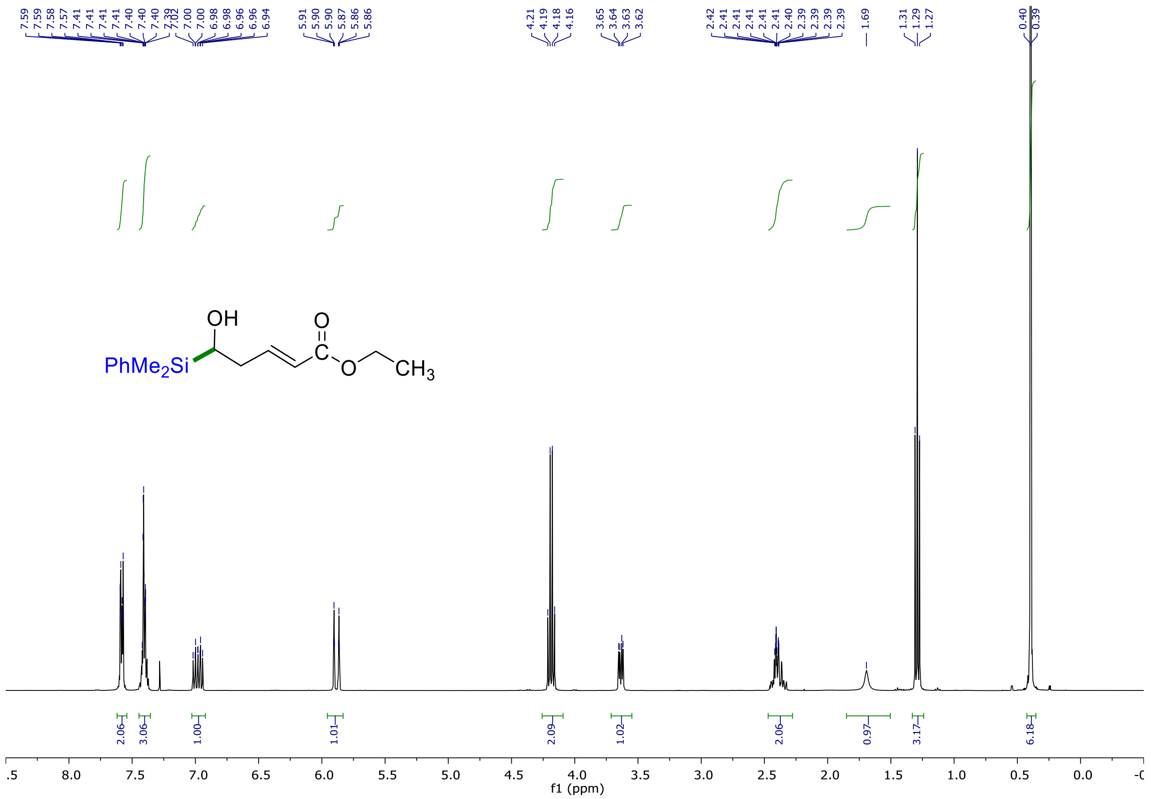

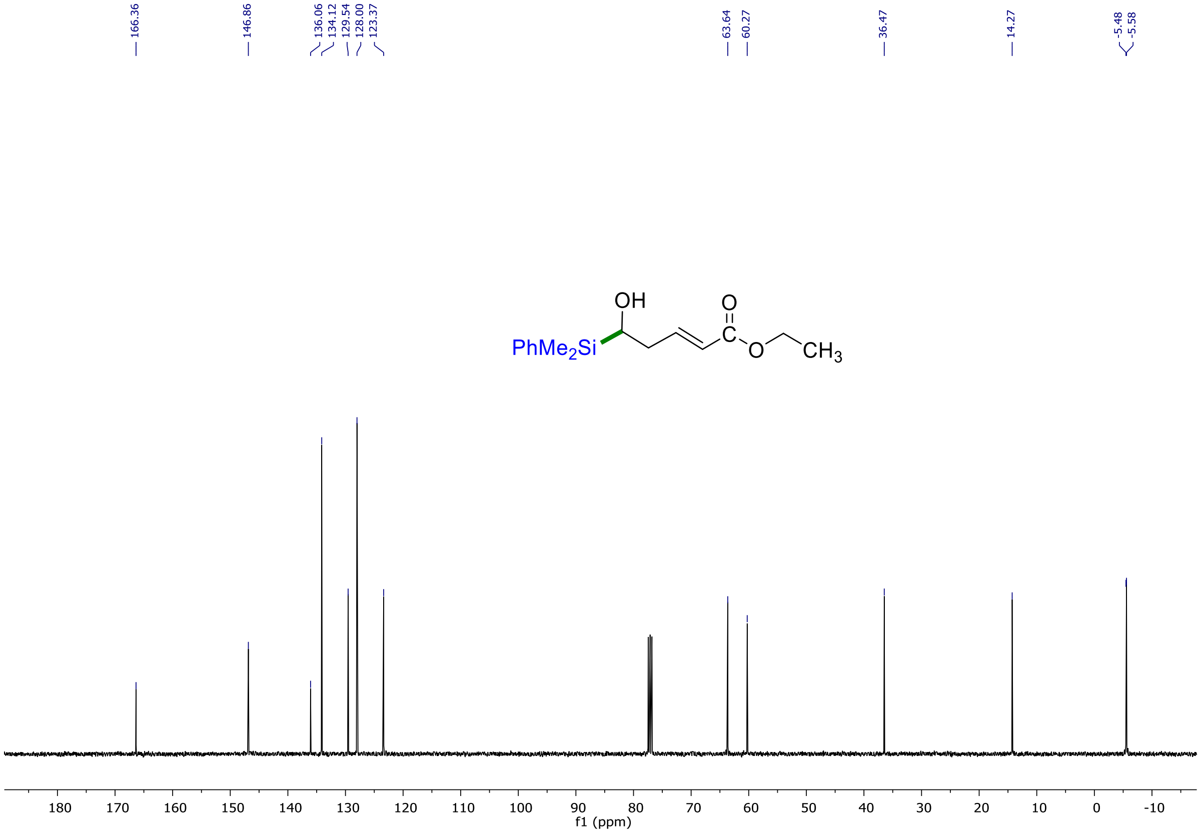

Supplementary Figure 179. ^{29}Si-NMR spectrum of ***E*-21**

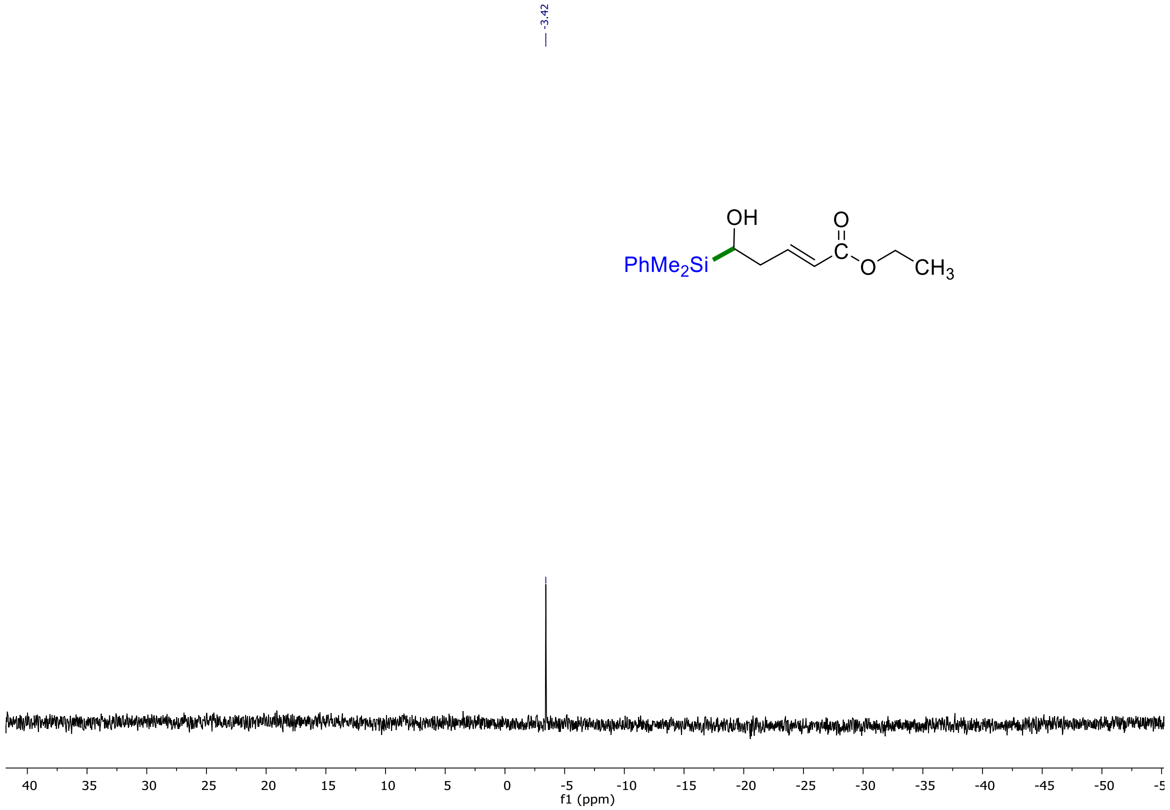

Supplementary Figure 180. ^{1}H-NMR spectrum of ***E*-22**

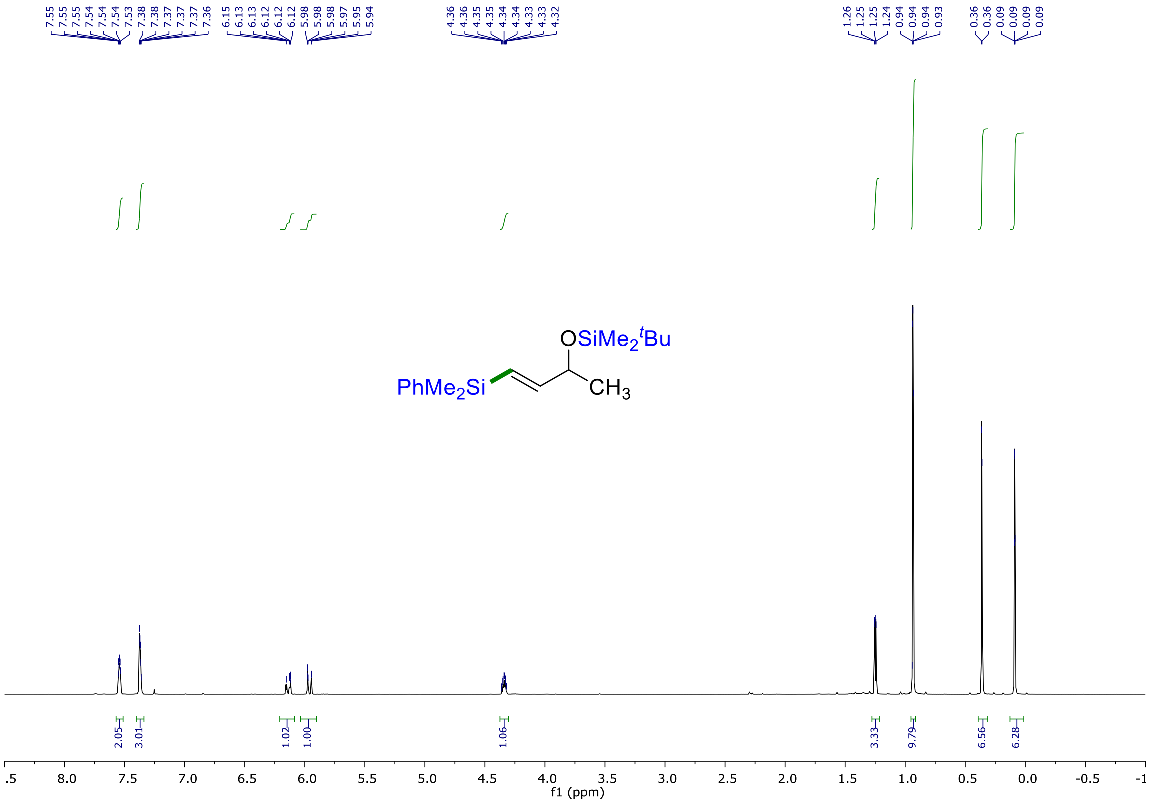

Supplementary Figure 181. ^{13}C and ^{29}Si-NMR spectra of ***E*-22**

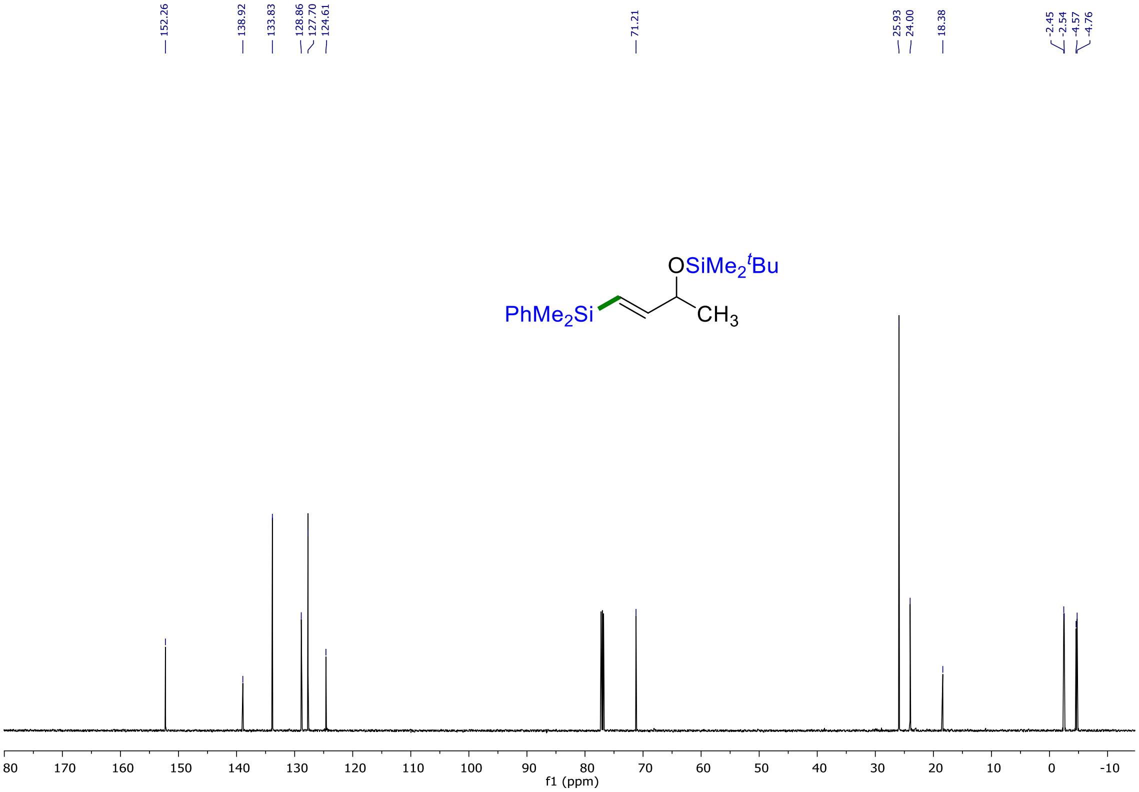

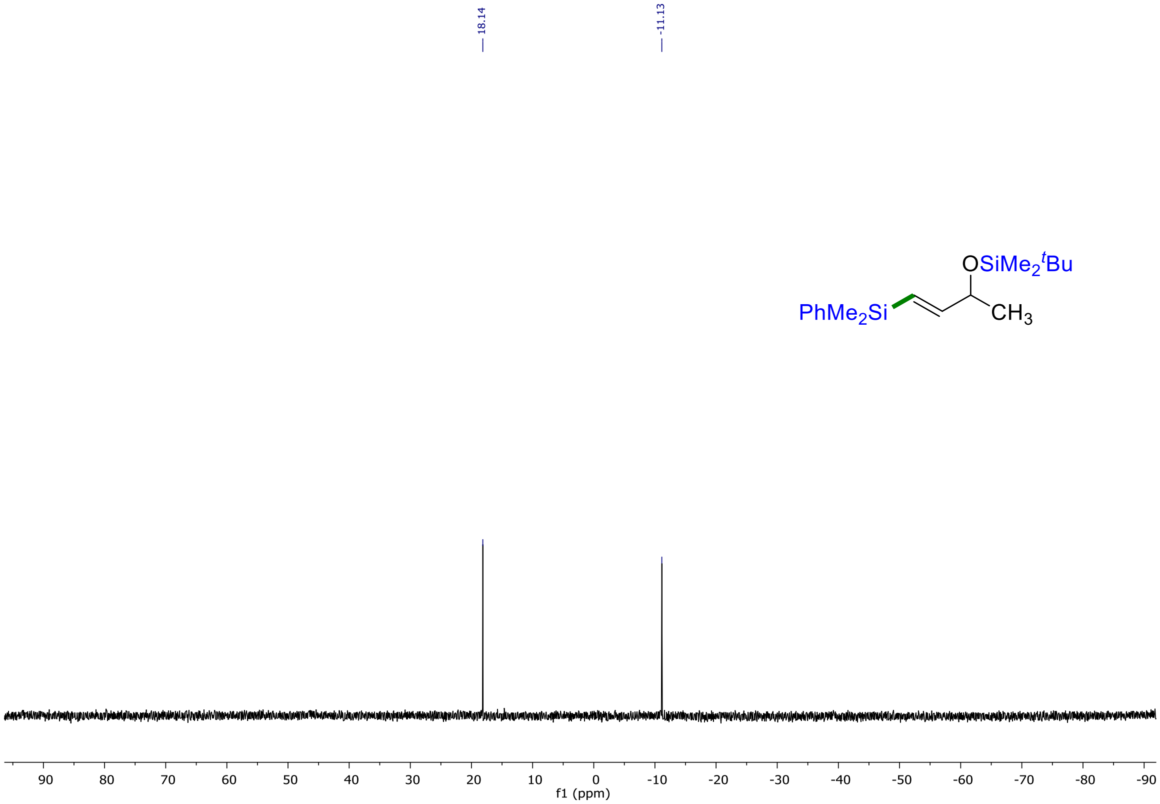

Supplementary Figure 182. ^{1}H and ^{13}C-NMR spectra of ***E*-23**

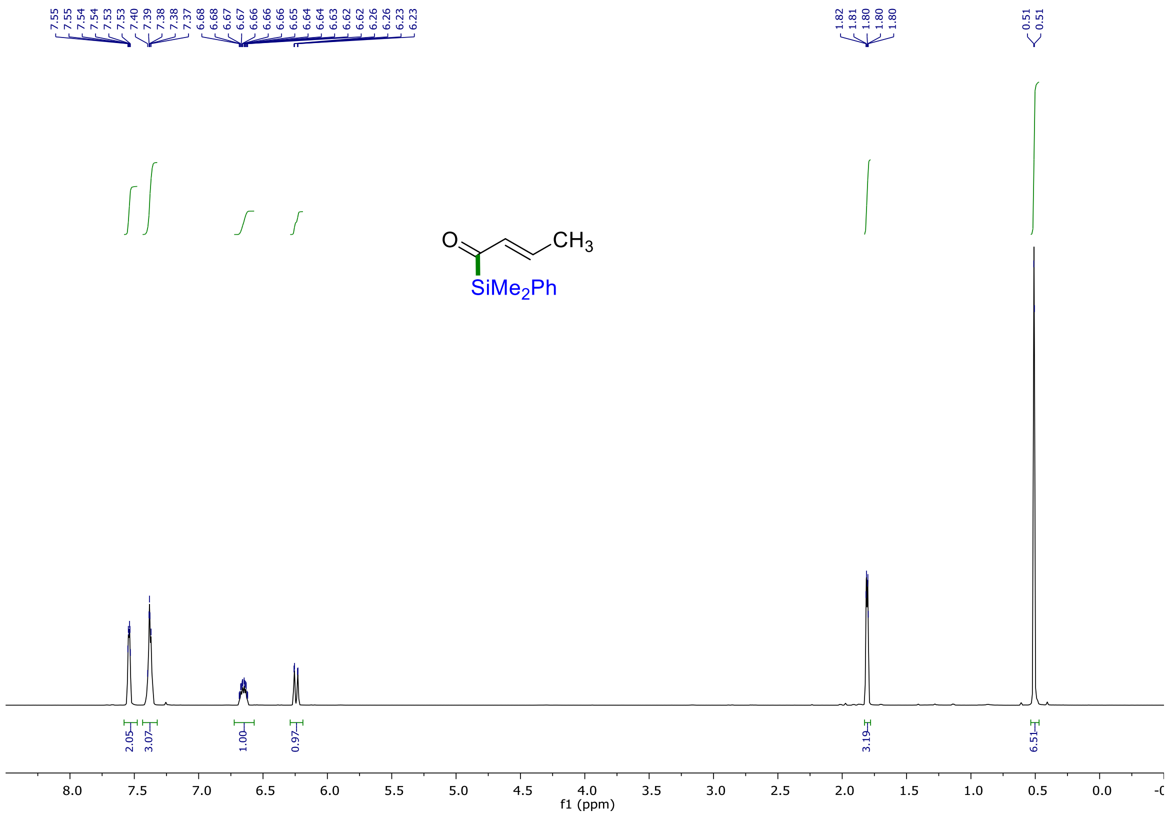

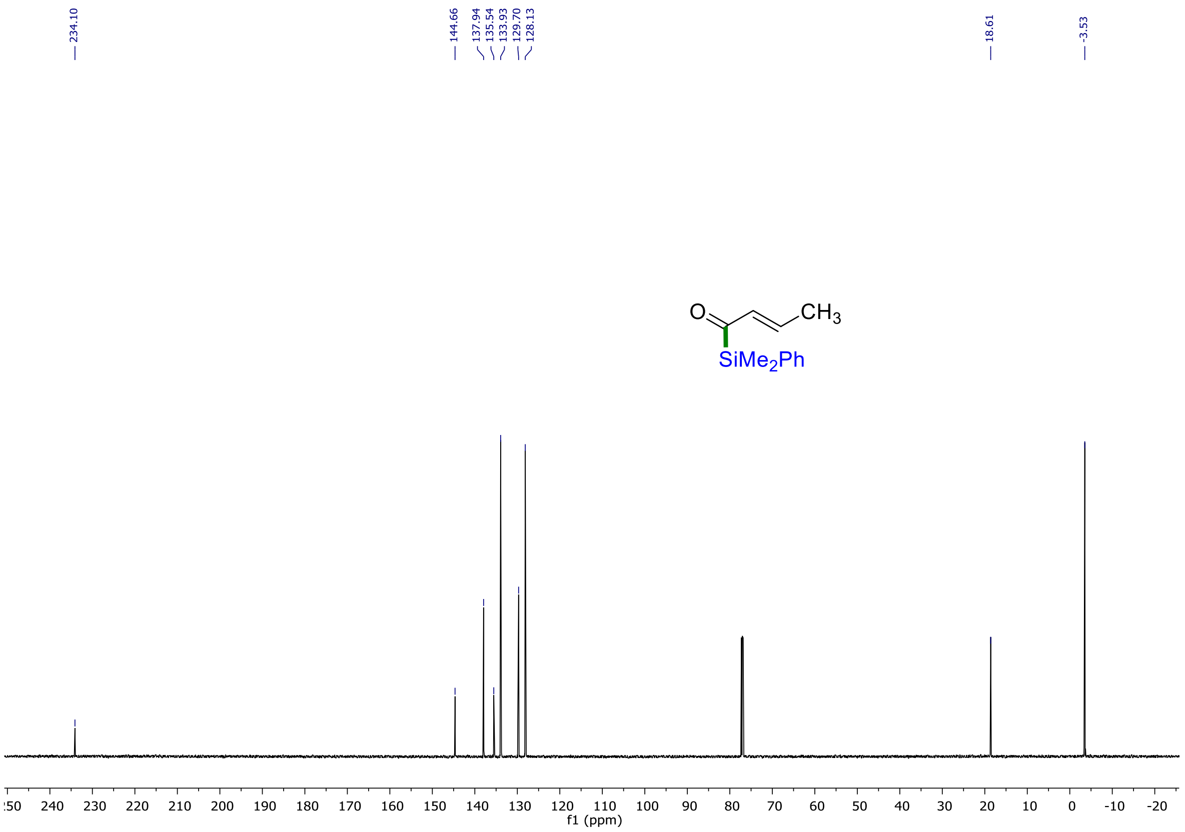

Supplementary Figure 183. ^{29}Si-NMR spectrum of ***E*-23**

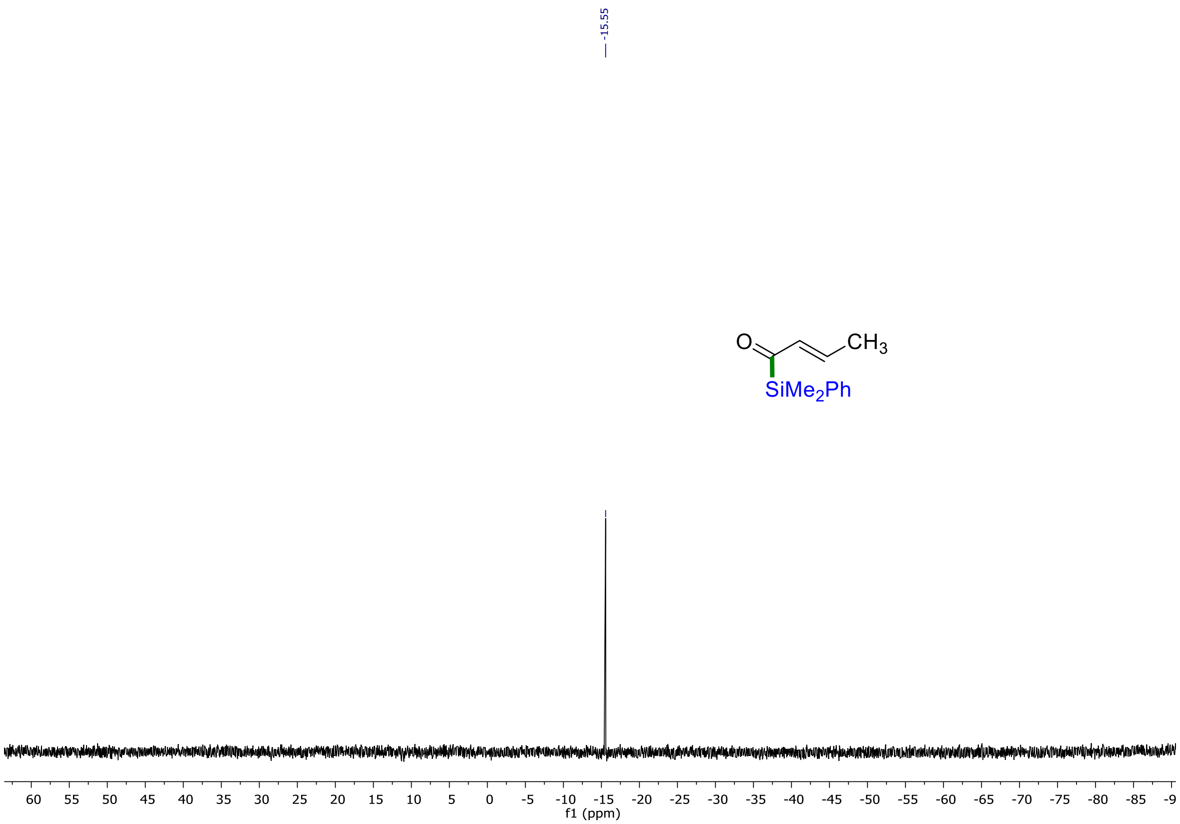

Supplementary Figure 184. ^{1}H-NMR spectrum of ***anti*-24**

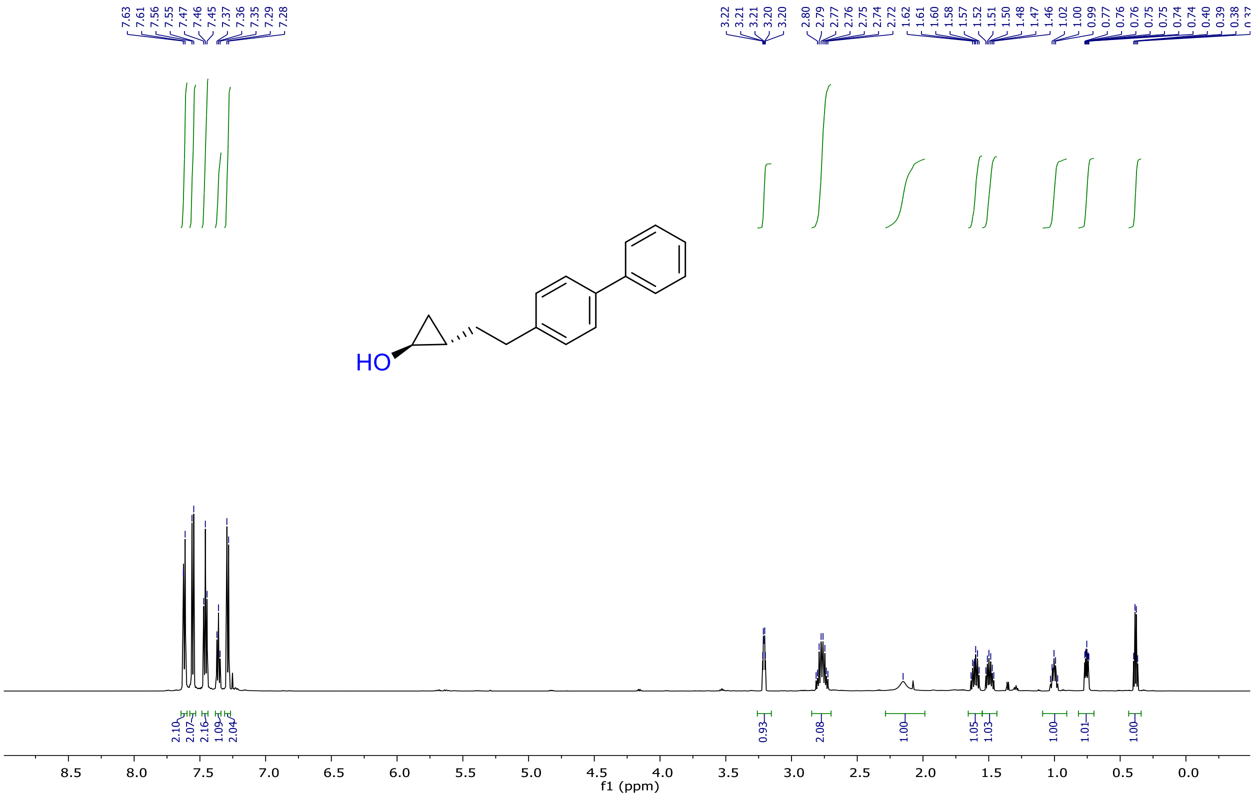

Supplementary Figure 185. ^{13}C-NMR spectrum of ***anti*-24**

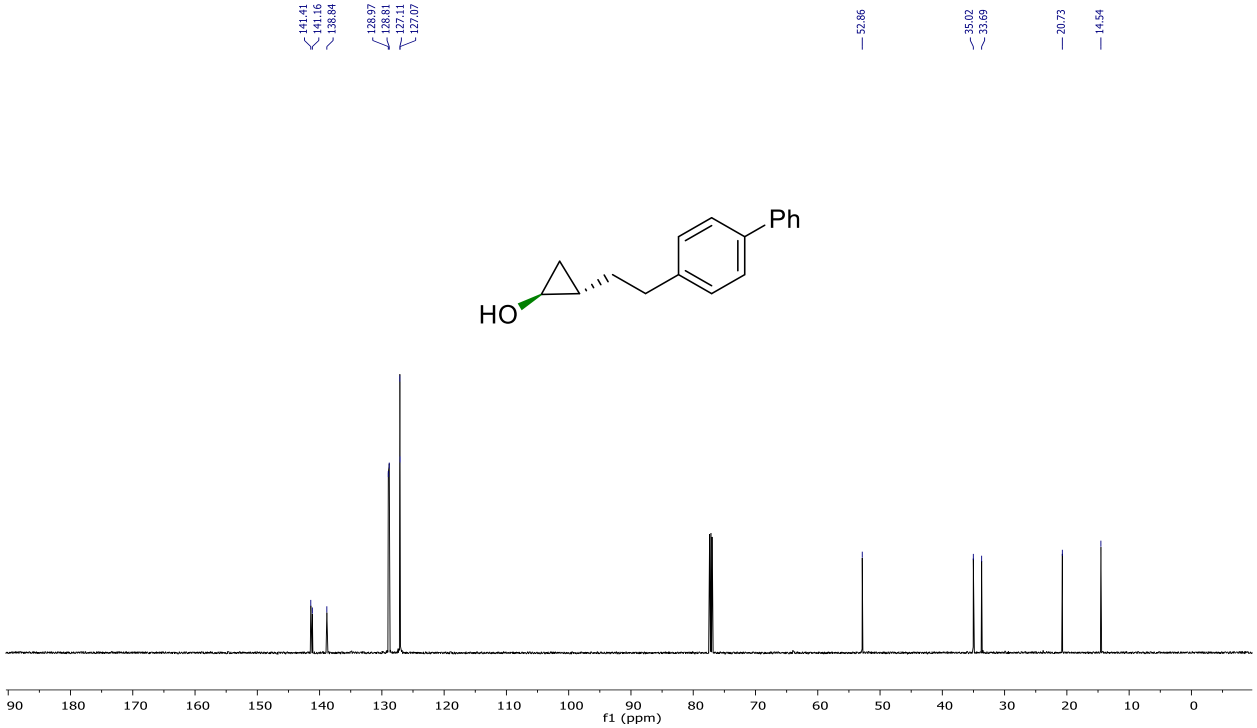

Supplementary Figure 186. ^{1}H-NMR spectrum of ***anti*-25**

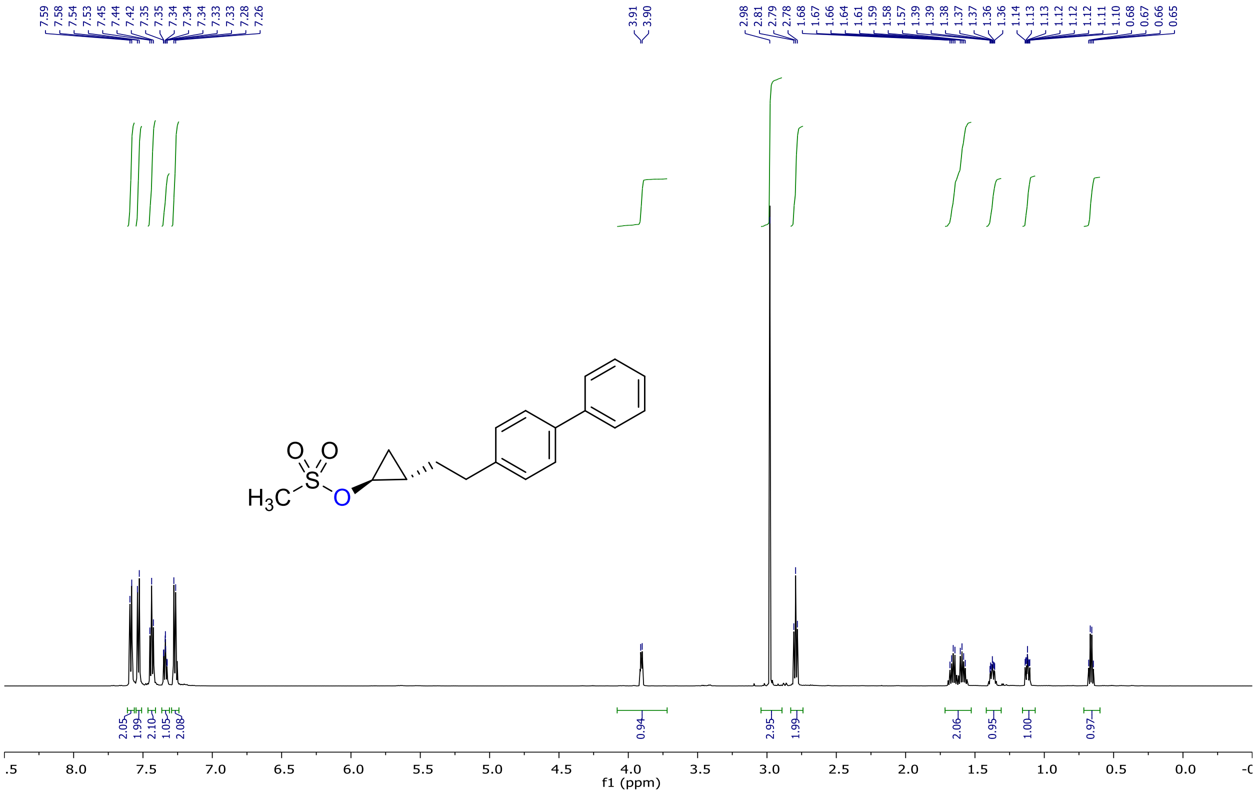

Supplementary Figure 187. ^{13}C-NMR spectrum of ***anti*-25**

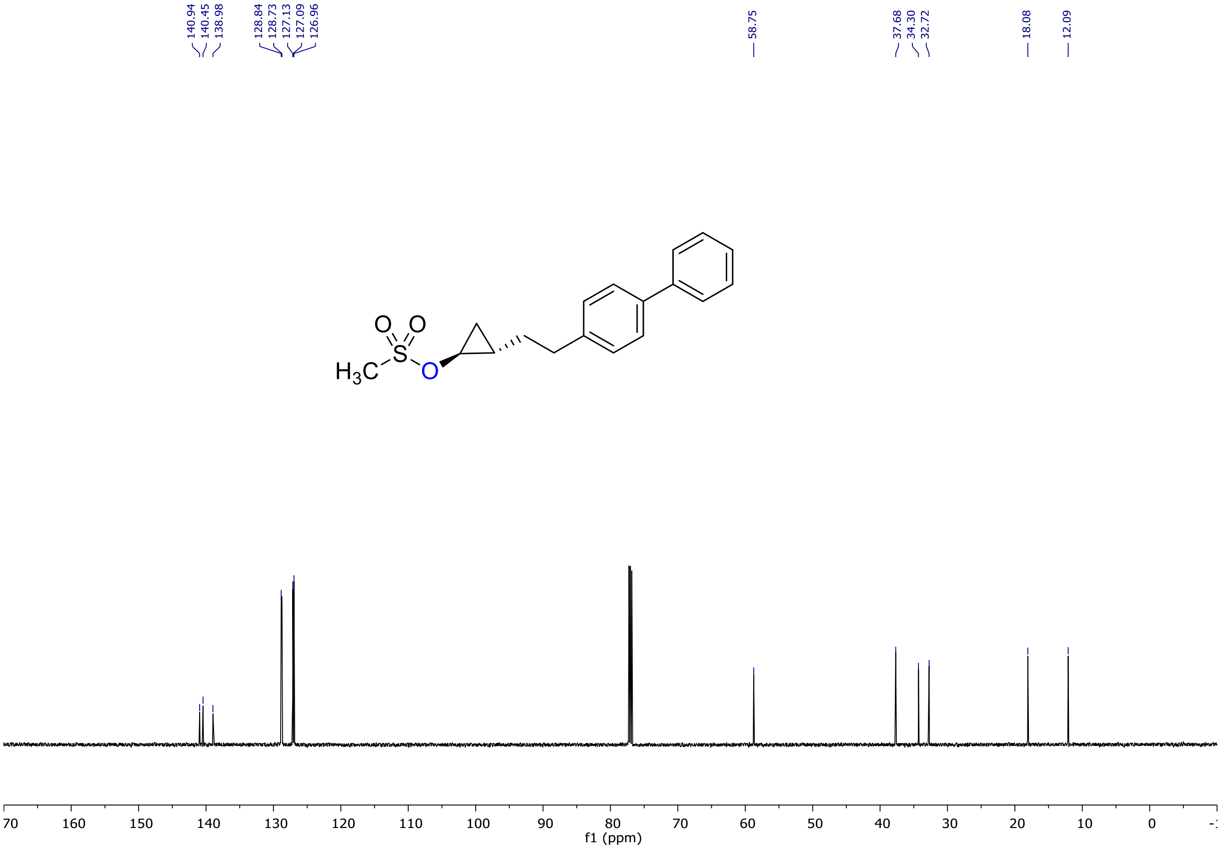

Supplementary Table 1. Optimization of the $B(C_6F_5)_3$-Catalyzed Silylative Reduction of Furans[a]

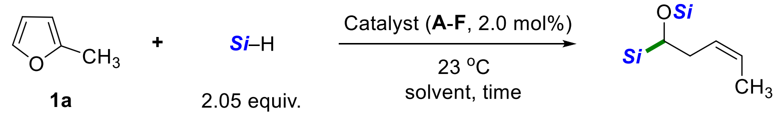

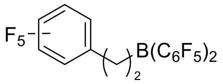

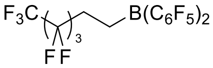

A[1]	B[1]	C[2-3]	D[2-3]	E[4-5]	F
(structure)	(structure)	$ClB(C_6F_5)_2$	$HB(C_6F_5)_2$	**$B(C_6F_5)_3$**	$B(C_6H_5)_3$

Entry	Silane	Catalyst	Solvent	Time (h)	Conv. (%)[b]	Yield (%)[b]
1	$PhMe_2SiH$	**A**	CD_2Cl_2	2	78	73
2	$PhMe_2SiH$	**B**	CD_2Cl_2	2	20	16
3	$PhMe_2SiH$	**C**	CD_2Cl_2	2	68	64
4	$PhMe_2SiH$	**D**	CD_2Cl_2	2	54	44
**5**	**$PhMe_2SiH$**	**E**	**CD_2Cl_2**	**2**	**>99**	**>99**
6	$PhMe_2SiH$	**E**	$CDCl_3$	2	>99	92
7	$PhMe_2SiH$	**E**	chlorobenzene-d_5	2	>99	80
8	$PhMe_2SiH$	**E**	toluene-d_8	2	92	50
9	$PhMe_2SiH$	No catalyst	CD_2Cl_2	24	<1	<1
10	Ph_2MeSiH	**E**	CD_2Cl_2	2	>99	91
11	Et_3SiH	**E**	CD_2Cl_2	2	>99	8
12	Ph_3SiH	**E**	CD_2Cl_2	2	60	<1
13	$PhMe_2SiH$	**F**	CD_2Cl_2	2	6	<1
14	$PhMe_2SiH$	**$AlCl_3$**	CD_2Cl_2	2	10	<1
15	$PhMe_2SiH$	**$[Ir(COD)Cl]_2$**	CD_2Cl_2	2	10	<1
16	$PhMe_2SiH$	**$[Rh(COD)Cl]_2$**	CD_2Cl_2	2	10	<1
17	$PhMe_2SiH$	**Pt**[c]	CD_2Cl_2	2	12	<1

[a]Carried out in a J-Young NMR tube on a 0.50 mmol scale (substrate), silane (1.03 mmol), and solvent (0.4 mL) under argon atmosphere at 23 °C. [b]Conversion and crude yields were determined by ^1H NMR spectroscopy using 1,1,2,2- tetrachloroethane (TCE) as an internal standard. [c]**Pt** = Pt(0)-1,3-divinyl-1,1,3,3-tetramethyl-disiloxane.

Supplementary Table 2. Optimization for the Cyclopropanation Reaction of (*Z*)-α-Silyloxyalkenylsilanes[a]

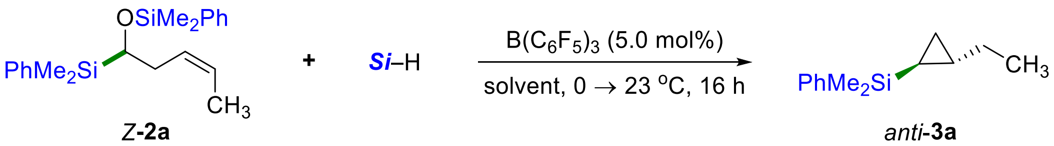

Entry	Silane (equiv.)	Solvent	Conv. (%)[b]	Yield (%)[b]
1	$PhMe_2SiH$ (1.0)	CD_2Cl_2	70	68
2	$PhMe_2SiH$ (1.0)	$CDCl_3$	60	54
3	$PhMe_2SiH$ (1.0)	chlorobenzene-d_5	62	60
5	$PhMe_2SiH$ (1.0)	toluene-d_8	68	64
5	$(i\text{Pr})_3SiH$ (1.0)	CD_2Cl_2	12	<1
6	Ph_2MeSiH (1.0)	CD_2Cl_2	10	<1
7	Ph_2SiH_2 (1.0)	CD_2Cl_2	78	76
8	Ph_3SiH (1.0)	CD_2Cl_2	<1	<1
9	Et_2SiH_2 (1.0)	CD_2Cl_2	90	84
**10**	**$PhMe_2SiH$ (1.5)**	**CD_2Cl_2**	**>99**	**84**
11	$PhMe_2SiH$ (2.0)	CD_2Cl_2	>99	88

[a]Carried out in a J-Young NMR tube on a 0.50 mmol (substrate), silane (0.5 ~ 1.0 mmol), and solvent (0.2 mL) under argon atmosphere. [b]Conversion and crude yields were determined by ^{1}H NMR spectroscopy using 1,1,2,2- tetrachloroethane (TCE) as an internal standard.

Supplementary Table 3. Optimization for the Cyclopropanation Reaction of 2-Methylfuran[a]

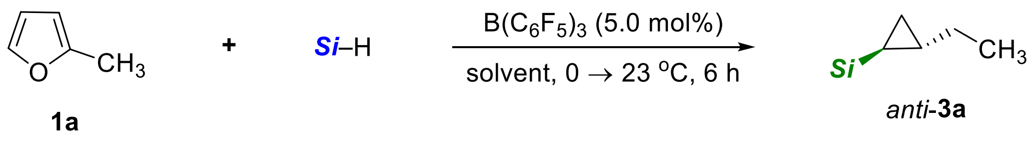

Entry	Silane (equiv.)	Solvent	Conv. (%)[b]	Yield (%)[b]
1	$PhMe_2SiH$ (3.0)	CD_2Cl_2	>99	84
2	$PhMe_2SiH$ (3.0)	$CDCl_3$	>99	48
3	$PhMe_2SiH$ (3.0)	chlorobenzene-d_5	>99	58
5	$PhMe_2SiH$ (3.0)	toluene-d_8	98	50
5	$(i\text{Pr})_3SiH$ (3.0)	CD_2Cl_2	2	<1
6	Ph_2MeSiH (3.0)	CD_2Cl_2	>99	<1
7	Ph_2SiH_2 (3.0)	CD_2Cl_2	98	86
8	Ph_3SiH (3.0)	CD_2Cl_2	94	<1
9	Et_2SiH_2 (3.0)	CD_2Cl_2	>99	74
**10**	**$PhMe_2SiH$ (4.0)**	**CD_2Cl_2**	**>99**	**96**

[a]Carried out in a J-Young NMR tube on a 0.50 mmol (substrate), silane (1.5 ~ 2.0 mmol), and solvent (0.2 mL) under argon atmosphere. [b]Conversion and crude yields were determined by ^{1}H NMR spectroscopy using 1,1,2,2- tetrachloroethane (TCE) as an internal standard.

Supplementary Table 4. Crystal data and structure refinement for *Z*-2a'' (CCDC 1505482)

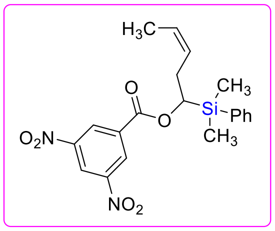

Identification code	0314b-1	
Empirical formula	$C_{20}H_{22}N_2O_6Si$	
Formula weight	414.49	
Temperature	120(2) K	
Wavelength	0.71073 Å	
Crystal system	Triclinic	
Space group	P-1	
Unit cell dimensions	a = 7.5090(4) Å	α= 70.390(3)°.
	b = 11.4202(7) Å	β= 79.619(3)°.
	c = 13.2271(8) Å	γ = 80.649(3)°.
Volume	1044.61(11) $Å^3$	
Z	2	
Density (calculated)	1.318 Mg/m^3	
Absorption coefficient	0.151 mm^{-1}	
F(000)	436	
Crystal size	0.13 x 0.09 x 0.09 mm^3	
Theta range for data collection	1.65 to 26.63°.	
Index ranges	-9<=h<=9, -14<=k<=14, -16<=l<=16	
Reflections collected	28370	
Independent reflections	4355 [R(int) = 0.0576]	
Completeness to theta = 26.63°	99.0 %	
Absorption correction	Semi-empirical from equivalents	
Max. and min. transmission	0.9866 and 0.9807	
Refinement method	Full-matrix least-squares on F^2	
Data / restraints / parameters	4355 / 0 / 262	
Goodness-of-fit on F^2	1.137	
Final R indices [I>2sigma(I)]	R1 = 0.0632, wR2 = 0.2104	
R indices (all data)	R1 = 0.0802, wR2 = 0.2238	
Largest diff. peak and hole	0.556 and -0.418 e.$Å^{-3}$	

Supplementary Table 5. Crystal data and structure refinement for *syn*-20' (CCDC 1505484)

Identification code	p21n	
Empirical formula	$C_{27}H_{27}BrN_2O_7Si$	
Formula weight	599.50	
Temperature	120(2) K	
Wavelength	0.71073 Å	
Crystal system	Monoclinic	
Space group	P 21/n	
Unit cell dimensions	a = 17.8456(17) Å	□= 90°.
	b = 7.9478(7) Å	□= 96.747(3)°.
	c = 19.1615(17) Å	□ = 90°.
Volume	2698.9(4) $Å^3$	
Z	4	
Density (calculated)	1.475 Mg/m^3	
Absorption coefficient	1.616 mm^{-1}	
F(000)	1232	
Crystal size	0.381 x 0.2 x 0.1 mm^3	
Theta range for data collection	2.78 to 27.54°.	
Index ranges	-23<=h<=23, -10<=k<=10, -24<=l<=24	
Reflections collected	40504	
Independent reflections	6213 [R(int) = 0.1202]	
Completeness to theta = 27.54°	99.8 %	
Absorption correction	Semi-empirical from equivalents	
Max. and min. transmission	0.9798 and 0.9505	
Refinement method	Full-matrix least-squares on F^2	
Data / restraints / parameters	6213 / 0 / 346	
Goodness-of-fit on F^2	0.892	
Final R indices [I>2sigma(I)]	R1 = 0.0447, wR2 = 0.1145	
R indices (all data)	R1 = 0.0943, wR2 = 0.1472	
Largest diff. peak and hole	0.427 and -0.604 e.$Å^{-3}$	

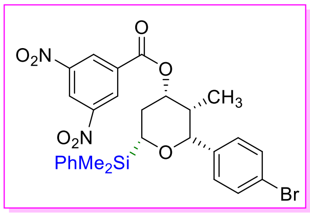

Supplementary Discussion 1

Proposed Mechanistic Pathway for the Silylative Reduction of Furan

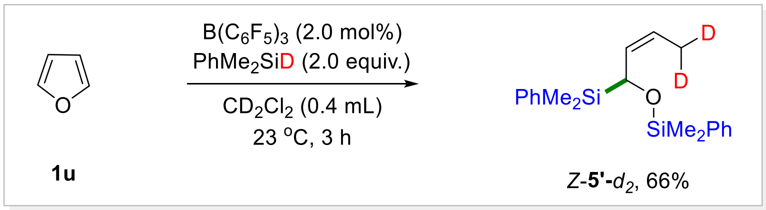

In regard to the silylative ring-opening of 2-methylfuran, a catalytic cycle was proposed on the basis of the above deuterium experimental data. Ring-opening of furan is assumed to proceed mainly via two steps: facile formation of a (silyl)oxonium intermediate and subsequent stereoselective attack of a borohydride nucleophile. Upon the *in situ* generation of a borane–silane adduct **I**, furan reacts with the silylium species **I** to give an oxonium species **II** that immediately undergoes a nucleophilic attack of a borohydride, leading to a partially reduced furan intermediate **III** bearing a sp^3 C-Si bond next to an oxygen atom. Subsequent C-O bond cleavage on **III** is proposed to occur via *O*-silyl oxonium species **IV** to produce (*Z*)-α-silyloxyalkenylsilane **V** in a highly stereoselective manner. The deuterium labeling experiment supports the current mechanistic reasoning.

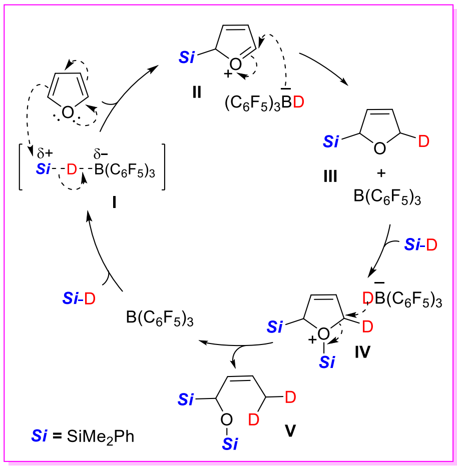

Supplementary Discussion 2

Proposed Mechanistic Pathway for the Silylative Reduction of 2,3-Dimethylfuran

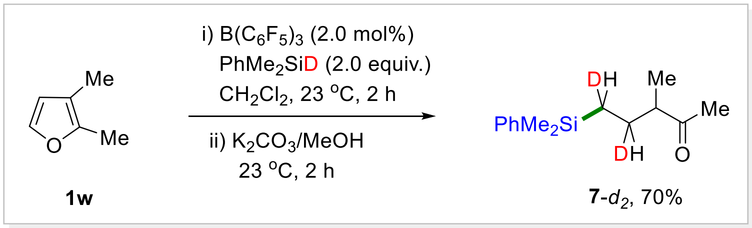

In relevance to the mechanistic pathway of 2-methylfuran, a catalytic cycle of ring-opening process of 2,3-dimethylfuran is proposed on the basis of the above present deuterium experiment. Ring-opening of 2,3-dimethylfuran is assumed to be mainly dependent on two factors: a facile formation of (silyl)oxonium intermediates and subsequent stereoselective attack of borohydride nucleophile. Upon the *in situ* generation of a borane–silane adduct **I**, 2,3-dimethylfuran attacks the silylium species to afford an oxonium species **II** that immediately reacts with borohydride leading to a partially reduced furan intermediate **III** bearing a sp^3 C-Si bond next to an oxygen atom. The second hydrosilylation of **III** is proposed to occur forming an *O*-silyl oxonium species **IV** which is immediately reacted with borohydride at the silicon attached carbon center leading to **V** to minimize the steric repulsion between substituents and borohydride nucleophile. Upon hydrolysis, intermediate **V** is smoothly converted to another silylated enol intermediate **VI** that is immediately tautomerized to γ-silylketone **VII**. In fact, the deuterium labeling experiment is fully consistent with this mechanistic reasoning.

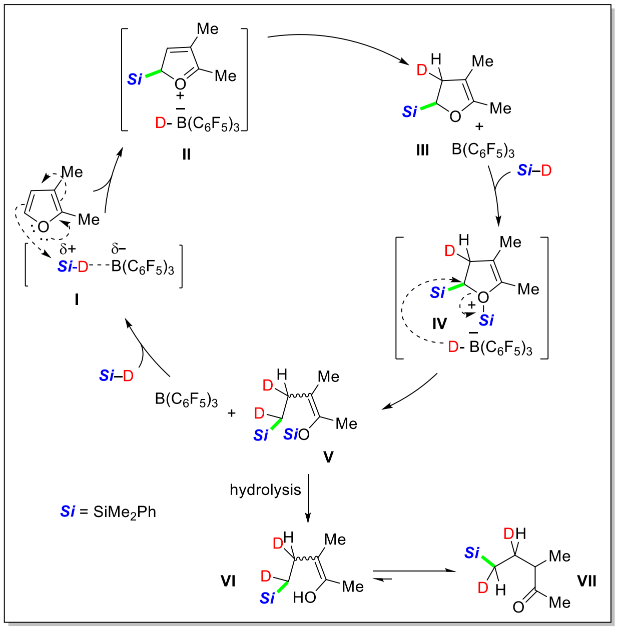

Supplementary Discussion 3

Proposed Mechanistic Pathway for the Silylative Reduction of 2-Methylbenzofuran

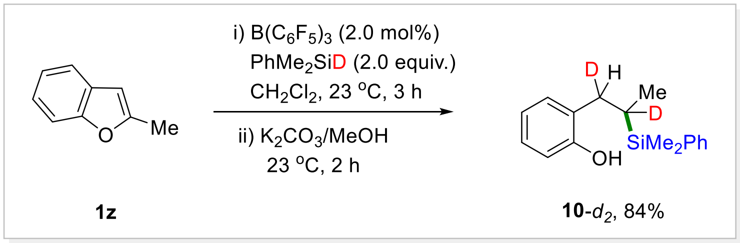

In relevance to the mechanistic pathway of 2-methylfuran, a catalytic cycle of ring-opening process of 2-methylbenzofuran is proposed on the basis of the above present deuterium experiment. Ring-opening of 2-methylbenzofuran is believed to be mainly dependent on two factors: a facile formation of (silyl)oxonium intermediate and subsequent stereoselective attack of borohydride nucleophile. Upon the *in situ* generation of a borane–silane adduct **I**, 2-methylbenzofuran attacks the silylium species to form an oxonium intermediate **II** that immediately reacts with borohydride leading to a silylated cyclic ether type species **III**. In the second stage, hydrosilylation of the silylated cyclic ether cleavage in **IV** is assumed to occur to afford *ortho*-(β-silylethyl)phenol derivative **V**. Phenol products (**10-**d_2) having an *ortho*-(β-silylethyl) group were obtained in high yields upon treating the initially generated unstable *O*-silyl ether species with potassium carbonate/methanol in one pot.

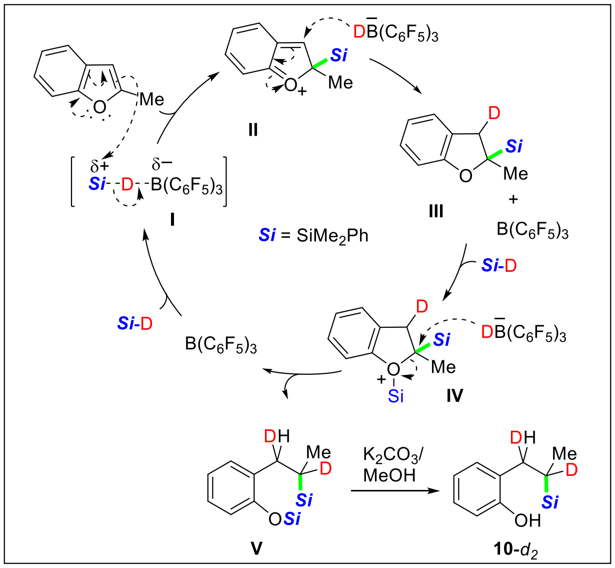

Supplementary Methods:

General

All reactions were performed in flame-dried glassware using sealed tube or NMR tube under argon atmosphere. Liquids and solutions were transferred with syringes. Dichloromethane-d_2 purchased from Cambridge Isotope Laboratories, Inc. was degassed and used as solvent without additional purification for optimization and mechanistic studies. Tris(pentafluorophenyl)borane was purchased from TCI and Sigma-Aldrich, stored at -20 ^{o}C. Technical grade solvents for extraction and chromatography (hexane, cyclohexane, dichloromethane, pentane, ether, toluene, and ethyl acetate) were used without purification. All reagents and commercially available catalysts were purchased from standard suppliers (Sigma Aldrich, Acros, Alfa Aesar, and Apollo scientific). The known borane species (**A**-**F**) were prepared according to the reported literature.[1-5] Analytical thin layer chromatography (TLC) was performed on pre-coated silica gel 60 F254 plates. Visualization on TLC was achieved by the use of UV light (254 nm), exposure to treatment with acidic anisaldehyde, phosphomolybdic acid, ninhydrin or ceric ammonium molybdate stain followed by heating. Column chromatography was undertaken on silica gel (400-630 mesh) using a proper eluent. ^{1}H, ^{2}H, ^{13}C, ^{19}F, and ^{29}Si NMR spectra were recorded in $CDCl_3$ or CD_2Cl_2 on Bruker Avance (400 MHz) and Agilent Technologies DD2 (600 MHz) instruments. Chemical shifts were reported in parts per million (ppm) downfield from tetramethylsilane and referenced to the residual solvent resonance as the internal standard ($CHCl_3$ in $CDCl_3$: 7.26, $CHCl_3$ in $CDCl_3$: δ = 77.17; $Cl_2CHCHCl_2$ in $Cl_2CDCDCl_2$, 5.85; $Cl_2CHCHCl_2$ in $Cl_2CDCDCl_2$: 75.5; CH_2Cl_2 in CD_2Cl_2: 5.32, CH_2Cl_2 in CD_2Cl_2: 53.84). Data were reported as follows: chemical shift, multiplicity (brs = broad singlet, s = singlet, d = doublet, t = triplet, q = quartet, m = multiplet), coupling constant (Hz) and integration. Infrared (IR) spectra were recorded on Bruker Alpha ATR FT-IR spectrometer equipped with an ATR unit and reported in wavenumbers (cm^{-1}). Melting points (m.p.) were determined for solid/crystalline compounds with a Melting Point Apparatus M-565. High resolution mass spectra (HRMS) were obtained by using EI or FAB method from Korea Basic Science Institute (Daegu) or ESI from KAIST Research Analysis Center (Daejeon).

Preparation Details for Furans and Benzofurans as the Substrates

General Procedure for the Preparation of Furan and Benzofuran Substrates (GP1)

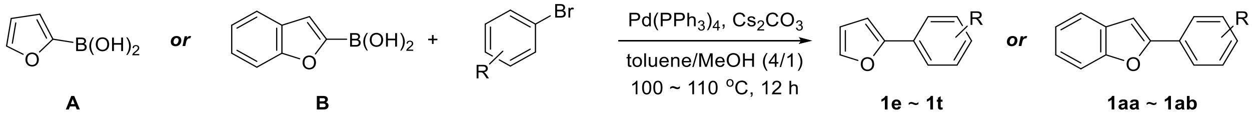

A round bottom flask was charged with aryl bromide (2 mmol = 1.0 equiv.), boronic acid (1.1 ~ 1.5 equiv.), $Pd(PPh_3)_4$ (5 ~ 10 mol%), and Cs_2CO_3 (1.1 ~ 1.4 equiv.), into which toluene (16 mL) and MeOH (4 mL) were subsquently added. Then, the reaction mixture was heated in an oil bath at 100 ~ 110 °C until a TLC analysis of an aliquot indicated a complete conversion of the starting materials (12 h). The reaction mixture was then cooled to 23 °C and quenched with saturated NH_4Cl solution. Aqueous phase was extracted with ethyl acetate (10 mL x 3), and the combined organic phases were washed with brine (10 mL), dried over $MgSO_4$, filtrated and concentrated under reduced pressure to afford the crude furan or benzofuran derivatives. Purification by flash column chromatography on silica gel using either hexane or the mixture of hexane and ethyl acetate gave the corresponding coupling products.[6]

Characterization Data of the Prepared Furan and Benzofuran Substrates

2-[4-(Trifluoromethyl)phenyl]furan (Table 1, 1e, 84%). [**A** + 1-bromo-4-(trifluoromethyl)benzene];

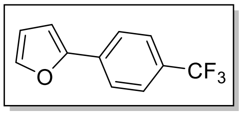

The compound **1e** was prepared according to **GP1** and was purified by flash column chromatography (hexane); colorless solid; m.p. 90–92 °C; ^{1}H NMR (600 MHz, $CDCl_3$): δ 7.90–7.73 (m, 2H), 7.73–7.61 (m, 2H), 7.53 (s, 1H), 6.80 (s, 1H), 6.53 (s, 1H); ^{13}C NMR (100 MHz, $CDCl_3$): δ 152.7, 143.2, 134.1, 129.2 (q, J = 32.3 Hz), 125.8 (q, *J* = 4 Hz), 124.3 (q, J = 272.7 Hz), 123.9, 112.1, 107.1; ^{19}F NMR (375 MHz, $CDCl_3$): δ -62.5; HRMS: (EI, m/z): calcd. for $C_{11}H_7F_3O$ $[M]^+$: 212.0449; found: 212.0447.

2-(4-Fluorophenyl)furan (Table 1, 1f, 84%). [**A** + 1-bromo-4-fluorobenzene]; The compound **1f** was

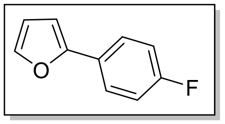

prepared according to **GP1** and was purified by flash column chromatography (hexane); colorless solid; m.p. 32–34 °C; ^{1}H NMR (600 MHz, $CDCl_3$): δ 7.73–7.58 (m, 2H), 7.54–7.40 (m, 1H), 7.09 (t, J = 8.7 Hz, 2H), 6.59 (d, J = 3.4 Hz, 1H), 6.48 (dd, J = 3.3, 1.8 Hz, 1H); ^{13}C NMR (150 MHz, $CDCl_3$): δ 162.3 (d, J = 247.6 Hz), 153.3, 142.1, 127.4 (d, J = 3.0 Hz), 125.7 (d, J = 7.6 Hz), 115.8 (d, J = 22.7 Hz), 111.8, 104.8; ^{19}F NMR (565 MHz, $CDCl_3$): δ -114.4; HRMS (EI, m/z): calcd. for $C_{10}H_7FO$ $[M]^+$: 162.0481; found: 162.0483.

2-(4-Chlorophenyl)furan (**Table 1**, **1g**, 90%). [**A** + 1-bromo-4-chlorobenzene]; The compound **1g** was prepared according to **GP1** and was purified by flash column chromatography (hexane); colorless solid; m.p. 64–66 °C; ^{1}H NMR (600 MHz, $CDCl_3$): δ 7.65–7.58 (m, 2H), 7.43 (d, J = 1.9 Hz, 1H), 7.41–7.27 (m, 2H), 6.64 (d, J = 3.4 Hz, 1H), 6.48 (dd, J = 3.5, 1.8 Hz, 1H); ^{13}C NMR (150 MHz, $CDCl_3$): δ 153.1, 142.5, 133.1, 129.5, 129.0, 125.2, 111.9, 105.6; HRMS (EI, m/z): calcd. for $C_{10}H_7ClO$ $[M]^+$: 178.0185; found: 178.0186.

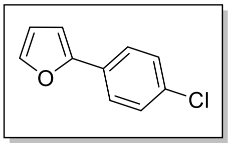

2-(4-Bromophenyl)furan (Table 1, **1h**, 52%**)**. [**A** + 1,4-dibromobenzene]; The compound **1h** was prepared according to **GP1** and was purified by flash column chromatography (hexane); colorless solid; m.p. 75–77 °C; ^{1}H NMR (600 MHz, $CDCl_3$): δ 7.54 (d, J = 8.5 Hz, 2H), 7.50 (d, J = 8.5 Hz, 2H), 7.47 (s, 1H), 6.66 (s, 1H), 6.47 (s, 1H); ^{13}C NMR (150 MHz, $CDCl_3$): δ 153.1, 142.5, 131.9, 129.9, 125.4, 121.2, 111.9, 105.7; HRMS (EI): calcd. for $C_{10}H_7BrO$ $[M]^+$: 221.9680; Found: 221.9678.

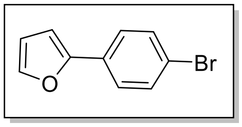

2-(1,1'-Biphenyl-4-yl)furan (Table 1, **1i**, 85%**)**. [**A** + 4-bromo-1,1'-biphenyl]; The compound **1i** was prepared according to **GP1** and was purified by flash column chromatography (hexane); colorless solid; m.p. 156–158 °C; ^{1}H NMR (400 MHz, $CDCl_3$): δ 7.86–7.79 (m, 2H), 7.73–7.70 (m, 2H), 7.71–7.68 (m, 2H), 7.57 (dd, J = 1.8, 0.8 Hz, 1H), 7.55–7.49 (m, 2H), 7.47–7.39 (m, 1H), 6.76 (dd, J = 3.3, 0.8 Hz, 1H), 6.57 (dd, J = 3.4, 1.8 Hz, 1H); ^{13}C NMR (100 MHz, $CDCl_3$): δ 153.8, 142.2, 140.6, 140.0, 129.9, 128.9, 127.42, 127.41, 126.9, 124.3, 111.8, 105.2; HRMS (EI, m/z): calcd. for $C_{16}H_{12}O$ $[M]^+$: 220.0888; found: 220.0890.

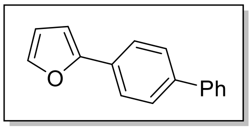

2-Phenylfuran (Table 1, **1j**, 92%**)**. [**A** + bromobenzene]; The compound **1j** was prepared according to **GP1** and was purified by flash column chromatography (hexane); colorless liquid; ^{1}H NMR (600 MHz, $CDCl_3$): δ 7.77–7.67 (m, 2H), 7.51 (d, J = 1.8 Hz, 1H), 7.42 (t, J = 7.8 Hz, 2H), 7.30 (t, J = 7.2, Hz, 1H), 6.69 (d, J = 3.3 Hz, 1H), 6.51 (dd, J = 3.4, 1.8 Hz, 1H); ^{13}C NMR (150 MHz, $CDCl_3$): δ 154.1, 142.2, 131.0, 128.8, 127.5, 123.9, 111.8, 105.1. HRMS (EI): calcd. for $C_{10}H_8O$ $[M]^+$: 144.0575; Found: 144.0576.[6]

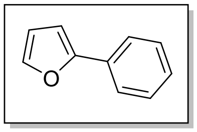

2-(*m*-Tolyl)furan (**Table 1**, **1k**, 86%**)**. [**A** + 1-bromo-3-methylbenzene]; The compound **1k** was prepared according to **GP1** and was purified by flash column chromatography (hexane); colorless liquid; ^{1}H NMR (600 MHz, $CDCl_3$): δ 7.57–7.45 (m, 3H), 7.29 (s, 1H), 7.09 (d, J = 7.9 Hz, 1H), 6.65 (d, J = 3.0 Hz, 1H), 6.48 (d, J = 1.7 Hz, 1H), 2.40 (s, 3H); ^{13}C NMR (150 MHz, $CDCl_3$): δ 154.1, 141.9, 138.2, 130.8, 128.5, 128.1, 124.4, 120.9, 111.5, 104.8, 21.5.[7]

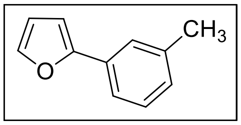

2-(4-Phenoxyphenyl)furan (Table 1, 1l, 91%**)**. [**A** + 1-bromo-4-phenoxybenzene]; The compound **1l**

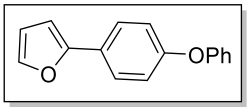

was prepared according to **GP1** and was purified by flash column chromatography (hexane); colorless solid; m.p. 63–65 °C; ^{1}H NMR (600 MHz, $CDCl_3$): δ 7.65 (d, J = 8.4 Hz, 2H), 7.46 (s, 1H), 7.36 (t, J = 7.7 Hz, 2H), 7.13 (t, J = 7.4 Hz, 1H), 7.04 (dd, J = 8.2, 5.8 Hz, 4H), 6.59 (s, 1H), 6.48 (s, 1H); ^{13}C NMR (150 MHz, $CDCl_3$): δ 157.2, 156.8, 153.8, 141.9, 129.9, 126.5, 125.5, 123.5, 119.2, 119.1, 111.8, 104.4; HRMS (EI, m/z): calcd. for $C_{16}H_{12}O_2$ $[M]^+$: 236.0837; found: 236.0835.

2-[4-(Methylthio)phenyl]furan (Table 1, 1m, 83%**)**. [**A** + (4-bromophenyl)(methyl)sulfane]; The

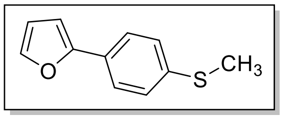

compound **1m** was prepared according to **GP1** and was purified by flash column chromatography (hexane); brown colored solid; m.p. 79–81 °C; ^{1}H NMR (600 MHz, $CDCl_3$): δ 7.63 (d, J = 8.2, Hz, 2H), 7.48–7.40 (m, 1H), 7.26 (d, J = 8.4 Hz, 2H), 6.60 (t, J = 3.3 Hz, 1H), 6.48–6.42 (m, 1H), 2.49 (s, 3H); ^{13}C NMR (150 MHz, $CDCl_3$): δ 153.6, 141.8, 137.5, 127.9, 126.8, 124.2, 111.6, 104.6, 15.8; HRMS (EI, m/z): calcd. for $C_{11}H_{10}OS$ $[M]^+$: 190.0452; found: 190.0452.

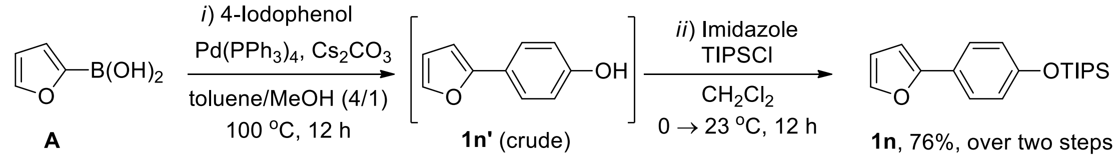

[4-(Furan-2-yl)phenoxy]triisopropylsilane (Table 1, 1n, 76%**)**. (*i*) *Cross-coupling reaction*: A round

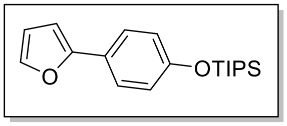

bottom flask was charged with 4-iodophenol (2.0 mmol, 1.0 equiv.), boronic acid (**A**, 1.02 equiv.), $Pd(PPh_3)_4$ (10 mol%), and Cs_2CO_3 (1.07 equiv.), into which toluene (16 mL) and MeOH (4 mL) were subsequently added. Then, the reaction mixture was heated in an oil bath at 100 °C until a TLC analysis of an aliquot indicated a complete conversion of the starting materials (12 h). The reaction mixture was then cooled to 23 °C and quenched with saturated NH_4Cl solution. Aqueous phase was extracted with ethyl acetate (5 mL x 3), and the combined organic phases were washed with brine (5 mL), dried over $MgSO_4$, filtered and concentrated under reduced pressure to give **1n'**, and this resulting residue was subjected to *O*-silylation.

(*ii*) *O-silylation*: The crude reaction mixture of **1n'** was diluted with *anhydrous* CH_2Cl_2 and cooled to 0 °C, at which imidazole (1.3 equiv.) was added in one portion. This solution was briefly stirred for 10 min, into which triisopropylsilyl chloride (1.2 equiv.) in CH_2Cl_2 (2 mL) was finally added at 0 °C. Then, the reaction mixture was allowed to react at 23 °C. After 12 h, the reaction was quenched with saturated aqueous NH_4Cl solution (5 mL), and the aqueous phase was extracted with dichloromethane (5 mL x 3). The combined organic phases were washed with brine (5 mL), dried over anhydrous Na_2SO_4 and

concentrated under reduced pressure. Purification of this crude reaction mixture by flash column chromatography on silica gel using cyclohexane as an eluent eventually afforded the desired product **1n** in 76% yield over 2 steps. Colorless liquid; ^{1}H NMR (600 MHz, $CDCl_3$): δ 7.61–7.52 (m, 2H), 7.47–7.40 (m, 1H), 7.01–6.88 (m, 2H), 6.52 (d, J = 3.4 Hz, 1H), 6.45 (dd, J = 3.4, 1.8 Hz, 1H), 1.34–1.25 (m, 3H), 1.14 (d, J = 7.4 Hz, 18H); ^{13}C NMR (150 MHz, $CDCl_3$): δ 155.8, 154.3, 141.5, 125.3, 124.5, 120.3, 111.7, 103.5, 18.1, 12.9; ^{29}Si NMR (120 MHz, $CDCl_3$): δ 15.7; HRMS (EI, m/z): calcd. for $C_{19}H_{28}O_2Si$ $[M]^+$: 316.1859; found: 316.1858.

2-(3,5-Dibromophenyl)furan (Table 1, 1o, 56%**).** [**A** + 1,3,5-tribromobenzene]; The compound **1o** was prepared according to **GP1** and was purified by flash column chromatography (hexane); colorless solid; m.p. 44–46 °C; ^{1}H NMR (600 MHz, $CDCl_3$): δ 7.72 (t, J = 1.6 Hz, 2H), 7.52 (d, J = 1.8 Hz, 1H), 7.47 (s, 1H), 6.68 (dd, J = 3.2, 1.6 Hz, 1H), 6.52–6.42 (m, 1H); ^{13}C NMR (150 MHz, $CDCl_3$): δ 150.8, 143.2, 133.9, 132.3, 125.3, 123.24, 111.9, 107.1; HRMS (EI, m/z): calcd. for $C_{10}H_6Br_2O$ $[M]^+$: 299.8785; found: 299.8786.

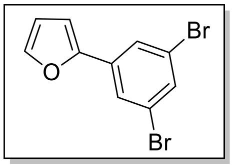

2-(2,4,6-Triisopropylphenyl)furan (Table 1, 1p, 28%**).** [**A** + 2-bromo-1,3,5-triisopropylbenzene]; The compound **1p** was prepared according to **GP1** and was purified by flash column chromatography (hexane); colorless solid; m.p. 103–105 °C; ^{1}H NMR (600 MHz, $CDCl_3$): δ 7.53 (s, 1H), 7.10 (s, 2H), 6.50 (s, 1H), 6.29 (s, 1H), 3.06–2.88 (m, 1H), 2.80–2.63 (m, 2H), 1.33 (d, J = 6.9 Hz, 6H), 1.19 (d, J = 6.9 Hz, 12H); ^{13}C NMR (150 MHz, $CDCl_3$): δ 152.3, 150.2, 149.7, 141.6, 126.8, 120.8, 110.3, 109.4, 34.6, 30.9, 24.4, 24.2; HRMS (EI, m/z): calcd. for $C_{19}H_{26}O$ $[M]^+$: 270.1984; found: 270.1982.

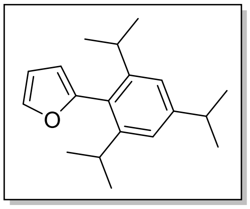

2-(Phenanthren-9-yl)furan (Table 1, 1q, 79%**).** [**A** + 9-bromophenanthrene]; The compound **1q** was prepared according to **GP1** and was purified by flash column chromatography (hexane); colorless solid; m.p. 86–88 °C; ^{1}H NMR (600 MHz, $CDCl_3$): δ 8.87–8.78 (m, 1H), 8.76–8.70 (m, 1H), 8.66–8.57 (m, 1H), 8.15 (d, J = 3.4 Hz, 1H), 8.00 (dd, J = 7.9, 3.4 Hz, 1H), 7.82–7.65 (m, 5H), 6.91 (s, 1H), 6.73 (s, 1H); ^{13}C NMR (150 MHz, $CDCl_3$): δ 153.5, 142.5, 131.4, 130.9, 130.3, 129.7, 129.0, 127.5, 127.4, 127.1, 126.9, 126.9, 126.7, 126.4, 123.1, 122.6, 111.5, 109.7; HRMS (EI, m/z): calcd. for $C_{18}H_{12}O$ $[M]^+$: 244.0888; found: 244.0887.

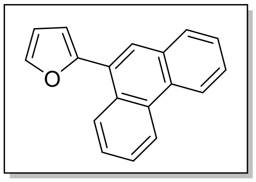

1,4-Di(furan-2-yl)benzene (Table 1, 1r, 50%**).** [**A** (2.0 equiv.) + 1,4-dibromobenzene (1.0 equiv.)]; The compound **1r** was prepared according to **GP1** and was purified by flash column chromatography (hexane); yellow color solid; m.p. 135–140 °C; ^{1}H NMR (600

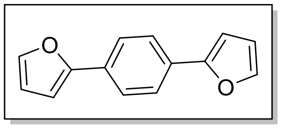

MHz, $CDCl_3$): δ 7.83–7.61 (m, 4H), 7.47 (d, J = 1.8 Hz, 2H), 6.66 (d, J = 3.3 Hz, 2H), 6.48 (dd, J = 3.4, 1.8 Hz, 2H); ^{13}C NMR (150 MHz, $CDCl_3$): δ 153.9, 142.3, 129.9, 124.2, 111.9, 105.3; HRMS (EI, m/z): calcd. for $C_{14}H_{10}O_2$ $[M]^+$: 210.0681; found: 210.0677.

2,2'-(5-Bromo-1,3-phenylene)difuran (Table 1, 1s, 55%**)**. [**A** (2.0 equiv.) + 1,3,5-tri-bromobenzene (1.0 equiv.)]; The compound **1s** was prepared according to **GP1** and was purified by flash column chromatography (hexane); colorless solid; m.p. 77–79 °C; ^1H NMR (600 MHz, $CDCl_3$): δ 7.86 (d, J = 2.0 Hz, 1H), 7.67 (t, J = 1.7 Hz, 2H), 7.49 (d, J = 2.6 Hz, 2H), 6.71 (t, J = 2.7 Hz, 2H), 6.49 (dt, J = 3.4, 1.8 Hz, 2H); ^{13}C NMR (150 MHz, $CDCl_3$): δ 152.1, 142.7, 132.9, 125.2, 123.1, 117.5, 111.8, 106.4.

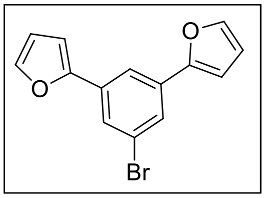

1,3,5-Tri(furan-2-yl)benzene (Table 1, 1t, 81%**)**. [**A** (3.0 equiv.) + 1,3,5-tri-bromo-benzene (1.0 equiv.)]; The compound **1t** was prepared according to **GP1** and was purified by flash column chromatography (hexane); colorless solid; m.p. 138–140 °C; ^1H NMR (600 MHz, $CDCl_3$): δ 7.90 (s, 3H), 7.53 (d, J = 2.3 Hz, 3H), 6.78 (d, J = 3.2 Hz, 3H), 6.52 (dd, J = 3.5, 1.8 Hz, 3H); ^{13}C NMR (150 MHz, $CDCl_3$): δ 153.4, 142.3, 131.6, 118.0, 111.7, 105.7; HRMS (EI, m/z): calcd. for $C_{18}H_{12}O_3$ $[M]^+$: 276.0786; found: 276.0787.

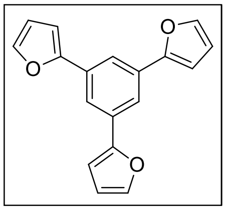

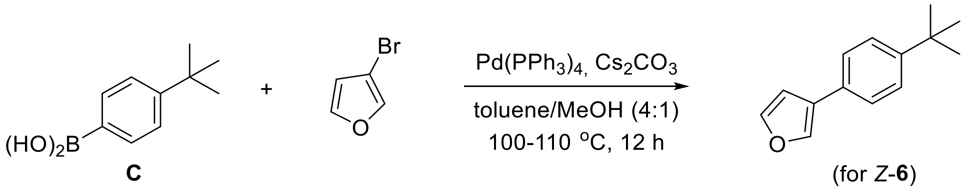

3-[4-(*tert*-Butyl)phenyl]furan (Table 2, starting material for *Z*-**6**, 68%**)**. [**C** + 3-bromofuran]; Starting material was prepared according to **GP1** and was purified by flash column chromatography (hexane); colorless solid; m.p. 59–61 °C; ^1H NMR (600 MHz, $CDCl_3$): δ 7.73 (s, 1H), 7.49 (s, 1H), 7.47–7.40 (m, 4H), 6.72 (s, 1H), 1.37 (s, 9H); ^{13}C NMR (150 MHz, $CDCl_3$): δ 150.1, 143.6, 138.4, 129.7, 126.4, 125.8, 125.8, 109.1, 34.7, 31.5; HRMS (EI, m/z): calcd. for $C_{14}H_{16}O$ $[M]^+$: 200.1201; found: 200.1199.

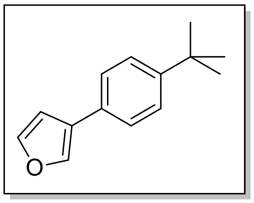

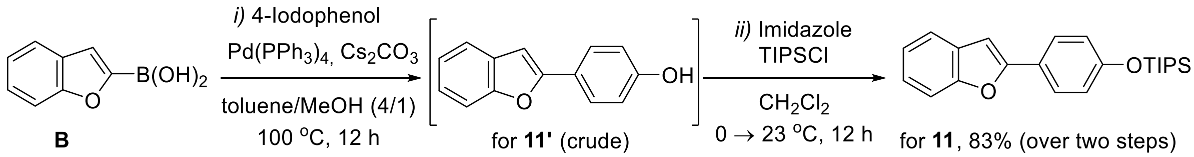

[4-(Benzofuran-2-yl)phenoxy]triisopropylsilane (Table 2, starting material for **11**, 83%, over two steps**)**. *(i) Cross-coupling reaction*: A round bottom flask was charged with 4-iodophenol (2.0 mmol, 1.34 equiv.), boronic acid (**B**, 1.0 equiv.), $Pd(PPh_3)_4$ (10 mol%), and Cs_2CO_3 (1.40 equiv.), into which toluene (16 mL) and MeOH (4 mL) were subsequently added. The reaction mixture was heated in an oil bath at 100 °C until a TLC analysis of an aliquot indicated a complete conversion of the starting materials (12 h). Then, this mixture was cooled to 23 °C and quenched with saturated NH_4Cl solution. Aqueous phase was extracted with ethyl acetate (5 mL x 3), and the combined organic phases were washed with brine (5 mL), dried over $MgSO_4$, filtered and concentrated under reduced pressure to give the corresponding starting material, and this resulting residue was subjected to *O*-silylation.

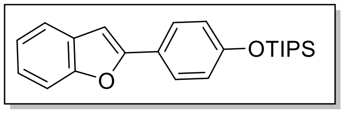

(ii) O-silylation: The crude reaction mixture was diluted with *anhydrous* CH_2Cl_2 and cooled to 0 °C, at which imidazole (1.3 equiv.) was added in one portion. This solution was briefly stirred for 10 min, into which triisopropylsilyl chloride (1.2 equiv.) in CH_2Cl_2 (2 mL) was finally added at 0 °C. Then, the reaction mixture was allowed to react at 23 °C. After 12 h, the reaction was quenched with saturated aqueous NH_4Cl solution (5 mL), and the aqueous phase was extracted with dichloromethane (5 mL x 3). The combined organic phases were washed with brine (5 mL), dried over anhydrous Na_2SO_4 and concentrated under reduced pressure. Purification of this crude reaction mixture by flash column chromatography on silica gel using cyclohexane as an eluent eventually afforded the desired product in 76% yield over 2 steps. Colorless solid; m.p. 46–48 °C; ^{1}H NMR (600 MHz, $CDCl_3$): δ 7.74 (d, J = 8.7 Hz, 2H), 7.55 (d, J = 6.9 Hz, 1H), 7.50 (d, J = 8.1 Hz, 1H), 7.33–7.17 (m, 2H), 6.96 (d, J = 8.7 Hz, 2H), 6.88 (s, 1H), 1.41–1.20 (m, 3H), 1.14 (d, J = 2.3 Hz, 9H), 1.14 (d, J = 2.3 Hz, 9H); ^{13}C NMR (150 MHz, $CDCl_3$): δ 156.9, 156.3, 154.9, 129.7, 126.5, 123.8, 123.8, 122.9, 120.7, 120.4, 111.1, 99.8, 18.1, 12.9; ^{29}Si NMR (120 MHz, $CDCl_3$): δ 16.1; HRMS (EI, m/z): calcd. for $C_{23}H_{30}O_2Si$ $[M]^+$: 366.2015; found: 366.2018.

2-Phenylbenzofuran (Table 2, starting material for **12**, 89%**)**. [**B** + bromobenzene]; The starting material was prepared according to **GP1** and was purified by flash column chromatography (hexane); colorless liquid; ^{1}H NMR (600 MHz, $CDCl_3$): δ 7.97 (d, J = 7.2 Hz, 2H), 7.68 (d, J = 8.1 Hz, 1H), 7.63 (d, J = 8.7 Hz, 1H), 7.55 (t, J = 7.7 Hz, 2H), 7.45 (t, J = 6.3 Hz, 1H), 7.38 (t, J = 7.7 Hz, 1H), 7.36–7.30 (m, 1H), 7.12 (s, 1H); ^{13}C NMR (150 MHz, $CDCl_3$): δ

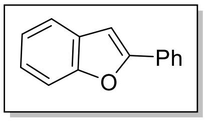

156.1, 155.1, 130.7, 129.4, 128.9, 128.7, 125.1, 124.4, 123.1, 121.0, 111.3, 101.5; HRMS (EI, m/z): calcd. for $C_{14}H_{10}O$ $[M]^+$: 194.0732; found: 194.0730.

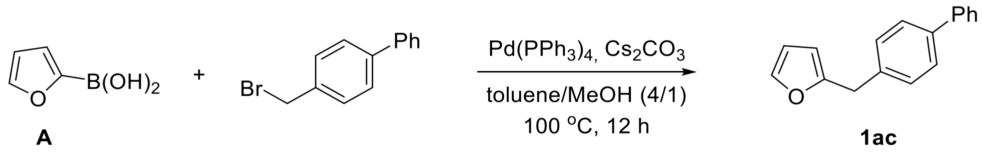

2-[(1,1'-Biphenyl)-4-ylmethyl]furan (Table 1, 1ac, 92%**)**. [**A** + 4-(bromomethyl)-1,1'-biphenyl]; The

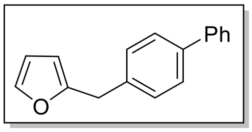

compound **1ac** was prepared according to **GP1** and was purified by flash column chromatography (hexane and ethyl acetate); m.p. 81–83 °C; ^{1}H NMR (600 MHz, $CDCl_3$) δ 7.60 (d, J = 7.8 Hz, 2H), 7.56 (d, J = 8.0 Hz, 2H), 7.45 (t, J = 7.6 Hz, 2H), 7.37 (s, 1H), 7.36–7.31 (m, 3H), 6.34 (s, 1H), 6.08 (s, 1H), 4.04 (s, 2H); ^{13}C NMR (150 MHz, $CDCl_3$) δ 154.6, 141.7, 141.1, 139.6, 137.4, 129.2, 128.9, 127.4, 127.3, 127.2, 110.4, 106.5, 34.3; HRMS (ESI, m/z): calcd. for $C_{17}H_{14}ONa$ $[M+Na]^+$: 257.0942; found: 257.0911.

Dimethylphenylsilane-*d* (Fig. 2). ^{1}H NMR (600 MHz, $CDCl_3$): δ 7.58 (d, J = 5.5 Hz, 2H), 7.40 (d, J =

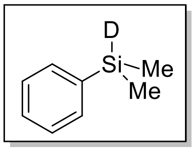

5.3 Hz, 3H), 0.38 (s, 6H); ^{13}C NMR (150 MHz, $CDCl_3$): δ 137.6, 134.1, 129.3, 128.0, -3.7; ^{29}Si NMR (120 MHz, $CDCl_3$): δ -17.5 (t, J = 28.6 Hz).

Reaction of 2-methylfuran with $PhMe_2SiH$ (2 equiv., Fig. 2B, upper part)

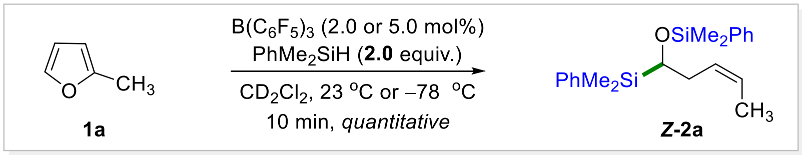

$PhMe_2SiH$ (1.0 mmol, 2.0 equiv.) was added to a solution of $B(C_6F_5)_3$ (0.01 mmol, 2.0 mol% or 0.025 mmol, 5.0 mol%) in CD_2Cl_2 (0.4 mL) in a J. Young NMR tube at 23 °C, and the solution was shaken briefly followed by the addition of the corresponding 2-methylfuran (**1a**, 0.50 mmol, 1.0 equiv.) and mesitylene (0.50 mmol, 1.0 equiv.: internal standard) under argon atmosphere. After 10 min at 23 °C, the reaction mixture was subjected to ^{1}H NMR spectroscopy. The conversion and crude NMR yields were measured on the basis of an internal standard: NMR yield > 99%, *Z*/*E* >99/1.

Reaction of 2-methylfuran with $PhMe_2SiH$ (3 equiv., Fig. 2B, lower part)

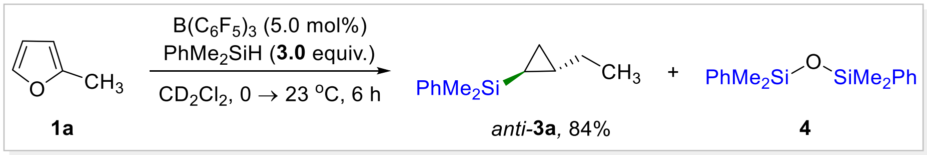

$PhMe_2SiH$ (1.5 mmol, 3.0 equiv.) was added to a solution of $B(C_6F_5)_3$ (0.025 mmol, 5.0 mol%) in CD_2Cl_2 (0.4 mL) in a J. Young NMR tube at 0 °C, and the solution was shaken briefly followed by the addition of the corresponding 2-methylfuran (**1a**, 0.50 mmol, 1.0 equiv.) and 1,1,2,2-tetrachloroethane (TCE, 1.0 equiv., 0.50 mmol: internal standard) under argon atmosphere and then the reaction mixture was stirred at 23 °C for 6 h. The reaction mixture was subjected to ^1H NMR spectroscopy. The conversion and crude NMR yields were measured on the basis of an internal standard: NMR yield = 84%, dr >99/1

^1H NMR Monitoring of the Reaction of 2-methylfuran with $PhMe_2SiH$ (4.0 equiv., Fig. 2C)

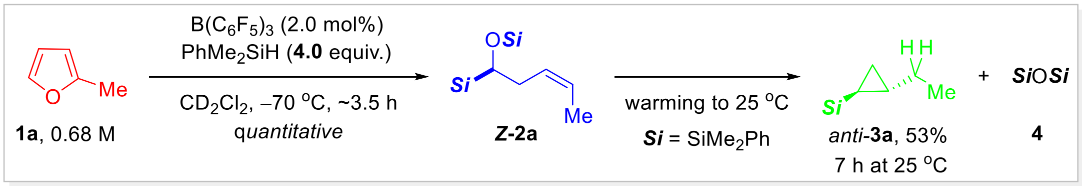

$PhMe_2SiH$ (2.0 mmol, 2.70 M, 4.0 equiv.) was added to a solution of $B(C_6F_5)_3$ (5.12 mg, 0.01 mmol, 2.0 mol%) and mesitylene (70 µL, 0.5 mmol, 1.0 equiv.) as an internal standard in CD_2Cl_2 (0.3 mL) in a J. Young NMR tube, and the solution was well shaken. The NMR tube was then placed in a bath of -78 °C to cool down the solution, then 2-methylfuran (**1a**, 0.50 mmol, 0.68 M, 1.0 equiv.) was gently added to bring a total volume of the reaction mixture to 0.740 mL. After briefly shaking, the NMR tube was quickly placed in the NMR probe pre-cooled at -70 °C, at which **1a** was smoothly converted to *Z*-**2a** over time to attain a quantitative conversion of **1a** in 3.0 h. Although prolonged time (~0.5 h) at the same temperature did not bring about any conversion of *Z*-**2a**, upon warming up to 25 °C, *Z*-**2a** smoothly underwent cyclopropanation within the borane catalytic system to give the desired silylated cyclopropane *anti*-**3a** in 53% NMR yield in 7 h. Initial rates (v_i) of conversions from the furan (**1a**, red) to *Z*-**2a** (blue), and from *Z*-**2a** (blue) to *anti*-**3a** (green) were determined, respectively from the linear portion of each "concentration *versus* time" curve in the early stage of the reaction.

Initial-Rate Kinetic Measurements (Fig. 3E): General Considerations for Kinetic Experiments: Reactions used for the kinetic analysis were set up in an Ar-filled J-Young NMR tube. Kinetic analysis of the NMR scale reaction was carried out by collecting multiple (7 ~ 20) data points early in the reaction before the substrate concentrations were depleted. Reaction progress was monitored by ^{1}H NMR (400 MHz, CD_2Cl_2) analysis at 54 sec intervals over 1 h at the specified temperature (25 ^{O}C). The kinetic data obtained from intensity increase in the terminal methyl group of cyclopropane over time (up to 20% conversion) on the basis of the internal standard of mesitylene provided the initial rate. Data were fit by least-squares analysis ($R^2 > 0.983$).

Representative Procedure for Initial-Rate Kinetics

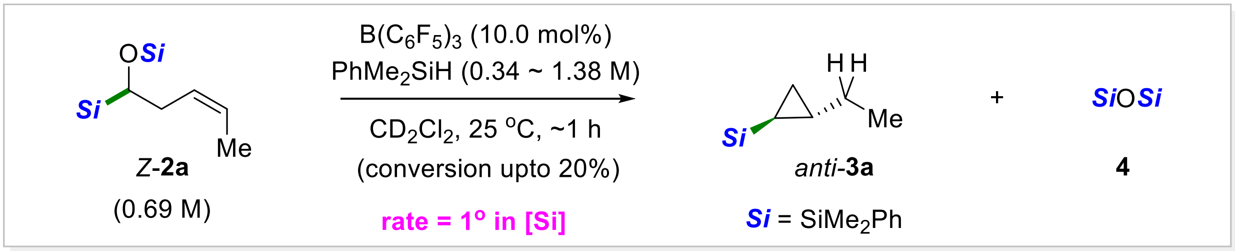

Four different reactions were carried out to determine the initial rate kinetics. $B(C_6F_5)_3$ (25.6 mg, 0.05 mmol, 10.0 mol%) was weighed into a J. Young NMR tube, dried in vacuo, and suspended in CD_2Cl_2 (0.44 ~ 0.32 mL) under Ar atmosphere, into which $PhMe_2SiH$ (0.34 ~ 1.38 M, 0.5 ~ 2.0 equiv.) and mesitylene as an internal standard (0.070 mL, 0.5 mmol, 1.0 equiv.) were added, and then the mixture was briefly shaken for 1 min at 25 ^{o}C. as a homogeneous solution formed. The NMR tube of the resulting solution was put in a pre-cooled bath at -78 ^{o}C under argon atmosphere. After 5 min, *Z*-**2a** (0.69 M, 0.50 mmol, 1.0 equiv.) was gently added into the solution containing the $B(C_6F_5)_3$ precatalyst and silane with maintaining the low temperature (-78 ^{o}C), leading to a total reaction volume for each experiments of approximately 0.726 mL. The NMR tube was well shaken to mix up all components for approximately 5 seconds, and was quickly put into the NMR probe that was pre-adjusted to the target temperature, 25 ^{o}C (298 K). Then, the ring closing of *Z*-**2a** was monitored by ^{1}H NMR at 54 seconds intervals for 1 h to determine an initial rate (v_i) for the appearance of *anti*-**3a** (up to ~20% conversion) at 25 ^{o}C.

General Procedure for the Silylative Ring-Opening Reaction (Conditions A, GP2, Table 1)

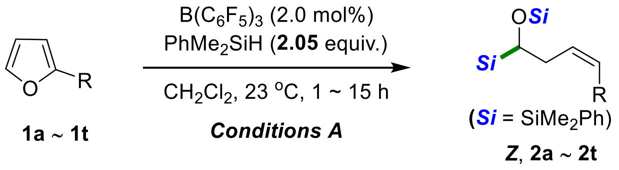

In a flame-dried flask bearing a stirring bar, $B(C_6F_5)_3$ (0.01 ~ 0.02 mmol, 2.0 mol%) was dissolved in CH_2Cl_2 (0.4 ~ 0.8 mL). Silane (1.025 ~ 2.050 mmol) was added, and the solution was shaken shortly to make it homogeneous. The corresponding furan derivative (**1a** ~ **1t**, 0.50 ~ 1.0 mmol) was then added and the reaction mixture was stirred at 23 °C for the indicated time (1 ~ 15 h). After quenching the reaction mixture with Et_3N (10.0 ~ 20.0 mol%), the crude reaction mixture was concentrated under reduced pressure and then purified by flash column chromatography on silica gel (using either hexane only or a mixture of hexane/ethyl acetate) to afford the desired products (*Z*-**2a** ~ *Z*-**2t**, in all cases *Z*/*E* >99/1).

Characterization Data of the Obtained (*Z*)-α-Silyloxyalkenylsilanes [Table 1, *Z*-2a ~ *Z*-2t]

(*Z*)-[1-(Dimethylphenylsilyloxy)pent-3-en-1-yl]dimethylphenylsilane [Table 1, *Z*-**2a**, Reaction time: 1 h, 95% (1.0 mmol scale)]. Employed 2-methylfuran (**1a**, 82.2 mg, 1 mmol); $B(C_6F_5)_3$ (1.0 mol%) and dimethylphenylsilane (2.05 mmol) were used at 23 °C for 1 h (*Z*-**2a**, 95%). The compound *Z*-**2a** was prepared according to **GP2** and was purified by flash column chromatography (hexane and ethyl acetate).

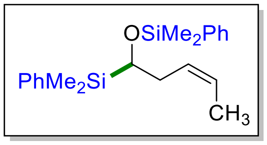

Gram-scale reaction: Employed 2-methylfuran (**1a**, 3.284 g, 40 mmol); dimethylphenylsilane (11.18 g, 82 mmol), $B(C_6F_5)_3$ (10.2 mg, 0.05 mol%), and CH_2Cl_2 (8 mL) were used at 23 °C for 12 h under argon atmosphere to show >95% conversion determined by a ^{1}H NMR analysis. *Z*-**2a** was isolated with 91% (12.88 g) yield as colorless liquid; ^{1}H NMR (600 MHz, $CDCl_3$): δ 7.66–7.57 (m, 4H), 7.50–7.38 (m, 6H), 5.52–5.40 (m, 2H), 3.75 (t, J = 6.9 Hz, 1H), 2.44–2.30 (m, 2H), 1.56 (dd, J = 6.6, 1.6 Hz, 3H), 0.39 (s, 6H), 0.36 (s, 3H), 0.35 (s, 3H); ^{13}C NMR (100 MHz, $CDCl_3$): δ 138.8, 137.8, 134.4, 133.8, 129.4, 129.1, 128.4, 127.7 (2C), 125.1, 66.9, 32.0, 13.0, -0.8, -0.9, -4.5, -4.7; ^{29}Si NMR (120 MHz, $CDCl_3$): δ 6.0, -4.2; HRMS (EI, m/z): calcd. for $C_{21}H_{30}OSi_2$ $[M]^+$: 354.1835; found: 354.1831.

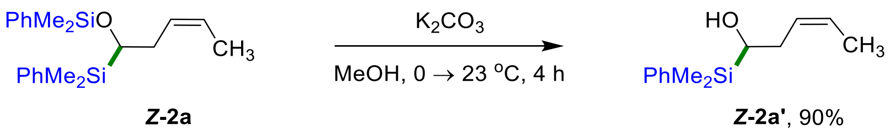

(*Z*)-1-(Dimethylphenylsilyl)pent-3-en-1-ol (Fig. 4, *Z*-2a', Reaction time: 4 h, 90%). Employed *Z*-**2a** (3.54 g, 10.0 mmol), K_2CO_3 (2.76 g, 20 mmol), and MeOH (40 mL); colorless liquid; ^{1}H NMR (600 MHz, $CDCl_3$): δ 7.65–7.55 (m, 2H), 7.43–7.34 (m, 3H), 5.75–5.57 (m, 1H), 5.49–5.31 (m, 1H), 3.49 (dd, J = 12, 6.0 Hz, 1H), 2.49–2.33 (m, 1H), 2.26–2.07 (m, 1H), 1.61 (d, J = 6.7 Hz, 3H), 1.53–1.33 (m, 1H), 0.39 (s, 3H), 0.37 (s, 3H); ^{13}C NMR (150 MHz, $CDCl_3$): δ 136.9, 134.2, 129.4, 127.9, 127.6, 127.5, 64.6, 31.1, 13.1, -5.3, -5.3; ^{29}Si NMR (120 MHz, $CDCl_3$): δ -3.8; HRMS (EI, m/z): calcd. for $C_{13}H_{20}OSi$ $[M]^+$: 220.1283; found: 220.1279.

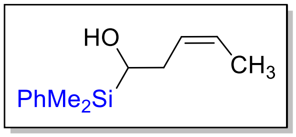

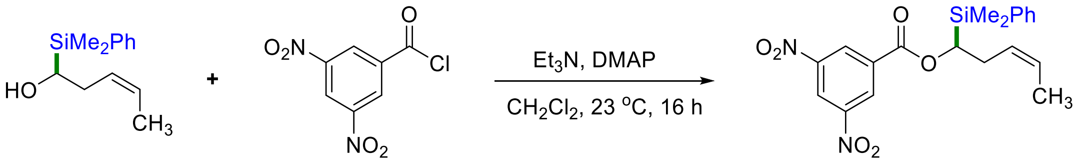

***Z*-2a'** ***Z*-2a''**, 83%

(*Z*)-1-[(Dimethylphenylsilyl)(pent-3-en-1-yl)]-3,5-dinitrobenzoate (Table 1, *Z*-2a'', Reaction time: 16 h, 83%). A flame-dried Schlenk flask was charged with *Z*-**2a'** (1.00 g, 4.54 mmol) in CH_2Cl_2 (40 mL). At 0 °C, triethylamine (Et_3N, 2.3 g, 22.7 mmol), dimethylaminopyridine (DMAP, 0.910 mmol, 111 mg, 20.0 mol%), and 3,5-dinitrobenzoyl chloride (1.36 g, 5.9 mmol) were slowly added. The resultant solution was allowed to warm to 23 °C and stirred for 16 h. The reaction mixture was then diluted with CH_2Cl_2 and quenched with water (5 mL). The aqueous phase was extracted with CH_2Cl_2, and the combined organic layers were washed with water and brine, followed by dried over Mg_2SO_4. Evaporation of the solvents under reduced pressure afforded the crude product, which was purified by flash column chromatography on silica gel (ethyl acetate/hexane, 1/10) to give *Z*-**2a''** (1.553 g, 83%). X-ray quality crystals of *Z*-**2a''** were grown by slow evaporation from CH_2Cl_2 and *n*-pentane as yellow crystals. m.p. 79–81 °C; ^{1}H NMR (600 MHz, $CDCl_3$): δ 9.19 (s, 1H), 9.02 (s, 2H), 7.57 (s, 2H), 7.38 (s, 3H), 5.53–5.44 (m, 1H), 5.37–5.32 (m, 1H), 5.31–5.26 (m, 1H), 2.71–2.55 (m, 1H), 2.46–2.28 (m, 1H), 1.54 (d, J = 6.6 Hz, 3H), 0.45 (s, 6H); ^{13}C NMR (100 MHz, $CDCl_3$): δ 162.7, 148.8, 135.0, 134.5, 134.1, 130.0, 129.3, 128.2, 126.9, 126.3, 122.2, 71.6, 28.8, 13.0, -4.7, -4.8; ^{29}Si NMR (120 MHz, $CDCl_3$): δ -2.6; HRMS (EI, m/z): calcd. for $C_{20}H_{22}N_2O_6Si$ $[M]^+$: 414.1247; found: 414.1244.

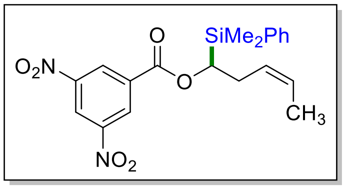

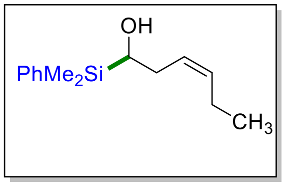

(*Z*)-1-(Dimethylphenylsilyl)hex-3-en-1-ol (Table 1, *Z*-2b, Reaction time: 4.5 h for two steps, 86%). (*i*) Employed 2-ethylfuran (**1b**, 96 mg, 1.0 mmol), dimethylphenylsilane (279 mg, 2.1 mmol), $B(C_6F_5)_3$ (10.2 mg, 2.0 mol%), and CH_2Cl_2 (0.8 mL). The reaction mixture was stirred at 23 °C for 1.5 h under

argon atmosphere. (*ii*) After completion, the reaction mixture was treated with K_2CO_3 (276 mg, 2.00 mmol) in MeOH (2 mL) at 23 °C for 3 h under air to afford *Z*-**2b** (201.5 mg, 86% for two steps) as colorless liquid; ^{1}H NMR (600 MHz, $CDCl_3$): δ 7.70–7.57 (m, 2H), 7.48–7.30 (m, 3H), 5.97–5.57 (m, 1H), 5.45–5.10 (m, 1H), 3.58–3.42 (m, 1H), 2.51–2.32 (m, 1H), 2.28–2.17 (m, 1H), 2.07 (d, J = 6.7 Hz, 2H), 1.56 (brs, 1H), 0.99 (t, J = 6.7 Hz, 3H), 0.42 (s, 3H), 0.41 (s, 3H); ^{13}C NMR (150 MHz, $CDCl_3$): δ 136.9, 135.3, 134.2, 129.3, 127.9, 125.9, 64.5, 31.4, 20.7, 14.4, -5.3, -5.5; ^{29}Si NMR (120 MHz, $CDCl_3$): δ -3.8; HRMS (EI, m/z): calcd. for $C_{14}H_{22}OSi$ $[M]^+$: 234.1440; found 234.1440.

(*Z*)-[1-(Dimethylphenylsilyloxy)hept-3-en-1-yl]dimethylphenylsilane (Table 1, *Z*-**2c**, Reaction time: 2 h, 92%**)**. Employed 2-propylfuran (**1c**); The compound *Z*-**2c** was prepared according to **GP2** and was purified by flash column chromatography (hexane and ethyl acetate);.colorless liquid; ^{1}H NMR (600 MHz, $CDCl_3$): δ 7.60–7.51 (m, 4H), 7.32–7.42 (m, 6H), 5.56–5.17 (m, 2H), 3.66 (t, J = 6.9 Hz, 1H), 2.67–2.23 (m, 2H), 1.87 (q, J = 6.6 Hz, 2H), 1.37–1.26 (m, 2H), 0.87 (t, J = 7.4 Hz, 3H), 0.32 (s, 6H), 0.29 (s, 3H), 0.28 (s, 3H); ^{13}C NMR (150 MHz, $CDCl_3$): δ 138.8, 137.8, 134.4, 133.8, 131.0, 129.4, 129.1, 127.7 (2C), 127.5, 67.1, 32.4, 29.5, 22.9, 13.9, -0.7, -0.9, -4.5, -4.7; ^{29}Si NMR (120 MHz, $CDCl_3$):δ 5.9, -4.3; HRMS (EI, m/z): calcd. for $C_{23}H_{34}OSi_2$ $[M]^+$: 382.2148; found: 382.2144.

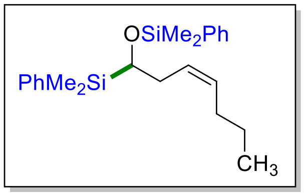

(*Z*)-1-[(Dimethylphenylsilyloxy)-non-3-en-1-yl]dimethylphenylsilane (Table 1, *Z*-**2d**, Reaction time: 1 h, 86%**)**. Employed 2-pentylfuran (**1d**); The compound *Z*-**2d** was prepared according to **GP2** and was purified by flash column chromatography (hexane and ethyl acetate);.colorless liquid; ^{1}H NMR (600 MHz, $CDCl_3$): δ 7.57–7.48 (m, 4H), 7.42–7.31 (m, 6H), 5.41–5.12 (m, 2H), 3.65 (t, J = 6.9 Hz, 1H), 2.34–2.23 (m, 2H), 1.94–1.82 (m, 2H), 1.33–1.26 (m, 4H), 1.26–1.17 (m, 2H), 0.90 (t, J = 7.1 Hz, 3H), 0.31 (s, 6H), 0.28 (s, 3H), 0.27 (s, 3H); ^{13}C NMR (150 MHz, $CDCl_3$): δ 138.8, 137.8, 134.4, 133.79, 131.3, 129.4, 129.1, 127.7 (2C), 127.3, 67.1, 32.4, 31.70, 29.4, 27.5, 22.7, 14.2, -0.7, -0.9, -4.5, -4.7; ^{29}Si NMR (120 MHz, $CDCl_3$): δ 5.9, -4.3; HRMS (ESI, m/z): calcd. for $C_{25}H_{38}ONaSi_2$ $[M+Na]^+$: 433.2359; found: 433.2368.

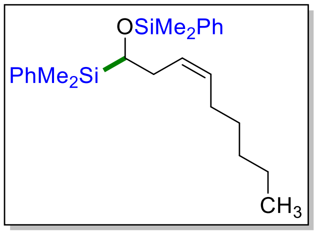

(*Z*)-1-[(Dimethylphenylsilyloxy)-4-(4-trifluoromethylphenyl)but-3-en-1-yl]dimethylphenylsilane (Table 1, *Z*-**2e**, Reaction time: 4 h, 90%**)**. Employed 2-[4-(trifluoromethyl) phenyl]furan (**1e**); The compound *Z*-**2e** was prepared according to **GP2** and was purified by flash column chromatography (hexane and ethyl acetate);.colorless liquid; ^{1}H NMR (600 MHz, $CDCl_3$): δ 7.62–7.57 (m, 1H), 7.57–7.52 (m, 2H), 7.52–7.46 (m, 3H), 7.41–7.34 (m, 5H), 7.33–7.27 (m, 3H), 6.39 (d, J = 11.7 Hz,

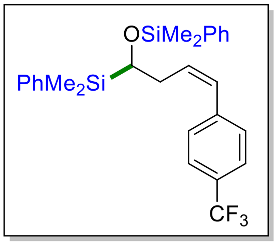

1H), 5.75 (dt, J = 11.7, 7.4 Hz, 1H), 3.77 (dd, J = 8.0, 5.2 Hz, 1H), 2.65–2.55 (m, 1H), 2.50–2.41 (m, 1H), 0.38 (s, 3H), 0.31 (s, 6H), 0.30 (s, 3H); ^{13}C NMR (150 MHz, $CDCl_3$): δ 141.1, 138.4, 137.2, 134.3, 133.8, 133.1, 129.6, 129.3, 129.0, 128.9, 128.5 (q, J = 33.2 Hz), 127.8, 126.2, 125.1 (q, J = 4.5 Hz), 124.5 (q, J = 273 Hz), 66.8, 33.2, -0.8, -0.9, -4.7, -4.9; ^{29}Si NMR (120 MHz, $CDCl_3$): δ 6.6, -4.0; ^{19}F NMR (565 MHz, $CDCl_3$) δ -114.4; HRMS (ESI, m/z): calcd. for $C_{27}H_{31}F_3NaOSi_2$ $[M+Na]^+$: 507.1763; found: 507.1771.

(*Z*)-1-[(Dimethylphenylsilyloxy)-4-(4-fluorophenyl)but-3-en-1-yl]dimethylphenylsilane (Table 1, *Z*-**2f**, Reaction time: 1.5 h, 85%**)**. Employed 2-(4-fluorophenyl)furan (**1f**); The compound *Z*-**2f** was prepared according to **GP2** and was purified by flash column chromatography (hexane and ethyl acetate);.colorless liquid; ^{1}H NMR (600 MHz, $CDCl_3$): δ 7.53 (d, J = 6.7 Hz, 2H), 7.47 (d, J = 7.2 Hz, 2H), 7.41–7.30 (m, 6H), 7.18–7.12 (m, 2H), 6.99–6.90 (m, 2H), 6.33 (d, J = 11.6 Hz, 1H), 5.62 (dt, J = 11.3, 7.3 Hz, 1H), 3.74 (dd, J = 7.9, 5.5 Hz, 1H), 2.58 (dt, J = 15.6, 8.0 Hz, 1H), 2.45 (dt, J = 15.1, 6.0 Hz, 1H), 0.30 (s, 3H), 0.29 (s, 6H), 0.28 (s, 3H); ^{13}C NMR (150 MHz, $CDCl_3$): δ 161.6 (d, J = 246.1 Hz), 138.5, 137.4, 134.4, 133.8, 133.6 (d, J = 3.0 Hz), 133.1, 130.7, 130.4 (d, J = 7.6 Hz), 129.5, 129.2, 128.9, 127.8, 115.0 (d, J = 21.1 Hz), 66.9, 33.1, -0.8, -0.9, -4.7, -4.9; ^{29}Si NMR (120 MHz, $CDCl_3$): δ 6.5, -4.1; ^{19}F NMR (375 MHz, CD_2Cl_2): δ -115.9; HRMS (EI, m/z): calcd. for $C_{26}H_{31}FOSi_2$ $[M]^+$: 434.1897; found: 434.1901.

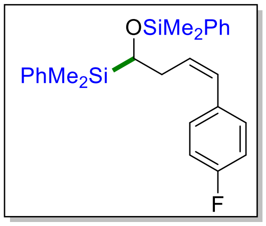

(*Z*)-1-[(Dimethylphenylsilyloxy)-4-(4-chlorophenyl)but-3-en-1-yl]dimethylphenylsilane (Table 1, *Z*-**2g**, Reaction time: 2 h, 86%**)**. Employed 2-(4-chlorophenyl)furan (**1g**); The compound *Z*-**2g** was prepared according to **GP2** and was purified by flash column chromatography (hexane and ethyl acetate);.colorless liquid; ^{1}H NMR (600 MHz, $CDCl_3$): δ 7.56–7.52 (m, 2H), 7.47 (d, J = 7.2 Hz, 2H), 7.42–7.30 (m, 6H), 7.23 (d, J = 8.1 Hz, 2H), 7.12 (d, J = 8.2 Hz, 2H), 6.32 (d, J = 11.6 Hz, 1H), 5.65 (dt, J = 11.7, 7.5 Hz, 1H), 3.75 (dd, J = 8.0, 5.5 Hz, 1H), 2.69–2.53 (m, 1H), 2.48–2.29 (m, 1H), 0.30 (s, 9H), 0.29 (s, 3H); ^{13}C NMR (150 MHz, $CDCl_3$): δ 138.5, 137.3, 136.0, 134.4, 133.8, 132.3, 131.5, 130.1, 129.5, 129.3, 128.9, 128.3, 127.80, 127.78, 66.8, 33.2, -0.8, -0.9, -4.7, -4.9; ^{29}Si NMR (120 MHz, $CDCl_3$): δ 6.5, -4.1; HRMS (EI, m/z): calcd. for $C_{26}H_{31}ClOSi_2$ $[M]^+$: 450.1602; found: 450.1600.

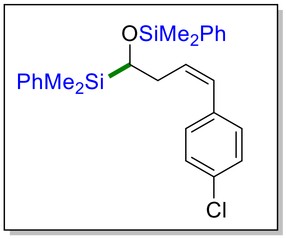

(*Z*)-1-[(Dimethylphenylsilyloxy)-4-(4-bromophenyl)but-3-en-1-yl]dimethylphenylsilane (Table 1, *Z*-2h, Reaction time: 2 h, 91%**)**. Employed 2-(4-bromophenyl)furan (**1h**); The compound *Z*-**2h** was prepared according to **GP2** and was purified by flash column chromatography (hexane and ethyl acetate);.colorless liquid; ^{1}H NMR (600 MHz, $CDCl_3$): δ 7.53 (d, J = 9.2 Hz, 2H), 7.47 (d, J = 7.9 Hz, 2H), 7.42–7.30 (m, 8H), 7.06 (d, J = 8.3 Hz, 2H), 6.30 (d, J = 11.7 Hz, 1H), 5.89–5.56 (m, 1H), 3.79–3.70 (m, 1H), 2.58 (dd, J = 15.7, 7.5 Hz, 1H), 2.51–2.36 (m, 1H), 0.30 (s, 6H), 0.30 (s, 3H), 0.29 (s, 3H); ^{13}C NMR (150 MHz, $CDCl_3$): δ 138.5, 137.3, 136.4, 134.3, 133.8, 131.6, 131.2, 130.4, 129.5, 129.3, 128.9, 127.80, 127.78, 120.4, 66.8, 33.1, -0.8, -0.9, -4.7, -4.9; ^{29}Si NMR (120 MHz, $CDCl_3$): δ 6.5, -4.1; HRMS (EI, m/z): calcd. for $C_{26}H_{31}BrOSi_2$ $[M]^+$: 494.1097; found: 494.1092.

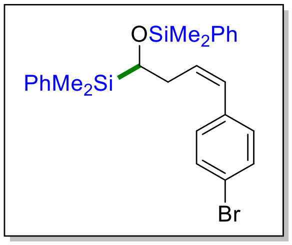

(*Z*)-{4-[(1,1'-Biphenyl)-4-yl]-1-(dimethylphenylsilyloxy)but-3-en-1-yl}dimethylphenylsilane (Table 1, *Z*-2**i**, Reaction time: 3 h, 92%**)**. Employed 2-(1,1'-biphenyl-4-yl)furan (**1i**); The compound *Z*-**2i** was prepared according to **GP2** and was purified by flash column chromatography (hexane and ethyl acetate);.colorless liquid; ^{1}H NMR (400 MHz, $CDCl_3$): δ 7.86–7.79 (m, 2H), 7.79–7.74 (m, 2H), 7.73–7.67 (m, 4H), 7.64 (t, J = 7.6 Hz, 2H), 7.58–7.46 (m, 9H), 6.63 (d, J = 11.7 Hz, 1H), 5.95–5.81 (m, 1H), 4.00 (dd, J = 7.8, 5.6 Hz, 1H), 2.98–2.84 (m, 1H), 2.84–2.70 (m, 1H), 0.53 (s, 3H), 0.52 (s, 6H), 0.51 (s, 3H); ^{13}C NMR (100 MHz, $CDCl_3$): δ 141.1, 139.36, 138.6, 137.4, 136.7, 134.5, 133.9, 131.1, 129.7, 129.6, 129.4, 129.3, 128.9, 127.9 (2C), 127.4, 127.1, 126.9, 67.1, 33.4, -0.6, -0.7, -4.6, -4.7; ^{29}Si NMR (80 MHz, $CDCl_3$): δ 6.6, -3.9; HRMS (ESI, m/z): calcd. for $C_{32}H_{36}NaOSi_2$ $[M+Na]^+$: 515.2202; found: 515.2200.

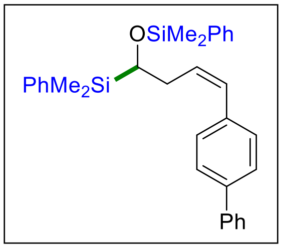

(*Z*)-1-[(Dimethylphenylsilyloxy)-4-phenylbut-3-en-1-yl]dimethylphenylsilane (Table 1, ***Z*-2j**, Reaction time: 2 h, 91%**)**. Employed 2-phenylfuran (**1j**) at 0 °C to 23 °C; The compound *Z*-**2j** was prepared according to **GP2** and was purified by flash column chromatography (hexane and ethyl acetate);.colorless liquid; ^{1}H NMR (600 MHz, $CDCl_3$): δ 7.59–7.55 (m, 2H), 7.53–7.50 (m, 2H), 7.43–7.48 (m, 4H), 7.37–7.29 (m, 4H), 7.28–7.21 (m, 3H), 6.43 (d, J = 11.7 Hz, 1H), 5.78–5.56 (m, 1H), 3.93–3.53 (m, 1H), 2.66 (t, J = 7.1 Hz, 1H), 2.61–2.39 (m, 1H), 0.34 (s, 3H), 0.33 (s, 6H), 0.32 (s, 3H); ^{13}C NMR (150 MHz, $CDCl_3$): δ 138.6, 137.6, 137.5, 134.4, 133.8, 133.1, 130.8, 130.1, 129.5, 129.2, 128.9, 128.2, 127.8, 126. 6, 67.0, 33.3, -0.8, -0.8, -4.8, -4.8; ^{29}Si NMR (120 MHz, $CDCl_3$): δ 6.4, -4.2; HRMS (ESI, m/z): calcd. for $C_{26}H_{32}NaOSi_2$ $[M+Na]^+$: 439.1889; found: 439.1888.

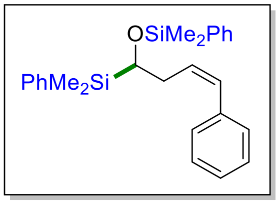

(*Z*)-[1-(Dimethylphenylsilyloxy)-4-(*m*-tolyl)but-3-en-1-yl]dimethylphenylsilane (Table 1, *Z*-2k,

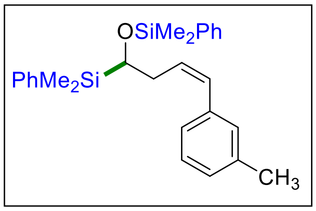

Reaction time: 2 h, 83%). Employed 2-(*m*-tolyl)furan (**1k**); The compound *Z*-**2k** was prepared according to **GP2** and was purified by flash column chromatography (hexane and ethyl acetate);.colorless liquid; ^{1}H NMR (600 MHz, $CDCl_3$): δ 7.59–7.46 (m, 4H), 7.47–7.29 (m, 6H), 7.19 (t, J = 7.6 Hz, 1H), 7.14–7.00 (m, 3H), 6.39 (d, J = 11.6 Hz, 1H), 5.75–5.54 (m, 1H), 3.78 (t, J = 6.8 Hz, 1H), 2.73–2.59 (m, 1H), 2.59–2.47 (m, 1H), 2.35 (s, 3H), 0.31 (s, 12H); ^{13}C NMR (150 MHz, $CDCl_3$): δ 138.5, 137.5, 137.4, 137.3, 134.3, 133.6, 130.5, 130.0, 129.5, 129.3, 129.0, 127.9, 127.6 (2C), 127.2, 125.8, 66.9, 33.1, 21.4, -0.9 (2C), -4.8, -4.9; ^{29}Si NMR (120 MHz, $CDCl_3$): δ = 6.3, -4.1; HRMS (ESI, m/z): calcd. for $C_{27}H_{34}NaOSi_2$ $[M+Na]^+$: 453.2046; found: 453.2040.

(*Z*)-1-[(Dimethylphenylsilyloxy)-4-(4-phenoxyphenyl)-but-3-en-1-yl]dimethylphenylsilane (Table 1,

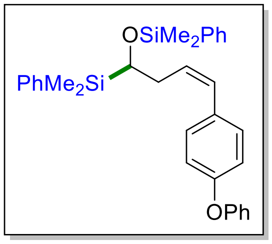

***Z*-2l, Reaction time: 2 h, 70%).** Employed 2-(4-phenoxyphenyl)furan (**1l**); The compound *Z*-**2l** was prepared according to **GP2** and was purified by flash column chromatography (hexane and ethyl acetate);.colorless liquid; ^{1}H NMR (600 MHz, $CDCl_3$): δ 7.62 (d, J = 6.4 Hz, 2H), 7.57 (d, J = 6.4 Hz, 2H), 7.48–7.37 (m, 8H), 7.25 (d, J = 8.3 Hz, 2H), 7.20 (t, J = 7.4 Hz, 1H), 7.12 (d, J = 8.5 Hz, 2H), 7.00 (d, J = 8.4 Hz, 2H), 6.44 (d, J = 11.5 Hz, 1H), 5.71 (dt, J = 11.5, 7.1 Hz, 1H), 3.85 (t, J = 6.7 Hz, 1H), 2.76–2.67 (m, 1H), 2.65–2.53 (m, 1H), 0.38 (s, 12H); ^{13}C NMR (150 MHz, $CDCl_3$): δ 157.4, 155.8, 138.5, 137.4, 134.4, 133.8, 132.8, 130.3, 130.1, 129.8, 129.5, 129.3, 129.2, 127.8 (2C), 123.3, 118.9, 118.6, 67.0, 33.2, -0.7, -0.8, -4.7, -4.8; ^{29}Si NMR (120 MHz, $CDCl_3$): δ 6.5, -4.1; HRMS (EI, m/z): calcd. for $C_{32}H_{36}O_2Si_2$ $[M]^+$: 508.2254; found: 508.2256.

(*Z*)-1-[(Dimethylphenylsilyloxy)-4-(4-methylthiophenyl)-but-3-en-1-yl]dimethylphenylsilane (Table

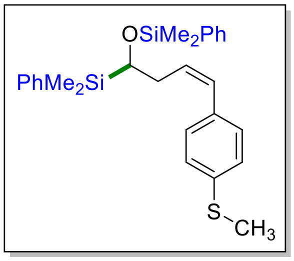

1, *Z*-2m, Reaction time: 1.5 h, 91%). Employed **1m**; The compound *Z*-**2m** was prepared according to **GP2** and was purified by flash column chromatography (hexane and ethyl acetate);.colorless liquid; ^{1}H NMR (600 MHz, $CDCl_3$): δ 7.51 (d, J = 7.6 Hz, 2H), 7.46 (d, J = 7.0 Hz, 2H), 7.40–7.27 (m, 6H), 7.19–7.09 (m, 4H), 6.30 (d, J = 11.6 Hz, 1H), 5.59 (dt, J = 12.1, 7.5 Hz, 1H), 3.73 (t, J = 6.6 Hz, 1H), 2.73–2.55 (m, 1H), 2.49 (s, 3H), 2.48–2.43 (m, 1H), 0.27 (s, 12 H); ^{13}C NMR (150 MHz, $CDCl_3$): δ 138.6, 137.4, 136.4, 134.6, 134.4, 133.8, 130.7, 129.5, 129.4, 129.3, 129.2, 127.8 (2C), 126.5, 66.9, 33.3, 16.1, -0.8, -0.9, -4.8, -4.8; ^{29}Si NMR (120 MHz, $CDCl_3$): δ 6.4, -4.1; HRMS (EI, m/z): calcd. for $C_{27}H_{34}OSSi_2$ $[M]^+$: 462.1869; found: 462.1871.

(*Z*)-{1-[(Dimethylphenylsilyl)-4-(dimethylphenylsilyloxy)-but-1-en-1-yl]phenoxy}triisopropylsilane (Table 1, *Z*-2n, Reaction time: 3 h, 95%**)**. Employed **1n**; The compound *Z*-**2n** was prepared according to

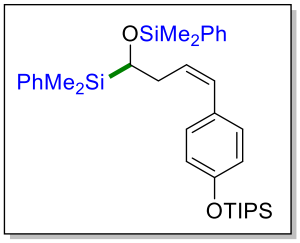

GP2 and was purified by flash column chromatography (hexane and ethyl acetate);.colorless liquid; ^{1}H NMR (600 MHz, $CDCl_3$): δ 7.54 (d, J = 6.5 Hz, 2H), 7.50 (d, J = 6.5 Hz, 2H), 7.41–7.30 (m, 6H), 7.08 (d, J = 8.4 Hz, 2H), 6.80 (d, J = 8.5 Hz, 2H), 6.32 (d, J = 11.6 Hz, 1H), 5.61–5.45 (m, 1H), 3.79–3.73 (m, 1H), 2.66–2.51 (m, 2H), 1.36–1.25 (m, 3H), 1.15 (d, J = 7.4 Hz, 18H), 0.29 (s, 12H); ^{13}C NMR (150 MHz, $CDCl_3$): δ 154.8, 138.7, 137.6, 134.4, 133.8, 130.6, 130.0, 129.6, 129.4, 129.2, 128.9, 127.7 (2C), 119.6, 67.1, 33.3, 18.1, 12.9, -0.7, -0.8, -4.8, -4.8; ^{29}Si NMR (120 MHz, $CDCl_3$): δ 15.2, 6.3, -4.2; HRMS (EI, m/z): calcd. for $C_{35}H_{52}O_2Si_3$ $[M]^+$: 588.3275; found: 588.3279.

(*Z*)-[4-(3,5-Dibromophenyl)-1-(dimethylphenylsilyloxy)but-3-en-1-yl]dimethylphenylsilane (Table 1,

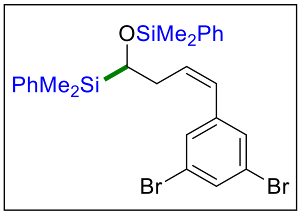

Z-**2o**, Reaction time: 15 h, 79%**)**. Employed 2-(3,5-dibromophenyl)furan (**1o**); The compound *Z*-**2o** was prepared according to **GP2** and was purified by flash column chromatography (hexane and ethyl acetate);. colorless liquid; ^{1}H NMR (600 MHz, $CDCl_3$): δ 7.54–7.49 (m, 3H), 7.49–7.44 (m, 2H), 7.43–7.28 (m, 8H), 6.21 (d, J = 11.7 Hz, 1H), 5.72–5.67 (m, 1H), 3.75 (dd, J = 8.6, 4.9 Hz, 1H), 2.62–2.52 (m, 1H), 2.37–2.27 (m, 1H), 0.29 (s, 6H), 0.28 (s, 6H); ^{13}C NMR (150 MHz, $CDCl_3$): δ 140.8, 138.1, 136.9, 134.1, 133.6, 133.6, 131.8, 130.3, 129.4, 129.2, 127.7, 127.6, 127.5, 122.5, 66.4, 32.8, -1.0 (2C), -4.9, -5.0; ^{29}Si NMR (120 MHz, $CDCl_3$): δ 6.7, -3.9; HRMS (ESI, m/z): calcd. for $C_{26}H_{30}Br_2NaOSi_2$ $[M+Na]^+$: 595.0100; found: 595.0095.

(*Z*)-1-[(Dimethylphenylsilyloxy)-4-(2,4,6-triisopropylphenyl)but-3-en-1-yl]dimethylPhenylsilane

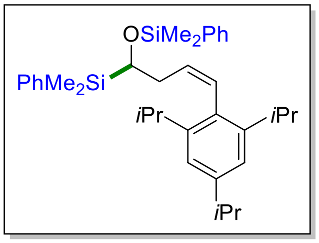

(Table 1, *Z*-**2p**, Reaction time: 6 h, 80%**)**. Employed 2-(2,4,6-triisopropylphenyl)furan (**1p**); The compound *Z*-**2p** was prepared according to **GP2** and was purified by flash column chromatography (hexane and ethyl acetate);.colorless liquid; ^{1}H NMR (600 MHz, $CDCl_3$): δ 7.51–7.46 (m, 2H), 7.46–7.41 (m, 2H), 7.40–7.31 (m, 4H), 7.29 (t, J = 7.3 Hz, 2H), 7.05–6.90 (m, 2H), 6.28 (d, J = 11.6 Hz, 1H), 5.81–5.71 (m, 1H), 3.57 (dd, J = 8.9, 5.6 Hz, 1H), 3.12–2.98 (m, 2H), 2.95–2.85 (m, 1H), 2.17–2.09 (m, 1H), 2.08–1.90 (m, 1H), 1.29 (d, J = 6.8 Hz, 6H), 1.24–1.13 (m, 4H), 1.13–1.07 (m, 8H), 0.23 (s, 3H), 0.21 (s, 3H), 0.19 (s, 6H); ^{13}C NMR (150 MHz, $CDCl_3$): δ 147.4, 138.7, 137.5, 134.3, 133.8, 131.6, 131.1, 129.4, 129.1, 128.2, 127.7, 127.7, 120.3, 120.3, 66.6, 34.4, 33.5, 30.2, 24.5, 24.3, 23.2, -0.9, -1.1, -4.8, -5.0; ^{29}Si NMR (120 MHz, $CDCl_3$): δ 5.9, -4.5; HRMS (EI, m/z): calcd. for $C_{35}H_{50}OSi_2$ $[M]^+$: 542.3400; found: 542.3396.

(*Z*)-1-[(Dimethylphenylsilyloxy)-4-(phenanthren-9-yl)but-3-en-1-yl] dimethylphenylsilane (Table 1, *Z*-2q, Reaction time: 4 h, 71%). Employed 2-(phenanthren-9-yl)furan (**1q**); The compound *Z*-**2q** was prepared according to **GP2** and was purified by flash column chromatography (hexane and ethyl acetate);.colorless liquid; ^{1}H NMR (600 MHz, $CDCl_3$): δ 8.82 (d, J = 8.2 Hz, 1H), 8.77 (d, J = 8.2 Hz, 1H), 8.11 (d, J = 8.1 Hz, 1H), 7.84 (d, J = 7.8 Hz, 1H), 7.80–7.66 (m, 5H), 7.64–7.60 (m, 2H), 7.52–7.44 (m, 3H), 7.44–7.38 (m, 2H), 7.37–7.32 (m, 1H), 7.29–7.24 (m, 2H), 6.97 (d, J = 11.4 Hz, 1H), 6.08 (dt, J = 11.5, 6.9 Hz, 1H), 3.87 (dd, J = 8.0, 5.6 Hz, 1H), 2.90–2.64 (m, 1H), 2.60–2.29 (m, 1H), 0.39 (s, 3H), 0.37 (s, 3H), 0.34 (s, 3H), 0.31 (s, 3H); ^{13}C NMR (150 MHz, $CDCl_3$): δ 138.5, 137.4, 134.3, 133.8, 132.8, 132.7, 131.7, 131.4, 130.5, 130.0, 129.5, 129.1, 128.7, 128.3, 127.8, 127.7, 127.2, 126.7, 126.6, 126.5, 126.4, 125.7, 123.0, 122.5, 67.0, 33.5, -0.8, -0.9, -4.7, -4.8; ^{29}Si NMR (120 MHz, $CDCl_3$): δ 6.4, -4.3; HRMS (EI, m/z): calcd. for $C_{34}H_{36}OSi_2$ $[M]^+$: 516.2305; found: 516.2307.

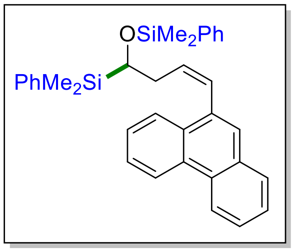

(*Z*)-1,4-Bis[4-(dimethylphenylsilyl)-4-(dimethylphenylsilyloxy)but-1-en-1-yl]benzene (Table 1, *Z*-2r, Reaction time: 2 h, 72%). Employed 1,4-di(furan-2-yl)benzene (**1r**) and dimethylphenylsilane (4.0 equiv.); The compound *Z*-**2r** was prepared according to **GP2** and was purified by flash column chromatography (hexane and ethyl acetate);.colorless liquid; ^{1}H NMR (600 MHz, $CDCl_3$): δ 7.54 (d, J = 6.4 Hz, 4H), 7.49 (d, J = 7.8 Hz, 4H), 7.41–7.34 (m, 8H), 7.33–7.28 (m, 4H), 7.12 (s, 4H), 6.36 (d, J = 11.7 Hz, 2H), 5.84–5.53 (m, 2H), 3.86–3.71 (m, 2H), 2.76–2.61 (m, 2H), 2.58–2.49 (m, 2H), 0.30 (s, 24H); ^{13}C NMR (150 MHz, $CDCl_3$): δ 138.6, 137.5, 135.8, 134.4, 133.8, 130.6, 129.8, 129.5, 129.2, 128.6, 127.8, 67.0, 33.4, -0.7, -0.8, -4.8, -4.8; ^{29}Si NMR (120 MHz, $CDCl_3$): δ 6.4, -4.1; HRMS (EI, m/z): calcd. for $C_{46}H_{58}O_2Si_4$ $[M]^+$: 754.3514; found: 754.3517.

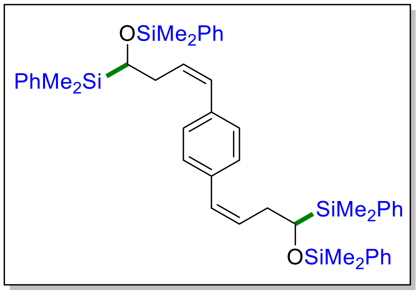

(3*Z*,3'*Z*)-{[(5-Bromo-1,3-phenylene)bis1-(dimethylphenylsilyloxy)but-3-ene-4,1-diyl]bis (dimethylphenylsilane)} (Table 1, *Z*-2s, Reaction time: 8 h, 81%). Employed 2,2'-(5-bromo-1,3-phenylene)difuran (**1s**), $B(C_6F_5)_3$ (4.0 mol%), and dimethylphenylsilane (4.0 equiv.); The compound *Z*-**2s** was prepared according to **GP2** and was purified by flash column chromatography (hexane and ethyl acetate);.colorless liquid; ^{1}H NMR (600 MHz, $CDCl_3$): δ 7.53 (d, J = 7.2 Hz, 4H), 7.48 (d, J = 7.1 Hz, 4H), 7.42–7.29 (m, 14H), 6.95 (s, 1H), 6.26 (d, J = 11.7 Hz, 2H), 5.76–5.55 (m, 2H), 3.76 (t, J = 6.7 Hz, 2H), 2.71–2.53 (m, 2H), 2.46–2.38 (m, 2H), 0.29 (s, 24H); ^{13}C NMR (150 MHz, $CDCl_3$): δ 139.1, 138.3, 137.1, 134.2, 133.6, 132.0, 129.5, 129.4, 129.1, 128.7, 127.9, 127.7, 127.6, 121.8, 66.6, 33.0, -

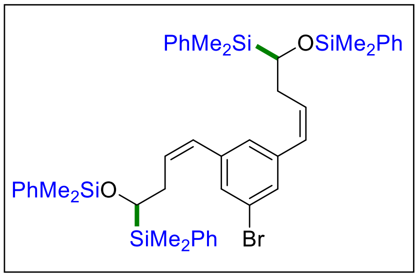

0.94, -0.97, -4.8, -4.9; ^{29}Si NMR (120 MHz, $CDCl_3$): δ 6.5, -4.1; HRMS (ESI, m/z): calcd. for $C_{46}H_{57}BrNaO_2Si_4$ $[M+Na]^+$: 855.2517; found: 855.2498.

(*Z*)-1,3,5-Tris[4-(dimethylphenylsilyl)-4-(dimethylphenylsilyloxy)but-1-en-1-yl]benzene (Table 1, *Z*-2t, Reaction time: 2.5 h, 87%). Employed 1,3,5-tri(furan-2-yl)benzene (**1t**), $B(C_6F_5)_3$ (6.0 mol%), and dimethylphenylsilane (6.0 equiv.); The compound *Z*-**2t** was prepared according to **GP2** and was purified by flash column chromatography (hexane and ethyl acetate);.colorless liquid; ^{1}H NMR (600 MHz, $CDCl_3$): δ 7.58–7.52 (m, 6H), 7.52–7.45 (m, 6H), 7.42–7.28 (m, 18H), 7.01 (s, 3H), 6.33 (d, J = 12.5 Hz, 3H), 5.74–5.61 (m, 3H), 3.78 (d, J = 6.5 Hz, 3H), 2.72–2.61 (m, 3H), 2.61–2.50 (m, 3H), 0.35–0.25 (m, 36H); ^{13}C NMR (150 MHz, $CDCl_3$): δ 138.4, 137.4, 137.1, 134.2, 133.6, 130.5, 129.9, 129.3, 129.1, 127.6, 127.4, 66.8, 33.2, -0.8, -4.6, -4.9; ^{29}Si NMR (120 MHz, $CDCl_3$): δ 6.4, -4.2; HRMS (ESI, m/z): calcd. for $C_{66}H_{84}NaO_3Si_6$ $[M+Na]^+$: 1115.4934; cound: 1115.4914.

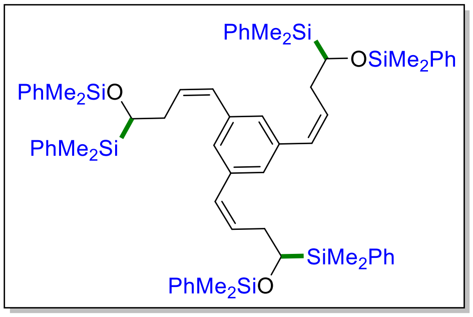

General Procedure for the Cyclopropanation Reaction (Conditions B, GP3, Table 1)

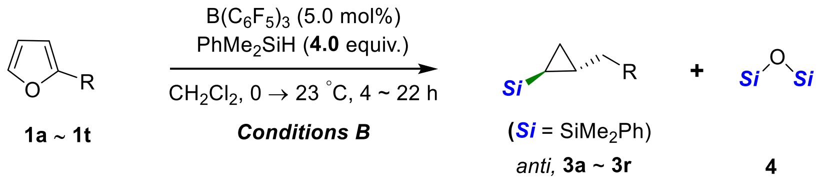

In a flame-dried flask bearing a stirring bar, $B(C_6F_5)_3$ (0.025 mmol, 5.0 mol%) was dissolved in CH_2Cl_2 (0.2 mL). Silane (1.5 ~ 2.0 mmol) was added, and the solution was shaken shortly to make it homogeneous. The corresponding furan derivative (**1a** ~ **1t**, and 1ac, 0.50 mmol) was then added at 0 °C and the reaction mixture was stirred at 23 °C for the indicated time (4 ~ 22 h). After quenching the reaction mixture with Et_3N (10.0 ~ 20.0 mol%), the crude reaction mixture was concentrated under reduced pressure and then purified by flash column chromatography on silica gel (using either hexane or a mixture of hexane and ethyl acetate) to afford the desired products (*anti*, **3a** ~ **3r**, in all cases dr >99/1).

General Procedure for the Purification of Silylated Cyclopropane Products

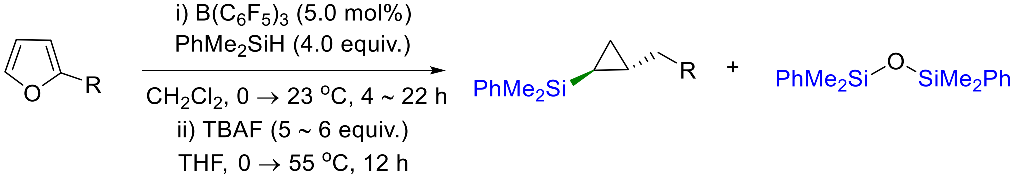

(i) In a flame-dried flask bearing a stirring bar, $B(C_6F_5)_3$ (0.025 mmol, 5.0 mol%) was dissolved in CH_2Cl_2 (0.2 mL), into which silane (1.5 ~ 2.0 mmol) was added, and this catalyst solution was shaken shortly to make it homogeneous. The corresponding furan derivative (**1a** ~ **1t**, 0.50 mmol) was subsequently added at 0 °C and the reaction mixture was stirred at 23 °C for the indicated time (4 ~ 22 h).

(ii) After completion of the reaction, some silylated cyclopropane products were purified by the following procedure: The crude reaction mixture was concentrated under reduced pressure and the resulting residue was diluted with THF (0.5 mL) and cooled down to 0 °C. Then, tetrabutylammonium fluoride (TBAF, 1 M in THF, 5 ~ 6 equiv.) was slowly added into the reaction mixture. The reaction mixture was stirred at 55 °C. After 12 h, the reaction mixture was cooled down to 23 °C, quenched by adding water (5 mL), and extracted with ethyl acetate (5 mL x 3). The combined organic layers were concentrated under reduced pressure and then purified by flash column chromatography on silica gel (using either hexane or mixture of hexane and ethyl acetate) to afford the desired products.

Characterization Data of the Obtained Silylated Cyclopropane Products

***anti*-(2-Ethylcyclopropyl)dimethylphenylsilane (Table 1**, *anti*-**3a**, Reaction time: 6 h, 83%**)**. Employed

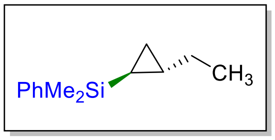

2-methylfuran (**1a**); The compound *anti*-**3a** was prepared according to **GP3** and was purified by flash column chromatography (hexane); colorless liquid; ^1H NMR (600 MHz, $CDCl_3$): δ 7.82–7.76 (m, 2H), 7.56–7.52 (m, 3H), 1.69–1.59 (m, 1H), 1.48–1.38 (m, 1H), 1.19 (t, J = 7.3 Hz, 3H), 0.92–0.83 (m, 1H), 0.69–0.59 (m, 2H), 0.43 (s, 3H), 0.41 (s, 3H), -0.17–-0.30 (m, 1H); ^{13}C NMR (150 MHz, $CDCl_3$): δ 139.6, 133.9, 128.9, 127.8, 29.1, 17.8, 14.13, 9.1, 3.4, -3.4, -3.7; ^{29}Si NMR (120 MHz, $CDCl_3$): δ -2.9; HRMS (EI, m/z): calcd. for for $C_{13}H_{20}Si$ $[M]^+$: 204.1334; found: 204.1333.

Gram-Scale Reaction with ***Z*****-2a (Fig. 4**, 'xi', *anti*-**3a**, Reaction time: 16 h, 77%**)**. *Z*-**2a** (3.54 g, 10.0

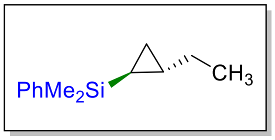

mmol) was reacted with dimethylphenylsilane (2.0 g, 15.0 mmol) in the presence of $B(C_6F_5)_3$ (153.6 mg, 3.0 mol%) in CH_2Cl_2 (4.0 mL). The reaction mixture was stirred at 23 °C for 12 h under argon atmosphere to show 83% conversion determined by a ^1H NMR analysis. The isolated yield was 77% (1.57 g) as colorless liquid.

***anti*-(2-Ethylcyclopropyl)diphenylsilane (Table 1**, *anti*-**3b**, Reaction time: 8 h, 81%**)**. Employed 2-methylfuran (**1a**, 164 mg, 2.0 mmol), diphenylsilane (1.11 g, 6.0 mmol), $B(C_6F_5)_3$ (51.2 mg, 5.0 mol%), and CH_2Cl_2 (1.0 mL) to afford the *anti*-**3b** as colorless liquid; The compound *anti*-**3b** was prepared according to **GP3** and was purified by flash column chromatography (hexane); ^{1}H NMR (600 MHz, $CDCl_3$): δ 7.69 (d, J = 6.9 Hz, 4H), 7.51–7.39 (m, 6H), 4.79 (d, J = 3.8 Hz, 1H), 1.50–1.27 (m, 2H), 1.07 (t, J = 7.3 Hz, 3H), 0.91–0.80 (m, 1H), 0.71–0.49 (m, 2H), -0.03–-0.18 (m, 1H); ^{13}C NMR (150 MHz, $CDCl_3$): δ 135.5, 134.6, 129.7, 127.9, 28.8, 18.6, 13.9, 9.7, 0.12; ^{29}Si NMR (120 MHz, $CDCl_3$): δ -10.7; HRMS (EI, m/z): calcd. for $C_{17}H_{20}Si$ $[M]^+$: 252.1334; found 252.1336.

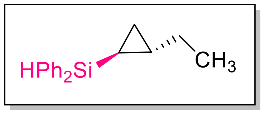

***anti*-(2-Propylcyclopropyl)dimethylphenylsilane (Table 1**, *anti*-**3c**, Reaction time: 5 h, 77%**)**. Employed 2-ethylfuran (**1b**); The compound *anti*-**3c** was prepared according to **GP3** and was purified by flash column chromatography (hexane);colorless liquid; ^{1}H NMR (600 MHz, $CDCl_3$): δ 7.72–7.62 (m, 2H), 7.49–7.40 (m, 3H), 1.57–1.41 (m, 3H), 1.35–1.20 (m, 1H), 1.07–0.94 (m, 3H), 0.84–0.73 (m, 1H), 0.58–0.43 (m, 2H), 0.30 (s, 3H), 0.28 (s, 3H), -0.30–-0.41 (m, 1H); ^{13}C NMR (150 MHz, $CDCl_3$): δ 139.7, 133.9, 128.9, 127.8, 38.3, 23.1, 15.7, 14.2, 9.2, 3.4, -3.4, -3.7; ^{29}Si NMR (120 MHz, $CDCl_3$): δ -2.9; HRMS (EI, m/z): calcd. for $C_{14}H_{22}Si$ $[M]^+$: 218.1491; found 218.1495.

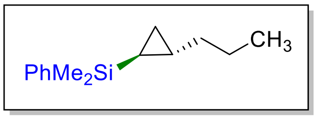

***anti*-(2-Butylcyclopropyl)dimethylphenylsilane (Table 1**, *anti*-**3d**, Reaction time: 5 h, 66%**)**. Employed 2-propylfuran (**1c**); The compound *anti*-**3d** was prepared according to **GP3** and was purified by flash column chromatography (hexane); colorless liquid; ^{1}H NMR (600 MHz, $CDCl_3$): 7.67–7.56 (m, 2H), 7.48–7.29 (m, 3H), 1.47–1.31 (m, 5H), 1.28–1.17 (m, 1H), 0.97–0.90 (m, 3H), 0.79–0.59 (m, 1H), 0.52–0.38 (m, 2H), 0.25 (s, 3H), 0.22 (s, 3H), -0.33–0.49 (m, 1H); ^{13}C NMR (150 MHz, $CDCl_3$): δ 139.7, 133.9, 128.9, 127.8, 35.8, 32.3, 22.7, 15.9, 14.3, 9.3, 3.5, -3.5, -3.7; ^{29}Si NMR (120 MHz, $CDCl_3$): δ -2.9; HRMS (EI, m/z): calcd. for $C_{15}H_{24}Si$ $[M]^+$: 232.1647; found: 232.1646.

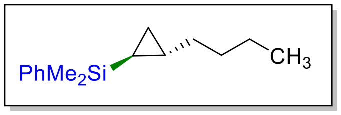

***anti*-(2-Hexcyclopropyl)dimethylphenylsilane (Table 1**, *anti*-**3e**, Reaction time: 6 h, 83%**)**. Employed 2-pentylfuran (**1d**) under neat conditions; The compound *anti*-**3e** was prepared according to **GP3** and was purified by flash column chromatography (hexane); colorless liquid; ^{1}H NMR (600 MHz, $CDCl_3$): δ 7.73–7.53 (m, 2H), 7.36–7.01 (m, 3H), 1.42–1.04 (m, 10H), 0.91–0.77 (m, 3H), 0.70–0.58 (m, 1H), 0.49–0.29 (m, 2H), 0.16 (s, 3H), 0.14 (s, 3H), -0.45–-0.55 (m, 1H); ^{13}C NMR (150 MHz, $CDCl_3$): δ 139.3, 133.5, 128.5, 127.4, 76.8, 35.7, 31.8, 29.6, 29.0, 22.5, 15.6, 13.9, 8.9, 3.1, -3.8, -4.1; ^{29}Si NMR

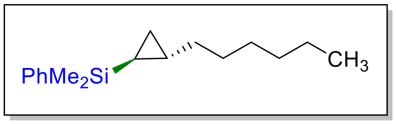

(120 MHz, $CDCl_3$): δ -2.9; HRMS (EI, m/z): calcd. for $C_{17}H_{28}Si$ $[M]^+$: 260.1960; found: 260.1962.

***anti*-2-{2-[(1,1'-Biphenyl-4-yl)ethyl]cyclopropyl}dimethylphenylsilane (Table 1**, ***anti*-3f**, Reaction time: 8 h, 74% for two steps**)**. Employed 2- [(1,1'-biphenyl)-4-ylmethyl]furan (**1ac**). The compound *anti*-**3f** was prepared according to **GP3**. The crude reaction mixture was concentrated under reduced pressure and the resulting residue was purified by using tetrabutylammonium fluoride (TBAF, 1 M in THF, 3.0 mL) in THF (0.5 mL) to afford *anti*-**3f** as colorless liquid. ^1H NMR (600 MHz, $CDCl_3$): δ 7.71 (t, J = 7.0 Hz, 4H), 7.63 (d, J = 8.0 Hz, 2H), 7.54 (t, J = 7.6 Hz, 2H), 7.50–7.46 (m, 3H), 7.44 (t, J = 6.9 Hz, 1H), 7.35 (d, J = 7.9 Hz, 2H), 2.92–2.81 (m, 2H), 1.93–1.84 (m, 1H), 1.73–1.62 (m, 1H), 0.94–0.82 (m, 1H), 0.67–0.52 (m, 2H), 0.35 (s, 3H), 0.33 (s, 3H), -0.19–-0.28 (m, 1H); ^{13}C NMR (150 MHz, $CDCl_3$): δ 141.8, 141.3, 139.4, 138.7, 133.9, 128.9, 128.9, 128.8, 127.8, 127.1, 38.0, 35.9, 15.9, 9.3, 3.9, -3.5, -3.6; ^{29}Si NMR (120 MHz, $CDCl_3$): δ -2.9; HRMS (ESI, m/z): calcd. for $C_{25}H_{28}NaSi$ $[M+Na]^+$: 379.1858; found: 379.1852.

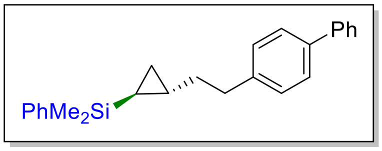

***anti*-2-{[(1-phenyl)-4-ylmethyl]cyclopropyl}dimethylphenylsilane (Table 1**, *anti*-**3g**, Reaction time: 10 h, 76% for two steps**)**. Employed 2-phenylfuran (**1j**) at 0 °C to 23 °C. The compound *anti*-**3g** was prepared according to **GP3**. The crude reaction mixture was concentrated under reduced pressure and the resulting residue was purified by using tetrabutylammonium fluoride (TBAF, 1 M in THF, 3.0 mL) in THF (0.5 mL) to afford *anti*-**3g** as colorless liquid ^1H NMR (600 MHz, $CDCl_3$): δ 7.51–7.47 (m, 2H), 7.37–7.31 (m, 3H), 7.31–7.27 (m, 2H), 7.26–7.19 (m, 3H), 2.72 (dd, J = 14.5, 6.6 Hz, 1H), 2.59 (dd, J = 14.5, 6.8 Hz, 1H), 1.05–0.96 (m, 1H), 0.65–0.50 (m, 2H), 0.18 (s, 3H), 0.16 (s, 3H), -0.17–-0.31 (m, 1H); ^{13}C NMR (150 MHz, $CDCl_3$): δ 142.1, 139.3, 133.9, 128.9, 128.6, 128.4, 127.8, 126.0, 41.7, 17.0, 9.3, 3.7, -3.6, -3.7; ^{29}Si NMR (120 MHz, $CDCl_3$): δ –2.8; HRMS (ESI, m/z): calcd. for $C_{18}H_{22}NaSi$ $[M+Na]^+$: 289.1388; found: 289.1392.

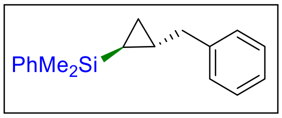

***anti*-{2-[4-(Trifluoromethyl)benzyl]cyclopropyl}dimethylphenylsilane (Table 1**, Reaction time: 6 h, *anti*-**3h**, 80% for two steps**)**. Employed 2-[4-(trifluoromethyl)phenyl]-furan (**1e**). The compound *anti*-**3h** was prepared according to **GP3**. The crude reaction mixture was concentrated under reduced pressure and the resulting residue was purified by using tetrabutylammonium fluoride (TBAF, 1 M in THF, 3.0 mL) in THF (0.5 mL) to afford *anti*-**3h** as colorless liquid. ^1H NMR (600 MHz, $CDCl_3$): δ 7.54 (d, J = 7.8 Hz, 2H), 7.48 (d, J = 6.9 Hz, 2H), 7.39 (d, J = 9.9 Hz, 1H), 7.34 (t, J = 7.1 Hz, 4H), 2.82 (dd, J = 14.7, 6.3 Hz, 1H), 2.59 (dd, J = 14.6, 7.1 Hz, 1H), 1.04–0.94 (m, 1H), 0.68–0.56 (m, 2H), 0.22 (s, 3H), 0.17 (s, 3H), -0.13–-0.31 (m, 1H); ^{13}C NMR (150 MHz, $CDCl_3$): δ 146.2, 139.0, 133.8, 129.1, 128.8, 127.8, 126.2,

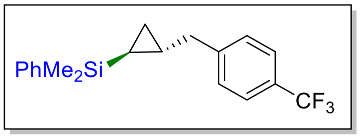

125.3 (q, J = 3.7 Hz), 124. (q, J = 273 Hz), 41.5, 16.8, 9.3, 3.9, -3.6, -3.9; ^{29}Si NMR (120 MHz, $CDCl_3$): δ -2.9; ^{19}F NMR ($CDCl_3$, 375 MHz): -62.3; HRMS (EI, m/z): calcd. for $C_{19}H_{21}F_3Si$ $[M]^+$: 334.1365; found: 334.1367.

***anti*-2-[(4-Chlorobenzyl)cyclopropyl]dimethylphenylsilane (Table 1**, *anti*-**3i**, Reaction time: 10 h, 80% for two steps**)**. Employed 2-(4-chlorophenyl)furan (**1g**). The compound *anti*-**3i** was prepared according to **GP3**. The crude reaction mixture was concentrated under reduced pressure and the resulting residue was purified by using tetrabutylammonium fluoride (TBAF, 1 M in THF, 3.0 mL) in THF (0.5 mL) to afford *anti*-**3i** as colorless liquid. ^{1}H NMR (600 MHz, $CDCl_3$): δ 7.48 (d, J = 7.5 Hz, 2H), 7.41–7.30 (m, 3H), 7.24 (d, J = 7.5 Hz, 2H), 7.15 (d, J = 7.9 Hz, 2H), 2.72 (dd, J = 14.5, 6.3 Hz, 1H), 2.51 (dd, J = 14.5, 7.0 Hz, 1H), 1.00–0.91 (m, 1H), 0.66–0.53 (m, 2H), 0.20 (s, 3H), 0.16 (s, 3H), -0.17– -0.29 (m, 1H); ^{13}C NMR (150 MHz, $CDCl_3$): δ 140.5, 139.1, 133.8, 131.7, 129.9, 129.0, 128.4, 127.8, 41.0, 16.9, 9.3, 3.8, -3.6, -3.8; ^{29}Si NMR (120 MHz, $CDCl_3$): δ -2.9; HRMS (EI, m/z): calcd. for $C_{18}H_{21}ClSi$ $[M]^+$: 300.1101; found: 300.1097.

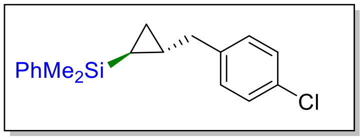

***anti*-2-[(4-Bromobenzyl)cyclopropyl]dimethylphenylsilane (Table 1**, *anti*-**3j**, Reaction time: 10 h, 71% for two steps**)**. Employed 2-(4-bromophenyl)-furan (**1h**). The compound *anti*-**3j** was prepared according to **GP3**. The crude reaction mixture was concentrated under reduced pressure and the resulting residue was purified by using tetrabutylammonium fluoride (TBAF, 1 M in THF, 3.0 mL) in THF (0.5 mL) to afford *anti*-**3j** as colorless liquid. ^{1}H NMR (600 MHz, $CDCl_3$): δ 7.49 (d, J = 6.4 Hz, 2H), 7.43–7.38 (m, 3H), 7.35 (t, J = 7.0 Hz, 2H), 7.11 (d, J = 8.3 Hz, 2H), 2.71 (dd, J = 14.6, 6.4 Hz, 1H), 2.50 (dd, J = 14.6, 7.0 Hz, 1H), 1.07–0.89 (m, 1H), 0.68–0.54 (m, 2H), 0.21 (s, 3H), 0.17 (s, 3H), -0.15– -0.31 (m, 1H); ^{13}C NMR (150 MHz, $CDCl_3$): δ 141.0, 139.1, 133.8, 131.4, 130.3, 129.0, 127.8, 119.8, 41.1, 16.9, 9.3, 3.8, -3.6, -3.8; ^{29}Si NMR (120 MHz, $CDCl_3$): δ -2.9; HRMS (EI, m/z): calcd. for $C_{18}H_{21}BrSi$ $[M]^+$: 344.0596; found: 344.0592.

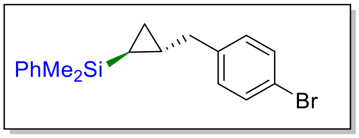

***anti*-2-{[(1,1'-Biphenyl)-4-ylmethyl]cyclopropyl}dimethylphenylsilane (Table 1**, *anti*-**3k**, Reaction time: 10 h, 85%**)**. Employed 2-(1,1'-biphenyl-4-yl)furan (**1i**) at -78 °C to 40 °C; The compound *anti*-**3k** was prepared according to **GP3** and was purified by flash column chromatography (hexane); colorless liquid; ^{1}H NMR (600 MHz, $CDCl_3$): δ 7.65 (d, J = 7.6 Hz, 2H), 7.58–7.51 (m, 4H), 7.49 (t, J = 7.8 Hz, 2H), 7.43–7.28 (m, 6H), 2.85–2.73 (m, 1H), 2.70–2.58 (m, 1H), 1.07 (q, J = 5.8 Hz, 1H), 0.71–0.58 (m, 2H), 0.24 (s, 3H), 0.21 (s, 3H), -0.11–-0.29 (m, 1H); ^{13}C NMR (150 MHz, $CDCl_3$): δ 141.2, 141.1, 139.1, 138.8, 133.8,

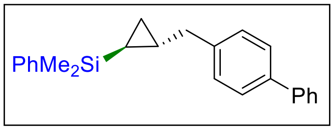

128.8, 128.7, 127.7, 127.04, 127.02, 41.2, 16.9, 9.2, 3.6, -3.6, -3.7; ^{29}Si NMR (120 MHz, $CDCl_3$): δ -2.8; HRMS (EI, m/z): calcd. for $C_{24}H_{26}Si$ $[M]^+$: 342.1804; found: 342.1801.

***anti*-2-{[(4-Methylthio)benzyl]cyclopropyl}dimethylphenylsilane (Table 1**, *anti*-**3l**, Reaction time: 6 h, 90%**)**. Employed 2-[4-(methylthio)phenyl]furan (**1m**); The compound *anti*-**3l** was prepared according to **GP3** and was purified by flash column chromatography (hexane and ethyl acetate); colorless liquid; ^{1}H NMR (600 MHz, $CDCl_3$): δ 7.53 (d, J = 7.0 Hz, 2H), 7.41–7.36 (m, 3H), 7.25 (d, J = 8.0 Hz, 2H), 7.20 (d, J = 8.1 Hz, 2H), 2.74 (dd, J = 14.5, 6.4 Hz, 1H), 2.58 (dd, J = 14.5, 6.9 Hz, 1H), 2.53 (s, 3H), 1.07–0.99 (m, 1H), 0.66–0.58 (m, 2H), 0.24 (s, 3H), 0.21 (s, 3H), -0.14– -0.24 (m, 1H); ^{13}C NMR (150 MHz, $CDCl_3$): δ 139.3, 135.5, 133.9, 129.1, 127.8, 127.3, 41.1, 17.0, 16.54, 9.2, 3.7, -3.6, -3.7; ^{29}Si NMR (120 MHz, $CDCl_3$): δ -2.9; HRMS (EI, m/z): calcd. for $C_{19}H_{24}SSi$ $[M]^+$: 312.1368; found: 312.1366.

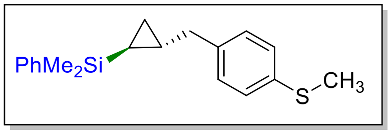

***anti*-[2-(3-Methylbenzyl)cyclopropyl]dimethylphenylsilane (Table 1**, *anti*-**3m**, Reaction time: 12 h, 83% for two steps**)**. Employed 2-(*m*-tolyl)furan (**1k**) at -78 °C to 23 °C; The compound *anti*-**3m** was prepared according to **GP3**. The crude reaction mixture was concentrated under reduced pressure and the resulting residue was purified by using tetrabutylammonium fluoride (TBAF, 1 M in THF, 2.5 mL) in THF (0.5 mL) to afford *anti*-**3m** as colorless liquid. ^{1}H NMR (600 MHz, $CDCl_3$): δ 7.50 (d, J = 7.8 Hz, 2H), 7.39–7.30 (m, 3H), 7.18 (t, J = 7.5 Hz, 1H), 7.06 (s, 1H), 7.05–7.01 (m, 2H), 2.69–2.63 (m, 1H), 2.61–2.49 (m, 1H), 2.34 (s, 3H), 1.05–0.89 (m, 1H), 0.61–0.52 (m, 2H), 0.18 (s, 3H), 0.16 (s, 3H), -0.16– -0.30 (m, 1H); ^{13}C NMR (150 MHz, $CDCl_3$): δ 141.9, 139.2, 137.7, 133.7, 129.1, 128.8, 128.1, 127.6, 126.6, 125.4, 41.5, 21.4, 16.8, 9.1, 3.5, -3.7, -3.8; ^{29}Si NMR (120 MHz, $CDCl_3$): δ -2.9; HRMS (EI, m/z): calcd. for $C_{19}H_{24}Si$ $[M]^+$: 280.1647; found: 280.1648.

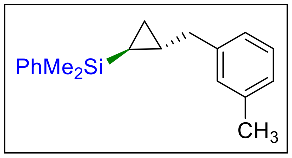

***anti*-[2-(3,5-Dibromobenzyl)cyclopropyl]dimethylphenylsilane (Table 1**, *anti*-**3n**, Reaction time: 22 h, 81%**)**. Employed 2-(3,5-dibromophenyl)furan (**1o)** at -78 °C to 40 °C; The compound *anti*-**3n** was prepared according to **GP3** and was purified by flash column chromatography (hexane and ethyl acetate); colorless liquid; ^{1}H NMR (600 MHz, $CDCl_3$): δ 7.58–7.47 (m, 3H), 7.43–7.36 (m, 3H), 7.34 (s, 2H), 2.72–2.64 (m, 1H), 2.52–2.43 (m, 1H), 1.00–0.87 (m, 1H), 0.64–0.54 (m, 2H), 0.22 (s, 3H), 0.21 (s, 3H), -0.18– -0.27 (m, 1H); ^{13}C NMR (150 MHz, $CDCl_3$): δ 146.0, 138.6, 133.7, 131.6, 130.1, 129.0, 127.7, 122.7, 40.9, 16.4, 9.3, 3.9, -3.6, -3.8; ^{29}Si NMR (120 MHz, $CDCl_3$): δ -2.9.

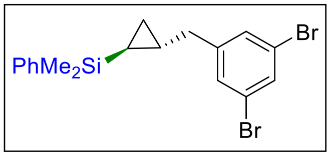

***anti*-[2-(2,4,6-Triisopropylbenzyl)cyclopropyl]dimethylphenylsilane (Table 1**, *anti*-**3o**, Reaction time: 12 h, 77% for two steps**)**. Employed 2-(2,4,6-triisopropylphenyl)furan (**1p**). The compound *anti*-**3o** was prepared according to **GP3**. The crude reaction mixture was concentrated under reduced pressure and the resulting residue was purified by using tetrabutylammonium fluoride (TBAF, 1 M in THF, 3.0 mL) in THF (0.5 mL) to afford *anti*-**3o** as colorless liquid. ^{1}H NMR (600 MHz, $CDCl_3$): δ 7.57 (d, J = 7.3 Hz, 2H), 7.45–7.33 (m, 3H), 7.06 (s, 2H), 3.35–3.26 (m, 2H), 3.02 (dd, J = 14.5, 4.2 Hz, 1H), 2.99–2.91 (m, 1H), 2.77 (dd, J = 14.5, 6.3 Hz, 1H), 1.35 (s, 3H), 1.34 (s, 3H), 1.30 (s, 6H), 1.29 (s, 6H), 0.96–0.87 (m, 1H), 0.67–0.60 (m, 1H), 0.57–0.51 (m, 1H), 0.27 (s, 3H), 0.23 (s, 3H), -0.17– -0.29 (m, 1H); ^{13}C NMR (150 MHz, $CDCl_3$): δ 147.0, 146.4, 139.3, 133.9, 131.9, 128.9, 127.8, 120.9, 34.3, 30.9, 29.5, 24.7, 24.4, 24.3, 16.8, 9.4, 2.6, -3.7; ^{29}Si NMR (120 MHz, $CDCl_3$): δ -2.7; HRMS (EI, m/z): calcd. for $C_{27}H_{40}Si$ $[M]^+$: 392.2899; found: 392.2898.

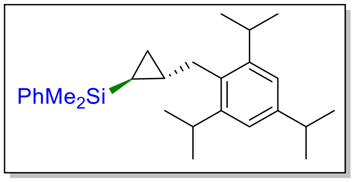

***anti*-[2-(Phenanthren-9-ylmethyl)cyclopropyl]dimethylphenylsilane (Table 1**, *anti*-**3p**, Reaction time: 10 h, 81%**)**. Employed 2-(phenanthren-9-yl)furan (**1q**); The compound *anti*-**3p** was prepared according to **GP3** and was purified by flash column chromatography (hexane and ethyl acetate); colorless liquid; ^{1}H NMR (600 MHz, $CDCl_3$): δ 8.78 (d, J = 7.9 Hz, 1H), 8.71 (d, J = 8.0 Hz, 1H), 8.16 (d, J = 7.9 Hz, 1H), 7.82 (d, J = 7.6 Hz, 1H), 7.75–7.57 (m, 5H), 7.51 (d, J = 7.0 Hz, 2H), 7.35 (t, J = 7.1 Hz, 1H), 7.28 (t, J = 7.4 Hz, 2H), 3.32 (dd, J = 15.3, 5.9 Hz, 1H), 3.08 (dd, J = 15.3, 6.7 Hz, 1H), 1.41–1.25 (m, 1H), 0.79–0.67 (m, 2H), 0.25 (s, 3H), 0.21 (s, 3H), -0.08–-0.11 (m, 1H); ^{13}C NMR (150 MHz, $CDCl_3$): δ 139.2, 136.0, 133.9, 132.1, 131.6, 130.7, 129.8, 128.9, 128.3, 127.8, 126.7, 126.6, 126.2, 126.1, 125.9, 124.5, 123.3, 122.5, 38.7, 15.5, 9.7, 4.2, -3.5, -3.7; ^{29}Si NMR (120 MHz, $CDCl_3$): δ -2.8; HRMS (EI, m/z): calcd. for $C_{26}H_{26}Si$ $[M]^+$: 366.1804; found: 366.1803.

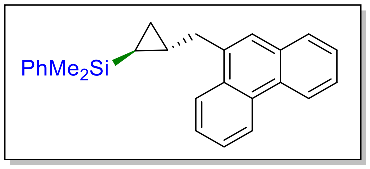

***anti*-{[(5-Bromo-1,3-phenylene)bis(methylene)]bis(cyclopropane-2,1-diyl)bis(dimethylphenylsilane)} (Table 1**, *anti*-**3q**, Reaction time: 12 h, 75%**)**. Employed 2,2'-(5-bromo-1,3-phenylene)difuran (**1s**), $B(C_6F_5)_3$ (10.0 mol%), and dimethylphenylsilane (8.0 equiv.); The initial temperature was -78 °C and the recation was stirred for 12 h at 23 °C (*anti*-**3q**, 75%); The compound *anti*-**3q** was prepared according to **GP3** and was purified by flash column chromatography (hexane and ethyl acetate); colorless liquid; ^{1}H NMR (600 MHz, $CDCl_3$): δ 7.48 (d, J = 6.2 Hz, 4H), 7.39–7.29 (m, 6H), 7.24 (s, 2H), 6.97 (s, 1H), 2.61 (dd, J = 14.7, 6.5 Hz, 2H), 2.48 (dd, J = 14.7, 6.8 Hz, 2H), 0.92 (q, J = 6.7 Hz, 2H), 0.59–0.51 (m, 4H), 0.17 (s, 12H), -0.26 (q, J = 7.8 Hz, 2H); ^{13}C NMR (150 MHz, $CDCl_3$): δ 144.1, 138.9, 133.7, 128.86, 128.84, 127.6, 127.2, 122.1, 41.1, 16.6, 16.6, 9.16, 9.15, 3.7, -3.6, -3.8; ^{29}Si NMR (120

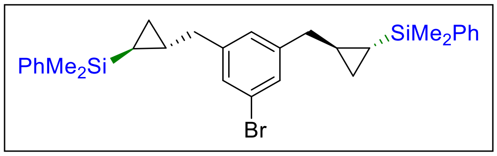

MHz, $CDCl_3$): δ -2.9; HRMS (EI, m/z): calcd. for $C_{30}H_{37}BrSi_2$ $[M]^+$: 532.1617; found: 532.1616.

***anti*-1,3,5-Tris{[2-(dimethylphenylsilyl)cyclopropyl]methyl}benzene (Table 1**, *anti*-**3r**, Reaction time: 8 h, 86%**)**. Employed 1,3,5-tri(furan-2-yl)benzene (**1t**), $B(C_6F_5)_3$ (20.0 mol%), and dimethylphenylsilane (12.0 equiv.). The initial temperature was -78 °C and the recation was stirred for 12 h at 23 °C (*anti*-**3r**, 86%); The compound *anti*-**3r** was prepared according to **GP3** and was purified by flash column chromatography (hexane and ethyl acetate); colorless liquid; ^1H NMR (400 MHz, $CDCl_3$): δ 7.69–7.61 (m, 6H), 7.50–7.42 (m, 9H), 7.11 (s, 3H), 2.82–2.61 (m, 6H), 1.21–1.00 (m, 3H), 0.82–0.56 (m, 6H), 0.32 (s, 9H), 0.31 (s, 9H), 0.00– -0.23 (m, 3H); ^{13}C NMR (150 MHz, $CDCl_3$): δ 141.8, 139.3, 133.8, 128.9, 127.7, 126.2, 41.6, 17.0 (t, J = 4.5 Hz, 3C), 9.2 (t, J = 3.0 Hz, 3C), 3.6, -3.4, -3.6; ^{29}Si NMR (120 MHz, $CDCl_3$): δ -2.8; HRMS (ESI, m/z): calcd. for $C_{42}H_{54}NaSi_3$ $[M+Na]^+$: 665.3431; found: 665.3432.

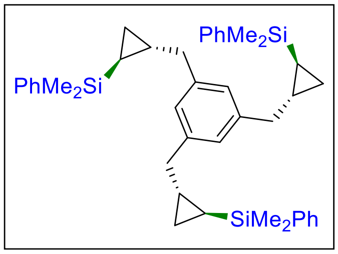

1,1,3,3-Tetramethyl-1,3-diphenyldisiloxane (4, Fig. 2A-C, and Table 1, Fig. 3D-E): ^1H NMR (600 MHz, $CDCl_3$): δ 7.68–7.52 (m, 4H), 7.50–7.27 (m, 6H), 0.36 (s, 12H); ^{13}C NMR (150 MHz, $CDCl_3$): δ.139.9, 133.2, 129.4, 127.9, 1.0; ^{29}Si NMR (120 MHz, $CDCl_3$): δ –1.2.

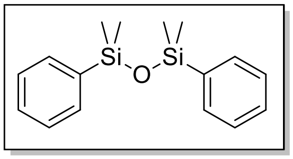

General Procedure for the Synthesis of (*Z*)-α-Silyloxyallylsilanes (GP4)

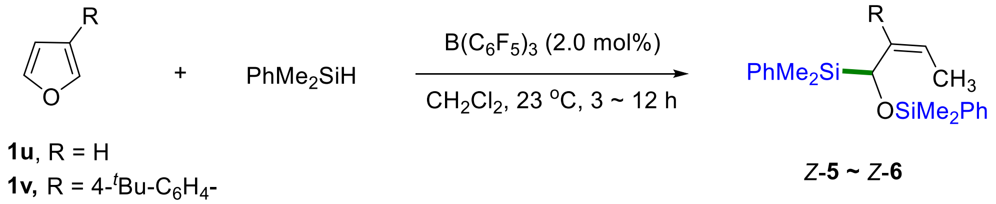

In a flame-dried flask bearing a stirring bar, $B(C_6F_5)_3$ (0.01 ~ 0.02 mmol, 2.0 mol%) was dissolved in CH_2Cl_2 (0.4 ~ 0.8 mL), into which silane (1.025 ~ 2.050 mmol) was added, and the solution was shaken shortly to make it homogeneous. The corresponding furan derivative (**1u** ~ **1v**, 0.50 ~ 1.0 mmol) was then added and the reaction mixture was stirred at 23 °C for the indicated time (3 ~ 12 h). After quenching the reaction mixture with Et_3N (10 ~ 20 mol%), the crude reaction mixture was concentrated under reduced pressure and then purified by flash column chromatography on silica gel (using either hexane only or a mixture of hexane/ethyl acetate) to afford the desired products (*Z*-**5** ~ *Z*-**6**, *Z*/*E* >99/1).

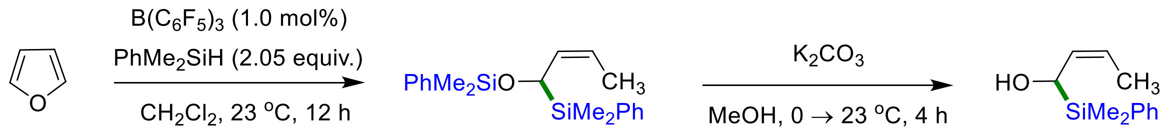

Iu *Z*-**5'** *Z*-**5**, 74%

(*Z*)-1-(Dimethylphenylsilyl)but-2-en-1-ol (Table 2, *Z*-**5**, 74% over two steps): employed furan (**1u**, 680

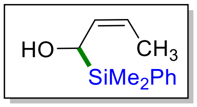

mg, 10.00 mmol) and dimethylphenylsilane (2.793 g, 20.5 mmol) with $B(C_6F_5)_3$ (51.2 mg, 1.0 mol%) in CH_2Cl_2 (8.0 mL) at 23 °C for 12 h to afford *Z*-**5'** as colorless liquid. The compound *Z*-**5'** was prepared according to **GP4** and the crude compound was treated with K_2CO_3 (2.76 g, 20.0 mmol) in MeOH (40 mL) to give *Z*-**5** (1.524 g, 74% for two steps) as colorless liquid; ^1H NMR (600 MHz, $CDCl_3$): δ 7.62–7.57 (m, 2H), 7.43–7.33 (m, 3H), 5.56–5.37 (m, 2H), 4.49 (d, J = 2.5 Hz, 1H), 1.49 (d, J = 3.3 Hz, 3H), 1.34 (brs, 1H), 0.37 (d, J = 2.5 Hz, 3H), 0.34 (d, J = 2.5 Hz, 3H); ^{13}C NMR (150 MHz, $CDCl_3$): δ 136.2, 134.2, 131.2, 129.3, 127.8, 123.5, 63.2, 13.5, -5.5, -6.0; ^{29}Si NMR (120 MHz, $CDCl_3$): δ -4.2; HRMS (ESI, m/z): calcd. for $C_{12}H_{18}NaOSi$ $[M+Na]^+$: 229.1025; found: 229.1003.

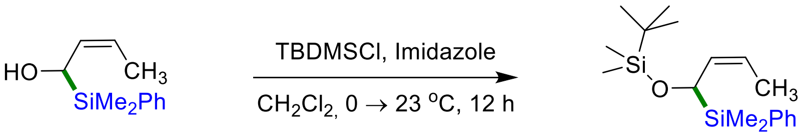

Z-**5** *Z*-**5''**, 96%

(*Z*)-*tert*-Butyl[1-(dimethylphenylsilyloxy)but-2-en-1-yl]dimethylsilane (Fig. 4, *Z*-**5''**, Reaction time: 12

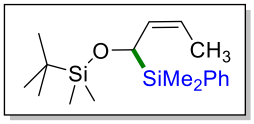

h, 96%**)**. Employed *Z*-**5** (0.206 g, 1.0 mmol), *tert*-butyldimethylsilylchloride (TBDMSCl, 0.18 g, 1.2 mmol), and imidazole (0.136 g, 2.0 mmol) in CH_2Cl_2 (4 mL) to afford *Z*-**5''** (0.291 g, 96%) as colorless oil; ^1H NMR (600 MHz, $CDCl_3$): δ 7.65–7.55 (m, 2H), 7.41–7.32 (m, 3H), 5.48–5.40 (m, 1H), 5.36–5.26 (m, 1H), 4.43 (d, J = 9.8 Hz, 1H), 1.43 (dd, J = 6.9, 1.5 Hz, 3H), 0.89 (s, 9H), 0.35 (s, 3H), 0.32 (s, 3H), -0.02 (s, 3H), -0.04 (s, 3H); ^{13}C NMR (150 MHz, $CDCl_3$): δ 137.1, 134.3, 132.7, 128.9, 127.4, 120.9, 64.1, 25.8, 18.1, 13.42, -4.4, -5.2, -5.5, -5.8; ^{29}Si NMR (120 MHz, $CDCl_3$): 19.9, -4.9; IR (neat, cm^{-1}): 2955, 2855, 1471, 1297, 1113, 1047, 998, 832, 697; HRMS (ESI, m/z): calcd. for $C_{18}H_{32}NaOSi_2$ $[M+Na]^+$: 343.1889; found: 343.1863.

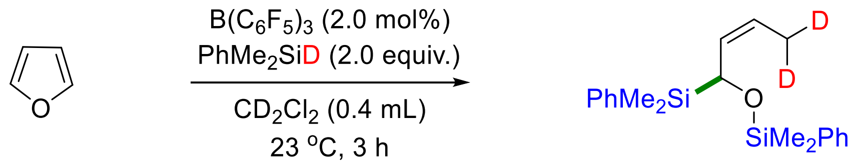

1u *Z*-**5'**-d_2, 66%

(*Z*)-[1-(Dimethylphenylsilyloxy)but-2-en-1-yl]dimethylphenylsilane-d_2 (Table 2, *Z*-5'-d_2, Reaction time: 3 h, 66%): Employed furan (**1u**); The compound *Z*-**5'**-d_2 was prepared according to **GP4** with $PhMe_2SiD$ in presence of 2.0 mol% of $B(C_6F_5)_3$ and was purified by flash column chromatography (hexane and ethyl acetate); colorless liquid; ^1H NMR (600 MHz, $CDCl_3$): δ 7.63–7.57 (m, 2H), 7.55–7.47 (m, 2H), 7.44–7.30 (m, 6H), 5.56–5.46 (m, 1H), 5.30 (dd, J = 10.9, 7.0 Hz, 1H), 4.45 (d, J = 10.0 Hz, 1H), 1.29 (s, 1H), 0.35 (s, 3H), 0.31 (s, 3H), 0.30 (s, 6H); ^{13}C NMR (150 MHz, $CDCl_3$): δ 138.8, 137.2, 134.5, 133.7, 132.3, 129.4, 129.2, 127.7, 127.6, 121.7, 64.5, 12.9 (p, J = 19.3 Hz), -0.9, -1.0, -5.5, -5.7; ^{29}Si NMR (120 MHz, $CDCl_3$): δ 8.1, -4.9; ^2H NMR (60 MHz, $CDCl_3$) δ 1.29 (s, 2D); HRMS (EI, m/z): calcd. for $C_{12}H_{26}D_2OSi$ $[M]^+$: 342.1804; found: 342.1802.

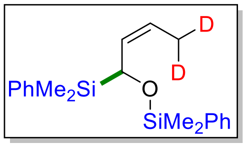

(*Z*)-[2-(4-*tert*-Butyl-phenyl)-1-(dimethylphenylsilyloxy)but-2-en-1-yl]dimethylphenylsilane (Table 2, *Z*-6, Reaction time: 3 h, 84%). Employed 3-[4-(*tert*-butyl)phenyl]-furan (**1v**); The compound *Z*-**6** was prepared according to **GP4** and was purified by flash column chromatography (hexane and ethyl acetate); colorless liquid; ^1H NMR (600 MHz, $CDCl_3$): δ 7.53 (d, J = 7.2 Hz, 2H), 7.48–7.40 (m, 3H), 7.39–7.34 (m, 3H), 7.30 (t, J = 7.3 Hz, 3H), 7.22 (d, J = 7.3 Hz, 3H), 5.57 (q, J = 7.1 Hz, 1H), 4.79 (s, 1H), 1.88–1.53 (m, 3H), 1.34 (s, 9H), 0.35 (s, 3H), 0.31 (s, 3H), 0.19 (s, 3H), 0.13 (s, 3H); ^{13}C NMR (150 MHz, $CDCl_3$): δ 149.3, 141.4, 140.6, 138.2, 137.8, 134.4, 133.9, 129.5, 129.0, 127.9, 127.8, 127.5, 124.6, 124.2, 67.6, 34.5, 31.5, 14.9, -1.0, -1.4, -4.4, -4.6; ^{29}Si NMR (120 MHz, $CDCl_3$): δ 8.4, -3.8; HRMS (EI, m/z): calcd. for $C_{30}H_{40}OSi_2$ $[M]^+$: 472.2618; found: 472.2616.

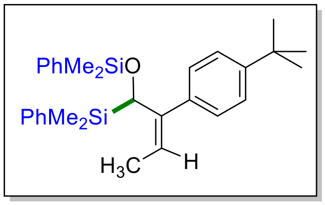

General Procedure for the Synthesis of γ-Silylated Ketone from 2,3-Dimethylfuran (GP5)

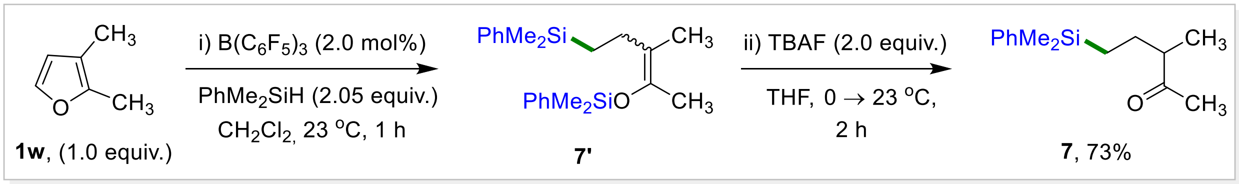

5-(Dimethylphenylsilyl)-3-methylpentan-2-one (Table 2, 7, 73% over two steps). (*i*) Dimethylphenylsilane (2.79 g, 20.5 mmol) was added to a solution of $B(C_6F_5)_3$ (0.102 g, 2.0 mol%) in CH_2Cl_2 (8.0 mL) at 23 °C in a 25 mL round bottom flask, and the solution was stirred for 2 min. 2,3-dimethylfuran (**1w**, 0.96 g, 10.0 mmol) was then added into the solution and stirred at 23 °C for 1 h. The reaction mixture was quenched with trimethylamine (Et_3N, 50 mg, 0.5 mmol), concentrated under reduced pressure and the resulting residue containing **7'** was subjected to hydrolysis.

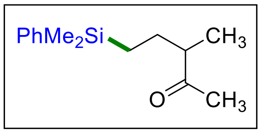

(*ii*) The above reaction mixture was diluted with anhydrous THF (5 mL) and cooled down to 0 °C. Tetrabutylammonium fluoride (TBAF, 1 M in THF, 20 mL, 20.0 mmol) was slowly added into the reaction mixture, and stirred at 23 °C. After 2 h, the reaction mixture was quenched by adding water (20 mL) and extracted with ethyl acetate (15 mL x 3). The combined organic layers were dried over Na_2SO_4, evaporated under reduced pressure, and purified by column chromatography on silica gel (ethyl acetate/hexane, 1/9) to give **7** (1.71 g, 73% over two steps) as colorless liquid; ^{1}H NMR (600 MHz, $CDCl_3$): δ 7.54–7.47 (m, 2H), 7.40–7.30 (m, 3H), 2.53–2.40 (m, 1H), 2.07 (s, 3H), 1.73–1.58 (m, 1H), 1.45–1.32 (m, 1H), 1.06 (d, J = 6.9 Hz, 3H), 0.78–0.62 (m, 2H), 0.28 (s, 6H); ^{13}C NMR (150 MHz, $CDCl_3$): δ 212.6, 138.8, 133.5, 128.9, 127.8, 49.8, 28.1, 27.2, 15.7, 13.0, -3.19, -3.27; ^{29}Si NMR (120 MHz, $CDCl_3$): δ -2.8; HRMS (ESI, m/z): calcd. for $C_{14}H_{22}NaOSi$ $[M+Na]^+$: 257.1338; found: 257.1330.

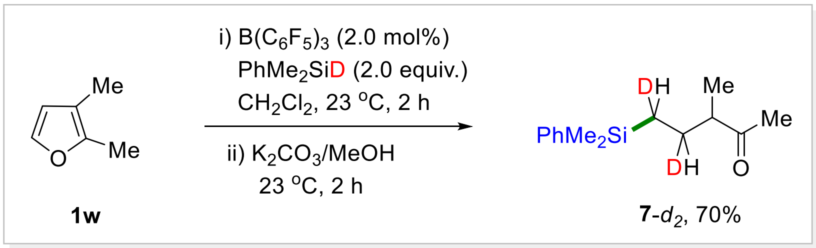

5-(Dimethylphenylsilyl)-3-methylpentan-2-one-*d*$_2$ (Table 2, 7-*d*$_2$, Reaction time: 4 h for two steps, 70% over two steps**):** The compound **7**-*d*$_2$ was prepared according to **GP5** in presence of $PhMe_2SiD$ with modified procedure (in the second step of hydrolysis, K_2CO_3 was used instead of TBAF) and was purified by flash column chromatography (hexane and ethyl acetate); colorless liquid; ^{1}H NMR (600 MHz, $CDCl_3$): δ 7.53–7.46 (m, 2H), 7.40–7.32 (m, 3H), 2.46 (p, J = 6.6 Hz, 1H), 2.07 (s, 3H), 1.63 (s, 0.5H), 1.37 (s, 0.5H), 1.06 (d, J = 6.9 Hz, 3H), 0.68 (s, 0.5H), 0.65 (s, 0.5H), 0.28 (s, 6H); ^{13}C NMR (150 MHz, $CDCl_3$): δ 212.8, 139.0, 133.6, 129.1, 127.9, 49.9, 28.2, 26.8 (t, J = 19.6 Hz), 15.8, 12.7 (t, J = 19.7 Hz), -3.1; ^{29}Si NMR (120 MHz, $CDCl_3$): δ -2.8; ^{2}H NMR (60 MHz, $CDCl_3$) δ 1.64 (s, 0.5D), 1.38 (s, 0.5D), 0.68 (s, 1D); HRMS (ESI, m/z): calcd. for $C_{14}H_{20}D_2NaOSi$ $[M+Na]^+$: 259.1463; found: 259.1465.

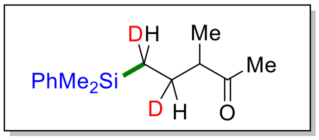

General Procedure for the Synthesis of γ-Silylated Ketone from 2,5-Dimethylfuran

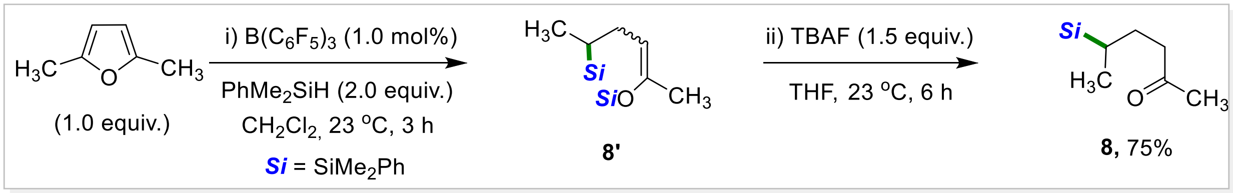

5-(Dimethylphenylsilyl)hexan-2-one (Table 2, 8, 75% over two steps). (*i*) Dimethylphenylsilane (2.726 g, 20.0 mmol) was added to a solution of $B(C_6F_5)_3$ (51.2 mg, 1.0 mol%) in CH_2Cl_2 (8.0 mL) at 23 °C in a 25 mL round bottom flask, and the solution was stirred for 2 min. 2,5-dimethylfuran (961 mg, 10 mmol) was then added into the solution at 23 °C and stirred for 3 h. The reaction mixture was quenched with trimethylamine (Et_3N, 1.0 mmol), concentrated under reduced pressure and the resulting residue containing **8'** was subjected to hydrolysis.

(*ii*) The above reaction mixture was diluted with anhydrous THF (5 mL) and tetrabutylammonium fluoride (TBAF, 1 M in THF, 15 mL, 15.0 mmol) was slowly added into the reaction mixture and stirred at 23 °C. After 6 h, the reaction mixture was purified by column chromatography on silica gel (ethyl acetate/hexane, 1/9) to give **8** (1.766 g, 75% over two steps) as colorless liquid; ^{1}H NMR (600 MHz, $CDCl_3$): δ 7.52–7.48 (m, 2H), 7.38–7.33 (m, 3H), 2.54–2.45 (m, 1H), 2.39–2.26 (m, 1H), 2.05 (s, 3H), 1.87–1.78 (m, 1H), 1.42–1.30 (m, 1H), 0.94 (d, J = 7.3 Hz, 3H), 0.89–0.80 (m, 1H), 0.29 (s, 3H), 0.28 (s, 3H); ^{13}C NMR (150 MHz, $CDCl_3$): δ 209.4, 138.3, 133.9, 128.9, 127.8, 42.9, 29.8, 25.9, 18.9, 13.9, -4.8, -5.1; ^{29}Si NMR (120 MHz, $CDCl_3$): δ -0.04; HRMS (ESI, m/z): calcd. for $C_{14}H_{22}NaOSi$ $[M+Na]^+$: 257.1338; found: 257.1325.

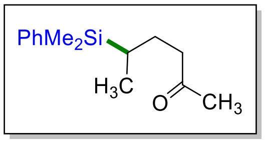

General Procedure for the Synthesis of *ortho*-(*β*-Silylethyl)-Phenols (GP6)

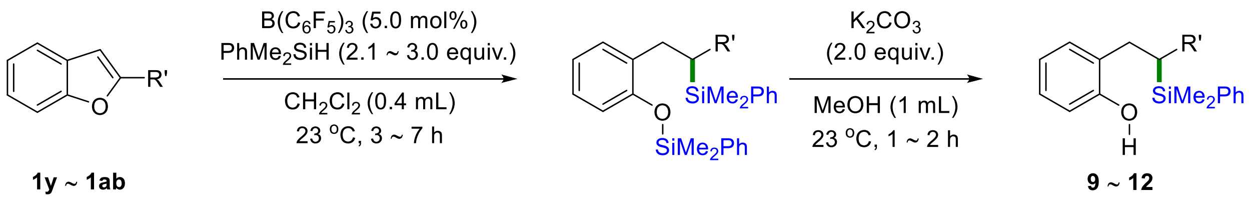

In a flame-dried flask, $B(C_6F_5)_3$ (0.025 mmol, 5.0 mol%) was dissolved in CH_2Cl_2 (0.4 mL), into which dimethylphenylsilane (1.05 ~ 1.5 mmol) was added, and the solution was shaken shortly to make it homogeneous. The corresponding benzofuran derivatives (**1y** ~ **1ab**, 0.5 mmol) were added and the reaction mixture was stirred at 23 °C for the indicated time (3 ~ 7 h) under argon atmosphere. The reaction mixture was then treated with K_2CO_3 (138 mg, 1.00 mmol) in MeOH (2 mL) at 23 °C for 1 ~ 2 h.

The crude reaction mixture was filtered through a small pad of Celite to remove salt and other impurities and the filtrate solution was concentrated under reduced pressure and then purified by flash column chromatography on silica gel (hexane/ethyl acetate) to afford products (**9** ~ **12**, the reported yields were for two steps).

Characterization Data of the Obtained *ortho*-(*β*-Silylethyl)-Phenols

2-[2-(Dimethylphenylsilyl)ethyl]phenol (Table 2, 9, Reaction time: 5 h for two steps, 90% over two steps**)**. Employed benzofuran (**1y**); The compound **9** was prepared according to **GP6** and was purified by flash column chromatography (hexane and ethyl acetate); colorless liquid; ^{1}H NMR (600 MHz, $CDCl_3$): δ 7.64–7.52 (m, 2H), 7.45–7.31 (m, 3H), 7.14 (d, J = 7.3 Hz, 1H), 7.07 (t, J = 8.3 Hz, 1H), 6.88 (t, J = 7.7 Hz, 1H), 6.73 (d, J = 7.9 Hz, 1H), 4.69 (s, 1H), 2.97–2.41 (m, 2H), 1.29–0.96 (m, 2H), 0.33 (s, 6H); ^{13}C NMR (150 MHz, $CDCl_3$): δ 153.3, 139.2, 133.7, 130.9, 129.4, 129.1, 127.9, 127.0, 120.9, 115.3, 24.2, 15.9, -2.9; ^{29}Si NMR (120 MHz, $CDCl_3$): δ -2.7; HRMS (EI, m/z): calcd. for $C_{16}H_{20}OSi$ $[M]^+$: 256.1283; found: 256.1286.

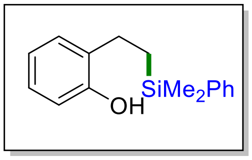

2-[2-(Dimethylphenylsilyl)propyl]phenol (Table 2, 10, Reaction time: 4 h, 88% over two steps**)**. Employed 2-methylbenzofuran (**1z**); The compound **10** was prepared according to **GP6** and was purified by flash column chromatography (hexane and ethyl acetate); colorless liquid; ^{1}H NMR (600 MHz, $CDCl_3$): δ 7.77–7.56 (m, 2H), 7.51–7.32 (m, 3H), 7.16–7.02 (m, 2H), 6.87 (t, J = 6.0 Hz, 1H), 6.75 (d, J = 6.0 Hz, 1H), 4.60 (s, 1H), 2.88 (dd, J = 12.0, 6.0 Hz, 1H), 2.19 (d, J = 12.0 Hz, 1H), 1.56–1.21 (m, 1H), 0.94 (d, J = 7.3 Hz, 3H), 0.37 (s, 6H); ^{13}C NMR (150 MHz, $CDCl_3$): δ 153.7, 138.4, 134.1, 130.9, 129.1, 127.9, 127.1, 120.5, 115.4, 32.0, 20.3, 13.9, -4.7, -5.2; ^{29}Si NMR (120 MHz, $CDCl_3$): δ 0.55; HRMS (EI, m/z): calcd. for $C_{17}H_{22}OSi$ $[M]^+$: 270.1440; found: 270.1440.

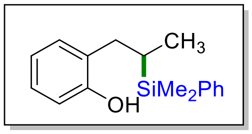

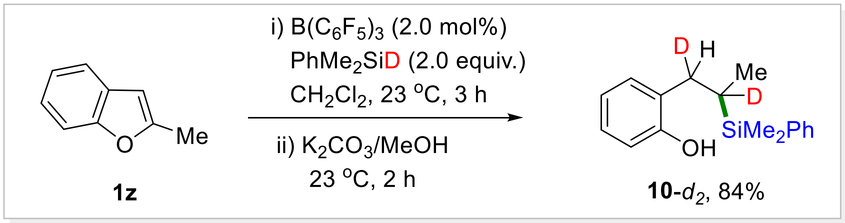

2-[2-(Dimethylphenylsilyl)propyl]phenol-*d*$_2$ (Table 2, 10-*d*$_2$, Reaction time: 5 h, 84% over two steps**):** The compound **10-*d*$_2$** was prepared according to **GP6** with $PhMe_2SiD$ in presence of 2.0 mol% of $B(C_6F_5)_3$ and was purified by flash column chromatography (hexane and ethyl acetate); colorless liquid; ^{1}H NMR (600 MHz, $CDCl_3$): δ 7.69–7.54 (m,

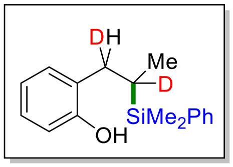

2H), 7.54–7.66 (m, 3H), 7.13–7.00 (m, 2H), 6.87 (t, 1H), 6.74 (d, J = 8.4 Hz, 1H), 4.57 (s, 1H), 2.26 (s, 1H), 0.93 (s, 3H), 0.37 (s, 6H); ^{13}C NMR (150 MHz, CDCl3): δ 153.7, 138.4, 134.1, 131.0, 129.1, 128.2, 127.9, 127.1, 120.6, 115.4, 31.6 (t, J = 19.6 Hz), 19.7 (t, J = 19.6 Hz), 13.8, -4.7, -5.2; ^{29}Si NMR (120 MHz, $CDCl_3$): δ 0.54; ^{2}H NMR (60 MHz, $CDCl_3$) δ 2.76 (s, 1D), 1.16 (s, 1D); HRMS (EI, m/z): calcd. for for $C_{17}H_{20}D_2OSi$ $[M]^+$: 272.1565; found: 272.1563.

2-{2-(Dimethylphenylsilyl)-2-[4-(triisopropylsilyloxy)phenyl]ethyl}phenol (Table 2, 11, Reaction time: 9 h for two steps, 70% over two steps**)**. Employed 2-(naphthalen-1-yl)benzofuran (**1aa**); The compound **11** was prepared according to **GP6** and was purified by flash column chromatography (hexane and ethyl acetate); colorless liquid; ^{1}H NMR (600 MHz, $CDCl_3$): δ 7.53–7.44 (m, 2H), 7.41–7.31 (m, 3H), 6.98–6.89 (m, 1H), 6.82–6.75 (m, 3H), 6.74–6.68 (m, 2H), 6.66–6.56 (m, 2H), 4.38 (s, 1H), 3.07 (dd, J = 14.7, 3.2 Hz, 1H), 2.96–2.85 (m, 1H), 2.49 (dd, J = 11.7, 3.2 Hz, 1H), 1.27–1.18 (m, 3H), 1.14–0.89 (m, 18H), 0.34 (s, 3H), 0.24 (s, 3H); ^{13}C NMR (150 MHz, $CDCl_3$): δ 153.5, 137.7, 135.1, 134.3, 130.5, 129.4, 129.3, 129.1, 128.5, 127.8, 127.0, 120.6, 120.1, 115.6, 36.5, 31.0, 18.0, 12.7, -3.8, -4.9; ^{29}Si NMR (120 MHz, $CDCl_3$): δ 15.2, -1.8; HRMS (EI, m/z): calcd. for $C_{31}H_{44}O_2Si_2$ $[M]^+$: 504.2880; found: 504.2879.

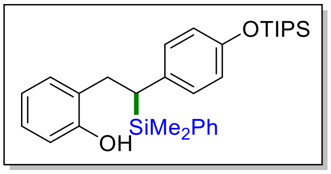

2-[2-(Dimethylphenylsilyl)-2-phenylethyl]phenol (Table 2, 12, Reaction time: 6 h for two steps, 79% over two steps**)**. Employed 2-phenylbenzofuran (**1ab**); The compound **12** was prepared according to **GP6** and was purified by flash column chromatography (hexane and ethyl acetate); colorless liquid; ^{1}H NMR (600 MHz, $CDCl_3$): δ 7.57–7.49 (m, 2H), 7.47–7.32 (m, 3H), 7.19 (t, J = 6.7 Hz, 2H), 7.09 (t, J = 7.3 Hz, 1H), 7.04–7.90 (m, 3H), 6.85 (d, J = 7.4 Hz, 1H), 6.69 (t, J = 6.9 Hz, 1H), 6.62 (d, J = 7.9 Hz, 1H), 4.47 (s, 1H), 3.11 (d, J = 14.4 Hz, 1H), 3.03 (t, J = 13.0 Hz, 1H), 2.66 (d, J = 10.6 Hz, 1H), 0.37 (s, 3H), 0.26 (s, 3H); ^{13}C NMR (150 MHz, $CDCl_3$): δ 153.5, 142.7, 137.5, 134.3, 130.4, 129.3, 128.3, 128.3, 128.2, 127.9, 127.1, 125.0, 120.6, 115.5, 37.2, 30.5, -3.7, -5.1; ^{29}Si NMR (120 MHz, $CDCl_3$): δ -1.3; HRMS (EI, m/z): calcd. for $C_{22}H_{24}OSi$ $[M]^+$: 332.1596; found: 332.159.

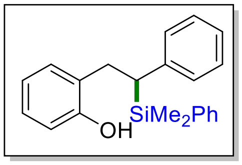

Preliminary Mechanistic Experiments.

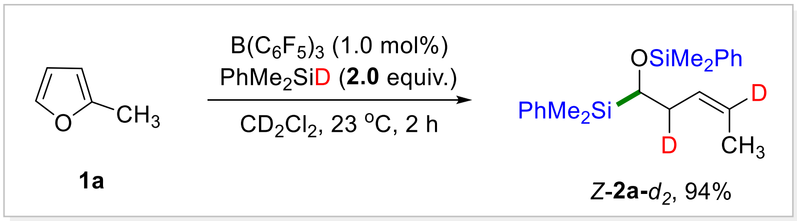

(*Z*)-[1-(Dimethylphenylsilyloxy)pent-3-en-1-yl]dimethylphenylsilane-*d*$_2$ (Fig. 3B, *Z*-2a-*d*$_2$, Reaction

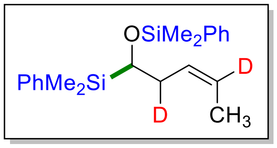

time: 2 h, *Z/E* >99/1, 94%**)**. Employed 2-methylfuran (**1a**); The compound **10** was prepared according to **GP2** with PhMe$_2$SiD and was purified by flash column chromatography (hexane and ethyl acetate); colorless liquid; ^{1}H NMR (600 MHz, CDCl$_3$): δ 7.50–7.56 (m, 4H), 7.40–7.30 (m, 6H), 5.33 (d, J = 7.5 Hz, 1H), 3.66 (d, J = 7.4 Hz, 1H), 2.28 (s, 1H), 1.47 (s, 3H), 0.31 (s, 6H), 0.28 (s, 3H), 0.26 (s, 3H); ^{13}C NMR (150 MHz, CDCl$_3$): δ 138.8, 137.8, 134.4, 133.8, 129.4, 129.1, 128.2, 127.7, 124.8 (t, J = 24.2 Hz), 66.87, 31.6 (t, J = 19.6 Hz), 12.9, -0.8, -4.7; ^{29}Si NMR (120 MHz, CDCl$_3$): δ 5.9, -4.2; ^{2}H NMR (60 MHz, CDCl$_3$): δ 5.50 (s, 1D), 2.34 (s, 1D); HRMS (EI, m/z): calcd. for $C_{21}H_{28}D_2OSi_2$ $[M]^+$: 356.1961; found: 356.1957.

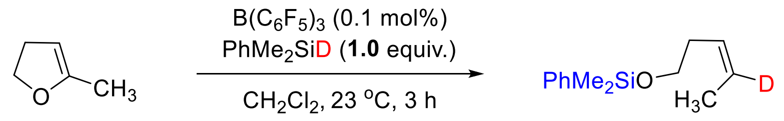

13 *Z*-**14**-*d*, 78%

@ -40 °C, 1.0 mol% B(C$_6$F$_5$)$_3$, 1 h: 86%

(*Z*)-(Pent-3-en-1-yloxy)dimethylphenylsilane-*d* (Fig. 3C, *Z*-**14**-***d***, Reaction time: 3 h, 78% @ 23 °C;

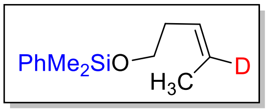

Reaction time: 1 h, 86% @ -40 °C**:** *Z/E* >99/1**)**. In both cases 2,3-dihydro-5-methylfuran (**13**, 2.0 equiv.) and and PhMe$_2$SiD (1.0 equiv.) were used; colorless liquid; ^{1}H NMR (600 MHz, CDCl$_3$): δ 7.62 (d, J = 7.1 Hz, 2H), 7.41 (d, J = 6.2 Hz, 3H), 5.39 (s, 1H), 3.95–3.36 (m, 2H), 2.50–2.11 (m, 2H), 1.61 (s, 3H), 0.42 (s, 6H); ^{13}C NMR (150 MHz, CDCl$_3$): δ 138.1, 133.6, 129.7, 127.9, 126.3, 125.9, 125.7, 125.6, 62.8, 30.6, 12.9, -1.6; ^{29}Si NMR (120 MHz, CDCl$_3$): δ 7.3; ^{2}H NMR (60 MHz, CDCl$_3$): δ 5.61 (s, 1D); HRMS (EI, m/z): calcd. for $C_{13}H_{19}DOSi$ $[M]^+$: 221.1346; found: 221.1344.

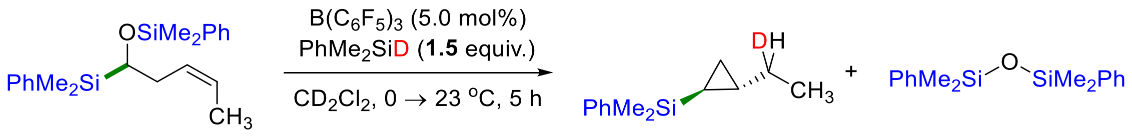

Z-**2a** *anti*-**3a**-*d*, 66% (82%) **4**

anti*-(2-Ethylcyclopropyl)dimethylphenylsilane-*d* (Fig. 3D**, *anti*-**3a**-*d,** Reaction time: 5 h, 82% NMR

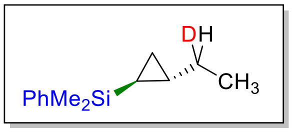

yield, 66% isolated yield**)**. Employed *Z*-**2a**; colorless liquid; ^{1}H NMR (600 MHz, CD$_2$Cl$_2$): δ 7.66–7.59 (m, 2H), 7.43–7.35 (m, 3H), 1.45–1.40 (m, 0.6H), 1.30–1.21 (m, 0.4H), 1.02 (d, J = 7.5 Hz, 3H), 0.75–0.67 (m, 1H), 0.51–0.42 (m, 2H), 0.26 (s, 3H), 0.24 (s, 3H), -0.33– -0.46 (m, 1H); ^{13}C NMR (150 MHz, CD$_2$Cl$_2$): δ 140.1, 134.3,

129.2, 128.1, 29.0 (t = 19.6 Hz), 18.1, 14.1, 9.2, 3.6, -3.4, -3.7; ^{29}Si NMR (120 MHz, CD_2Cl_2): δ -3.1; ^{2}H NMR (60 MHz, CD_2Cl_2): δ 1.58 – 1.36 (s, 0.4D), 1.36 – 1.12 (s, 0.6D); HRMS (ESI, m/z): calcd. for $C_{13}H_{20}DSi$ $[M+H]^+$: 206.1475; found: 206.1428.

Synthetic Applications

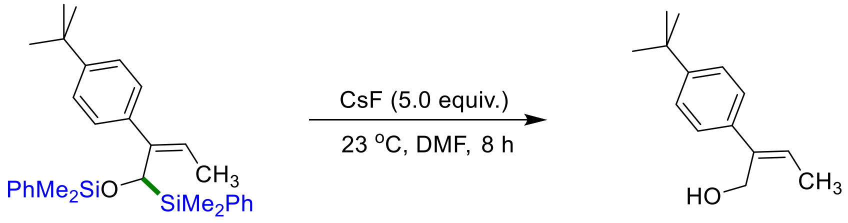

Z-**6** *Z*-**15**, 65%

(*Z*)-2-[4-(*tert*-Butyl)phenyl]but-2-en-1-ol (Fig. 4, *Z*-**15**, Reaction time: 8 h, 65%**)**. A round bottom flask

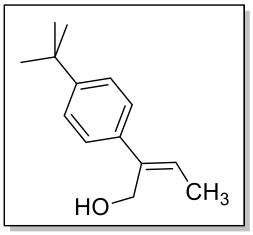

was charged with (*Z*)-[2-(4-*tert*-butyl-phenyl)-1-(dimethylphenylsilyloxy)but-2-en-1-yl]dimethyl phenylsilane (*Z*-**6**, 0.5 mmol, 1.0 equiv.) and CsF (2.5 mmol, 1.0 equiv), into which DMF (1.0 mL) was subsequently added. Then, the reaction mixture was at 23 °C until a TLC analysis of an aliquot indicated a complete conversion of the starting materials (8 h), after which it was poured into H_2O (5 mL). The aqueous phase was back-extracted with diethylether (10 mL x 3). The combined organic layers were dried over Na_2SO_4, evaporated under reduced pressure, and purified by flash column chromatography on silica gel (ethyl acetate/hexane, 1/2) to give the corresponding product (*Z*-**15**, 65%); colorless liquid; ^{1}H NMR (600 MHz, $CDCl_3$): δ 7.41–7.34 (m, 4H), 5.96 (q, J = 7.1, Hz, 1H), 4.59 (d, J = 2.7 Hz, 2H), 2.17 (brs, 1H), 1.89 (d, J = 7.0, 3H), 1.32 (s, 9H); ^{13}C NMR (150 MHz, $CDCl_3$): δ 150.1, 139.4, 138.0, 126.1, 125.9, 125.6, 59.6, 34.6, 31.5, 14.2; HRMS (EI, m/z): Calcd. for $C_{14}H_{20}O$ $[M]^+$: 204.1514; found: 204.1511.

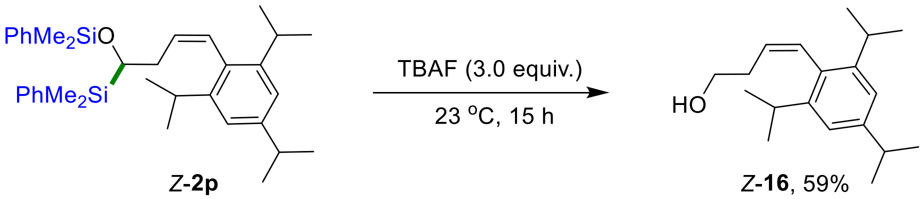

Z-**2p** *Z*-**16**, 59%

(*Z*)-4-(2,4,6-triisopropylphenyl)but-3-en-1-ol (Fig. 4, *Z*-**16**, Reaction time: 15 h, 59%**)**. A round bottom

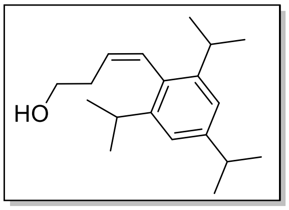

flask was charged with (*Z*)-1-[(dimethylphenylsilyloxy)-4-(2,4,6-triisopropylphenyl)but-3-en-1-yl]dimethylPhenylsilane (*Z*-**2p**, 0.4 g, 0.737 mmol) and tetrabutylammonium fluoride (TBAF, 2.2 mL, 2.2 mmol, 1 M in

THF) at 0 °C. Then, the reaction mixture was stirred at 23 °C. After 15 h, the reaction mixture was purified by column chromatography on silica gel (ethyl acetate/hexane, 1/2) to give (*Z*-**16**, 59%) as colorless liquid; ^{1}H NMR (600 MHz, $CDCl_3$): δ 6.98 (s, 2H), 6.48 (d, J = 12 Hz, 1H), 5.89–5.81 (m, 1H), 3.64 (q, J = 6 Hz, 2H), 3.21–3.07 (m, 2H), 2.96–2.83 (m, 1H), 2.22–2.08 (q, J = 6.0 Hz, 2H), 1.27 (d, J = 6 Hz, 6H), 1.24–1.20 (m, 1H), 1.19 (d, J = 6.0 Hz, 6H), 1.13 (d, J = 5.4 Hz, 6H); ^{13}C NMR (150 MHz, $CDCl_3$): δ 147.7, 146.3, 131.4, 129.9, 129.2, 120.5, 62.4, 34.4, 32.5, 30.3, 24.3, 24.2, 23.5; HRMS (EI, m/z): calcd. for $C_{19}H_{30}O$ $[M]^+$: 274.2297; found: 274.2294.

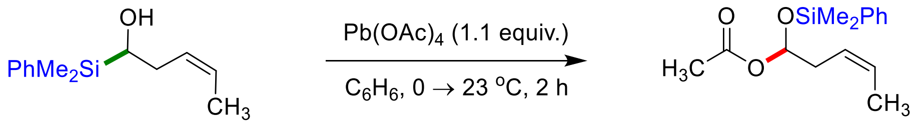

Z-**2a'** *Z*-**17**, 75%

(*Z*)-1-[(Dimethylphenylsilyl)oxy]pent-3-en-1-yl-acetate (Fig. 4, *Z*-**17**, Reaction time: 2 h, 75%**)**. To a

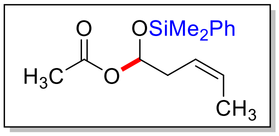

stirred solution of (*Z*)-1-(dimethylphenylsilyl)pent-3-en-1-ol (*Z*-**2a'**, 0.22 g, 1.0 mmol) in degassed benzene (14 mL) in a light protected vessel, $Pb(OAc)_4$ (0.488 g, 1.1 mmol) was added at 0 °C under N_2 atmosphere. Then, the reaction mixture was stirred at 23 °C. After 2 h, the suspension was filtered and the solid was washed with ethyl acetate. The resulting filtrate was concentrated under reduced pressure, and purified by flash column chromatography on silica gel (ethyl acetate/hexane, 1/9) to give the corresponding product (*Z*-**17)** obtained as colorless liquid (0.209 g, 75%); ^{1}H NMR (600 MHz, $CDCl_3$): δ 7.59 (d, J = 1.6 Hz, 1H), 7.58 (d, J = 1.7 Hz, 1H), 7.40–7.34 (m, 3H), 6.00 (t, J = 5.3 Hz, 1H), 5.64–5.54 (m, 1H), 5.40–5.30 (m, 1H), 2.48–2.34 (m, 2H), 1.91 (s, 3H), 1.58 (d, J = 6.9 Hz, 3H), 0.44 (s, 3H), 0.43 (s, 3H); ^{13}C NMR (150 MHz, $CDCl_3$): δ 169.9, 137.0, 133.5, 129.7, 127.7, 127.5, 123.2, 92.1, 34.4, 21.1, 12.9, -1.4; ^{29}Si NMR (120 MHz, $CDCl_3$): δ 8.8; IR (neat, cm^{-1}): 2958, 1737, 1590, 1427, 1370, 1238, 1117, 1007, 935, 823, 699; HRMS (ESI, m/z): calcd. for $C_{15}H_{22}NaO_3Si$ $[M+Na]^+$: 301.1236; found: 301.1230.[8]

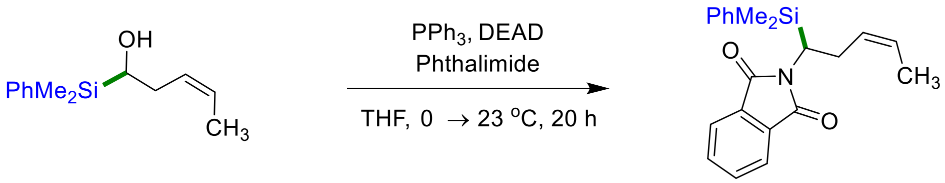

Z-**2a'** *Z*-**18**, 76%

(*Z*)-2-[1-(Dimethylphenylsilyl)pent-3-en-1-yl]isoindoline-1,3-dione (Fig. 4, ***Z*-18,** Reaction time: 20 h, 76%**)**.

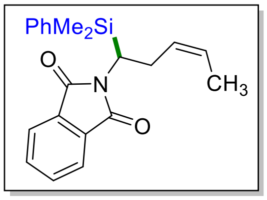

To a mixture solution of (*Z*)-1-(dimethylphenylsilyl)pent-3-en-1-ol (*Z*-**2a'**, 0.22 g, 1.0 mmol), triphenylphosphine (TPP, 0.34 g, 1.3 mmol), and phthalimide (0.191 g, 1.3 mmol) in THF (2.0 mL) at 0 °C was slowly added diethyl azodicarboxylate (DEAD, 0.226 g, 1.3 mmol) under nitrogen atmosphere. Then, the reaction mixture was stirred at 23 °C. After 20 h, the crude reaction mixture was concentrated under reduced pressure, and purified by flash column chromatography on silica gel (ethyl acetate/hexane, 1/9) to give the corresponding product *Z*-**18** obtained as colorless viscous oil (0.265 g, 76%). ^{1}H NMR (400 MHz, $CDCl_3$): δ 7.79–7.72 (m, 2H), 7.68–7.61 (m, 2H), 7.60–7.53 (m, 2H), 7.35–7.29 (m, 3H), 5.49–5.34 (m, 1H), 5.34–5.18 (m, 1H), 4.03 (dd, J = 11.6, 4.5 Hz, 1H), 2.99–2.83 (m, 1H), 2.41–2.25 (m, 1H), 1.6 (d, J = 6.7 Hz, 3H), 0.59 (s, 3H), 0.45 (s, 3H); ^{13}C NMR (100 MHz, $CDCl_3$): δ 168.9, 136.8, 133.8, 133.6, 131.9, 129.3, 127.8, 127.5, 126.6, 122.8, 41.1, 26.0, 12.6, -3.3, -3.8; ^{29}Si NMR (80 MHz, $CDCl_3$): δ -0.3; IR (neat, cm^{-1}): 2954, 1770, 1705, 1466, 1427, 1385, 1249, 1172, 1078, 985, 882, 717; HRMS (ESI, m/z): calcd. for $C_{21}H_{23}NNaO_2Si$ $[M+Na]^+$: 372.1396; found: 372.1383.[9]

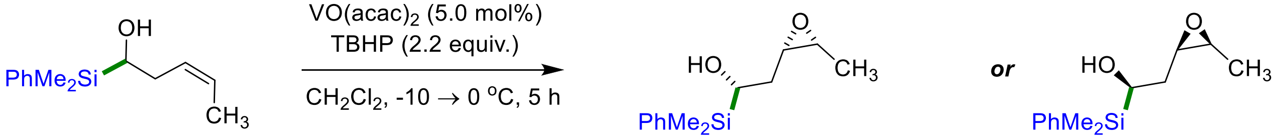

Z-**2a'**

syn-**19**, 72% (single isomer)

1-[(Dimethylphenylsilyl)-2-(3-methyloxiran-2-yl)]ethan-1-ol (Fig. 4, *syn*-**19,** Reaction time: 5 h, 72%**)**.

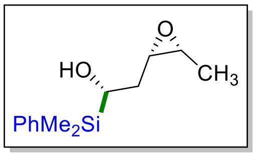

The compound (*Z*)-1-(dimethylphenylsilyl)pent-3-en-1-ol (*Z*-**2a'**, 0.22 g, 1.0 mmol) was dissolved in CH_2Cl_2 (14 mL) and cooled down to -10 °C, into which $VO(acac)_2$ (0.026 g, 5 mol%) was added, followed by *tert*-butylhydroperoxide (TBHP, 6.3 M in decanes, 0.35 mL, 2.2 mmol) under N_2 atmosphere. The resulting reaction mixture was then stirred for 5 h at 0 °C, after which it was poured into ethyl acetate (5 mL), washed with 10% aqueous $Na_2S_2O_3$ and brine. The aqueous phase was back-extracted once with ethyl acetate. The combined organic layers were dried over Na_2SO_4, evaporated under reduced pressure, and purified by flash column chromatography on silica gel (ethyl acetate/hexane, 2/8) to give the corresponding product *syn*-**19** obtained as single diastereomer (colorless oil, 0.170 g, 72%). ^{1}H NMR (400 MHz, $CDCl_3$): δ 7.64–7.54 (m, 2H), 7.44–7.35 (m, 3H), 3.85 (dd, J = 10.8, 2.9 Hz, 1H), 3.18–3.09 (m, 1H), 3.08–2.97 (m, 1H), 2.49 (brs, 1H), 1.85–1.61 (m, 2H), 1.25 (d, J = 5.5 Hz, 3H), 0.41 (s, 3H), 0.40 (s, 3H); ^{13}C NMR (100 MHz, $CDCl_3$): δ 136.3, 134.1, 129.4, 127.9, 64.6, 57.2, 51.7, 30.6, 13.3, -5.6, -5.8; ^{29}Si NMR (80 MHz, $CDCl_3$): δ -4.1; IR (neat, cm^{-1}): 3443, 2956, 1717, 1450, 1389, 1247, 1140, 983, 812, 699; HRMS (ESI, m/z):

calcd. for $C_{13}H_{20}NaO_2Si$ $[M+Na]^+$: 259.1130; found: 259.1138.[10]

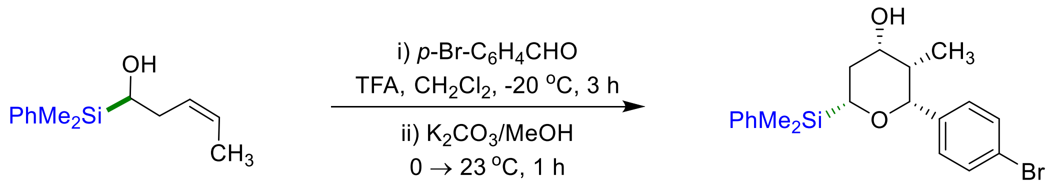

Z-**2a'**

syn-**20**, 57% for two steps
(single isomer)

2-[(4-Bromophenyl)-6-(dimethylphenylsilyl)]-3-methyltetrahydro-2*H*-pyran-4-ol (Fig. 4, *syn*-20,

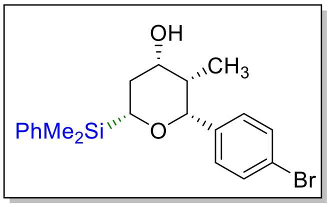

Reaction time: 4 h for two steps, 57% over two steps**)**. Trifluoroacetic acid (0.285 g, 2.25 mmol) was added slowly to a solution of the (*Z*)-1-(dimethylphenylsilyl)pent-3-en-1-ol (*Z*-**2a'**, 0.22 g, 1.0 mmol) and 4-bromobenzaldehyde (0.221 g, 1.2 mmol) in CH_2Cl_2 (5 mL) at -20 °C under nitrogen atmosphere. The resulting mixture was stirred for 3 h at the same temperature and then quenched with saturated sodium hydrogen carbonate solution (5 mL) and the pH was adjusted to >7 by addition of triethylamine. The layers were separated and the aqueous layer was extracted with CH_2Cl_2 (5 mL x 3). The combined organic layers were dried over anhydrous Na_2SO_4 and the solvent was removed under reduced pressure. The residue was dissolved in MeOH (6 mL) and cooled down to 0 °C, into which K_2CO_3 (0.276 g, 2.0 mmol) was added and stirred at 23 °C. Completion of the reaction was confirmed by the TLC analysis (2 h). The suspension was filtered through a small pad of Celite and the solid was washed with ethyl acetate. The resulting filtrate was concentrated under reduced pressure and purified by flash column chromatography on silica gel (ethyl acetate/hexane, 3/7) to give the desired product *syn*-**20** (0.230 g, 57% for two steps) as colorless liquid of a single diastereomer. ^1H NMR (600 MHz, $CDCl_3$): δ 7.69–7.58 (m, 2H), 7.47 (d, J = 4.9 Hz, 2H), 7.42–7.33 (m, 3H), 7.17 (d, J = 5.1 Hz, 2H), 4.41 (d, J = 3.4 Hz, 1H), 4.07–4.00 (m, 1H), 3.46 (d, J = 13.2 Hz, 1H), 2.21–2.12 (m, 1H), 1.80–1.71 (m, 1H), 1.67 (q, J = 13.0 Hz, 1H), 1.60–1.52 (m, 1H), 0.57 (d, J = 3.5 Hz, 3H), 0.42 (s, 6H); ^{13}C NMR (150 MHz, $CDCl_3$): δ 140.8, 136.4, 134.1, 131.1, 129.3, 127.8, 127.1, 120.2, 81.8, 72.2, 70.0, 40.7, 30.0, 4.9, -5.4, -5.6; ^{29}Si NMR (120 MHz, $CDCl_3$): δ -4.7; IR (neat, cm^{-1}): 3369, 2968, 1707, 1589, 1488, 1361, 1247, 1114, 1009, 817, 699; HRMS (ESI, m/z): calcd. for $C_{20}H_{25}BrNaO_2Si$ $[M+Na]^+$: 427.0705; found: 427.0688.[11,12]

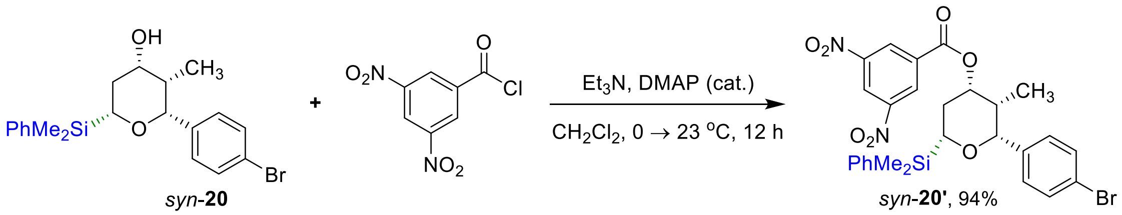

2-[(4-Bromophenyl)-6-(dimethylphenylsilyl)]-3-methyltetrahydro-2*H*-pyran-4-yl-3,5-dinitrobenzoate

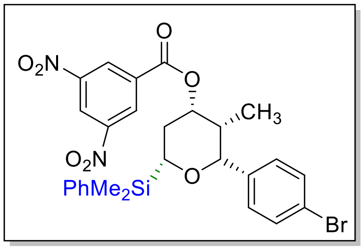

(Fig. 4, *syn*-20', **Reaction time: 12 h, 94%). ***O*-benzoylation: The compound *syn*-**20** (40.4 mg, 0.1 mmol) was diluted with CH_2Cl_2 (2 mL) and cooled down to 0 °C. Then, 3,5-dinitrobenzoyl chloride (28 mg, 0.12 mmol) was slowly added into the reaction mixture in the presence of triethylamine (51 mg, 0.3 mmol,) and a catalytic amount of 4-dimethylaminopyridine (DMAP, 0.1 mg, 0.001 mmol, 1 mol %). Then, the reaction mixture was allowed to react at 23 °C. After 12 h, the reaction mixture was quenched by adding water (3 mL) and the reaction mixture was extracted with CH_2Cl_2 (3 mL x 3). The combined organic layers were dried over Na_2SO_4, evaporated under reduced pressure, and purified by flash column chromatography on silica gel (ethyl acetate/hexane, 2/8) to give *syn*-**20'** (56 mg, 94%) as a single diastereomer. X-ray quality crystals of **syn-20'** were grown by slow evaporation in a mixture of ethyl acetate and hexane. Yellow color crystals; m.p. 145 – 147 °C; ^{1}H NMR (600 MHz, $CDCl_3$): δ 9.21 (t, J = 2.1 Hz, 1H), 9.11 (d, J = 2.1 Hz, 2H), 7.67–7.55 (m, 2H), 7.47 (d, J = 8.5 Hz, 2H), 7.43–7.33 (m, 3H), 7.18 (d, J = 8.1 Hz, 2H), 5.56–5.46 (m, 1H), 4.59 (d, J = 1.4 Hz, 1H), 3.60 (dd, J = 13.3, 2.4 Hz, 1H), 2.50–2.44 (m, 1H), 1.99 (td, J = 13.0, 11.6 Hz, 1H), 1.79–1.71 (m, 1H), 0.70 (d, J = 6.9 Hz, 3H), 0.45 (s, 3H), 0.44 (s, 3H); ^{13}C NMR (150 MHz, $CDCl_3$): δ 161.7, 148.6, 139.7, 135.8, 134.1, 134.06, 131.2, 129.5, 129.3, 127.9, 127.0, 122.4, 120.6, 81.6, 77.6, 70.0, 37.9, 26.7, 6.0, -5.4, -5.6; ^{29}Si NMR (120 MHz, $CDCl_3$): δ -4.2; IR (neat, cm^{-1}): 3099, 1728, 1628, 1546, 1487, 1343, 1277, 1168, 1047, 906, 783; HRMS (ESI, m/z): calcd. for $C_{27}H_{27}BrN_2NaO_7Si$ $[M+Na]^+$: 621.0669; found: 621.0684.

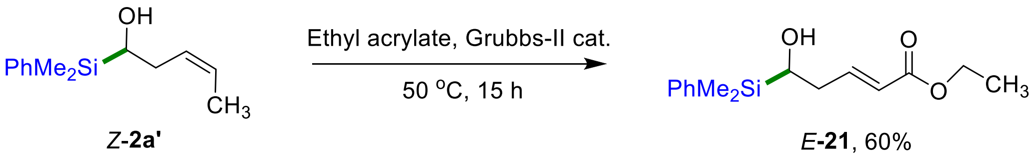

(*E*)-Ethyl-5-(dimethylphenylsilyl)-5-hydroxypent-2-enoate (Fig. 4, *E*-21, Reaction time: 15 h, 60%).

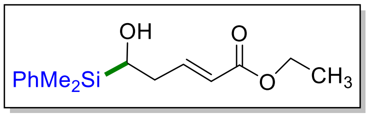

To a stirred solution of *Z*-**2a'** (0.22 g, *Z*/*E* >99/1, 1.0 mmol) in CH_2Cl_2 (10 mL) in a dried round bottom flask (25 mL), Grubbs 2nd generation catalyst (42.4 mg, 0.05 mmol), and ethyl acrylate (1.0 g, 10.0 mmol) were added. The resulting mixture was then refluxed under N_2 atmosphere (oil bath, 50 °C) for 15 h. The mixture was filtered through a small pad of Celite, washed with CH_2Cl_2. The solvents were removed under reduced pressure and the resulting mixture was purified by flash column chromatography on silica gel (hexane/ethyl acetate, 9/1) to give the corresponding product *E*-**21** as colorless oil (0.167 g, *E*/*Z* >99/1, 60%). ^{1}H NMR (400 MHz, $CDCl_3$): δ 7.63–7.53 (m, 2H), 7.47–7.34 (m, 3H), 7.03–6.92 (m, 1H),

5.88 (dt, J = 15.6, 1.4 Hz, 1H), 4.19 (q, J = 7.1 Hz, 2H), 3.64 (dd, J = 10.0, 4.0 Hz, 1H), 2.48–2.29 (m, 2H), 1.69 (brs, 1H), 1.29 (t, J = 7.1 Hz, 3H), 0.40 (s, 3H), 0.39 (s, 3H); ^{13}C NMR (100 MHz, $CDCl_3$): δ 166.3, 146.8, 136.0, 134.1, 129.5, 128.0, 123.3, 63.6, 60.2, 36.4, 14.2, -5.4, -5.5; ^{29}Si NMR (80 MHz, $CDCl_3$): δ -3.4; IR (neat, cm^{-1}): 3465, 2978, 1715, 1650, 1465, 1368, 1249, 1154, 1037, 977, 812, 699; HRMS (ESI, m/z): calcd. for $C_{15}H_{22}NaO_3Si$ $[M+Na]^+$: 301.1236; found: 301.1238.[13]

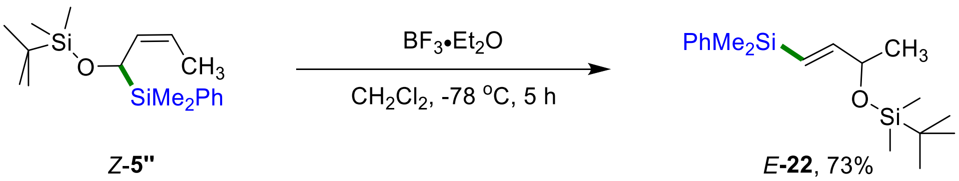

(*E*)-*tert*-Butyl{[4-(dimethylphenylsilyl)but-3-en-2-yl]oxy}dimethylsilane (Fig. 4, *E*-22, Reaction time: 5 h, 73%**)**. To a cooled solution (-78 °C) of *Z*-**5''** (*Z*/*E* >99/1, 0.16 g, 0.5 mmol) in CH_2Cl_2 (5 mL, 0.1 M) was added BF_3•OEt_2 (1.1 equiv.) under N_2 atmosphere. The resulting mixture was stirred at -78 °C for 5 h, into which then an aqueous solution of $NaHCO_3$ was added at 25 °C for quenching the reaction. The two layers were separated and the aqueous layer was extracted with CH_2Cl_2. The combined organic layers were dried over anhydrous Na_2SO_4 and the solvent was removed under reduced pressure. The resulting crude product was purified by flash column chromatography on silica gel (hexane/ethyl acetate, 9/1) afforded the *E*-**22** as colorless oil (0.117 g, *E*/*Z* >99/1, 73%). ^{1}H NMR (600 MHz, $CDCl_3$): δ 7.58–7.50 (m, 2H), 7.40–7.34 (m, 3H), 6.19–6.09 (m, 1H), 5.98 (d, J = 12 Hz, 1H), 4.37–4.30 (m, 1H), 1.25 (d, J = 6.5 Hz, 3H), 0.94 (s, 9H), 0.36 (s, 6H), 0.09 (s, 6H); ^{13}C NMR (150 MHz, $CDCl_3$): δ 152.2, 138.9, 133.8, 128.8, 127.7, 124.6, 71.2, 25.9, 24.0, 18.4, -2.4, -2.5, -4.5, -4.7; ^{29}Si NMR (120 MHz, $CDCl_3$): δ 18.1, -11.1; IR (neat, cm^{-1}): 2955, 1619, 1462, 1312, 1248, 1197, 1093, 984, 823, 697; HRMS (ESI, m/z): calcd. for $C_{18}H_{32}NaOSi_2$ $[M+Na]^+$: 343.1889; found: 343.1845.[14]

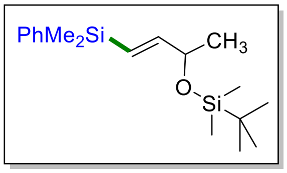

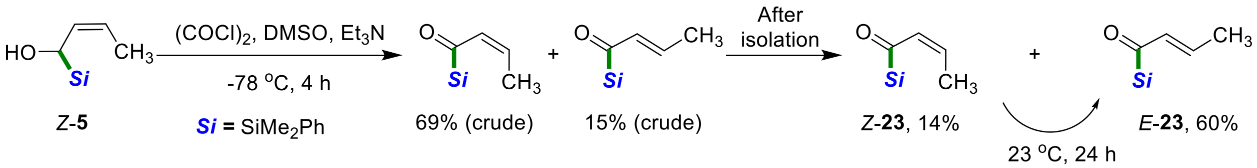

(*E*)-1-(Dimethylphenylsilyl)but-2-en-1-one (Fig. 4, *E*-**23)**. To a stirred solution of oxalyl chloride (0.127 g, 1.0 mmol) in CH_2Cl_2 (2.0 mL) at -78 °C, dried DMSO (0.156 g, 2.0 mmol) in CH_2Cl_2 (1 mL) was added dropwise. After 30 min (*Z*)-1-(dimethylphenylsilyl)but-2-en-1-ol (*Z*-**5**, 0.103 g, 0.5 mmol, *Z*/*E* >99/1), in CH_2Cl_2 (1.0 mL) was added dropwise over 5 min. After 30 min, trimethylamine (0.252 g, 2.5 mmol) was added slowly at -78 °C over 30 min, and

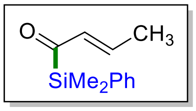

then the reaction mixture was allowed to stir at 23 °C for 30 min. This crude reaction mixture was quenched with water. The layers were separated and the aqueous layer was extracted with CH_2Cl_2 and the combined organic layers were dried over anhydrous Na_2SO_4 and the solvent was removed under reduced pressure and 1,1,2,2-tetrachloroethane (TCE, 84 mg, 0.5 mmol) was added as an internal standard. The resulting crude reaction mixture was subjected to ^{1}H NMR spectroscopy. The crude NMR yields and isomer ratio were calculated on the basis of an internal standard. The resulting crude product was purified by flash column chromatography on silica gel (hexane/ethyl acetate, 9/1) afforded *Z*-**23** (14.3 mg, 14%) and *E*-**23** (61 mg, 60%). *Z*-**23** was further converted to thermodynamically stable *E*-**23** (*E*/*Z* >99/1) in 24 h at 23 °C. Yellow color oil; ^{1}H NMR (600 MHz, $CDCl_3$): δ 7.60–7.49 (m, 2H), 7.44–7.31 (m, 3H), 6.71-6.59 (m, 1H), 6.24 (dd, J = 16.2, 2.3 Hz, 1H), 1.81 (d, J = 6.4 Hz, 3H), 0.51 (s, 6H); ^{13}C NMR (150 MHz, $CDCl_3$): δ 234.1, 144.6, 137.9, 135.5, 133.9, 129.7, 128.1, 18.6, -3.5; ^{29}Si NMR (120 MHz, $CDCl_3$): δ -15.55; IR (neat, cm^{-1}): 2956, 1699, 1591, 1426, 1251, 1117, 1041, 827, 785, 697; HRMS (FAB, m/z): calcd. for $C_{12}H_{17}OSi$ $[M+H]^+$: 205.1049; found: 205.1050.[15]

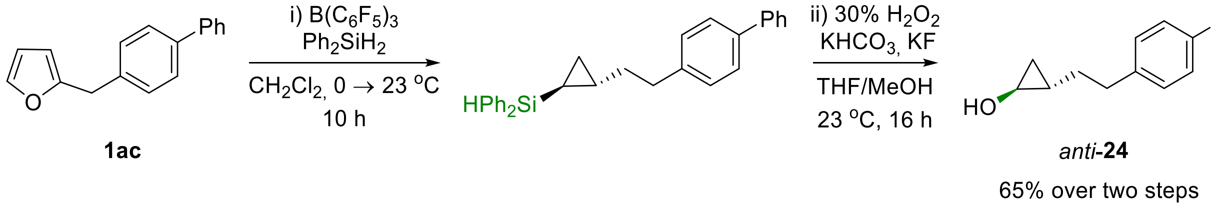

***anti*-2-{2-[(1,1'-Biphenyl)-4-yl]ethyl}cyclopropanol (Fig. 4**, *anti*-**24**, Reaction time: 26 h for two steps, 65% over two steps**)**. Employed 2-[(1,1'-biphenyl)-4-ylmethyl]furan (**1ac**, 937 mg, 4.0 mmol) with Ph_2SiH_2 (2.21 g, 12.0 mmol) using $B(C_6F_5)_3$ (102.4 mg, 5.0 mol%) in CH_2Cl_2 (1.0 mL) at 0 to 23 °C. After completion, the reaction mixture was concentrated under reduced pressure and the resulting residue was directly subjected to Tamao oxidation. The residue was dissolved in THF/MeOH (20/20 mL), into which potassium fluoride (KF, 2.32 g, 40 mmol), potassium bicarbonate ($KHCO_3$, 4.04 g, 40 mmol), and 30% hydrogen peroxide in water (16 mL, 130 mmol) were added and stirred at 23 °C for 16 h. The reaction mixture was then quenched with 10% sodium bisulfite solution in water (20 mL) at 0 °C and the aqueous layer was extracted with diethyl ether (20 mL x 3). The combined organic layer was washed with saturated Na_2CO_3 solution in water (20 mL x 2) and dried over anhydrous $MgSO_4$ and then filtered. The resulting filtrate was concentrated under reduced pressure and purified by column chromatography on silica gel (hexane/ethyl acetate, 1/1) to give *anti*-**24** (621 g, 65% for two steps) as colorless solid, m.p. 91 – 92 °C; ^{1}H NMR (600 MHz, $CDCl_3$): δ 7.67–7.59 (m, 2H), 7.58–7.53 (m, 2H), 7.46 (t, J = 7.7 Hz, 2H), 7.41–7.34 (m, 1H), 7.29 (d, J = 7.9 Hz, 2H), 3.21 (dt, J = 6.2, 2.6 Hz, 1H), 2.88–2.67 (m, 2H), 2.15 (s, 1H), 1.67–

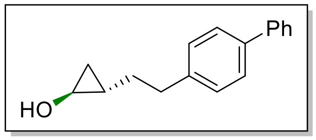

1.56 (m, 1H), 1.54–1.45 (m, 1H), 1.10–0.94 (m, 1H), 0.83–0.63 (m, 1H), 0.38 (q, J = 6.0 Hz, 1H); ^{13}C NMR (150 MHz, $CDCl_3$): δ 141.4, 141.2, 138.8, 128.9, 128.81, 127.11 (2C), 127.07, 52.9, 35.0, 33.6, 20.7, 14.5; HRMS (ESI, m/z): calcd. for $C_{17}H_{17}ONa$ $[M+Na]^+$: 260.1177; found 260.1179.

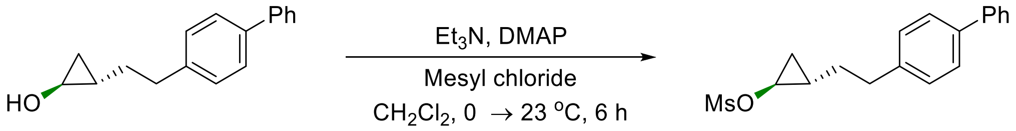

anti-**24** *anti*-**25**, 87%

2-{2-[(1,1'-Biphenyl)-4-yl]ethyl}cyclopropyl methanesulfonate (Fig. 4, *anti*-**25**, Reaction time: 6 h, 87%**)**. Employed *anti*-**24** (119 mg, 0.5 mmol), mesyl chloride (69 mg, 0.6 mmol), triethylamine (Et_3N, 152 mg, 1.5 mmol), and 4-dimethylaminopyridine (DMAP, 0.6 mg, 1.0 mol%) in CH_2Cl_2 (4 mL) at 0 °C. After completion of the reaction, the crude solution was stirred at 23 °C. After 6 h, the crude reaction mixture was quenched by adding water (3 mL) and the reaction mixture was extracted with CH_2Cl_2 (3 mL x 3). The combined organic layers were then dried over Na_2SO_4, evaporated under reduced pressure, and purified by flash column chromatography on silica gel (hexane/ethyl acetate, 7/3) to give *anti*-**25** (137 mg, 87%) as brown solid. m.p. 63 – 65 °C; ^{1}H NMR (600 MHz, $CDCl_3$): δ 7.59 (d, J = 7.8 Hz, 2H), 7.53 (d, J = 8.2 Hz, 2H), 7.44 (t, J = 7.7 Hz, 2H), 7.36–7.31 (m, 1H), 7.27 (d, J = 7.8 Hz, 2H), 4.08–3.72 (m, 1H), 2.98 (s, 3H), 2.79 (t, J = 7.6 Hz, 2H), 1.71–1.53 (m, 2H), 1.42–1.31 (m, 1H), 1.16–1.06 (m, 1H), 0.66 (q, J = 6.7 Hz, 1H); ^{13}C NMR (150 MHz, $CDCl_3$): δ 140.9, 140.4, 138.9, 128.8, 128.7, 127.1, 127.1, 126.9, 58.7, 37.6, 34.3, 32.7, 18.1, 12.1; HRMS (ESI, m/z): calcd. for $C_{18}H_{20}NaO_3S$ $[M+Na]^+$: 339.1031; found: 339.1024.

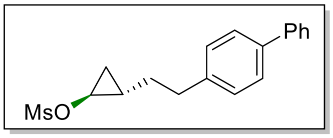

Supplementary References

1. Liu, Y. & Du, H. Metal-free borane-catalyzed highly stereoselective hydrogenation of pyridines. *J. Am. Chem. Soc.* **135,** 12968–12971 (2013).
2. Parks, D. J., Piers, W. E. & Yap, G. P.A. Synthesis, properties, and hydroboration activity of the highly electrophilic borane bis(pentafluorophenyl)borane, $HB(C_6F_5)_2$. *Organometallics* **17,** 5492–5503 (1998).
3. Chambers, R. D. & Chivers, T. Polyfluoroaryl organometallic compounds. Part II. Pentafluorophenylboron halides and some derived compounds. *J. Chem. Soc.* 3933–3939 (1965).
4. Chambers, R. D. & Chivers, T. Polyfluoroaryl organometallic compounds. Part I. Pentafluorophenyl derivatives of tin. *J. Chem. Soc.* 4782–4790 (1964).
5. Parks, D. J., Spence, R. E. von H. & Piers, W. E. Bis(pentafluorophenyl)borane: Synthesis, properties, and hydroboration chemistry of a highly electrophilic borane reagent. *Angew. Chem., Int. Ed.* **34,** 809–811 (1995).
6. Kuhl, N., Hopkinson, M. N. & Glorius F. Selective Rhodium(III)-catalyzed cross-dehydrogenative coupling of furan and thiophene derivatives. *Angew. Chem., Int. Ed.* **51,** 8230–8234 (2012).
7. Haner, J., Jack, K., Menard, M. L., Howell, J., Nagireddy, J., Raheem, M. A. & Tam, W. W. Synthesis of C1-substituted oxabenzonorbornadienes. *Synthesis* **44,** 2713–2722 (2012).
8. Paredes, M. D. & R. Alonso. On the radical brook rearrangement: Reactivity of α-silyl alcohols, α-silyl alcohol nitrite esters, and β-haloacylsilanes under radical-forming conditions. *J. Org. Chem.* **65,** 2292–2304 (2000).
9. Kim, J., Hewitt, G., Carroll, P. & Sieburth, S. McN. Silanediol inhibitors of angiotensin-converting enzyme: Synthesis and evaluation of four diastereomers of Phe[Si]Ala dipeptide analogues. *J. Org. Chem.* **70,** 5781–5789 (2005).
10. Trost, B. M., Papillon, J. P. N. & Nussbaumer, T. Ru-catalyzed alkene−alkyne coupling: Total synthesis of amphidinolide P. *J. Am. Chem. Soc.* **127,** 17921–17937 (2005).
11. Barry, C. S. J., Crosby, S. R., Harding, J. R., Hughes, R. A., King, C. D., Parker, G. D. & Willis, C. L. Stereoselective synthesis of 4-hydroxy-2,3,6-trisubstituted tetrahydropyrans. *Org. Lett.* **5,** 2429–2432 (2003).
12. Yadav, J. S., Reddy, M. S. & Prasad, A. R. Stereoselective synthesis of polyketide precursors containing an *anti*-1,3-diol system via a Prins cyclisation and reductive cleavage sequence. *Tetrahedron Lett.* **47,** 4937–4941 (2006).
13. Chatterjee, A. K., Choi, T.-L., Sanders, D. P. & Grubbs, R. H. A General model for selectivity in olefin cross metathesis. *J. Am. Chem. Soc.* **125,** 11360–11370 (2003).
14. Kim, A. I., Kimmel, K. L., Romero, A., Smitrovich, J. H., & Woerpel, K. A. Mechanistic studies of the allylic rearrangements of α-silyloxy allylic silanes to silyloxy vinylic silanes. *J. Org. Chem.* **72,** 6595–6598 (2007).
15. Reich, H. J., Eisenhart, E. K., Olson, R. E. & Kelly, M. J. Silyl ketone chemistry: Preparation and reactions of silyl allenol ethers. Diels-Alder reaction of siloxy vinylallenes leading to sesquiterpenes. *J. Am. Chem. Soc.* **108,** 7791–7800 (1986).
